# Genome-wide analysis and expression profiling under heat and drought treatments of *HSP70* gene family in soybean (*Glycine max* L.)

**DOI:** 10.3389/fpls.2015.00773

**Published:** 2015-09-25

**Authors:** Ling Zhang, Hong-Kun Zhao, Qian-Li Dong, Yuan-Yu Zhang, Yu-Min Wang, Hai-Yun Li, Guo-Jie Xing, Qi-Yun Li, Ying-Shan Dong

**Affiliations:** ^1^Agro-Biotechnology Research Institute, Jilin Academy of Agricultural SciencesChangchun, China; ^2^Crop Germplasm Institute, Jilin Academy of Agricultural SciencesGongzhuling, China; ^3^Department of Biology, Beijing Normal UniversityBeijing, China; ^4^Institute of Plant Protection, Jilin Academy of Agricultural SciencesGongzhuling, China

**Keywords:** soybean (*Glycine max* L.), *HSP70* gene family, genome-wide analysis, phylogeny, gene structure, expression pattern

## Abstract

Heat shock proteins (HSPs) perform a fundamental role in protecting plants against abiotic stresses. Previous studies have made great efforts in the functional analysis of individual family members, but there has not yet been an overall analysis or expression profiling of the *HSP70* gene family in soybeans (*Glycine max* L.). In this study, an investigation of the soybean genome revealed 61 putative *HSP70* genes, which were evaluated. These genes were classified into eight sub-families, denoted I–VIII, based on a phylogenetic analysis. In each sub-family, the constituent parts of the gene structure and motif were relatively conserved. These *GmHSP70* genes were distributed unequally on 17 of the 20 chromosomes. The analysis of the expression profiles showed that 53 of the 61 *GmHSP70* genes were differentially expressed across the 14 tissues. However, most of the *GmHSP70s* were differentially expressed in a tissue-specific expression pattern. Furthermore, the expression of some of the duplicate genes was partially redundant, while others showed functional diversity. The quantitative real-time PCR (qRT-PCR) analysis of the 61 soybean *HSP70* genes confirmed their stress-inducible expression patterns under both drought and heat stress. These findings provide a thorough overview of the evolution and modification of the *GmHSP70* gene family, which will help to determine the functional characteristics of the *HSP70* genes in soybean growth and development.

## Introduction

Heat shock proteins (HSPs) are a group of proteins induced by heat shock, and they are found in virtually all living organisms, from bacteria to humans (De Maio, 1999). In plants, the *HSP* gene family members play important roles in developmental processes, as well as different kinds of environmental stress conditions, such as heat and drought (Swindell et al., 2007; Cho and Choi, 2009), low and high temperatures (Krishna et al., 1995; Sabehat et al., 1998; Lopez-Matas et al., 2004), salinity (Zou et al., 2012), and heavy metals (Kim et al., 2014). HSPs have recently been discovered to be associated with plant responses to infection by pathogens such as nematodes (Maimbo et al., 2007). Based on their molecular weight, HSPs are classified into five major families: sHSP, small heat shock protein; HSP60, chaperonin family; HSP70, 70-kDa-heat shock protein; HSP90 and HSP100 family (Wang et al., 2004). Of these, the HSP70 family is of greatest interest, as it is fundamental to plant developmental processes and functions during heat stress (Sung et al., 2001).

HSP70 proteins are central components of the cellular network of molecular chaperones and folding catalysts (Mayer and Bukau, 2005), and were first identified and characterized in the early 1960s (Ritossa, 1996). HSP70s are comprised of two major functional domains. One is a conserved ~44-kD N-terminal ATPase domain (NBD), which is also called the nucleotide binding domain and the other is a ~18-kD substrate binding domain (SBD) with a ~10-kD variable C-terminal “lid” (Dragovic et al., 2006). The diverse biological function of the *HSP70* gene family has been well characterized in many plants. For example, in *Arabidopsis*, the *cpHsc70-1* mutant plants exhibited defective phenotypes after germinated seeds were treated with heat stress (Su and Li, 2008), and *AtHSP70-15* was shown to play an essential role during heat response (Jungkunz et al., 2011). HSP70s have also been reported to play roles in response to heat stress in rubber trees (*Hevea brasiliensis*) (Zhang et al., 2009), wheat (*Triticum aestivum* L) (Francki et al., 2002; Duan et al., 2011), pepper (*Capsicum annuum* L) (Guo et al., 2014), and cucumber (*Cucumis sativus*) (Li et al., 2014). HSP70 also shows complex developmental regulation during the vegetative and reproductive phases of growth. In tomato, for example, *HSP70* transcripts were detected in mature anthers (Duck and Folk, 1994). In alfalfa (*Medicago sativa*), the *MsHSP70-1* plays an important role in the development of alfalfa nodules (He et al., 2008). Recently, some studies have suggested that the HSP70 family members may play various roles in plant RNA virus multiplication, such as viral protein folding, virion assembly and protein expression (Nagy et al., 2011). Among the plant (+) RNA viruses, it has been reported that HSP70 participates in the assembly of the replicase complexes of the TBSV (Hafrén et al., 2010; Mathioudakis et al., 2012), TuMV (Dufresne et al., 2009; Wang et al., 2009a), and rice stripe virus (Wang et al., 2009b). These results indicate that the HSP70 family members are multi-functional in the life cycle of plants (Friedman and Brandon, 2001; Jiang et al., 2014).

Soybean (*Glycine max* L.) is one of the most economically and nutritionally crucial crops in the world. It provides not only vegetable protein and edible oil, but also essential amino acids for humans and animals. However, soybean production is threatened by drought, as well as other environmental stresses. With the sequencing of the soybean genome (Schmutz et al., 2010), a few members of the HSP family were characterized in this species (Jinn et al., 1995; Fuganti et al., 2010; Mohammadi et al., 2012; Liu and Whitham, 2013; Lopes-Caitar et al., 2013; Wang et al., 2014). However, most of their functions remain to be determined. There remains no genome-wide characterization of the HSP70 family in soybean to date.

In our study, all of the non-redundant sets of the soybean *HSP70* genes were scanned and integrated, and we also determined their chromosomal locations, predicted their protein structures with available software and network stations, and analyzed the expression levels of the soybean *HSP70* genes using qRT-PCR in order to determine their tolerance to drought and heat stresses. Our findings will be useful resources for future studies to unravel the functions of the *GmHSP70* genes and will contribute to our understanding of the evolutionary history of the *HSP70* genes in different species.

## Materials and methods

### Genome-wide identification of HSP70 proteins in soybean

The whole soybean genome sequence was downloaded from the annotation database (DB) Phytozome v9.1 (Joint Genome Institute, JGI). To gather the probable candidate soybean HSP70 amino acid sequences, the HMM profile of the HSP70 domain was first downloaded from the Pfam (http://www.sanger.ac.uk/Software/Pfam/) database (Pfam: PF00012) and then submitted as a query in a BLASTP (*P* = 0.001) search of the soybean genome database (Finn et al., 2014). All obtained protein sequences were examined for the presence of the HSP70 domain by SMART (http://smart.embl-heidelberg.de/) tools (Letunic et al., 2004).

Soybean *HSP70* gene information, including the locations on the chromosomes, genomic sequences, full CDS sequences, protein sequences, and 2000 bp of the nucleotide sequences upstream of the translation initiation codon were downloaded from the website Phytozome (www.phytozome.net). The molecular weight (Da) and isoelectric point (pI) of each gene were calculated using compute pI/Mw tool from ExPASy (http://www.expasy.org/tools/) (Gasteiger et al., 2003).

### Subcellular localization and promoter analysis of the *GMHSP70* family

Subcellular localization was predicted using the WoLF PSORT (http://www.genscript.com/psort/wolf_psort.html) (Horton et al., 2007) and TargetP 1.1 (http://www.cbs.dtu.dk/services/TargetP/) (Emanuelsson et al., 2007). The 5′ upstream region, including a 2000 bp DNA sequence of each gene of the *GmHSP70* family, was subjected to the plantCARE database (http://bioinformatics.psb.ugent.be/webtools/plantcare/html/) for a *cis*-element scan.

### Multiple alignment and phylogenetic analysis

We constructed two phylogenetic trees to classify the soybean *HSP70* genes in this paper. The first one contained only soybean HSP70 protein sequences and the second included also *Arabidopsis thaliana* and rice HSP70 sequences. The gene sequences and protein sequences of *Arabidopsis* and rice HSPs were acquired from TAIR (Lamesch et al., 2012), and TIGR (Yuan et al., 2003), respectively. Multiple sequence alignment of all predicted HSP70 protein sequences were performed with Clustal X2.0 software using default parameters. The phylogenetic trees were constructed using MEGA 5.0 with the Neighbor-Joining (NJ) method, and a bootstrap analysis was conducted using 1000 replicates with a pairwise gap deletion mode (Tamura et al., 2011).

### Gene structure analysis and identification of conserved motifs

To investigate the diversity and structure of members of the *GmHSP70* gene family, we compared the exon/intron organization between the cDNA sequences with the corresponding genomic DNA sequences of *HSP70* by using Gene Structure Display Server (GSDS; http://gsds.cbi.pku.edu.cn/) (Guo et al., 2007). In addition, their amino acid sequences were subjected to “predict the domain and motif analyses” online with MEME (http://meme.sdsc.edu/meme/website/intro.html) (Bailey et al., 2006). The parameters were as follows: number of repetitions: any; maximum number of motifs: 10; and optimum motif widths: 6 to 200 amino acid residues.

### Chromosomal location and gene duplication

The chromosomal location image of the *GmHSP70* genes was generated by MapChart software, according to the chromosomal position information provided in the Phytozome database. In order to identify the tandem and segmental duplications, two genes in the same species, located in the same clade of the phylogenetic tree, were defined as being coparalogs. The SoyBase browser (http://soybase.org/gb2/gbrowse/gmax1.01/) was queried in order to detect the segmental duplication coordinates of the target genes (Grant et al., 2010). The coparalogs were deemed be the results of segmental duplication if they were located on duplicated chromosomal blocks (Wei et al., 2007). The paralogs were deemed to be tandem duplicated genes if two genes were separated by five or fewer genes in a 100 kb region (Wang et al., 2010). The local alignment of two protein sequences was calculated using the Smith-Waterman algorithm (http://www.ebi.ac.uk/Tools/psa/).

### RNA-seq atlas analysis

To acquire the tissue-specific transcript data, the 61 *GmHSP70* genes were investigated based on the RNA Seq-Atlas from fourteen tissues (http://soybase.org/soyseq/), including underground tissues (root and nodule), seed development (seed 10-DAF, seed 14-DAF, seed 21-DAF, seed 25-DAF, seed 28-DAF, seed 35-DAF, and seed 42-DAF) and aerial tissues (young leaf, flower, pod-shell 10-DAF, pod-shell 14-DAF, and 1 cm pod). The expression data were gene-wise normalized and the heat map was drawn using MeV v4.8 software (http://www.tm4.org/) (Mar et al., 2011).

### Plant growth and stress treatments

Soybean Williams 82 seeds were germinated in vermiculite in a light chamber at 25 ± 2°C for about 2 weeks. For heat stress, the seedlings were transferred to a growth chamber at 42 ± 1°C, while for drought stress, 15% (w/v) PEG3000 was used. The leaves of heat-stressed and drought-stressed plants were collected at 0, 3, 6, 12, and 24-h intervals. After collection, the samples were immediately frozen in liquid N_2_, and stored at −80°C for RNA extraction. Three biological replicates were obtained per sample.

### RNA extraction and qRT-PCR analysis

The total RNA from all of the samples was isolated using Trizol reagent (Invitrogen, USA), followed by DNaseI (Promega, USA) treatment for the purpose of removing any traces of genomic DNA. The first-strand cDNA synthesis was performed using the TransScript First-Strand cDNA Synthesis SuperMix Kit (TransGen, China). Gene-specific primers for the 61 *GmHSP70* genes were designed using Primer 5.0 (Additional File 13). A housekeeping gene constitutively expressed in soybean, Actin (LOC100792119), was used as a reference for normalization. The real-time quantitative reverse transcription-polymerase chain reaction (PCR) was conducted using an Applied Biosystems StepOne Real-Time PCR System (Applied Biosystems, USA). The reaction volume consisted of 10 μL SYBR premix Ex Taq™ (2×) mixture, 1 μL cDNA (diluted 10 times), 0.4 μL upstream primer (10 pM), 0.4 μL downstream primer (10 pM) and 8.2 μL ddH_2_O (20 μL in total). The reaction was performed with the following cycling profile: 95°C for 30s, 40 cycles at a denaturation of 95°C for 5s, and 60°C for 30s. Three technical replicates were performed for each sample. The calculation of the gene expression levels followed the 2^−ΔΔ*CT*^ method described by Livak and Schmittgen (2001).

## Results

### Identification of the *HSP70* gene family in soybean

A soybean genome blast, as well as online software identification, were utilized to identify a total of 61 soybean *HSP70* genes based on their nomenclature (Table 1). All of the 61 members contained the domains PF00012, based on Pfam and SMART tests. The proteins that had only one of these domains, or that did not have an integral open reading frame, were excluded. Then, the protein sequences (Additional File 1), coding sequences (CDS) (Additional File 2), genomic sequences (Additional File 3), and 2000 bp of the nucleotide sequences upstream from the translation initiation codon (Additional File 4) were downloaded from the Phytozome database (www.phytozome.net). The basic information of all of the soybean *HSP70* genes (including gene name, chromosome location, ORF length, exon and intron number, protein length, molecular weight and pI value) is provided in Table 1. The *GmHSP70s* encoded proteins varied from 134 to 924 amino acids (aa) in length. Among these proteins the Glyma18g13077 protein sequence was the shortest, with 134 amino acids. The Glyma20g16070 was the longest protein sequence, with 927 amino acids. The predicted molecular weights of the *GmHSP70* candidates were distributed in a range from 15188 Da (Glyma18g13077, 134aa) to 102932 Da (Glyma20g16070, 927aa). The predicted isoelectric points (pI) of the *GmHSP70* candidates were between 4.89 (Glyma05g03770) and 9.94 (Glyma18g13077). The predicted protein instability indices showed that only 19 of the 61 *GmHSP70* candidates could be considered stable proteins (cutoff < 40), according to the ExPASy analysis.

**Table 1:** **List of 61 ***HSP70*** genes identified in soybean, their sequence characteristics and subcellular localization**.

**No**.	**Feature name**	**Chr**.	**Location coordinates (5′–3′)**	**ORF length (bp)**	**Exons**	**Protein**			**Subcellular location**	
						**Length (aa)**	**MW (Da)**	**pI**	**WolF PSPORT**	**TargetP**
1	Glyma01g44910	1	55378502–55381508	2119	1	571	62044.6	5.48	chlo: 5, cyto: 5, nucl: 1, mito: 1, plas: 1	–
2	Glyma02g09400	2	7365311–7372106	3335	4	892	99415.7	6.99	cyto: 7, nucl: 4, chlo: 1, plas: 1	–
3	Glyma02g10195	2	8079000–8079761	636	3	211	23815.1	4.9	chlo: 5, cyto: 5, nucl: 2.5, cysk_nucl: 2	–
4	Glyma02g10261	2	8155483–8156483	843	2	281	30817.7	6.21	cyto: 7, nucl: 2.5, pero: 2, cysk_nucl: 2, chlo: 1	–
5	Glyma02g10320	2	8186068–8188789	2115	2	625	68722.9	5.19	Unknown	–
6	Glyma02g36700	2	42135636–42138080	2445	1	652	71626.2	5.2	cyto: 11, chlo: 1, nucl: 1	–
7	Glyma03g03250	3	3021324–3034627	1968	14	490	53668.1	9.41	chlo: 10, mito: 4	–
8	Glyma03g17870	3	22495770–22499239	1756	6	384	42339.7	5.18	cyto: 8, nucl: 4, nucl_plas: 3.5	–
9	Glyma03g32850	3	40584885–40588047	2525	2	653	71474.9	5.04	cyto: 8, cysk: 4, chlo: 1	–
10	Glyma05g03770	5	2918810–2923984	1665	12	400	43129.2	4.89	nucl: 13	–
11	Glyma05g15130	5	16402621–16403816	417	4	139	15506.9	8.54	Unknown	–
12	Glyma05g36600	5	40426889–40430895	2594	8	701	77640.3	5.49	chlo: 6, mito: 3, cyto: 2, plas: 1, E.R.: 1	M 0.437/5
13	Glyma05g36620	5	40443107–40447303	2532	8	668	73639.5	5.08	E.R.: 8, chlo: 2, golg: 2, extr: 1	S 0.980/1
14	Glyma06g00310	6	109523–111629	1722	6	573	63305.6	5.51	Unknown	–
15	Glyma07g00820	7	445423–451250	3493	10	857	94777.3	5.27	cyto: 9, nucl: 3, chlo: 2	–
16	Glyma07g02450	7	1667004–1668640	1194	7	398	42974.5	5.5	Unknown	–
17	Glyma07g26550	7	29388810–29393550	2366	3	633	70529.8	5.69	cyto: 8, chlo: 3, nucl: 2	–
18	Glyma07g30290	7	35323611–35327243	2418	6	677	72469	5.82	mito: 11, chlo: 3	M 0.793/4
19	Glyma07g32921	7	37780575–37781764	1173	2	390	43148.6	6.27	nucl: 5, cyto: 5, chlo: 2, cysk: 2	–
20	Glyma08g02940	8	2029878–2033833	2403	8	667	73477.3	5.08	E.R.: 8, chlo: 2, golg: 2, extr: 1	S 0.981/1
21	Glyma08g02960	8	2043506–2047440	2560	8	708	78058.6	5.14	cyto: 7.5, cyto_nucl: 4.5, E.R.: 4, chlo: 1	–
22	Glyma08g06950	8	4980953–4984577	2452	6	677	72554.1	5.82	mito: 12, chlo: 2	M 0.793/4
23	Glyma08g22100	8	16779202–16784587	3152	10	852	94038.5	5.22	cyto: 9, chlo: 3, nucl: 2	–
24	Glyma08g42720	8	42703873–42710339	3193	10	769	86037.9	5.71	nucl: 7, cyto: 2, cysk: 2, chlo: 1, vacu: 1	–
25	Glyma11g14950	11	10696044–10699039	2426	2	649	71081.4	5.1	cyto: 8, cysk: 4, chlo: 1	–
26	Glyma11g31670	11	33019748–33021169	1163	6	386	43590.2	9.08	Unknown	–
27	Glyma11g31673	11	33019957–33021200	963	4	320	35684.1	8.57	chlo: 10, cyto: 2, mito: 1	–
28	Glyma11g31810	11	33165094–33173107	2074	14	429	47122.5	6.36	chlo: 14	C 0.330/5
29	Glyma12g06910	12	4706699–4709528	2324	2	649	71087.5	5.1	cyto: 8, cysk: 4, chlo: 1	–
30	Glyma12g28750	12	32105319–32107516	1666	3	432	45506.7	5.51	chlo: 10, mito: 4	C 0.774/4
31	Glyma13g10700	13	12773269–12782190	3387	13	891	99038.5	5.33	plas: 11, chlo: 1, vacu: 1	S 0.974/1
32	Glyma13g19330	13	22884387–22887412	2328	2	385	42851.2	8.45	cysk: 8, cyto: 5	–
33	Glyma13g19331	13	22884740–22887383	1899	3	632	69269.4	5.13	cyto: 9, cysk: 4	–
34	Glyma13g29580	13	32473031–32474833	1584	3	527	59206.9	8.63	cyto: 9, chlo: 3, nucl: 1	–
35	Glyma13g29590	13	32478782–32481232	2343	3	547	60742	7.86	chlo: 5, cyto: 5, mito: 2, plas: 1	M 0.525/4
36	Glyma13g29591	13	32478808–32481336	1776	3	591	66326.7	8.81	Unknown	–
37	Glyma13g32790	13	34852495–34856172	2657	6	674	72428	5.68	mito: 14	M 0.863/2
38	Glyma13g43630	13	43249474–43254319	3169	10	863	95459.1	5.12	cyto: 12, chlo: 2	–
39	Glyma14g02740	14	1726618–1732679	3066	10	773	85879.5	5.5	nucl: 9, cyto: 3, chlo: 1	–
40	Glyma15g01750	15	1153804–1159313	3326	10	863	95720.5	5.13	cyto: 11, chlo: 2	–
41	Glyma15g06530	15	4615125–4618773	2623	6	674	72470.1	5.78	mito: 14	M 0.863/2
42	Glyma15g09420	15	6732693–6735395	1749	2	583	65066.9	6.57	cyto: 9, chlo: 3, plas: 2	–
43	Glyma15g09430	15	6739540–6741346	1758	2	585	65290.7	6.77	cyto: 7, chlo: 3, mito: 3	–
44	Glyma15g10280	15	7459804–7461615	1539	5	512	57064.6	8.71	Unknown	–
45	Glyma16g00410	16	136433–139838	2695	7	689	73754.4	5.2	chlo: 13	C 0.869/2
46	Glyma17g08020	17	5928339–5930881	2431	2	645	70865.5	5.27	cyto: 12, chlo: 2	–
47	Glyma17g11650	17	8745134–8748031	1848	4	352	38531.3	5.56	cyto: 9, chlo: 4	–
48	Glyma17g14280	17	11034223–11039394	1704	11	403	43564.7	4.93	nucl: 13	–
49	Glyma18g05480	18	4147180–4155147	2015	14	432	47360.6	6.06	plas: 3, E.R.: 3, chlo: 2, extr: 2, nucl: 1, cyto: 1, mito: 1	–
50	Glyma18g05610	18	4218455–4221721	1404	6	467	52282.7	6.01	cyto: 5, cysk: 4, nucl: 2, chlo: 1, plas: 1	–
51	Glyma18g11520	18	10253358–10259654	3064	10	766	85879.7	5.57	nucl: 7, cyto: 2, cysk: 2, chlo: 1, vacu: 1	–
52	Glyma18g13077	18	12516456–12526090	1351	8	134	15188	9.94	chlo: 9, mito: 5	C 0.826/2
53	Glyma18g52470	18	61065907–61071752	2311	4	710	79186.1	6.22	cyto: 10, chlo: 4	–
54	Glyma18g52471	18	61067504–61071806	2476	2	673	74813.3	6.06	cyto: 5, plas: 3, E.R.: 3, cysk: 3	–
55	Glyma18g52480	18	61075242–61082432	2564	2	673	74710.1	5.85	cyto: 7, chlo: 5, nucl: 2	–
56	Glyma18g52610	18	61172753–61175746	2566	2	649	71232.7	5.09	cyto: 9, cysk: 3, chlo: 1	–
57	Glyma18g52650	18	61203298–61206577	2419	3	647	70839.1	5.1	cyto: 7, cysk: 5, chlo: 1	–
58	Glyma18g52760	18	61262307–61264485	1797	3	598	66820.5	6.74	chlo: 10, nucl: 1, cyto: 1, vacu: 1	–
59	Glyma19g35560	19	43115295–43118308	2390	2	654	71499.9	5.04	cyto: 8, cysk: 4, chlo: 1	–
60	Glyma19g44140	19	49648396–49650414	1046	4	352	38501.3	5.88	cyto: 8, chlo: 4, nucl: 1	–
61	Glyma20g16070	20	22168917–22180179	3215	14	927	102932.2	5.52	plas: 9, chlo: 2, cyto: 2	M 0.535/4

*bp, base pair; aa, amino acids; Da, Dalton*.

*WoLF PSORT predictions: chlo, chloroplast; cyto, cytosol; nucl, nucleus; E.R., endoplasmic reticulum; mito, mitocondria; plas, plasma membrane; extr, extracelular; cysk, cytoskeleton; plas, plasma membrane; vacu, vacuolar membrane*.

*TargetP predictions: C, chloroplast; M, mitochondrion; S, secretory pathway;—(any other location); values indicate score (0.00–1.00) and reliability class (1–5; best class is 1)*.

### Subcellular localization and *cis*-acting elements of *GmHSP70* family

The Wolf PSORT assessment revealed that within the 61 *GmHSP70* proteins, 27 soybean HSP70 proteins were localized to the cytosol, 11 to the chloroplast, six to the nucleus, four to the mitochondria, three to the plasma membrane, two to the endoplasmic reticulum, one to the cytoskeleton, and seven were not identified by WoLF PSORT. The TargetP analysis revealed that seven soybean HSP70 proteins were located in the mitochondria, four in the chloroplast, and three in the secretory pathway and 47 in other compartments. These detailed parameters are provided in Table 1.

Plant gene promoter, an important *cis*-acting element that is located upstream of the start codon region, is the control center of gene transcription. Our *cis*-acting element analysis identified a total of 115 types of *cis*-regulatory elements in the promoter regions of *GmHSP70s* (see Additional Files 5, 6). These *cis*-regulatory elements could be broadly divided into eight categories: Light responsive elements, Promoter related elements, Hormone responsive elements, Environmental stress-related elements, Development related elements, Site-binding related elements and other elements). The light responsive category contains 42 kinds of *cis*-regulatory elements and accounts for the largest proportion in the total. Twelve types of *cis*-regulatory elements were found to be involved in plant hormone responsiveness, which cover most types of hormone, including ABRE, abscisic acid responsive element; ERE, ethylene responsive element; GARE-motif, p-box, gibberellins responsive element; TCA-element, salicylic acid responsiveness; TGA-element, auxin-responsive element; CGTCA-motif, TATC-box, TGACG-motif, the MeJA-responsiveness; and AuxRR-core, auxin responsiveness element. The third category contained 11 types of *cis*-regulatory elements that might respond to environmental stress, such as heat stress, Box-W1 and W3, fungal elicitor responsive element; MBS, MYB binding site involved in drought-responsiveness; LTR, low-temperature responsiveness; and W-box, wound-responsive element. The flowing category is related to plant growth and development, and contains 15 types of *cis*-regulatory elements: AACA-motif, AAGAA-motif, AC-I, AC-II, CAT-box, CCAAT-motif, O2-site, as1, circadian, GCN4-motif, RY-element, Skn-1_motif, HD-Zip1, HD-Zip2, and MSA-like. Two categories were promoter related elements and site-binding related elements; they contained eight and seven types of *cis*-regulatory elements, respectively. Finally, the functions of the remaining 20 types of *cis*-regulatory elements are unclear (Additional File 6).

### Phylogenetic, gene structure, and motif analysis of the HSP70 proteins in soybean

**Figure 1:**
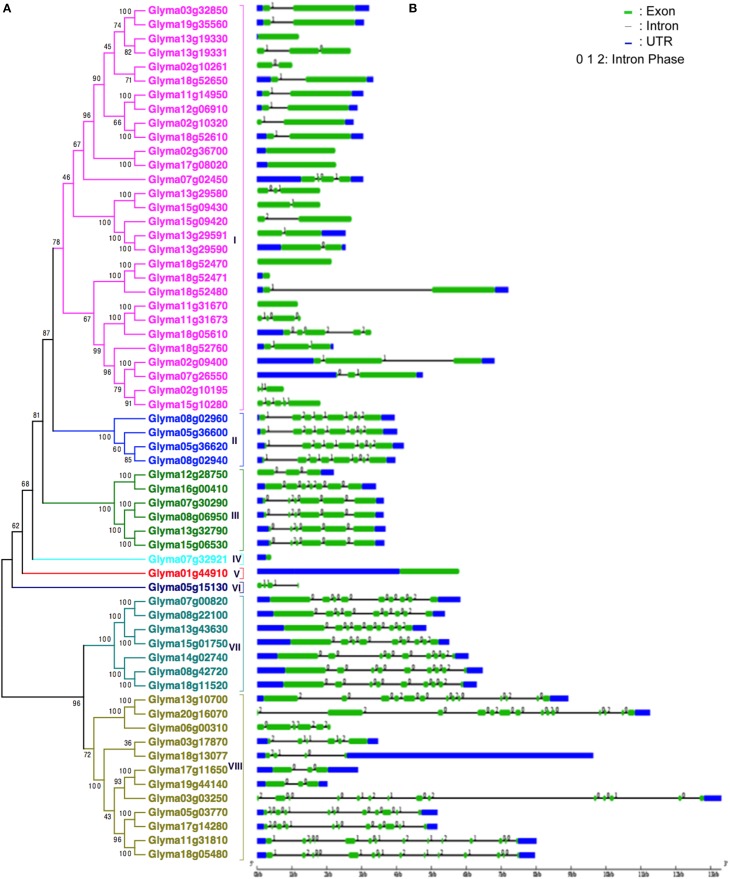
**Phylogenetic relationships and gene structure of soybean ***HSP70*** genes**. **(A)** The unrooted tree was generated with the MEGA5.0 program using the full-length amino acid sequences of the 61 soybean HSP70 proteins by the Neighbor-Joining (NJ) method, with 1000 bootstrap replicates. Subfamilies of *HSP70* genes (I–VIII) are highlighted with different colored backgrounds and vertical bars next to the gene names of the tree. **(B)** Exon/intron organization of soybean *HSP70* genes. Green boxes represent exons and black lines represent introns. The untranslated regions (UTRs) are indicated by blue boxes. The numbers 0, 1, and 2 represents the splicing phases. The sizes of exons and introns can be estimated using the scale at the bottom.

**Figure 2:**
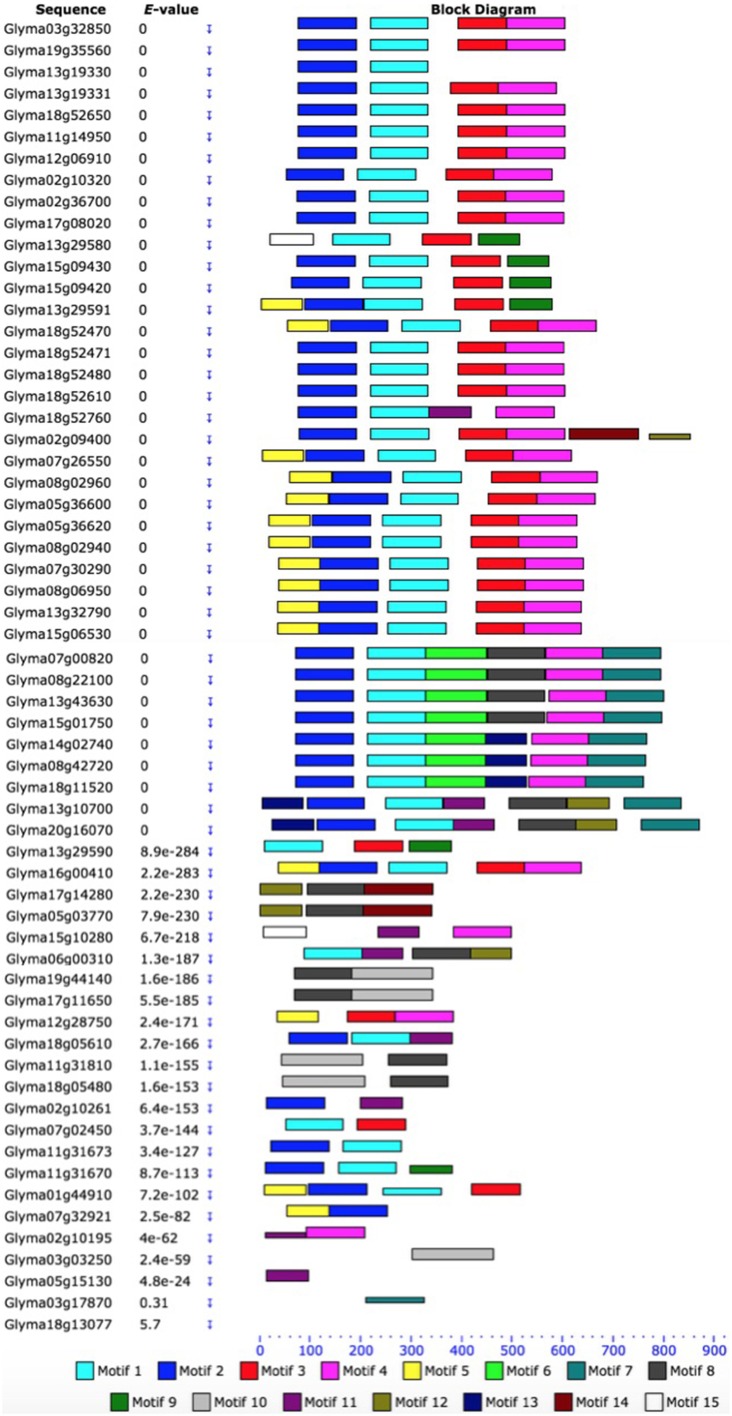
**Schematic representation of the conserved motifs in soybean HSP70 proteins elucidated from publicly available data**. Each colored box represents a motif in the protein, with the motif name indicated in the box on the right. The length of the protein and motif can be estimated using the scale at the bottom. The scale at the top of the image may be used to estimate motif length. aa, amino acids. A detailed motif introduction is shown in Additional File 7 and 8.

Based on the unrooted phylogenetic tree of the 61 *GmHSP70* protein sequences, the soybean *HSP70* gene family was categorized into eight major sub-families (sub-family I, II, III, IV, V, VI, VII, and VIII). Sub-family I (containing 29 members) was the largest group, followed by sub-family VIII (12), sub-family VII (7), sub-family III (6), and sub-family II (4). The sub-families IV, V, VI were the smallest, with only one *GmHSP70* gene member each (Figure 1A). Correspondingly, Figure 1B provides a detailed illustration of the relative lengths of the introns, and the conservation of the corresponding exon sequences within each *HSP70* gene within the soybean. The number of introns in all of these genes ranged from 0 to 13. However, no intron was found in the *Glyma13g19330, Glyma02g36700, Glyma17g08020, Glyma18g52470, Glyma18g52471, Glyma11g31670, Glyma07g32921*, or *Glyma01g44910*, whereas 13 introns were found in the *Glyma20g16070, Glyma03g03250, Glyma11g31810*, and *Glyma18g05480*. The MEME of the total 61 *GmHSP70* genes were also analyzed (Figure 2). The results revealed the conserved domains or motifs shared among related proteins, and identified 15 conserved motifs (Additional Files 7 and 8). The type, order, and number of motifs were similar in proteins with the same sub-family, but differed from the proteins in other sub-families.

### Chromosomal location and gene duplication

**Figure 3:**
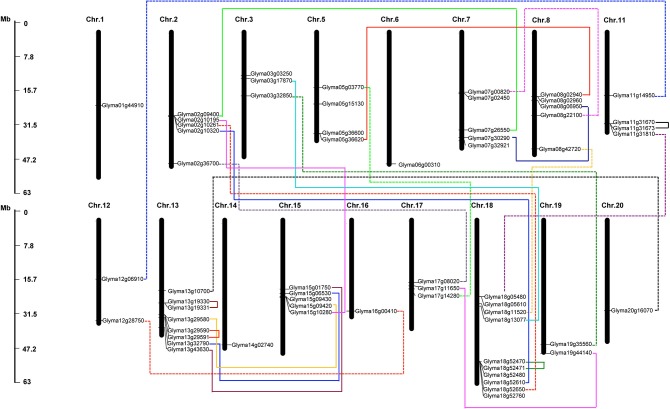
**Chromosomal map and duplication event coordinates of paralogous ***GmHSP70*** gene candidates**. The identity of each linkage group is indicated at the top of each bar. Only the chromosomes where *GmHSP70* genes were mapped are shown. Possible duplicated genes are connected by different color lines. The bar located on the left side indicates chromosome sizes in megabases. The scale represents the length of the chromosome.

The 61 putative *GmHSP70* gene candidates were distributed across 17 of the 20 chromosomes in the soybean genome. Among them, chromosome 18 had the highest density of *GmHSP70* genes, with 10 members; chromosome 13 had the next highest density, with eight genes; five *GmHSP70* genes were located in each of chromosomes 2, 7, 8, and 15; four *GmHSP70* genes were situated in chromosomes 5 and 11; three *GmHSP70* genes were positioned in chromosomes 3 and 17; two genes each were found in chromosomes 12 and 19; only one gene was found in each of chromosomes 1, 6, 14, 16, and 20; and no *GmHSP70* genes were detected in chromosome 3, 9, or 10 (Figure 3). We also investigated the gene duplication events of the *GmHSP70* family, and the results showed that there were 24 sister pairs in the 61 *GmHSP70* genes (Additional File 10). Only one pair of paralogous genes (*Glyma03g17870* and *Glyma18g13077*) did not reach this standard. The other 23 paralog pairs were all fitted for duplications of the *HSP70* genes. Four of the 23 *GmHSP70* paralog gene pairs were detected within a distance of less than 5 kb (>100 kb) on chromosomes 11, 13, and 18, which were identified as tandem duplications. The other 19-paralog gene pairs were segmental duplications. These results suggest that segmental duplication played a crucial role in the expansion of the *HSP70* gene family in soybeans.

### Comparative analysis of the *GmHSP70* genes in soybean, *Arabidopsis*, and rice

**Figure 4:**
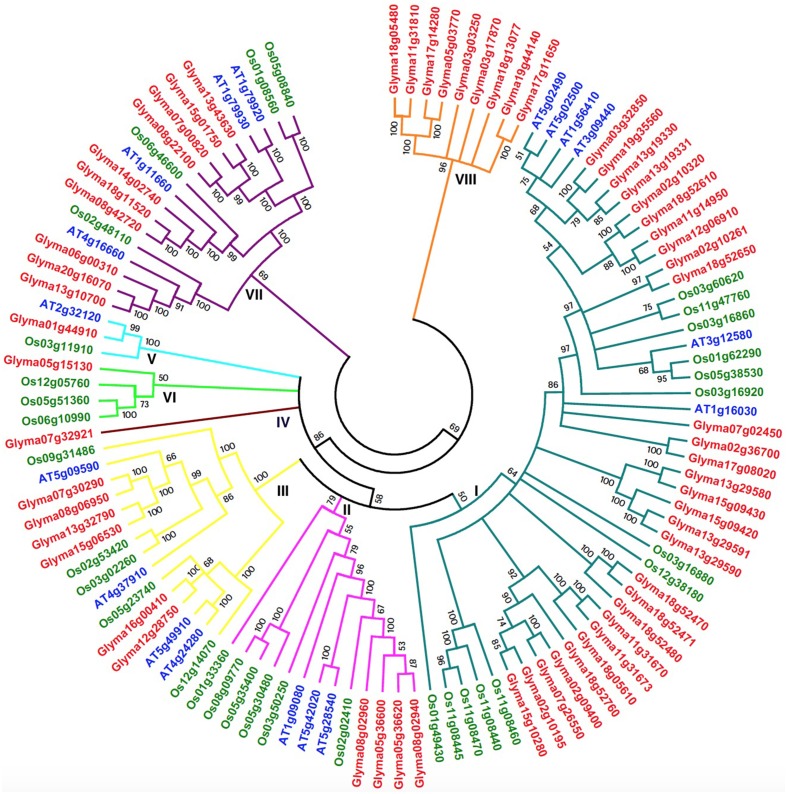
**Phylogenetic tree of full-length HSP70 proteins from soybean, ***Arabidopsis*** and rice**. The 61 soybean, 18 *Arabidopsis* and 32 rice HSP70 protein sequences were aligned by Clustal X 1.83 and the phylogenetic tree was constructed using MEGA5.0 by the Neighbor-Joining (NJ) method. The Bootstrap value was 1000 replicates. Each HSP70 subfamily is indicated by a specific color.

The development of comparative genomics has enabled the analysis of the same protein families among different species. The NJ phylogenetic tree was constructed using 111 full-length protein sequences in order to reveal the evolutionary relationships among soybean (61), *Arabidopsis* (18), and rice (32) HSP70 proteins (Figure 4 and Additional File 9) in our study. The 18 *Arabidopsis* HSP70 proteins were identified from the TAIR database, and, according to previous studies, the *AtHSP70* gene family is divided into five sub-families (Lin et al., 2001). There were 32 *OsHSP70* family members identified from the TIGR database, while rice contained six sub-families (Jung et al., 2013; Sarkar et al., 2013). Therefore, based on their phylogenetic relationships, the combined soybean, rice, and *Arabidopsis* phylogenetic trees can be divided into eight distinct sub-families (class I–VIII; Figure 4). Among the eight clusters, class I was the largest, containing 48 members, and composed of 29 members from soybean, 13 from rice, and 6 from *Arabidopsis*. Class VII was the second largest, containing 10 members from soybean, four from rice, and four from *Arabidopsis*. Class II contained 13 members (four soybean, six rice, and three *Arabidopsis* members). The HSP70 members in Class III were six soybean, five rice, and four in *Arabidopsis*. Class IV was a small sub-family, which only included one soybean *HSP70* gene (*Glyma07g32921*), and no rice or *Arabidopsis*. Class V contained three members: one soybean, one rice, and one *Arabidopsis*. Class VI contained three rice and one soybean member. Class VIII only contained 9 soybean members. With the exceptions of classes IV and VIII, all of the other sub-families contained rice, *Arabidopsis*, and soybean *HSP70* genes. These results suggest that the main characteristics of this family of plants were generated before the dicot/monocot split.

The combined phylogenetic tree also revealed that the paralogous and orthologous relationships among the *HSP70* family members, in particular the duplicated genes, are contained in the paralogous pairs of each species, which supports the occurrence of species-specific *HSP70* gene duplication events. In contrast, 24 pairs of paralogous genes from soybean, eight pairs from rice, and five pairs from *Arabidopsis* were identified (Additional Files 10, 11). Two pairs of orthologous genes from soybean and *Arabidopsis* were identified, where one was the pair of *Glyma01g44910* and *At2g32120* in sub-family V, and the other pair was *Glyma07g02450* and *At1g16030*. These results suggest that the ortholog pair originated from the common ancestral genes that existed before the divergence of the monocots and dicots. The fact that the genes in the paralogs accounted for most of the family members confirmed the fact that the soybean had undergone two duplication events after the monocot/dicot split, and most of the *HSP70* genes in the soybean had expanded in a species-specific manner.

### Expression patterns of soybean *GmHSP70* genes in various tissues

**Figure 5:**
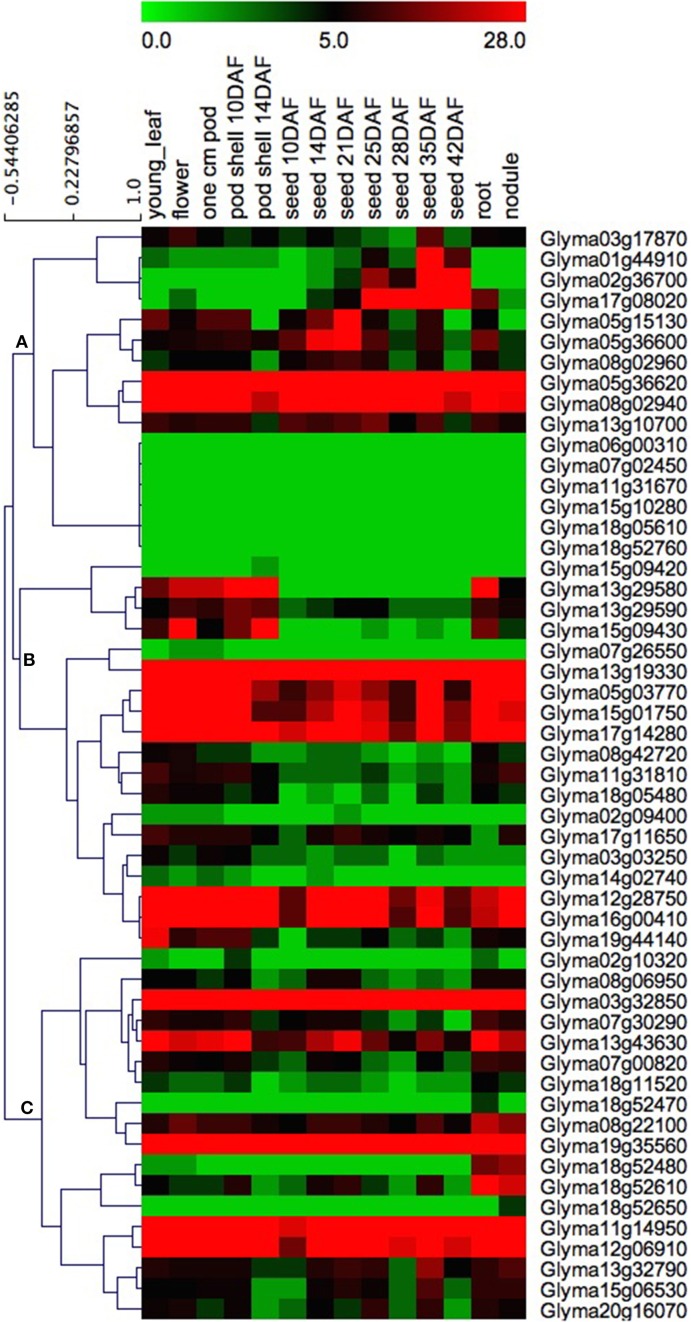
**Heat map of the expression profiles of ***GmHSP70*** candidate genes in 14 tissues**. RNA-seq relative expression data from 14 tissues were used to reconstruct the expression patterns of soybean genes. Genes were clustered into three groups **(A–C)**. The raw data was normalized and retrieved from the online database http://soybase.org/soyseq/. The normal relative expression levels of 53 *GmHSP70* genes are shown in Additional File 12.

In order to obtain more insight into the temporal and spatial expression patterns of the soybean *HSP70* genes during soybean development, the RNA-Seq Atlas of the Glycine max was searched, and the RNA-Seq atlas data of the soybean *HSP70* genes (Additional File 12) were downloaded from the Soybase (http://soybase.org/soyseq/). Due to the fact that the expression profiles of eight *GmHSP70* genes (*Glyma02g10195, Glyma02g10261, Glyma07g32921, Glyma11g31673, Glyma13g19331, Glyma13g29591, Glyma18g13077*, and *Glyma18g52471*) were not obtained in the soybean database, only the expression patterns of 53/61 *GmHSP70* genes were examined. The data analysis revealed that most soybean *HSP70* genes exhibit broad expression patterns (Figure 5). With the exception of the six *GmHSP70* genes (*Glyma06g00310, Glyma07g02450, Glyma11g31670, Glyma15g10280, Glyma18g05610*, and *Glyma18g52760*), which were not expressed in any tissues, the other 47 *GmHSP70* genes were expressed in at least one of the seven tissues (young leaves, flowers, 1cm pod, pod-shell, roots, nodules, and seed). This particularly applied to five genes, *Glyma05g36620, Glyma13g19330, Glyma03g32850*, and *Glyma19g35560*, which were highly expressed in all types of tissues. These findings indicate that the *GmHSP70* genes were involved in multiple processes during the development of the soybean. The heat map also revealed that the majority of the *GmHSP70s* showed preferential expressions. Based on a hierarchical clustering analysis, the 53 *HSP70* genes were mainly clustered into three groups (A, B, and C), as shown in Figure 5. In Group A, with the exception of the six un-expressed genes (*Glyma06g00310, Glyma07g02450, Glyma11g31670, Glyma15g10280, Glyma18g05610*, and *Glyma18g52760*), the genes showed partial expressions in the seed (from 10 to 42 DAF). The other two groups (B and C) were the opposite, with most of the genes showing down-expression in the seeds compared with the other tissues. Four *GmHSP70* genes (*Glyma15g09420, Glyma18g52470, Glyma18g52650*, and *Glyma11g14960*) showed markedly high transcript abundance profiles in only a single tissue type.

### qRT-PCR analysis of soybean *HSP70* gene expression under heat and drought stress

**Figure 6:**
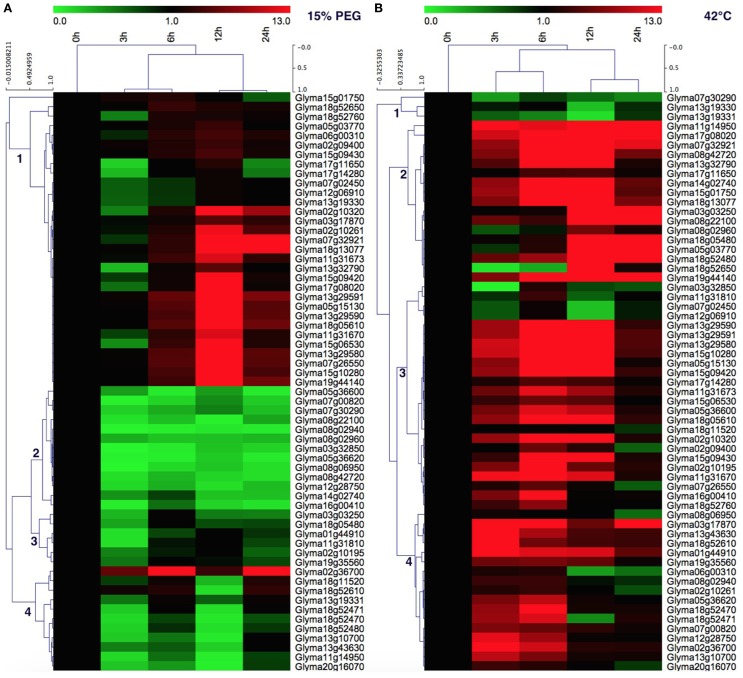
**Quantitative RT-PCR analysis of the ***GmHSP70*** gene expression in soybean leaves in response to drought stress and 42°C heat stress**. **(A)** Expression levels of 61 *GmHSP70* family genes under drought stress. **(B)** Expression levels of 61 *GmHSP70* family genes under heat stress. The normalized relative expression level of the 0 h treatment time point was set up as 1. Leaves collected at 0, 3, 6, 12, and 24 h post-drought or at 0, 3, 6, 12, and 24 h post heat stress. Means are calculated from technical triplicate qRT-PCR measurements within three biological replicates. The qRT-PCR data was shown in the Additional File 14.

In order to gain more insight into the roles of soybean *HSP70* genes in heat and drought tolerance, the expression profiles of 61 soybean *HSP70* genes in response to drought (0, 3, 6, 12, 24 h) and heat (0, 3, 6, 12, 24 h) stresses were reanalyzed by qRT-PCR. These genes expressed diversely under both stresses (Figures 6A,B). For the drought treatment (15% PEG), these genes could be divided into four clusters. Cluster 1 contains 31 (50.8%) members of detectable *GmHSP70* genes, which were widely upregulated after 6 h by drought treatment, and up to the highest expression level at 12 h, then downregulated at 24 h after drought treatment. Cluster 2 mainly consists of 13 (21.3%) genes, which were widely downregulated by drought treatment. Cluster 3 has 6 (9.8%) members, which showed a weakly upregulated at 6 or 12 h, but downregulated at 24 h after drought treatment. Cluster 4 contains 11 (18%) genes, which were mainly upregulated with the increased level of drought treatment after 24 h, and the *Glyma02g36700* in particular was highly induced at 3 h after drought treatment. Overall, 30 *GmHSP70* genes were up-regulated from two-fold to 222-fold after drought treatment relative to the control, and the other *GmHSP70* genes were down-regulated (<0.5-fold) at some stage or showed no changes during the drought stress treatment (Additional File 14).

For the heat stress treatment, the heatmap also showed the genes clustered in four groups. Cluster 1 has 3 (4.91%) members of 61 detectable *GmHSP70* genes, which were widely downregulated after 42°C treatment. The 17 members of cluster 2 were mainly upregulated after the heat treatment, and exhibited the highest expression after 12 h. Most members (24) of cluster 3 were mainly upregulated after 3 h under the 42°C treatment, but were downregulated after 24 h. And the cluster 4 members (17) had a similar expression with cluster 3, but the difference was most of genes downregulated after 12 h. Overall, 55 of *GmHSP70* genes were upregulated from two-fold to 241-fold after 42°C treatment; only 6 *GmHSP70* genes were downregulated or nearly unchanged under the heat treatment.

Notably, 29 *GmHSP70* genes showed upregulation under both drought and heat stress conditions. Five soybean *HSP70* genes were downregulated both under the drought and heat stress. 26 upregulated genes typically induced by heat were downregulated during drought stress. In contrast, only one gene, *Glyma18g11520*, was downregulated by the heat treatment and upregulated by the drought stress (Figure 6 and Additional File 14).

## Discussion

Preliminary analysis of the *HSP70* gene family has been performed in the model plants *Arabidopsis* and rice (Lin et al., 2001; Jung et al., 2013; Sarkar et al., 2013). However, this family has not previously been studied in soybean. Therefore, we performed an overall analysis of the *HSP70* gene family in soybean, including analysis of their phylogeny, chromosomal location, gene structure, conserved motifs and expression profiles. The *Arabidopsis* genome is 125 Mbp in size, with 26,500 predicted coding genes, while the soybean is 1150 Mbp, with 46,400 predicted coding genes. Therefore, the soybean possesses a 9.2-fold larger genome size and a 1.75-fold higher gene count than the *Arabidopsis* (Sterck et al., 2007; Cannon and Shoemaker, 2012). The *HSP70* gene family in soybean is by far the largest, compared with that in other plant species (18 in *Arabidopsis*, 32 in rice, and 30 in cotton) (Lin et al., 2001; Jung et al., 2013; Sarkar et al., 2013; Zhang et al., 2014).

HSP70 is a multi-genic family. Various members constituting this family were present in different cellular compartments (Cho and Choi, 2009; Kose et al., 2012). In our study, with the analysis of subcellular localization of GmHSP70 proteins, we found that multiple HSP70 members were identified in various cellular compartments in soybean. For instance, we found 34 putative nucleo/cytosolic members in soybean, compared with the OsHSP70 proteins, which contained 11 putative nucleo/cytosolic members and five cytosolic HSP70 in *Arabidopsis* (Lin et al., 2001; Jung et al., 2013; Sarkar et al., 2013). The GmHSP70 proteins encoded for 11 chloroplast, four mitochondria, three plasma membrane, and two endoplasmic reticulum.

Analyses of *cis*-elements in the promoters suggest that *GmHSP70s* might respond to regulate plant growth and development, as well as different environmental stresses and stimuli (Additional File 6). In a previous study, the promoters for 11 Arabidopsis *HSP70* genes were examined for the presence of two major temperature responsive *cis*-elements, heat shock element (HSE), and C-repeat or dehydration responsive element (CRT/DRE) (Sung et al., 2001). HSE has been linked with the heat inducible expression of many heat shock genes (Czarnecka et al., 1989). CRT/DRE is known to be associated with drought- and cold-inducible expression of many genes (Yamaguchi-Shinozaki and Shinozaki, 1994). In our *cis*-acting elements analysis of *GmHSP70s*, we found that 45 of 61 *GmHSP70* genes contain the HSE element, and one gene (*Glyma03g17870*) contains the DRE element. These results suggest that most of the *GmHSP70s* might be significantly related to heat-stress response.

Based on the phylogenetic, gene structure, and motif analysis of the HSP70 proteins in soybean, we discovered that the most closely related members in the same sub-families share similar exon/intron structures and intron numbers, and these results were also consistent with the characteristics defined in the above phylogenetic analysis (Figure 1B). For example, in sub-family I, most members contain 1 to 5 exons, while those in sub-family II contain 8 exons. In the terminal branch of the phylogenetic tree, the number of exons/introns were very similar in some of the sister pairs (Figure 1 and Additional File 10). However, there were still some sister pairs that showed changes in their intron/exon structure and numbers, for instance, *Glyma13g19330* and *Glyma13g19331*. These findings indicated that some intron loss, along with intron gain events, might have occurred during the structural evolution between the two families of soybean *HSP70* encoding genes. The same situation was also revealed by motif analysis; the type, order, and number of motifs were similar in proteins with the same sub-family, but differed from the proteins in other sub-families.

We also investigated gene duplication events in order to further understand the expansion mechanism of the soybean *HSP70* gene family. The duplications of individual genes, chromosomal segments, or entire genomes, have been major forces in the evolution of plant genome structure and content during the process of genome evolution (Sémon and Wolfe, 2007). The soybean genome is speculated to undergo at least two rounds of genome-wide duplication, followed by multiple segmental duplication, tandem duplication, and transposition events. A tandem duplication event is confirmed by the presence of two or more genes on the same chromosome, while a segmental duplication event is defined as gene duplication on different chromosomes (Schlueter et al., 2007).

Our heat map data showed that most *GmHSP70* genes were expressed in different tissues and organs of soybean. These results indicate that they may participate in growth and development. The expression patterns of the paralogous pairs in the different sub-families were revealed by heat maps, and it was determined that most of the paralogous pairs with high sequence similarity had similar expression patterns in different tissues. For instance, the *Glyma03g32850* and *Glyma19g35560* pair showed a strong expression in all of the tissues types. However, the *Glyma13g29580* and *Glyma15g09430* had a low expression in all the seed stages. An expression divergence was also found in the paralogous pairs. For example, in the *Glyma11g31810* and *Glyma18g05480* pair, *Glyma18g05480* was only highly expressed in the roots and nodules, with little or no expression in the other tissue types. Meanwhile, its paralog, *Glyma11g31810*, was not only expressed in the roots and nodules, but also had an obvious expression in the young leaves, flowers, and pods. The paralogous pair of *Glyma02g10320* and *Glyma18g52610* showed under-expression in all of the tissue types, with the exception of *Glyma18g52610* exhibiting an obvious expression in the roots and nodules. When the *Glyma07g00820* and *Glyma08g22100* pair was compared with *Glyma07g00820*, it was found that *Glyma08g22100* also displayed an obvious expression in the pods and seeds. Similar cases have also been observed in the *Glyma13g10700* and *Glyma20g16070* pair (Figure 5 and Additional File 10).

Previous studies have shown that *HSP70* genes play an important role in plants in response to various stresses, such as drought, salt, freezing temperatures, and heat (Kurepa et al., 2009). In this study, the expression profiles of the *GmHSP70* genes under drought and heat stresses (Figure 6 and Additional File 14) revealed that the soybean *HSP70* genes were widely involved in responding to drought and heat stresses. It is interesting to note that most of *GmHSP70* genes (55) were strongly upregulated during heat stress, which indicates that those genes might be mainly involved in the heat stress biological pathway. Some *GmHSP70* genes showed the same expression profile under the heat or drought treatment, being either upregulated or downregulated. This result suggests that these *GmHSP70* genes were co-expressed in responses to drought and heat stresses, and those genes might play shared roles in drought and heat stresses. However, the gene expressions profiles which responded to the different stress conditions usually tended to be different; some heat-regulated genes were downregulated in drought stress, which indicated that two sets of *GmHSP70* genes were involved in the response to the drought and heat stress, respectively. These results also imply that the signaling pathways in the plants' abiotic stress responses are very complicated systems. From an applied perspective, the identification of the *HSP70s* with potential value in the improvement of stress resistance of the soybean would likely benefit from targeting such genes which are part of the abiotic and biotic stress response networks.

## Conclusions

The soybean (*Glycine max* L.) genome contains 61 members of the *HSP70* gene family, and these genes are located within 17 chromosomes. The genome distribution, gene organizations, and gene structures suggest a complex evolutionary history of this family in soybeans. The soybean *HSP70* family is greatly contributed to by segment/chromosomal duplications, which may be associated with the soybean genome fusion events. Their expression profiles in various tissues and responses to drought and heat stress conditions demonstrate that this gene family is widely involved in soybean organ development, as well as drought and heat stress responses. The overall picture of this gene family and their potential involvement in growth, development, and stress responses, will facilitate further research on the *HSP70* gene family, particularly in regards to their evolutionary history and biological functions.

## Author contributions

LZ, HZ, and YD planned and designed the study. LZ performed the computational analysis, executed the experiments, generated the figures, and drafted the manuscript. YZ and QD contributed to the execution of the abiotic and biotic experiments. HL and GX also contributed to the sample preparations. YW provided the nematode material, along with contributing by leading the biotic experiment. LZ and YZ performed the qRT-PCR data analysis. QL and YD contributed to the discussion portion of the manuscript. All of the authors read and approved the final manuscript.

### Conflict of interest statement

The authors declare that the research was conducted in the absence of any commercial or financial relationships that could be construed as a potential conflict of interest.
