# Supplementary material for: Genome-wide analysis and expression profiling under heat and drought treatments of HSP70 gene family in soybean (Glycine max L.)
Source: Front Plant Sci. 2015 Sep 25;6:773. doi: 10.3389/fpls.2015.00773 (PMC4585176; doi:10.3389/fpls.2015.00773)
Supplement: Supplementary file 1 [file DataSheet1.DOCX]

>Glyma01g44910 MVEPAYTVTSDSETTGEEKSSTFPEIAIGIDIGTSQCSVAVWNGSQVELLKNTRNQKIMKSYVTFKDNIPSGGVSSQLSHEDEMLSGATIFNMKRLIGRVDTDPVVHACKNLPFLVQTLDIGVRPFIAALVNNMWRSTTPEEVLAIFLVELRAMAEAQLKRRIRNVVLTVPVSFSRFQLTRIERACAMAGLHVLRLMPEPTAVALLYGQQQQQTSHENMGSGTEKIALIFSMGAGYCDVAVTATAGGVSQIKALAGSTIGGEDLLQNMMHHLLPNSENLFKNHGVKEIKQMGLLRVATQDAIRQLSSQTIVQVDVDLGDGLKICKAVNREEFEEVNRKVFEKCESLIIQCLQDAKVEVEEVNDVIIVGGCSYIPRVKNLVTNVCKGKELYKGMNPLEAAVCGAAVEGAIASGVNDPFGNLDLLTIQATPLAIGIRADGNKFVPVIPRDTTMPARKELVFTTTHDNQTEALILVYEGEGEKAEENHLLGYFKIMGIPAAPKGVPEINVCMDIDAANVLRVLAGVVMPGSRQPAIPVMEVRMPTVDDGHGWCAEALNRTYGATLDLVTLQKKA

>Glyma02g09400 MAKKYEGCAVGIDLGTTYSCVAVWLEQHCRVEIIHNDQGNNTTPSCVAFTDQQRLIGEAAKNQAATNPENTVFDAKRLIGRKFSDPVIQKDKMLWPFKVVAGINDKPMISLNYKGQEKHLLAEEVSSMVLIKMREIAEAYLETPVENAVVTVPAYFNDSQRKATIDAGAIAGLNVMRIINEPTAAAIAYGLDKRTDCVEERNIFIFDLGGGTFDVSLLTIKDKVFQVKATAGNTHLGGEDFDNRMVNYFVQEFKRKNKVDISGNPRALRRLRSACERAKRILSYAVTTNIEVDALFQGVDFCSSITRAKFEEINMELFEECMETVDRCLSDANMDKSSVHDVVLVGGSSRIPKVQELLQGFFDGKVLCKSINPDEAVAYGAAVQAALLSKGIVNVPNLVLLDITPLSLGVSVQGDLMSVVIPRNTTIPVRRTKTYVTTEDNQSAVMIEVYEGERTRASDNNLLGFFTLSGIPPAPRGHPLYETFDIDENGILSVSAEEESTGNKNEITITNEKERLSTKEIKRMIQEAEYYKAEDKKFLRKAKAMNDLDYYVYKIKNALKKKDISSKLCSKEKENVSSAIARATDLLEDNNQQDDIVVFEDNLKELESIIERMKAMGKIEFVYIHTHLSLISSQSKVTRDIEMSTKKGCPSHAEAMQLKQPGAPFKCSGCKQMGFGPSYHCESSNCSYVLHEECANAVSIAFHPFFSKSNFEFHEKAPGKRTRYCDGCGKDVLGFVYHCSTTGYDLHPCCLKLKHNISDQEGRVTLELCQKVPSKCVKCKHRNVVERVKGWSYVSSGGDCCYHVSCVKELILENWKKGYFSQETNNSIGMSSDRENTQVALRSMEIVPSGRRSRRINKYTKIAVLVFKLVVSAIFGNPISAIAALVEALVTD*

>Glyma02g10195 MRFMSYFNGKGLCMSINPNEAVAYGIKNVPDLVLLDVMSLSLENLTSVQINVYEGERTRASDNNLLGFFSLSGFPPTPQYHPFDICFDIDVNGILSVSAEEKTTGYKNDIAITNDEGKLSAEEIKRMIEKAETYQAEDNKFLRKANAMNALDDYIYKMKTILKKDDISLKLCSQERQKISFAVTKATNLLHDDKQQNEAVVFEDSLKELAI*

>Glyma02g10261 MIGGCYFADAKRLIGRRVSDPSVHSDMKLWPFKVIAGAGEKPMIGVNYKGKEKQFSTEEISSMVLTKMRKIAEAYLGSTVKNAFVTVPAYFNDSQRQASKDVGVITGLNVMRIINEPTVVAIALGLDKKATSVGEKNVLIFDLGGGTFDTTIEIDSLFEGIDFYSTITRARFEELNMNLFRKCMEPVEKCLREAKMSKITVHDVVLVGGSTRIPKVQQLLQDFFNGKDLCKNINPNEVAAYGVAVQATILSGEGNEKVQDLLLLDFTPLSLGLETAGDVMT

>Glyma02g10320 HFCHVEIIANDQGNRTTPSYVGFTDSERLIGDAAKNQVAMNPVNTVFDAKRLIGRRISDASVQSDMKLWPFKVIPGPADKPMIVVNYKGEDKQFAAEEISSMVLMKMREIAEAYLGSTVKNAVVTVPAYFNDSQRQATKDAGVIAGLNVMRIINEPTAAAIAYGLDKKATSVGEKNVLIFDLGGGTFDVSLLTIEEGIFEVKATAGDTHLGGEDFDNRMVNHFVQEFKRKHKKDISGNPRALRRLRTACERAKRTLSSTAQTTIEIDSLYEGVDFYTTITRARFEELNMDLFRKCMEPVEKCLRDAKMDKSTVHDVVLVGGSTRIPKVQQLLQDFFNGKELCKSINPDEAVAYGAAVQAAILSGEGNEKVQDLLLLDVTPLSLGLETAGGVMTVLIPRNTTIPTKKEQVFSTYSDNQPGVLIQVYEGERARTRDNNLLGKFELSGIPPAPRGVPQITVCFDIDANGILNVSAEDKTTGQKNKITITNDKGRLSKEEIEKMVQEAEKYKAEDEEHKKKVDAKNALENYAYNMRNTIKDEKIASKLSGDDKKKIEDAIESAIQWLDGNQLAEADEFEDKMKELESTCNPIIAKMYQGAGAPDMAGGMDEDVPPSGSGGAGPKIEEVD*

>Glyma02g36700 MATKEGKAIGIDLGTTYSCVGVWQNDRVEIIPNDQGNRTTPSYVAFTDTERLIGDAAKNQVAMNPQNTVFDAKRLIGRRFSDSPVQNDMKLWPFKVVAGPGDKPMIVVNYKGEEKKFSAEEISSMVLVKMREVAEAFLGHAVKNAVITVPAYFNDSQRQATKDAGAISGLNVLRIINEPTAAAIAYGLDKKASRKGEQNVLIFDLGGGTFDVSILTIEEGIFEVKATAGDTHLGGEDFDNRMVNHFVSEFRRKNKKDISGNARALRRLRTACERAKRTLSSTAQTTIEIDSLYEGIDFYATITRARFEEMNMDLFRKCMEPVEKCLRDAKIDKSHVHEVVLVGGSTRIPKVQQLLQDFFNGKELCKSINPDEAVAYGASVQAAILSGEGDEKVQDLLLLDVTPLSLGLETAGGVMTVLIPRNTTIPTKKEQIFSTYSDNQPGVLIQVFEGERARTKDNNLLGKFELTGIPPAPRGVPQINVCFDIDANGILNVSAEDKTAGVKNKITITNDKGRLSKEEIEKMLKDAERYKAEDEEVKKKVEAKNSLENYAYNMRNTIKDEKIGEKLSPDEKEKIEKAVEDAIQWLEGNQLAEVDEFEDKQKELEGICNPIIAKMYQGAAARPGGDVPTGDDDMPGAGGAGSGAGPKIEEVD*

>Glyma03g03250 MTHNHKHPYKRMNKYISSVIVFGKKIVPFRRKTLSALSSASSSLTMVLSLSTLGFSSALSRLNFFPKPPPSLFSALTTFRRNQLPLKSSLAASKRDGDVVVLGIETSCDDTAAAVVRSDGEILSQVVSSQADLLAKYGGVAPKMAEEAHSKVIDQVVQEALDKAYLTEKDLTAVAVTIGPGLSLCLRVGVQKARKIAGGFNLPIIGIHHMEAHALVARLIEKDLQFPFMALLISGGHNLLVLARDLGQYIQLGTTIDDAIGEAYDKTAKWLGLDLRRSGGPAIEKLAMEGNAESVKFSIPMKQHKDCNFSYAGLKTQVRLAIESKKIDAKIPISSASNGDRLSRADIAASFQRIAVLHLEERCERAIQWALKMEPSIRHLVVSGGVASNQYVRARLDMVVKKNGLQLVCPPPRLCTDNGVMIAWTGIEHFRMGRYDPPPPAEEPEDFVYDIRPRWPLGEEYAEGKSVARSLRTARIHPSLTSIIQASLQQ*

>Glyma03g17870 MAKEGPNWDGLLKWSIAHSDGTSPTRNLSEEDRKWFMEAMQAQTIDVVKRMKEITLVMQTPEQVLKDQGVTPADIEDMLEELQEHVESIDMANDLHSIGGLVPLLGYLKNSHANIRAMAADVVTTIVQNNPRSQQLVMEANGFEPLISNFSSDPDVTVRTKALGAISSLIRHNKPGITAFRLANGYAALKDALASENVRFQRKALNLIHYLLHENNSDCNIVNELGFPRMLMHLASSEDSDVREAALRGLLQLAHNAKDGKDGNEKDSVKIKQLLQERINNISLMSAEDLGVVREERQLVDSLWSTCFNEPSSLREKGLLVLPGEDVPPPDVASKYFEPPLRSSTANPSSKKDPEKNEIPLLLGSGPSPTYTNNQGSNKGDASS*

>Glyma03g32850 MAGKGEGPAIGIDLGTTYSCVGVWQHDRVEIIANDQGNRTTPSYVGFTDTERLIGDAAKNQVAMNPINTVFDAKRLIGRRFSDSSVQSDIKLWPFKVIPGAADKPMIVVNYKGEEKQFAAEEISSMVLIKMREIAEAYLGSTVKNAVVTVPAYFNDSQRQATKDAGVIAGLNVMRIINEPTAAAIAYGLDKKATSVGEKNVLIFDLGGGTFDVSLLTIEEGIFEVKATAGDTHLGGEDFDNRMVNHFVQEFKRKNKKDISGNPRALRRLRTACERAKRTLSSTAQTTIEIDSLYEGIDFYSTVTRARFEELNMDLFRKCMEPVEKCLRDAKMDKRSVDDVVLVGGSTRIPKVQQLLQDFFNGKELCKSINPDEAVAYGAAVQAAILSGEGNEKVQDLLLLDVTPLSLGLETAGGVMTVLIPRNTTIPTKKEQVFSTYSDNQPGVLIQVFEGERARTRDNNLLGKFELSGIPPAPRGVPQITVCFDIDANGILNVSAEDKTTGQKNKITITNDKGRLSKEDIEKMVQEAEKYKSEDEEHKKKVEAKNALENYAYNMRNTVKDDKIGEKLDPADKKKIEDAIEQAIQWLDSNQLAEADEFEDKMKELESICNPIIAKMYQGGAGPDVGGAGAAEDEYAAPPSGGSGAGPKIEEVD

>Glyma05g03770 MDASKLNQLKHFIEQCKSNPSLLSDPSLSFFRDYLESLGAKLPESAYSESTGVESDEDIEDVTEEQEKVEEEEEDDEIIESDVELEGETCQSDDDPPQKMGDPSVEVTEENRDASQMAKIKAMDAISEGKLEEAIENLTEAILLNPTSAIMYGTRASVYIKMKKPNAAIRDANAALEINPDSAKGYKSRGVARAMLGQWEEAAKDLHVASKLDYDEEINAVLKKVEPNAHKIEEHRRKYERLHKEREDKKKERERQRRRAEAQAAYEKAKKQEQSSSSRNPGGMPGGFPGGMPGGFPGAGGMPGGFPGAGGMPGGFPGAGGMPGGGFPGAGGMPGGVPGNIDFSKILSDPELMAAFSDPEVMAALQDVMKNPANFAKHQSNPKVGPVIAKMMTKLGGGPK

>Glyma05g15130 YKKGMEDVGLHKNQMDEIDLVGGSTRIPKVRHLLKDYFEGKKPNKVQRSILSEEGGEETKGTLVCNLAFFITYYYVVRFLIVVCSGSRYPSPGCGSPPFLLSFRIFFCFVVSGFHCKGWPLEFATPATMSASTVDCATT

>Glyma05g36600 MLVLSSEHYKWRSPRRKLNKTAIRPSRKERKGDMIMARSFSRGSLLPLAIVSLVCLFVISIAKEEATKLGTVIGIDLGTTYSCVGVYKNGHVEIIANDQGNRITPSWVAFTDSERLIGEAAKNLAAVNPERTIFDVKRLIGRKFEDKEVQRDMKLVPYKIVNKDGKPYIQVKIKDGETKVFSPEEISAMILTKMKETAEAFLGKKINDAVVTVPAYFNDAQRQATKDAGVIAGLNVARIINEPTAAAIAYGLDKKGGEKNILVFDLGGGTFDVSILTIDNGVFEVLATNGDTHLGGEDFDQRIMEYFIKLIKKKHGKDISKDSRALGKLRREAERAKRALSSQHQVRVEIESLFDGVDFSEPLTRARFEELNNDLFRKTMGPVKKAMEDAGLQKSQIDEIVLVGGSTRIPKVQQLLKDYFDGKEPNKGVNPDEAVAYGAAVQGSILSGEGGEETKDILLLDVAPLTLGIETVGGVMTKLIPRNTVIPTKKSQVFTTYQDQQTTVSIQVFEGERSLTKDCRLLGKFELSGIPPAPRGTPQIEVTFEVDANGILNVKAEDKGTGKSEKITITNEKGRLSQEEIERMVREAEEFAEEDKKVKERIDARNSLETYVYNMKNQIGDKDKLADKLESDEKEKIETAVKEALEWLDDNQSVEKEEYEEKLKEVEAVCNPIISAVYQRSGGAPGGGASGEEDDDSHDEL*

>Glyma05g36620 MAGSWARRSLIVLAIISFGCLFAISIAKEEATKLGTVIGIDLGTTYSCVGVYKNGHVEIIANDQGNRITPSWVAFTDSERLIGEAAKNLAAVNPERTIFDVKRLIGRKFEDKEVQRDMKLVPYKIVNKDGKPYIQVKIKDGETKVFSPEEISAMILTKMKETAEAFLGKKINDAVVTVPAYFNDAQRQATKDAGVIAGLNVARIINEPTAAAIAYGLDKKGGEKNILVFDLGGGTFDVSILTIDNGVFEVLATNGDTHLGGEDFDQRIMEYFIKLIKKKHGKDISKDNRALGKLRREAERAKRALSSQHQVRVEIESLFDGVDFSEPLTRARFEELNNDLFRKTMGPVKKAMEDAGLQKSQIDEIVLVGGSTRIPKVQQLLKDYFDGKEPNKGVNPDEAVAYGAAVQGSILSGEGGEETKDILLLDVAPLTLGIETVGGVMTKLIPRNTVIPTKKSQVFTTYQDQQTTVSIQVFEGERSLTKDCRLLGKFDLSGIPPAPRGTPQIEVTFEVDANGILNVKAEDKGTGKSEKITITNEKGRLSQEEIERMVREAEEFAEEDKKVKERIDARNSLETYVYNMKNQISDKDKLADKLESDEKEKIETAVKEALEWLDDNQSMEKEDYEEKLKEVEAVCNPIISAVYQRSGGAPGGGGASGEEDEDDSHDEL*

>Glyma06g00310 EDSRGGVSFVADAVYSPEELVAMMLGHTASLAEFHAKVPIKDAVIAVPPNLGQAERRGLLVAVQFAGINSRHVIFYDMGSSSTYAAVVYFSSCGKVNPELGGQHMELRLVEYFADEFNAQVGGGIDVRHFPKAMATLKKQVKRRKEMLSANTVAPISVESLDDGVDFGSTMNREKFEDLCQDIWDKSLLPVKEVLQHSGLSLDLIYALQLIGGATRVPKLQAQLQQFLGRKQLDRHLDADEAIVLGSAPHAANLSDGIKLKSKLGILDASMYGFVVELSAPDLSKDESSRQLLVPQMKKVPSKDPEHHLPPGVTSPEIAQYQISGLTDASEKYSSRNLRSGILSLDRADAIIEITERVEVPRKNMTIENSTISSNVSAESAGSNSSEENMQTDSEISKTSNGSAEEQATAAEPATEEKLKKRTFRVPLNIVEKITGPGMPLSQDFLAEAKRKLLALDEKDADRKRTTDEERQSFIEKLDQVQDWLYRDGEDANATEFQELLDQLKTVGNPIFFRLKELTARPAAVEHAHRYIDELKEWKANKKPAFISEAVYSKVLDLQNKVSSINRIPKQNT*

>Glyma07g00820 MSVVGFDFGNESCVVAVARQRGIDVVLNDESKRETPAIVCFGDKQRFIGTAGAASTMMNPKNSISQIKRLIGRKFADPELQRDLKSLPFLVTEGSDGYPLIHARYMGEAKTFTPTQVFGMMLSNLKEIAEKNLTTAVVDCCIGIPVYFTDLQRRAVLDAATIAGLHPLRLIHEMTATALAYGIYKTDLPENDQLNVAFVDVGHASLQVCIAGFKKGQLKVLAHSYDRSFGGRDFDEVLFHHFAEKFKDEYKIDVFQNARACIRLRAACEKIKKMLSANPEAPLNIECLMDEKDVRGFIKRDEFEQLSLPILERVKGPLEKALAEAGLTVENVHTVEVVGSGSRVPAINKILTEFFKKEPRRTMNASECVARGCALECAILSPTFKVREFQVNESLPFSISLSWKSSGPDAQDNGPENQQSSLVFPKGNPIPSIKALTFYRSGTFSVDVQFGDVSGLQTPAKISTYTIGPFQTTNGEKAKVKVKVRLNLHGIVSLESATLLEEEEVDVPVSKEAAGENTKMDIDEVPAEAAAPPSSNDTGANMENGKASIDASGVEDGIPESGGKPLQTDTDTKVQAPKKKVKKTNIPVVELIYGAMVPVDVQKALEKEFEMALQDRVMEETKDKKNAVEAYVYDMRNKLNDKYQEFVTASERDDFTAKLQEVEDWLYGEGEDETKGVYTAKLEELKKHGDPIDERYKEFMERGTIIEQFVYCINSYRQVAMSNDPRFEHIDINEKQKVINECVEAEKWFNEKQQQQNSLPKYANPVLLSAEIRKKAEAVDRFCKPIMATPRPTKATTPPGPATHPSSQSDEQQQQQQPPQGDADANSNENGGNSSSQAAPASTEPMETDKSEKTASA*

>Glyma07g02450 PTAAAIAYGLDKKASRSGEKNVVIFDLGGGTFDVSLLTIQEAIFQVKATAGDTHLGVESNYILSSSNVSFAYQLVNHFVSEFKRKHKKDVSTNARALRRLRTACERGLRGLSLPPLKLPSRLTLSTKVLTSIPPSPEPGLRSSTWTRSRCCPCWWIHQDSKSATTSINPDEAVAYGAAVQAAILSGEGNEKVQDLLLLDVTPLSLGIETAGGVMTVLIPRNTTIPTKKEQIFSTYADNQPGVLIQVYEGERASTKDNNLLGKFELTGIPSAPRGVPQINVCFDIDANDGPGGGEVDAKNSLENLAYNMRNTVKDDKFAGKMNPSDKEKIEKAVDETIEWLDRNLLTEVEEFQDKLKELEGLCNPIISNMYQGSGADDIPNGAGYGKSSTGGAGPKIEE

>Glyma07g26550 MICDFCFLKENQIMAREYEGCAVGIDLGTTYSCVAVWLEQHCRVEIIHNDQGNNTTPSCVAFTDHQRLIGEAAKNQAATNPENTVFDAKRLIGRKFSDPVIQKDKMLWPFKIVAGINDKPMISLNYKGQEKHLLAEEVSSMVLTKMREIAEAYLETPVKNAVVTVPAYFNDSQRKATIDAGSIAGLNVMRIINEPTAAAIAYGLDKRTNCVGERSIFIFDLGGGTFDVSLLIIKDKVFRVKATAGNTHLGGEDFDNRMVNYFVQEFKRKNKVDISGNARALRRLRSACERAKRILSYAVTTNIEVDALFQGIDFCSSITRAKFEEINMELFEECMETVDRCLSDANMDKSSVHDVVLVGGSSRIPKVQELLQDFFNGKILCKSINPDEAVAYGAAVQAALLSKGIVNVPDLVLLDITPLSLGISLKGDLMSVVIPRNTTIPVKTTETYSTAVDNQSAVLIEVYEGERTRASDNNLLGFFRLSGIPPVPRNHLVYICFAIDENGILSVSAEEKSTGNKNEITITNDKERLSTKEIKRMIQEAEYYQAEDKKFLRKAKAMNDLDCYVYKIKNALKQKDISSKLCSKEKEDVSSAITRATDLLEGNNQQDDIAVFEDNLKELESIIERMKAMGKIV*

>Glyma07g30290 MAAATALLRSLRRRDLPSSSLSAFRSLTSGTKTSYVGNKWASLSRPFSSKPAGNDVIGIDLGTTNSCVSVMEGKNPKVIENSEGARTTPSVVAFNQKAELLVGTPAKRQAVTNPTNTLFGTKRLIGRRFDDSQTQKEMKMVPYKIVKASNGDAWVEANGQQYSPSQVGAFVLTKMKETAESYLGKSVSKAVITVPAYFNDAQRQATKDAGRIAGLDVQRIINEPTAAALSYGMNNKEGLIAVFDLGGGTFDVSILEISNGVFEVKATNGDTFLGGEDFDNALLDFLVNEFKRTESIDLSKDKLALQRLREAAEKAKIELSSTSQTEINLPFITADASGAKHLNITLTRSKFEALVNHLIERTKAPCKSCLKDANISIKEVDEVLLVGGMTRVPKVQEVVSAIFGKSPSKGVNPDEAVAMGAAIQGGILRGDVKELLLLDVTPLSLGIETLGGIFTRLINRNTTIPTKKSQVFSTAADNQTQVGIKVLQGEREMAVDNKSLGEFELVGIPPAPRGMPQIEVTFDIDANGIVTVSAKDKSTGKEQQITIRSSGGLSEDEIDKMVKEAELHAQKDQERKALIDIRNSADTSIYSIEKSLGEYRDKIPSEVAKEIEDAVSDLRTAMAGDNADEIKAKLDAANKAVSKIGEHISGGSSGGSSAGGSQGGEQAPEAEYEEVKK*

>Glyma07g32921 MPAHNSHETTMDCLEEALSHLTQNISTMTAKSLEMAAKLDVILDWLSALQPTPSSPKSLAPPDAPMPNLPPMVGINSGTTYSCVKVWQHHHVEIIATDQRNRSSASYFAFTERLIGDAAKYYVTINPINTIFNAKRLCGRRFSAASIRSDRQLWLYKVFPGPPDKPMIVVNYKGEDKKFAAEEMSSMLPFIDVIGDQSSSKSSVLESLAAIKLPRGQGTCTRVPLDMRLRNHPFTTLELVLEFYGQTISIDEAHISQAISAATAATEELACHGKGISNNPLTLLEKKNGVPDLYPVDLPCIIQVPVHGQPKNIYDQIKDMIMEYIKPEASILLTVLSASVDFTTCESIGMSQSVEKTELRTLAVVTKTDKSPESLLKRHSGWFNCCQWPI*

>Glyma08g02940 MAGSWARRSLIVLAIISFGCLFAISIAKEEATKLGTVIGIDLGTTYSCVGVYKNGHVEIIANDQGNRITPSWVAFTDSERLIGEAAKNQAAVNPERTIFDVKRLIGRKFEDKEVQKDMKLVPYKIVNKDGKPYIQVKIKDGETKVFSPEEISAMVLIKMKETAEAFLGKKINDAVVTVPAYFNDAQRQATKDAGVIAGLNVARIINEPTAAAIAYGLDKKGGEKNILVFDLGGGTFDVSILTIDNGVFEVLATNGDTHLGGEDFDQRIMEYFIKLIKKKHGKDISKDNRALGKLRREAERAKRALSSQHQVRVEIESLFDGVDFSEPLTRARFEELNNDLFRKTMGPVKKAMEDAGLQKSQIDEIVLVGGSTRIPKVQQLLKDYFDGKEPNKGVNPDEAVAYGAAVQGSILSGEGGEETKDILLLDVAPLTLGIETVGGVMTKLIPRNTVIPTKKSQVFTTYQDQQTTVSIQVFEGERSLTKDCRLLGKFDLSGIPPAPRGTPQIEVTFEVDANGILNVKAEDKGTGKSEKITITNEKGRLSQEEIDRMVREAEEFAEEDKKVKERIDARNSLETYVYNMKNQVSDKDKLADKLESDEKEKIETAVKEALEWLDDNQSVEKEDYEEKLKEVEAVCNPIISAVYQRSGGAPGGAGGEGEDEDDSHDEL*

>Glyma08g02960 MTCSYLRVSTINGEVPAVNSIEPTIHRPSRKEEKKQGDIIMACSFSRGSLLPLAIIVSLGCLFAISIAKEEATKLGTVIGIDLGTTYSCVGVYKNGHVEIIANDQGNRITPSWVAFTDSERLIGEAAKNLAAVNPERVIFDVKRLIGRKFEDKEVQRDMKLVPYKIVNKDGKPYIQVKIKDGETKVFSPEEISAMILTKMKETAEAFLGKKINDAVVTVPAYFNDAQRQATKDAGVIAGLNVARIINEPTAAAIAYGLDKKGGEKNILVFDLGGGTFDVSILTIDNGVFEVLATNGDTHLGGEDFDQRIMEYFIKLINKKHKKDISKDSRALGKLRREAERAKRALSSQHQVRVEIESLFDGVDFSEPLTRARFEELNNDLFRKTMGPVKKAMEDAGLQKNQIDEIVLVGGSTRIPKVQQLLKDYFDGKEPNKGVNPDEAVAYGAAVQGSILSGEGGEETKDILLLDVAPLTLGIETVGGVMTKLIPRNTVIPTKKSQVFTTYQDQQSTVSIQVFEGERSLTKDCRLLGKFELSGIPPAPRGTPQIEVTFEVDANGILNVKAEDKGTGKSEKITITNEKGRLSQEEIERMVREAEEFAEEDKKVKERIDARNSLETYVYNMKNQVSDKDKLADKLESDEKEKIETAVKEALEWLDDNQSVEKEEYEEKLKEVEAVCNPIISAVYQRSGGAPGGGASGEDDDEDSHDEL*

>Glyma08g06950 MAAATALLRSLRRRDLPSSSLSAFRSLTSGTKTSYVGNKWASLSRPFSSKPAGNDVIGIDLGTTNSCVSVMEGKNPKVIENSEGARTTPSVVAFNQKAELLVGTPAKRQAVTNPTNTLFGTKRLIGRRFDDSQTQKEMKMVPYKIVKAPNGDAWVEANGQQYSPSQVGAFVLTKMKETAESYLGKSVSKAVITVPAYFNDAQRQATKDAGRIAGLDVQRIINEPTAAALSYGMNNKEGLIAVFDLGGGTFDVSILEISNGVFEVKATNGDTFLGGEDFDNALLDFLVNEFKRTENIDLSKDKLALQRLREAAEKAKIELSSTSQTEINLPFITADASGAKHLNITLTRSKFEALVNHLIERTKAPCKSCLKDANVSIKEVDEVLLVGGMTRVPKVQEVVSAIFGKSPSKGVNPDEAVAMGAAIQGGILRGDVKELLLLDVTPLSLGIETLGGIFTRLINRNTTIPTKKSQVFSTAADNQTQVGIKVLQGEREMAVDNKSLGEFELVGIPPAPRGMPQIEVTFDIDANGIVTVSAKDKSTGKEQQITIRSSGGLSEDEIDKMVKEAELHAQKDQERKALIDIRNSADTTIYSIEKSLGEYRDKIPSEVAKEIEDAVSDLRTAMAGDNADEIKAKLDAANKAVSKIGEHMSGGSSGSSSAGGSQGGEQAPEAEYEEVKK*

>Glyma08g22100 MSVVGFDFGNESCIVAVARQRGIDVVLNDESKRETPAIVCFGDKQRFIGTAGAASTMMNPKNSISQFKRLIGRKFSDPELQRDLKSLPFLVTEGSDGYPLIHARYMGESKTFTPTQVFGMMLSNLKEIAEKNLTTAVVDCCIGIPVYFTDLQRRAVLDAATIAGLHPLRLIQEMTATALAYGIYKTDLPENDQLNVAFVDVGHASMQVCIAGFKKGQLKVLAHSYDRSLGGRDFDEVLFHHFAGKFKEEYKIDVFQNARACIRLRTACEKIKKMLSANPVAPLNIECLMDEKDVRGFIKRDEFEQLSLPILERVKGPLEKALAEAGLTVENVHTVEVVGSGSRVPAINKILTEFFKKEPRRTMNASECVARGCALECAILSPTFKVREFQVNESLPFSISLSWKGSGPDAQDNGSENQQSSLVFPKGNPIPSIKALTFCRAGTFSVDVLYDDASGLQTPAKISTYTIGPFQTTNGERAKVKVKVRLNLHGIVSLESATLLEEEKVGVPVTKEAAGENTKMDIDEVPAEAAAPPASNDTGANMEGAKASTDASGVENGIPEGGDKPLQKDTDTKVQAPKKKVKKTNIPVAELVYGAMVPVDVQKALEKEFEMALQDRVMEETKDKKNAVEAYVYDMRNKLNDEYQEFVTASERDDFTAKLQEVEDWLYDEGEDETKGVYIAKLEELKKQGDPIDGRYEEFTERGTIIEQFVYCINSYRQVAMSNDPRFEHIDINEKQKVINKCVEAEKWFNEKQQQQSSLPKYANPVLLSAEMRKKAEDVDRFCKPIMTTQKPTKAVTPAGPATPSSQSDEQQQPQGDSDVNSNENAGNSSSQAAPASTEPMETEKSENTGSA*

>Glyma08g42720 MSVVGFDIGNENCVIAVVRQRGIDVLLNYESKRETPAVVCFSEKQRILGSAGAASAMMHIKSTISQIKRLIGRKFADPDVKKELKMLPGKTSEGQDGGILIHLKYSGEIHVFTPVQFLSMLFAHLKTMTENDLEMPISDCVIGIPSYFTDLQRRAYLDAAKIAGLQPLRLIHDCTATALSYGMYKTDFGSAGPAYVAFIDIGHCDTQVCIASFEFGKMEILSHAFDRSLGGRDFDEVIFSHFAAKFKEEYHIDVYSKTKACFRLRAACEKLKKVLSANLEAPLNIECLMDGKDVKGFITREEFEKLASGLLERVSIPCRRALTDANLTAEKISSVELVGSGSRIPAISTSLTSLFKREPSRQLNASECVARGCALQCAMLSPVYRVREYEVKDVIPFSIGLSSDEGPVAVRSNGVLFPRGQPFPSVKVITFQRSNLFHLEAFYANPDELPPRTSPKISCVTIGPFHGSHGSKIRVKVRVPLDLHGIVSIESATLIKDDMDDLVMAGDYHSNSDAMDIDPISETVTNGFEDDTNKKLEFPCSSADGTRKDNRRLNVPVNENVYGGMTKAEISEALEKELQLAQQDRIVEQTKEKKNSLESFVYDMRSKLFHTYRSFASEQEKDGISRSLQETEEWLYEDGVDETEHAYSSKLEDLKKLVDPIENRYKDDKERVHATRDLSKCILKHRASADSLPPQDKELIINECNKVEQWLKEKIQQQESFPKNTDPILWSSDIKSKTEELNLKCQQILGSNASPSPEDKDKPDTFNDP

>Glyma11g14950 MAGKGEGPAIGIDLGTTYSCVGVWQHDRVEIIANDQGNRTTPSYVAFTDTERLIGDAAKNQVAMNPTNTVFDAKRLIGRRFSDASVQGDMKLWPFKVIPGPAEKPMIVVNYKGEEKQFSAEEISSMVLMKMKEIAEAYLGSTIKNAVVTVPAYFNDSQRQATKDAGVISGLNVMRIINEPTAAAIAYGLDKKATSSGEKNVLIFDLGGGTFDVSLLTIEEGIFEVKATAGDTHLGGEDFDNRMVNHFVQEFKRKNKKDISGNARALRRLRTACERAKRTLSSTAQTTIEIDSLYEGIDFYTTITRARFEELNMDLFRKCMEPVEKCLRDAKMDKSTVHDVVLVGGSTRIPKVQQLLQDFFNGKELCKSINPDEAVAYGAAVQAAILSGEGNEKVQDLLLLDVTPLSTGLETAGGVMTVLIPRNTTIPTKKEQVFSTYSDNQPGVLIQVYEGERTRTRDNNLLGKFELSGIPPAPRGVPQITVCFDIDANGILNVSAEDKTTGQKNKITITNDKGRLSKEEIEKMVQEAEKYKSEDEEHKKKVEAKNALENYAYNMRNTIKDDKIASKLSSDDKKKIEDAIEQAIQWLDGNQLAEADEFEDKMKELESICNPIIAKMYQGAGGDAGGAMDEDGPAAGSGSGAGPKIEEVD*

>Glyma11g31670 INLGTTYSCVAVWREHHRRVEIIHNDQGNTRSEATNDQNSFKFADSKRLIGRKYSCCRVRRSTFVLRKKMSIINGSCEDNEVVTVPAYFNDSQYKATIDAGKIAGLNILRIINEPVAAAIMHGLDMRTNNCVGERNIFIFDLGGGTFDASLLTLKGKIFKVKATAGNGHLGGEDIDNRMLDHFVKEIKRKKKVDISGNLKVLRRLKTTCERAKRTLSHAVTTNIEVDALSDAIDFCSSITRAKFEEINMELFKECMETVDKCLTDSKMNKSSVHDVILVVVLQGFPKCKSYCRTFPTERICERASTLMKLLLMVQLMIQEAEEYQAEDKKFLRKATAMNKLNDYVNKMNNGLENENLSSKLCSEDKEKISSAITKATKLIDGDNKK*

>Glyma11g31673 MAKEGHRIAIGINLGTTYSCVAVWREHHRRVEIIHNDQGNTRSEATVDSKRLIGRKYSDPVVQKDKLLWHSRLLPMWQIAEAFLEKHAKNEVVTVPAYFNDSQYKATIDAGKIAGLNILRIINEPVAAAIMHGLDMRTNNCVGERNIFIFDLGGGTFDASLLTLKGKIFKVKATAGNGHLGGEDIDNRMLDHFVKEIKRKKKVDISGNLKVLRRLKTTCERAKRTLSHAVTTNIEVDALSDAIDFCSSITRAKFEEINMELFKECMETVDKCLTDSKMNKSSVHDVILVVSINPDEAVAYGAAVHAALLSEDCQGKKLIE*

>Glyma11g31810 MEKLNLALVSSPKPLMLGHVPARDVFRRKHFSFGRVLIAPHRCRFRVSALSSSHHNPKSVQEKLIVKHFASISSSNTQETTSIGVKPQLSPSPSSTIGSPLFWIGVGVGLSALFSVVASRLKKYAMQQAFKTMMGQMNSQNNQFGNAAFSPGSPFPFPMPTAAGPTAPASSATTQSRAPSASSASQSTITVDLPAAKVEAAPTTNVKDEVELKNEPKKIAFVDVSPEETVRESPFESFKDDESSSVKEAWVPDEVSQNGAPSNLGFGDFPGSQSTKKSALSVDALEKMMEDPTVQKMVYPYLPEEMRNPTTFKWMLQNPQYRQQLEEMLNNMGGSTEWDNRMMDTLKNFDLNSPEVKQQFDQIGLSPEEVISKIMANPEVAMAFQNPRVQAAIMDCSQNPMNITKYQNDKEVMDVFNKISELFPGVGSP*

>Glyma12g06910 MAGKGEGPAIGIDLGTTYSCVGVWQHDRVEIIANDQGNRTTPSYVAFTDTERLIGDAAKNQVAMNPINTVFDAKRLIGRRFSDASVQSDMKLWPFKVIPGPADKPMIVVNYKGDEKQFSAEEISSMVLIKMKEIAEAYLGSTIKNAVVTVPAYFNDSQRQATKDAGVISGLNVMRIINEPTAAAIAYGLDKKATSSGEKNVLIFDLGGGTFDVSLLTIEEGIFEVKATAGDTHLGGEDFDNRMVNHFVQEFKRKNKKDISGNARALRRLRTACERAKRTLSSTAQTTIEIDSLYEGIDFYTTITRARFEELNMDLFRKCMEPVEKCLRDAKMDKSTVHDVVLVGGSTRIPKVQQLLQDFFNGKELCKSINPDEAVAYGAAVQAAILSGEGNEKVQDLLLLDVTPLSLGLETAGGVMTVLIPRNTTIPTKKEQVFSTYSDNQPGVLIQVYEGERTRTRDNNLLGKFELSGIPPAPRGVPQITVCFDIDANGILNVSAEDKTTGQKNKITITNDKGRLSKEEIEKMVQEAEKYKAEDEEHKKKVEAKNTLENYAYNMRNTIKDDKIASKLSADDKKKIEDAIEQAIQWLDGNQLAEADEFEDKMKELESICNPIIAKMYQGAGGDAGGAMDEDGPAAGSGSGAGPKIEEVD*

>Glyma12g28750 MACSSAQIHGLGTPSSRTLFLGQRLNTKAAFIKLKSTPRRLRPLRVVNEKVVGIDLGTTNSAVAAMEGGKPTIITNAEGQRTTPSVVAYTKNGDRLVGQIAKRQAVVNPENTFFSVKRFIGRKMSEVDEESKQVSYRVIRDDNGNVKLDCPAIGKQFAAEEISAQAGVLAGDVSDIVLLDVTPLSLGLETLGGVMTKIIPRNTTLPTSKSEVFSTAADGQTSVEINVLQGEREFVRDNKSLGSFRLDGIPPAPRGVPQIEVKFDIDANGILSVTAIDKGTGKKQDITITGASTLPSDEVERMVNEAEKFSKEDKEKRDAIDTKNQADSVVYQTEKQLKELGDKVPGPVKEKVEAKLGELKDAISGGSTQAIKDAMAALNQEVMQLGQSLYNQPGAAGAGGPTPPPGADSGPSESSGKGPDGDVIDADFTDSK*

>Glyma13g10700 MASKVALMALFSVALLFSPSQSAVFSVDLGSESVKVAVVNLKPGQSPISVAINEMSKRKSPALVSFHDGDRLLGEEAAGLAARYPQKVYSQMRDLIAKPYASAQRILDSMYLPFDAKEDSRGGVSFQSENDDAVYSPEELVAMVLGYTVNLAEFHAKIQIKDAVIAVPPYMGQAERRGLLAAAQLAGINVLSLINEHSGAALQYGIDKDFSNESRHVIFYDMGASSTHAALVYFSAYKGKEYGKSVSVNQFQVKDVRWDPELGGQHMELRLVEYFADQFNAQVGGGIDVRKFPKAMAKLKKQVKRTKEILSANTAAPISVESLHDDVDFRSTITREKFEELCEDIWEKSLLPVKEVLENSGLSLEQIYAVELIGGATRVPKLQAKLQEFLRRKELDRHLDADEAIVLGAALHAANLSDGIKLNRKLGMIDGSLYGFVVELNGPDLLKDESSRQLLVPRMKKVPSKMFRSINHNKDFEVSLAYESENHLPPGVTSPEIARYQISGLTDASEKYSSRNLSSPIKTNIHFSLSRSGILSLDRADAVIEITEWVEVPRKNLTIENSTVSSNVSAESAAGNSSEENNESVQTDSGINKTSNISSEEQAAAEPATEKKLKKRTFRVPLKIVEKITGFGMSLSQDFLAEAKRKLQVLDKKDADRKRTAELKNNLEGYIYTTKEKIETLEEFEKVSTSEERQSFIEKLDQVQDWLYTDGEDANATEFQERLDQLKAVGDPIFFRLKELTARPAAVEHANKYIDELKQIVEEWKAKKSWLPQERVDEVIKSSEKLKNWLDEKEAEQTKTSGFSKPAFTSEEVYLKVLDLQTKVASINRIPKPKPKVQKPVKNETESSEQNTENSDSNSADSSSSSDSSVNSSEGTSEETVTEQTEGHDEL*

>Glyma13g19330 MAGKGEGPAIGIDLGTTYSCVGVWQHDRVEIIANDQGNRTTPSYVGFTDTERLIGDAAKNQVAMNPINTVFDAKRLIGRRFSDASVQSDIKLWPFKVLSGPAEKPMIQVSYKGEDKQFAAEEISSMVLMKMREIAEAYLGSSIKNAVVTVPAYFNDSQRQATKDAGVIAGLNVMRIINEPTAAAIAYGLDKKATSVGEKNVLIFDLGGGTFDVSLLTIEEGIFEVKATAGDTHLGGEDFDNRMVNHFVQEFKRKNKKDISGNPRALRRLRTACERAKRTLSSTAQTTIEIDSLYEGIDFYSTITRARFEELNMDLFRKCMEPVEKCLRDAKMDKRTVHDVVLVGGSTRIPKVQQLLQDFFNGKELCRASILMRLWHMVLQFKLLS*

>Glyma13g19331 MAGKGEGPAIGIDLGTTYSCVGVWQHDRVEIIANDQGNRTTPSYVGFTDTERLIGDAAKNQVAMNPINTVFDAKRLIGRRFSDASVQSDIKLWPFKVLSGPAEKPMIQVSYKGEDKQFAAEEISSMVLMKMREIAEAYLGSSIKNAVVTVPAYFNDSQRQATKDAGVIAGLNVMRIINEPTAAAIAYGLDKKATSVGEKNVLIFDLGGGTFDVSLLTIEEGIFEVKATAGDTHLGGEDFDNRMVNHFVQEFKRKNKKDISGNPRALRRLRTACERAKRTLSSTAQTTIEIDSLYEGIDFYSTITRARFEELNMDLFRKCMEPVEKCLRDAKMDKRTVHDVVLVGGSTRIPKSINPDEAVAYGAAVQAAILSGEGNEKVQDLLLLDVTPLSLGLETAGGVMTVLIPRNTTIPTKKEQVFSTYSDNQPGVLIQVYEGERTRTRDNNLLGKFELSGIPPAPRGVPQITVCFDIDANGILNVSAEDKTTGQKNKITITNDKGRLSKEEIEKMVQEAEKYKSEDEEHKKKVEAKNALENYSYNMRNTIKDEKIGGKLDPADKKKIEDAIEQAIQWLDSNQLGEADEFEDKMKELESICNPIIAKMYQGGAGPDVGGAMDDDVPAAGSGAGPKIEEVD*

>Glyma13g29580 MAPGNVKAIGIDLGTTYSCVAVWQHNHVEVIPNDQGNRTTPSYVAFTDTQRLLGDAAINQRSMNPQNTVFDAKRLIGRRFSDQSVQQDMKLWPFKVVPGNRDKPMISRQATKDAGKIAGLNVLRIINEPTAAAIAYGLNKKGWREGMFKVKATVGDTHLGGVDFDNKMVDYLVSIFKRRYKKDIGENPKALGRLRSACEKAKRILSSSSQTTIELDSLCGGVDLHANFSRALFEELNKDLFMKCMETVEKCLKEARIAKSQVHEFVLVGGSTRIPKVQQLLKDMFSVNGNKELCKSINPDEAVAYGAAVQAAILSGEGDKKVEDLLLLDVMPLSLGIETDGGEMSVLIPKNTMIPTKRESVFSTFSDNQTSVLIKVFEGERAKTEDNFLLGKFELSGFTPSPRGVPQINVGFDVDVDGIVEVTARDRSTGLKKKITISNKHGRLSPEEMRRMVRDAVRYKAEDEEVRNKVRIKNLLENYAFEMRDRVKNLEKVVEETIEWLDRNQLAETDEFEYKRQELEEKVLKFM

>Glyma13g29590 MFPWLLFMKACLRLRPVLGDTHLGGVDFDNRLVNHLVNVFREKHKKDISGNAKALARLRSECEKAKRILSSTSQTTIELDCLYEGLDLYAPVTRALFNELNKDLFMKCMDTVEKCLLEARIDKIQVHEIILVGGSTRIPKVQQLLKDMFSVNGNTKELCKGINPDEAVAYGAAVQAAILSGEGDKKVEELLLLDVMPLSLGFEGAGGVMSVLIPKNTMIPTKKERICSTFYDNQKSFNVKVFEGERVKTKDNFFLGKFVLKGFDPLPKGVPQINVIFDVDADGIVEVTAEDKATGIEKKITINNKHGRLNPEEIRRMVRDSKKYKAEDELAKKKVKAKNALENYAYEMRERAKKIEEAVEETIEWLECNQLAEIGEFDYKKQELGRCPPNGWNHRNGCSGSACGGLVRDSSGCYLGGFTVNLGNTSVTLAELWGVVHGLKLAWDLGCKKVKVDIDSGNALGLVRHGPVANDPAFALVSEINELVRKEWLVEFSHVFRESNRAADKLAHLGHSHSLESGAKRFSDPPSALVAILQDDLAGLAKQRGVN*

>Glyma13g29591 FPCDSLSSFTAKLFQWHKKVKAIGIDLGTTYSFFAVWQHNRVEVISNDQGNRTTPSYVAFSDTQRLLGDSAMNQRSMNPKNTVFDAKRLIGRRFSDQTVQQDMKMWPFKVVPGNKDKPMIAVTYKGEEKLLAPEEISSMVLYKMKEVAEGYLGHFIKDAVITVPAYFSNAQRQATKDAGKIAGMNVLRIINEPTAAAIAYGLDKKGLRVGEQNVLVFDLGGGTFDVSLVTIYEGMFKVKAKKHKKDISGNAKALARLRSECEKAKRILSSTSQTTIELDCLYEGLDLYAPVTRALFNELNKDLFMKCMDTVEKCLLEARIDKIQVHEIILVGGSTRIPKVQQLLKDMFSVNGNTKELCKGINPDEAVAYGAAVQAAILSGEGDKKVEELLLLDVMPLSLGFEGAGGVMSVLIPKNTMIPTKKERICSTFYDNQKSFNVKVFEGERVKTKDNFFLGKFVLKGFDPLPKGVPQINVIFDVDADGIVEVTAEDKATGIEKKITINNKHGRLNPEEIRRMVRDSKKYKAEDELAKKKVKAKNALENYAYEMRERAKKIEEAVEETIEWLECNQLAEIGEFDYKKQELGSVYIKFI*

>Glyma13g32790 MASLLRSLRRRDVASASLSAYRSLTGSTKPAYVAHNWSSLSRPFSSRPAGNDVIGIDLGTTNSCVSVMEGKNPKVIENSEGARTTPSVVAFNQKGELLVGTPAKRQAVTNPTNTLFGTKRLIGRRFDDAQTQKEMKMVPFKIVKAPNGDAWVEANGQQYSPSQIGAFVLTKMKETAEAYLGKSISKAVITVPAYFNDAQRQATKDAGRIAGLDVQRIINEPTAAALSYGMNNKEGLIAVFDLGGGTFDVSILEISNGVFEVKATNGDTFLGGEDFDNALLDFLVNEFKRTESIDLSKDRLALQRLREAAEKAKIELSSTSQTEINLPFITADASGAKHLNITLTRSKFEALVNHLIERTKVPCKSCLKDANISIKDVDEVLLVGGMTRVPKVQEVVSEIFGKSPSKGVNPDEAVAMGAAIQGGILRGDVKELLLLDVTPLSLGIETLGGIFTRLINRNTTIPTKKSQVFSTAADNQTQVGIKVLQGEREMAADNKMLGEFDLVGIPPAPRGLPQIEVTFDIDANGIVTVSAKDKSTGKEQQITIRSSGGLSDDEIEKMVKEAELHAQKDQERKALIDIRNSADTTIYSIEKSLGEYREKIPSEVAKEIEDAVSDLRQAMSGDNVDEIKSKLDAANKAVSKIGEHMSGGSSGGSSAGGSQGGDQAPEAEYEEVKK*

>Glyma13g43630 MSVVGFDFGNESCIVAVARQRGIDVVLNDESKRETPAIVCFGDKQRFLGTAGAASTMMNPKNSISQIKRLIGRQFADPELQQDIKTFPFVVTEGPDGYPLIHARYLGESRTFTPTQVFGMMLSNLKEIAEKNLNAAVVDCCIGIPLYFTDLQRRAVLDAATIAGLHPLRLFHETTATALAYGIYKTDLPENDQLNVAFVDVGHASMQVCIAGFKKGQLKVLSQSYDRSLGGRDFDEVLFNHFAAKFKEEYKIDVFQNARACLRLRAACEKLKKVLSANPEAPLNIECLMDEKDVRGFIKRDEFEQLSLPILERVKGPLEKALAEAGLTVENVHMVEVVGSGSRVPAINKILTEFFKKEPRRTMNASECVARGCALQCAILSPTFKVREFQVNESFPFSISLSWKAPSSDAQESGPDNKQSTLVFPKGNPIPSVKALTIYRSGTFSIDVQYDDVSGLQTPAKISTYTIGPFQSTKNEKAKVKVKVRLNVHGIISVESATLLEEEEEIEVPVYKEPAGENSKMETDEAPADAAAAAATPSTNDNDVSMQDANTKATANAPGAENGTPEAGDKPVQMDTDTKVEAPKKKVKKINIPVVELVYGAMAATDVQKAVEKEFEMALQDRVMEETKDKKNAVEAYVYDMRNKLNDKYQEFVIDSEREAFTAKLQEVEDWLYEDGEDETKGVYIAKLEELKKQGDPIEERYKEYMERGTVIDQLAYCINSYREAAMSNDPKFDHIDINEKQKVLNECVEAENWLREKKQHQDSLPKYATPVLLSADVRKKAEAVDRFCKPIMTKPKPLPPKPATPEAPATPPPQGGEQQQQPPQENPNASTNENAGDNANPAPPPASAEPMETDKPENTGSA*

>Glyma14g02740 MSGVGIDIGNENCVIAAVKQRVIDVLLNDESKRETPGVVCFGEKQRFIGSAGAVSAMMHPKSTISQVKRLIGRRFTDPDVQNDLKLLPVETSEGPDGGILIRLKYLKEIHAFTPVQIVAMLFAHLKTIAEKDFGTAVSDCVIGVPSYFTNLQRQAYLDAAAIVGLKPLRLIHDCTATGLSYGVYKTDIPNAAHIYVAFVDIGHCDTQVSIAAFQAGQMKILSHAFDSSLGGRDFDEVLFSHFAARFKEQYSIDVYSNGRACRRLRVACEKLKKVLSANAVADLSIECLMDEKDVKGFIKREEFENLASGLLEKFNIPCNKALADAGMTVEKINSVELVGSGSRIPAITNLLTSLFKRELSRTLNASECVARGCALQCAMLSPIFRVKEYEVQDSIPFSIGLSCDGSPICEGSDGVLFPKGQPIPSVKILTFQCSNLLHLEAFYANPDELPPGTSPKISCFTIDPFHGSHGSKARIKVRVQLNLHGIISIESATLMEDHVDDSVTTGDYHSNSEAMNVEPVSETVENVTEDSINKKCEAPRHLADGTKKDKANRRLHVPVSENIYGGMTKAEILEAQEKELQLADQDRTIELTKDRKNSLESYIYETRSKLFSTYLSFSSEHERKDISRSLKATEDWLYDDGDDETVDAYSAKLEDLKQLVDPIEFRYKDTEARPQATRDLLSCIVEYRMSADSLPPQDKEQIINECNKAEQWLREMRQQQDLYPKNFDPVLLSSDIKSKTEDLNSVCQQILKSKGSPFPKDKGEDKQNTSNHQ

>Glyma15g01750 MSVVGFDFGNESCIVAVARQRGIDVVLNDESKRETPAIVCFGDKQRFLGTAGAASTMMNPKNSISQIKRLIGRQFSDPELQRDLKTFPFVVTEGPDGYPLIHARYLGEARTFTPTQVFGMMLSNLKEIAEKNLNAAVVDCCIGIPLYFTDLQRRAVLDAATIAGLHPLRLFHETTATALAYGIYKTDLPENDQLNVAFVDVGHASMQVCIAGFKKGQLKVLSQSYDRSLGGRDFDEVLFNHFAAKFKEEYKIDVFQNARACLRLRAACEKLKKVLSANPEAPLNIECLMDEKDVRGFIKRDEFEQLSLPILERVKGPLEKALAEAGLTVENVHMVEVVGSGSRVPAINKILTEFFKKEPRRTMNASECVARGCALQCAILSPTFKVREFQVNESFPFSISLSWKGPSSDAQESGPNNTQRTLVFPKGNPIPSVKALTIYRSGTFSIDVQYDDVSELQTPAKISTYTIGPFQSTITEKAKVKVKVRLNLHGIVSVESATLLEEEEIEVPVSKEPAGENTKMETDEAPANVAAPPSTNDNDVNMQDANSKATADAPGSENGTPEAGDKPVQMDTDTKVEAPKKKVKKINIPVVELVYGAMAAADVQKAVEKEFEMALQDRVMEETKDKKNAVEAYVYDTRNKLNDKYQEFVVDSERESFTAKLQEVEDWLYEDGEDETKGVYIAKLEELKKQGDPIEERYKEYMERGTVIDQLVYCINSYREAAMSNDPKFDHIDINEKQKVLNECVEAENWLREKKQQQDSLPKYVTPVLLSADIRKKAEAVDRFCKPIMMKPKPPPPKPATPEAPATPPPQGGEQPQQQQQQPPEENPNASTNEKAGDNANPAPPPASAEPMETDKPENTGSA*

>Glyma15g06530 MASLLRSLRRRDVASASLSAYRSLTGSTKPAYVAHNWSSLSRPFSSRPAGNDVIGIDLGTTNSCVSVMEGKNPKVIENSEGARTTPSVVAFNQKGELLVGTPAKRQAVTNPTNTLFGTKRLIGRRFDDAQTQKEMKMVPFKIVKAPNGDAWVEANGQQYSPSQIGAFVLTKMKETAEAYLGKSISKAVITVPAYFNDAQRQATKDAGRIAGLDVQRIINEPTAAALSYGMNKKEGLIAVFDLGGGTFDVSILEISNGVFEVKATNGDTFLGGEDFDNALLDFLVNEFKRTESIDLAKDRLALQRLREAAEKAKIELSSTSQTEINLPFITADASGAKHLNITLTRSKFEALVNHLIERTKAPCKSCLKDANISIKDVDEVLLVGGMTRVPKVQEVVSEIFGKSPSKGVNPDEAVAMGAAIQGGILRGDVKELLLLDVTPLSLGIETLGGIFTRLINRNTTIPTKKSQVFSTAADNQTQVGIKVLQGEREMAADNKMLGEFDLVGIPPAPRGLPQIEVTFDIDANGIVTVSAKDKSTGKEQQITIRSSGGLSEDEIEKMVKEAELHAQKDQERKALIDIRNSADTTIYSIEKSLGEYRDKIPSEVAKEIEDAVSDLRKAMSEDNVDEIKSKLDAANKAVSKIGEHMSGGSSGGSSAGGSQGGDQAPEAEYEEVKK*

>Glyma15g09420 MATKKVKAIGIDLGTSYSCVAVWQHNRIEVISNDQGNCTTPSYVAFNDNQRLLGDSSMSQRSMNPQNTVFDDKQTYYLHRPFKVVPDNRDKPMVTVTYKGEEKLLAPEEISSMVLFKMKEVVEAHLGHFVKDAVITVPAYFSNAQRQATKDVGKIAGLNVLRIISEPTAAAIAYGLDRKGLRVGEQNVLVFDLGGGTFDVSLVTIYEGMFKVKASVGDTHLGGVDFDNKLVNHLVNVFREKHKKDISGNAEALVRLRSACEKAKRILSSTAQTTIELDCLYEGVDLYATVTRALFEELNKDLFMKCMETVEKCLLEARSDKIQVHEIVLVGGSTRIPKVQQLLKDMFSLNGTTKELCKGINPDEAVAYGAAVQAAILSGEGDKKVEELLLLDVMPISIGFEGAGGVMSVLIPKNTAIPTKKERVCSIFYDNQKSLTVKVFEGEQVKTKDNFFLGKFILYRFDPLPKGVSQISVIFDVDADGIVEVTAEDQAKGLKKKITINSKHGRLSPEEIRRMVRDSKRYKAEDEVAKKKVKAKNTLENYAYEMRERAKKIEEAVEETIEWLECNQLAEIEEFDCKKQELG

>Glyma15g09430 MAPRKVKAMGIDLGTTYSCVAVWNHNRVEVIPNDQGNRTTPSYVAFTDTQRLLGDAAINQRSMNPQNTVFDAKRLVGRRFSDQSVQQDIKLWPFKVVPGARDKPMIAVTYKDEEKLLAAEEISSMVLFKMKEVAEAHLGHFVKDAVITVPAYFSNAQRQATKDAGKIAGLNVLRIINEPTAAAIAYGLDKKGWREGEQNVLVFDLGGGTFDVSLVTIDEGMFKVKATVGDTHLGGVDFDNKLVNYLVGIFKRRYKKDIGENPKALGRLRSACEKAKRILSSSSQTTIELDSLCGGADLHAIVTRALFEELNKDLFMKSQVHELVLVGGSTRIPKVQQLLKDMFSVNGNKELCKSINPDEAVAYGAAVQAAILSGEGDKKVEELLLLDVMPLSLGIETDAGEMSVLIPKNTMIPTKRESVFSTFSDNQTSVLIKVFEGEHAKTEDNFLLGKFELSGFTPSPRGVPQINVGFDVGVDGIVEVTARDRSTGLKKKITISNKHGRLSPEEMRRMVRDAEKYKAEDEEVSNKVRAKNLLENYAFEMRDRVKNLEKVVEETIEWLDRNQLAETDEFEYKKQELEEKFRKFR

>Glyma15g10280 SCVGVWLEQHNRVEIIHNQQGHKTTPSFVAFTDNQRLIGDAAKNQAVTNPENTVFDAKRLIGRKYSDPIIQKEKTLWSFKVVAGINDKPMIVVKYKGQEKQIYAGAIAGLNVMSIINEPTATDIAYGLNKRTNCVGERNIFIFDLGGGTLDAALLTIKDVYEVKATAGKNDFKKKNKVDISGNPRALRRLRTSCERAKRILPTLRKFEEIDMELFEECMETVDKCLTDSKMGKGSVRDVVLVGGSSRISKVQELLQDLFDGKDLCKSINPDEAVPYGASVQAAMLSEGIKNVPDLVLLGVTPLSLGILTKGDVMSVVIPRNTRIPVRKTQVCCNLDNQKRVPFSVYEGERARANDNNLLGSFVLSGLPPSPRGHPLDVSFAIDVNGILSVSTEEKTSGNKNEITIINDKDRLSTEEIGRLIQEAEKYRAEDKKFLRKANAMNSLGYYVYKMRNVLKKDISSLCSKEREKIDYAITKATNLLDDSKYQYEVEVFEDHHKELASFFESIASKIG*

>Glyma16g00410 MACSSAQIHGLGTPSFSRTLFLGQRLNTKAAFIKVKSAPTPRRLRPLRVVNEKVVGIDLGTTNSAVAAMEGGKPTIITNAEGQRTTPSVVAYTKNGDRLVGQIAKRQAVVNPENTFFSVKRFIGRKMSEVDEESKQVSYRVIRDDNGNVKLDCPAIGKQFAAEEISAQVLRKLVDDASKFLNDKVTKAVVTVPAYFNDSQRTATKDAGRIAGLEVLRIINEPTAASLAYGFEKKNNETILVFDLGGGTFDVSVLEVGDGVFEVLSTSGDTHLGGDDFDKRIVDWLASNFKRDEGIDLLKDKQALQRLTETAEKAKMELSTLTQTNISLPFITATADGPKHIETTITRAKFEELCSDLLDRLRTPVENSLRDAKLSFKDLDEVILVGGSTRIPAVQELVKKLTGKDPNVTVNPDEVVALGAAVQAGVLAGDVSDIVLLDVTPLSLGLETLGGVMTKIIPRNTTLPTSKSEVFSTAADGQTSVEINVLQGEREFVRDNKSLGSFRLDGIPPAPRGVPQIEVKFDIDANGILSVAAIDKGTGKKQDITITGASTLPSDEVERMVNEAEKFSKEDKEKRDAIDTKNQADSVVYQTEKQLKELGDKVPGPVKEKVEAKLGELKDAISGGSTQAIKDAMAALNQEVMQLGQSLYNQPGAAGAGGPTPPGADSGPSESSGKGPDGDVIDADFTDSK*

>Glyma17g08020 MATKEGKAIGIDLGTTYSCVGVWQNDRVEIIPNDQGNRTTPSYVAFTDTERLIGDAAKNQVAMNPQNTVFDAKRLIGRRFSDSSVQNDMKLWPFKVVAGPGDKPMIVVNYKGEEKKFSAEEISSMVLVKMREVAEAFLGHAVKNAVVTVPAYFNDSQRQATKDAGAISGLNVLRIINEPTAAAIAYGLDKKASRKGEQNVLIFDLGGGTFDVSILTIEEGIFEVKATAGDTHLGGEDFDNRMVNHFVSEFKRKNKKDISGNARALRRLRTACERAKRTLSSTAQTTIEIDSLYEGIDFYATITRARFEEMNMDLFRKCMEPVEKCLRDAKIDKSQVHEVVLVGGSTRIPKVQQLLQDFFNGKELCKSINPDEAVAYGAAVQAAILSGEGDEKVQDLLLLDVTPLSLGLETAGGVMTVLIPRNTTIPTKKEQIFSTYSDNQPGVLIQVFEGERARTKDNNLLGKFELTGIPPAPRGVPQINVCFDIDANGILNVSAEDKTAGVKNKITITNDKGRLSKEEIEKMVKDAERYKAEDEEVKKKVEAKNSLENYAYNMRNTIKDEKIGGKLSPDEKQKIEKAVEDAIQWLEGNQMAEVDEFEDKQKELEGICNPIIAKMYQGAAGPGGDVPMGADMPAAGAGPKIEEVD*

>Glyma17g11650 MKRMIALGFEGSANKIGVGVVTLDGTILSNPRHTYITPPGQGFLPRETAQHHLQHVLPLVKSALEVAQIAPQDIDCLCYTKGPGMGAPLQVSAIVVRVLSQLWKKPIVAVNHCVAHIEMGRIVTGADDPVVLYVSGGNTQVIAYSEGRYRIFGETIDIAVGNCLDRFARVLTLSNDPSPGYNIEQLAKKGEKFIDLPYTVKGMDVSFSGILSYIEATAAEKLKNNECTPADLCYSLQETLFAMLVEITERAMAHCDTKDVLIVGGVGCNERLQEMMRIMCSERGGRLFATDDRYCIDNGAMIAYTGLLEFAHGASTPLEDSTFTQRFRTDEVKAIWREANLENLNGLAEKSI*

>Glyma17g14280 MDASKLNQLKHFIEQCKSNPSLLADPSLSFFRDYLQSLGAKLPESAYSESTGVERDEDIEDLTEEHEKVEEEEEEEDDVIIESDVELEGETCEPDDDPPQKMGDPSVEVTEENRDASQMAKIKAMDAISEGKLEEAIENLTEAISLNPTSAIMYGTRASVYIKMKKPNAAIRDANAALEINPDSAKGYKSRGIARAMLGQWEEAAKDLHVASKLDYDEEINAVLKKVEPNAHKIEEHRRKYERLHKEREDKKKERERQRRRAEAQAAYEKAKKQEQSSSSRNPGGMPGGFPGGFPGAGGMPGGFPGAGGMPGGFPGAGGMPGGFPGAGGMPGGFPGAGGMPGNIDFSKILSDPELMASFGDPEIMAALQDVMKNPANFAKHQSNPKVAPVIAKMMTKLGGGPK*

>Glyma18g05480 MEKLNLALVSSPKPLMLGHVPAIDATSRDVFRRKHFSFGRVLIAPHRCRFRVSALSSSHRNPKSVQEKLIVKHFASISSSNTQEATSTGVNPQLSPSSTIGSPLFWIGVGVGLSALFSVVASRLKKYAMQQAFKTMMGQMNSQNNQFGNAAFSPGSPFPFPMPTAAGPTAPASSATTQSRAPSASSASQSTITVDIPAAKVEVAPTTNVKDEVEVKNEPKKIAFVDVSPEETVQESPFESFKDDESSSVKEARVPDEVSQNGAPSNQGFGDFPGSQSTKKSVLSVDALEKMMEDPTVQKMVYPYLPEEMRNPTTFKWMLQNPQYRQQLEEMLNNMGGSTEWDSRMMDTLKNFDLNSPEVKQQFDQIGLSPEEVISKIMANPEVAMAFQNPRVQAAIMDCSQNPMNITKYQNDKEVMDVFNKISELFPGVGSP

>Glyma18g05610 MTKSKEDHGIAIGIDLGTTYSCVAVWQEHHCRVEIIHNDQGNNTTSFVAFTDDERLLKIRLLPIQRTMSLDKMLWPFKVVAGTHDKPMIILNYKDEEKHFCAEEISSIVLAKMWEIAEAFLEKRVKNAVIAGLNVMRIINEPTPAAIAYGLNRRTNNCVGERKIFIFDLGGGTFDVSLLTHKGKIFQVKVTTGNGHLGGEEIDNRMVDYFVKEIKRKKKVDISGNPKALRRLKTACERSKRILSCAVATHIETYALSDGIDFCSSTTRARFEEINMDLFKECMETVDKCLTDAEMDKSSVHDVILVGGSSRIPKVQELLPGFFNGKDLVYDNQSSVGIKVYEDERTRASDNNLLGSFSLSGLPPAPHGHPFDVCFAIDENGILSVSAKEKTTGNSNKIVITNERERFIQMENALENGNLSSKLCSEDKEKISSAITKATKLLEGENQNGEIDVFENLFERVIGKFDF*

>Glyma18g11520 MSVVGFDIGNENCVIAVVRQRGIDVLLNYESKRETPAVVCFGEKQRILGSAGAASAMMHIKSTISQIKRLIGRKFADPDVEKELKMLPVETSEGQDGGILIHLKYMGEIHVFTPVQLLSMLFAHLKTMTEKDLEMLISDCVIGIPSYFTDLQRRAYLDAAKIAGLKPLRLIHDCTATALSYGMYKKDFGSAGPVNVAFIDIGHCDTQVSIASFEFGKMKILSHAFDRSLGGRDFDEVIFSHFAAKFKEEYHIDVYSNTKACFRLRAACEKLKKVLSANLEAPLNIECLMDEKDVKGFITREEFEKLASGLLERVSIPCRRALIDANLTEEKISSVELVGSGSRIPAISTLLTSLFKREPSRQLNASECVARGCALQCAMLSPIYRVREYEVKDVIPFSIGLSSDEGPVAVRSNGVLFPRGQPFPSVKVITFRRSDLFHLEAFYANPDELPPGTSPIISCVTIGPFHGSHGSKIRVKVRVPLDLHGIVSIESATLIKDDSVMAGDYHSNSDAMDIDPISETVTNGFEDNTNKNLESPCSSADGTRKDNRRLNVPVNENVYGGMTKAEISEAREKELQLAHQDRIVEQTKEKKNSLESYVYDMRSKLFHTYRSFASEQEKDDISRTLQETEEWLYEDGVDETEHAYSSKLEDLKKLVDPIENRYKDDKERVQATRDLSKCILKHRASADSLPTQDKELIINECNKVEQWLEEKIQQQESFPRNTDPILWSSDIKSKTEELNLKCQQILGSKASPSPEDKDKPDTFNDP*

>Glyma18g13077 MAFACSRIAQRTSISSIKSAIKSNIRASSFSKPASSFSPICQSLLARILQELRCVQSMLPLHSTVAAARMMSCLTFKSCRALSPEFFKKEPRWTMCAILSPTFKVREFQCMRMMGLEWKDIAFVQKSNLESYIQ*

>Glyma18g52470 MATNGKTPAIGIDLGTTYSCVAVWRHDRVEIIVNDQGNRTTPSYVAFNNTQRMIGDAAKNQAATNPTNTSTPVIGIDLGTTYSCVAVWQHDRVVIITNDQGNRTTPSCVAFKNTQRMIGDAAINQAAANPTNTVFGAKRLIGRRFSNPEVQSDMKQWPFKVIADVNDKPMIAVNYNCEERHFSAEEISSMVLEKMRAIAESFLGSTVKNAVITVPAYFNDSQRQATKDAGAIAGLNVLRIINEPTAAAIAYRLERKNCNNERRNVFVFDLGGGTLDVSLLVFEKDYIRVKATSGDTHLGGEDFDNNMVTYCVKEFQRKNKKDISGNERALRRLRTACEKAKRILSSTVMTTIEVDSLYDGIDFHSSISRAKFEELNMDYLNKCMEFVEKCLIDAKMDKSSVHDVVLAGGSTRIPKLQQLLSDFFDGKDLCKCINADEAVAYGAAVHASMLNGESSEKVQNTLPREVTPLSLGLEKEGGIMKVIIPRNTSIPTKMEDVFTTHLDNQINILIHVYEGERQRTRDNNLLGKFVLEIPPVPRGVPQIIVCFEVDDEGILHVSAKENSLGITKKVTIINDKGRLSEEEIKRMISEAERYKAEDEMYRKKVEARYALEKYAYNIRNAIKHKGISLKLSPEDKEKINDAVDRALEWLEVSVDAEKEDVDNFRGNLSSVFDTIMVKMIKGEDNGAPPESLVINIGKIWSPGSVFSCYR*

>Glyma18g52471 MATNESTPVIGIDLGTTYSCVAVWQHDRVVIITNDQGNRTTPSCVAFKNTQRMIGDAAINQAAANPTNTVFGAKRLIGRRFSNPEVQSDMKQWPFKVIADVNDKPMIAVNYNCEERHFSAEEISSMVLEKMRAIAESFLGSTVKNAVITVPAYFNDSQRQATKDAGAIAGLNVLRIINEPTAAAIAYRLERKNCNNERRNVFVFDLGGGTLDVSLLVFEKDYIRVKATSGDTHLGGEDFDNNMVTYCVKEFQRKNKKDISGNERALRRLRTACEKAKRILSSTVMTTIEVDSLYDGIDFHSSISRAKFEELNMDYLNKCMEFVEKCLIDAKMDKSSVHDVVLAGGSTRIPKLQQLLSDFFDGKDLCKCINADEAVAYGAAVHASMLNGESSEKVQNTLPREVTPLSLGLEKEGGIMKVIIPRNTSIPTKMEDVFTTHLDNQINILIHVYEGERQRTRDNNLLGKFVLEIPPVPRGVPQIIVCFEVDDEGILHVSAKENSLGITKKVTIINDKGRLSEEEIKRMISEAERYKAEDEMYRKKVEARYALEKYAYNIRNAIKHKGISLKLSPEDKEKINDAVDRALEWLEVSVDAEKEDVDNFRGNLSSVFDTIMVKMIKGEDNGAPPGAVASSGSKSGKNRWLSILAKFGLQAVYSAVTGDIIGFVSVIVDCLAN*

>Glyma18g52480 MATNGKTPAIGIDLGTTYSCVAVWQRDRVEIIANDQGNRTTPSYVAFNNTQRMIGDAAKNQAATNPTNTVFDAKRLIGRRFSDQEVQSDMELWPFKVIADVNGKPMIAVDYNCEKKQFSAEEISSMVLAKMLDIAESFLGSTVKNAVITVPAYFNDSQRQATKDAGKIAGLNVLRILHEPTAAAIAYRLEMKNCNNDRRNVFVFDLGGGTLDVSLLVFEKDHIRVKATTGDTHLGGEDFDNNMVTYCVKEFKRKNKMDISGNKRALRRLRTACEKAKRILSCSTMTTIEVDSLYDGIDFHSSISRAKFEELNKDYLNKCIEFVGKCLIDAKMDKSSVHDVVLAGGSTRIPKLQQLLSDFFDGKDLCKCINADEAVAYGAAVHAYMLNGESSEKVQNASLWEVTPLSLGLQEDGGIMKVIIPRNTSIPTKMEDVLTTHFDNQTNILIHVYEGERKRTRDNNLLGKFVLEIPPVPRGVPQISVCFELDYDGILHVSAEEKSRGISKKLAITNDKGRLSKKEIERMISEAEKYKAEDEMYRNKVQSRHALEKYAYNMRDAINIKEISLKLSPEDKKNINDAIDSALEWLEVSMDANPNDFDNMRSTLSSVFNPVIVKMIKDEDNVAPPDTVASSGSNSVKNGLLSILANFALDAVYSAATGDIIGFASVIVDCLSI*

>Glyma18g52610 MAGKGDGPAIGIDLGTTYSCVGVWQHDRVEIIANDQGNRTTPSYVAFTDSERLIGDAAKNQVAMNPVNTVFDAKRLIGRRFSDASVQSDMKLWPFKVIPGPADKPMIVVNYKGEDKQFSAEEISSMVLMKMREIAEAYLGSTVKNAVVTVPAYFNDSQRQATKDAGVIAGLNVMRIINEPTAAAIAYGLDKKATSVGEKNVLIFDLGGGTFDVSLLTIEEGIFEVKATAGDTHLGGEDFDNRMVNHFVQEFKRKHKKDINGNPRALRRLRTACERAKRTLSSTAQTTIEIDSLYEGVDFYTTITRARFEELNMDLFRKCMEPVEKCLRDAKMDKSTVHDVVLVGGSTRIPKVQQLLQDFFNGKELCKSINPDEAVAYGAAVQAAILSGEGNEKVQDLLLLDVTPLSLGLETAGGVMTVLIPRNTTIPTKKEQVFSTYSDNQPGVLIQVYEGERARTRDNNLLGKFELSGIPPAPRGVPQITVCFDIDANGILNVSAEDKTTGQKNKITITNDKGRLSKDEIEKMVQEAEKYKAEDEEHKKKVDAKNALENYAYNMRNTIKDEKIASKLSDDDKKKIEDAIESAIQWLDGNQLAEADEFEDKMKELESICNPIIAKMYQGAGAPDMAGGMDEDVPPSGSGGAGPKIEEVD*

>Glyma18g52650 MAGKGEGLAIGIDLGTTYSCVGVWQHDRVEIIANDQGNRTTPSYVAFTDTERLIGDAAKNQVAMNPINTVFDAKRLIGRRVSDPSVQSDMKLWPFKVTAGAGEKPMIGVNYKGEEKQFAAEEISSMVLTKMREIAEAYLGSTVKNAVVTVPAYFNDSQRQATKDAGVIAGLNVMRIINEPTAAAIAYGLDKKATSVGEKNVLIFDLGGGTFDVSLLTIEEGIFEVKATAGDTHLGGEDFDNRMVNHFVQEFKRKNKKDITGNPRALRRLRTSCERAKRTLSSTAQTTIEIDSLFEGIDFYSTITRARFEELNMDLFRKCMEPVEKCLRDAKMDKSSVHDVVLVGGSTRIPKVQQLLQDFFNGKDLCKSINPDEAVAYGAAVQAAILSGEGNEKVQDLLLLDVTPLSLGLETAGGVMTVLIPRNTTIPTKKEQVFSTYSDNQPGVLIQVYEGERTRTRDNNLLGKFELSGIPPAPRGVPQITVCFDIDANGILNVSAEDKTTGQKNKITITNDKGRLSKEEIEKMVQEAEKYKSEDEEHKKKVEGKNALENYAYNMRNTIKDEKISSKLSSEDKTKIDNAIEQAIQWLDTNQLAEADEFEDKMKELEGICNPIIAKMYQGGAGTGGDVDDDAPPAGGSGAGPKIEEVD

>Glyma18g52760 MAKNQGFAVGIDLGTTYSCVAVWQGQQNRVEIIHNDQGNRTTPSFVAFTDDQRLIGDAAKNQAAANPENTVFDAKRLIGRKYSDPTIQNDKMLWPFKVIADNNDKPMITVKYKGHEKLLSAEEVSSMILMKMREIAEAYLETPVKSAVVTVPAYFNDSQRKATIDAGTIAGLNVMRIINEPTAAAIAYGLDKRINCVGERNIFIFDLGGGTFDVSLLTIKDKVFQVKATAGNTHLGGEDFDNRMVNYLVQEFKRMNKVDISGNPRALRRLRTACEKVKRTLSFAVTTTIEVDSLSKGIDFCISITRAKFQELNMDLFEECLKTVNKCLTDAKTDKSSVHDVVLVGGSSRIPKVQELLQEFFEGKDFCKSINPDEAVAYGAAVQAALLSDDIQNVPNLVLLDVAPLSLGILLFQLRGLKEDNQTSARIEVYEGERTRANDNNLLGFFSLLGLVPAPRGHPVDVCFTIDVNGILSVSAEETTTGYRNEITITNDQKRLSAEQIKRMIHEAEKYQVNDMKFMKKANTMNALDHYVYKMRNALNNKNISSKLCLQERKKIKSVITKVTDLLEGDNQRDKIEVFEDHLNELVNLFDRVIGKFA*

>Glyma19g35560 MAGKGEGPAIGIDLGTTYSCVGVWQHDRVEIIANDQGNRTTPSYVGFTDTERLIGDAAKNQVAMNPINTVFDAKRLIGRRFSDSSVQSDIKLWPFKVIAGAADKPMIVVNYKGEEKQFAAEEISSMVLIKMREIAEAYLGSTVKNAVVTVPAYFNDSQRQATKDAGVIAGLNVMRIINEPTAAAIAYGLDKKATSVGEKNVLIFDLGGGTFDVSLLTIEEGIFEVKATAGDTHLGGEDFDNRMVNHFVQEFKRKNKKDISGNPRALRRLRTACERAKRTLSSTAQTTIEIDSLYEGIDFYSTVTRARFEELNMDLFRKCMEPVEKCLRDAKMDKRSVDDVVLVGGSTRIPKVQQLLQDFFNGKELCKSINPDEAVAYGAAVQAAILSGEGNEKVQDLLLLDVTPLSLGLETAGGVMTVLIPRNTTIPTKKEQVFSTYSDNQPGVLIQVFEGERARTKDNNLLGKFELSGIPPAPRGVPQITVCFDIDANGILNVSAEDKTTGQKNKITITNDKGRLSKEDIEKMVQEAEKYKSEDEEHKKKVEAKNALENYAYNMRNTVKDDKIGEKLDPTDKKKIEDAIEQAIQWLDSNQLAEADEFEDKMKELESICNPIIAKMYQGGAGPDMGGAGAGAAEDDYAAPSGGSGAGPKIEEVD

>Glyma19g44140 MKRMIALGFEGSANKIGVGVVTLDGTILSNPRHTYITPPGQGFLPRETAQHHLQHVLPLIKSALETAQITPHDIDCLCYTKGPGMGAPLQVSAIVVRVLSLLWKKPIVAVNHCVAHIEMGRIVTGADDPVVLYVSGGNTQVIAYSEGRYRIFGETIDIAVGNCLDRFARVLTLSNDPSPGYNIEQLAKKGEKFIDLPYVVKGMDVSFSGILSYIEATAAEKLKNNECTPADLCYSLQETLFAMLVEITERAMAHCDTKDVLIVGGVGCNERLQEMMRTMCSERGGRLFATDDRYCIDNGAMIAYTGLLEFAHGASTPLEDSTFTQRFRTDEVKAIWREANLAKLNGLAEKST*

>Glyma20g16070 MIDWNYGRMMTACGQTPQKMASLKVALLALFSVALMFSPSQSAVFSVDLGSESVKVAVVNLKPGQSPICIAINEMSKRKSPALVSFHDGDRLLGEEAAGLAARYPQKVYSQMRDLIAKPYASGQRILNSMYLPFQTKEDSRGGVSFQSENDDAVYSPEELVAMVLGYAANLAEFHAKIPIKDAVIAVPPHMGQAERRGLLAAAQLAGINVLSLINEHSGAALQYGIDKDFSNESRHVIFYDMGASSSYAALVYFSAYKGKEYGKSVSVNQFQVKDVRWNPELGGQHMELRLVEYFADQFNAHVGGGIDVRKFPKAMAKLKKQVKRTKEILSANTAAPISVESLLDDVDFRSTITREKFEELCEDIWEKSLLPVKEVLEHSGLSLEQIYAVELIGGATRVPKLQAKLQEFLGRKELDRHLDADEAIVLGAALHAANLSDGIKLNRKLGMVDGSLYGFVVELNGPDLLKDESSRQILVPRMKKVPSKMFRSVNHNKDFEVSLAYESDNYLPPGVTSPEIAQYQISGLTDASQKYSSRNLSSPIKANIHFSLSRSGILSLDRADAVIEITEWVEVPRKNLTIENSTISSNVSAESAAGNSTEENNESVQTDSGVNKASNISAEEQAATEPATEKKLKRQTFRIVEKITGFGMSLSQDFLAEAKRKLQVLDQKDADRKRTAELKNNLEGYIYTTKEKIETLEEFEKVSTSEERQSFIEKLDQVNPFPWVQDWLYTDGEDANATEFQEHLDQLKAVGDPIFFSGKVFPELVNICFRLKELTTRPAAVEHAHKYIDELKQIVQEWKAKKPWLPQERVDEVIKSSEKLKNWLDEKEAEQKKTSGFSKPAFTSEEVYLKVLDLQTKVASINRIPKPKPKVQKPVKNETESSSAQNTETSDSNSADSSSSSDSSANSSEGTSKETVTEQSEGHDEL
