# Supplementary material for: Genome-wide analysis and expression profiling under heat and drought treatments of HSP70 gene family in soybean (Glycine max L.)
Source: Front Plant Sci. 2015 Sep 25;6:773. doi: 10.3389/fpls.2015.00773 (PMC4585176; doi:10.3389/fpls.2015.00773)
Supplement: Supplementary file 2 [file DataSheet2.DOCX]

>Glyma01g44910 ATGGTGGAACCTGCATATACTGTGACATCTGACAGTGAAACCACTGGTGAAGAAAAATCGTCTACTTTTCCTGAAATAGCAATTGGCATTGATATTGGCACATCACAATGTAGTGTTGCTGTGTGGAATGGCTCCCAAGTGGAGCTTTTGAAGAACACAAGGAATCAAAAGATTATGAAATCATATGTAACCTTCAAAGATAACATCCCTTCTGGTGGAGTTAGCAGTCAACTCTCCCATGAGGACGAGATGTTGTCTGGAGCCACGATTTTCAACATGAAACGCTTGATTGGAAGAGTTGATACTGACCCTGTTGTCCATGCATGTAAGAATCTCCCATTTCTAGTGCAGACTTTGGACATTGGCGTTCGGCCATTTATTGCCGCATTAGTGAACAATATGTGGAGATCCACGACTCCAGAAGAAGTCCTGGCAATATTTCTGGTGGAATTAAGAGCAATGGCTGAAGCTCAGCTGAAACGAAGAATAAGAAATGTGGTTCTTACCGTCCCAGTTTCATTCAGTCGATTTCAGCTAACCCGGATAGAACGTGCTTGTGCCATGGCTGGCCTTCATGTTCTCAGGTTGATGCCTGAACCAACAGCTGTGGCTTTGTTATATGGACAGCAACAACAGCAGACTTCTCATGAGAATATGGGCAGTGGAACTGAGAAAATTGCTCTCATTTTCAGTATGGGTGCTGGTTATTGTGATGTTGCTGTCACTGCTACAGCGGGTGGAGTATCACAGATTAAAGCCTTGGCAGGAAGTACCATTGGTGGTGAAGACTTGCTTCAGAATATGATGCATCATCTGCTACCAAATTCTGAAAATCTATTTAAGAACCATGGGGTCAAAGAAATTAAACAGATGGGCCTGCTTCGAGTTGCAACCCAGGATGCAATTCGCCAGCTTTCCTCTCAGACCATCGTTCAGGTTGATGTAGACCTGGGAGATGGTTTGAAGATATGCAAGGCTGTTAACCGGGAGGAGTTTGAGGAGGTAAACAGAAAGGTGTTTGAGAAATGTGAAAGCCTTATCATACAGTGTTTGCAAGATGCCAAGGTAGAAGTTGAAGAAGTAAATGATGTGATAATTGTAGGCGGATGTTCTTACATCCCGAGGGTGAAAAATCTTGTTACTAACGTATGTAAAGGCAAGGAACTTTATAAAGGCATGAATCCTTTAGAAGCTGCTGTTTGCGGTGCAGCAGTGGAAGGAGCTATTGCTTCAGGCGTCAATGATCCCTTTGGGAACTTGGACTTGTTAACTATCCAAGCTACACCTCTTGCCATTGGGATTCGAGCTGATGGGAACAAGTTTGTCCCTGTAATTCCGAGGGATACTACAATGCCAGCACGGAAGGAGCTAGTTTTCACAACTACTCATGACAATCAAACTGAGGCGTTGATCCTTGTCTATGAAGGAGAGGGTGAAAAGGCAGAAGAAAACCACCTATTGGGATATTTCAAGATAATGGGAATACCTGCTGCTCCTAAAGGAGTTCCAGAAATCAATGTGTGCATGGACATAGACGCTGCAAACGTGCTAAGAGTTTTAGCTGGTGTTGTGATGCCTGGTTCTCGCCAACCTGCGATTCCTGTTATGGAGGTAAGGATGCCAACGGTGGATGATGGGCATGGTTGGTGCGCCGAGGCTCTAAATAGAACCTATGGTGCCACACTGGATTTAGTTACTCTCCAGAAGAAGGCATGA

>Glyma02g09400 ATGGCCAAAAAATATGAGGGATGCGCAGTGGGAATCGACCTTGGTACAACTTACTCGTGTGTTGCAGTGTGGCTGGAGCAGCACTGTCGAGTGGAGATCATCCACAACGACCAAGGCAACAATACCACCCCTTCTTGTGTTGCTTTCACAGACCAACAGAGGTTGATCGGTGAAGCTGCTAAAAATCAGGCTGCCACCAACCCAGAGAACACTGTGTTTGATGCTAAGAGGTTGATTGGTAGGAAATTTAGTGACCCCGTTATTCAAAAAGATAAAATGTTGTGGCCATTCAAGGTTGTTGCTGGTATTAATGACAAACCCATGATTTCCCTTAACTACAAGGGCCAAGAGAAACACCTTTTAGCTGAGGAAGTGTCATCTATGGTCCTCATAAAGATGCGGGAGATTGCAGAGGCATATTTGGAAACACCCGTAGAGAATGCAGTGGTTACTGTGCCTGCTTATTTCAATGACTCTCAACGTAAAGCCACCATAGATGCTGGTGCTATTGCAGGCCTCAATGTTATGCGGATAATCAATGAACCCACTGCTGCAGCTATTGCATATGGCCTTGACAAGAGAACTGATTGTGTTGAAGAGCGAAACATTTTCATCTTTGACCTTGGTGGTGGTACTTTTGATGTATCTCTCCTCACAATTAAGGATAAGGTCTTTCAAGTTAAGGCTACTGCAGGAAACACTCACCTTGGAGGGGAGGACTTTGACAACCGAATGGTGAACTACTTTGTACAGGAATTCAAGAGGAAGAACAAAGTTGACATTAGTGGGAACCCAAGAGCCCTAAGGAGGTTGAGAAGTGCATGCGAGAGGGCAAAAAGGATACTCTCATATGCAGTGACTACCAACATTGAGGTAGATGCTTTATTTCAGGGTGTTGATTTTTGCTCCTCAATCACTCGTGCAAAGTTTGAGGAAATCAATATGGAGCTCTTTGAAGAGTGTATGGAAACAGTTGATAGGTGTCTTTCTGATGCTAACATGGACAAGAGCAGTGTACATGATGTTGTCCTTGTTGGTGGTTCTTCTAGGATTCCCAAAGTGCAGGAGCTATTGCAGGGCTTCTTCGATGGGAAGGTTCTGTGCAAGAGCATCAACCCTGACGAGGCTGTTGCTTATGGTGCAGCTGTGCAGGCTGCTTTGTTGAGTAAAGGCATTGTGAATGTTCCAAACTTGGTCCTATTGGATATTACACCACTGTCTCTTGGTGTATCGGTACAAGGAGATCTCATGAGTGTGGTGATTCCTAGAAATACTACCATTCCTGTAAGGAGGACAAAAACATATGTTACAACTGAAGACAACCAATCTGCTGTCATGATTGAGGTTTATGAGGGCGAGAGAACAAGAGCGAGTGATAACAATTTGCTGGGTTTCTTCACACTTTCTGGCATTCCTCCTGCTCCTCGTGGCCATCCTTTGTATGAAACCTTTGACATAGATGAAAATGGTATTCTATCTGTTTCCGCTGAGGAAGAAAGCACCGGCAATAAGAACGAGATTACCATAACCAATGAGAAAGAAAGACTGTCAACCAAAGAAATTAAAAGAATGATTCAAGAAGCTGAATATTACAAGGCTGAAGATAAGAAATTCCTTAGGAAGGCCAAAGCAATGAATGATTTGGATTATTATGTTTACAAAATCAAGAATGCTTTAAAGAAAAAGGATATCAGCTCAAAGCTTTGCTCAAAAGAAAAGGAGAATGTCAGTTCTGCAATTGCAAGAGCCACAGATTTGCTTGAGGATAATAACCAGCAGGATGATATAGTTGTGTTTGAGGATAATCTGAAAGAGCTTGAGAGCATCATTGAACGCATGAAGGCCATGGGAAAAATTGAGTTTGTGTATATTCACACACACCTGTCCTTAATTTCATCACAATCAAAAGTTACAAGAGATATAGAAATGAGTACTAAGAAGGGTTGTCCAAGCCATGCAGAAGCAATGCAATTGAAGCAACCAGGGGCACCATTCAAATGCAGTGGATGTAAACAAATGGGGTTCGGACCCAGTTACCACTGTGAAAGCAGCAACTGCAGTTACGTCCTCCATGAAGAGTGTGCAAACGCTGTTTCCATAGCCTTCCATCCCTTTTTCTCGAAGAGCAATTTCGAGTTTCATGAGAAAGCACCTGGGAAACGCACAAGGTACTGTGATGGCTGTGGAAAAGATGTGTTAGGGTTTGTGTACCACTGCTCCACCACAGGATATGATCTTCATCCATGCTGCTTGAAGCTGAAACACAACATTTCCGACCAAGAGGGGCGCGTGACGCTTGAACTGTGCCAGAAGGTTCCATCCAAGTGCGTGAAGTGTAAGCATAGGAATGTTGTGGAGAGAGTTAAAGGGTGGTCTTATGTGTCTTCTGGGGGGGATTGTTGTTACCATGTGTCTTGTGTGAAGGAACTGATTCTTGAGAATTGGAAGAAGGGTTATTTCTCTCAAGAAACCAATAATTCAATTGGGATGAGTAGTGACAGGGAGAACACTCAAGTTGCACTGAGAAGCATGGAGATTGTTCCAAGTGGAAGAAGGTCAAGGAGAATCAACAAGTACACCAAGATTGCTGTGTTGGTGTTCAAGCTAGTGGTTTCGGCTATTTTTGGAAACCCTATATCTGCCATTGCTGCTCTTGTGGAAGCCCTTGTTACTGATTGA

>Glyma02g10195 ATGAGATTTATGAGCTACTTCAATGGGAAGGGTCTGTGCATGAGTATCAACCCTAATGAGGCTGTTGCTTATGGCATTAAGAATGTTCCTGATTTGGTTCTGTTGGATGTTATGTCGCTGTCACTTGAAAACCTAACATCTGTTCAGATTAATGTGTATGAGGGCGAGAGAACAAGAGCTAGTGATAACAATTTACTGGGTTTTTTTAGTCTTTCTGGTTTTCCTCCTACTCCTCAGTACCATCCTTTTGATATATGCTTTGATATAGATGTGAATGGTATTTTATCTGTTTCTGCTGAGGAAAAAACCACCGGCTATAAGAATGATATTGCAATAACTAATGATGAAGGAAAATTGTCAGCAGAAGAAATTAAAAGAATGATTGAAAAAGCTGAGACTTACCAGGCTGAGGATAACAAGTTCCTTAGGAAGGCTAACGCAATGAATGCTTTGGATGATTACATTTACAAGATGAAAACGATTTTAAAGAAGGACGATATCAGCTTAAAGCTTTGCTCACAAGAAAGGCAGAAGATCAGTTTTGCAGTTACAAAGGCTACCAATTTGCTCCATGATGATAAACAACAGAATGAAGCAGTGGTGTTTGAGGATTCTCTGAAGGAGCTTGCCATTTGA

>Glyma02g10261 ATGATTGGGGGTTGTTATTTTGCAGATGCCAAGAGGTTGATTGGTAGGAGAGTTAGCGACCCTTCTGTTCATAGTGATATGAAGTTGTGGCCATTTAAGGTTATTGCTGGTGCTGGTGAGAAACCCATGATTGGTGTCAATTACAAGGGTAAGGAAAAGCAATTTTCTACTGAGGAAATCTCCTCTATGGTCCTAACAAAGATGCGGAAGATTGCAGAGGCTTACCTTGGGTCGACTGTGAAGAATGCCTTTGTTACTGTGCCTGCTTACTTCAATGATTCTCAGCGTCAAGCCAGCAAAGATGTTGGTGTCATTACTGGCCTCAATGTTATGAGAATAATAAATGAGCCAACTGTTGTTGCAATTGCATTAGGACTAGACAAGAAAGCCACTAGTGTTGGTGAGAAGAATGTCTTGATCTTTGATCTTGGAGGTGGCACCTTTGATACCACTATTGAGATTGATTCTCTGTTTGAGGGCATTGACTTCTATTCAACCATCACTCGTGCTAGGTTTGAGGAGCTCAACATGAACCTTTTTAGGAAATGTATGGAGCCTGTGGAGAAGTGTCTTAGGGAAGCAAAGATGAGCAAGATCACCGTTCATGATGTTGTCCTCGTTGGTGGCTCTACAAGGATTCCTAAAGTTCAGCAGTTGTTGCAGGACTTCTTCAATGGGAAAGATCTATGCAAGAACATTAACCCCAATGAAGTCGCTGCTTATGGAGTTGCTGTCCAGGCAACTATATTGAGTGGTGAAGGAAATGAGAAGGTTCAAGATTTGTTGCTTTTGGATTTTACCCCATTGTCTTTGGGTTTGGAAACTGCTGGAGATGTCATGACT

>Glyma02g10320 CATTTTTGCCATGTCGAAATCATCGCCAATGACCAGGGTAACAGAACCACGCCCTCTTATGTTGGTTTCACCGATTCAGAGCGTTTGATCGGTGATGCCGCCAAGAATCAGGTCGCCATGAACCCCGTCAACACCGTCTTCGATGCTAAGCGTTTGATTGGAAGGAGAATTTCTGATGCCTCCGTTCAGAGTGATATGAAGCTATGGCCATTTAAGGTTATCCCTGGCCCAGCTGACAAACCTATGATTGTGGTCAACTACAAGGGTGAGGACAAACAGTTCGCTGCTGAGGAAATTTCTTCCATGGTTCTCATGAAGATGCGTGAGATTGCCGAGGCTTATCTGGGTTCCACCGTGAAGAATGCTGTGGTTACTGTTCCTGCTTACTTCAATGACTCCCAGCGTCAGGCCACAAAGGATGCCGGGGTCATTGCTGGTCTCAATGTCATGCGTATCATCAATGAACCCACTGCTGCTGCCATTGCTTATGGTCTTGACAAGAAGGCCACCAGCGTGGGTGAGAAGAACGTGTTGATTTTTGACTTGGGTGGTGGTACCTTTGATGTCTCTCTTCTCACTATTGAAGAGGGTATTTTTGAGGTCAAGGCCACTGCGGGAGATACCCATCTTGGAGGTGAAGATTTTGATAACAGAATGGTTAACCATTTTGTTCAGGAGTTCAAGAGGAAGCACAAGAAGGATATCAGTGGAAACCCTAGGGCTCTTAGGAGGTTGAGGACTGCCTGTGAAAGGGCGAAGAGGACCCTTTCATCTACTGCACAGACTACAATTGAGATTGATTCTCTTTACGAGGGTGTTGACTTCTACACCACAATCACCCGAGCCAGGTTTGAGGAGCTCAACATGGATCTCTTCAGGAAGTGTATGGAGCCTGTTGAGAAGTGTTTGAGGGATGCCAAGATGGACAAGAGCACTGTCCATGATGTTGTCCTTGTTGGTGGATCCACTAGAATTCCTAAAGTCCAGCAATTGTTGCAAGATTTCTTCAATGGAAAGGAACTTTGCAAGAGCATTAACCCTGATGAGGCCGTTGCTTATGGAGCTGCTGTTCAGGCTGCTATTTTGAGTGGTGAAGGTAACGAGAAGGTGCAGGATCTGTTGCTGTTGGATGTTACACCCCTTTCTCTTGGTTTAGAAACTGCCGGTGGTGTCATGACTGTCCTCATTCCCAGGAACACTACTATCCCCACCAAGAAGGAGCAAGTGTTCTCTACTTACTCAGACAACCAACCTGGTGTCTTGATTCAGGTGTACGAGGGAGAGAGAGCTAGAACCAGGGACAACAATTTGTTGGGAAAATTTGAGCTTTCTGGCATTCCCCCAGCACCCAGGGGTGTTCCTCAGATTACTGTTTGCTTCGATATAGATGCCAATGGTATCTTGAATGTCTCTGCCGAGGACAAGACCACAGGGCAGAAGAATAAGATCACCATTACCAACGACAAGGGTAGACTCTCAAAGGAGGAAATCGAGAAGATGGTCCAGGAAGCAGAAAAATACAAGGCTGAGGATGAGGAGCACAAGAAGAAGGTTGATGCAAAGAATGCATTGGAGAACTACGCCTACAACATGAGGAACACCATTAAAGACGAGAAGATTGCATCAAAGCTCTCTGGTGACGACAAGAAGAAGATCGAAGATGCCATTGAGAGTGCTATTCAGTGGTTGGATGGAAACCAGCTGGCAGAGGCTGATGAGTTCGAAGACAAGATGAAGGAGCTTGAGAGCACTTGCAACCCAATCATTGCTAAGATGTACCAGGGTGCAGGTGCTCCCGACATGGCCGGAGGCATGGATGAAGATGTTCCTCCATCTGGTTCCGGTGGTGCTGGCCCCAAGATCGAGGAAGTTGACTAA

>Glyma02g36700 ATGGCGACAAAAGAAGGCAAAGCCATAGGCATAGACCTCGGCACGACCTACAGCTGCGTGGGCGTGTGGCAAAACGACCGCGTTGAGATCATCCCCAACGACCAAGGCAACCGAACCACCCCCTCCTATGTCGCCTTCACCGACACCGAGAGGCTCATCGGAGACGCCGCGAAGAACCAAGTCGCGATGAACCCGCAGAACACCGTCTTCGACGCCAAGCGTTTAATCGGTCGCAGATTCTCAGACTCTCCTGTTCAAAACGACATGAAGCTGTGGCCGTTTAAGGTCGTGGCGGGACCGGGCGACAAGCCCATGATCGTGGTCAATTACAAAGGCGAGGAGAAGAAATTCTCTGCGGAGGAGATATCTTCAATGGTGTTGGTGAAGATGAGAGAAGTAGCAGAGGCGTTTCTCGGACACGCGGTGAAGAACGCGGTTATCACTGTCCCTGCTTACTTCAACGACTCTCAGAGGCAGGCGACGAAGGACGCAGGGGCAATTTCGGGTTTGAATGTGTTGAGGATTATCAATGAACCTACCGCTGCTGCCATCGCGTATGGATTGGATAAGAAGGCTTCGAGAAAAGGTGAACAGAACGTGCTTATTTTCGACCTTGGTGGTGGCACTTTTGATGTTTCAATATTGACCATCGAGGAGGGGATTTTCGAAGTGAAAGCCACTGCTGGTGATACTCATCTTGGAGGTGAAGATTTTGATAACAGAATGGTGAATCACTTTGTTTCTGAATTCAGAAGGAAGAACAAGAAGGACATCAGTGGGAATGCTAGAGCGTTGAGAAGGTTGAGGACAGCGTGTGAGAGAGCAAAGAGAACACTCTCTTCTACCGCGCAGACAACTATTGAAATCGATTCTTTATACGAAGGGATTGATTTCTATGCTACAATCACGAGGGCGAGGTTTGAGGAAATGAACATGGATTTGTTCAGGAAGTGCATGGAGCCGGTGGAGAAGTGTTTGCGTGATGCCAAGATAGACAAGAGTCATGTTCATGAGGTTGTGCTTGTTGGAGGGTCCACTAGGATCCCCAAGGTTCAGCAACTCTTGCAGGATTTCTTCAATGGGAAAGAGCTTTGCAAGAGCATTAACCCCGATGAAGCTGTTGCATACGGTGCTTCAGTTCAAGCTGCCATCTTGAGCGGTGAAGGAGACGAGAAGGTTCAGGATTTGTTGCTGCTGGATGTTACACCTCTCAGTCTCGGGCTCGAAACTGCGGGTGGTGTTATGACTGTACTAATTCCGCGGAACACTACAATTCCCACCAAGAAGGAGCAGATTTTCTCAACCTATTCCGATAACCAACCCGGGGTGTTGATCCAAGTGTTCGAAGGAGAACGAGCTAGAACAAAGGACAACAATCTTCTTGGGAAGTTTGAGCTTACTGGGATCCCTCCAGCACCAAGAGGAGTGCCTCAGATCAATGTCTGCTTCGACATCGACGCTAACGGGATTCTGAATGTCTCCGCAGAGGACAAGACTGCTGGTGTGAAGAACAAGATCACGATCACCAACGACAAGGGTAGGCTGAGCAAGGAGGAGATTGAGAAGATGTTGAAGGATGCAGAGAGGTACAAGGCGGAGGATGAAGAGGTGAAGAAGAAGGTGGAGGCGAAAAACTCGCTTGAGAACTACGCGTATAATATGAGAAACACAATAAAGGATGAGAAGATTGGAGAGAAGTTGAGCCCGGATGAGAAGGAGAAGATTGAGAAGGCTGTGGAGGATGCGATACAGTGGTTGGAGGGGAACCAGTTGGCGGAAGTGGATGAGTTTGAGGACAAGCAGAAGGAGTTGGAAGGGATCTGCAACCCCATCATTGCGAAAATGTACCAGGGTGCTGCTGCTAGACCTGGTGGAGATGTTCCTACGGGTGATGATGACATGCCTGGTGCTGGTGGTGCTGGTTCTGGCGCGGGACCTAAGATTGAAGAAGTTGACTAA

>Glyma03g03250 ATGACCCATAACCATAAACATCCCTACAAGCGGATGAATAAATATATTTCTAGCGTGATTGTCTTTGGTAAAAAGATCGTCCCTTTCCGTAGAAAAACGTTGTCTGCACTCTCCAGTGCTTCATCTTCTCTGACAATGGTACTTTCTCTCTCTACATTAGGGTTTTCTTCAGCACTTTCGCGCCTAAACTTCTTCCCAAAGCCACCACCTTCTCTTTTCTCCGCACTCACCACTTTCCGCAGAAACCAACTTCCACTCAAATCGTCGCTCGCCGCATCGAAACGTGACGGCGATGTTGTCGTTTTGGGCATCGAAACCAGCTGCGACGATACCGCCGCTGCTGTCGTGAGAAGTGACGGTGAAATTCTCAGCCAAGTAGTGTCTTCTCAGGCAGACCTGCTTGCGAAATATGGAGGTGTTGCTCCAAAAATGGCAGAAGAAGCTCACTCAAAAGTTATAGACCAGGTTGTGCAAGAAGCCCTTGATAAAGCTTATCTGACCGAGAAGGATCTTACTGCTGTTGCTGTTACTATTGGTCCTGGTTTGAGTCTCTGCCTTCGTGTGGGGGTGCAGAAAGCCCGGAAAATTGCTGGCGGATTTAATTTACCAATTATTGGCATACATCACATGGAAGCTCATGCTCTGGTTGCCAGGTTAATTGAGAAAGATTTGCAGTTTCCATTCATGGCCCTACTTATTTCAGGGGGACACAATTTACTCGTTCTTGCTCGTGATCTTGGGCAGTACATACAACTTGGAACTACGATAGATGATGCTATCGGTGAGGCATATGACAAGACAGCAAAATGGCTCGGACTGGATTTGAGGAGAAGTGGTGGTCCTGCTATTGAGAAGCTTGCTATGGAGGGTAATGCTGAATCAGTCAAGTTTTCTATTCCAATGAAGCAGCACAAAGACTGCAATTTTTCCTATGCTGGTCTAAAAACCCAAGTACGTCTGGCAATTGAGTCCAAAAAGATTGATGCCAAAATTCCAATTTCTTCAGCAAGTAATGGAGATCGACTGTCACGGGCTGATATTGCTGCTTCTTTTCAGCGAATTGCTGTGTTACATCTTGAGGAGAGGTGTGAACGAGCAATACAGTGGGCATTGAAGATGGAGCCTTCCATAAGACACTTGGTTGTCTCTGGTGGAGTTGCATCAAATCAATATGTCCGAGCCCGGCTTGATATGGTTGTGAAGAAGAATGGCCTGCAACTTGTATGCCCACCTCCTCGGCTCTGTACTGATAATGGTGTAATGATTGCTTGGACTGGTATTGAGCACTTCCGCATGGGAAGATATGACCCTCCTCCTCCTGCAGAAGAACCTGAAGACTTTGTGTATGATATACGCCCAAGGTGGCCGCTGGGGGAAGAATATGCTGAAGGAAAAAGTGTAGCACGTTCGTTAAGAACAGCCCGCATTCATCCTTCTCTTACATCTATAATTCAAGCATCATTGCAACAATGA

>Glyma03g17870 ATGGCCAAAGAAGGACCTAACTGGGACGGGTTGCTGAAATGGAGCATTGCTCACTCCGATGGGACTAGCCCTACTCGCAATTTAAGTGAGGAGGATCGAAAATGGTTTATGGAAGCGATGCAAGCACAGACCATTGATGTTGTAAAACGTATGAAAGAGATCACACTTGTGATGCAAACTCCAGAACAAGTATTGAAAGACCAAGGAGTTACCCCTGCAGACATTGAAGATATGTTGGAAGAGTTGCAAGAGCATGTTGAGTCGATTGACATGGCCAATGATCTCCACTCAATTGGTGGTTTGGTTCCTCTTCTTGGTTACCTAAAGAATTCTCATGCCAATATTCGAGCAATGGCTGCTGATGTTGTGACCACAATAGTCCAGAATAATCCTCGAAGTCAGCAACTTGTTATGGAAGCAAATGGCTTTGAACCTCTTATTTCTAATTTTAGTTCGGATCCTGACGTGACTGTTAGAACTAAAGCACTGGGTGCAATATCTTCACTAATTCGGCACAACAAACCAGGCATTACTGCATTTCGTTTGGCAAATGGGTATGCAGCTTTGAAAGATGCTCTAGCCTCTGAAAATGTGAGATTTCAAAGGAAAGCTCTCAACTTGATCCATTACCTGTTGCATGAGAATAATTCAGACTGCAACATCGTGAACGAGCTTGGGTTTCCTCGAATGTTGATGCACCTTGCCTCAAGTGAAGATTCAGATGTGAGAGAAGCTGCCCTTCGTGGCCTTCTCCAGCTTGCTCACAATGCGAAAGATGGCAAGGATGGCAATGAGAAAGACAGTGTGAAAATAAAGCAACTTCTTCAAGAACGAATAAACAACATCAGTTTAATGTCAGCTGAGGACCTTGGTGTAGTCAGGGAGGAGAGGCAACTTGTGGACTCCCTGTGGAGCACTTGCTTCAACGAGCCGTCTTCTCTTCGAGAGAAAGGTCTTCTAGTGCTTCCGGGGGAGGACGTGCCCCCTCCAGATGTTGCTAGCAAATATTTTGAGCCTCCTCTTAGATCTTCGACTGCAAATCCATCTTCAAAGAAAGACCCAGAAAAGAATGAGATCCCTTTGCTTTTAGGGTCAGGACCTTCTCCTACATACACTAACAACCAAGGTTCAAATAAAGGAGATGCTAGTTCATAG

>Glyma03g32850 ATGGCCGGAAAAGGAGAGGGTCCTGCTATCGGAATCGATCTCGGAACCACCTACTCTTGCGTCGGTGTGTGGCAACATGACCGCGTTGAAATCATCGCCAACGACCAAGGGAACAGAACCACGCCGTCTTACGTCGGATTCACTGACACCGAGCGTCTCATCGGTGATGCGGCCAAGAATCAAGTCGCCATGAACCCCATCAACACCGTCTTCGATGCCAAGAGGTTGATTGGTCGTAGATTCAGTGATTCCTCTGTTCAGAGTGATATCAAATTGTGGCCTTTCAAGGTCATTCCTGGTGCTGCTGACAAGCCAATGATCGTGGTTAACTACAAGGGTGAAGAGAAGCAATTTGCCGCAGAAGAAATCTCTTCCATGGTGCTCATCAAGATGCGTGAGATTGCTGAGGCTTACCTAGGCTCCACAGTGAAGAATGCTGTTGTCACCGTCCCTGCTTACTTCAATGATTCTCAGCGTCAAGCTACCAAAGACGCTGGTGTCATTGCTGGTCTAAATGTGATGCGAATTATCAATGAGCCTACTGCAGCTGCCATTGCATATGGTCTTGATAAGAAGGCCACGAGTGTTGGTGAGAAGAATGTGTTGATTTTTGACCTTGGTGGTGGGACATTTGATGTGTCTTTACTCACCATTGAGGAGGGTATCTTTGAGGTGAAAGCCACTGCTGGTGACACCCATCTTGGAGGTGAAGATTTTGATAACAGGATGGTGAACCACTTTGTTCAAGAGTTCAAGAGAAAGAACAAGAAGGACATTAGTGGAAACCCCAGAGCACTTAGAAGGTTGAGGACTGCTTGTGAGAGGGCCAAGAGAACACTTTCATCGACTGCTCAGACCACCATTGAAATTGATTCTCTATACGAGGGAATTGATTTCTACTCCACTGTTACTCGTGCCAGATTTGAGGAGCTCAACATGGATCTCTTCAGGAAATGTATGGAGCCGGTGGAGAAATGTTTGAGGGATGCTAAGATGGACAAAAGAAGTGTTGATGATGTTGTCCTTGTTGGTGGTTCTACCAGAATTCCCAAGGTTCAACAACTGCTGCAGGACTTCTTTAATGGAAAAGAGCTTTGCAAGAGCATTAATCCCGATGAGGCTGTTGCATATGGTGCGGCTGTTCAGGCTGCAATCTTAAGTGGTGAGGGCAATGAGAAGGTTCAGGATCTTCTCCTCCTGGATGTCACCCCTCTGTCTCTTGGTTTGGAGACTGCCGGTGGTGTGATGACTGTCCTGATCCCTAGGAACACTACAATTCCAACAAAGAAGGAACAAGTTTTCTCAACATACTCTGACAACCAGCCTGGTGTGCTTATCCAGGTCTTTGAGGGTGAAAGAGCAAGGACCAGAGATAACAATTTGTTGGGCAAATTTGAGCTATCTGGCATTCCTCCTGCACCCAGGGGTGTTCCTCAGATTACAGTGTGCTTTGACATTGATGCCAATGGTATCTTGAATGTCTCTGCCGAAGATAAAACCACTGGCCAGAAAAATAAGATCACTATCACCAATGACAAGGGTAGATTGTCAAAGGAAGATATTGAGAAGATGGTTCAAGAGGCTGAGAAGTACAAGTCTGAGGATGAAGAGCACAAGAAGAAGGTTGAGGCCAAAAACGCTTTGGAAAACTATGCATACAACATGAGGAACACCGTGAAGGATGACAAGATTGGTGAGAAACTTGACCCGGCTGACAAGAAGAAGATTGAGGATGCAATTGAGCAAGCAATCCAGTGGTTAGACAGCAACCAGCTTGCAGAAGCAGATGAGTTTGAGGACAAAATGAAGGAATTGGAAAGCATCTGCAATCCAATCATTGCCAAGATGTACCAAGGTGGTGCTGGTCCAGACGTGGGTGGTGCTGGTGCAGCAGAGGATGAGTATGCTGCTCCTCCTTCTGGTGGAAGTGGTGCTGGCCCCAAGATTGAGGAAGTGGACTAA

>Glyma05g03770 ATGGATGCATCCAAACTCAATCAATTGAAGCATTTCATCGAACAGTGCAAGTCCAACCCTTCCCTCCTCTCCGATCCTTCACTCTCCTTCTTCCGCGACTATCTCGAAAGTCTCGGGGCGAAACTCCCTGAATCTGCTTATTCCGAATCGACGGGCGTGGAGAGCGATGAGGACATAGAAGATGTTACGGAGGAGCAAGAGAAGGTAGAAGAAGAAGAAGAAGATGATGAAATAATTGAATCCGATGTTGAGCTCGAGGGTGAAACCTGTCAGTCTGATGATGATCCTCCACAGAAGATGGGAGACCCCTCTGTCGAGGTCACTGAAGAGAATCGCGACGCTTCGCAGATGGCCAAAATTAAAGCCATGGATGCTATTTCTGAAGGTAAGTTGGAGGAGGCGATTGAGAACTTAACAGAAGCTATTTTACTCAATCCTACCTCTGCCATAATGTATGGAACTAGAGCCAGTGTTTACATCAAAATGAAGAAACCCAATGCTGCGATCCGTGATGCTAATGCTGCTTTGGAGATTAATCCTGATTCTGCTAAAGGATACAAGTCACGTGGCGTAGCACGAGCAATGCTTGGTCAATGGGAAGAAGCTGCAAAGGATCTTCATGTGGCTTCAAAGTTAGACTATGATGAGGAAATAAATGCTGTACTTAAAAAGGTGGAACCAAATGCTCACAAGATTGAGGAACACCGTCGGAAGTATGAAAGGCTGCACAAAGAAAGAGAGGATAAAAAAAAGGAGCGTGAGAGGCAGCGGCGCCGTGCTGAAGCTCAGGCTGCCTATGAGAAGGCCAAGAAGCAAGAGCAATCATCTTCCAGTAGAAATCCTGGAGGTATGCCTGGTGGGTTTCCTGGTGGCATGCCTGGGGGCTTCCCAGGGGCCGGGGGCATGCCTGGGGGCTTCCCAGGGGCCGGGGGTATGCCGGGAGGCTTCCCAGGGGCCGGGGGTATGCCTGGGGGAGGCTTCCCAGGAGCTGGTGGCATGCCTGGAGGGGTGCCTGGAAACATTGATTTTAGCAAAATCTTGAGTGACCCTGAACTGATGGCGGCATTTAGTGATCCGGAGGTTATGGCTGCTCTTCAAGATGTTATGAAGAACCCTGCTAATTTTGCCAAGCACCAATCAAATCCAAAGGTAGGTCCTGTAATTGCGAAAATGATGACCAAACTTGGAGGTGGTCCCAAGTGA

>Glyma05g15130 TATAAAAAGGGTATGGAAGATGTTGGATTACACAAGAATCAGATGGATGAGATCGATCTTGTTGGTGGAAGCACAAGGATTCCAAAGGTACGACATCTTTTGAAGGACTACTTTGAAGGAAAAAAGCCAAACAAGGTGCAAAGAAGCATTTTGAGTGAAGAGGGTGGGGAAGAAACCAAAGGTACCTTAGTCTGTAATCTAGCTTTTTTTATTACTTATTACTATGTTGTTCGCTTTCTAATTGTTGTGTGCTCTGGATCCAGATATCCTTCTCCTGGATGTGGCTCCCCTCCCTTTTTATTGAGTTTCAGAATCTTCTTTTGTTTTGTTGTGTCGGGTTTTCATTGTAAGGGATGGCCACTTGAATTTGCTACCCCAGCTACAATGTCTGCTTCTACAGTTGATTGTGCTACAACT

>Glyma05g36600 ATGCTCGTACTTTCGAGTGAGCACTATAAATGGCGAAGTCCCCGTCGTAAACTCAATAAAACTGCAATTCGTCCATCAAGAAAGGAAAGAAAAGGTGACATGATAATGGCTCGCTCGTTTTCACGCGGGTCTCTGCTTCCTCTCGCCATCGTTTCCTTAGTATGTCTATTTGTGATTTCCATTGCAAAGGAGGAAGCCACCAAGTTGGGGACGGTCATTGGGATTGATCTTGGAACAACCTATTCATGTGTTGGTGTTTACAAGAACGGCCATGTTGAAATCATAGCCAATGATCAAGGTAACCGTATCACCCCATCGTGGGTTGCTTTCACCGACAGTGAGAGACTAATTGGAGAGGCTGCCAAGAATCTGGCAGCTGTCAATCCAGAAAGGACCATCTTTGATGTCAAGAGACTTATTGGAAGAAAGTTTGAAGATAAGGAAGTTCAAAGAGATATGAAGCTTGTTCCTTATAAGATTGTTAACAAGGATGGAAAACCTTACATACAGGTGAAAATTAAGGATGGTGAGACCAAGGTGTTCAGCCCTGAGGAAATCAGTGCCATGATTCTGACTAAGATGAAGGAAACTGCGGAAGCATTCCTTGGGAAGAAAATTAATGATGCTGTGGTCACTGTCCCAGCTTACTTCAATGATGCTCAGAGGCAGGCCACCAAGGATGCTGGTGTCATTGCTGGTCTCAATGTTGCTAGAATTATTAATGAACCTACTGCTGCTGCCATTGCGTATGGATTGGACAAGAAAGGTGGCGAGAAGAACATTCTTGTTTTTGACCTTGGGGGTGGAACATTTGATGTTAGTATCTTGACAATTGATAATGGTGTTTTTGAGGTTCTTGCTACAAATGGAGATACTCATCTTGGAGGTGAGGACTTTGATCAGAGAATAATGGAGTACTTCATTAAATTGATCAAGAAAAAGCATGGAAAGGATATTAGCAAGGACAGTAGAGCACTTGGCAAGCTGAGGAGAGAGGCTGAGCGTGCAAAGAGAGCTCTCAGCAGCCAGCACCAGGTCCGTGTGGAAATTGAATCACTTTTTGATGGTGTTGATTTTTCTGAGCCACTCACCCGAGCTCGGTTTGAGGAGTTGAACAATGACTTGTTCCGGAAGACCATGGGTCCAGTGAAGAAGGCTATGGAAGATGCTGGATTACAGAAGAGTCAGATTGATGAGATTGTTCTTGTTGGTGGAAGCACAAGGATTCCAAAGGTACAACAACTTTTGAAGGACTACTTTGATGGAAAGGAGCCAAACAAGGGTGTCAACCCTGATGAAGCAGTTGCCTATGGTGCTGCAGTGCAAGGAAGCATTTTGAGTGGAGAGGGTGGTGAAGAAACCAAAGATATCCTTCTCTTGGATGTGGCTCCCCTCACCCTTGGAATTGAAACTGTTGGTGGGGTGATGACGAAGTTGATTCCCAGAAACACTGTTATCCCTACCAAGAAATCTCAGGTGTTCACCACCTACCAGGATCAGCAGACTACCGTCTCCATTCAGGTTTTTGAAGGTGAGAGGAGTCTCACAAAGGATTGCCGTCTGCTTGGGAAATTTGAACTGTCTGGAATTCCTCCAGCTCCAAGGGGTACCCCTCAAATTGAAGTGACCTTCGAAGTTGATGCCAACGGCATTCTAAATGTGAAGGCAGAAGACAAGGGCACTGGTAAATCAGAAAAGATCACCATTACAAACGAAAAGGGACGTCTTAGCCAGGAAGAAATTGAGAGAATGGTTCGTGAAGCAGAGGAGTTTGCCGAGGAAGACAAGAAGGTGAAGGAGAGGATTGATGCTCGCAACAGTCTTGAAACCTATGTCTACAACATGAAAAACCAGATCGGTGACAAGGACAAGCTCGCTGACAAGTTGGAGTCTGATGAAAAGGAGAAAATTGAGACTGCAGTAAAAGAAGCATTGGAATGGCTGGATGACAACCAGAGTGTGGAGAAAGAAGAATATGAGGAGAAGCTCAAAGAGGTGGAAGCTGTTTGCAACCCAATCATCAGTGCTGTATATCAGAGATCAGGTGGAGCCCCAGGTGGTGGTGCATCAGGCGAGGAGGATGATGATTCTCATGACGAGCTCTAG

>Glyma05g36620 ATGGCTGGCTCGTGGGCACGCCGTTCTCTGATTGTTCTGGCTATCATTTCCTTCGGATGTTTATTTGCAATTTCCATTGCTAAGGAGGAAGCCACAAAATTGGGGACGGTCATCGGGATTGATCTTGGAACGACCTATTCATGTGTTGGTGTTTACAAGAACGGCCATGTTGAAATCATAGCCAATGATCAAGGTAACCGTATCACCCCATCGTGGGTTGCTTTCACCGACAGTGAGAGACTAATTGGAGAGGCTGCCAAGAATCTGGCAGCTGTCAACCCAGAAAGGACCATCTTTGATGTCAAGAGACTTATTGGAAGAAAGTTCGAAGATAAGGAAGTTCAAAGAGATATGAAGCTTGTTCCTTATAAGATTGTCAACAAGGATGGAAAACCTTACATACAAGTGAAAATTAAGGATGGTGAGACCAAGGTGTTCAGCCCTGAGGAAATCAGTGCCATGATTCTGACTAAGATGAAGGAAACTGCGGAAGCATTCCTTGGAAAGAAAATTAATGATGCCGTGGTCACTGTCCCAGCTTACTTCAATGATGCTCAGAGGCAGGCCACCAAGGATGCTGGTGTCATTGCTGGTCTCAATGTTGCTAGAATTATCAATGAACCCACTGCTGCTGCCATTGCCTATGGATTGGACAAGAAAGGTGGCGAGAAGAACATTCTAGTCTTTGATCTTGGTGGTGGGACCTTTGATGTCAGTATCTTGACAATTGATAATGGTGTTTTTGAAGTTCTTGCCACAAATGGAGATACTCATCTTGGAGGTGAGGACTTTGATCAGAGAATAATGGAGTACTTCATTAAATTGATCAAGAAAAAGCATGGAAAGGATATTAGCAAGGACAACAGAGCACTTGGCAAGCTGAGGAGAGAGGCTGAGCGTGCAAAGAGGGCTCTCAGCAGCCAGCACCAGGTCCGCGTGGAAATTGAATCACTTTTTGATGGTGTTGATTTTTCTGAGCCACTCACCCGAGCTCGTTTTGAGGAGTTGAACAATGATTTGTTCCGGAAGACCATGGGGCCAGTGAAGAAGGCTATGGAAGATGCAGGATTACAGAAGAGTCAGATTGATGAGATTGTTCTTGTTGGTGGAAGCACAAGGATTCCAAAGGTACAACAGCTTTTGAAGGACTACTTTGATGGAAAGGAGCCAAACAAGGGTGTCAACCCTGATGAAGCAGTTGCCTATGGTGCTGCAGTGCAAGGAAGCATTTTGAGTGGAGAGGGTGGTGAAGAAACCAAAGACATCCTTCTCCTGGATGTGGCTCCCCTCACTCTCGGAATTGAAACTGTTGGTGGAGTCATGACAAAGTTGATTCCCAGAAACACTGTTATCCCAACCAAGAAATCTCAGGTGTTCACCACCTATCAGGACCAGCAGACTACAGTCTCCATTCAGGTTTTCGAAGGCGAGAGGAGTCTCACAAAGGATTGCCGCCTTCTTGGGAAATTTGATCTGTCTGGAATTCCTCCAGCCCCAAGGGGTACGCCTCAAATTGAAGTGACCTTCGAAGTTGATGCCAACGGCATTCTAAATGTGAAGGCAGAAGACAAGGGCACTGGTAAATCAGAAAAGATCACCATTACAAACGAAAAGGGACGTCTTAGCCAGGAAGAAATTGAGAGAATGGTTCGCGAAGCAGAGGAGTTTGCAGAGGAAGACAAGAAGGTGAAGGAGAGGATCGATGCTCGCAACAGTCTTGAAACCTATGTATACAACATGAAGAACCAGATCAGTGACAAGGACAAGCTCGCTGACAAGTTGGAGTCTGATGAAAAGGAGAAAATTGAGACTGCTGTGAAAGAAGCACTGGAATGGCTGGATGACAACCAGAGTATGGAGAAAGAAGATTATGAAGAGAAGCTGAAGGAGGTTGAAGCCGTTTGCAACCCAATCATTAGTGCTGTGTATCAGAGATCTGGAGGAGCCCCAGGCGGTGGTGGTGCATCAGGCGAAGAAGACGAGGACGATTCTCACGATGAGCTCTAG

>Glyma06g00310 GAAGATTCCAGAGGCGGCGTGAGTTTCGTCGCGGACGCCGTATACTCGCCCGAGGAACTGGTGGCCATGATGTTGGGTCACACGGCGAGTTTGGCGGAGTTTCACGCGAAGGTTCCTATAAAGGACGCAGTGATTGCGGTGCCACCGAATCTGGGGCAAGCCGAGCGGAGAGGATTGCTTGTGGCGGTGCAGTTTGCGGGGATTAACTCGAGGCACGTGATCTTCTATGACATGGGTTCCAGCAGTACCTATGCAGCGGTTGTGTATTTCTCGTCGTGTGGGAAGGTGAACCCGGAGCTTGGGGGTCAGCATATGGAATTGCGATTGGTGGAGTATTTTGCTGATGAGTTCAATGCACAAGTTGGTGGTGGAATAGATGTCAGGCATTTTCCCAAGGCCATGGCTACATTGAAGAAACAGGTTAAACGAAGAAAAGAAATGCTTAGTGCAAACACAGTTGCTCCTATTTCAGTTGAATCGCTTGATGATGGCGTCGACTTCGGGAGCACAATGAACCGTGAGAAATTTGAAGACCTCTGTCAAGACATTTGGGATAAATCTCTTTTGCCTGTGAAAGAGGTGCTTCAGCATTCTGGCCTGTCATTGGACCTAATATATGCACTGCAGTTGATTGGAGGTGCTACCAGAGTGCCAAAATTACAGGCTCAGCTTCAACAATTCCTTGGGAGAAAACAACTTGACAGGCATCTTGATGCTGATGAAGCAATAGTTCTTGGCTCAGCTCCGCACGCTGCAAATTTAAGTGACGGAATCAAATTGAAAAGCAAACTAGGAATACTTGATGCTTCCATGTATGGTTTTGTGGTTGAGTTGAGTGCTCCTGATCTTTCCAAAGATGAAAGCTCTAGGCAGTTACTTGTACCGCAAATGAAGAAAGTCCCCAGTAAAGATCCTGAGCATCATTTGCCTCCTGGTGTTACCTCTCCTGAAATTGCTCAATACCAGATATCTGGTTTGACAGATGCCAGTGAGAAATACTCATCTCGGAATCTTAGGAGTGGGATTCTTTCTCTGGATCGGGCAGATGCCATTATTGAAATAACAGAGAGGGTGGAAGTTCCAAGGAAGAATATGACCATAGAGAATTCAACCATTTCATCAAATGTTTCGGCTGAATCTGCTGGTAGTAATAGTTCTGAGGAAAACATGCAAACTGATAGTGAGATTAGTAAGACATCCAACGGTAGTGCAGAGGAGCAAGCTACTGCTGCTGAGCCCGCTACAGAGGAAAAGCTGAAAAAGCGAACCTTTAGGGTACCATTAAATATTGTTGAGAAGATAACTGGACCTGGAATGCCTCTATCTCAAGATTTTCTTGCTGAAGCCAAAAGAAAATTACTAGCACTAGATGAAAAAGACGCAGACAGAAAAAGAACAACTGATGAGGAACGCCAGTCCTTCATTGAGAAGCTTGATCAGGTTCAAGATTGGTTGTATAGAGATGGTGAAGATGCCAATGCCACAGAGTTTCAAGAGCTTCTAGATCAGTTAAAAACTGTTGGAAATCCGATTTTCTTCAGGTTGAAAGAGCTGACAGCTCGACCAGCAGCAGTTGAGCATGCTCATAGATACATTGATGAGTTGAAAGAGTGGAAAGCAAACAAGAAGCCCGCATTTATCTCTGAAGCAGTATATTCAAAGGTGCTTGATCTGCAAAACAAGGTTTCCAGTATTAATAGAATTCCCAAGCAAAATACATGA

>Glyma07g00820 ATGAGCGTGGTTGGTTTCGATTTCGGTAACGAGAGTTGCGTTGTTGCGGTTGCGAGGCAGAGAGGGATTGACGTTGTGCTCAATGATGAGTCCAAGCGTGAAACGCCCGCCATTGTGTGCTTCGGTGACAAGCAACGCTTCATTGGCACTGCCGGTGCTGCCTCCACTATGATGAACCCTAAGAATTCAATCTCACAGATTAAGAGACTCATTGGTAGGAAATTCGCTGATCCCGAATTGCAGCGGGATCTTAAGTCATTGCCGTTTCTCGTCACTGAGGGGAGTGATGGGTACCCGTTGATTCATGCGCGATACATGGGTGAGGCCAAGACATTTACGCCTACCCAAGTGTTTGGAATGATGCTGTCGAATCTTAAGGAAATTGCGGAGAAGAATCTCACTACGGCAGTTGTTGATTGTTGCATTGGAATCCCGGTTTATTTCACTGATCTGCAGAGAAGGGCGGTGTTGGATGCGGCCACAATTGCTGGTCTGCACCCGCTTCGGTTGATTCACGAAATGACTGCCACTGCCTTGGCCTATGGGATTTATAAAACGGACCTTCCGGAAAATGATCAGCTGAATGTTGCGTTTGTTGATGTTGGACATGCTAGCTTGCAAGTATGCATTGCTGGATTCAAGAAGGGGCAGCTGAAAGTGTTGGCTCATTCGTATGATAGGTCTTTCGGCGGTAGGGATTTTGATGAGGTTTTGTTCCATCACTTTGCTGAGAAGTTTAAGGACGAGTACAAGATTGATGTTTTTCAAAATGCCAGGGCTTGCATAAGGCTCAGGGCTGCCTGTGAGAAGATCAAGAAGATGCTTAGTGCAAATCCTGAGGCACCTCTCAACATTGAGTGCTTGATGGATGAGAAGGATGTCCGGGGCTTCATCAAGCGAGATGAATTTGAGCAACTAAGTCTTCCAATTTTGGAACGTGTGAAGGGGCCTCTGGAGAAGGCACTTGCTGAAGCAGGTCTTACCGTCGAAAATGTGCACACGGTTGAGGTGGTTGGTTCAGGTTCTCGTGTGCCGGCCATTAACAAAATATTGACAGAGTTTTTCAAAAAGGAGCCTAGGCGGACAATGAATGCTAGTGAGTGTGTTGCTAGGGGATGTGCGTTGGAATGTGCAATTCTTAGTCCAACGTTCAAAGTACGAGAATTTCAGGTCAACGAAAGCCTTCCTTTCTCGATTTCTCTTTCATGGAAAAGTTCTGGTCCAGATGCACAGGACAATGGACCAGAAAATCAGCAGAGTTCCCTTGTTTTTCCCAAGGGTAATCCCATACCAAGTATCAAGGCACTGACATTCTACAGGTCAGGAACATTCTCTGTTGATGTACAATTTGGTGATGTGAGTGGGCTGCAAACACCTGCTAAGATCAGCACCTATACTATTGGTCCTTTCCAAACTACAAATGGTGAAAAGGCAAAAGTTAAAGTGAAAGTTCGTCTGAATCTGCATGGAATTGTATCCCTTGAGTCTGCAACGCTCCTGGAAGAGGAAGAAGTTGATGTTCCAGTTAGCAAAGAAGCAGCAGGGGAAAATACTAAGATGGACATCGATGAAGTCCCAGCTGAGGCCGCTGCACCTCCTTCCTCCAATGACACTGGTGCTAATATGGAAAATGGAAAGGCTAGTATTGATGCCTCTGGGGTTGAAGATGGCATCCCTGAGAGTGGAGGTAAGCCTTTGCAAACAGATACTGATACCAAGGTTCAGGCTCCAAAGAAAAAGGTTAAGAAAACAAACATTCCTGTAGTAGAGTTAATTTATGGAGCAATGGTGCCTGTGGATGTCCAGAAAGCACTAGAGAAGGAGTTTGAAATGGCTTTGCAAGATCGTGTGATGGAAGAAACAAAAGACAAGAAAAACGCAGTTGAGGCTTATGTTTATGACATGAGAAACAAGCTTAATGACAAATACCAAGAGTTTGTCACTGCTTCAGAGAGAGATGATTTTACTGCTAAACTTCAGGAAGTGGAAGATTGGCTTTATGGTGAGGGTGAAGATGAAACTAAAGGTGTATATACTGCCAAGCTTGAGGAACTCAAAAAGCATGGTGATCCAATTGATGAGCGTTACAAAGAATTCATGGAGAGGGGTACTATAATCGAACAGTTTGTCTATTGTATAAATAGTTACAGACAAGTTGCAATGTCGAATGATCCCAGATTTGAGCACATTGACATTAACGAGAAACAGAAGGTCATAAATGAATGTGTTGAAGCTGAAAAGTGGTTTAATGAGAAGCAGCAGCAGCAGAACTCACTTCCAAAATATGCCAACCCTGTACTCTTGTCAGCTGAAATAAGAAAGAAAGCTGAAGCTGTCGATAGGTTCTGCAAGCCGATTATGGCAACACCAAGGCCAACCAAGGCGACTACTCCACCAGGACCAGCAACACATCCATCTTCTCAGAGTGATGAACAGCAGCAGCAGCAGCAACCTCCTCAGGGGGATGCTGATGCCAACAGTAATGAGAATGGTGGGAATAGCAGTAGTCAGGCTGCACCAGCGTCTACTGAACCAATGGAAACTGATAAGTCTGAGAAAACAGCCTCTGCCTAA

>Glyma07g02450 CCTACCGCTGCTGCCATTGCCTATGGATTAGACAAGAAGGCTTCCAGGAGTGGCGAAAAGAATGTGGTTATCTTCGACCTCGGTGGTGGAACTTTTGATGTCTCCCTCCTCACCATCCAGGAAGCTATTTTCCAAGTGAAGGCCACTGCTGGGGACACTCATTTGGGAGTTGAATCAAACTATATTCTTTCTTCTTCTAATGTTTCTTTTGCATACCAGCTTGTGAACCACTTCGTGTCTGAGTTCAAGAGGAAACACAAGAAGGATGTCAGCACTAATGCCAGAGCACTTAGGAGATTGAGGACTGCATGCGAGAGAGGGCTAAGAGGACTCTCTCTTCCACCACTCAAACTACCATCGAGATTGACTCTCTCTACGAAGGTATTGACTTCTATTCCACCATCACCAGAGCCAGGTTTGAGGAGCTCAACATGGACACGTTCACGATGTTGTCCTTGTTGGTGGATCCACCAGGATTCCAAAAGTGCAACAACTAGCATTAACCCGGATGAGGCTGTAGCCTATGGGGCTGCTGTGCAGGCTGCGATTTTGAGCGGTGAAGGGAACGAGAAGGTTCAGGACTTGCTTCTGCTTGATGTCACTCCTCTTAGCCTCGGCATTGAGACTGCAGGAGGTGTGATGACAGTGTTGATTCCAAGGAACACAACCATTCCCACCAAGAAGGAACAGATTTTCTCAACTTATGCTGATAATCAGCCAGGAGTGCTAATTCAGGTGTATGAAGGCGAGAGGGCAAGCACCAAAGACAACAATCTTTTAGGGAAGTTTGAACTCACAGGCATCCCTTCAGCACCAAGAGGAGTTCCACAGATCAATGTGTGCTTTGACATTGACGCCAATGATGGTCCAGGAGGCGGAGAGGTGGACGCCAAGAACTCCTTGGAGAACTTGGCGTACAACATGAGGAATACTGTGAAGGATGACAAGTTTGCAGGCAAAATGAACCCTTCTGATAAGGAGAAGATAGAGAAGGCGGTTGATGAGACCATTGAGTGGCTAGATAGGAACCTACTGACTGAGGTTGAGGAATTTCAGGACAAATTGAAGGAGTTGGAGGGACTTTGCAACCCAATTATCTCAAACATGTACCAAGGAAGTGGTGCAGATGACATTCCTAATGGTGCTGGCTATGGAAAATCATCCACTGGTGGTGCTGGGCCTAAGATTGAAGAA

>Glyma07g26550 ATGATTTGTGACTTCTGTTTTCTGAAGGAAAATCAGATCATGGCCAGAGAATACGAGGGATGTGCAGTGGGAATTGACCTTGGCACAACTTACTCGTGTGTTGCAGTGTGGCTGGAGCAGCACTGTCGAGTGGAGATCATCCACAATGACCAAGGCAACAATACCACCCCTTCTTGTGTTGCTTTCACAGACCATCAAAGGTTGATTGGTGAAGCTGCTAAAAACCAGGCTGCTACCAACCCAGAGAACACTGTGTTTGATGCTAAGAGGTTGATTGGTAGGAAATTTAGTGACCCTGTTATTCAAAAAGATAAAATGTTGTGGCCGTTCAAGATTGTTGCTGGTATTAATGACAAACCCATGATTTCCCTTAATTACAAGGGCCAGGAGAAACACCTTTTAGCCGAGGAAGTATCATCTATGGTCCTCACAAAGATGCGGGAGATTGCAGAGGCATATTTGGAAACACCTGTAAAAAATGCAGTGGTTACTGTGCCTGCTTATTTCAATGACTCACAACGTAAAGCCACCATAGATGCTGGTTCTATTGCAGGCCTGAATGTTATGCGGATAATCAATGAACCTACTGCTGCAGCTATTGCATATGGGCTTGACAAGAGAACTAATTGTGTTGGAGAGCGAAGCATCTTCATCTTTGACCTTGGTGGTGGTACTTTTGACGTGTCTCTCCTCATAATTAAGGATAAGGTCTTCCGAGTTAAGGCCACTGCAGGAAACACTCACCTTGGAGGGGAGGACTTTGACAACAGAATGGTGAACTACTTTGTACAAGAGTTCAAGAGGAAGAACAAAGTTGACATTAGTGGGAATGCAAGAGCCCTAAGGAGGTTGAGAAGTGCATGCGAGAGGGCAAAAAGGATACTCTCGTATGCAGTGACTACCAACATTGAGGTAGATGCTTTATTCCAGGGCATTGACTTTTGCTCCTCAATCACCCGTGCAAAATTTGAGGAAATCAATATGGAGCTCTTTGAAGAGTGTATGGAAACAGTAGATAGGTGTCTTTCTGATGCCAACATGGACAAGAGCAGTGTACATGATGTTGTCCTTGTTGGTGGTTCTTCTAGGATTCCAAAAGTGCAGGAGCTATTGCAGGACTTCTTCAATGGGAAGATTCTGTGCAAGAGCATCAACCCTGACGAGGCTGTTGCTTATGGTGCAGCTGTGCAGGCTGCTTTGTTGAGTAAAGGCATTGTGAATGTTCCAGACTTGGTCCTGTTGGATATTACACCTCTGTCTCTTGGTATATCGCTAAAAGGAGATCTCATGAGTGTGGTGATTCCTAGAAATACTACCATTCCTGTAAAGACGACAGAAACATACTCTACAGCTGTAGATAACCAATCTGCTGTCCTGATTGAGGTTTATGAGGGTGAGAGAACAAGAGCCAGTGATAACAATTTGCTGGGTTTTTTTAGGCTTTCTGGCATTCCTCCTGTTCCTCGTAACCATCTTGTGTATATTTGCTTTGCCATAGATGAAAATGGTATTCTATCTGTTTCTGCCGAGGAAAAAAGCACTGGCAATAAGAATGAGATTACCATAACCAATGACAAAGAAAGGTTATCAACCAAAGAAATTAAAAGAATGATTCAAGAAGCTGAGTATTACCAGGCTGAAGATAAGAAATTCCTTAGGAAGGCCAAAGCAATGAATGATTTGGATTGTTACGTTTACAAGATCAAGAATGCTTTAAAGCAAAAGGATATCAGCTCAAAGCTTTGCTCAAAAGAAAAGGAGGATGTTAGTTCTGCAATTACAAGAGCTACAGATTTGCTTGAAGGTAATAACCAGCAGGATGACATAGCTGTGTTTGAGGATAATCTGAAAGAGCTTGAGAGCATCATTGAACGCATGAAGGCCATGGGCAAAATTGTTTAG

>Glyma07g30290 ATGGCCGCCGCCACTGCATTGCTCCGCTCTCTCCGCCGCCGCGACCTTCCCTCTTCTTCTCTCTCCGCCTTCCGATCGTTGACGAGTGGCACAAAGACATCATATGTAGGTAACAAGTGGGCAAGTTTGTCACGACCGTTCAGTTCAAAGCCTGCTGGTAATGATGTCATTGGAATTGATCTGGGTACTACCAATTCGTGTGTCTCTGTTATGGAGGGAAAGAATCCTAAAGTTATTGAGAATTCTGAAGGAGCTCGTACAACACCATCAGTGGTTGCCTTCAACCAGAAAGCGGAGCTTCTTGTTGGTACACCAGCCAAGCGTCAGGCTGTGACTAACCCAACAAACACTCTTTTCGGAACCAAGCGTTTGATCGGTAGGCGTTTTGATGATTCTCAAACTCAGAAGGAGATGAAGATGGTTCCATACAAGATTGTTAAGGCGTCCAATGGAGATGCTTGGGTTGAAGCCAATGGGCAGCAGTATTCTCCCAGCCAAGTTGGTGCTTTTGTTCTCACCAAGATGAAGGAAACTGCTGAATCATATCTTGGAAAGTCAGTTTCAAAAGCTGTAATTACTGTACCAGCTTACTTCAATGATGCTCAGAGGCAGGCAACAAAAGATGCCGGTAGAATTGCTGGTCTTGATGTTCAGAGAATCATCAATGAGCCCACTGCCGCTGCACTTTCCTATGGGATGAACAACAAGGAGGGTCTCATTGCAGTTTTTGATCTTGGAGGTGGAACATTTGATGTGTCCATCTTAGAGATTTCTAATGGTGTTTTTGAGGTGAAAGCAACAAATGGTGACACTTTCTTGGGAGGAGAGGATTTTGATAATGCTTTATTGGATTTTCTAGTGAACGAATTCAAAAGAACTGAGAGTATTGATCTTTCAAAGGATAAGCTTGCATTGCAAAGGCTTCGGGAAGCTGCTGAGAAAGCCAAAATAGAACTGTCTTCAACATCTCAAACAGAAATTAATCTTCCTTTCATCACTGCTGATGCATCTGGTGCAAAGCATCTGAACATCACATTGACTAGATCTAAGTTTGAGGCTTTGGTAAACCACTTGATTGAAAGGACAAAGGCACCGTGTAAGAGCTGTTTGAAGGATGCTAACATATCTATCAAGGAAGTTGATGAGGTTCTTCTTGTTGGAGGAATGACTCGTGTGCCTAAAGTCCAGGAGGTGGTTTCAGCGATCTTTGGAAAGAGTCCTAGCAAAGGAGTAAATCCTGATGAGGCAGTTGCCATGGGAGCAGCTATTCAGGGTGGTATCCTACGTGGAGATGTTAAAGAGCTACTACTCCTAGATGTCACTCCACTTTCTCTGGGTATTGAGACTTTGGGTGGTATCTTTACCAGGTTGATTAACCGCAACACCACAATTCCTACAAAAAAGAGTCAGGTGTTTTCAACAGCAGCTGACAATCAGACTCAGGTTGGTATCAAGGTGCTACAAGGTGAGAGGGAAATGGCTGTAGACAACAAATCGCTTGGAGAATTTGAGCTTGTTGGCATTCCTCCTGCCCCAAGAGGCATGCCTCAGATTGAAGTCACATTTGACATAGATGCCAATGGGATTGTTACTGTCTCTGCCAAAGACAAGTCTACTGGGAAAGAACAACAAATCACCATCCGTTCATCTGGAGGACTCTCAGAAGATGAGATTGATAAGATGGTCAAAGAAGCAGAGTTGCACGCTCAGAAAGACCAGGAAAGAAAGGCTCTCATTGACATCAGAAACAGTGCAGATACAAGCATCTACAGCATTGAGAAGAGTTTAGGTGAGTACAGAGATAAGATCCCCAGTGAAGTGGCCAAAGAGATTGAAGATGCAGTATCGGATTTGAGAACAGCAATGGCAGGAGACAATGCTGACGAAATTAAGGCAAAGCTTGATGCTGCAAACAAAGCTGTCTCCAAGATTGGAGAGCACATTTCAGGTGGTTCTAGTGGCGGTTCCTCAGCCGGAGGTTCTCAGGGTGGTGAACAGGCTCCCGAGGCCGAGTATGAGGAAGTCAAGAAATGA

>Glyma07g32921 ATGCCCGCTCACAACAGTCACGAGACCACCATGGACTGCCTTGAAGAGGCACTGTCCCACCTCACCCAGAACATTTCCACCATGACCGCCAAGTCTCTGGAGATGGCCGCTAAGCTTGATGTCATCCTTGACTGGTTATCTGCTCTCCAACCAACTCCATCTTCTCCCAAATCACTCGCTCCACCGGACGCACCCATGCCGAATCTGCCCCCCATGGTCGGAATCAATTCCGGCACCACCTACTCTTGCGTCAAGGTGTGGCAGCACCACCATGTCGAAATCATCGCCACTGATCAGCGTAATAGAAGCTCGGCATCTTACTTTGCTTTCACCGAACGTTTGATCGGTGACGCCGCCAAGTACTACGTCACCATCAACCCCATCAACACCATCTTCAATGCTAAGCGTTTGTGTGGAAGGAGATTTTCCGCTGCCTCCATTCGGAGTGATAGGCAGTTATGGCTATATAAGGTTTTCCCTGGTCCTCCTGACAAACCTATGATTGTGGTCAATTACAAGGGTGAGGACAAGAAATTCGCTGCTGAGGAAATGTCTTCCATGCTTCCTTTCATTGATGTTATTGGCGATCAATCTTCAAGTAAATCCAGTGTTCTTGAATCTTTGGCTGCTATTAAACTACCACGTGGCCAAGGTACTTGCACTAGGGTGCCTTTGGACATGAGGCTCCGAAATCACCCTTTTACAACCCTAGAGCTTGTCTTGGAGTTCTATGGCCAGACCATTTCAATAGATGAAGCACACATTTCTCAGGCCATAAGTGCTGCCACTGCTGCCACAGAAGAGCTTGCTTGCCATGGCAAAGGGATTTCCAACAACCCTTTGACATTGTTGGAGAAGAAGAATGGTGTTCCTGATCTTTATCCGGTTGATCTTCCTTGTATAATTCAGGTTCCAGTTCATGGACAACCTAAGAATATTTATGATCAGATTAAGGATATGATCATGGAGTATATAAAGCCGGAAGCAAGTATTTTATTGACTGTTCTCTCTGCTAGTGTTGATTTTACTACTTGTGAGTCCATCGGGATGTCTCAAAGTGTTGAGAAAACTGAGCTCAGGACGTTGGCTGTGGTGACAAAGACTGACAAGTCCCCTGAAAGCTTGTTAAAGAGACACTCAGGCTGGTTCAATTGCTGCCAGTGGCCGATATGA

>Glyma08g02940 ATGGCTGGCTCGTGGGCACGCCGTTCTCTGATTGTTCTAGCCATCATTTCCTTCGGATGTCTATTTGCGATTTCCATTGCTAAGGAGGAAGCCACCAAGTTAGGGACGGTCATCGGCATTGATCTTGGAACAACCTATTCATGTGTCGGTGTTTACAAGAATGGCCATGTTGAAATCATAGCCAACGACCAAGGTAACCGTATCACCCCATCGTGGGTTGCTTTCACCGACAGTGAGAGACTCATTGGGGAGGCTGCCAAGAATCAGGCAGCTGTCAACCCAGAAAGGACCATCTTTGATGTCAAGAGACTTATCGGAAGAAAGTTCGAAGATAAGGAAGTTCAAAAAGACATGAAGCTTGTTCCTTATAAGATTGTCAACAAGGATGGAAAACCTTACATTCAGGTGAAAATTAAGGATGGTGAGACCAAGGTGTTCAGCCCTGAGGAAATCAGTGCCATGGTTCTGATCAAGATGAAGGAAACTGCGGAAGCATTCCTCGGGAAGAAAATTAATGATGCTGTGGTCACTGTCCCAGCTTACTTCAATGATGCTCAGAGGCAGGCCACCAAGGATGCTGGTGTCATTGCTGGTCTCAATGTTGCTAGAATTATCAACGAACCCACTGCCGCTGCCATTGCCTATGGATTGGACAAGAAAGGTGGCGAGAAGAACATTCTAGTCTTTGATCTTGGTGGTGGGACCTTTGATGTCAGTATCTTGACAATTGATAACGGTGTTTTTGAAGTTCTTGCTACAAATGGAGATACTCATCTTGGAGGTGAGGATTTTGATCAAAGAATAATGGAGTACTTCATTAAATTGATCAAGAAAAAGCATGGAAAGGATATTAGCAAGGACAACAGAGCACTTGGAAAGCTGAGGAGAGAGGCTGAGCGTGCAAAGAGGGCACTCAGCAGCCAGCACCAGGTCCGCGTGGAAATTGAATCACTTTTTGATGGTGTTGATTTTTCTGAGCCACTCACCCGAGCTAGGTTTGAGGAGTTGAACAATGACTTGTTCCGGAAGACCATGGGTCCAGTGAAGAAGGCTATGGAAGATGCTGGATTACAGAAGAGTCAGATTGATGAGATTGTTCTTGTTGGTGGAAGCACAAGGATTCCAAAGGTACAACAGCTTTTGAAGGACTACTTTGATGGAAAGGAGCCAAACAAGGGGGTCAACCCTGATGAAGCAGTTGCCTATGGTGCTGCAGTGCAAGGAAGCATTTTGAGTGGAGAGGGTGGTGAAGAAACCAAAGATATCCTTCTCCTGGATGTGGCTCCCCTCACTCTCGGAATTGAAACTGTTGGTGGAGTCATGACAAAGTTGATTCCCAGAAACACTGTTATCCCGACCAAGAAATCTCAAGTGTTCACCACCTACCAGGACCAGCAGACTACCGTCTCCATTCAGGTTTTCGAAGGTGAGAGGAGTCTCACAAAGGATTGTCGCCTTCTTGGGAAATTTGATCTGTCTGGAATTCCACCAGCTCCAAGGGGCACCCCTCAAATTGAAGTGACCTTCGAAGTTGATGCCAACGGCATTCTAAATGTGAAGGCAGAAGACAAGGGAACTGGTAAATCAGAAAAGATCACCATTACAAACGAAAAGGGACGTCTTAGCCAGGAAGAAATAGATAGAATGGTTCGTGAAGCAGAAGAGTTTGCCGAGGAAGACAAGAAGGTGAAGGAGAGGATCGATGCTCGCAACAGTCTTGAAACCTACGTATACAACATGAAGAATCAGGTCAGTGACAAGGACAAGCTCGCGGACAAGTTGGAGTCAGATGAAAAGGAGAAAATTGAGACTGCAGTGAAAGAAGCATTGGAATGGCTGGATGACAACCAGAGTGTGGAGAAAGAAGATTATGAAGAGAAGCTGAAGGAGGTTGAAGCCGTTTGCAACCCAATCATCAGTGCTGTGTATCAGAGATCAGGAGGAGCCCCAGGTGGTGCAGGTGGCGAAGGCGAAGACGAGGATGATTCTCACGATGAGCTCTAG

>Glyma08g02960 ATGACATGCTCGTACTTGCGAGTGAGCACTATAAATGGCGAAGTCCCCGCCGTGAACTCAATAGAACCTACAATTCATCGTCCATCGAGAAAGGAAGAAAAGAAACAAGGTGACATCATCATGGCTTGCTCGTTTTCTCGCGGGTCTCTGCTTCCTCTCGCCATCATCGTTTCCTTAGGATGTCTATTTGCGATTTCCATTGCAAAGGAGGAAGCCACCAAGTTGGGGACGGTCATTGGGATTGATCTTGGAACGACCTATTCATGTGTTGGTGTTTACAAGAACGGCCATGTTGAAATCATAGCCAATGACCAAGGTAACCGTATCACCCCTTCTTGGGTTGCTTTCACCGACAGTGAGAGACTCATTGGGGAGGCTGCCAAGAATCTGGCAGCTGTCAACCCAGAAAGGGTCATCTTTGATGTCAAGAGACTTATTGGAAGAAAGTTTGAAGATAAGGAAGTTCAACGAGACATGAAGCTTGTTCCTTATAAGATTGTCAACAAGGATGGAAAACCTTACATACAGGTGAAAATTAAGGATGGTGAGACCAAGGTGTTCAGCCCTGAGGAAATCAGTGCCATGATTCTGACCAAGATGAAGGAAACTGCGGAAGCATTCCTTGGGAAGAAAATTAATGATGCTGTGGTCACTGTCCCAGCTTACTTCAATGATGCTCAGAGGCAGGCCACCAAAGATGCTGGTGTCATTGCTGGTCTCAATGTTGCTAGAATTATTAATGAACCTACTGCTGCTGCCATTGCGTATGGATTGGACAAGAAAGGTGGCGAGAAGAACATTCTTGTTTTTGACCTTGGGGGTGGAACATTTGATGTCAGTATCTTGACAATTGATAATGGTGTTTTTGAGGTTCTTGCTACAAATGGAGATACTCATCTTGGAGGTGAGGACTTTGATCAGAGAATAATGGAGTACTTCATTAAATTGATCAATAAAAAGCATAAAAAGGATATTAGCAAGGACAGCCGAGCACTTGGCAAGCTGAGGAGAGAGGCTGAACGTGCAAAGAGAGCTCTCAGCAGCCAGCACCAGGTCCGCGTGGAAATTGAATCACTTTTTGATGGTGTTGATTTTTCTGAGCCACTCACCCGAGCTCGGTTTGAGGAGTTGAACAATGACTTGTTCCGGAAGACCATGGGACCAGTGAAGAAGGCTATGGAAGATGCTGGATTACAGAAGAATCAAATTGACGAGATTGTTCTTGTCGGTGGAAGCACAAGGATTCCAAAGGTACAACAGCTTTTGAAGGACTACTTTGATGGAAAGGAGCCAAACAAGGGTGTCAACCCTGATGAAGCAGTTGCCTATGGTGCTGCAGTGCAAGGAAGCATTTTGAGTGGAGAGGGTGGTGAGGAAACCAAAGATATCCTTCTCTTGGATGTGGCTCCCCTCACCCTTGGAATTGAAACTGTTGGTGGGGTGATGACGAAGTTGATTCCCAGAAACACTGTTATCCCTACCAAGAAATCCCAGGTGTTTACCACCTACCAGGATCAGCAGAGTACCGTCTCCATTCAGGTTTTTGAAGGGGAGAGGAGTCTCACAAAGGATTGCCGTCTGCTTGGGAAATTTGAACTGTCTGGAATTCCTCCAGCTCCAAGGGGTACCCCTCAAATTGAAGTGACCTTCGAAGTTGATGCAAATGGCATTCTAAATGTGAAGGCAGAAGACAAGGGCACTGGTAAATCAGAAAAGATAACAATTACAAATGAAAAGGGACGTCTTAGCCAGGAGGAAATTGAGCGGATGGTTCGCGAAGCAGAAGAGTTTGCTGAGGAAGACAAAAAAGTGAAGGAGAGGATTGATGCTCGTAACAGTCTTGAAACCTATGTCTACAACATGAAAAACCAGGTCAGTGACAAAGACAAGCTCGCTGACAAGTTGGAGTCTGATGAAAAAGAGAAAATTGAGACTGCAGTGAAAGAAGCATTGGAATGGCTGGATGACAACCAGAGTGTGGAGAAAGAAGAATATGAGGAGAAGCTCAAAGAGGTGGAAGCTGTTTGCAACCCAATCATCAGTGCTGTGTATCAGAGATCAGGTGGAGCCCCAGGTGGTGGTGCATCAGGCGAGGACGACGATGAAGATTCTCATGACGAGCTCTAG

>Glyma08g06950 ATGGCCGCCGCGACCGCCTTGCTCCGCTCTCTCCGCCGCCGCGACCTTCCCTCGTCTTCTCTCTCCGCCTTTCGTTCGTTGACGAGTGGCACAAAGACATCATATGTAGGGAACAAGTGGGCAAGTTTGTCTCGGCCGTTCAGTTCAAAGCCTGCTGGTAATGATGTCATTGGAATTGATCTGGGTACTACCAATTCATGTGTTTCTGTTATGGAGGGAAAGAATCCCAAAGTTATTGAGAATTCTGAAGGAGCTCGAACAACACCATCAGTGGTTGCCTTCAACCAGAAAGCGGAGCTTCTTGTTGGTACACCAGCCAAGCGTCAGGCTGTGACAAACCCAACAAACACTCTTTTTGGAACCAAGCGTTTGATAGGTAGGCGTTTTGATGATTCTCAAACTCAGAAGGAGATGAAAATGGTTCCATACAAGATTGTTAAGGCTCCCAATGGAGATGCTTGGGTTGAAGCCAATGGGCAGCAGTATTCTCCCAGCCAAGTTGGTGCTTTTGTTCTCACCAAGATGAAGGAAACTGCTGAGTCATATCTTGGAAAGTCAGTTTCAAAAGCTGTAATTACTGTACCAGCTTACTTCAATGATGCTCAGAGACAGGCAACAAAAGATGCCGGTAGAATTGCTGGTCTTGATGTTCAAAGAATCATCAATGAGCCCACTGCTGCTGCACTTTCATATGGGATGAACAACAAGGAGGGTCTCATTGCAGTTTTTGATCTTGGAGGTGGAACATTTGATGTGTCCATCTTAGAGATTTCTAATGGTGTTTTTGAGGTGAAAGCCACAAATGGTGACACGTTCTTGGGAGGAGAGGATTTTGATAATGCTTTATTGGATTTTCTAGTGAATGAATTCAAAAGAACTGAGAATATTGATCTTTCAAAGGATAAACTTGCATTGCAGAGGCTTCGAGAAGCTGCTGAGAAAGCAAAAATAGAACTGTCTTCAACATCTCAAACAGAAATCAATCTTCCTTTCATCACTGCTGATGCATCTGGTGCAAAGCATCTGAATATCACATTGACTAGATCTAAGTTTGAGGCTTTGGTAAACCACTTGATTGAAAGGACAAAGGCACCGTGTAAGAGCTGTTTGAAGGATGCTAATGTATCCATCAAGGAAGTTGATGAGGTTCTTCTTGTTGGAGGGATGACCCGGGTGCCTAAAGTCCAGGAGGTGGTTTCAGCGATCTTTGGAAAGAGTCCTAGCAAAGGAGTAAATCCTGATGAGGCAGTTGCCATGGGAGCAGCTATTCAGGGTGGTATCCTACGTGGAGATGTTAAAGAGCTACTACTCCTAGATGTCACTCCACTTTCTCTGGGTATTGAGACTTTGGGTGGTATCTTTACCAGGTTGATTAACCGCAACACCACAATTCCTACTAAAAAGAGTCAGGTGTTTTCAACAGCAGCTGACAATCAGACTCAGGTTGGTATCAAGGTGCTACAAGGTGAGAGGGAGATGGCTGTAGACAACAAATCGCTTGGAGAATTTGAGCTTGTTGGCATTCCTCCTGCCCCAAGAGGCATGCCTCAGATTGAAGTCACATTTGATATAGATGCCAATGGGATTGTTACTGTCTCTGCCAAAGACAAGTCCACTGGGAAAGAACAACAAATCACCATCCGTTCATCTGGAGGACTCTCAGAAGATGAGATTGATAAGATGGTCAAAGAAGCAGAGTTGCATGCTCAGAAAGACCAAGAAAGAAAGGCTCTCATTGACATCAGAAACAGTGCAGATACAACCATCTACAGCATCGAGAAGAGTTTAGGTGAGTACAGAGATAAGATCCCCAGTGAAGTGGCCAAAGAGATTGAAGATGCAGTATCAGATTTGAGAACAGCAATGGCAGGAGACAATGCTGATGAAATTAAGGCAAAGCTTGACGCTGCAAACAAAGCTGTCTCCAAGATTGGAGAGCACATGTCAGGTGGTTCTAGTGGCAGTTCCTCAGCTGGAGGTTCTCAGGGTGGTGAACAGGCTCCCGAGGCAGAGTATGAGGAAGTCAAGAAATGA

>Glyma08g22100 ATGAGCGTGGTGGGATTCGATTTCGGTAACGAGAGTTGCATTGTTGCGGTTGCGAGGCAGAGAGGGATTGACGTTGTGCTCAATGATGAGTCCAAGCGTGAAACGCCCGCCATTGTGTGCTTCGGTGACAAGCAACGCTTCATTGGCACTGCTGGTGCTGCCTCCACTATGATGAACCCTAAGAATTCAATCTCCCAGTTTAAGAGACTCATTGGTAGGAAATTCTCTGATCCCGAATTGCAGCGGGATCTTAAGTCGTTGCCGTTTCTTGTCACCGAGGGGAGTGATGGGTACCCGTTGATTCATGCGCGGTACATGGGTGAGTCCAAGACGTTTACGCCTACCCAAGTGTTTGGAATGATGCTGTCGAATCTTAAGGAAATTGCGGAGAAGAATCTTACTACAGCGGTTGTGGATTGTTGCATTGGAATCCCGGTTTATTTTACTGATCTGCAGAGAAGGGCGGTGCTGGATGCGGCCACGATTGCTGGTCTGCACCCACTTCGGTTGATTCAGGAAATGACTGCCACTGCCTTGGCCTATGGGATTTATAAAACGGACCTTCCAGAAAACGATCAGCTGAATGTTGCGTTTGTTGATGTTGGGCATGCTAGCATGCAAGTGTGCATTGCTGGATTCAAGAAGGGGCAGCTGAAAGTGTTGGCTCATTCATATGATAGGTCTCTCGGCGGTAGGGATTTTGACGAGGTTTTGTTCCATCACTTTGCTGGGAAGTTTAAGGAGGAGTACAAGATTGATGTTTTCCAGAATGCCAGGGCTTGCATCAGGCTCAGGACTGCCTGTGAGAAGATCAAGAAGATGCTTAGTGCGAATCCTGTGGCGCCTCTCAACATTGAGTGCTTGATGGATGAGAAGGATGTCCGGGGCTTCATCAAGCGAGATGAGTTTGAGCAACTAAGTCTTCCAATTTTGGAACGTGTGAAGGGACCTCTGGAGAAGGCACTTGCTGAAGCAGGTCTTACCGTTGAAAATGTACACACAGTTGAGGTGGTTGGTTCAGGTTCTCGGGTACCGGCCATTAACAAAATATTGACAGAGTTTTTCAAAAAGGAGCCTAGGCGGACAATGAATGCTAGTGAGTGTGTTGCTAGGGGCTGTGCGTTGGAATGCGCAATTCTTAGTCCAACGTTCAAAGTACGAGAATTTCAGGTCAACGAAAGCCTTCCTTTCTCGATTTCTCTTTCATGGAAAGGTTCTGGTCCAGATGCACAGGACAATGGATCAGAAAATCAGCAGAGTTCCCTTGTTTTTCCTAAGGGTAATCCCATACCAAGTATCAAGGCACTGACATTCTGCAGGGCAGGAACATTCTCTGTTGATGTATTATATGATGATGCAAGTGGGCTGCAAACACCTGCTAAGATCAGCACATATACTATTGGTCCTTTCCAAACTACAAATGGTGAAAGGGCAAAAGTTAAAGTGAAAGTTCGTCTGAATCTGCACGGAATTGTATCCCTTGAGTCTGCAACACTCCTGGAAGAGGAAAAAGTTGGGGTTCCAGTTACCAAAGAGGCAGCAGGGGAAAATACTAAGATGGACATTGATGAAGTCCCTGCTGAGGCTGCTGCACCTCCCGCCTCCAATGACACTGGTGCTAATATGGAAGGTGCAAAGGCTAGTACTGATGCCTCTGGGGTTGAAAATGGCATCCCTGAGGGTGGAGATAAGCCTTTGCAAAAGGATACTGATACCAAGGTTCAGGCTCCAAAGAAAAAGGTTAAGAAAACAAACATTCCTGTAGCAGAGTTAGTTTATGGAGCAATGGTGCCTGTGGATGTCCAGAAAGCACTAGAAAAGGAGTTTGAAATGGCTTTGCAAGATCGTGTGATGGAAGAAACAAAAGACAAGAAAAATGCAGTTGAGGCTTATGTTTATGACATGAGAAACAAGCTTAATGACGAATACCAAGAGTTTGTCACTGCTTCAGAGAGAGATGATTTTACTGCTAAACTTCAGGAAGTGGAAGATTGGCTTTATGATGAGGGTGAAGATGAAACTAAAGGTGTATATATTGCCAAGCTTGAAGAACTCAAAAAGCAAGGTGATCCAATTGATGGGCGATACGAAGAATTCACGGAGAGGGGTACTATAATTGAACAGTTCGTGTATTGTATAAATAGTTACAGACAAGTTGCAATGTCAAATGATCCTAGATTTGAGCACATTGACATTAACGAGAAACAGAAGGTCATAAATAAATGTGTTGAAGCTGAGAAGTGGTTTAATGAGAAGCAGCAGCAGCAGAGCTCACTTCCAAAATATGCCAACCCTGTACTCTTGTCAGCTGAAATGAGAAAGAAAGCTGAAGATGTCGATAGGTTCTGCAAGCCGATTATGACAACACAGAAGCCAACAAAGGCAGTTACTCCAGCAGGACCAGCAACCCCATCTTCTCAGAGTGATGAGCAACAGCAACCCCAGGGGGATTCTGATGTCAACAGTAATGAGAATGCTGGGAATAGCAGTAGTCAGGCTGCACCAGCATCTACCGAACCAATGGAGACTGAAAAGTCTGAGAACACAGGCTCTGCCTAA

>Glyma08g42720 ATGAGTGTGGTGGGGTTTGACATTGGTAATGAGAACTGTGTCATTGCTGTAGTTAGGCAACGTGGCATTGATGTTTTGTTGAATTATGAATCCAAACGTGAAACCCCGGCTGTGGTCTGCTTCAGCGAGAAGCAGCGGATTTTGGGGTCTGCTGGTGCTGCTTCTGCTATGATGCACATCAAGTCCACAATATCTCAAATAAAGAGACTAATTGGAAGGAAGTTTGCAGACCCTGATGTGAAAAAAGAGCTGAAAATGCTCCCTGGTAAGACTTCTGAGGGTCAAGATGGAGGCATTTTGATTCACTTGAAGTACTCGGGGGAGATTCATGTATTTACACCTGTTCAATTTCTGTCCATGCTCTTTGCTCACTTAAAGACTATGACCGAAAACGATTTGGAGATGCCCATTTCAGATTGTGTTATTGGGATCCCATCTTACTTTACCGACTTGCAGAGACGGGCGTATCTTGATGCAGCGAAAATTGCCGGGTTGCAGCCTTTGAGATTGATCCATGATTGTACTGCAACTGCCCTTAGTTATGGAATGTATAAAACAGATTTTGGCAGTGCAGGTCCAGCTTATGTTGCATTTATTGACATTGGTCACTGTGATACTCAGGTCTGTATTGCATCATTTGAGTTTGGGAAAATGGAGATACTTTCACATGCATTTGATAGGAGCTTAGGAGGGAGGGACTTTGATGAGGTTATATTTAGTCATTTTGCAGCAAAATTCAAGGAAGAGTACCACATTGACGTGTATTCTAAAACAAAGGCGTGCTTTAGGCTACGTGCAGCATGTGAGAAATTGAAGAAAGTTTTGAGTGCAAATCTAGAGGCACCTCTAAATATCGAGTGCTTGATGGACGGGAAAGATGTTAAGGGATTTATCACAAGGGAAGAATTTGAGAAGCTGGCATCAGGATTACTGGAGAGAGTTTCTATTCCTTGCCGCAGAGCATTAACTGATGCAAACTTGACAGCAGAGAAGATTTCTTCTGTAGAGCTAGTTGGTTCAGGTTCTAGGATTCCAGCTATAAGTACATCACTAACTTCTCTGTTCAAGAGAGAACCCAGCCGACAGCTGAATGCAAGTGAGTGTGTAGCTCGTGGTTGTGCTCTACAGTGTGCAATGCTCAGTCCTGTTTACCGCGTGAGAGAATACGAGGTCAAGGATGTTATTCCCTTTTCAATTGGACTTTCATCAGATGAAGGTCCAGTTGCTGTGAGATCAAATGGTGTACTTTTCCCAAGAGGCCAACCCTTTCCAAGTGTTAAAGTCATAACCTTTCAGCGAAGTAATTTGTTTCATTTGGAAGCTTTCTATGCTAACCCAGATGAACTACCACCTAGGACATCTCCTAAAATTAGCTGTGTCACGATTGGTCCTTTCCATGGATCCCATGGTAGTAAGATCAGAGTTAAAGTTAGAGTTCCACTTGATCTGCATGGCATTGTCAGTATTGAATCAGCTACATTGATCAAGGATGACATGGATGATTTGGTTATGGCTGGTGATTATCATTCAAATTCTGATGCAATGGACATTGATCCCATTTCTGAGACAGTTACCAATGGGTTTGAAGATGATACCAATAAGAAGTTGGAATTTCCATGTAGTTCTGCTGATGGTACAAGAAAAGATAATAGAAGGCTTAATGTGCCAGTGAATGAGAATGTCTATGGTGGAATGACAAAGGCAGAGATCTCAGAAGCTCTTGAAAAAGAACTCCAGTTAGCCCAACAGGACAGAATTGTAGAGCAAACCAAAGAAAAGAAGAATAGCTTGGAGTCTTTTGTTTATGATATGAGGAGTAAGCTCTTCCACACATATCGGAGCTTTGCAAGTGAACAAGAGAAGGATGGCATATCTAGAAGCCTTCAAGAGACTGAGGAATGGCTTTATGAGGATGGTGTTGATGAAACTGAACATGCTTATTCTTCAAAACTGGAAGATCTGAAAAAGCTGGTAGATCCAATTGAGAATCGGTACAAAGATGATAAAGAAAGAGTGCATGCTACACGAGATTTATCAAAGTGCATTTTAAAGCATCGTGCTTCTGCAGATTCCCTTCCACCCCAGGATAAAGAACTGATCATCAATGAGTGCAATAAAGTGGAGCAGTGGTTGAAAGAGAAGATCCAGCAACAAGAATCATTTCCAAAGAATACTGACCCAATATTATGGTCAAGTGATATCAAGAGCAAGACAGAGGAGTTAAACTTAAAATGCCAACAGATATTGGGATCTAACGCTTCTCCATCTCCAGAAGACAAAGACAAGCCGGATACTTTCAATGATCCATGA

>Glyma11g14950 ATGGCTGGAAAAGGCGAGGGTCCTGCTATCGGAATCGATTTGGGAACGACGTACTCTTGCGTCGGCGTGTGGCAACACGATCGTGTTGAAATCATAGCCAACGATCAGGGTAACAGAACTACCCCATCCTACGTGGCTTTCACCGACACAGAACGGTTGATCGGCGATGCGGCGAAGAACCAGGTCGCTATGAACCCAACCAACACCGTTTTTGATGCTAAGCGTTTGATTGGAAGGCGTTTTTCCGATGCATCAGTACAAGGTGACATGAAATTGTGGCCGTTCAAGGTGATTCCTGGCCCTGCTGAGAAACCTATGATTGTGGTGAACTACAAGGGGGAGGAGAAACAGTTTTCCGCGGAAGAGATATCCTCCATGGTTCTTATGAAGATGAAGGAGATTGCGGAGGCGTATCTAGGTTCCACCATAAAGAATGCGGTTGTCACTGTGCCTGCTTACTTCAACGACTCACAACGTCAAGCCACCAAGGACGCTGGTGTCATTTCTGGGCTCAACGTGATGCGAATTATCAACGAGCCTACCGCGGCTGCCATTGCTTATGGGCTCGACAAAAAGGCCACTAGCTCTGGGGAGAAGAATGTTCTCATTTTTGATCTCGGTGGTGGGACTTTTGATGTCTCTCTTCTCACCATCGAGGAGGGTATTTTCGAGGTGAAGGCCACTGCTGGTGATACTCACTTGGGAGGTGAAGATTTTGATAACAGAATGGTGAACCATTTTGTTCAGGAATTCAAGAGGAAGAACAAGAAGGATATTAGTGGAAATGCCAGAGCTCTGAGGAGGTTGAGAACAGCATGTGAGCGGGCAAAGAGGACTCTCTCTTCCACTGCTCAAACCACCATAGAGATTGATTCCTTGTACGAGGGTATTGACTTCTACACAACCATTACCCGTGCCCGTTTTGAAGAGCTAAACATGGATTTATTCAGGAAGTGCATGGAGCCCGTGGAGAAGTGTTTGCGGGATGCCAAGATGGATAAGAGTACCGTCCATGATGTTGTTCTTGTTGGTGGTTCTACTAGGATTCCCAAGGTTCAACAGTTGTTGCAGGACTTCTTCAACGGAAAGGAACTTTGCAAGAGTATTAACCCAGATGAAGCTGTTGCTTATGGTGCTGCAGTGCAGGCTGCGATTCTCAGTGGCGAGGGTAATGAGAAAGTGCAGGATCTTCTTTTGTTGGATGTTACTCCTCTATCCACTGGTTTGGAGACTGCAGGAGGAGTCATGACTGTGTTGATTCCCAGAAACACAACCATTCCCACCAAGAAGGAGCAGGTGTTCTCAACCTACTCTGACAACCAGCCCGGTGTGTTGATTCAGGTCTATGAAGGTGAACGAACGAGGACTCGTGACAACAATTTGCTTGGCAAATTTGAGTTATCTGGAATTCCTCCTGCTCCCAGAGGTGTTCCTCAGATCACTGTTTGCTTCGACATTGATGCCAACGGTATATTGAACGTGTCTGCGGAGGACAAAACCACTGGACAGAAGAACAAGATTACAATTACCAACGACAAGGGCAGGCTTTCTAAGGAGGAGATTGAGAAGATGGTGCAGGAAGCTGAGAAATACAAGTCTGAGGACGAGGAGCATAAGAAGAAAGTGGAGGCCAAAAATGCATTGGAAAATTATGCCTATAACATGAGGAACACAATCAAGGATGACAAGATTGCTTCCAAACTGTCTTCTGATGATAAGAAGAAAATTGAAGATGCGATTGAGCAGGCTATCCAATGGCTAGATGGAAACCAACTTGCTGAGGCTGACGAATTTGAGGATAAGATGAAGGAGTTGGAGAGCATTTGTAATCCCATCATAGCAAAGATGTACCAGGGTGCTGGTGGTGATGCGGGTGGAGCCATGGATGAGGATGGTCCTGCAGCTGGCAGTGGAAGCGGTGCTGGACCCAAAATTGAGGAAGTCGATTAA

>Glyma11g31670 ATCAATCTGGGAACAACTTACTCATGTGTTGCAGTATGGCGGGAGCATCACCGTCGAGTGGAGATAATCCACAATGATCAGGGCAACACTAGGAGTGAAGCAACTAATGATCAGAATTCTTTTAAGTTTGCAGATTCTAAGAGATTGATTGGTAGGAAATATAGTTGTTGCCGGGTGAGGAGAAGCACTTTTGTGCTGAGGAAAAAAATGTCTATCATCAATGGTTCTTGCGAAGATAATGAAGTGGTTACTGTGCCTGCCTATTTCAATGATTCTCAGTATAAAGCTACCATAGATGCTGGAAAAATTGCAGGCCTAAATATTCTGCGGATAATCAATGAACCCGTTGCTGCGGCAATCATGCATGGTCTTGACATGAGGACTAATAATTGTGTTGGAGAGCGAAACATTTTCATCTTTGACCTTGGTGGTGGTACTTTTGATGCGTCTCTCCTTACTCTTAAGGGTAAGATCTTTAAAGTTAAGGCCACAGCTGGAAATGGTCACCTTGGGGGAGAGGACATTGATAATAGAATGCTGGACCATTTTGTAAAGGAGATCAAAAGAAAAAAAAAAGTGGACATTAGTGGGAACCTGAAGGTGCTGAGGAGGTTAAAAACCACGTGCGAGAGGGCAAAAAGAACACTCTCACATGCTGTTACTACCAACATTGAAGTAGATGCTTTATCTGATGCCATTGACTTCTGTTCTTCGATCACTCGTGCAAAGTTTGAGGAAATCAATATGGAGCTCTTTAAGGAGTGTATGGAGACAGTGGATAAGTGTCTTACTGATTCTAAGATGAACAAGAGCAGTGTACATGATGTTATCCTTGTTGTGGTTCTTCAAGGATTCCCAAAGTGCAAGAGCTATTGCAGGACTTTTCCAACGGAAAGGATCTGTGAAAGAGCATCAACCCTGATGAAGCTGTTGCTTATGGTGCAGCTAATGATTCAAGAAGCAGAGGAATACCAAGCTGAAGATAAGAAATTCCTTAGGAAGGCCACTGCAATGAATAAATTGAATGATTATGTTAACAAGATGAACAATGGGTTGGAAAATGAAAATTTAAGTTCAAAACTCTGCTCAGAAGACAAGGAGAAGATAAGTTCTGCAATTACAAAGGCCACAAAGTTGATTGATGGTGATAATAAAAAATGA

>Glyma11g31673 ATGGCTAAAGAAGGTCATAGAATTGCCATAGGAATCAATCTGGGAACAACTTACTCATGTGTTGCAGTATGGCGGGAGCATCACCGTCGAGTGGAGATAATCCACAATGATCAGGGCAACACTAGGAGTGAAGCAACTGTTGATTCTAAGAGATTGATTGGTAGGAAATATAGTGATCCTGTGGTTCAAAAGGACAAGCTGTTATGGCATTCAAGGTTGTTGCCGATGTGGCAGATTGCAGAAGCGTTTTTGGAGAAACATGCAAAGAATGAAGTGGTTACTGTGCCTGCCTATTTCAATGATTCTCAGTATAAAGCTACCATAGATGCTGGAAAAATTGCAGGCCTAAATATTCTGCGGATAATCAATGAACCCGTTGCTGCGGCAATCATGCATGGTCTTGACATGAGGACTAATAATTGTGTTGGAGAGCGAAACATTTTCATCTTTGACCTTGGTGGTGGTACTTTTGATGCGTCTCTCCTTACTCTTAAGGGTAAGATCTTTAAAGTTAAGGCCACAGCTGGAAATGGTCACCTTGGGGGAGAGGACATTGATAATAGAATGCTGGACCATTTTGTAAAGGAGATCAAAAGAAAAAAAAAAGTGGACATTAGTGGGAACCTGAAGGTGCTGAGGAGGTTAAAAACCACGTGCGAGAGGGCAAAAAGAACACTCTCACATGCTGTTACTACCAACATTGAAGTAGATGCTTTATCTGATGCCATTGACTTCTGTTCTTCGATCACTCGTGCAAAGTTTGAGGAAATCAATATGGAGCTCTTTAAGGAGTGTATGGAGACAGTGGATAAGTGTCTTACTGATTCTAAGATGAACAAGAGCAGTGTACATGATGTTATCCTTGTTGTGAGCATCAACCCTGATGAAGCTGTTGCTTATGGTGCAGCTGTGCATGCTGCTTTGCTGAGTGAAGACTGTCAAGGGAAGAAATTAATAGAATGA

>Glyma11g31810 ATGGAGAAGCTTAACTTGGCACTCGTTTCTTCCCCAAAGCCTTTGATGTTGGGACATGTTCCTGCAAGAGACGTTTTCAGAAGAAAACACTTCTCTTTTGGGAGGGTCTTAATTGCTCCTCACCGCTGCAGATTCCGTGTTTCTGCACTCTCTTCCTCCCATCATAACCCCAAATCTGTGCAGGAGAAGCTGATAGTAAAGCATTTTGCTAGTATTTCTTCTTCAAATACTCAAGAAACAACATCAATTGGAGTTAAGCCACAATTATCACCATCTCCATCTTCAACTATAGGGTCACCTCTCTTCTGGATTGGTGTTGGTGTTGGGCTTTCTGCACTGTTTTCAGTGGTAGCTTCAAGACTGAAGAAATATGCAATGCAACAAGCTTTCAAGACCATGATGGGTCAGATGAATTCTCAAAATAACCAATTTGGCAATGCTGCCTTTTCTCCGGGATCACCTTTTCCCTTTCCAATGCCTACAGCAGCAGGGCCCACTGCACCTGCTAGTTCTGCAACTACTCAATCTCGAGCACCTTCAGCATCTAGTGCATCTCAATCCACTATCACAGTAGATTTACCTGCAGCAAAAGTAGAAGCTGCTCCAACTACTAATGTTAAAGATGAAGTCGAACTAAAGAATGAACCCAAAAAAATTGCTTTTGTAGATGTTTCTCCAGAAGAAACTGTGCGGGAGAGTCCTTTTGAAAGTTTTAAAGATGATGAATCAAGTTCCGTCAAGGAAGCTTGGGTTCCAGATGAAGTTTCTCAAAATGGAGCTCCCTCTAACCTAGGTTTTGGTGATTTTCCTGGTTCTCAATCTACAAAAAAATCAGCCTTGTCAGTGGATGCTTTGGAGAAAATGATGGAGGACCCAACAGTGCAGAAGATGGTTTATCCCTATTTACCTGAGGAGATGAGGAACCCTACTACCTTCAAATGGATGCTGCAGAATCCACAGTACCGTCAACAACTTGAAGAAATGCTAAACAACATGGGTGGAAGCACTGAATGGGACAATCGAATGATGGATACCTTGAAGAATTTTGACCTTAATAGTCCTGAAGTGAAGCAGCAATTTGATCAAATTGGGCTTTCTCCCGAAGAAGTCATTTCAAAGATTATGGCCAATCCTGAAGTTGCAATGGCATTTCAAAATCCTAGAGTTCAAGCAGCTATCATGGATTGTTCGCAGAATCCAATGAATATTACTAAATACCAAAACGATAAGGAGGTAATGGATGTCTTCAACAAAATATCAGAACTCTTCCCTGGAGTAGGTTCACCTTGA

>Glyma12g06910 ATGGCTGGAAAAGGCGAGGGTCCTGCTATCGGAATCGATTTGGGAACGACGTACTCTTGCGTCGGCGTGTGGCAACACGATCGTGTTGAAATCATAGCCAACGATCAGGGTAATAGAACTACCCCATCCTACGTGGCTTTCACCGATACAGAACGGTTGATCGGCGATGCGGCGAAGAACCAGGTCGCTATGAACCCAATCAACACTGTTTTTGATGCTAAGCGTTTGATTGGAAGACGTTTTTCTGATGCATCGGTACAAAGTGACATGAAACTGTGGCCGTTCAAGGTTATTCCTGGCCCTGCTGACAAGCCTATGATTGTGGTGAATTACAAGGGGGATGAGAAGCAGTTTTCGGCGGAAGAGATATCTTCCATGGTTCTTATAAAGATGAAGGAGATCGCGGAGGCGTATCTAGGATCGACGATAAAGAATGCAGTTGTCACCGTCCCTGCTTACTTCAACGACTCACAGCGTCAAGCTACAAAGGACGCTGGTGTCATTTCCGGGCTCAACGTGATGCGAATTATCAACGAGCCTACAGCGGCCGCCATTGCTTATGGACTCGACAAGAAGGCCACTAGCTCTGGGGAGAAGAATGTTCTCATCTTCGACCTCGGTGGTGGGACTTTTGATGTCTCCCTTCTCACCATTGAGGAGGGTATTTTCGAAGTGAAAGCCACTGCTGGTGATACTCACTTGGGAGGTGAAGATTTTGATAACAGAATGGTGAACCATTTTGTTCAGGAATTCAAGAGGAAGAACAAGAAGGATATTAGTGGAAATGCCAGAGCATTGAGGAGGTTGAGAACAGCATGTGAGAGGGCCAAGAGGACTCTCTCTTCCACTGCTCAAACCACCATTGAGATTGATTCCTTGTATGAGGGTATTGACTTCTACACAACCATCACCCGTGCCCGTTTCGAAGAGCTTAACATGGACCTATTCAGGAAGTGCATGGAGCCCGTGGAGAAGTGTTTGCGGGATGCTAAGATGGACAAGAGCACCGTCCATGATGTTGTTCTTGTCGGTGGTTCTACTAGGATTCCCAAGGTTCAACAGTTGTTGCAGGACTTCTTCAACGGAAAGGAGCTTTGCAAGAGCATTAACCCAGATGAAGCTGTTGCCTATGGTGCAGCAGTGCAGGCTGCCATTCTCAGTGGCGAGGGTAATGAGAAAGTGCAGGATCTTCTGTTGTTGGATGTTACTCCTCTCTCCCTTGGGTTGGAGACCGCGGGAGGAGTCATGACTGTGTTGATTCCCAGAAACACAACCATTCCCACCAAGAAGGAGCAGGTATTTTCGACCTATTCCGACAACCAGCCTGGTGTGTTGATTCAGGTCTATGAAGGTGAACGTACGAGGACTCGTGATAACAATTTGCTTGGCAAATTTGAGTTATCTGGAATTCCTCCTGCTCCGAGAGGTGTTCCTCAGATCACCGTTTGCTTTGACATTGACGCCAACGGTATATTGAACGTGTCTGCGGAGGACAAAACCACTGGACAGAAGAACAAGATTACAATTACCAACGACAAAGGAAGGCTTTCTAAGGAGGAGATTGAGAAGATGGTGCAGGAAGCTGAGAAGTACAAGGCTGAGGATGAGGAGCATAAGAAGAAAGTGGAGGCCAAAAATACATTGGAGAATTACGCCTATAACATGAGGAACACAATTAAAGATGATAAGATTGCTTCCAAGCTGTCTGCTGATGATAAGAAGAAAATTGAGGATGCAATTGAGCAGGCTATCCAATGGCTCGATGGAAACCAACTTGCTGAAGCTGACGAATTCGAGGACAAGATGAAGGAGTTGGAGAGCATTTGTAACCCCATCATAGCAAAGATGTACCAAGGAGCTGGTGGTGATGCGGGTGGAGCCATGGATGAGGATGGTCCTGCTGCTGGCAGTGGAAGCGGTGCTGGACCCAAAATTGAGGAAGTCGATTAA

>Glyma12g28750 ATGGCTTGCTCAAGCGCCCAAATACACGGTCTCGGAACCCCTTCCTCCCGAACCCTATTTTTAGGTCAGAGGCTAAATACCAAGGCCGCCTTTATCAAGCTCAAGTCCACACCTAGAAGACTCCGCCCTCTCAGAGTGGTTAATGAGAAAGTCGTCGGTATCGATTTGGGAACCACCAACTCCGCCGTGGCTGCCATGGAAGGCGGCAAGCCCACTATCATCACCAACGCCGAGGGCCAGAGAACCACGCCCTCCGTCGTGGCCTACACCAAGAATGGCGACAGGCTCGTTGGCCAAATCGCCAAGCGTCAGGCCGTCGTCAACCCCGAGAACACTTTCTTCTCCGTCAAGAGGTTCATCGGCCGCAAGATGTCTGAGGTCGACGAAGAGTCCAAGCAGGTCTCTTACAGAGTCATCAGAGACGACAACGGCAACGTCAAACTCGACTGCCCCGCCATTGGCAAACAGTTCGCTGCCGAGGAAATTTCTGCCCAGGCTGGTGTCTTGGCTGGAGATGTCAGTGATATTGTGCTGTTGGATGTCACTCCATTATCTTTGGGTCTGGAAACTCTAGGTGGTGTGATGACAAAAATTATTCCAAGAAACACTACTCTTCCCACCTCAAAGTCCGAGGTTTTCTCAACCGCTGCTGATGGACAGACCAGTGTAGAGATCAATGTCCTTCAGGGGGAGAGAGAATTTGTTAGGGACAACAAATCACTTGGTAGCTTCCGCTTGGATGGTATCCCTCCTGCGCCTCGTGGTGTTCCCCAGATTGAGGTGAAATTTGACATTGATGCCAATGGCATTCTATCCGTCACTGCTATCGACAAAGGCACAGGGAAGAAACAAGATATTACCATCACTGGTGCTAGCACCTTGCCTTCAGATGAGGTGGAGAGAATGGTAAACGAAGCTGAGAAATTTTCGAAGGAAGACAAAGAGAAGAGGGATGCTATTGACACAAAGAACCAGGCAGACTCTGTGGTATACCAGACAGAAAAGCAATTGAAAGAGCTTGGAGACAAGGTTCCTGGCCCTGTGAAAGAGAAGGTTGAAGCAAAACTAGGAGAGCTTAAAGACGCCATTTCTGGGGGTTCAACCCAAGCTATCAAGGATGCCATGGCTGCACTAAATCAGGAAGTCATGCAGCTTGGTCAGTCCCTTTACAACCAACCAGGAGCTGCAGGCGCAGGAGGGCCAACACCACCACCCGGTGCCGACTCTGGCCCCTCAGAATCCTCAGGCAAGGGACCCGACGGTGATGTCATTGATGCAGATTTCACTGACTCTAAATGA

>Glyma13g10700 ATGGCGTCGAAGGTGGCGCTGATGGCGCTGTTCTCCGTCGCGCTCCTGTTCTCTCCGTCGCAATCCGCGGTCTTCAGCGTAGATCTAGGCTCCGAATCGGTGAAGGTGGCGGTGGTGAACCTCAAGCCCGGCCAATCCCCGATCTCCGTCGCGATCAACGAGATGTCCAAACGCAAATCGCCGGCGCTGGTCTCCTTCCATGACGGCGACCGCCTCCTCGGAGAGGAGGCCGCCGGCCTCGCCGCGCGCTACCCGCAGAAGGTCTATTCCCAAATGCGCGACCTCATCGCCAAACCCTACGCCTCCGCGCAGAGGATTCTCGACTCAATGTACTTGCCATTCGATGCAAAGGAAGATTCACGAGGCGGCGTGAGTTTCCAAAGCGAAAACGACGACGCTGTTTATTCCCCCGAGGAGCTGGTTGCCATGGTGTTAGGTTACACGGTGAATTTGGCAGAGTTTCACGCAAAGATTCAGATAAAGGACGCGGTGATCGCGGTGCCGCCGTACATGGGGCAGGCGGAGCGGAGAGGGCTGCTTGCCGCGGCGCAGTTAGCGGGGATTAACGTTTTGTCTCTGATAAACGAGCATTCCGGCGCGGCGCTGCAGTACGGGATCGACAAGGACTTCTCCAACGAGTCTCGGCACGTGATCTTCTACGACATGGGCGCGAGCAGCACCCACGCGGCGCTCGTTTACTTCTCGGCGTACAAGGGGAAGGAGTACGGGAAGAGCGTGTCGGTGAATCAGTTTCAGGTGAAGGACGTGCGCTGGGATCCGGAGCTCGGTGGCCAGCACATGGAGCTGCGGTTGGTGGAGTATTTTGCGGATCAGTTCAATGCGCAGGTTGGAGGTGGAATCGATGTCAGGAAGTTCCCCAAGGCTATGGCTAAGTTGAAGAAACAGGTTAAAAGGACTAAAGAGATACTTAGTGCTAACACAGCAGCTCCTATTTCAGTTGAATCGCTTCATGATGACGTCGACTTCAGGAGCACAATAACCCGTGAGAAATTTGAAGAGCTTTGTGAAGACATTTGGGAAAAATCGCTCTTACCTGTGAAAGAGGTGCTTGAGAATTCTGGCCTGTCATTGGAACAAATATATGCAGTGGAGTTGATTGGAGGTGCCACCAGAGTGCCAAAATTACAGGCTAAGCTTCAAGAATTCCTCAGGAGAAAAGAACTTGATAGGCATCTTGATGCTGATGAAGCAATAGTTCTTGGCGCAGCTCTGCATGCTGCAAATTTAAGTGATGGAATCAAATTGAACCGCAAACTAGGAATGATTGATGGTTCCTTATATGGATTTGTGGTTGAGTTGAATGGTCCTGATCTTTTAAAAGATGAAAGCTCTAGGCAGCTACTTGTGCCACGAATGAAGAAGGTCCCGAGTAAGATGTTTAGATCCATTAATCATAACAAGGATTTTGAAGTTTCACTTGCTTATGAAAGTGAGAATCATTTGCCTCCTGGTGTTACCTCTCCTGAAATTGCTCGATACCAGATATCTGGTTTGACAGATGCAAGTGAGAAATACTCATCTCGGAATCTGTCATCCCCCATCAAGACAAACATACATTTTTCTCTTAGTAGAAGTGGAATTCTTTCACTGGATCGGGCTGATGCTGTTATTGAAATAACAGAGTGGGTGGAAGTTCCTAGAAAGAATTTGACCATAGAGAATTCAACCGTTTCATCAAATGTTTCAGCTGAATCTGCTGCGGGTAATAGTTCTGAAGAAAACAACGAAAGCGTGCAAACTGATAGTGGGATTAATAAGACATCCAACATTAGTTCAGAGGAGCAAGCTGCTGCTGAGCCTGCTACAGAGAAAAAGCTGAAAAAGCGGACCTTTAGGGTACCATTAAAGATTGTAGAGAAGATAACTGGATTTGGAATGTCTCTATCACAAGATTTTCTTGCTGAAGCCAAAAGAAAACTACAAGTACTAGATAAAAAAGATGCAGACAGAAAAAGAACAGCTGAGTTAAAAAATAATTTAGAAGGATATATATATACAACTAAGGAAAAGATTGAAACTCTTGAGGAGTTTGAAAAAGTTTCTACAAGTGAGGAACGCCAGTCCTTCATTGAGAAGCTTGATCAGGTGCAAGATTGGTTGTATACAGATGGTGAAGATGCCAATGCCACAGAGTTTCAAGAGCGTCTAGATCAGTTAAAAGCTGTTGGAGATCCAATTTTCTTCAGGTTAAAAGAGCTTACAGCTCGGCCAGCAGCAGTTGAGCATGCTAATAAATACATTGATGAGTTGAAACAGATTGTTGAGGAGTGGAAAGCAAAGAAGTCTTGGCTTCCACAAGAACGAGTAGACGAGGTCATAAAAAGTTCTGAAAAATTGAAGAATTGGTTGGATGAGAAAGAAGCTGAGCAAACAAAGACTTCTGGATTCAGTAAGCCAGCATTTACATCTGAAGAAGTATATCTGAAGGTGCTTGATCTGCAAACCAAGGTTGCCAGTATTAATAGAATTCCCAAGCCCAAACCTAAGGTTCAGAAGCCCGTAAAAAATGAAACCGAAAGCAGTGAGCAGAATACAGAGAATTCTGATTCTAACTCAGCTGATAGTTCCTCTTCAAGTGATTCATCCGTTAACAGTTCAGAAGGCACAAGCGAAGAGACGGTAACTGAGCAAACTGAAGGTCATGATGAGCTATGA

>Glyma13g19330 ATGGCTGGCAAAGGAGAGGGTCCAGCCATCGGAATCGATCTCGGAACCACGTACTCGTGCGTCGGTGTCTGGCAACACGATCGCGTCGAGATCATCGCCAACGACCAAGGTAACAGAACGACGCCGTCTTACGTCGGGTTCACCGACACCGAGCGTCTCATTGGTGATGCCGCAAAAAATCAGGTCGCCATGAACCCCATCAACACCGTCTTTGATGCCAAGAGATTGATTGGTCGTAGATTCAGTGATGCCTCTGTTCAGAGTGATATTAAACTGTGGCCTTTTAAGGTTTTGTCTGGACCTGCTGAAAAGCCAATGATTCAGGTCAGTTACAAGGGTGAGGATAAGCAGTTTGCTGCAGAGGAAATCTCTTCTATGGTGCTAATGAAAATGCGTGAGATTGCTGAAGCTTATCTTGGCTCTTCAATCAAGAATGCCGTTGTTACTGTCCCTGCTTACTTTAACGATTCTCAGCGTCAAGCTACCAAGGATGCTGGTGTTATTGCTGGTCTCAATGTGATGCGTATTATCAATGAGCCTACTGCTGCAGCCATTGCTTATGGTCTTGACAAGAAGGCTACCAGTGTCGGCGAGAAGAATGTCTTGATTTTTGATCTCGGGGGTGGTACTTTTGATGTCTCTTTACTTACCATTGAGGAGGGTATCTTTGAGGTGAAAGCCACAGCTGGGGACACCCATCTTGGAGGTGAGGATTTTGATAACAGGATGGTCAACCACTTTGTTCAGGAATTTAAGAGAAAGAACAAGAAAGATATCAGTGGCAACCCCAGAGCACTTAGGAGGTTGAGGACTGCCTGTGAGAGGGCAAAGAGAACTCTATCTTCAACTGCCCAAACCACCATTGAAATTGATTCTCTCTATGAGGGAATTGATTTCTACTCCACCATTACTCGTGCTAGATTTGAGGAGCTCAATATGGATCTCTTCCGCAAGTGCATGGAACCAGTTGAGAAGTGTTTAAGAGATGCTAAGATGGACAAGAGGACTGTCCATGATGTTGTACTTGTTGGTGGTTCCACCAGAATTCCCAAGGTGCAACAACTCCTGCAAGATTTCTTTAATGGGAAGGAGCTCTGCAGAGCATCAATCCTGATGAGGCTGTGGCATATGGTGCTGCAGTTCAAGCTGCTATCTTGA

>Glyma13g19331 ATGGCTGGCAAAGGAGAGGGTCCAGCCATCGGAATCGATCTCGGAACCACGTACTCGTGCGTCGGTGTCTGGCAACACGATCGCGTCGAGATCATCGCCAACGACCAAGGTAACAGAACGACGCCGTCTTACGTCGGGTTCACCGACACCGAGCGTCTCATTGGTGATGCCGCAAAAAATCAGGTCGCCATGAACCCCATCAACACCGTCTTTGATGCCAAGAGATTGATTGGTCGTAGATTCAGTGATGCCTCTGTTCAGAGTGATATTAAACTGTGGCCTTTTAAGGTTTTGTCTGGACCTGCTGAAAAGCCAATGATTCAGGTCAGTTACAAGGGTGAGGATAAGCAGTTTGCTGCAGAGGAAATCTCTTCTATGGTGCTAATGAAAATGCGTGAGATTGCTGAAGCTTATCTTGGCTCTTCAATCAAGAATGCCGTTGTTACTGTCCCTGCTTACTTTAACGATTCTCAGCGTCAAGCTACCAAGGATGCTGGTGTTATTGCTGGTCTCAATGTGATGCGTATTATCAATGAGCCTACTGCTGCAGCCATTGCTTATGGTCTTGACAAGAAGGCTACCAGTGTCGGCGAGAAGAATGTCTTGATTTTTGATCTCGGGGGTGGTACTTTTGATGTCTCTTTACTTACCATTGAGGAGGGTATCTTTGAGGTGAAAGCCACAGCTGGGGACACCCATCTTGGAGGTGAGGATTTTGATAACAGGATGGTCAACCACTTTGTTCAGGAATTTAAGAGAAAGAACAAGAAAGATATCAGTGGCAACCCCAGAGCACTTAGGAGGTTGAGGACTGCCTGTGAGAGGGCAAAGAGAACTCTATCTTCAACTGCCCAAACCACCATTGAAATTGATTCTCTCTATGAGGGAATTGATTTCTACTCCACCATTACTCGTGCTAGATTTGAGGAGCTCAATATGGATCTCTTCCGCAAGTGCATGGAACCAGTTGAGAAGTGTTTAAGAGATGCTAAGATGGACAAGAGGACTGTCCATGATGTTGTACTTGTTGGTGGTTCCACCAGAATTCCCAAGAGCATCAATCCTGATGAGGCTGTGGCATATGGTGCTGCAGTTCAAGCTGCTATCTTGAGTGGTGAGGGTAATGAGAAGGTTCAGGATCTTCTCCTTCTTGATGTCACCCCTCTCTCTCTTGGGTTGGAAACTGCTGGTGGGGTGATGACAGTCTTGATCCCCAGGAACACTACTATCCCAACAAAGAAAGAACAGGTTTTCTCAACCTATTCTGACAACCAGCCTGGTGTGTTGATCCAGGTTTATGAAGGTGAGAGAACAAGGACTAGAGATAACAACTTGTTGGGCAAATTTGAACTTTCTGGCATTCCTCCAGCTCCCAGGGGTGTTCCTCAAATCACAGTGTGCTTTGACATTGATGCCAATGGTATCTTGAATGTCTCTGCCGAAGACAAAACTACCGGCCAAAAGAACAAGATCACCATTACCAATGACAAGGGTAGGTTGTCAAAGGAAGAGATTGAGAAGATGGTTCAAGAGGCTGAGAAGTACAAATCAGAAGATGAAGAGCACAAGAAGAAGGTAGAGGCCAAGAATGCATTGGAGAACTACTCATACAACATGAGGAATACCATTAAAGACGAGAAGATTGGAGGAAAACTTGACCCAGCTGACAAGAAAAAGATTGAGGATGCCATTGAGCAGGCCATCCAGTGGCTAGACAGCAACCAGCTTGGAGAGGCAGATGAATTTGAGGACAAAATGAAGGAGTTGGAAAGCATCTGCAACCCTATCATTGCCAAGATGTACCAGGGTGGTGCTGGTCCAGACGTGGGTGGTGCAATGGACGATGATGTTCCTGCTGCTGGAAGCGGTGCGGGGCCAAAGATTGAGGAAGTAGACTAA

>Glyma13g29580 ATGGCACCCGGAAATGTCAAAGCTATAGGCATTGACTTGGGAACAACCTATAGCTGCGTGGCAGTGTGGCAACACAACCACGTCGAGGTCATTCCCAATGACCAAGGCAACCGCACAACCCCTTCCTACGTTGCCTTCACCGACACCCAAAGGTTGTTGGGTGACGCTGCCATCAACCAGCGATCCATGAACCCACAAAACACCGTCTTTGACGCCAAACGTTTGATAGGTCGCAGATTCTCCGACCAATCCGTCCAGCAAGACATGAAGCTCTGGCCTTTTAAGGTTGTCCCTGGAAACAGAGACAAGCCCATGATTTCTAGACAAGCCACCAAGGATGCCGGGAAAATCGCGGGTTTGAACGTGTTGAGGATCATCAACGAGCCAACCGCAGCTGCTATTGCTTATGGTTTGAACAAGAAAGGGTGGAGAGAAGGAATGTTTAAGGTTAAGGCCACGGTGGGAGATACCCATTTGGGAGGTGTTGACTTTGATAACAAAATGGTTGACTATCTTGTGAGTATTTTCAAGAGGAGGTACAAGAAGGACATTGGTGAAAATCCCAAAGCTCTTGGAAGGTTGAGGTCAGCGTGTGAGAAAGCTAAGAGGATACTCTCTTCAAGTTCTCAAACCACTATTGAGCTTGATTCTTTATGTGGGGGGGTTGATTTACATGCAAATTTTTCAAGGGCCTTGTTCGAGGAATTGAACAAGGACTTGTTCATGAAGTGTATGGAGACGGTGGAGAAGTGCCTCAAGGAGGCAAGGATTGCTAAAAGTCAAGTTCATGAGTTTGTTCTTGTAGGAGGGTCTACTAGAATTCCAAAGGTGCAGCAACTTTTGAAGGACATGTTCAGTGTTAATGGTAACAAGGAGCTTTGCAAAAGCATCAACCCTGATGAGGCCGTGGCGTATGGTGCGGCGGTTCAGGCGGCGATTTTGAGTGGCGAAGGAGACAAGAAGGTGGAGGATTTGTTGCTGCTGGATGTGATGCCGCTTAGTCTTGGAATTGAGACTGATGGTGGTGAGATGTCAGTGTTGATTCCCAAGAACACCATGATCCCCACCAAGAGGGAGAGTGTTTTCTCCACGTTTTCTGATAATCAAACAAGCGTTTTGATCAAAGTTTTCGAAGGGGAGCGAGCGAAGACAGAGGATAACTTCCTTCTTGGGAAGTTTGAGCTTTCTGGTTTCACTCCATCGCCAAGGGGAGTTCCACAGATCAATGTTGGGTTTGATGTTGATGTTGATGGCATTGTGGAAGTCACTGCTAGAGATAGGAGCACGGGGCTGAAGAAGAAGATCACGATCAGCAACAAGCATGGGAGGTTGAGTCCTGAAGAGATGAGGAGAATGGTGAGAGATGCAGTGAGGTATAAGGCAGAGGATGAGGAGGTGAGGAACAAGGTGAGGATAAAAAACTTGCTTGAGAATTATGCTTTTGAAATGAGGGACAGAGTGAAGAACCTTGAGAAGGTTGTGGAGGAGACCATAGAATGGCTTGACAGAAACCAATTGGCTGAAACTGATGAGTTTGAGTACAAGAGGCAGGAGTTGGAAGAAAAGGTTTTGAAGTTTATGTAA

>Glyma13g29590 ATGTTTCCTTGGTTACTATTTATGAAGGCATGTTTAAGGTTAAGGCCAGTGTTGGGAGACACTCATTTGGGTGGTGTGGATTTTGATAACAGATTGGTGAACCATCTTGTGAATGTGTTTAGAGAGAAGCACAAGAAGGATATTAGTGGGAATGCGAAAGCTTTGGCGAGGTTAAGGTCGGAGTGCGAGAAAGCAAAGAGGATTCTGTCTTCGACTTCTCAGACAACGATTGAGCTTGATTGTTTATACGAAGGGCTTGATCTGTATGCCCCTGTTACAAGGGCCTTGTTCAACGAACTGAACAAGGACTTGTTCATGAAGTGTATGGATACAGTGGAAAAGTGTCTCCTTGAGGCAAGGATTGATAAGATCCAAGTTCATGAGATTATTCTTGTTGGTGGGTCCACTAGAATTCCAAAGGTACAACAACTTCTGAAGGACATGTTCAGTGTCAATGGTAACACCAAAGAGCTTTGCAAAGGCATCAACCCTGATGAAGCTGTGGCGTACGGTGCTGCAGTTCAAGCAGCGATTTTGAGCGGTGAAGGAGATAAAAAAGTGGAGGAATTGTTGTTGCTGGATGTGATGCCACTTAGTCTTGGATTTGAGGGTGCTGGTGGTGTTATGTCAGTGTTGATCCCCAAGAACACCATGATCCCCACCAAGAAGGAGAGGATTTGTTCTACCTTCTATGACAATCAGAAATCTTTTAATGTCAAAGTGTTCGAGGGAGAACGAGTTAAGACAAAGGATAACTTCTTTCTCGGCAAGTTTGTCCTCAAAGGGTTCGATCCATTACCAAAGGGAGTGCCACAAATCAACGTTATCTTTGATGTGGATGCTGATGGCATTGTGGAGGTCACCGCAGAAGATAAAGCCACAGGAATAGAAAAGAAGATCACAATCAACAACAAGCACGGAAGGCTGAACCCGGAAGAGATAAGAAGAATGGTGAGAGACTCAAAGAAGTACAAGGCAGAGGATGAGTTGGCAAAGAAGAAGGTGAAGGCGAAGAACGCACTAGAGAATTACGCTTATGAGATGAGGGAGAGAGCAAAGAAGATTGAAGAGGCAGTGGAGGAAACCATAGAGTGGCTAGAGTGTAACCAATTGGCAGAGATAGGGGAGTTTGATTACAAGAAGCAGGAGCTAGGAAGGTGTCCACCTAATGGGTGGAACCATAGAAACGGGTGTAGTGGGAGTGCTTGTGGGGGTCTAGTTCGTGATTCCTCGGGATGTTACCTAGGTGGTTTCACAGTTAACTTAGGCAACACATCGGTTACTTTAGCGGAGCTATGGGGTGTTGTTCACGGTCTGAAGTTGGCGTGGGATCTTGGTTGCAAGAAGGTGAAGGTGGACATTGATTCTGGTAATGCTCTTGGTCTTGTTAGGCATGGCCCTGTGGCTAACGACCCTGCTTTCGCGTTGGTTTCGGAGATCAACGAGCTTGTTCGGAAAGAGTGGTTGGTAGAGTTCTCGCACGTGTTTAGAGAATCTAACCGTGCGGCTGATAAGTTGGCTCACTTGGGACACTCGCACTCTCTGGAATCGGGTGCGAAGCGATTCTCGGACCCACCCTCTGCCCTTGTTGCTATTCTTCAAGATGACTTGGCAGGGCTTGCCAAGCAACGTGGTGTTAATTAA

>Glyma13g29591 TTCCCCTGTGACTCACTCTCTTCCTTCACTGCTAAACTCTTCCAATGGCACAAAAAAGTCAAAGCCATAGGCATTGACTTGGGCACGACCTACAGCTTCTTTGCAGTGTGGCAACACAACCGCGTCGAGGTCATTTCCAACGACCAAGGCAATCGCACCACCCCTTCCTACGTTGCCTTCTCCGACACCCAAAGGTTGTTGGGTGACTCCGCCATGAACCAGCGATCCATGAATCCGAAAAACACCGTCTTTGACGCCAAACGTTTGATTGGTCGCCGATTCTCCGACCAAACCGTTCAGCAAGACATGAAGATGTGGCCTTTTAAGGTTGTCCCTGGAAACAAAGACAAGCCCATGATCGCGGTCACTTACAAAGGCGAAGAGAAACTCCTTGCCCCAGAAGAGATATCTTCCATGGTTTTGTATAAGATGAAGGAAGTTGCCGAAGGGTACTTGGGTCATTTCATAAAAGATGCGGTTATCACTGTCCCCGCTTACTTCAGCAACGCGCAGAGACAGGCCACGAAGGATGCGGGGAAAATCGCGGGTATGAACGTGTTGAGGATCATCAACGAGCCAACCGCTGCTGCTATTGCTTATGGGTTGGACAAGAAAGGATTGAGAGTTGGTGAGCAGAACGTGCTTGTGTTTGATCTCGGTGGGGGTACTTTTGATGTTTCCTTGGTTACTATTTATGAAGGCATGTTTAAGGTTAAGGCCAAGAAGCACAAGAAGGATATTAGTGGGAATGCGAAAGCTTTGGCGAGGTTAAGGTCGGAGTGCGAGAAAGCAAAGAGGATTCTGTCTTCGACTTCTCAGACAACGATTGAGCTTGATTGTTTATACGAAGGGCTTGATCTGTATGCCCCTGTTACAAGGGCCTTGTTCAACGAACTGAACAAGGACTTGTTCATGAAGTGTATGGATACAGTGGAAAAGTGTCTCCTTGAGGCAAGGATTGATAAGATCCAAGTTCATGAGATTATTCTTGTTGGTGGGTCCACTAGAATTCCAAAGGTACAACAACTTCTGAAGGACATGTTCAGTGTCAATGGTAACACCAAAGAGCTTTGCAAAGGCATCAACCCTGATGAAGCTGTGGCGTACGGTGCTGCAGTTCAAGCAGCGATTTTGAGCGGTGAAGGAGATAAAAAAGTGGAGGAATTGTTGTTGCTGGATGTGATGCCACTTAGTCTTGGATTTGAGGGTGCTGGTGGTGTTATGTCAGTGTTGATCCCCAAGAACACCATGATCCCCACCAAGAAGGAGAGGATTTGTTCTACCTTCTATGACAATCAGAAATCTTTTAATGTCAAAGTGTTCGAGGGAGAACGAGTTAAGACAAAGGATAACTTCTTTCTCGGCAAGTTTGTCCTCAAAGGGTTCGATCCATTACCAAAGGGAGTGCCACAAATCAACGTTATCTTTGATGTGGATGCTGATGGCATTGTGGAGGTCACCGCAGAAGATAAAGCCACAGGAATAGAAAAGAAGATCACAATCAACAACAAGCACGGAAGGCTGAACCCGGAAGAGATAAGAAGAATGGTGAGAGACTCAAAGAAGTACAAGGCAGAGGATGAGTTGGCAAAGAAGAAGGTGAAGGCGAAGAACGCACTAGAGAATTACGCTTATGAGATGAGGGAGAGAGCAAAGAAGATTGAAGAGGCAGTGGAGGAAACCATAGAGTGGCTAGAGTGTAACCAATTGGCAGAGATAGGGGAGTTTGATTACAAGAAGCAGGAGCTAGGAAGTGTGTACATAAAGTTTATCTGA

>Glyma13g32790 ATGGCCTCCTTGCTCCGCTCTCTCCGCCGCCGTGATGTCGCTTCCGCCTCCCTCTCCGCCTATCGCTCGTTAACGGGCAGCACCAAGCCAGCATATGTAGCTCACAACTGGTCTAGTTTGTCTCGACCATTCAGTTCAAGGCCTGCTGGAAACGATGTCATTGGTATTGATTTGGGTACTACCAATTCATGTGTTTCCGTTATGGAAGGAAAGAACCCCAAAGTCATTGAGAATTCTGAAGGTGCACGAACAACACCATCTGTGGTTGCTTTCAACCAGAAAGGGGAGCTGCTTGTAGGTACCCCAGCTAAGCGTCAAGCTGTAACTAACCCAACAAACACTCTCTTTGGTACCAAGCGGTTGATTGGTAGGCGCTTTGATGACGCTCAAACACAAAAGGAGATGAAAATGGTTCCATTCAAGATTGTTAAGGCTCCAAATGGAGATGCGTGGGTGGAAGCCAATGGGCAGCAGTATTCCCCTAGTCAAATTGGTGCCTTTGTTCTCACCAAGATGAAGGAAACTGCAGAAGCTTATCTAGGAAAGTCAATTTCTAAGGCTGTAATTACTGTACCAGCTTACTTCAATGATGCTCAGAGGCAGGCAACAAAAGATGCTGGTAGAATTGCAGGTCTTGACGTGCAGAGAATTATCAATGAGCCCACTGCTGCTGCACTTTCATATGGGATGAACAACAAGGAGGGTCTCATTGCCGTTTTTGACCTTGGAGGTGGAACATTTGATGTGTCCATCTTAGAAATTTCTAATGGTGTTTTTGAGGTGAAAGCAACAAATGGTGACACTTTCTTGGGAGGAGAGGATTTTGACAATGCCTTATTGGATTTTCTGGTGAATGAATTCAAAAGAACCGAGAGTATCGACCTTTCAAAGGACAGGCTTGCACTGCAGAGGCTTCGTGAAGCTGCTGAGAAAGCTAAGATCGAGCTGTCTTCAACATCTCAAACTGAGATCAACCTGCCTTTCATCACTGCTGATGCATCTGGTGCTAAGCATCTGAACATAACATTGACTAGATCGAAGTTTGAGGCTTTGGTGAATCACTTGATTGAAAGGACCAAGGTACCATGTAAAAGCTGCTTGAAGGATGCTAACATCTCTATCAAGGATGTTGATGAGGTTCTTCTAGTTGGAGGGATGACTCGTGTTCCTAAAGTCCAAGAGGTGGTTTCAGAGATCTTTGGAAAGTCTCCTAGTAAAGGAGTAAACCCTGATGAGGCAGTTGCCATGGGGGCGGCAATCCAAGGTGGTATTCTACGGGGAGATGTTAAAGAGCTACTACTCCTAGATGTAACACCACTCTCTCTGGGTATTGAGACTTTGGGTGGTATCTTTACAAGATTGATCAACCGCAACACTACTATTCCTACAAAGAAGAGTCAGGTCTTTTCAACAGCAGCTGACAATCAAACTCAGGTAGGTATCAAGGTGCTACAAGGTGAGCGGGAAATGGCTGCAGACAACAAAATGCTTGGAGAGTTTGACCTTGTTGGTATTCCTCCTGCTCCCAGAGGTCTGCCTCAGATTGAGGTCACATTTGACATTGATGCTAATGGGATTGTTACTGTCTCTGCCAAAGACAAGTCCACTGGTAAAGAACAACAAATAACTATTCGGTCATCCGGTGGACTCTCGGATGATGAGATTGAAAAGATGGTCAAAGAAGCAGAATTGCATGCTCAGAAAGACCAAGAGAGAAAGGCTCTCATTGACATTAGAAACAGTGCTGACACTACCATCTATAGCATTGAGAAGAGTTTAGGTGAATACAGAGAGAAGATTCCCAGTGAAGTGGCCAAAGAAATTGAGGATGCAGTTTCGGATTTGAGACAGGCGATGTCAGGGGATAATGTTGATGAAATCAAGTCAAAGCTTGATGCTGCAAACAAAGCTGTGTCCAAGATTGGAGAGCACATGTCAGGTGGTTCTAGTGGCGGTTCCTCAGCTGGTGGTTCTCAGGGTGGGGACCAGGCTCCTGAGGCAGAATATGAGGAGGTGAAGAAGTAA

>Glyma13g43630 ATGAGCGTGGTTGGATTTGATTTTGGTAATGAGAGCTGCATTGTTGCTGTCGCAAGACAAAGGGGGATTGACGTTGTGCTCAACGATGAGTCAAAGCGTGAAACACCTGCAATTGTATGCTTTGGTGACAAACAGCGGTTCCTTGGGACAGCCGGGGCTGCTTCTACTATGATGAACCCAAAGAATTCCATCTCACAGATAAAGAGGTTAATTGGCAGACAATTTGCGGATCCGGAATTGCAGCAAGATATCAAGACGTTCCCCTTTGTGGTCACTGAAGGACCTGATGGATATCCTTTAATTCATGCACGGTACTTGGGTGAATCTAGAACATTTACACCTACCCAAGTATTTGGAATGATGTTATCAAACCTTAAAGAAATAGCTGAGAAAAATTTGAATGCTGCTGTTGTTGATTGCTGCATTGGAATTCCACTTTATTTTACCGATCTTCAAAGGAGGGCAGTCTTGGATGCTGCCACTATTGCAGGTTTGCACCCACTTCGTCTGTTTCATGAAACAACAGCAACTGCATTGGCTTATGGAATTTATAAGACTGATCTTCCTGAAAATGATCAGCTGAATGTTGCATTTGTTGATGTTGGACATGCTAGCATGCAAGTATGCATTGCTGGATTTAAAAAGGGGCAGCTGAAAGTGTTGTCCCAATCATATGATAGGTCCCTAGGTGGTAGGGACTTTGACGAGGTTCTGTTCAACCATTTTGCTGCAAAGTTTAAGGAGGAGTACAAGATTGATGTATTCCAAAATGCCAGGGCTTGTCTGAGGTTGAGGGCTGCCTGCGAGAAGCTGAAGAAGGTGCTTAGTGCTAATCCGGAGGCCCCTTTGAACATTGAGTGCTTAATGGATGAGAAGGATGTCAGAGGCTTTATCAAGCGTGATGAGTTTGAGCAACTAAGTCTTCCAATTTTGGAACGGGTGAAGGGACCTTTGGAGAAGGCACTTGCAGAGGCAGGTCTTACAGTCGAAAATGTGCACATGGTTGAGGTGGTTGGTTCTGGATCTCGTGTACCAGCTATAAACAAAATATTGACAGAGTTCTTCAAAAAGGAGCCTAGGCGGACAATGAATGCTAGTGAGTGTGTTGCCAGGGGTTGTGCATTGCAGTGTGCAATTCTCAGTCCAACTTTTAAAGTACGGGAGTTTCAGGTCAACGAAAGCTTTCCTTTCTCAATTTCTCTCTCGTGGAAAGCTCCAAGTTCTGATGCACAGGAAAGTGGACCAGATAATAAACAAAGTACCCTTGTTTTCCCCAAGGGAAATCCCATACCAAGTGTCAAGGCACTGACCATCTACAGGTCAGGAACCTTTTCTATTGATGTTCAGTATGATGACGTGAGTGGTTTGCAAACACCTGCAAAGATCAGCACATATACTATTGGCCCTTTCCAATCTACAAAAAATGAAAAAGCAAAAGTTAAAGTGAAAGTTCGGTTGAATGTGCATGGAATTATATCTGTCGAATCTGCAACTCTACTGGAAGAGGAAGAAGAAATTGAGGTTCCAGTTTACAAAGAACCTGCAGGAGAAAATTCCAAGATGGAAACTGATGAAGCTCCTGCTGATGCTGCTGCTGCTGCTGCAACTCCTAGCACCAATGACAATGATGTTAGCATGCAAGATGCTAATACCAAGGCAACTGCTAATGCCCCTGGAGCCGAAAATGGTACCCCTGAGGCAGGAGATAAGCCTGTGCAGATGGATACTGATACCAAGGTTGAGGCTCCAAAGAAGAAAGTTAAGAAAATAAACATTCCTGTGGTAGAGTTAGTTTATGGAGCAATGGCAGCCACAGATGTCCAGAAAGCTGTAGAGAAGGAGTTTGAAATGGCTTTGCAAGATAGAGTCATGGAGGAAACGAAAGATAAGAAAAATGCAGTTGAGGCTTATGTTTATGACATGAGAAACAAGCTTAATGACAAATACCAAGAGTTTGTCATTGATTCAGAGAGGGAAGCATTTACTGCTAAACTTCAGGAGGTGGAAGACTGGCTATATGAGGATGGTGAAGATGAAACTAAAGGCGTATACATTGCCAAGCTAGAAGAGCTCAAGAAGCAAGGTGATCCAATTGAAGAGCGTTACAAGGAATACATGGAGAGGGGTACAGTAATTGATCAACTTGCCTATTGTATAAATAGTTATAGAGAAGCTGCAATGTCAAATGATCCCAAGTTTGATCACATTGACATCAATGAGAAACAAAAGGTCTTAAATGAATGTGTGGAAGCTGAGAACTGGCTCAGGGAGAAAAAACAGCATCAGGACTCGCTTCCAAAATATGCCACCCCTGTACTTTTGTCAGCTGACGTAAGAAAGAAAGCTGAAGCTGTGGACAGGTTTTGTAAGCCAATTATGACGAAACCAAAGCCACTGCCACCCAAGCCAGCTACACCAGAAGCACCAGCAACCCCACCTCCTCAGGGTGGTGAGCAGCAGCAACAACCACCTCAGGAGAATCCTAATGCCAGTACTAATGAAAATGCTGGGGACAATGCTAATCCAGCCCCGCCACCAGCATCTGCTGAACCAATGGAGACTGACAAACCAGAGAACACAGGCTCTGCTTAA

>Glyma14g02740 ATGAGTGGAGTGGGGATTGATATTGGAAATGAGAACTGTGTGATTGCTGCAGTGAAGCAACGTGTGATTGATGTTTTGTTGAATGATGAATCCAAACGTGAAACCCCTGGTGTGGTCTGCTTTGGAGAGAAGCAACGGTTTATAGGGTCAGCTGGTGCTGTTTCTGCTATGATGCACCCCAAGTCCACAATATCTCAAGTGAAGAGACTAATAGGCAGGAGATTTACGGACCCTGATGTTCAGAATGATTTAAAACTGCTCCCAGTTGAAACTTCGGAAGGCCCGGATGGTGGCATTCTGATTCGCTTGAAATACTTGAAGGAGATTCATGCATTTACCCCGGTTCAAATAGTAGCAATGCTCTTTGCTCACTTGAAGACTATAGCTGAAAAGGATTTTGGGACCGCAGTTTCTGACTGCGTTATTGGGGTTCCGTCATACTTTACCAACTTGCAGAGACAAGCATATCTCGATGCAGCAGCAATTGTTGGGTTGAAGCCTTTGCGGTTGATCCATGATTGCACTGCAACTGGTCTTAGTTATGGAGTTTACAAAACAGATATTCCCAATGCAGCTCATATTTATGTTGCATTTGTTGACATAGGTCATTGTGATACTCAGGTCTCTATTGCAGCATTTCAGGCTGGTCAAATGAAGATACTTTCACATGCTTTTGACAGTAGCTTAGGGGGGAGAGACTTTGATGAGGTTCTGTTTAGTCATTTTGCAGCAAGATTTAAGGAACAGTATAGCATTGACGTGTATTCTAATGGCAGGGCATGTAGGAGGCTGCGTGTAGCATGTGAGAAGTTGAAGAAGGTTTTGAGTGCAAATGCAGTGGCTGATCTGAGCATTGAGTGTTTGATGGATGAAAAAGATGTTAAGGGCTTTATCAAGAGGGAAGAATTTGAGAATTTGGCATCAGGATTACTGGAGAAATTTAATATTCCCTGCAACAAAGCATTGGCTGATGCCGGCATGACTGTAGAAAAGATTAATTCTGTTGAGTTAGTTGGTTCAGGTTCACGGATTCCAGCTATAACTAATTTATTAACTTCTCTATTTAAGAGAGAACTCAGCCGCACGCTGAATGCAAGTGAGTGTGTAGCACGTGGTTGTGCTCTCCAGTGTGCAATGCTCAGTCCTATTTTCCGTGTCAAAGAATATGAGGTCCAGGATTCTATTCCTTTTTCCATTGGACTTTCATGTGATGGAAGTCCAATTTGTGAAGGATCGGATGGTGTACTCTTCCCAAAAGGCCAACCCATTCCAAGTGTTAAAATTCTAACATTTCAGTGCAGTAATTTGCTCCATCTGGAAGCATTCTATGCTAATCCAGATGAATTACCACCTGGGACATCTCCTAAAATAAGTTGCTTCACAATTGATCCTTTCCATGGATCCCATGGAAGTAAGGCAAGAATTAAAGTTCGAGTTCAACTAAATTTGCATGGCATTATCAGTATTGAATCAGCTACATTGATGGAGGATCATGTGGATGATTCAGTTACAACAGGTGATTATCATTCAAATTCTGAAGCAATGAATGTTGAACCTGTTTCTGAGACAGTTGAAAATGTCACAGAAGATAGTATCAACAAAAAGTGTGAAGCTCCACGCCATTTGGCTGATGGTACAAAAAAAGATAAAGCTAACAGAAGGCTTCATGTACCTGTGAGTGAAAACATTTATGGTGGAATGACCAAGGCTGAGATATTGGAAGCTCAAGAAAAAGAACTCCAGTTAGCTGATCAGGACAGAACAATTGAGCTGACCAAAGACAGGAAGAATTCGTTGGAGTCTTATATTTACGAAACGAGGAGTAAGCTATTCAGCACATATCTAAGCTTTTCAAGCGAACATGAGAGGAAGGACATATCTAGGAGCCTGAAAGCGACTGAGGATTGGCTTTATGATGATGGTGATGACGAAACTGTAGATGCTTATTCTGCAAAACTAGAAGATCTAAAACAGCTGGTGGATCCAATTGAGTTTCGGTATAAAGACACAGAAGCAAGACCACAAGCTACAAGAGATTTGTTAAGTTGCATTGTAGAGTATCGAATGTCTGCAGATTCTCTTCCACCCCAAGATAAAGAACAGATCATCAATGAGTGCAATAAAGCAGAGCAGTGGCTAAGAGAGATGAGGCAACAGCAAGATCTTTACCCTAAGAACTTTGATCCAGTATTATTGTCAAGTGATATCAAGAGCAAGACAGAAGATTTAAACTCAGTATGCCAACAGATATTGAAATCCAAGGGTTCTCCATTTCCAAAAGACAAGGGCGAAGACAAGCAGAATACTTCTAATCATCAATGA

>Glyma15g01750 ATGAGCGTGGTTGGATTTGATTTTGGTAATGAGAGCTGCATTGTTGCTGTCGCAAGACAAAGGGGGATCGACGTTGTGCTCAATGATGAGTCAAAGCGTGAAACACCTGCAATTGTATGCTTTGGTGACAAACAGCGGTTCCTTGGGACAGCTGGGGCTGCTTCTACAATGATGAACCCTAAGAATTCCATCTCTCAGATAAAGAGGTTAATTGGTAGACAGTTTTCTGATCCAGAATTGCAGCGAGACCTGAAGACATTCCCTTTTGTTGTCACTGAAGGACCTGATGGATATCCATTAATTCATGCACGGTACTTGGGTGAAGCTAGAACATTTACACCCACCCAAGTATTTGGAATGATGTTATCAAACCTTAAAGAAATAGCCGAGAAAAATCTGAATGCTGCCGTTGTTGATTGCTGCATTGGAATTCCACTTTATTTTACTGATCTTCAGAGGAGGGCGGTCTTGGATGCTGCTACTATTGCTGGTTTGCATCCACTTCGTCTGTTTCATGAAACAACTGCAACTGCATTGGCTTATGGAATTTATAAGACCGATCTTCCTGAAAATGATCAACTGAATGTTGCCTTTGTTGATGTTGGACATGCTAGCATGCAAGTATGCATTGCTGGATTTAAAAAGGGGCAGCTGAAAGTGTTGTCCCAATCATATGATAGGTCCCTAGGTGGGAGGGACTTTGACGAGGTTCTGTTCAATCATTTTGCTGCAAAGTTTAAGGAGGAGTACAAGATTGATGTATTCCAAAATGCCAGGGCTTGTCTGAGGTTGAGGGCTGCTTGCGAGAAGCTGAAGAAGGTGCTTAGTGCTAATCCCGAGGCACCTTTGAACATTGAGTGCTTAATGGATGAGAAGGATGTCAGAGGCTTTATCAAGCGCGATGAGTTTGAGCAACTGAGTCTTCCAATTTTGGAACGGGTGAAGGGACCTTTGGAGAAGGCACTTGCAGAGGCAGGTCTTACAGTTGAGAATGTACACATGGTTGAGGTGGTTGGTTCTGGATCTCGTGTGCCAGCTATAAACAAAATTTTGACAGAGTTCTTTAAAAAGGAGCCTAGGCGGACAATGAATGCTAGTGAGTGTGTTGCCAGGGGCTGTGCATTGCAGTGTGCAATTCTCAGCCCAACTTTTAAAGTACGGGAGTTTCAGGTCAATGAAAGCTTTCCTTTCTCAATTTCTCTCTCGTGGAAAGGTCCAAGTTCTGATGCACAGGAAAGTGGACCAAATAATACGCAGAGAACCCTTGTTTTCCCCAAGGGAAATCCCATACCAAGTGTCAAGGCACTAACCATCTACAGGTCAGGAACCTTTTCTATTGATGTTCAATATGATGATGTGAGTGAATTGCAAACACCTGCAAAGATCAGCACATATACTATTGGACCTTTCCAATCTACAATAACTGAAAAAGCAAAAGTTAAAGTGAAAGTTCGGTTGAATCTTCATGGAATTGTATCTGTCGAATCTGCAACTCTGCTGGAAGAGGAAGAAATTGAGGTTCCAGTTTCCAAAGAACCAGCAGGAGAAAATACCAAGATGGAAACTGATGAAGCTCCTGCCAATGTTGCTGCACCTCCTAGCACCAATGACAATGATGTTAACATGCAAGATGCTAATTCCAAGGCAACTGCTGATGCCCCTGGGTCCGAAAATGGGACCCCTGAGGCAGGAGATAAGCCTGTGCAAATGGATACTGATACCAAGGTTGAGGCTCCAAAGAAAAAAGTTAAGAAAATAAACATTCCTGTGGTGGAGTTAGTTTATGGGGCAATGGCAGCTGCAGATGTCCAGAAAGCTGTAGAGAAGGAGTTTGAAATGGCTTTGCAAGATAGAGTGATGGAGGAAACAAAGGATAAGAAAAATGCAGTAGAGGCTTATGTTTATGACACGAGAAACAAGCTTAATGACAAATACCAAGAGTTTGTCGTTGATTCAGAGAGGGAATCATTTACTGCTAAACTTCAGGAGGTAGAAGACTGGCTATATGAGGATGGTGAAGATGAAACTAAAGGTGTATACATTGCCAAGCTAGAAGAACTCAAGAAGCAAGGTGATCCAATTGAAGAGCGTTACAAAGAATACATGGAGAGGGGTACAGTAATAGATCAACTTGTCTATTGTATAAATAGTTATAGAGAAGCTGCAATGTCAAATGATCCCAAATTCGATCACATTGACATCAATGAGAAACAAAAGGTCTTAAATGAATGTGTGGAAGCTGAGAACTGGCTCAGGGAGAAAAAACAGCAACAGGACTCTCTTCCGAAATATGTCACCCCGGTACTTTTGTCAGCTGACATAAGAAAGAAAGCTGAAGCTGTTGACAGGTTCTGTAAGCCAATTATGATGAAACCAAAGCCACCACCACCCAAGCCAGCTACACCAGAAGCACCAGCAACCCCACCTCCTCAGGGTGGTGAGCAGCCACAGCAGCAGCAACAACAACCACCTGAGGAGAATCCTAATGCCAGTACTAATGAAAAGGCAGGGGACAATGCTAATCCAGCCCCACCACCAGCATCTGCTGAACCAATGGAGACTGACAAACCAGAGAACACAGGCTCTGCTTAA

>Glyma15g06530 ATGGCCTCCTTGCTCCGCTCTCTCCGCCGCCGCGATGTCGCCTCCGCCTCCCTCTCTGCTTATCGCTCGTTAACGGGCAGCACCAAGCCAGCATATGTAGCTCACAACTGGTCTAGTTTGTCTCGACCATTCAGTTCAAGGCCTGCTGGAAACGATGTCATTGGTATTGATTTGGGTACAACCAATTCATGTGTTTCCGTTATGGAAGGAAAGAACCCCAAAGTTATTGAGAATTCTGAAGGTGCACGAACAACACCATCTGTGGTTGCTTTCAACCAGAAAGGGGAGCTGCTTGTAGGTACCCCAGCTAAGCGTCAAGCTGTAACTAACCCAACAAACACTCTCTTTGGTACCAAGCGGTTGATTGGTAGGCGCTTTGATGATGCTCAAACACAAAAGGAGATGAAAATGGTTCCATTCAAGATTGTTAAGGCTCCAAATGGAGATGCTTGGGTGGAAGCTAATGGGCAGCAGTATTCCCCTAGCCAAATTGGTGCCTTTGTTCTCACCAAGATGAAGGAAACTGCAGAAGCTTATCTAGGGAAGTCAATTTCTAAGGCTGTAATTACTGTACCAGCTTACTTCAACGATGCTCAGAGGCAGGCAACAAAAGATGCTGGTAGAATTGCAGGTCTTGACGTGCAGAGAATTATCAATGAGCCCACTGCTGCTGCACTTTCATATGGGATGAACAAGAAGGAGGGTCTCATTGCCGTTTTTGACCTTGGTGGTGGAACATTTGATGTGTCCATCTTAGAAATTTCTAATGGTGTTTTTGAGGTGAAAGCAACAAATGGTGACACTTTCTTGGGAGGAGAGGATTTTGACAATGCCTTGTTGGATTTTCTGGTGAATGAATTCAAAAGAACTGAGAGTATTGACCTTGCAAAGGACAGGCTTGCACTGCAGAGGCTTCGTGAAGCTGCTGAGAAAGCTAAGATCGAGCTGTCTTCAACATCTCAAACTGAGATCAACCTGCCTTTCATCACTGCTGATGCATCTGGTGCAAAGCATCTGAACATAACATTGACAAGATCGAAGTTTGAGGCTTTGGTGAATCACTTGATTGAAAGGACCAAGGCACCATGTAAAAGCTGCTTGAAGGATGCTAACATCTCTATCAAGGATGTTGATGAGGTTCTTCTTGTTGGAGGGATGACTCGAGTTCCTAAAGTCCAAGAGGTGGTTTCAGAGATCTTTGGAAAGTCTCCTAGCAAAGGAGTAAACCCTGATGAGGCAGTTGCCATGGGGGCAGCAATCCAAGGTGGTATTCTACGGGGGGATGTTAAAGAGCTACTACTCCTAGATGTAACACCACTCTCTCTCGGTATTGAGACTTTGGGTGGTATCTTTACAAGATTGATCAACCGCAACACTACTATTCCTACTAAGAAGAGTCAGGTCTTTTCAACGGCAGCTGACAATCAAACTCAGGTAGGTATCAAGGTGCTACAAGGCGAGCGGGAAATGGCTGCAGACAACAAAATGCTTGGAGAATTTGACCTTGTTGGTATTCCTCCTGCTCCCAGAGGTCTGCCTCAGATTGAGGTTACATTTGACATTGATGCCAATGGGATTGTTACTGTCTCTGCAAAAGACAAGTCCACTGGTAAAGAACAACAAATCACTATTCGGTCTTCCGGTGGACTCTCGGAAGATGAGATTGAAAAGATGGTCAAAGAAGCAGAGTTGCATGCTCAGAAAGACCAAGAGAGAAAGGCTCTCATTGACATTAGAAACAGTGCTGACACCACCATATATAGCATTGAGAAGAGTTTAGGTGAATACAGAGACAAGATTCCCAGTGAAGTGGCCAAAGAAATTGAGGATGCAGTTTCGGATCTGAGGAAGGCGATGTCAGAGGATAATGTTGATGAAATCAAGTCAAAGCTTGATGCTGCAAACAAAGCTGTATCCAAGATTGGAGAGCACATGTCAGGAGGTTCTAGTGGCGGTTCCTCAGCTGGTGGTTCTCAGGGTGGGGACCAGGCTCCCGAGGCAGAATACGAGGAGGTGAAGAAGTAA

>Glyma15g09420 ATGGCAACAAAAAAAGTCAAAGCTATAGGCATTGACTTGGGCACCAGCTACAGCTGTGTGGCTGTGTGGCAACACAACCGCATCGAGGTCATTTCCAACGACCAAGGCAACTGCACCACCCCTTCCTACGTTGCCTTCAACGACAACCAAAGGTTGTTGGGGGACTCCTCCATGAGCCAAAGATCCATGAATCCGCAAAATACCGTCTTTGACGACAAACAAACTTACTATTTACATCGGCCTTTTAAGGTTGTCCCTGACAATAGAGACAAGCCCATGGTCACGGTCACTTACAAAGGTGAGGAGAAACTCCTTGCCCCCGAAGAGATATCTTCCATGGTGCTGTTTAAGATGAAGGAAGTTGTCGAAGCCCATTTGGGTCATTTCGTAAAGGATGCAGTGATCACTGTCCCTGCTTACTTCAGCAACGCGCAGAGACAGGCCACTAAGGATGTCGGGAAAATCGCGGGTTTGAACGTGTTGAGGATCATCAGCGAACCAACTGCGGCTGCTATTGCTTACGGGTTGGACAGAAAAGGATTGAGAGTGGGTGAGCAGAACGTGCTTGTGTTTGATCTCGGTGGTGGTACTTTTGATGTTTCCTTGGTGACTATTTATGAAGGGATGTTTAAGGTTAAGGCTAGTGTGGGAGATACTCATTTGGGTGGTGTGGATTTTGATAACAAATTGGTGAACCATCTGGTGAATGTGTTTAGAGAGAAGCACAAGAAGGATATTAGCGGGAATGCGGAAGCTTTGGTGAGGTTGAGGTCAGCGTGCGAGAAAGCAAAGAGGATTCTGTCTTCGACTGCTCAGACAACGATTGAGCTTGATTGTTTATATGAAGGGGTTGATCTGTATGCCACTGTGACAAGGGCCTTGTTCGAGGAACTGAACAAGGACTTGTTTATGAAGTGTATGGAGACGGTGGAGAAGTGTCTCCTTGAGGCAAGGAGTGATAAGATTCAAGTCCATGAGATCGTTCTTGTTGGTGGGTCTACTAGAATTCCAAAGGTACAACAACTTCTGAAGGACATGTTCAGTCTCAATGGTACCACCAAAGAGCTTTGCAAAGGCATCAACCCTGATGAAGCTGTGGCGTATGGTGCAGCAGTTCAAGCAGCGATTTTGAGCGGTGAAGGAGATAAAAAAGTGGAGGAATTGTTGTTGCTGGATGTGATGCCAATTAGTATTGGATTCGAGGGTGCTGGTGGTGTGATGTCAGTGTTGATCCCCAAGAACACCGCGATCCCCACCAAGAAGGAGAGGGTTTGTTCCATCTTCTACGACAATCAGAAATCTCTTACAGTCAAAGTGTTCGAGGGAGAACAAGTTAAGACAAAGGATAACTTCTTTCTCGGCAAGTTTATCCTCTACAGGTTCGATCCATTACCAAAGGGAGTGTCACAAATCAGCGTTATCTTTGATGTGGATGCTGATGGCATTGTGGAGGTAACCGCGGAAGATCAAGCCAAAGGGTTAAAAAAGAAGATCACAATCAATAGCAAGCATGGAAGGCTAAGCCCGGAAGAGATAAGAAGAATGGTGAGAGATTCAAAGAGGTACAAGGCAGAGGATGAGGTGGCAAAGAAGAAGGTGAAGGCGAAGAACACACTCGAGAATTACGCTTATGAAATGAGGGAGAGAGCAAAGAAGATTGAAGAGGCAGTTGAGGAAACCATAGAGTGGCTAGAGTGTAACCAATTGGCAGAGATAGAGGAGTTTGATTGCAAGAAGCAAGAGCTAGGA

>Glyma15g09430 ATGGCACCCAGAAAAGTCAAAGCCATGGGCATTGACTTGGGCACCACCTACAGCTGCGTGGCTGTGTGGAACCATAACCGCGTCGAGGTCATTCCCAACGACCAAGGCAACCGCACCACCCCTTCCTACGTTGCCTTCACCGACACTCAAAGGTTGTTGGGCGACGCTGCCATCAACCAGCGATCCATGAATCCTCAAAACACCGTCTTCGACGCCAAACGTTTGGTCGGTCGCAGATTCTCCGACCAGTCCGTACAGCAAGACATAAAGTTGTGGCCTTTTAAGGTTGTCCCTGGAGCCAGAGACAAGCCCATGATTGCTGTAACATACAAAGACGAAGAGAAACTCCTTGCAGCCGAAGAGATATCTTCCATGGTGCTGTTTAAGATGAAGGAGGTTGCCGAAGCCCATTTGGGTCATTTCGTAAAGGATGCAGTGATCACTGTCCCTGCTTACTTCAGCAACGCGCAGAGACAGGCCACTAAGGATGCCGGGAAAATCGCGGGTTTGAACGTGTTGAGGATCATCAACGAGCCAACCGCGGCTGCTATTGCTTACGGGTTGGACAAGAAAGGGTGGAGAGAAGGTGAGCAGAACGTGCTTGTGTTTGACCTCGGTGGTGGTACTTTTGATGTTTCCCTGGTTACAATTGATGAAGGGATGTTTAAGGTTAAAGCCACGGTGGGAGATACCCATTTGGGAGGTGTTGACTTTGACAACAAATTGGTCAACTATCTCGTGGGTATTTTCAAGAGGAGGTACAAGAAGGACATTGGTGAAAACCCCAAAGCTCTTGGAAGGTTGAGGTCAGCGTGTGAGAAAGCTAAGAGGATTCTCTCTTCAAGTTCCCAAACCACTATTGAGCTTGATTCTTTATGTGGAGGGGCTGATCTACATGCAATTGTTACAAGGGCCTTGTTCGAGGAACTGAACAAGGACTTGTTTATGAAGTCCCAAGTTCATGAGCTTGTTCTGGTAGGAGGGTCTACTAGAATTCCAAAGGTGCAGCAACTTTTGAAGGACATGTTCAGTGTTAATGGTAACAAGGAACTTTGTAAAAGCATAAACCCTGATGAGGCCGTGGCGTATGGTGCGGCGGTTCAGGCCGCCATTTTGAGTGGCGAAGGAGACAAGAAGGTGGAGGAGTTGTTGCTGCTGGATGTGATGCCGCTTAGTCTTGGGATTGAGACTGATGCTGGTGAAATGTCAGTGTTGATTCCCAAGAACACCATGATCCCCACCAAGAGGGAGAGCGTCTTCTCCACTTTTTCTGATAATCAAACAAGTGTTTTGATCAAAGTGTTCGAGGGGGAGCATGCAAAGACAGAGGATAACTTCCTTCTTGGGAAGTTTGAGCTTTCTGGTTTCACTCCATCGCCAAGGGGAGTTCCACAAATCAATGTGGGGTTTGATGTTGGTGTTGATGGCATTGTGGAAGTCACTGCTAGAGATAGGAGCACGGGGCTGAAGAAGAAGATCACGATCAGCAACAAGCATGGGAGGTTGAGTCCTGAAGAGATGAGGAGGATGGTGAGAGATGCAGAGAAGTATAAGGCAGAGGATGAGGAGGTGAGTAACAAGGTGAGGGCCAAGAACTTGCTTGAGAATTATGCCTTTGAAATGAGGGACAGAGTGAAGAACCTTGAGAAGGTTGTGGAGGAGACCATAGAGTGGCTTGACAGAAACCAATTGGCTGAAACTGATGAGTTTGAGTACAAGAAGCAGGAGTTGGAAGAAAAGTTTCGGAAGTTTAGGTAA

>Glyma15g10280 TCATGTGTTGGAGTGTGGCTGGAGCAACACAATAGAGTAGAAATAATTCACAACCAACAAGGCCACAAAACCACACCTTCTTTTGTTGCTTTCACAGACAATCAGAGATTGATTGGTGATGCTGCTAAGAATCAAGCTGTCACCAACCCAGAAAACACTGTTTTTGATGCAAAGAGATTAATTGGCAGGAAATACAGTGATCCCATTATTCAAAAAGAGAAAACGTTGTGGTCATTCAAGGTTGTTGCCGGTATCAATGACAAACCCATGATTGTTGTTAAGTACAAGGGTCAGGAGAAGCAAATTTATGCTGGTGCTATTGCTGGCCTAAATGTTATGAGTATAATTAATGAACCTACTGCAACAGATATAGCATATGGTCTTAACAAGAGAACTAATTGTGTTGGAGAGCGAAACATTTTCATTTTTGACCTTGGTGGTGGTACTTTAGACGCTGCTCTCCTCACGATTAAGGACGTCTACGAAGTTAAGGCTACTGCCGGAAAAAACGACTTCAAAAAGAAGAACAAAGTGGACATTAGTGGGAACCCAAGAGCACTAAGGAGGTTGAGGACTTCATGTGAGAGGGCCAAAAGAATACTACCAACATTGAGAAAGTTTGAGGAAATCGATATGGAGCTGTTTGAAGAATGCATGGAGACAGTAGATAAGTGTCTTACTGATTCTAAGATGGGCAAGGGCAGTGTCCGTGATGTTGTCCTTGTTGGTGGTTCTTCTAGGATTTCCAAAGTGCAAGAGCTATTGCAGGACCTCTTCGATGGAAAGGATTTGTGCAAGAGCATCAACCCTGACGAGGCTGTTCCTTATGGCGCATCTGTGCAGGCTGCTATGTTGAGTGAAGGCATTAAGAATGTTCCAGACTTGGTTCTATTGGGTGTTACACCACTGTCACTTGGTATATTGACCAAGGGAGATGTCATGAGTGTGGTGATTCCAAGGAATACTAGGATTCCTGTAAGGAAGACGCAAGTATGCTGTAATTTAGATAACCAAAAACGTGTTCCTTTTAGTGTTTACGAGGGTGAAAGGGCGAGAGCCAATGATAATAATTTGTTGGGTTCTTTTGTTCTTTCTGGTTTGCCCCCTTCTCCTCGTGGTCATCCTTTAGATGTAAGTTTTGCTATAGATGTAAATGGTATCCTATCTGTTTCCACCGAGGAGAAAACTAGTGGCAATAAGAATGAGATTACCATAATCAATGACAAAGATAGACTGTCAACTGAAGAAATTGGAAGATTGATTCAAGAAGCTGAGAAGTACAGGGCTGAAGATAAGAAGTTTCTTAGGAAGGCCAATGCTATGAATTCTTTGGGTTATTATGTTTACAAGATGAGGAATGTTTTAAAGAAGGATATTAGTTCGCTTTGCTCAAAAGAAAGGGAGAAGATCGATTATGCCATTACTAAGGCCACAAATTTGCTAGATGATAGTAAATACCAGTATGAAGTGGAGGTGTTTGAGGATCATCACAAGGAGCTTGCCAGCTTCTTTGAATCCATCGCGAGCAAGATTGGTTAG

>Glyma16g00410 ATGGCTTGCTCAAGCGCCCAAATACACGGCCTCGGAACCCCTTCCTTTTCCCGAACCCTATTTTTAGGTCAGAGGCTAAATACCAAGGCCGCCTTTATCAAGGTCAAGTCCGCACCCACTCCCAGGAGGCTCCGCCCTCTCAGAGTCGTTAATGAGAAAGTCGTCGGTATCGATTTGGGAACCACCAACTCCGCCGTGGCCGCCATGGAAGGCGGTAAGCCCACCATCATCACCAACGCCGAGGGCCAGAGAACCACTCCCTCCGTCGTGGCCTACACCAAGAACGGCGACAGGCTCGTGGGCCAAATCGCCAAGCGTCAGGCCGTCGTCAACCCCGAGAACACTTTCTTCTCCGTCAAGAGGTTCATCGGCCGCAAGATGTCTGAGGTCGACGAAGAGTCCAAGCAGGTCTCTTACAGAGTCATCCGAGACGACAACGGCAACGTCAAACTCGACTGCCCCGCCATTGGCAAACAGTTCGCTGCTGAGGAAATTTCTGCCCAGGTTCTTAGGAAGCTTGTGGATGATGCTTCCAAGTTTTTGAACGATAAGGTTACCAAGGCTGTTGTTACTGTGCCTGCTTACTTCAATGACTCCCAAAGGACTGCCACCAAGGATGCCGGTCGGATTGCTGGTCTTGAGGTTCTTCGTATTATCAATGAACCAACCGCTGCATCCTTGGCCTATGGCTTTGAAAAGAAAAACAATGAAACAATCCTTGTTTTTGACCTTGGAGGCGGCACCTTTGATGTCTCTGTGCTCGAGGTTGGTGATGGAGTGTTTGAGGTCCTCTCTACTTCTGGTGACACCCACTTGGGTGGTGATGACTTTGATAAGAGAATTGTTGATTGGCTGGCTTCCAACTTCAAGAGAGATGAAGGCATAGACCTTTTGAAAGACAAACAAGCTCTTCAGCGTCTCACTGAGACAGCCGAGAAAGCAAAGATGGAGCTCTCAACATTGACTCAAACTAACATCAGTTTGCCATTCATAACTGCCACGGCTGATGGACCCAAACATATTGAGACCACCATCACAAGGGCTAAATTTGAGGAATTGTGTTCAGATCTTCTTGACAGGCTCAGGACACCCGTTGAAAACTCATTGAGGGATGCAAAACTCTCGTTTAAGGATCTTGACGAAGTCATCCTTGTTGGTGGATCAACACGTATCCCAGCTGTTCAGGAGCTTGTAAAGAAGTTGACTGGCAAGGACCCAAATGTCACTGTCAATCCAGATGAAGTGGTTGCCCTTGGAGCTGCTGTTCAGGCTGGTGTCTTGGCTGGAGATGTCAGCGACATTGTGCTGTTGGATGTCACTCCATTATCTTTGGGTCTGGAAACTCTAGGTGGTGTGATGACAAAAATTATCCCCAGAAACACTACCCTTCCCACCTCAAAGTCTGAGGTTTTCTCAACTGCTGCTGATGGACAGACCAGTGTAGAGATCAACGTCCTTCAGGGTGAGAGAGAATTTGTTAGGGACAATAAATCACTTGGTAGCTTCCGCCTGGACGGTATCCCTCCTGCACCTCGTGGTGTTCCCCAGATTGAGGTGAAATTTGACATTGATGCCAATGGCATTCTCTCCGTCGCTGCTATTGACAAAGGCACAGGGAAGAAGCAAGATATTACCATTACTGGTGCTAGCACCTTGCCTTCAGATGAGGTGGAGAGAATGGTAAACGAAGCTGAGAAATTTTCAAAGGAAGACAAAGAAAAGAGGGATGCCATTGACACAAAGAACCAGGCAGATTCTGTGGTGTACCAGACAGAAAAGCAATTGAAAGAGCTTGGAGATAAGGTTCCTGGCCCTGTAAAAGAGAAGGTTGAAGCAAAACTAGGGGAGCTTAAAGATGCAATTTCTGGGGGTTCAACCCAAGCTATTAAGGATGCCATGGCTGCACTGAACCAGGAAGTCATGCAGCTTGGTCAGTCCCTTTACAACCAGCCGGGAGCTGCAGGTGCAGGAGGGCCAACACCACCTGGTGCCGACTCTGGCCCCTCAGAATCCTCAGGTAAGGGACCCGACGGAGATGTCATCGATGCAGATTTCACCGACTCTAAATGA

>Glyma17g08020 ATGGCGACAAAGGAAGGCAAAGCCATAGGCATCGATCTCGGCACGACCTACAGCTGCGTGGGCGTGTGGCAAAACGACCGCGTCGAGATCATCCCCAACGACCAAGGCAACCGAACCACTCCCTCTTATGTAGCCTTCACCGACACCGAGAGGCTCATCGGAGACGCGGCGAAGAACCAAGTCGCCATGAACCCGCAGAACACCGTCTTCGACGCCAAGCGTTTAATCGGTCGCAGATTCTCAGACTCTTCAGTTCAAAACGACATGAAGCTGTGGCCGTTTAAGGTCGTGGCTGGCCCTGGCGACAAGCCCATGATCGTGGTCAATTACAAAGGCGAGGAGAAGAAATTCTCCGCCGAAGAGATATCTTCCATGGTGTTGGTCAAGATGAGGGAAGTGGCAGAGGCGTTTCTCGGACACGCCGTGAAGAACGCTGTTGTCACTGTCCCTGCGTACTTCAACGACTCGCAGAGGCAGGCTACGAAGGACGCAGGGGCAATTTCGGGTTTGAATGTGTTGAGGATTATCAATGAACCCACCGCTGCTGCCATTGCGTATGGGTTGGATAAAAAAGCTTCGAGAAAAGGTGAACAGAACGTGCTTATCTTTGACCTTGGTGGTGGTACTTTTGATGTTTCGATATTGACCATCGAGGAAGGGATTTTCGAAGTGAAGGCCACTGCTGGTGATACTCATCTCGGAGGTGAAGATTTTGATAACAGAATGGTGAATCACTTTGTTTCTGAATTCAAAAGGAAGAACAAGAAGGATATTAGTGGGAATGCCAGAGCGTTGAGGAGGTTGAGGACAGCGTGTGAGAGAGCCAAGAGAACGCTCTCTTCCACAGCGCAGACAACTATTGAAATCGATTCACTATACGAAGGGATTGATTTCTATGCTACAATTACGAGAGCTAGGTTTGAGGAGATGAACATGGATTTGTTCAGGAAGTGCATGGAGCCGGTGGAGAAGTGTTTGCGTGACGCCAAGATAGACAAGAGTCAGGTTCATGAGGTTGTGCTTGTTGGAGGTTCCACTAGGATCCCCAAGGTTCAGCAACTCTTGCAGGATTTCTTCAACGGGAAAGAGCTTTGCAAGAGTATTAACCCCGATGAAGCTGTGGCGTACGGTGCTGCTGTTCAGGCCGCGATCTTGAGCGGCGAAGGAGACGAGAAGGTTCAGGATTTATTGCTGCTGGATGTTACACCACTCAGTCTCGGTCTTGAAACTGCTGGTGGTGTCATGACTGTGCTGATTCCGCGGAACACAACTATTCCCACGAAGAAGGAGCAGATTTTCTCAACCTATTCTGATAACCAGCCCGGGGTGTTGATCCAAGTGTTTGAAGGAGAACGGGCTAGAACAAAGGACAACAATCTTCTCGGGAAGTTCGAGCTTACAGGGATCCCTCCAGCACCAAGAGGAGTGCCTCAGATCAATGTCTGCTTCGACATCGACGCTAACGGGATTCTGAATGTCTCTGCAGAGGATAAGACTGCTGGTGTGAAGAACAAGATCACGATCACAAACGACAAGGGTAGGTTGAGCAAGGAGGAGATTGAGAAGATGGTGAAGGATGCAGAGAGGTACAAGGCAGAGGATGAAGAGGTGAAGAAAAAAGTGGAGGCTAAAAATTCGCTTGAGAATTACGCGTATAACATGAGGAACACGATAAAGGATGAGAAGATAGGAGGGAAGCTGAGCCCGGATGAGAAGCAGAAGATTGAGAAGGCTGTGGAGGATGCGATACAGTGGTTGGAGGGAAACCAGATGGCGGAAGTGGACGAGTTTGAGGACAAGCAGAAGGAGTTGGAAGGGATCTGCAACCCCATCATTGCTAAGATGTACCAGGGTGCTGCTGGACCTGGTGGAGATGTTCCTATGGGTGCTGACATGCCTGCTGCTGGTGCTGGACCTAAAATTGAAGAAGTTGACTAA

>Glyma17g11650 ATGAAGAGAATGATAGCGTTGGGATTTGAGGGTTCAGCAAACAAGATTGGTGTTGGGGTAGTGACCTTAGATGGCACAATTCTGTCAAACCCACGCCACACATACATCACCCCTCCTGGTCAAGGCTTTCTTCCCAGAGAGACAGCACAGCACCACCTACAACACGTTCTTCCCCTCGTCAAATCCGCTTTGGAAGTCGCACAAATCGCTCCGCAGGACATTGACTGCCTCTGCTACACCAAGGGTCCCGGCATGGGAGCTCCTTTGCAAGTCTCCGCCATTGTTGTCCGTGTTCTCTCACAGCTTTGGAAGAAGCCGATTGTTGCTGTCAATCACTGCGTGGCACACATCGAGATGGGAAGGATTGTAACCGGTGCTGATGACCCTGTTGTCTTGTATGTTAGTGGTGGCAACACTCAAGTCATTGCTTACAGCGAGGGGCGTTATAGAATCTTTGGAGAAACTATTGACATTGCTGTGGGGAATTGCTTGGATCGCTTTGCAAGGGTCTTGACGCTTTCCAATGATCCAAGCCCCGGATATAACATTGAGCAGCTTGCAAAAAAAGGAGAGAAGTTTATAGACCTGCCTTATACTGTTAAAGGGATGGATGTATCTTTTAGTGGAATATTGAGCTATATTGAAGCAACCGCTGCTGAAAAGCTAAAGAATAATGAGTGCACTCCTGCGGACTTGTGCTACTCTCTGCAGGAGACACTGTTTGCCATGCTTGTGGAGATAACGGAGCGGGCTATGGCTCATTGTGACACGAAAGATGTGCTTATAGTTGGTGGTGTAGGTTGCAATGAGCGGTTGCAAGAGATGATGAGAATCATGTGCTCTGAACGCGGCGGAAGATTGTTTGCCACCGATGATAGATATTGCATTGATAATGGAGCAATGATAGCTTATACTGGCCTCCTTGAATTTGCTCATGGTGCATCAACTCCACTAGAGGATTCTACGTTCACCCAGCGGTTCCGGACAGATGAAGTGAAAGCAATATGGAGAGAAGCAAATTTGGAAAATTTGAATGGGCTTGCAGAGAAGAGTATTTGA

>Glyma17g14280 ATGGACGCATCAAAACTCAATCAATTGAAGCATTTCATCGAACAGTGCAAGTCCAACCCTTCCCTCCTCGCTGATCCTTCACTCTCCTTCTTCCGCGACTATCTTCAAAGTCTCGGCGCGAAACTTCCTGAGTCTGCTTATTCCGAATCGACGGGCGTGGAGAGGGATGAGGACATAGAGGATCTTACGGAGGAGCACGAGAAGGTAGAAGAAGAAGAAGAAGAAGAGGACGATGTAATTATTGAATCCGATGTTGAGCTTGAGGGTGAAACCTGTGAGCCTGATGATGATCCTCCACAGAAGATGGGAGACCCCTCTGTCGAGGTTACTGAAGAGAATCGTGATGCATCGCAGATGGCCAAAATTAAAGCCATGGATGCTATTTCTGAAGGTAAGTTGGAGGAGGCGATTGAGAACTTAACAGAAGCTATTTCACTCAATCCTACCTCTGCCATAATGTATGGAACTAGAGCCAGTGTTTACATCAAAATGAAGAAACCCAATGCTGCGATCCGTGATGCAAATGCTGCTTTGGAGATTAATCCTGATTCTGCTAAAGGATACAAATCACGTGGCATAGCACGAGCAATGCTTGGTCAATGGGAAGAAGCTGCAAAGGATCTTCATGTGGCTTCAAAGTTAGACTATGACGAGGAAATAAATGCTGTACTAAAAAAGGTGGAACCAAATGCTCACAAGATAGAGGAACACCGTCGGAAATATGAAAGGCTGCACAAAGAAAGAGAGGATAAAAAAAAGGAGCGTGAGAGGCAGCGGCGCCGTGCTGAAGCTCAGGCTGCCTATGAGAAGGCTAAGAAGCAAGAGCAATCATCTTCCAGTAGAAATCCTGGAGGCATGCCTGGTGGGTTTCCTGGGGGCTTCCCAGGGGCCGGGGGCATGCCAGGGGGCTTCCCAGGGGCCGGGGGCATGCCTGGGGGCTTCCCAGGGGCTGGGGGCATGCCTGGGGGATTCCCAGGGGCTGGTGGCATGCCTGGGGGATTCCCAGGGGCTGGTGGCATGCCTGGAAACATTGATTTTAGCAAAATCTTGAGTGACCCTGAACTGATGGCGTCATTTGGTGATCCAGAGATTATGGCTGCTCTTCAAGATGTTATGAAGAACCCTGCTAATTTTGCCAAGCACCAATCAAATCCAAAGGTAGCTCCTGTAATTGCGAAAATGATGACCAAACTTGGAGGTGGTCCCAAGTGA

>Glyma18g05480 ATGGAGAAGCTTAACTTGGCCCTTGTGTCTTCCCCAAAGCCTTTGATGTTGGGACATGTTCCTGCAATAGACGCCACATCCAGAGACGTTTTCAGAAGAAAGCACTTCTCTTTTGGGAGGGTCTTAATTGCTCCTCATCGTTGCAGATTCCGTGTTTCTGCACTCTCTTCCTCCCATCGTAATCCGAAATCTGTGCAGGAGAAGCTGATAGTAAAGCATTTTGCTAGTATTTCTTCTTCGAATACTCAAGAAGCGACGTCAACTGGAGTTAACCCACAATTATCACCATCTTCAACTATAGGGTCACCTCTCTTCTGGATTGGTGTTGGTGTTGGGCTTTCTGCACTGTTTTCAGTGGTAGCTTCAAGATTAAAGAAATATGCAATGCAACAAGCTTTCAAGACCATGATGGGCCAGATGAATTCACAAAATAACCAATTTGGCAATGCTGCCTTTTCTCCGGGATCTCCTTTTCCCTTTCCAATGCCTACAGCAGCAGGGCCCACTGCACCTGCTAGTTCTGCAACTACTCAATCTCGAGCACCTTCAGCATCTAGTGCATCTCAATCCACTATCACAGTAGATATACCTGCAGCAAAAGTAGAAGTTGCTCCAACTACTAATGTTAAAGATGAAGTCGAAGTAAAGAATGAACCCAAAAAAATTGCTTTTGTAGATGTTTCTCCAGAAGAAACTGTGCAGGAGAGTCCTTTTGAAAGTTTTAAAGATGATGAATCAAGTTCCGTCAAGGAAGCTCGGGTTCCAGATGAAGTTTCTCAAAATGGAGCCCCCTCTAACCAAGGTTTTGGTGATTTTCCTGGTTCTCAATCTACAAAAAAATCAGTCTTGTCAGTGGATGCTTTGGAGAAAATGATGGAGGACCCAACAGTGCAGAAGATGGTTTATCCCTATTTACCCGAGGAGATGAGGAACCCTACTACCTTCAAATGGATGCTGCAGAATCCACAGTACCGTCAACAACTTGAAGAAATGCTAAACAACATGGGTGGAAGCACAGAATGGGACAGCCGAATGATGGATACCTTAAAGAATTTTGACCTTAATAGTCCTGAAGTTAAGCAGCAATTTGATCAAATTGGGCTTTCTCCAGAAGAAGTCATTTCAAAGATTATGGCCAATCCTGAGGTTGCAATGGCATTTCAAAATCCTAGAGTTCAAGCAGCTATCATGGATTGTTCGCAGAATCCAATGAATATTACTAAATACCAAAATGATAAGGAGGTTATGGATGTCTTCAATAAAATATCAGAACTCTTCCCTGGAGTAGGTTCACCTTGA

>Glyma18g05610 ATGACAAAATCTAAAGAAGATCATGGAATTGCCATAGGAATCGACCTTGGCACGACTTACTCATGTGTTGCAGTATGGCAGGAGCATCACTGTCGAGTGGAGATCATCCACAATGATCAGGGTAACAATACAACTTCTTTTGTTGCTTTCACGGACGACGAAAGGTTGCTAAAAATCAGGCTGCTACCAATCCAGAGAACAATGTCTTTGGACAAGATGTTATGGCCATTTAAGGTTGTTGCTGGTACTCATGACAAACCTATGATTATCCTTAACTACAAGGATGAGGAGAAGCACTTTTGTGCCGAGGAAATATCGTCCATAGTTCTTGCAAAGATGTGGGAGATTGCAGAAGCGTTTTTGGAGAAACGTGTAAAGAATGCAGTGATTGCTGGTCTCAATGTTATGCGGATAATCAATGAACCCACTCCTGCAGCCATTGCATATGGCCTCAACAGGAGGACTAATAATTGTGTTGGAGAGCGAAAAATTTTCATCTTTGACCTTGGTGGTGGTACTTTTGATGTGTCTCTCCTTACTCATAAGGGTAAGATCTTCCAAGTTAAGGTAACAACTGGAAATGGTCACCTTGGGGGAGAGGAAATCGATAACAGAATGGTGGACTACTTTGTAAAGGAGATCAAAAGGAAAAAAAAAGTAGACATTAGTGGGAACCCAAAAGCGCTAAGGAGGTTAAAAACTGCGTGTGAGAGGTCAAAAAGAATACTCTCATGTGCTGTAGCTACTCACATTGAGACATATGCTTTATCTGATGGCATTGACTTCTGTTCTTCAACCACTCGTGCAAGGTTTGAGGAAATCAATATGGATCTCTTTAAGGAGTGTATGGAGACAGTAGATAAGTGTCTTACTGATGCTGAAATGGACAAGAGCAGTGTACATGATGTTATCCTTGTTGGTGGTTCTTCTAGGATTCCCAAAGTGCAAGAGCTATTGCCAGGCTTTTTCAATGGAAAGGATCTAGTTTATGATAATCAATCCTCTGTTGGAATTAAGGTTTATGAGGATGAGAGAACAAGAGCCAGTGATAACAATTTGCTGGGTTCTTTTAGTCTTTCTGGCCTTCCTCCAGCTCCTCATGGCCATCCTTTTGATGTGTGTTTTGCCATTGATGAAAATGGTATCCTATCTGTTTCTGCTAAGGAAAAAACCACTGGCAATAGCAATAAAATTGTCATAACCAATGAAAGAGAAAGATTCATACAAATGGAGAATGCGTTGGAAAATGGGAATTTAAGTTCAAAACTTTGCTCAGAAGACAAGGAGAAGATCAGTTCTGCAATTACAAAGGCCACAAAGTTGCTTGAAGGTGAAAATCAAAATGGTGAAATAGATGTGTTTGAGAATCTCTTTGAACGTGTCATTGGAAAGTTTGATTTTTAA

>Glyma18g11520 ATGAGTGTGGTGGGGTTTGACATTGGTAATGAGAACTGTGTCATTGCCGTAGTCAGGCAACGAGGCATTGATGTTTTGTTGAATTATGAATCTAAACGCGAAACCCCGGCTGTGGTCTGCTTTGGCGAGAAGCAGCGGATTTTGGGGTCTGCTGGTGCTGCTTCTGCTATGATGCACATCAAGTCCACAATATCTCAAATAAAGAGACTGATAGGAAGGAAGTTTGCGGATCCTGATGTGGAAAAAGAGCTGAAAATGCTCCCTGTTGAAACTTCTGAGGGTCAAGATGGAGGCATTTTGATTCATTTGAAGTACATGGGGGAGATTCATGTATTTACACCTGTTCAATTATTGTCCATGCTCTTTGCTCACTTGAAGACCATGACCGAGAAAGATTTGGAGATGCTCATTTCGGATTGTGTTATCGGGATCCCATCATACTTTACCGACTTGCAGAGACGGGCGTATCTTGATGCAGCGAAAATTGCCGGGTTGAAGCCTTTGAGATTGATCCATGATTGTACTGCAACTGCCCTTAGTTATGGAATGTATAAAAAAGATTTTGGAAGTGCAGGTCCAGTAAATGTTGCATTTATTGATATTGGTCACTGTGATACTCAGGTCTCAATTGCGTCATTTGAGTTTGGGAAAATGAAGATACTTTCACATGCGTTTGACAGGAGCTTAGGGGGGAGGGACTTTGATGAGGTTATATTTAGTCATTTTGCAGCAAAATTCAAGGAAGAGTACCACATTGACGTGTATTCTAATACCAAGGCATGCTTTAGGCTACGTGCAGCATGTGAGAAATTGAAGAAAGTTTTGAGTGCAAATCTTGAGGCGCCTCTAAATATCGAGTGTTTGATGGATGAGAAAGATGTCAAGGGATTTATCACAAGGGAAGAATTTGAGAAGCTGGCATCAGGATTACTGGAGAGAGTTTCTATTCCTTGCCGCAGAGCATTAATTGATGCAAACTTGACAGAAGAGAAGATTTCTTCTGTAGAGCTAGTTGGTTCGGGTTCTAGGATTCCAGCAATAAGTACATTACTAACTTCTCTGTTCAAGAGAGAACCCAGCCGACAGCTGAATGCAAGTGAGTGTGTAGCCCGTGGTTGTGCTCTACAGTGTGCAATGCTCAGTCCTATTTACCGTGTGAGAGAATACGAGGTCAAGGATGTTATTCCCTTTTCAATTGGACTTTCATCAGATGAAGGTCCAGTTGCTGTGAGATCAAATGGTGTACTTTTCCCAAGAGGCCAACCCTTTCCAAGTGTTAAAGTCATAACCTTTCGGCGAAGTGATTTGTTTCATTTGGAAGCTTTCTATGCTAATCCAGATGAACTACCACCTGGGACATCTCCTATAATTAGTTGTGTCACGATTGGTCCTTTCCATGGATCCCACGGTAGTAAGATCAGAGTTAAAGTTAGAGTTCCACTTGATCTGCATGGCATTGTCAGTATTGAATCAGCTACATTGATCAAGGATGATTCGGTTATGGCTGGTGATTATCATTCAAATTCTGATGCAATGGATATTGATCCTATTTCTGAGACAGTTACCAATGGGTTTGAAGATAATACCAATAAGAACTTGGAATCTCCATGTAGTTCTGCTGATGGTACAAGAAAAGATAACAGAAGGCTTAATGTGCCAGTGAATGAGAATGTCTATGGTGGAATGACAAAGGCAGAGATCTCAGAAGCTCGTGAAAAAGAACTCCAGTTGGCCCATCAGGACAGAATTGTAGAGCAAACCAAAGAAAAGAAGAACAGCTTGGAGTCTTATGTCTATGATATGAGGAGTAAGCTCTTCCACACATACCGAAGCTTTGCAAGTGAACAAGAGAAGGATGACATATCTAGGACCCTTCAAGAGACTGAGGAATGGCTTTATGAGGATGGTGTTGATGAAACTGAACATGCTTATTCTTCAAAACTGGAAGATCTGAAAAAGCTGGTAGATCCAATTGAGAATCGGTACAAAGATGATAAAGAAAGAGTGCAAGCTACAAGAGATTTATCGAAGTGCATTTTAAAGCATCGTGCTTCTGCAGATTCCCTTCCAACCCAGGATAAAGAACTGATCATCAATGAGTGCAATAAAGTGGAGCAGTGGTTGGAAGAGAAGATCCAGCAACAAGAATCATTTCCTAGAAATACTGATCCAATATTATGGTCAAGTGATATCAAGAGCAAGACAGAGGAGTTAAACTTAAAATGCCAACAGATATTGGGATCTAAGGCTTCTCCATCTCCAGAAGACAAAGACAAGCCGGATACATTCAATGATCCCTGA

>Glyma18g13077 ATGGCTTTCGCATGCAGCAGAATCGCGCAACGAACATCAATTTCATCTATAAAATCAGCCATCAAATCCAACATTCGCGCTTCTTCATTCTCCAAACCAGCTTCCTCCTTTTCTCCTATTTGCCAATCCTTATTGGCCAGGATTTTGCAGGAGCTGAGATGCGTGCAGTCGATGTTGCCGCTGCACAGCACGGTGGCGGCGGCGAGAATGATGTCGTGTCTGACTTTCAAAAGTTGTCGAGCGCTTTCACCGGAGTTCTTTAAAAAGGAGCCTAGGTGGACTATGTGTGCAATTCTCAGCCCAACTTTTAAAGTACGGGAGTTTCAGTGTATGAGAATGATGGGTCTTGAGTGGAAGGATATTGCTTTTGTTCAGAAGTCAAATTTGGAGAGTTATATACAATGA

>Glyma18g52470 ATGGCAACAAATGGCAAGACACCTGCGATAGGAATCGATTTGGGCACGACATACTCATGCGTTGCAGTGTGGCGGCATGATCGAGTGGAGATCATCGTGAACGACCAAGGAAACAGAACAACACCCTCTTATGTTGCTTTCAATAACACCCAAAGGATGATTGGTGATGCTGCCAAGAACCAGGCTGCTACCAATCCAACCAACACTAGCACACCGGTGATAGGGATCGATCTAGGCACGACATACTCGTGCGTTGCAGTGTGGCAACACGACCGTGTGGTGATCATCACGAACGACCAAGGGAACAGAACAACACCCTCTTGTGTTGCCTTCAAAAACACCCAAAGGATGATCGGTGATGCTGCTATAAATCAAGCTGCTGCCAATCCAACCAATACTGTCTTTGGTGCTAAGCGGCTAATTGGTAGGAGATTTAGTAATCCAGAGGTTCAAAGTGATATGAAGCAATGGCCATTCAAAGTCATTGCTGATGTTAATGACAAACCAATGATCGCTGTTAATTACAATTGTGAGGAAAGGCACTTTTCTGCAGAAGAAATTTCGTCCATGGTTTTGGAAAAAATGCGAGCGATTGCAGAGTCATTCCTTGGATCAACAGTGAAGAATGCTGTTATCACTGTGCCAGCTTACTTCAATGACTCTCAGCGACAAGCTACCAAAGATGCTGGTGCCATTGCTGGCCTCAATGTTTTGAGAATCATCAATGAGCCAACTGCTGCGGCAATTGCATATCGGCTTGAAAGGAAAAATTGTAATAATGAAAGAAGGAATGTTTTTGTGTTTGATCTTGGTGGTGGTACTTTGGATGTGTCTCTTCTTGTTTTTGAGAAGGATTATATCCGAGTTAAGGCAACATCTGGAGACACTCACCTCGGAGGAGAGGACTTCGATAACAATATGGTGACTTACTGTGTGAAAGAGTTTCAGAGAAAGAATAAAAAGGACATTAGTGGAAACGAAAGAGCCCTTAGGAGGTTGAGGACTGCTTGTGAGAAAGCAAAGAGAATACTTTCATCCACTGTAATGACCACCATTGAGGTAGACTCTTTGTATGATGGTATTGATTTCCACTCATCAATAAGTCGCGCAAAGTTTGAGGAACTCAACATGGACTACCTTAACAAGTGTATGGAGTTTGTAGAGAAGTGTCTGATAGATGCTAAGATGGACAAGAGTAGTGTTCATGATGTTGTCCTCGCAGGTGGATCTACTAGGATTCCCAAATTGCAGCAACTATTAAGTGACTTCTTTGATGGGAAGGATCTCTGCAAATGCATCAATGCTGATGAGGCCGTTGCATATGGTGCTGCTGTCCATGCTTCTATGCTTAATGGTGAGTCCAGTGAGAAGGTTCAAAACACTTTACCGAGGGAAGTCACTCCTCTTTCCCTTGGGTTGGAAAAAGAAGGAGGTATCATGAAAGTAATCATTCCTAGGAATACTAGCATTCCTACAAAGATGGAAGATGTATTCACAACACATTTGGATAACCAAATCAATATCTTGATTCATGTTTATGAGGGTGAGAGGCAAAGAACTAGAGACAACAACTTGTTGGGCAAGTTTGTGCTAGAAATTCCTCCAGTTCCGCGTGGTGTTCCTCAAATAATCGTTTGCTTTGAAGTTGATGATGAGGGTATCTTGCACGTCTCTGCCAAGGAGAATTCCTTGGGAATAACCAAGAAGGTGACCATAATAAATGACAAAGGAAGGCTTTCTGAGGAAGAAATTAAGAGGATGATATCAGAAGCAGAGAGGTACAAAGCTGAAGATGAGATGTATAGGAAGAAGGTAGAAGCAAGGTATGCATTGGAGAAGTACGCATACAACATAAGGAATGCTATAAAGCATAAGGGGATTAGTTTGAAGCTTTCTCCGGAAGACAAGGAAAAGATCAATGATGCAGTTGATCGTGCCTTAGAATGGCTTGAGGTCAGTGTGGATGCTGAAAAAGAAGATGTTGACAATTTCCGGGGCAATCTTTCTAGCGTTTTTGATACAATCATGGTTAAAATGATAAAGGGTGAGGATAATGGTGCGCCTCCAGAATCGCTGGTTATCAATATTGGCAAAATTTGGTCTCCAGGCAGTGTATTCAGCTGTTACAGGTGA

>Glyma18g52471 ATGGCAACAAATGAGAGCACACCGGTGATAGGGATCGATCTAGGCACGACATACTCGTGCGTTGCAGTGTGGCAACACGACCGTGTGGTGATCATCACGAACGACCAAGGGAACAGAACAACACCCTCTTGTGTTGCCTTCAAAAACACCCAAAGGATGATCGGTGATGCTGCTATAAATCAAGCTGCTGCCAATCCAACCAATACTGTCTTTGGTGCTAAGCGGCTAATTGGTAGGAGATTTAGTAATCCAGAGGTTCAAAGTGATATGAAGCAATGGCCATTCAAAGTCATTGCTGATGTTAATGACAAACCAATGATCGCTGTTAATTACAATTGTGAGGAAAGGCACTTTTCTGCAGAAGAAATTTCGTCCATGGTTTTGGAAAAAATGCGAGCGATTGCAGAGTCATTCCTTGGATCAACAGTGAAGAATGCTGTTATCACTGTGCCAGCTTACTTCAATGACTCTCAGCGACAAGCTACCAAAGATGCTGGTGCCATTGCTGGCCTCAATGTTTTGAGAATCATCAATGAGCCAACTGCTGCGGCAATTGCATATCGGCTTGAAAGGAAAAATTGTAATAATGAAAGAAGGAATGTTTTTGTGTTTGATCTTGGTGGTGGTACTTTGGATGTGTCTCTTCTTGTTTTTGAGAAGGATTATATCCGAGTTAAGGCAACATCTGGAGACACTCACCTCGGAGGAGAGGACTTCGATAACAATATGGTGACTTACTGTGTGAAAGAGTTTCAGAGAAAGAATAAAAAGGACATTAGTGGAAACGAAAGAGCCCTTAGGAGGTTGAGGACTGCTTGTGAGAAAGCAAAGAGAATACTTTCATCCACTGTAATGACCACCATTGAGGTAGACTCTTTGTATGATGGTATTGATTTCCACTCATCAATAAGTCGCGCAAAGTTTGAGGAACTCAACATGGACTACCTTAACAAGTGTATGGAGTTTGTAGAGAAGTGTCTGATAGATGCTAAGATGGACAAGAGTAGTGTTCATGATGTTGTCCTCGCAGGTGGATCTACTAGGATTCCCAAATTGCAGCAACTATTAAGTGACTTCTTTGATGGGAAGGATCTCTGCAAATGCATCAATGCTGATGAGGCCGTTGCATATGGTGCTGCTGTCCATGCTTCTATGCTTAATGGTGAGTCCAGTGAGAAGGTTCAAAACACTTTACCGAGGGAAGTCACTCCTCTTTCCCTTGGGTTGGAAAAAGAAGGAGGTATCATGAAAGTAATCATTCCTAGGAATACTAGCATTCCTACAAAGATGGAAGATGTATTCACAACACATTTGGATAACCAAATCAATATCTTGATTCATGTTTATGAGGGTGAGAGGCAAAGAACTAGAGACAACAACTTGTTGGGCAAGTTTGTGCTAGAAATTCCTCCAGTTCCGCGTGGTGTTCCTCAAATAATCGTTTGCTTTGAAGTTGATGATGAGGGTATCTTGCACGTCTCTGCCAAGGAGAATTCCTTGGGAATAACCAAGAAGGTGACCATAATAAATGACAAAGGAAGGCTTTCTGAGGAAGAAATTAAGAGGATGATATCAGAAGCAGAGAGGTACAAAGCTGAAGATGAGATGTATAGGAAGAAGGTAGAAGCAAGGTATGCATTGGAGAAGTACGCATACAACATAAGGAATGCTATAAAGCATAAGGGGATTAGTTTGAAGCTTTCTCCGGAAGACAAGGAAAAGATCAATGATGCAGTTGATCGTGCCTTAGAATGGCTTGAGGTCAGTGTGGATGCTGAAAAAGAAGATGTTGACAATTTCCGGGGCAATCTTTCTAGCGTTTTTGATACAATCATGGTTAAAATGATAAAGGGTGAGGATAATGGTGCGCCTCCAGGTGCTGTTGCTAGCTCTGGTAGCAAAAGTGGGAAGAATCGCTGGTTATCAATATTGGCAAAATTTGGTCTCCAGGCAGTGTATTCAGCTGTTACAGGTGATATCATTGGATTTGTTTCCGTGATTGTTGACTGTTTGGCAAATTAG

>Glyma18g52480 ATGGCAACAAATGGCAAGACACCTGCGATAGGAATCGATTTGGGCACGACATACTCATGCGTTGCAGTGTGGCAGCGTGATCGAGTGGAGATCATCGCGAACGACCAAGGAAACAGAACAACACCCTCTTATGTTGCTTTCAATAACACCCAAAGGATGATTGGTGATGCTGCCAAGAACCAGGCTGCTACCAATCCAACCAACACTGTCTTTGACGCAAAGCGCCTAATTGGTAGGAGATTTAGTGATCAAGAGGTTCAAAGTGATATGGAGCTATGGCCATTCAAAGTCATTGCTGATGTTAATGGCAAACCAATGATTGCTGTTGATTACAATTGTGAGAAAAAGCAATTTTCTGCAGAAGAAATTTCATCCATGGTTTTGGCAAAGATGCTTGACATTGCAGAGTCTTTCCTTGGATCAACAGTGAAGAATGCTGTTATCACTGTGCCTGCTTACTTCAATGATTCTCAGCGACAAGCTACTAAAGATGCTGGTAAAATTGCTGGCCTCAATGTTTTGAGAATCCTCCATGAGCCAACTGCTGCTGCAATTGCATATCGGCTTGAAATGAAAAATTGTAATAATGATAGAAGGAACGTTTTTGTGTTTGATCTTGGTGGTGGTACTTTGGATGTGTCTCTTCTTGTTTTTGAGAAGGATCATATCCGAGTTAAGGCAACTACTGGAGACACTCACCTCGGAGGAGAGGACTTCGATAACAATATGGTGACTTACTGTGTGAAAGAGTTTAAGAGAAAGAATAAAATGGACATTAGTGGAAACAAAAGAGCCCTTAGGAGGTTGAGGACTGCTTGTGAGAAAGCAAAGAGGATACTCTCATGCTCTACAATGACCACCATTGAGGTAGACTCTTTGTATGATGGTATTGATTTCCACTCATCAATAAGTCGCGCAAAGTTTGAGGAACTCAACAAGGACTACCTTAACAAGTGTATTGAGTTTGTAGGGAAGTGTCTGATAGATGCTAAGATGGACAAGAGTAGTGTTCATGATGTTGTCCTCGCAGGTGGATCTACTAGGATTCCCAAATTGCAGCAACTATTAAGTGACTTCTTTGATGGGAAGGATCTCTGCAAATGCATCAATGCTGATGAGGCCGTTGCATATGGTGCTGCTGTCCATGCTTATATGCTTAATGGTGAGTCCAGTGAGAAGGTTCAAAACGCTTCACTTTGGGAAGTCACTCCTCTTTCCCTTGGGTTGCAAGAAGATGGAGGTATCATGAAAGTAATCATTCCTAGGAATACTAGCATTCCTACAAAGATGGAAGATGTACTCACAACACATTTTGATAACCAAACCAATATCTTGATTCATGTATACGAGGGTGAGAGGAAAAGAACTAGAGACAACAACTTGTTGGGTAAGTTTGTGCTAGAAATTCCTCCAGTTCCACGTGGTGTTCCTCAAATTAGCGTTTGCTTTGAACTTGATTATGATGGCATCCTACATGTTTCTGCCGAGGAAAAATCTAGGGGAATATCCAAGAAGCTGGCCATAACAAATGACAAAGGAAGGCTTTCGAAGAAAGAAATTGAAAGGATGATATCAGAAGCAGAGAAGTACAAAGCTGAAGATGAGATGTATAGGAACAAGGTACAATCAAGGCATGCATTGGAGAAGTACGCTTACAACATGAGGGATGCTATAAACATTAAGGAGATTAGCTTGAAGCTTTCTCCAGAAGACAAGAAAAATATCAATGATGCAATTGATTCTGCCTTAGAGTGGCTCGAGGTCAGCATGGATGCAAACCCAAATGATTTTGACAACATGCGGAGCACTCTTTCTAGCGTTTTTAATCCAGTTATTGTGAAGATGATAAAGGATGAGGATAATGTTGCGCCTCCAGATACTGTTGCTAGCTCTGGTAGCAATAGTGTGAAGAATGGCTTGTTATCAATATTGGCAAATTTTGCTCTCGATGCAGTGTATTCAGCTGCTACAGGTGATATCATTGGATTTGCTTCCGTGATTGTTGACTGTTTGTCAATTTAG

>Glyma18g52610 ATGGCCGGAAAAGGAGATGGTCCTGCTATCGGAATCGATCTCGGCACCACCTACTCCTGCGTCGGAGTGTGGCAGCACGACCGTGTCGAAATCATCGCCAATGACCAGGGTAACAGAACCACGCCGTCTTACGTCGCTTTCACTGATTCCGAGCGTTTGATCGGTGACGCCGCCAAGAACCAGGTCGCCATGAACCCCGTCAACACCGTCTTCGATGCTAAGCGTTTGATTGGAAGGAGATTTTCTGATGCCTCCGTTCAGAGTGACATGAAGCTATGGCCATTTAAGGTCATCCCTGGTCCTGCTGACAAACCTATGATTGTGGTCAACTACAAGGGTGAGGACAAGCAGTTCTCCGCTGAGGAAATTTCTTCCATGGTTCTCATGAAGATGCGTGAGATTGCCGAGGCTTATCTCGGTTCCACAGTGAAGAATGCCGTGGTCACTGTTCCCGCTTACTTCAATGACTCCCAGCGTCAGGCCACGAAGGATGCTGGAGTCATTGCGGGTCTCAATGTCATGCGTATCATCAATGAACCCACCGCTGCTGCCATTGCTTACGGTCTTGACAAGAAGGCCACCAGTGTGGGTGAGAAGAACGTGTTGATTTTTGACTTGGGTGGCGGTACCTTTGATGTCTCTCTTCTCACCATTGAGGAGGGTATTTTTGAGGTCAAGGCCACTGCCGGAGATACTCATCTTGGAGGTGAAGATTTTGATAACAGGATGGTTAACCATTTTGTTCAGGAATTCAAGAGGAAGCACAAGAAGGACATCAATGGAAACCCTAGGGCTCTTAGGAGGTTGAGGACTGCCTGTGAAAGGGCGAAGAGGACCCTATCATCTACTGCACAAACCACAATTGAGATAGATTCTCTTTATGAGGGTGTTGACTTCTACACCACAATCACCCGTGCCAGGTTTGAGGAGCTCAACATGGATCTCTTCAGGAAGTGTATGGAGCCCGTTGAGAAGTGTTTGAGGGATGCTAAGATGGACAAGAGCACTGTCCATGACGTTGTCCTTGTTGGTGGATCCACTAGAATTCCTAAAGTGCAGCAATTGTTGCAGGATTTCTTCAATGGCAAGGAGCTTTGCAAGAGCATTAACCCCGATGAGGCCGTTGCTTATGGAGCAGCTGTACAGGCTGCTATTTTGAGTGGTGAGGGCAACGAGAAGGTGCAGGATTTGCTGTTGTTGGATGTTACACCCCTTTCTCTTGGTTTGGAAACTGCCGGCGGCGTCATGACTGTTCTCATTCCCAGGAACACGACTATTCCCACCAAGAAGGAGCAAGTGTTCTCTACTTACTCAGACAACCAACCTGGTGTCTTGATTCAGGTGTATGAGGGAGAGAGAGCTAGAACCAGGGACAACAACTTGTTGGGTAAATTTGAGCTTTCTGGCATTCCCCCAGCACCCAGGGGTGTTCCTCAGATTACTGTCTGCTTTGATATCGATGCCAATGGTATCTTGAATGTCTCTGCCGAGGACAAGACCACAGGGCAGAAGAACAAGATCACCATCACCAACGACAAGGGTAGACTATCGAAGGATGAGATTGAGAAGATGGTCCAAGAGGCGGAGAAGTACAAGGCCGAGGATGAGGAGCACAAGAAGAAGGTCGACGCAAAGAATGCTTTGGAGAACTATGCCTACAACATGAGGAACACCATTAAGGATGAGAAGATTGCATCAAAGCTTTCTGATGACGACAAGAAGAAAATCGAAGATGCCATCGAGAGTGCTATTCAGTGGTTGGATGGAAACCAGCTAGCGGAGGCTGACGAGTTCGAAGACAAGATGAAGGAGCTTGAGAGCATTTGCAACCCAATCATTGCCAAAATGTACCAGGGTGCAGGTGCTCCCGACATGGCTGGAGGCATGGATGAAGATGTTCCTCCATCTGGATCCGGTGGTGCTGGCCCCAAGATCGAGGAAGTTGATTAA

>Glyma18g52650 ATGGCCGGAAAAGGAGAGGGACTCGCCATCGGAATCGACCTGGGCACCACCTACTCCTGCGTCGGAGTCTGGCAGCACGACCGCGTCGAAATCATCGCCAACGATCAGGGCAACCGTACCACACCCTCTTATGTCGCCTTCACCGATACCGAAAGACTCATCGGAGATGCCGCTAAGAACCAGGTCGCCATGAACCCCATCAACACCGTCTTCGATGCGAAGAGGTTGATTGGTAGGAGAGTTAGCGACCCTTCTGTTCAGAGTGATATGAAGTTGTGGCCATTCAAGGTTACTGCTGGTGCTGGTGAAAAACCCATGATTGGTGTCAATTACAAGGGTGAGGAAAAGCAATTCGCTGCTGAGGAAATCTCCTCTATGGTCCTAACAAAGATGCGGGAGATTGCAGAGGCTTACCTTGGGTCGACTGTGAAGAATGCCGTTGTTACTGTGCCTGCTTACTTCAATGATTCTCAGCGTCAAGCCACCAAAGACGCCGGTGTCATTGCTGGCCTCAATGTTATGAGAATAATAAATGAGCCAACTGCTGCTGCAATTGCATACGGGCTTGACAAGAAAGCCACTAGTGTTGGTGAGAAGAATGTCTTGATCTTTGATCTTGGAGGTGGCACCTTTGATGTTTCTCTCCTCACCATTGAGGAGGGTATCTTTGAAGTTAAGGCCACAGCTGGAGACACTCACCTTGGAGGGGAGGATTTTGATAATAGAATGGTGAACCACTTTGTACAAGAGTTCAAGAGGAAGAACAAGAAGGATATTACTGGAAACCCAAGAGCCTTGAGAAGGTTAAGAACCTCTTGTGAGAGAGCAAAGAGGACACTCTCATCCACTGCTCAGACCACCATTGAGATTGATTCTCTGTTTGAGGGCATTGACTTCTATTCAACCATCACTCGTGCCAGGTTTGAGGAGCTCAACATGGACCTCTTCAGGAAATGTATGGAGCCTGTGGAAAAGTGTCTTAGGGATGCAAAGATGGACAAGAGCTCTGTTCATGATGTTGTCCTTGTTGGTGGCTCTACAAGGATTCCTAAAGTTCAGCAGCTGTTGCAGGACTTCTTCAATGGGAAAGATCTTTGCAAGAGCATCAATCCCGACGAAGCAGTTGCTTATGGAGCTGCTGTCCAGGCAGCTATATTGAGCGGTGAAGGCAATGAGAAGGTTCAAGATTTGTTGCTTTTGGATGTCACTCCATTGTCATTGGGTTTGGAGACTGCTGGAGGTGTCATGACTGTATTGATTCCAAGGAATACTACCATTCCTACTAAGAAGGAACAAGTGTTCTCTACATATTCAGATAACCAACCTGGTGTCTTGATTCAAGTTTATGAGGGTGAGAGAACAAGAACCAGGGATAACAACTTGTTGGGTAAGTTTGAGCTGTCAGGCATTCCTCCAGCTCCCCGTGGTGTCCCTCAAATCACTGTTTGCTTCGATATTGATGCTAATGGTATCTTGAATGTCTCTGCCGAGGACAAAACAACTGGCCAGAAGAACAAGATTACCATTACCAATGACAAAGGAAGGTTGTCAAAGGAAGAAATCGAGAAGATGGTTCAAGAAGCTGAGAAATACAAGTCTGAAGATGAGGAGCACAAGAAGAAGGTTGAGGGAAAGAATGCCTTGGAGAACTATGCCTACAACATGAGAAACACAATAAAGGATGAGAAGATCAGTTCAAAGCTTTCCTCTGAAGACAAGACAAAGATTGATAATGCAATTGAGCAGGCCATTCAATGGCTTGATACCAATCAGCTTGCAGAAGCTGATGAATTTGAAGACAAGATGAAGGAGCTTGAGGGCATATGCAATCCCATTATAGCGAAGATGTACCAAGGCGGAGCTGGTACTGGTGGTGACGTCGATGATGATGCTCCACCTGCTGGTGGTAGTGGTGCTGGCCCCAAGATTGAGGAGGTTGATTAA

>Glyma18g52760 ATGGCCAAAAACCAGGGATTTGCCGTAGGAATCGACCTTGGTACAACCTACTCGTGTGTTGCAGTGTGGCAGGGGCAACAAAATAGAGTAGAAATAATTCACAACGACCAAGGGAACAGAACTACACCTTCTTTTGTCGCTTTCACTGACGATCAAAGGTTGATTGGTGATGCTGCTAAGAATCAGGCTGCAGCCAACCCAGAAAACACTGTCTTTGATGCCAAGAGGTTAATTGGTAGGAAATACAGCGATCCCACTATTCAAAATGATAAAATGTTATGGCCATTCAAGGTCATAGCTGATAATAATGACAAACCCATGATCACCGTTAAATACAAGGGCCACGAGAAGCTCCTTTCAGCAGAGGAAGTGTCATCTATGATCCTCATGAAAATGCGGGAGATTGCTGAGGCATATTTGGAAACACCGGTAAAGAGTGCTGTTGTTACAGTGCCTGCTTATTTCAATGATTCTCAGCGTAAAGCCACCATAGATGCTGGAACCATAGCTGGCCTTAATGTTATGCGGATAATCAATGAACCCACTGCAGCAGCTATTGCATATGGCCTTGACAAGAGAATTAACTGTGTTGGAGAGCGTAACATTTTTATCTTTGACCTTGGTGGTGGTACTTTTGATGTTTCTCTCCTTACAATTAAGGACAAGGTCTTCCAAGTCAAGGCTACTGCAGGGAACACCCACCTTGGGGGAGAGGACTTCGACAACAGAATGGTGAATTACTTGGTGCAGGAGTTCAAAAGAATGAACAAGGTCGACATTAGTGGTAACCCCAGAGCTTTACGAAGGTTGAGAACTGCGTGCGAAAAGGTGAAAAGGACACTCTCGTTTGCTGTTACAACCACAATTGAGGTTGATTCATTATCTAAAGGCATTGATTTCTGCATTTCAATCACTCGTGCAAAGTTTCAGGAACTTAATATGGATCTCTTTGAGGAGTGTCTGAAGACTGTTAATAAGTGTCTTACAGATGCAAAGACGGACAAGAGCAGTGTACATGATGTTGTCCTTGTTGGTGGTTCTTCGAGGATTCCCAAAGTGCAGGAACTATTGCAGGAATTCTTTGAGGGAAAGGATTTTTGCAAGAGTATCAACCCTGACGAGGCTGTCGCTTATGGAGCTGCCGTGCAGGCTGCTTTGTTGAGTGACGACATTCAGAATGTTCCAAATTTGGTTTTGTTGGATGTTGCACCGCTGTCACTTGGAATACTACTATTCCAGTTAAGAGGACTCAAGGAAGATAACCAAACCTCTGCGCGTATTGAGGTTTATGAAGGCGAGAGAACCAGAGCAAATGATAACAATTTGCTTGGTTTCTTTAGTCTTTTGGGTTTGGTTCCTGCTCCTCGTGGCCATCCTGTGGATGTATGCTTTACAATAGATGTAAATGGCATTCTATCTGTTTCTGCGGAGGAAACAACCACTGGTTATAGGAATGAGATTACCATAACCAATGACCAAAAAAGGCTTTCAGCTGAGCAGATTAAAAGAATGATTCATGAAGCTGAGAAATATCAGGTTAATGATATGAAGTTCATGAAGAAGGCTAATACAATGAATGCTTTGGATCACTATGTTTACAAGATGAGGAATGCATTGAATAATAAGAATATCAGTTCAAAGCTTTGTTTACAAGAAAGGAAGAAAATCAAATCTGTAATTACAAAGGTGACTGATTTGCTTGAGGGTGATAATCAGCGGGATAAAATAGAGGTGTTTGAGGATCATCTAAATGAGCTTGTGAACCTCTTTGATCGTGTCATTGGCAAGTTTGCTTAG

>Glyma19g35560 ATGGCCGGAAAAGGAGAGGGTCCTGCTATCGGAATCGATCTCGGAACCACCTACTCTTGTGTCGGTGTGTGGCAACATGACCGAGTTGAAATCATCGCCAACGACCAAGGGAACAGAACGACGCCGTCTTACGTCGGCTTCACTGATACCGAGCGTCTCATCGGTGATGCGGCTAAGAACCAAGTCGCCATGAACCCCATCAACACCGTCTTCGATGCCAAGAGGTTGATTGGTCGTAGATTCAGTGACTCCTCTGTTCAGAGTGATATCAAATTGTGGCCTTTTAAGGTCATTGCTGGTGCTGCTGACAAGCCAATGATCGTGGTTAACTACAAGGGTGAAGAGAAGCAATTTGCTGCAGAAGAAATCTCTTCTATGGTGCTCATCAAGATGCGTGAGATTGCTGAGGCTTACCTTGGCTCCACGGTGAAGAATGCTGTTGTCACTGTCCCTGCTTACTTCAATGATTCTCAGCGTCAAGCTACCAAGGATGCTGGAGTCATTGCTGGTCTTAATGTGATGCGAATTATCAATGAGCCTACTGCAGCTGCCATTGCTTATGGTCTTGATAAGAAGGCCACAAGTGTTGGTGAGAAGAATGTGTTGATTTTTGACCTTGGTGGTGGGACATTTGATGTTTCTTTACTAACCATTGAGGAGGGTATCTTTGAGGTGAAAGCCACAGCTGGTGACACCCATCTTGGAGGTGAGGATTTTGATAACAGAATGGTGAACCACTTTGTTCAAGAGTTTAAGAGAAAGAACAAGAAGGACATAAGTGGGAACCCCAGAGCACTTAGAAGGTTGAGGACTGCTTGTGAGAGGGCCAAGAGAACATTGTCATCCACTGCCCAGACCACCATTGAAATTGATTCTCTCTATGAGGGAATTGATTTCTACTCCACTGTTACTCGTGCCAGATTCGAGGAACTGAACATGGATCTCTTTAGGAAGTGTATGGAGCCGGTGGAGAAATGTTTGAGGGATGCTAAAATGGACAAAAGAAGTGTTGATGATGTTGTCCTTGTTGGTGGTTCTACCAGAATTCCCAAGGTTCAACAACTGCTGCAGGACTTCTTTAATGGAAAGGAGCTGTGCAAGAGCATCAATCCTGATGAAGCTGTTGCATATGGTGCTGCTGTTCAGGCTGCAATTTTAAGTGGTGAGGGCAATGAGAAGGTTCAGGATCTTCTCCTCCTTGATGTCACCCCTCTATCTCTTGGTTTGGAGACTGCTGGCGGTGTGATGACTGTCTTGATCCCTAGGAACACTACAATTCCAACCAAGAAGGAACAGGTTTTCTCAACATACTCTGACAACCAGCCTGGTGTGTTGATCCAGGTCTTTGAAGGTGAAAGAGCAAGGACTAAAGATAACAATTTGTTGGGCAAATTTGAACTTTCTGGCATTCCTCCTGCCCCCAGGGGTGTTCCTCAGATTACTGTGTGCTTTGACATTGATGCCAATGGTATTTTGAACGTCTCTGCCGAAGATAAGACCACTGGCCAGAAAAATAAGATCACTATCACCAATGACAAGGGTAGACTGTCAAAGGAAGATATCGAGAAGATGGTTCAAGAGGCTGAGAAGTACAAATCTGAGGATGAAGAGCACAAAAAGAAGGTTGAGGCCAAGAATGCTTTGGAAAACTATGCATACAACATGAGGAACACTGTGAAGGATGACAAAATTGGTGAGAAACTTGATCCTACTGACAAGAAGAAGATTGAGGACGCAATTGAGCAAGCTATCCAGTGGCTAGACAGCAACCAGCTCGCAGAAGCAGATGAATTTGAGGACAAAATGAAGGAATTGGAAAGCATCTGCAATCCTATCATTGCCAAGATGTACCAAGGTGGTGCTGGTCCTGACATGGGTGGTGCTGGTGCTGGGGCAGCCGAGGATGATTATGCTGCTCCTTCTGGTGGAAGTGGTGCTGGCCCCAAGATTGAGGAAGTGGACTAA

>Glyma19g44140 ATGAAGAGAATGATAGCCTTGGGGTTTGAGGGTTCAGCGAACAAGATTGGTGTTGGGGTAGTGACCTTAGATGGCACAATTCTTTCAAACCCACGGCACACATACATCACTCCTCCTGGCCAAGGCTTTCTTCCCAGAGAGACAGCACAGCACCACCTCCAACACGTTCTTCCCCTTATCAAATCCGCTTTGGAAACTGCACAAATCACTCCACATGACATTGACTGCCTCTGCTACACCAAGGGTCCTGGCATGGGAGCTCCTCTGCAAGTCTCTGCTATTGTTGTTCGTGTGCTTTCGCTTCTTTGGAAGAAGCCAATTGTTGCTGTCAATCACTGTGTTGCACACATTGAGATGGGAAGGATTGTTACCGGCGCTGATGACCCCGTTGTCTTGTATGTCAGTGGTGGCAACACTCAAGTCATTGCTTACAGCGAGGGGCGTTATCGAATCTTTGGAGAGACCATCGACATTGCTGTGGGGAATTGCTTGGATCGCTTTGCAAGGGTCTTGACACTTTCCAATGATCCTAGCCCCGGATATAACATTGAGCAGCTTGCGAAAAAGGGAGAGAAGTTTATCGACCTGCCTTATGTTGTCAAAGGGATGGATGTATCTTTTAGTGGGATATTGAGCTATATTGAAGCAACTGCTGCTGAAAAGCTAAAGAATAATGAGTGCACGCCTGCAGACTTGTGCTACTCTCTGCAGGAGACATTGTTTGCCATGCTTGTGGAGATAACGGAGCGGGCTATGGCTCATTGCGACACAAAAGATGTTCTTATAGTTGGTGGTGTAGGTTGCAATGAGCGGTTGCAAGAGATGATGAGGACCATGTGCTCTGAACGTGGTGGAAGATTGTTTGCTACTGATGATAGATATTGCATTGACAATGGAGCAATGATAGCTTATACTGGTCTCCTTGAATTTGCTCATGGCGCATCAACTCCACTAGAGGATTCTACATTCACCCAGCGGTTCCGGACAGATGAAGTGAAAGCAATATGGAGAGAAGCAAATCTGGCAAAATTGAATGGGCTTGCAGAGAAGAGCACTTGA

>Glyma20g16070 ATGATTGATTGGAATTATGGACGGATGATGACTGCTTGCGGCCAAACGCCGCAAAAAATGGCGTCGTTGAAGGTGGCGCTACTGGCGCTGTTCTCCGTCGCGCTCATGTTCTCTCCGTCGCAGTCCGCGGTCTTCAGCGTCGATCTAGGTTCCGAATCGGTGAAAGTGGCGGTGGTGAACCTGAAGCCCGGCCAATCCCCGATCTGCATTGCGATCAACGAGATGTCCAAGCGCAAATCCCCGGCGCTGGTCTCCTTCCACGACGGCGACCGCCTCCTCGGTGAGGAGGCCGCCGGCCTCGCCGCGCGCTACCCGCAGAAGGTCTATTCCCAAATGCGCGACCTCATCGCCAAACCCTACGCCTCCGGGCAGAGGATTCTCAACTCAATGTACCTTCCCTTCCAGACCAAGGAAGATTCACGAGGTGGCGTGAGTTTTCAAAGCGAAAACGACGACGCCGTTTACTCCCCCGAGGAGCTGGTGGCCATGGTGTTAGGTTATGCGGCTAATTTGGCAGAGTTTCACGCGAAGATTCCGATAAAGGACGCGGTGATCGCGGTGCCGCCGCACATGGGACAGGCGGAGCGGAGAGGGTTGCTTGCGGCGGCGCAGTTAGCGGGGATTAACGTTTTGTCTCTGATAAACGAGCATTCCGGCGCGGCGCTGCAGTACGGGATCGACAAGGACTTCTCGAACGAGTCTCGGCACGTGATCTTCTACGACATGGGCGCGAGCAGCAGCTACGCGGCGCTCGTGTACTTCTCAGCGTACAAGGGGAAGGAGTACGGGAAGAGCGTATCGGTGAATCAGTTTCAGGTGAAGGACGTGCGCTGGAACCCGGAGCTCGGTGGCCAGCATATGGAGCTTCGGTTGGTCGAGTATTTTGCGGATCAGTTCAATGCACATGTTGGAGGTGGAATCGATGTCCGGAAGTTCCCCAAGGCTATGGCTAAGTTGAAGAAACAGGTTAAAAGGACTAAAGAGATTCTTAGTGCTAACACAGCAGCTCCTATTTCAGTTGAATCGCTTCTTGATGACGTCGATTTCAGGAGCACAATAACCCGTGAGAAATTTGAAGAGCTCTGTGAAGACATTTGGGAAAAATCACTCTTACCTGTGAAAGAGGTGCTTGAGCATTCTGGCCTGTCATTGGAACAAATATATGCAGTGGAGTTGATTGGAGGTGCCACCAGAGTGCCAAAATTACAGGCTAAGCTTCAAGAATTCCTTGGGAGAAAAGAACTTGATAGGCATCTTGATGCTGATGAAGCAATAGTTCTTGGTGCAGCTCTGCATGCTGCAAATTTAAGTGATGGAATCAAATTGAACCGCAAACTAGGAATGGTTGATGGCTCCTTATATGGATTTGTGGTTGAGTTGAATGGCCCTGATCTTTTAAAAGACGAAAGCTCTAGGCAGATACTTGTACCACGAATGAAGAAAGTCCCGAGTAAGATGTTTAGATCCGTTAATCATAACAAAGATTTTGAAGTTTCACTAGCTTATGAAAGTGATAATTATTTGCCTCCTGGTGTTACCTCTCCTGAAATTGCTCAATACCAGATATCTGGTTTGACAGATGCAAGTCAGAAATACTCATCTCGGAATCTGTCATCCCCCATCAAGGCAAACATTCATTTTTCTCTTAGTAGAAGTGGAATTCTTTCTCTGGATCGGGCAGATGCTGTTATTGAAATAACAGAGTGGGTGGAAGTTCCTAGGAAGAATTTGACCATAGAGAATTCAACCATTTCATCAAATGTTTCGGCTGAATCTGCTGCTGGTAATAGTACTGAGGAAAACAACGAAAGCGTGCAAACTGATAGTGGGGTTAATAAGGCATCCAACATTAGTGCAGAGGAGCAAGCTGCCACTGAGCCTGCTACAGAGAAAAAGCTGAAAAGGCAGACTTTTAGGATAGTTGAGAAGATAACTGGATTTGGAATGTCTCTATCACAAGATTTTCTTGCTGAAGCCAAAAGAAAATTACAAGTACTAGATCAAAAAGATGCAGACAGAAAAAGAACAGCTGAGTTAAAGAATAATTTAGAAGGATATATATATACTACCAAGGAAAAGATTGAAACGCTTGAGGAGTTTGAAAAAGTTTCTACAAGTGAGGAACGCCAGTCCTTCATTGAGAAGCTTGATCAGGTTAACCCTTTTCCTTGGGTGCAAGATTGGTTGTATACAGATGGTGAAGATGCCAATGCAACAGAGTTTCAAGAGCATCTAGATCAGTTAAAAGCTGTTGGAGATCCAATTTTCTTCAGTGGTAAAGTTTTTCCTGAATTGGTTAATATCTGTTTCAGGTTAAAAGAGCTTACAACTCGGCCAGCAGCAGTTGAGCATGCTCATAAGTACATTGATGAGTTGAAACAGATTGTTCAAGAGTGGAAAGCAAAGAAGCCTTGGCTTCCACAAGAAAGAGTAGACGAGGTCATAAAAAGTTCTGAAAAATTGAAGAATTGGTTGGATGAGAAAGAAGCTGAGCAAAAGAAGACTTCTGGATTCAGTAAGCCAGCATTTACATCTGAAGAAGTATATCTGAAGGTGCTTGATCTGCAAACCAAGGTTGCCAGTATTAATAGAATTCCCAAGCCCAAACCTAAGGTTCAGAAGCCTGTAAAGAACGAAACTGAGAGCAGCAGTGCGCAGAATACAGAGACTTCTGATTCTAACTCAGCTGATAGTTCCTCTTCAAGTGATTCATCTGCCAACAGTTCAGAAGGCACAAGCAAAGAGACGGTTACTGAGCAATCTGAAGGTCACGATGAGCTATGA
