# Supplementary material for: Genome-wide analysis and expression profiling under heat and drought treatments of HSP70 gene family in soybean (Glycine max L.)
Source: Front Plant Sci. 2015 Sep 25;6:773. doi: 10.3389/fpls.2015.00773 (PMC4585176; doi:10.3389/fpls.2015.00773)
Supplement: Supplementary file 3 [file DataSheet3.DOCX]

>Glyma01g44910 TCTGTACAAAAAAATTAAAATAAATGTGATATAGATATAAAAAAAAATGCAGAGAAAAGTATTAGACATTAGACGGTTACTTTCTGGGAGGGTCTAGAACATTGAAGAGTGGTGTATTAGTATTAGTTATATGAAGCTGTCGGTTCTGCGTCTCATTCTCACATTGTGCTGCTGCGCCACTCAGGTTACTTTCACCTCTTCCACTACTCTTTTCTTATGCTAATTATTATTTTTATTTATGCTCACATTCTCACTACAAACGCAGTTTCACTTTGATGTCGAAATGTATCAAAAGATAATAAGAATTTGAACTGCTTTTATCTATCTTTTGTTTTAACCGTGTGCTTGATGAGGATTCGTTCCCTGGATGCTACTTAACTACCTTAATTAGAGAAAGAGATTCGTTGTTAGGCCCTTTGTGTTATTCAAATTCGTCGAAAGAATAATGCATTGTAAGTGCGAGTTTTTTTGCTCTAGATTGAGTGGAATTGAAATTACTCTTTATAGTAGCTTGTAGTGTACATGTTTGTAAGAAGACCCGCGAGGAATTTGTTTTGTAGTTTTTCACACCAGTACTTTTGGTGATATTCTTGGTATGTTGGATATCCCTCCTTCTCATCAACGAAGGAACTGAAATACGAACGACATCAGAATGTTTCCTATTTTACTTCGTGTGGTTGTTTGGGAAGATTATGTAACTATGTATGATTTTTTTTGTGATAAACTTACATATGAATTTACTTGTATGATGCAAATTTACTTCTAGAAACGTTATCTACACAAATTTCAAGAAAAGTCTAGTTTAACAGCTACAGAATGGATGACTCGTGCTCAATCATGAATCATCGCATGCTGATATGTTTCTTGTGTTTTTTCTTTTATATATGCTCTGTTCCTCACTTCTACGATGGTCATGTGGTTTTTGGACTAGAGTGCTCAATGTTTCCCATAATGAAAATTTCATAAAAAATAATGATAACTGAATAGACTGTTACTTTTCTAAGCTATGAACTCAAACAGTTTATAATGTTTGGGAGAAAACTCACAACTTGCTTCTCTCTGTTAAACGCAGGATGGTGGAACCTGCATATACTGTGACATCTGACAGTGAAACCACTGGTGAAGAAAAATCGTCTACTTTTCCTGAAATAGCAATTGGCATTGATATTGGCACATCACAATGTAGTGTTGCTGTGTGGAATGGCTCCCAAGTGGAGCTTTTGAAGAACACAAGGAATCAAAAGATTATGAAATCATATGTAACCTTCAAAGATAACATCCCTTCTGGTGGAGTTAGCAGTCAACTCTCCCATGAGGACGAGATGTTGTCTGGAGCCACGATTTTCAACATGAAACGCTTGATTGGAAGAGTTGATACTGACCCTGTTGTCCATGCATGTAAGAATCTCCCATTTCTAGTGCAGACTTTGGACATTGGCGTTCGGCCATTTATTGCCGCATTAGTGAACAATATGTGGAGATCCACGACTCCAGAAGAAGTCCTGGCAATATTTCTGGTGGAATTAAGAGCAATGGCTGAAGCTCAGCTGAAACGAAGAATAAGAAATGTGGTTCTTACCGTCCCAGTTTCATTCAGTCGATTTCAGCTAACCCGGATAGAACGTGCTTGTGCCATGGCTGGCCTTCATGTTCTCAGGTTGATGCCTGAACCAACAGCTGTGGCTTTGTTATATGGACAGCAACAACAGCAGACTTCTCATGAGAATATGGGCAGTGGAACTGAGAAAATTGCTCTCATTTTCAGTATGGGTGCTGGTTATTGTGATGTTGCTGTCACTGCTACAGCGGGTGGAGTATCACAGATTAAAGCCTTGGCAGGAAGTACCATTGGTGGTGAAGACTTGCTTCAGAATATGATGCATCATCTGCTACCAAATTCTGAAAATCTATTTAAGAACCATGGGGTCAAAGAAATTAAACAGATGGGCCTGCTTCGAGTTGCAACCCAGGATGCAATTCGCCAGCTTTCCTCTCAGACCATCGTTCAGGTTGATGTAGACCTGGGAGATGGTTTGAAGATATGCAAGGCTGTTAACCGGGAGGAGTTTGAGGAGGTAAACAGAAAGGTGTTTGAGAAATGTGAAAGCCTTATCATACAGTGTTTGCAAGATGCCAAGGTAGAAGTTGAAGAAGTAAATGATGTGATAATTGTAGGCGGATGTTCTTACATCCCGAGGGTGAAAAATCTTGTTACTAACGTATGTAAAGGCAAGGAACTTTATAAAGGCATGAATCCTTTAGAAGCTGCTGTTTGCGGTGCAGCAGTGGAAGGAGCTATTGCTTCAGGCGTCAATGATCCCTTTGGGAACTTGGACTTGTTAACTATCCAAGCTACACCTCTTGCCATTGGGATTCGAGCTGATGGGAACAAGTTTGTCCCTGTAATTCCGAGGGATACTACAATGCCAGCACGGAAGGAGCTAGTTTTCACAACTACTCATGACAATCAAACTGAGGCGTTGATCCTTGTCTATGAAGGAGAGGGTGAAAAGGCAGAAGAAAACCACCTATTGGGATATTTCAAGATAATGGGAATACCTGCTGCTCCTAAAGGAGTTCCAGAAATCAATGTGTGCATGGACATAGACGCTGCAAACGTGCTAAGAGTTTTAGCTGGTGTTGTGATGCCTGGTTCTCGCCAACCTGCGATTCCTGTTATGGAGGTAAGGATGCCAACGGTGGATGATGGGCATGGTTGGTGCGCCGAGGCTCTAAATAGAACCTATGGTGCCACACTGGATTTAGTTACTCTCCAGAAGAAGGCATGAAGTGATACACTACTACACAACATCATTGTATTTGTACCTTTTTTTTTAGTTGTTTTCTTGTGTTTGTTTCGGCAAATAGAAGTTGATGTACATGGCATGTCTGATGTTTGTATTTTGTACCTAGAATAATGGAAACAATGCGTTCCAAAAGCCTTAAGTTCAAAATGATTTCAATGGAAATTTTAAAACATGCTGACGGTAGTTGGGAAACCTTCAAC

>Glyma02g09400 CAACAAGTCATCTCTATTCATAAAATTTTATTCGATTTTTTTCTCCTCCAACTTTTAGACAACTTTTTGTTTAAAGCAAAAGACCTCAACACAAGTCAAGTCTATTGGTAGCTTCGTTGGGAAGTATTATTAGTTATAACATGTTTGGGAATGCGTTATAATTCATAATTCATGTTGAATGGAAAAAATACAGACACACTAGACAGTGAAAAGAACTACATTACTGAGCTTTCATTTTGATTCTCCAAAGAGTGTGATTGAGAAGGAAAAGGATCCAAAGAGTGTGATTGAGAAGGTAAATGTCCTGAATCAGAACCCTTGTTTATTTTGTCCCCTAAACATGTATCTATGTTCTCTGTATGACAATAGCCGTGCATCACTTAGGGTATGTTTGATAACCATTTAAAAAAAACTATTCTTAGTTTTCAAAAGCTCAAAAATAGAAAAAATAACAAAAAGAAAAACTTGTTTGATTGGTGAAATCAGTTTTTTAGTTTAGTTAAAATGGAAATAGCTACTGGTTTCCAGTTGTGACCTCCAGCTTACCTGTATCACTCCGTGAACCTTTCTTGGTTGGTGACTTGGTGCCGATGCCGGTTTTTTTTTCTACTTTTTTCTTTCTTCCTTTTCTCTTGCCTTGAGAACACAACAGTGTTTTTTGCTATTTTAACCTATCTGCTTCCATATAGAACTGAAAAGGAAAGCTTCTTTAAGCTGTTTTTCTGTTTTCTAGTTTTTAAAACTCTTTTTCAATAGATTTTGGATGATAAACTGAATGATAAGTAAAGGAAAATTGTTTTGAAAACAGCTTTTAGAAAAGCTACCTAACACTTCTTCACTGTGATACTCAAATAATCTTAGCCCAAGATAGCCAGCAAAGCTTTGGAGTTTGACATACTAACGGTGATTGTAGCCCCTGAAATTAGTACCTGAAATTGACATTGGCCCAATATTTAATCTTTACTTTAGTGCCTGTCAGTTTGATCCTATGTTTTGCAATTATGAATCCATTTAGTCCTTGATTTTGATTCCGTTCAGTTAATTTGGTTCCTAGCGAACTAAATGGATTTATTGTTGTCAAATTCAGGAATTGAATTTCTTTTTTTCATAATTTTAGGGACTAAATTAATTGGTGACTGAAATTTTAGGAACTAATTTGGTGTATTACTCATCTTTTAGCATACTTATATTTTAATTTCTACATACAAATATGATTAAACCATAATGTGCTTGTATTAGCGGGAGTCTATTGATGGGAGGTGATTCGTGACTTCTGTGTTCTGATAGAAAAGCAAGTAGTTCATTTTACAAAGTAAATGTAAACTACTTTAACTGTTAGCATCATCTAATTTTAGTATAGTTATTATGCTAATTGTCATGAAAGATTTTCTGATATGAATTGTACTACAACACTTTTGCACTTTTGATAAATTATAACAGTGGAGTTAATTGGTTACAAAGATGTAATCCACAATTCTCTTTGGCTGAAATCCAACTCTAATACTTAGGAATTACTTGTAACAACCAGAATGATTTCTTTGACTGTGCCCCATTTCTTCCGTGTCCTGACTTGAACCATTTTGATCTTGTTTCATTTACTCGAAAGATCATGGCCAAAAAATATGAGGGATGCGCAGTGGGAATCGACCTTGGTACAACTTACTCGTGTGTTGCAGTGTGGCTGGAGCAGCACTGTCGAGTGGAGATCATCCACAACGACCAAGGCAACAATACCACCCCTTCTTGTGTTGCTTTCACAGACCAACAGAGGTTGATCGGTGAAGCTGCTAAAAATCAGGCTGCCACCAACCCAGAGAACACTGTGTTTGGTAAGTTTTCTATTTTTATCAGTGCTTTCTTTTAAACATTTGCAGTTATGATTTCTCTGTGGACTTTTAGTTGGACAAATGATTGATCAGAATTTTTCAAACTTTTGTAGATGCTAAGAGGTTGATTGGTAGGAAATTTAGTGACCCCGTTATTCAAAAAGATAAAATGTTGTGGCCATTCAAGGTTGTTGCTGGTATTAATGACAAACCCATGATTTCCCTTAACTACAAGGGCCAAGAGAAACACCTTTTAGCTGAGGAAGTGTCATCTATGGTCCTCATAAAGATGCGGGAGATTGCAGAGGCATATTTGGAAACACCCGTAGAGAATGCAGTGGTTACTGTGCCTGCTTATTTCAATGACTCTCAACGTAAAGCCACCATAGATGCTGGTGCTATTGCAGGCCTCAATGTTATGCGGATAATCAATGAACCCACTGCTGCAGCTATTGCATATGGCCTTGACAAGAGAACTGATTGTGTTGAAGAGCGAAACATTTTCATCTTTGACCTTGGTGGTGGTACTTTTGATGTATCTCTCCTCACAATTAAGGATAAGGTCTTTCAAGTTAAGGCTACTGCAGGAAACACTCACCTTGGAGGGGAGGACTTTGACAACCGAATGGTGAACTACTTTGTACAGGAATTCAAGAGGAAGAACAAAGTTGACATTAGTGGGAACCCAAGAGCCCTAAGGAGGTTGAGAAGTGCATGCGAGAGGGCAAAAAGGATACTCTCATATGCAGTGACTACCAACATTGAGGTAGATGCTTTATTTCAGGGTGTTGATTTTTGCTCCTCAATCACTCGTGCAAAGTTTGAGGAAATCAATATGGAGCTCTTTGAAGAGTGTATGGAAACAGTTGATAGGTGTCTTTCTGATGCTAACATGGACAAGAGCAGTGTACATGATGTTGTCCTTGTTGGTGGTTCTTCTAGGATTCCCAAAGTGCAGGAGCTATTGCAGGGCTTCTTCGATGGGAAGGTTCTGTGCAAGAGCATCAACCCTGACGAGGCTGTTGCTTATGGTGCAGCTGTGCAGGCTGCTTTGTTGAGTAAAGGCATTGTGAATGTTCCAAACTTGGTCCTATTGGATATTACACCACTGTCTCTTGGTGTATCGGTACAAGGAGATCTCATGAGTGTGGTGATTCCTAGAAATACTACCATTCCTGTAAGGAGGACAAAAACATATGTTACAACTGAAGACAACCAATCTGCTGTCATGATTGAGGTTTATGAGGGCGAGAGAACAAGAGCGAGTGATAACAATTTGCTGGGTTTCTTCACACTTTCTGGCATTCCTCCTGCTCCTCGTGGCCATCCTTTGTATGAAACCTTTGACATAGATGAAAATGGTATTCTATCTGTTTCCGCTGAGGAAGAAAGCACCGGCAATAAGAACGAGATTACCATAACCAATGAGAAAGAAAGACTGTCAACCAAAGAAATTAAAAGAATGATTCAAGAAGCTGAATATTACAAGGCTGAAGATAAGAAATTCCTTAGGAAGGCCAAAGCAATGAATGATTTGGATTATTATGTTTACAAAATCAAGAATGCTTTAAAGAAAAAGGATATCAGCTCAAAGCTTTGCTCAAAAGAAAAGGAGAATGTCAGTTCTGCAATTGCAAGAGCCACAGATTTGCTTGAGGATAATAACCAGCAGGATGATATAGTTGTGTTTGAGGATAATCTGAAAGAGCTTGAGAGCATCATTGAACGCATGAAGGCCATGGGAAAAATTGGTTAGTTGTTGTTCAGTTGTAATTTTCTGTGGTGCTAACTTAGACTTTTGTGTGATAAATGCAAACTTTCATGATTGATGTTATCTTAAGCTCTAATAGCCGAAGTTTGATAACTTTGCAGAGGATATGTTCTTTAAATTTATTGCTATTACTGACTTGTTTTGCATAATATGAGTTGCGCTCGTACATTTCATTTTTCTTTTGCTGATTTTAGAGTTGACCTTTCTAGACCAGAGGTGTTAATAGAAACTAATTTTCTCACCTTAGTACACACTTGTTTTATTGAAAGACAATTTGGAGTATTTTAACAAATGTAAGATGTAATATGCTTCTTATAAACCCTTACGCTCACATGTTTGGAAAAATTATCCATGGTTAATTTCTAATTTTTTTTATTAGAATTTCATTTTTTTAATTTTAAGGCTTAAATACATTTTTAATTAGACTCTCATTTTTAAGCCATCATGAAGTCCAATAATCAATGAAAATGTAAAGAAATTAAGGTGTCACTTGAGATTGATTTGAATAAATTTGTAAAAGAAAAAATATCAAGTATTTGAATGAAAATGTAAAAAATAAAATGGATTCATTTATATTTTTATTCGAATAATACAATAGTTAATAACGTAGATATAGTTAATATATGTCTCCCGGTTGGATATAATCATAGGAAAAATATCATTTAAAATATACATAAGAAGGATAAATTTGAAAGACAAGAAGTTGAAACACGCGTTTGCCGTTCATATATATAAAAGAAACTACAGGAAATAACCGTGTTTTTTCTGAGCAATGAAGCAGAAGATTTTAGAATCATATCGCAACCACATTTTCTATGAAATGGGGACCTATCGAGAAGGACACAAAGTCATATTCTGCGCAAATGCATGAATTGATGTCTGAATATATATATATATATATATATAATCAAGACTAAATATTAAAATACTACTGTACAAGACAAAGTTATAAATTATTATTTGCTGAAAGAAACGTGCATCTAAGAAAGATTAATGATGTCATCTCATAAAATAGAACGCATGAAAATGCAGCTGAACTTTCCCGTAAGTCGTCATCGATCGCCAATGTACGTGTACAAGTTAAATGCAATTTTTAATCCAATTCAGCTGTCCTCAATGCAACACAGTTGCATTATTTGTACATCGAACAATTATCAATTAATATTATATTTCCTATATTATGTATACTTTTATCTCTCATTCTTTTTTATCTTCTCCATATACATTCATATAAAAAAATTCTCTCTCAATATATATTGAAAAGAATATTGTCTCTCACTTTAACGGTAAAGAAAAAGAAAAGCATTTTTTACTGCCATGTGAATTGTGATAGTGATAGTAAGATAGTTGTAATTAGAGATGTCAATTTTATGTTTATCGTTGTAATTAGGTAAAAACTAACCACAACATCATATACCTATGCATGCTTGCGAGTCCAATTAAAAGGGAAAATCACCTTCTAAAATCTAAAATAATAAATGATAAAAGGGTTACTCAGAAGGATCAGAAGCACCTATAAAAAAAAAAAAAGAAGGATCAGAAGCAATAACAAGTTGAGTTACGCGTTTTCTGTCGTATCAAACAAGATAAGGAAACAATATTAACCGACCTACCTTACTTTCCAAGTATCTAATAGTATTATTTTTAAAAAAGTAAAGCCATTACTTGCAGGTTTTGCCTATAAATAATAGTAACATTATCAATGTTTAAGAACAAAAAGGCATTTATATATAGACGTTTTTTCCTATATAACAGTAGTGGTGTTTCTGGTACGTATTTCATTTTCTTATTATCTTCACAATAATCGTAGAATTTACCTCTTTTATTCTATCACATCTTTATGAACCGTATCTATTGAGTCTCCTGTTATCTACAGTCATAGTTGAATTATATATATAGTTTGTTAATTTAATTATAATCAGTTATGACATATATATCTCAAAATTTCCAATTTTGCATTTTGAATTTTGACCATTACATTGATTTTCTATTCTTGTTGTTTTTCCTTTTGTTTCACCAGAGTTTGTGTATATTCACACACACCTGTCCTTAATTTCATCACAATCAAAAGTTACAAGAGATATAGAAATGAGTACTAAGAAGGGTTGTCCAAGCCATGCAGAAGCAATGCAATTGAAGCAACCAGGGGCACCATTCAAATGCAGTGGATGTAAACAAATGGGGTTCGGACCCAGTTACCACTGTGAAAGCAGCAACTGCAGTTACGTCCTCCATGAAGAGTGTGCAAACGCTGTTTCCATAGCCTTCCATCCCTTTTTCTCGAAGAGCAATTTCGAGTTTCATGAGAAAGCACCTGGGAAACGCACAAGGTACTGTGATGGCTGTGGAAAAGATGTGTTAGGGTTTGTGTACCACTGCTCCACCACAGGATATGATCTTCATCCATGCTGCTTGAAGCTGAAACACAACATTTCCGACCAAGAGGGGCGCGTGACGCTTGAACTGTGCCAGAAGGTTCCATCCAAGTGCGTGAAGTGTAAGCATAGGAATGTTGTGGAGAGAGTTAAAGGGTGGTCTTATGTGTCTTCTGGGGGGGATTGTTGTTACCATGTGTCTTGTGTGAAGGAACTGATTCTTGAGAATTGGAAGAAGGGTTATTTCTCTCAAGAAACCAATAATTCAATTGGGATGAGTAGTGACAGGGAGAACACTCAAGTTGCACTGAGAAGCATGGAGATTGTTCCAAGTGGAAGAAGGTCAAGGAGAATCAACAAGTACACCAAGATTGCTGTGTTGGTGTTCAAGCTAGTGGTTTCGGCTATTTTTGGAAACCCTATATCTGCCATTGCTGCTCTTGTGGAAGCCCTTGTTACTGATTGATCAGGATACCCTACGATTTTTTTTTGTTGGGACATAACTTAAATATAACTACTTATCTGTTATTTTTAGTGTTATTCTTCGATTGAAATTAGAATTTCACCTCGATAATTTAATTATGCTGACATTTAGTATAATTTTTTTATTGAAATGAAAATCTAGTTTTGATTGTTAAATTAAACATCACTAAAAGCATACTAAAGTAGTTGTATATAGGTTTTCTCTTTGTTTAATTGTTGTTGTTTGCTTTTTTAATAGTCACTGATGTTAATTAGTGTTGCTTTTTGATATTAGATTGGTGTCATATTGAATAAAGCTTCTGTAATATGTTCAATAATGATATGTGTAGAACAATAATCGT

>Glyma02g10195 ATGAGATTTATGAGCTACTTCAATGGGAAGGGTCTGTGCATGAGTATCAACCCTAATGAGGCTGTTGCTTATGGTGCAGCTATGTAGGCTGCTTTGTTGACTCAAGGCATTAAGAATGTTCCTGATTTGGTTCTGTTGGATGTTATGTCGCTGTCACTTGGTATAGCCGTAAAAGGATATCTTTTGAGTGTTGTGATTCCTAGGAATACTACTATTCCTGTAAAGAAGAAGTAAACATATGGAACATTTAAAGAAAACCTAACATCTGTTCAGATTAATGTGTATGAGGGCGAGAGAACAAGAGCTAGTGATAACAATTTACTGGGTTTTTTTAGTCTTTCTGGTTTTCCTCCTACTCCTCAGTACCATCCTTTTGATATATGCTTTGATATAGATGTGAATGGTATTTTATCTGTTTCTGCTGAGGAAAAAACCACCGGCTATAAGAATGATATTGCAATAACTAATGATGAAGGAAAATTGTCAGCAGAAGAAATTAAAAGAATGATTGAAAAAGCTGAGACTTACCAGGCTGAGGATAACAAGTTCCTTAGGAAGGCTAACGCAATGAATGCTTTGGATGATTACATTTACAAGATGAAAACGATTTTAAAGAAGGACGATATCAGCTTAAAGCTTTGCTCACAAGAAAGGCAGAAGATCAGTTTTGCAGTTACAAAGGCTACCAATTTGCTCCATGATGATAAACAACAGAATGAAGCAGTGGTGTTTGAGGATTCTCTGAAGGAGCTTGCCATTTGA

>Glyma02g10261 ATGATTGGGGGTTGTTATTTTGCAGATGCCAAGAGGTTGATTGGTAGGAGAGTTAGCGACCCTTCTGTTCATAGTGATATGAAGTTGTGGCCATTTAAGGTTATTGCTGGTGCTGGTGAGAAACCCATGATTGGTGTCAATTACAAGGGTAAGGAAAAGCAATTTTCTACTGAGGAAATCTCCTCTATGGTCCTAACAAAGATGCGGAAGATTGCAGAGGCTTACCTTGGGTCGACTGTGAAGAATGCCTTTGTTACTGTGCCTGCTTACTTCAATGATTCTCAGCGTCAAGCCAGCAAAGATGTTGGTGTCATTACTGGCCTCAATGTTATGAGAATAATAAATGAGCCAACTGTTGTTGCAATTGCATTAGGACTAGACAAGAAAGCCACTAGTGTTGGTGAGAAGAATGTCTTGATCTTTGATCTTGGAGGTGGCACCTTTGATGTTTCTTTCTTCACCATTTTGATAATAGAATGGTGAACCACTTTGTACAAGAGTTCAAAAGGAAGAACAAGAGGGAGATTACTGGAAACCCAAGAGCCTTGAGAAGGTTAAGAACTTCTTGTGAGAGAGCAAAGAGGACACTCTAATCCACTGCTCAGACCACTATTGAGATTGATTCTCTGTTTGAGGGCATTGACTTCTATTCAACCATCACTCGTGCTAGGTTTGAGGAGCTCAACATGAACCTTTTTAGGAAATGTATGGAGCCTGTGGAGAAGTGTCTTAGGGAAGCAAAGATGAGCAAGATCACCGTTCATGATGTTGTCCTCGTTGGTGGCTCTACAAGGATTCCTAAAGTTCAGCAGTTGTTGCAGGACTTCTTCAATGGGAAAGATCTATGCAAGAACATTAACCCCAATGAAGTCGCTGCTTATGGAGTTGCTGTCCAGGCAACTATATTGAGTGGTGAAGGAAATGAGAAGGTTCAAGATTTGTTGCTTTTGGATTTTACCCCATTGTCTTTGGGTTTGGAAACTGCTGGAGATGTCATGACT

>Glyma02g10320 CATTTTTGCCATGTCGAAATCATCGCCAATGACCAGGGTAACAGAACCACGCCCTCTTATGTTGGTTTCACCGATTCAGAGCGTTTGATCGGTGATGCCGCCAAGAATCAGGTCGCCATGAACCCCGTCAACACCGTCTTCGGTAAGATCCCTAGCCGACACTTCGCCTTTTCAGGATTTGCATTGTTCCTAGATTTTTGGATCTGTTGTTTGAAACTCCACTTTTCTATTTTGGTAATTTTTAGTTTTATTTTGTAATCCTGCTGTTTATATGTCTTATTGTTATTATTAATCGTTGCATGGTCTGAACTGGTTTAGAACTCTACTTGTATTGTTTGTTAAAATCTTATTTGAAATCGAATAGTAATATAATTTTAATCGAATGGTGATATGCATAAACATCGTATTTGTTCGTCGAATTCTGGTTTTGAATTGAATAATATTGTTATGCACACGCCACTCGTATTACACATTGAAATCTGATTTTGAATTGTGTAACGTAATGCCCTGTTATTTGTTTGAGGTCTAGTTTTGAATTGAATAACAGTATTACGTACACTCATATTGTTGGTTGAAATCTGATGTTGAGTTGAATAGCAATGTTGTGTGCTATTTTATTATTAAGAGTTATTTGATCTTACTATTTTGACGGAATATGGTGTGTGGATAGTTAAGATCTGTTTTTTATTGTCATTAATTAATTATCGTGAGGATTGTTTGGTGATTGATGATATGTTGTCTGATCTCAGATGCTAAGCGTTTGATTGGAAGGAGAATTTCTGATGCCTCCGTTCAGAGTGATATGAAGCTATGGCCATTTAAGGTTATCCCTGGCCCAGCTGACAAACCTATGATTGTGGTCAACTACAAGGGTGAGGACAAACAGTTCGCTGCTGAGGAAATTTCTTCCATGGTTCTCATGAAGATGCGTGAGATTGCCGAGGCTTATCTGGGTTCCACCGTGAAGAATGCTGTGGTTACTGTTCCTGCTTACTTCAATGACTCCCAGCGTCAGGCCACAAAGGATGCCGGGGTCATTGCTGGTCTCAATGTCATGCGTATCATCAATGAACCCACTGCTGCTGCCATTGCTTATGGTCTTGACAAGAAGGCCACCAGCGTGGGTGAGAAGAACGTGTTGATTTTTGACTTGGGTGGTGGTACCTTTGATGTCTCTCTTCTCACTATTGAAGAGGGTATTTTTGAGGTCAAGGCCACTGCGGGAGATACCCATCTTGGAGGTGAAGATTTTGATAACAGAATGGTTAACCATTTTGTTCAGGAGTTCAAGAGGAAGCACAAGAAGGATATCAGTGGAAACCCTAGGGCTCTTAGGAGGTTGAGGACTGCCTGTGAAAGGGCGAAGAGGACCCTTTCATCTACTGCACAGACTACAATTGAGATTGATTCTCTTTACGAGGGTGTTGACTTCTACACCACAATCACCCGAGCCAGGTTTGAGGAGCTCAACATGGATCTCTTCAGGAAGTGTATGGAGCCTGTTGAGAAGTGTTTGAGGGATGCCAAGATGGACAAGAGCACTGTCCATGATGTTGTCCTTGTTGGTGGATCCACTAGAATTCCTAAAGTCCAGCAATTGTTGCAAGATTTCTTCAATGGAAAGGAACTTTGCAAGAGCATTAACCCTGATGAGGCCGTTGCTTATGGAGCTGCTGTTCAGGCTGCTATTTTGAGTGGTGAAGGTAACGAGAAGGTGCAGGATCTGTTGCTGTTGGATGTTACACCCCTTTCTCTTGGTTTAGAAACTGCCGGTGGTGTCATGACTGTCCTCATTCCCAGGAACACTACTATCCCCACCAAGAAGGAGCAAGTGTTCTCTACTTACTCAGACAACCAACCTGGTGTCTTGATTCAGGTGTACGAGGGAGAGAGAGCTAGAACCAGGGACAACAATTTGTTGGGAAAATTTGAGCTTTCTGGCATTCCCCCAGCACCCAGGGGTGTTCCTCAGATTACTGTTTGCTTCGATATAGATGCCAATGGTATCTTGAATGTCTCTGCCGAGGACAAGACCACAGGGCAGAAGAATAAGATCACCATTACCAACGACAAGGGTAGACTCTCAAAGGAGGAAATCGAGAAGATGGTCCAGGAAGCAGAAAAATACAAGGCTGAGGATGAGGAGCACAAGAAGAAGGTTGATGCAAAGAATGCATTGGAGAACTACGCCTACAACATGAGGAACACCATTAAAGACGAGAAGATTGCATCAAAGCTCTCTGGTGACGACAAGAAGAAGATCGAAGATGCCATTGAGAGTGCTATTCAGTGGTTGGATGGAAACCAGCTGGCAGAGGCTGATGAGTTCGAAGACAAGATGAAGGAGCTTGAGAGCACTTGCAACCCAATCATTGCTAAGATGTACCAGGGTGCAGGTGCTCCCGACATGGCCGGAGGCATGGATGAAGATGTTCCTCCATCTGGTTCCGGTGGTGCTGGCCCCAAGATCGAGGAAGTTGACTAAATTATTTTATGATTCAGATGAGATGGGTTTGTGGTGGATCTATTCAGTTTTTATTTTCTTTATTTTTTGGTTTAGAATATTAGCCCATTATTTTGGTTACTCACACTAGGTATGGTGATAATGTTTTTTGGTTTTGAATAGTTCCACCTTATGGAGACTTGTGTTTACTTTTTGGCAAAGGAAACAATATTATTGCCAAATCTTGCCGTTCCTATTTAAAAGATTTCTTAAATTTCG

>Glyma02g36700 CTTAGCATGTTTTACTTCTGGAACATAATCAAACACTCCAGAAGTCTCTAGCCAGCTCTGATACTTTCTTTCGTAAATTCTCGAACTCACTCTGAAATTTCTAACGGTGACAATAAATTGCGTTCCCGGTTCTCTCTCTCCTCGCTCATAGTTACTGCACATACGAAACTACTCTACAAACACAACAAAACTCTCCAATTCTGAAGATTTTCTTATCTCTTGTCTTTGAAACTAGAAGCTCCGTGATTGATCAATGGCGACAAAAGAAGGCAAAGCCATAGGCATAGACCTCGGCACGACCTACAGCTGCGTGGGCGTGTGGCAAAACGACCGCGTTGAGATCATCCCCAACGACCAAGGCAACCGAACCACCCCCTCCTATGTCGCCTTCACCGACACCGAGAGGCTCATCGGAGACGCCGCGAAGAACCAAGTCGCGATGAACCCGCAGAACACCGTCTTCGACGCCAAGCGTTTAATCGGTCGCAGATTCTCAGACTCTCCTGTTCAAAACGACATGAAGCTGTGGCCGTTTAAGGTCGTGGCGGGACCGGGCGACAAGCCCATGATCGTGGTCAATTACAAAGGCGAGGAGAAGAAATTCTCTGCGGAGGAGATATCTTCAATGGTGTTGGTGAAGATGAGAGAAGTAGCAGAGGCGTTTCTCGGACACGCGGTGAAGAACGCGGTTATCACTGTCCCTGCTTACTTCAACGACTCTCAGAGGCAGGCGACGAAGGACGCAGGGGCAATTTCGGGTTTGAATGTGTTGAGGATTATCAATGAACCTACCGCTGCTGCCATCGCGTATGGATTGGATAAGAAGGCTTCGAGAAAAGGTGAACAGAACGTGCTTATTTTCGACCTTGGTGGTGGCACTTTTGATGTTTCAATATTGACCATCGAGGAGGGGATTTTCGAAGTGAAAGCCACTGCTGGTGATACTCATCTTGGAGGTGAAGATTTTGATAACAGAATGGTGAATCACTTTGTTTCTGAATTCAGAAGGAAGAACAAGAAGGACATCAGTGGGAATGCTAGAGCGTTGAGAAGGTTGAGGACAGCGTGTGAGAGAGCAAAGAGAACACTCTCTTCTACCGCGCAGACAACTATTGAAATCGATTCTTTATACGAAGGGATTGATTTCTATGCTACAATCACGAGGGCGAGGTTTGAGGAAATGAACATGGATTTGTTCAGGAAGTGCATGGAGCCGGTGGAGAAGTGTTTGCGTGATGCCAAGATAGACAAGAGTCATGTTCATGAGGTTGTGCTTGTTGGAGGGTCCACTAGGATCCCCAAGGTTCAGCAACTCTTGCAGGATTTCTTCAATGGGAAAGAGCTTTGCAAGAGCATTAACCCCGATGAAGCTGTTGCATACGGTGCTTCAGTTCAAGCTGCCATCTTGAGCGGTGAAGGAGACGAGAAGGTTCAGGATTTGTTGCTGCTGGATGTTACACCTCTCAGTCTCGGGCTCGAAACTGCGGGTGGTGTTATGACTGTACTAATTCCGCGGAACACTACAATTCCCACCAAGAAGGAGCAGATTTTCTCAACCTATTCCGATAACCAACCCGGGGTGTTGATCCAAGTGTTCGAAGGAGAACGAGCTAGAACAAAGGACAACAATCTTCTTGGGAAGTTTGAGCTTACTGGGATCCCTCCAGCACCAAGAGGAGTGCCTCAGATCAATGTCTGCTTCGACATCGACGCTAACGGGATTCTGAATGTCTCCGCAGAGGACAAGACTGCTGGTGTGAAGAACAAGATCACGATCACCAACGACAAGGGTAGGCTGAGCAAGGAGGAGATTGAGAAGATGTTGAAGGATGCAGAGAGGTACAAGGCGGAGGATGAAGAGGTGAAGAAGAAGGTGGAGGCGAAAAACTCGCTTGAGAACTACGCGTATAATATGAGAAACACAATAAAGGATGAGAAGATTGGAGAGAAGTTGAGCCCGGATGAGAAGGAGAAGATTGAGAAGGCTGTGGAGGATGCGATACAGTGGTTGGAGGGGAACCAGTTGGCGGAAGTGGATGAGTTTGAGGACAAGCAGAAGGAGTTGGAAGGGATCTGCAACCCCATCATTGCGAAAATGTACCAGGGTGCTGCTGCTAGACCTGGTGGAGATGTTCCTACGGGTGATGATGACATGCCTGGTGCTGGTGGTGCTGGTTCTGGCGCGGGACCTAAGATTGAAGAAGTTGACTAAGCTTGTGCATGTGACTAACTCAAAAACTTTGGTTAGGTTCTTGTTATTTGGTCTAATAATGTAAGTTTTAGTGATGATGTAAGGTTTCAGGAATCGTGAATAAGGTCTTTTTTGCTTCATGTGATGAGTGTAATATCAGAGATGGAGTTGAGATTAATGTAATTTTTTATACAGTTTGGGCTTTTAATGTATGTTTTGATTCAGAAAAACTTTGTTCCTTTCTACTTGATCGA

>Glyma03g03250 ATGACCCATAACCATAAACATCCCTACAAGCGGATGAATAAATATATTTCTAGGTTTTTTGAATAGCCTACAATACTTATAACTTAATGCTCAACTCAAATAATTATATTATTAATAAAACTCTTTCTTATTAAAAAATACAATATTATATTTTTTTAACCTATATATAATTTAGGAATGATTATTTAATTAACTAAATATTAGAAGATGACTAAATAAAAGTAAGAGTTTAATGTTTATGTACTCATTGTGTTAAATAATTTATATTGTCATTTAATTATACATCATTATTTGAATTACATTAAGATAATATTTTAAAAGTTAATAAATTTATCATGATAAATAGATTATGATTGAATATAACTTTTTTCCACTAAGAGAGTATTATCTTTTTTCTCTAAAAATCAATACCCAAAATTGAATATTGAGTATACAACTACATTAAAAATTTAGCACCAGGATTCAGTTAAAAAAAATATTTAGTGAGTTTTACTATTCATAATAATATTTTACCCCACTCTAGCGTGATTGTCTTTGGTAAAAAGATCGTCCCTTTCCGTAGAAAAACGTTGTCTGCACTCTCCAGTGCTTCATCTTCTCTGACAATGGTACTTTCTCTCTCTACATTAGGGTTTTCTTCAGCACTTTCGCGCCTAAACTTCTTCCCAAAGCCACCACCTTCTCTTTTCTCCGCACTCACCACTTTCCGCAGAAACCAACTTCCACTCAAATCGTCGCTCGCCGCATCGAAACGTGACGGCGATGTTGTCGTTTTGGGCATCGAAACCAGCTGCGACGATACCGCCGCTGCTGTCGTAAGTAATCTTGCCTTCGCTTTTTTTCATTCTTTCTTATTTTTTTGCGTTTGATGGTTTGGTTTGTCGTTTAATTTCTTTTGTGTGTGCTGTATAGGTGAGAAGTGACGGTGAAATTCTCAGCCAAGTAGTGTCTTCTCAGGTACGGTGTCGTATTGTATGTGCTGAATTTGCTTTCGTGGTTTTTCTCAGTCAAGTGTGAAAAAATTGTGGATTTTGGTGAAATTGATGCTGAATTATTCCCATTACCTGTTTTGAGTGGGGAATATTAATTGATGATTCGTTTGTGCGAGTTTGGATTGGTTAAATGAAGATAATATTTCAAGTTTGCAGTTTCCTGAATAGAGGTTTTGAAAGAGATGAAAACGATTACTTTTACAAAAAACTAAATAAATTGAAACAAACTTGAGTTTAAAAGTTGCAATTAGTATGATATATTTTTTTAGTTCTTGTTTCCTCAACACCAATTTTGTGTGTGCATGCGTGAGCTTTTGCCTTTATTAAGAAATTAATTCAAATACCTGAAACTTTATATATACTATGTTTTAAAATTAGAAAGTTTTTTTAACTAAGGAAATTTCAAAGTGGCAGTTGGTCAGAGATATCAGATGGGTGGGTGAGACTTTCTTTGAATGTGGTAATGCTATATGCATATCCCAGGCCAGAAAAAGAACATAGAAGGGGCATTCTAGGGAAGCATAATAAGATATAATAATGTGACAAAGTAAAAGCAAAAACCAAGTAAGCAAATTGCACAGGTAATTTTCTTTGTTGTTTGGTTAAAATGATTTTGATGCTTCTGAAAAGTTTTATTGATCATTTTGAGATGTGTGGGAAGAGGGTGTCAATCTGTTGTGAATTGTGATTACACTTTCTGTGTAATGATTAATCACTAATGATCTGAGTCACGTATTTTTTTATTAAGCACTTGATTTCATTGAACATGAAAATGCTTATGTTCTATTGGATGCCTTTTAGAAATATTGTTGTTTTCACTTGTTTCTTCATGAACTCAATAATGTTTGCTGAGCAAAAAAAAAAAAAAAATAATGTTAAATGTTTAATCACTTTTGTATTGGAATTGAAAACTACAGCATTTAATCTTATAAAATCTTTACAATGGGTGTAATGCATAAAAATGATCTTCCAAACATTTGATGTAATCTTTTACTGTTGTACATGTGCTCATCAGGAAAGAATATCACCTTATATTTTCCATAAAATGAAAAGTTTAAAATTTAAATAAAGAAAATAATTAGATGTCTTTAGTGTTAATTTGTTCATACATCTATAATTAGGCAGTGGTAATGATTCCTGCTACTAATAATGGATAGATCACAACCCATTGGTAGCTTCTTTCCCTTTATCCTAAAAAAATTATAAGCTTTATTCTTATTAGCCACTTGATTTTATATTCGTCATCTTTCAGCTACCTAACATACCTCTTTTTTCTTTCTAACTATGTAACTTCTCCAAATTAAGGCAGACCTGCTTGCGAAATATGGAGGTGTTGCTCCAAAAATGGCAGAAGAAGCTCACTCAAAAGTTATAGACCAGGTACATCATTCCTGTCCTTTTCTTTATATATCCCAGCTAGAACACATTTTGTATAGGTAGGTCAGGGCACGCAAGGTATGTCTTGAGTATTATTTGTTCACATACTAGTTTTTGGAACCTTTCCACGAAGTGGCCGCATACATCCATTGTAGTTCCCTTCTGTAAACTTTATTTATTTATCTGCATTCAACTATTTATTTATAAATATTGCATTGCGTATAATGCACTTCAATGCTATAAATCTTTGTAGTTCCTAGTGCTTGTCTAGGTTGCTGGTGGAATATTCCTTCAAACATAACCCTGGTGGAATGTAAGTAACTTCTGAGTTACATTGGGTGCCGTAGAATGATGTTTGGAGGGTGCCACCAGATTCTCAAATCGATTTCTAATTTGGCTTCTTATACTTTTTTCTTTGACTCTCTTTCTCTCTCTATCCCTTTCTAGAGTATTATTGGTTTGGGCTTAACCTTTTTTCTTATTCATTATAGGTTGTGCAAGAAGCCCTTGATAAAGCTTATCTGACCGAGAAGGATCTTACTGCTGTTGCTGTTACTATTGGTCCTGGTTTGAGTCTCTGCCTTCGTGGTAATGCATGAAGTCAAGAAACTTTAAGAATAGGCTTGCAAATTGTTTGAGTGAGAACACTGATCATCTTTGATCGTTATATTTAGAAGAATGAATACATTTTGTATTTAGAAGAATAATAATAAAAGATAAATATTACAGTCATAATTCATCTTGTCTTTCTTCTGCTTTTGAGGAATTATTGGTGTTAATTATGTATATCTTCCGTCTTTGTCTTTGTCTTGCTCAATGCAGTGGGGGTGCAGAAAGCCCGGAAAATTGCTGGCGGATTTAATTTACCAATTATTGGCATACATCACATGGAAGCTCATGCTCTGGTTGCCAGGTAAAGTTATAGTTCTTTACTGAATATGTTAATCTTCTACTAATAGATGGATGTGGAGGCATGCTATCAACAATTGAAATGGCTATGTATAGTCTGTTATTTCCTATTTTTAAAAACAGAAAAGCAATAAAAACTTGTTTGATAATAGTTGATTTGGAAAACTGTTTTCAAAACTAATATCTCTTAGTTTTTAAAACCAGAAAATCAAAATATCTCTTAAGTTGTTTTTGTTTTGTATTTTTAGTTTTCTATTACTCACGTCTTCTCTTATACTTTAACTTCTATTTTTTGAAAATTATTTATAGAATAGATTTTAAAACAGAAAAACAAAACTCAATCAAAGTATCAAACACACCTTTAGTTTCTTTTTATTGTTTTCTTCTACAATTTCTCTGCACCCTTTAGTGTTCCATAAGAAATTATTTTTTTTGTTGTGAGTCATAGTTATCTTTTTGTTATAGGTTAATTGAGAAAGATTTGCAGTTTCCATTCATGGCCCTACTTATTTCAGGTATCATTCCTAGTTTTGGGTTTTAGTTTTGTTAGTCAAGAAGCACTCATGCTGGAATTTTTCTTCCTCACACCTGATCTATCTTTATTCCTTGGATCATAAAATCATTGTTTCTTGTTTCTAGTTCTTTTTTCATGTTTTAAAGAATTTGGAATTTATAGCATGCTTGGGTAATAAGATCCATGTGGTATAAATAAATTAAGCCAGGTCAATTTTTGACGGTTTTGAAATTTGAATGAGTAGTATGCAGCTTGATACATCAGGAAAGTTTGATGATGTACAAAGAAATGTACAAAACTAACATATTTCACAAGGTAATGCCATTTATAAGAATGTGCTTTAGATATTCCATGTGCATGTATATTTGTGATGTATGTTAGATATATGTGGTAGCATATCATGTATATATGGTAGAATCAATCAGGAACAATCTTAGGAACACATTCTCTGAACTCATGCTCTTCATCTCTCTCTCTCTTTTTCTCTGCTTACTTTTATTCTCTCTCTTTCTCTCTGTCTTGTTCCCACTTTCCTACATTTTCTCATATATGCTTTGTTTCTTGCACTCACATGTCACCCTTCCAGACACTTTCCTAAATTTGGGCACATAATTGTGATAAATGTCATTTATGTAGAGGTGATTCTTGTGGTGTAGTTTCTGAACAGTCCAGTGTATTGCAAACTTGTGATTTGATACAGCCACTGCTGACTATATATTTTTCAGGTACAAATACACTAGGCTAACTTGTGGGAATTGTATTTATAGGGGGACACAATTTACTCGTTCTTGCTCGTGATCTTGGGCAGTACATACAACTTGGAACTACGATAGATGATGCTATCGGTGAGGCATATGACAAGACAGCAAAATGGCTCGGACTGGATTTGAGGAGAAGTGGTGGTCCTGCTATTGAGAAGCTTGCTATGGAGGGTAATGCTGAATCAGTCAAGTTTTCTGTAAGTTAGCTGCACAATATTATATTCCTCTCCCTTCCCCTAGCCCGTGATTGTTGCCTTAGGTTGAATATCTATCCTTTTCTGACATTGCCATGGCAGATGTTGTGCTTCTAGTGGAGATTTAAGTTTATTACCTTGTCATTCTAGATTCCAATGAAGCAGCACAAAGACTGCAATTTTTCCTATGCTGGTCTAAAAACCCAAGTACGTCTGGCAATTGAGTCCAAAAAGATGTAAGTAAATATTTGAAGTTGTTTGTTAATAGATTTGTTATTTTTAAAAATAAATATACGGAACAATGCTGTATCCTGATGGATAACCTGATGTAATATTTATTCCATATCCTATTAGCTTTCTCTAATGTAGTTGTTAGCAGCACAAATTTAATTTAATATAAAAACATTCTGAATTGAAGAAATGTCTGGTTGCCACATTCTGTATGGTATTATCTTTTTTCCCTCTCATTCACATCATTTCTTTTTTTTTGCTCAGCAAAAGTAGAAGTAATATTAGATGAGTACCAGAGGTACTAAGCTTACAAATAGTGATGTCAAGCACTAGGTTCCAGATCAGACTTGCTGCAGTCTTGATAGGAACCCAAGCCTGAAATCAGAATTACATAATTTGAACAAAACTGTAACATGATACTCCTACTCTAACTCATAAAAGCTAATCGAAGATTGGTCGACCAATGGTTGAAGCTTGTCTCAAAATCCTTCTCTAAGTTACGAAGCCAAGTCCATAATAAAAAGAGTGCATCATCCATGATTTTGTTAGCATTGAATAAGTCGTTGGAAAAGATAATGTTGTTCCGCTGCTGCCAAATAAACCATGTCAACGCTAGCCACCAACACTTCCACCTGTTGACCTTTACAGCCTCAGCCACCCCAAATATATGTTGAAGGAAATTATGTTTTGGGTTTTGCGGGAGAGGACCCACGCAATTCACCCAAGACATCGATTCCCACCACAGCGGACTGATTTTGCTGCAATGAAAAAACACATGACCTGTATTCTCCTCTAGATTACTGCAAAACACGCAACTCCTATCATTTAATTCCACCTGTCGTCTTCTGTGAAGGTTTGCCCTTGTAGGTAGTCGATCTCTAAGTAACCTCCAAGCGAAAGCTAATACTTTATTTGGTGCCTTTGTCTTCCATAGTTCCTCAAAAGCTGACAGCTCTGCCCCTTCTGCTGCAATCTCCCTCATCATGATGTAAGCACTTCTTGCGGAATAATGGCCACTAGGATCTGCCACCCACAACCACTCATCTTTTCTGTCTGGCTGAATTTGCTTGCTGTCCACATCGTTTAGAAAAGCGACAGCCATGCCTAACTCATTATCAAACAACGGTCTCCTCCAACTAAGCTCCCATTTCCATCCTATATCCTTACAAGCCCCCATCTGCTGAATGGTGTGATTTTGCTGACTAGAAATGAGGTACAACCTGGGATACTTTGCTGCTAATGATCTGTCTTCACCTATCCATTGATCCTCCCAAAACTTAATATGGTCACCACATCCAACCCTCCACTCTATACTCCTCGATAATGGCTCGCCATACTCCGGATGCTGTAATACCCTTTTTAAATCCCTCCACCAAATAGATTCATTGTTGGTTGTTGGTGCATCGCCCAAGCTCCTCCACCCGCCATATTTGGATTCCAAAACCCTGGCCAAAAGTGCTCCTTGCTGCTGAAATAGACTCCACTTCCATTTTGCAAGTAGTGCAATATTGAAAGTGTTTATATCCTTGATCCCCAACCCCCCTTTCTCCTTTGGAAGGCACACGGTGTCCCATTTGACCCAGGCTATCTTGTGATGATCTGCATCACCTCCCCATAAGAATTTTCTCTGTAAGCAAACCAGCTTGTCTATCACCTTTTTAGGAACAATTGAGGTAATTGGTTGCTTCAAGTTTCCACTGTTCTGACATACCAAATGCTCCAAAACTACTTTTTACAAAATTGATTTTGAGTCCCGATACAAGTTCGAAAGCCCTCAAGATTGCTTTGATGTTCATCATGGTAGCCTCCCCGAAGAATATTGTGTCATCTGCGTATTGGAGGAAGCTAATGTCCACATTGTTTGAACCCACTGGAAATCCCCTGTATAAATTACTCCCCACAGCTGTTCTTACTAGCCCATTTAAAGCTTCAGCGACGATATTAAATAACAAGGGTGCTAGTGGATCCCCTTGCCTTAGTCCTCTCTGTGGGATGAACTCCTGCGTGGGACTGCCATTCACCAGTATTGAAATAGAAGCAGACTTTAAGCATCCATCAATCCATCGAATCCATTTGTCATCGAATCCCATCCTTCTTAACATATACAGTAGAAAATTCCATGATACTGAGTCATAGGCCTTCTCATAATCGACTTTAAAAACAATGCAAGGCTTTTGGCCTTTCCTTGCTTCATCGACTACCTCGTTTGTTATCACCGCACTATGAAGCAAATGCCTTCCTTCGATGAATGCGGATTGTCTTTCATCAATGATGGAAGGCATAACCTTCATCAGACGATTGGCCAGTATCTTTGCCACTATCTTATAGATGCACCCTATCAGTGAAATAGGCCTGTAATCATTCAGAAGTTGTGGCTCCGGCACCTTCGGGAGGAGGGCTATAAATGATGCATTGGAACCTCTTGGAAAGCATTCGTTGACATGGAACTCATCTAAGAATCGGAGAACATCAGGTTTGATTAAATGCCAGAATCTTTTGATGAATTTAAAGTTAAGTCCATCTGGCCCTGGGCTTTTCTCGCTACCACATTCCCACACAGCGCTCTTCACCTCCTCTTCAGTAAAACGTGACAGTAACATCTCGTTTTGATGGTTGTCTATGGTGTGAAAGATTATGCTATCCAACCGAGGTCTGTGCTGGTCTGGTTCTTGAAACCGTTGCATAAAGAAAGATCTCACCTCCTCTTTCACCCGTGCCGGCTCTTCAGACCATGATCCATTAATCCACACTCCTCTTAGGCTATTATTTCTGCGACTAGCATTCATCATCAAATGAAAGTATCTTGTATTACAATCGCCTTCTTTGATCCATTTAGACCTTGCCTTCTGCCTTAGTAAAGACTCTTGTGATTGGGCCGCTCTCCACAAGTCTTCCTGAAGCTGCTTCCTTGTCATCATTTCATTAGGAGTCAGGTGTCTGTCGCCTGTGTTGGTCTCCAACTTGTTCAGCTCCTCCTCAATTTTCTTGTATCTCTTAAAAGTGTTACCGAACTGTTCCTTATTCCACGCTTTCAACTTTTGTTTCAGGCTTTTGATTTTTTCTTTCAACACATACCCACCCCAACCGCTTTGCTGATTAGAACTCCATGATTCTTGGACAATTTTCTTGAATGAATTATCCGAGAATCAACAATCTAAGATCCTAAAAGGTTTAGGATCCCAGTCAACGAATTTAGATCTAAGCAAAATTGGACAGTGATCAGAGAAATTTCTATCCAATGTGTGCTGGGTGCTTCCAGGCCATTTGGCTAACCACTCTGGTGACACCAGAAATCTGTCCAACTTGCTCTTAGCGGTGCCGTTTGGTCTAAACCATGTGAACTGTCTGCCCAACCATGGCACTTCCTCAACCTCTAACTCCTCAATCCATTCGTTAAACTCGCTCATGTTGCTTTCTCCTGACCCTCTATGACATATGCCCACTCTTTCTGATGATACTCTGATGTTATAAAATCTCCTAGAACACACCATAGCCCCTCTTGGCTTTGATATTTCAGCTGTTTCACATTGTCCCACAAAATTCTTTTATTCTGGATGTCACATGGTGAGTAGATGGTGATGATATGAATCAATTGCTGCTCCTGAAGCCACTCTCCTACTAACATTAAAAAACCTTGCCCAATGACCTTCCTTTATAACTTAAATCTTTTTTCACTCCACAGGCATAGAATCCCACCAGCTGAATTTGATGCCGCTTGAGCTTCCCATTTGACATCGGTGTTCCCCCACAGCGCTTGACACATGGATCTGTCTACAATCTCTTTTTTTGTCTCCTGAATACATAGCATATCTATGTGCTGTTTATTAACCATTCTTCTGATTGCTGGCCACTTAATCCCCCTCCCTAAACCTCTGACATTATATGTTATTATATTCATTGGACAATCCCCCTGCTTCCCAAGCTCTGTGCCTCTTTACGGTCTCTGTTCTCCATCTCCACTATTTTATTAATCGGCTCTGTTTGTGAAGAACCAGTAGTAACCCCCAGTGATTTTACCATCTCCCATATGTCTGCTGCCTCTTGGTTCAATAAGTGAGGCTCTGTCACTGCACACTCAGATTGTGTTGAGGAGTCCTCCGTATTGGCTATGTTTGTGTGATGTATTGTCCCACATTTGATTATGTTGTCACTCGGGACCTGCACTACATTTGGAGTGTTTGAAACTGAGTCCAGTTCTAAAGGTAATGGGATTCCAGGTGCAAGGCCCGAAATGGATTTCCTTTTGGACCACCTCTTCCGTGAATAAACTTGTGAATATAGCTGTAACTTGCTGGCTATTGGGCTAGGTGCTGGTTGTATATAGGAGGCCTTGGGGGTGTGGGTAGAAAAGCCCATAACTCCTTCTGAATTCCCCTTACAGTCACGTGTCTCATTTAAATTTGAGACTTGCCCCGTGCTTTCTTGTTGCTTCTTTTTTAAATTTCCTCTTTCAGACTCACTCACCAGCGTTACGTCAGCATTGAAGCTCCCGATCATTCACATCATTTCTAATATTTTATTATTGTAGATGTAGCCTTTACCTACAGGATAATTTGCATATTGCAAGAAGTATTATGTGATGCAGTAGTAAATTTAAATTGTGCAACATTGCAGACATCTTTTTAATTTTTATTATTACCTATTTATGAGTAACTGTATATGTGCAATTGCAGTGATGCCAAAATTCCAATTTCTTCAGCAAGTAATGGAGATCGACTGTCACGGGCTGATATTGCTGCTTCTTTTCAGGTTAGTTATACTTTTGTACATTTTGTAGATTACAAGAGTGTCTTCCCTTTCAAGTCTAGTTAATAAAGTTTAGTATCTCCCTGCTCTTGTTTAAAATTTCCTCATCTTCTCTTTCATTCACATTTAACCCATTTGTTTTAGCATGTGTCATATAACTACTATCTATTTCATTTTCTACATATCAAGCAGCGAATTGCTGTGTTACATCTTGAGGAGAGGTGTGAACGAGCAATACAGTGGGCATTGAAGATGGAGCCTTCCATAAGACACTTGGTAATTTGAAATCTAAATTACTGCCATGTCAAATAACATCAATACTCCTCCTATATATTAATTTCCATTGAAAAATCTAAATGTCATTCTATGTCTTGTTTGTTGCAGGTTGTCTCTGGTGGAGTTGCATCAAATCAATATGTCCGAGCCCGGCTTGATATGGTTGTGAAGAAGAATGGCCTGCAACTTGTATGCCCACCTCCTCGGCTCTGTACTGATAATGGTAATCATATATTCTTACCTTTGCTTTTAGTAGTGAAGGCAAACTCTATCAATACCGTGCTTCTTTGCAAACTTGACAGTCCAATGTCAGAACAAGTGAGGGTTGTATTAGACAACCAATATAAAAAAAATTGCCAATCTTTTATTACATCATATGGCTTGAGTGCAGTGTTAACTTCGCTACAACAGCTATATCGGTACTAGGATATATTGCAAAATTGGCAAGTGATGTCACTTTTTCTGGACACATGTGGATAAAGAAGTTTGTCCACAAGAATAAGATTGATGATTGAAATATTGAAATGCTAACAGTTAAATATATGGAAGTAGTTGACTGTATTATCAGAAGGAGTATTTACCCAGTGTGTCATCTAGCAACCAAGTGGGTTGGAGAAAAGGCTGAAGAGTTGGTCTATTAGACACTTCAAGATTTAAATAAGTTAATCCAAGCACATAAAAAAAGAACCAGTAACGTGGCTTTCTAACACTCCCCCTCATGCTGAAGGATACCATGTTAGAATGTGAGTTTTGACCTAATTGACCAAAAAAACTGACTTGTAAGGTGAGGATTAATTCCCACTTTTATACTTTATCTCGAGTCAATGTGGGACTTCAACACGCCCCTCTTTGCCCATGGCTACCTGCCACAACTAAGGTGAGATAGGGGTGAATTAAGGTGGCTTTCTAACAATAATGAAATAGTTCCTTCTTATTGATAGGGTACAAAAATTTACATGAATATAGTGTCTTGTGGTTGGGCTGTTTTAATGTTAGTTTTTACATTTTACCTTTCCCACTCAATATGAGGAAGAAGTGGACTTTAGGGGCTCTATGGTACTAATTAAGGGATCAAAACGTATCAGGTTATTTAGAAATCACATTTTCCCTTGAGAAGGAAACAGGTGCCAATCAAACTTGCACTGGTTCTGAAATTTCATTTACCCATATGTTAGGGCATTGACAAGATTTTCTTGTATAGATGAAATGTAATGCTCGTCCTTTTTCTCACATTCATTCTCCTTGATGCTCTTCATGAAGATAAAGAAAAAGAAATTCTCCATTTCTCAGTTGTAATAGGCTTCCAACCAGGGCCAACCTTCTCAGGAGAAATATTCACATCCAGGACACCCTATGCCCTCTTTGTGAATCTCATCAGGAGGAGGCTGGGCATTTGTTTTTCCACTGCAAGATGACAAGAGGAATGTGGGGGGAATCCATGAAATGGATCCGGGCTATTGGTGCTCTCTCAGCTGATCCGGCCAGTCATTTTATCCAATTCTGTAATGGTTTTGGTGCAGGGAGTAGGTACAGCAGGGTGTGTGGGTGGTGGATAGCCTTAACTCTTACCATTTGGCGACACAGGAATTCTTTGCTTTTCAAGGGGACCCCTTTTGATCCTCATAGAGTGATGGATGAGGCTCTATTCCTTGCCTGGTCTTGGTTAAAAGCTAGAGTCAAAGGTTTCACCACCCATTTTAATCACTGGTCTACCAATCTACAGGAGGCTTTTGGTTAATTTTGTTTTCCTTCTTGTTAGGGGACTGCTGGGTTTTATGTTTGTTGGGTTTTGCTTTTGGAAGGTGGCACCATAGGTGCCTTGTATTACTTTATTAAGTACCTCTGGTACTGATTACTTAATTTAATAATACTATTTTTGCCTTCCAAAAAAAAAAAAAAAAAAAAAAATATCTCAATTCTCTCATTTGTAGATCTTTTCGCAATCCAAATTAGTTGTTGAATGAATTTTCATTTCGTTTATCTATCTTTCTTGATCCATTGTTTAAAATACCAGATTAGGATGGTACTATTTTTTATGCAACATATCTTATTAGCTTGCTTTTGAACTGAATAGGTGTAATGATTGCTTGGACTGGTATTGAGCACTTCCGCATGGGAAGATATGACCCTCCTCCTCCTGCAGAAGAACCTGAAGACTTTGTGGTGTGTTTTCCTGAACATTGACAAATATATAGATCAAAGCATTTTTTATCAGCTGAATTTGCTTTCCCATTATGATTCTCTAGATGGATTTGAAAAATAACTTTATCTGAAAGAAAATTAAGGTTAATGCTTTTTACACAATCAATAACATCAATTGTAACCCAGGCCTTTAAAATAAATGTTTATGTCCATTTGCTTTCCTATCTACTCTCATGGGAAATACTGTTAAGATGTTTTTAACCTCTTCTAATTATTTTCTATATCCATATCCATTTCATTTTAACCTATTAGACAAATGTACTAGGCTACAGGATTTAGCAGACTTTTTCTCCATGTTCTTTGTTGATGTTATATGTTAGTAATGTAAAATTCAAACGACTTTAGTGCAACTTAGCATTGCTGAAGATTTGTTGGCTAACATGGAGTTTTTCTTCTCTAAATTTGAGTTAACCTGTGTTACATACAATGCCTATGTTTCACAAATCTGTAAATTTTGTTAAACTATGATTCATTTCAGTATGATATACGCCCAAGGTGGCCGCTGGGGGAAGAATATGCTGAAGGAAAAAGTGTAGCACGTTCGTTAAGAACAGCCCGCATTCATCCTTCTCTTACATCTATAATTCAAGCATCATTGCAACAATGACACTGCTTGAACTTCCATGTTGCTTCAGCCTCTCATGAAGATTTGCAGGGCAAGCCTCTCTCTTTTGGACTTATCTATGATTTGCCAAAATGACGTCTGGGCTGAATTTTCAAAAATACATGAAAAAGTGGGCTGAAGTTTGCTGTGGAAACTGACCATTGGCATCAAGCAGTAGCACGAGAAAATCCAGCAAAGCAATGTCACGGCAAGGAGAAACATTATAATGGTATGGTGGTTGGTAGAAGTGAAAAAAGGAGTGGGATGGGAAGAGATCAAAATTCATTGTGCTATTAACAAAAAATTTCAACTTAAGAGTAAAAGAAAAGAAAAGGCTAGGAAATTTTGTATTTATGTATTATTATTGACATATTTTGTCAATGGAATTGATTAACAAAATATTAGTTATTAATTGGCACAAATTGTAAAATATTTGTTATTATTGGCACAAATTTGATGGCCATTTAAAATAATAGATCTTACATGATTAAACAACTG

>Glyma03g17870 TGGACACCGGCCATCTCCAATATCCGTAGCAAGGCCCATCATCACATCACAACTTCCCAACCCAACCACCCAGAAAAAAACACACAAAAGCCAAATTTTGACGTTTCCTTTCCTCTTTCTTTCCCTTTTCCTCGTTTCCACGACCCTCTCGAATTCTTGAGACGTTTCCCGAATCTTCACTACGCACGTTTCTGAAACTATATAATATCTAAAATCCCTTCACTTCCCATTCCTAAAACCTCTTACAAAAACCGTACAACAAGGTTGTTTTTGTTTTGTTTTGTGTTTTCTCTCTAATCATTCGAATGGCCAAAGAAGGACCTAACTGGGACGGGTTGCTGAAATGGAGCATTGCTCACTCCGATGGGACTAGCCCTACTCGCAATTTAAGGTCCAAATTTTCAATTTTTACTTTTTTTTATTCCTTCTTCTTTTAATTGTTTTCAGTTGAAGTTGTGTTAGCCCTCTTTATGATGGTTCAGGTTAACAAAACTATGGAATTTCCTCGTTTTTATTATTTTTTGTTGCTGGGAAACGCTTACCCATGTTTCAAAATTTGATTTTTGAATGGAGAAATTTGTTATTTTTTATTGAATGTGATTGAGGGAGGTGGGGTTACTGATTAGTGGACTAGGGTGTGATTTTGTGATTTGTGGTTTTGTAATTTTTTTATTCTTTGAGGGGGAAATAGGGTTCGTATGTAAATTTATGATTGTATAGTTTATAGTTTCTATTCTTCGGGGTGGTAGTTTCTGACGTGTACTCATTTAGCAAAATTTTAATGTTTTTGAAAATAAAAAGGTGTCTTGTTGTTAAATGAGTCGGAGGGTTGGGAATAATATTCATTGGACTATGATTTGATTTTGTTAATTTTATAATTTTGCAGTTAAACAATTTATATAAGGTTTGTTTTTATTTTATTTGTAATTCTTGTTGCAAAATTGTATACCCATTTTGTGAAAAATTGAAAATTTAATACCGAAAGGGGTTGGTCTAGCTTAGTTGGTTGTGTGAGTTGCTGTGAATTTCTTGGTACTTAAAGCTTGATTCCCGTGCATAAATTTTTTTGAAAATTTAACTGTTATTTATAAAGATGCTATTTATGAATATGGAGGCTGATATTGATATAGTGGACTTGGTTTTGATTTTGTGATTTTGTAGTGAGGAGGATCGAAAATGGTTTATGGAAGCGATGCAAGCACAGACCATTGATGTTGTAAAACGTATGAAAGAGATCACACTTGTGATGCAAACTCCAGAACAAGTATTGAAAGACCAAGGAGTTACCCCTGCAGACATTGAAGGTATTCAACATTTATCAATGTGCAAGACTAATGAAAAATAAACTCCCTTTTATTTTAAGTTAATGTTTGGTTTTGTTAATCTTGTGCTAACTTTAATTTTTTGCAGATATGTTGGAAGAGTTGCAAGAGCATGTTGAGTCGATTGACATGGCCAATGGTGTGTTTTAAGGATTTTGAAGGCTTATGAATTTGACTTTGTTGATGATAGTAGGAACACTATTCATGTTATTGAAGTACTTAGTTGTCATGCTTAAGCTTGCTTATGGTAGTTTCCTGCAGGGAATTCCTACAAGTTTTTGTAGATCGTTATTTAATTTATCATTTGGATTTTTAGTTGCTGTCTCAGTGATATTTGCTGGATGATGAAGTGTCTTCATGGGCAATACAGGGGCCAATGTTGAATAAATAACAATTACTTGTTTTGAATTGTTATGATATTTACATAAATTAATGTGACAAGTTTATTTGTTCTGGCTTTGTGGTCAAGATGTTTTATTATTGTTATGCTTTTTCTGAGAATTGTTGACTCATAACTTCCCCAAATTTGTTCAATTATTTTCCATGTGATCATGGCAATAGTTCACATGATTTTTCTGCTGTTAGTCTACATTTTTCATGTTTGCATTTGCTTTAGCGTCTGTGGTATTGATAATAGGCTGGTTAATGGATTTGTGTTTTCTATGTTTCAACACCTATATGTCTCACCTATTCCCAGATCTCCACTCAATTGGTGGTTTGGTTCCTCTTCTTGGTTACCTAAAGAATTCTCATGCCAATATTCGAGCAATGGCTGCTGATGTTGTGACCACAATAGTCCAGAATAATCCTCGAAGTCAGCAACTTGTTATGGAAGCAAATGGCTTTGAACCTCTTATTTCTAATTTTAGTTCGGATCCTGACGTGACTGTTAGAACTAAAGCACTGGGTGCAATATCTTGTATGTATTATTATATTTTGGAGTTGTGATATTTTGTTACCTGAGATTGGAATGGTACTGATTTTTCTTTTGGGAACAACTTGACAGCACTAATTCGGCACAACAAACCAGGCATTACTGCATTTCGTTTGGCAAATGGGTATGCAGCTTTGAAAGATGCTCTAGCCTCTGAAAATGTGAGATTTCAAAGGTAAACAAGGTTTTCTCTGAAAAGTTTTTAATTGTGTTATTCCCTCTGAATATATCATCAATTAGGAGTAGTTTGTACCATATTATATCCTCATTATTGGTTCTATAGTTTATAGTTTAATTATAGACTTTTCAACCCTCATTATTAAATGGGAAATTGAATTCCAACTTCTGTTATTTTTATAATCTTTCAGGAAAGCTCTCAACTTGATCCATTACCTGTTGCATGAGAATAATTCAGACTGCAACATCGTGAACGAGCTTGGGTTTCCTCGAATGTTGATGCACCTTGCCTCAAGTGAAGATTCAGATGTGAGAGAAGCTGCCCTTCGTGGCCTTCTCCAGCTTGCTCACAATGCGAAAGATGGCAAGGATGGCAATGAGAAAGACAGTGTGAAAATAAAGCAACTTCTTCAAGAACGAATAAACAACATCAGTTTAATGTCAGCTGAGGACCTTGGTGTAGTCAGGGAGGAGAGGCAACTTGTGGACTCCCTGTGGAGCACTTGCTTCAACGAGCCGTCTTCTCTTCGAGAGAAAGGTCTTCTAGTGCTTCCGGGGGAGGACGTGCCCCCTCCAGATGTTGCTAGCAAATATTTTGAGCCTCCTCTTAGATCTTCGACTGCAAATCCATCTTCAAAGAAAGACCCAGAAAAGAATGAGATCCCTTTGCTTTTAGGGTCAGGACCTTCTCCTACATACACTAACAACCAAGGTTCAAATAAAGGAGATGCTAGTTCATAGACAGATGCTAATATATCGAAAGTTTAAATGCTTTATGTTCTGTTTTTTATACCATGCTTTCTTTCTATCTTATCATACAATAGTAATTTCTTTCTTTCACTTATAGGCTATGTTTTTAACCCTTTCTTCTGGCCTCAGATTGGTTTTCCCTAGTATTTCTAGGATGATGATCTCATGGTATGTTACACTGTGACACTATAACTAGCCCTCCATTGTAGTTTGTTAATGTATGATTGCAGCGGTGAATGCCTTCATTTTTTTGTGCCTTCATTTTTTTGCACTTTGATATTTTCAAA

>Glyma03g32850 AGAACATTCTGGAAGGACTCCAAACCCTAACTACACTACTATATAACCTAACTATTGCCCCTCATATCTCTCACTTGAATCACAGTGGTGCCTTATCGCTGTGGTTATCATCTTCTTCTTCCTCTTTCTTAGATCTCTTCTTTCTCTACGCCGCACGCCGAAACCATGGCCGGAAAAGGAGAGGGTCCTGCTATCGGAATCGATCTCGGAACCACCTACTCTTGCGTCGGTGTGTGGCAACATGACCGCGTTGAAATCATCGCCAACGACCAAGGGAACAGAACCACGCCGTCTTACGTCGGATTCACTGACACCGAGCGTCTCATCGGTGATGCGGCCAAGAATCAAGTCGCCATGAACCCCATCAACACCGTCTTCGGTAACCTCTCTTAACAAAATTATTTTATCTTTTCCATTCGATTGTTCTGTTTATGTATTTTTTTTATACTTATTTTAGGTTTTAGGCTAAACTATTCACTTTCTCTTTATAATTGTTTGGTCCTTAAATTTAAAATTTGCATTTTTATTCTCAATTTTGCGAAATACAAATTTTAGTTCCTCATGTGCTCAATTATCGCCGTAGACTGTACAACCAAACCGTTAACGAAGTATTTTACGATTTTGAGAACTAACAAATGTATATTTTCATTTTGGAAACTAAAAAAAGACAATCCCTAAACTTTTGAGGTACTAAAACCATATATTCTTTTTAGTTTTTAGTACACACGTACTTTTCTCGGAATGGAAGTAAAACCTAAACAGAGAAAACTATAAGGATCAAATGAATAGTTTAACCCTAGTAATATGTATACAAGACCTCTAGTTTTCAGATCTAATGTGTTATAAAAATTTGATCAATTATAAGACGCTTTTAGTATAGATCTAATCTAAATATTTTTATGAATGTTTGATTTATGTTTTATATTGCAAGAATTTAGTATAGATCTAAATTTTGTTTATCTATTATTGTGAGATGCAAAATGGCTTTTTTGTAAAGATGCTTTGTTTGTGTTGCAGATGCCAAGAGGTTGATTGGTCGTAGATTCAGTGATTCCTCTGTTCAGAGTGATATCAAATTGTGGCCTTTCAAGGTCATTCCTGGTGCTGCTGACAAGCCAATGATCGTGGTTAACTACAAGGGTGAAGAGAAGCAATTTGCCGCAGAAGAAATCTCTTCCATGGTGCTCATCAAGATGCGTGAGATTGCTGAGGCTTACCTAGGCTCCACAGTGAAGAATGCTGTTGTCACCGTCCCTGCTTACTTCAATGATTCTCAGCGTCAAGCTACCAAAGACGCTGGTGTCATTGCTGGTCTAAATGTGATGCGAATTATCAATGAGCCTACTGCAGCTGCCATTGCATATGGTCTTGATAAGAAGGCCACGAGTGTTGGTGAGAAGAATGTGTTGATTTTTGACCTTGGTGGTGGGACATTTGATGTGTCTTTACTCACCATTGAGGAGGGTATCTTTGAGGTGAAAGCCACTGCTGGTGACACCCATCTTGGAGGTGAAGATTTTGATAACAGGATGGTGAACCACTTTGTTCAAGAGTTCAAGAGAAAGAACAAGAAGGACATTAGTGGAAACCCCAGAGCACTTAGAAGGTTGAGGACTGCTTGTGAGAGGGCCAAGAGAACACTTTCATCGACTGCTCAGACCACCATTGAAATTGATTCTCTATACGAGGGAATTGATTTCTACTCCACTGTTACTCGTGCCAGATTTGAGGAGCTCAACATGGATCTCTTCAGGAAATGTATGGAGCCGGTGGAGAAATGTTTGAGGGATGCTAAGATGGACAAAAGAAGTGTTGATGATGTTGTCCTTGTTGGTGGTTCTACCAGAATTCCCAAGGTTCAACAACTGCTGCAGGACTTCTTTAATGGAAAAGAGCTTTGCAAGAGCATTAATCCCGATGAGGCTGTTGCATATGGTGCGGCTGTTCAGGCTGCAATCTTAAGTGGTGAGGGCAATGAGAAGGTTCAGGATCTTCTCCTCCTGGATGTCACCCCTCTGTCTCTTGGTTTGGAGACTGCCGGTGGTGTGATGACTGTCCTGATCCCTAGGAACACTACAATTCCAACAAAGAAGGAACAAGTTTTCTCAACATACTCTGACAACCAGCCTGGTGTGCTTATCCAGGTCTTTGAGGGTGAAAGAGCAAGGACCAGAGATAACAATTTGTTGGGCAAATTTGAGCTATCTGGCATTCCTCCTGCACCCAGGGGTGTTCCTCAGATTACAGTGTGCTTTGACATTGATGCCAATGGTATCTTGAATGTCTCTGCCGAAGATAAAACCACTGGCCAGAAAAATAAGATCACTATCACCAATGACAAGGGTAGATTGTCAAAGGAAGATATTGAGAAGATGGTTCAAGAGGCTGAGAAGTACAAGTCTGAGGATGAAGAGCACAAGAAGAAGGTTGAGGCCAAAAACGCTTTGGAAAACTATGCATACAACATGAGGAACACCGTGAAGGATGACAAGATTGGTGAGAAACTTGACCCGGCTGACAAGAAGAAGATTGAGGATGCAATTGAGCAAGCAATCCAGTGGTTAGACAGCAACCAGCTTGCAGAAGCAGATGAGTTTGAGGACAAAATGAAGGAATTGGAAAGCATCTGCAATCCAATCATTGCCAAGATGTACCAAGGTGGTGCTGGTCCAGACGTGGGTGGTGCTGGTGCAGCAGAGGATGAGTATGCTGCTCCTCCTTCTGGTGGAAGTGGTGCTGGCCCCAAGATTGAGGAAGTGGACTAAATGTAGTTGCTTTCCTCCAGATACCTGTTTTAAAAACTTATTTTACTTTATGCTTTTTTAATTGGATTTTAATCGCATGATTTTTGTTGGATGGGATTCAAACTATTTTTCCCTTCTGATATGAACGTTGCTTTAAGCATTATGTTATTTGCGTTATTTTCATTATTAGATGGTATACTCAATTGTTATGCACTTTACCTGTAGGAATTATTCTGTCTTGTTAAATGACATATCTTGTCTTTAGTCATCATATGTGTATGTTTTATTGGCAAGTTATTCTCTTTAAGTATACACGATAATTCAAGTTTTTAATGCTTGAAAAATTATTTTTGTAACAATATTTTTATACATATCTTTCAGTTGTGTAATTTATTCTCAAAGCTCTCCGGTTACTAGAT

>Glyma05g03770 GAAAAGGAGAGTTGAGCTACGGTGGGCATTAATAACTAGAGTATTATTAGTATCCCCCAAACACATATCCAACCGAAGCTACTAGAAGCCCAATCCGTCACGAAAAGTCTAGACCCTGTCGTGTAACATAAAAACCACCGAAGAAAACATCACTGCGCCGCGCTGCCACACCGAACAAACACAAACATGGATGCATCCAAACTCAATCAATTGAAGCATTTCATCGAACAGTGCAAGTCCAACCCTTCCCTCCTCTCCGATCCTTCACTCTCCTTCTTCCGCGACTATCTCGAAAGGTTAAATAAATTCAATCAGCACTCGTATTTGCGTTCCAAGACCACACATTTCTTATTAGTAACTTACTTTCTTTGATTCTTCTTTTCAGTCTCGGGGCGAAACTCCCTGAATCTGCTTATTCCGAATCGGTACCCTAATAATGTTCTATAAATACTTGCTTGTCACTACTCGCGCGTTGTTGTGTGAATCTTTACGTAATATATATTTATGTTGTTTTGTGTTATGTACGGAAGACGGGCGTGGAGAGCGATGAGGACATAGAAGATGTTACGGAGGAGCAAGAGAAGGTAGAAGAAGAAGAAGAAGATGATGAAATAATTGAATCCGATGTTGAGCTCGAGGGTGAAACCTGTCAGTCTGATGATGATCCTCCACAGAAGGTTTTTATTTTATTTTACTGTTTGTAAGAAAATAATAAAAATTTAGTTGGTTTATGTTGCATTTGGGCTTCTAATTAAGGCAAATGACTGTCACGTTTCAGATGGGAGACCCCTCTGTCGAGGTCACTGAAGAGAATCGCGACGCTTCGCAGATGGCCAAAATTAAAGCCATGGATGCTATTTCTGAAGGTAATATTTCATTCTAAAGTGAATAATGCTGATGCATTTATTTGATTTATTTATTTGTTTTTACATGGTAATTGCTGGGATGTGGATTGATCCACAACCAATGCACAAGTAAAAAGGAAGTATGAAAACAATAAGAGGAGTGGTCTTATGTATCTGTGTCAATGATCCGTAGAACGCATGGGAAAAAAAATCTCGGCAGTAAGATATATTACAATTTTTGGATTGGCACGGTGATAATGTTGTGCCTTGGTTTTCTTGGATGGCTTGGGTTGTGAACTCTTGTAAATGGTAAGGCAGGCAGGAAGGAACCCTGTTGGATTCAAGGATAATTGTTTTTTAGTTTTTTCTTGTATATAATATATTTGGTACAAGGAAGCTTGTATATCTGTCTGGTTCATAGGATGTAACTGACATTATTTGTGTTGTCTGGAACCATCCCTAATGCTAGATGCATTCTTTCTTTTGTTATTTGTCATTTGTCATTTTGAAATCAATGCTAAACATATGGAGTTTGATGCTTTATATCCTCAGTTTAAGTTTATGTGACTTCTTACCAGCTAATAAATACTACCTCTTCTCAAAGTGTTTAGGTAATTCTATAACAGGTTATATGCACGAATACTCAAAGAACATGTATTGTGCACTGTCACATTGTGCAATTATTCTTCCAATCAAAGAGCAGCATTTCAAATTTCATAGAATCATCCGTAGTGTGGGGAAACACTCTTCCCTAGCTTAGAATGTGGCACCCTAGATAACAAGCAGGTGTTGATTGTGTCGTTGGATGATATTACCCTCTGTTCCTTCATGCTCATATGACTATCTTCCCTCAATCAAACACAGGTTCTGATACCACTTGTGGATGAGGATCCACACTAAACATGCAAGGAGGAGGAAATGAAAACACAAAAATAATAAGACAATTTTATGTGGTTTGGTCTAACTCACCTCTGTCCATGATCTTTAGAATGGATAAATTTTGTCCATTAACAAAATATGCTACAACTTTTGAACAACCTAAGTTGCAAACTCTCCCACTAAACTCAGCCAAAATACTCCAATTTCAAACATCTATCAAGGGTTTATGCTATTCTTTCTGGTGATATTGTTTAAAACCGTAACAGTAATTGGACATCATTTGACTATGTAATGCAGGTAAGTTGGAGGAGGCGATTGAGAACTTAACAGAAGCTATTTTACTCAATCCTACCTCTGCCATAATGTATGGAACTAGAGGTATGACTTGATTTAATTGTTCTCTTTCTACATTGTTTTTATCACCAAGATAAGGATTAATTATTAATATTTTATAAATCATTTACAGCCAGTGTTTACATCAAAATGAAGAAACCCAATGCTGCGATCCGTGATGCTAATGCTGCTTTGGAGGTTATATTCTCTCCCCTCTGTTGCATAGGCTGACAAACTGAATTACATCCTTTCTTGTCTTAAAATAGATCTGTGAATAAAGTGTATGTGTTTAATGCTTTTGTTGCATTATTCTCTGAAAGAACAAGACACTAGAGTAAACTTGTCTCAAATGTGAGCAAGTAAGTCAATATCCATTCAAAAATACTTAATGGGTTAAGATCTTGATGCATTTCAATCAAATTATTGGTAAATGCACACTTTCTCACTTGAGTAAAACTCATTTTGGGGGAGTGTTTTCCCCTATGAAATGCATGATTTTGGAAATATATAGTATCTAAGCATTTTTTTAAATTGGGGCCTTAGTTTTTTTTGGGTGGGGGTTGGGGGGGAGGGGGGTCTGAAAACCTAATGTTTCCTGTGTTACATAACTCTTTAAGCAGTCACTCCTATAAATGTCAAATCGGCCCCTTCTAAAGTTGTATTGTGTAGCAGTCCTGCCTTTTTTGTTTGTTCTGCCCCTGCTTGTGCATATGACAACTGGATTGAGGGATTATGGGTTTCAATCAGTGTTATCAATGGTGGAAGGCTAAAATTCTGCCATATAAACATGCCATTGCAGCCTATGGTGCTCTGCCATAGCAGGCCTCCCTTCATGAATTGCCTTTGGCGGTTGACAAAAAAACCTGCCATAGTGCCACCATTTAACAACACTGGTTTCAATAACATTTCCTTCTTCCTGTATGCAATGATTTTTATTTTGTATTGCCTTTTCCAGATTAATCCTGATTCTGCTAAAGGATACAAGTCACGTGGCGTAGCACGAGCAATGCTTGGTCAATGGGAAGAAGCTGCAAAGGATCTTCATGTGGCTTCAAAGTTAGACTATGATGAGGAAATAAATGCTGTACTTAAAAAGGTTTGCTTTCTTGTCCCTTTATTTCATTTATCTTCCACAGATAAACCGTAGTTAAGCCACTAGTAGTGGTCCATACAACCAGTGCAATCTGTTGTTTTTTTACAGTATGTGATGTTATCCTCATAAGTGATATGGATTCCTTGAAATGTATTGATTTACCTGTTAACATATAAGAATATATACCTTGAAATATCATTTGGTGTCTCATTGTAAATATTGGATTGCCTAGGTGGAACCAAATGCTCACAAGATTGAGGAACACCGTCGGAAGTATGAAAGGCTGCACAAAGAAAGAGAGGATAAAAAAAAGGAGCGTGAGAGGCAGCGGCGCCGTGCTGAAGCTCAGGTTCACAATTATGATATAGATATGCTAAAACAGAGCATGCCATAAAATGTCATACCCATGTAATTTAGTTGCTAATTTGTGTGTACTTTTTTAGGCTGCCTATGAGAAGGCCAAGAAGCAAGAGCAATCATCTTCCAGTAGAAATCCTGGAGGTATGCCTGGTGGGTTTCCTGGTGGCATGCCTGGGGGCTTCCCAGGGGCCGGGGGCATGCCTGGGGGCTTCCCAGGGGCCGGGGGTATGCCGGGAGGCTTCCCAGGGGCCGGGGGTATGCCTGGGGGAGGCTTCCCAGGAGCTGGTGGCATGCCTGGAGGGGTGCCTGGAAACATTGATTTTAGCAAAATCTTGAGTGTAAGTAAATCTGGACTTTGGATGTTTTTGTGTGCTTTTGAGAAAAAATCATTACAAAAAATTCCTCTCTTCCTAATGAACATGCACACAATGCTTATTCTTGTCTTGATATCAAATGTTGCTTGATAACATTTCTCAGGACCCTGAACTGATGGCGGCATTTAGTGATCCGGAGGTTATGGCTGCTCTTCAAGATGGTAATTATCAGTTCTAATGTTTGGTTCCATTCTTTTTGTATTTTAGTTAATTTGGACTTTTCTCTATTTGAGAATCTATTTGACGTTGTCAAAGCTTGAAATTTTATAAACTTCTGACTTAATTAAAACTGCAATGCCATGCGCTTGTAGATGACAAGACTGAGAAATATATAAATCCCCTGCTCGGTCTCTCTCTGTCTCACACATTTAGTAGTTCCTATCCTGTTTGGGCATAAGACTAAATGAAATTGATTGACCTGGCTACTGGTTGTGAGGAATTGAATGGGCAGTGGGACAGTTTTGAAGTATAGCTTATAAAATGCGAAAGAATGAACTTTTCCATATCCATAACCTGCTCAGGAATAAATGACCATTTGAAAAGATCTATTAGGAAACGTCAAGTTCGATAGGAAGTTGATGATGACCCAGTTACACTAAATATGTTATGTATACCCATTTTTGTTTTTACTTTTGTCCCATATTTTAGAGTTCATTGTTGCGGAGAAAATATTTGATTTGCCGGTCATTAATTCCAATTTCTCCTTTTTTTAATTTTTGTTTTTTGTGCAGTTATGAAGAACCCTGCTAATTTTGCCAAGCACCAATCAAATCCAAAGGTAGGTCCTGTAATTGCGAAAATGATGACCAAACTTGGAGGTGGTCCCAAGTGAATACTTTTAGATGCTTATAAGTTATATGAGACTTCAACATTCCAGCCCTCTGGCATCGGGTATTTAATGTTTATTGCATTTTTAATTTTAACGAAATTATTTAACTTTTTACCATTTGTTAGTCCTGTTTCTTAGGTGCCCTATTATAGCTCGTGCTGGTAGCCATGTTTATTTGTGAACAATGTAATGTTATTTTCCCGAGTGAATTTGAATGTTTAAAGTTGATATTTTTTGCATTTCAGGAAAAATGAAATAATAGTTTCTTAGCTTTGTCCAGTGATTCCTGTAGAAGTCTGGTTTATGACTGGCAGTGGTTTGAACGCACTTAATTACTAGCACCATAATTATAGATAAAGGTGTTCGTAGATTAGATTACATCAGTTAAGGAGAGAAAATTTGTTTGATCCAATCGGTTGATTTAAAATTTCATCAGATCTTATGCGATCCAATAAATTTTCG

>Glyma05g15130 TATAAAAAGGGTATGGAAGATGTTGGATTACACAAGAATCAGATGGATGAGATCGATCTTGTTGGTGGAAGCACAAGGATTCCAAAGGTACGACATCTTTTGAAGGACTACTTTGAAGGAAAAAAGCCAAACAAGGGTGTCAACCCTGATGAAGAGTTGCATATGGTGATGCAGTGCAAAGAAGCATTTTGAGTGAAGAGGGTGGGGAAGAAACCAAAGGTACCTTAGTCTGTAATCTAGCTTTTTTTATTACTTGTCCATAAGTAATAAATATTATTTATGTAAGTAGATTACTATGTTGTTCGCTTTCTAATTGTTGTGTGCTCTGGATCCAGATATCCTTCTCCTGGATGTGGCTCCCCTCCCTTTTTATTGAGTTTCAGAATCTTCTTTTGTTTTGTTGTGTCGGGTTTTCATTGTAAGGGATGGCCACTTGAATTTGCTACCCCAGGTATAATTTTATAGTTCTTTTCTTTTAACTTTGGAACTTCTAAGTCTCTAACACATTTCATTTATATTTTTGCCAAATTTGTTTTTGTTGTTTTTTTTGTTAATAACAACAAATATAAAGTGTTAAAGAATGAAAGAGAAAGTAGAGGAAATTTAGACTGCAAAGACTTATTTTTATTCAGATATGAAGCAATGTATCTATAAACATAATAAAAAAAATGGTGAACAGAGAGATAAAATCACTAACAGATCATGCCTAAAGAATAGGACTAAAACAACTTTATCCTATCGATCAACATAAGTTTTATTTTTCCTAAAAAATAACATAAAAATTTGATTTACTATAGCTTGTTAGATAGTTACAACAATCACATCTTCAACACTCCTCCTTGACTTTGGAATTGTAAACTCCAAGCATTTGTCTCAAGTATTCAAATCTGGCTTTTGGAAGTATTTTGGTCAGAATATCAACACTTTGATTCTTTGTTTTGTAGTACAGTAACTTCACTTCTCCTTCCCTTTGAACTTCTCTTAGAAGAAAAATTTTTATCTTGAAATGCTTAGTTTTTCCATAAAACACCAGATCATTAACAATTGAGATGGCAGCCTGGTTGTTCATAAAAATCTGTGTGCTTTCTTCTTGTTCCATATGCAAATCTATCATATTTTTTTTTATCCAAAGAGTCTGATTCACTGCATCAACAATAGCTACAATGTCTGCTTCTACAGTTGATTGTGCTACAACT

>Glyma05g36600 GCGAGGTGTCCCCAAGCGAAAGGACCAATTTTATTGGCCAAACAATCCTTTCGGAACCTACGATTGGTCCACGTCACCACGTATGACATGCTCGTACTTTCGAGTGAGCACTATAAATGGCGAAGTCCCCGTCGTAAACTCAATAAAACTGCAATTCGTCCATCAAGAAAGGAAAGAAAAGGTGACATGATAATGGCTCGCTCGTTTTCACGCGGGTCTCTGCTTCCTCTCGCCATCGTTTCCTTAGGTAACAACCAGATCTCATCACTTTTCTCTTTCAATTTCAAATGTACGTAGTATCCAGATCCATCTCACTGCGATTCCTTTCAACCTTGTTTAGATCCCCTATTTTCATGGATCTGATGAGATCCTGACCCTACTTGCTTAACATTCTCATCACCGCCCTCGTTTTACTGCCACTGAGTATTATCATCATGCGTCTCTCGCTCTCTCCACATCTTAGAAATGCCTCAACAATGATTAGTCGCTGTTTTTCATTTGCGTCATTATTGTATCCATAATTTCTCTAATTTCGCAAAAACAACGCATGCTGCATAAACCTTTTTTTCTTCGTTAGACTAGTCGTGTTGCGTGTCTTGTGCAACAATAAATTAATTACGTGCTTGATTGCTTATTAATTTAGTTTCTCGTATTATAATATTCCTCATCCTTGGTCACTGATTAGATTACATGATGCATGATTTGATACCTAAAACATTTTTTTTCTTTCGTTTATGTCAATCTTTGTCACGCCTCCAAATAATTGATTTCTTCCTTTTAAAATAAGATTTGCCTCCTCCACTAAAGCAAGTAAGAGTAAATAAGTTATCTAGTTGATGAGAAAATCAATTTATTGATATTTCATCACATTCATAATTCAATGCTGTGCATGTATACTTGATTATCAACCATTTATATTGTCATGAATGAATGAGACTACCTACTTTACGTGAGTCAGTTTGCCATCAATTTAACTAGTTTGAGTTTACATTGTAATTAATGTTGAGAGAGTTCATCGCAGTATGTCTATTTGTGATTTCCATTGCAAAGGAGGAAGCCACCAAGTTGGGGACGGTCATTGGGATTGATCTTGGAACAACCTATTCATGTGTTGGTGTTTACAAGAACGGCCATGTTGAAATCATAGCCAATGATCAAGGTAACCGTATCACCCCATCGTGGGTTGCTTTCACCGACAGTGAGAGACTAATTGGAGAGGCTGCCAAGAATCTGGCAGCTGTCAATCCAGAAAGGACCATCTTTGATGTCAAGAGACTTATTGGAAGAAAGTAAGACTTACTCAAGTTGTTGATTTTTGACAATTTGATTTGATGGGATCAATGGTGACTGACATGTATCCTTTTGCAGGTTTGAAGATAAGGAAGTTCAAAGAGATATGAAGCTTGTTCCTTATAAGATTGTTAACAAGGATGGAAAACCTTACATACAGGTGAAAATTAAGGATGGTGAGACCAAGGTGTTCAGCCCTGAGGAAATCAGTGCCATGATTCTGACTAAGATGAAGGAAACTGCGGAAGCATTCCTTGGGAAGAAAATTAATGATGCTGTGGTCACTGTCCCAGGTAACTGGAAGTCTGGAACAACTCATGACTTATTTCTCCTGCAATCTTGATTGTTTTCTTCCTGCTTTTGCTTTTATGCCAATTTCTCACATTTTCCTTTCAACTGTACAGCTTACTTCAATGATGCTCAGAGGCAGGCCACCAAGGATGCTGGTGTCATTGCTGGTCTCAATGTTGCTAGAATTATTAATGAACCTACTGCTGCTGCCATTGCGTATGGATTGGACAAGAAAGGTGGCGAGAAGAACATTCTTGTTTTTGACCTTGGGGGTGGAACATTTGATGTTAGTATCTTGACAATTGATAATGGTGTTTTTGAGGTTCTTGCTACAAATGGAGATACTCATCTTGGAGGTGAGAATTTTTTTTTTTTATGAGTCTTAATAAGAAGAACTTATTATGTAGAAAAATATATTATTCTTTCCACCAAATTCTCCTCTGATGTTAATGCTTGCTATGTCTCCTTCGCCTTGCCAGGTGAGGACTTTGATCAGAGAATAATGGAGTACTTCATTAAATTGATCAAGAAAAAGCATGGAAAGGATATTAGCAAGGACAGTAGAGCACTTGGCAAGCTGAGGAGAGAGGCTGAGCGTGCAAAGAGAGCTCTCAGCAGCCAGCACCAGGTCCGTGTGGAAATTGAATCACTTTTTGATGGTGTTGATTTTTCTGAGCCACTCACCCGAGCTCGGTTTGAGGAGTTGAACAATGACTTGTTCCGGAAGACCATGGGTCCAGTGAAGAAGGCTATGGAAGATGCTGGATTACAGAAGAGTCAGATTGATGAGATTGTTCTTGTTGGTGGAAGCACAAGGATTCCAAAGGTACAACAACTTTTGAAGGACTACTTTGATGGAAAGGAGCCAAACAAGGGTGTCAACCCTGATGAAGCAGTTGCCTATGGTGCTGCAGTGCAAGGAAGCATTTTGAGTGGAGAGGGTGGTGAAGAAACCAAAGGTACTACAGTCTGTAATCTAACTTCTCATTACTTGTCCAGAAATCCTAAATATTATTTATGCAAGTAGATTGCCACGCATTACACTTTCTCATTGTTGTGTGGCTCTCTGGATCCAGATATCCTTCTCTTGGATGTGGCTCCCCTCACCCTTGGAATTGAAACTGTTGGTGGGGTGATGACGAAGTTGATTCCCAGAAACACTGTTATCCCTACCAAGAAATCTCAGGTGTTCACCACCTACCAGGATCAGCAGACTACCGTCTCCATTCAGGTATGGCTCATTATCTGACTGCATAAAAGTGCACGATGCTTGTTTTTCTTCTAAATCCAAAATTCATGACTGTGTAGTGGACTTTAATAATTTGATGCTTTTTCTCAATACATAGGTTTTTGAAGGTGAGAGGAGTCTCACAAAGGATTGCCGTCTGCTTGGGAAATTTGAACTGTCTGGAATTCCTCCAGCTCCAAGGTTTATTACCTGCTTTGATGATGCAGTGTCCTCTCTCAATATTGCTCAATTTAATGTTTGTAATTCATTGGTATTTTATTTGGTAATTGAACAGGGGTACCCCTCAAATTGAAGTGACCTTCGAAGTTGATGCCAACGGCATTCTAAATGTGAAGGCAGAAGACAAGGGCACTGGTAAATCAGAAAAGATCACCATTACAAACGAAAAGGGACGTCTTAGCCAGGAAGAAATTGAGAGAATGGTTCGTGAAGCAGAGGAGTTTGCCGAGGAAGACAAGAAGGTGAAGGAGAGGATTGATGCTCGCAACAGTCTTGAAACCTATGTCTACAACATGAAAAACCAGATCGGTGACAAGGACAAGCTCGCTGACAAGTTGGAGTCTGATGAAAAGGAGAAAATTGAGACTGCAGTAAAAGAAGCATTGGAATGGCTGGATGACAACCAGAGTGTGGAGAAAGAAGAATATGAGGAGAAGCTCAAAGAGGTGGAAGCTGTTTGCAACCCAATCATCAGTGCTGTATATCAGAGATCAGGTGGAGCCCCAGGTGGTGGTGCATCAGGCGAGGAGGATGATGATTCTCATGACGAGCTCTAGAGATGTTCTTGCGTAGCAATGAATGATGAGCAGTAGTGTAGTTTAAGCGGAGTCTTAAAGTTTTTATAACAGGTGGAAGTTGAGAAAAGCTTCCCCAGCTCTTGTATTTTTGGTGCCGAAAATACTTTAAAAGATAAATGCTTGAATTTGTTTTAGGTTGGCTAGTTTTACCACTCACCTTGCGTCTCGGTTTTCCGAGATTCGTTTTATTTTCATGCTGTATGTTCCTCTGCGTGCTTTTGGCTTGTGACTTTCCCCTTAGGACTAGGGAAGACGGTTGGACTCGGAACGACAGGCGTCTACGTAATGTATGCATTAGTGTCTGTTACGTACATTGATCTTGAAATGGTGGTTTAAAATTTAAATAAAATGTTTGACAAATAATAAAATGAATCTCTAGA

>Glyma05g36620 TCAAGTTCACGAAATGACCAGGTTTCGGTAGAGTCCCTCACCACACTACATAAAAGCCTCGCTCCCTCGTGCCATTTCAAAACCCAGAAACCAATTACAAATTACACTCTTTGCCCTCGCACCTTCAACTTAACCTTTTCTACTCATAATCTTAACAACTTGCTAGAAGAAGATAAAGAAAGAGAAAAAGTGTGTGTGTGAGAGAGAGAGCAATGGCTGGCTCGTGGGCACGCCGTTCTCTGATTGTTCTGGCTATCATTTCCTTCGGTAACTTTCACCACATAATAGATCTGATCATGTTTTCTTTTAGCTCTTTGCTGTTTTTGTTTAAATGCGCAAGTGGAAAAATGTAACCAGATCTCTCTTTCTTTGAACTTTTTTTTGGATCTGTTTTGTTTGATTCGTTGTGTTACGTAGCGGTAGATCAGTGACTGAGATCTTAGACGTAGTTGCTTAGGAGGATTTCGTAAATCGTGCCATGTTCTGCTTTTTGGTGACGTTCTTTGCGAACTCGTTAGTTCCAGGATCTTTCACGTTTGAGTTTGAGTAATATGCGTTTCTCTGCATCTGAGAATTTCGTCAACAGTGATCAGTTGGAGTTGTTATTCCGATTTCCGAGGGAGCGTTTTGTTTTTGAAAATGCTAATTGTTGCAAAATACTTTTGGTAACCAACACATTTATCTCTGATTTCTGTTCAATCAGATTTTATATTTCGTAACTAACACACGTACCTTTGATTTCTTTTAAATCAGATTTTATATATTTTAAAGTTTTTTTAATCAACATTTGTTTTCAAAATTATCATTGGTCGACGTTAATGTTTCGTGCAAATATGTTTCTAACTAACTAGCAATGCTCGTTTGTTTTTTGTTTTTTAAGTTGTCGATAAAGATTTTCACTATTAAGCGGAGTGATTAGTATTAGCCATGGAAAGAAGTTTTGTTCCGTTTGTTGCTTAAGATTATTGCTTTCATTGCATGGTTTTTCAAAGGATTGACGTTTGCGTATTTGCGATTTGAATATGACATCGTGAAGTTTCCTCCGGCTGATAATTGAATACATTAGTAAAAAGTATAAAGTAGAAATGTCTTTATGGTAATTTGTTAACGGATTCATATCATTATTGTATTTTATCGTTTATTATGCTAAATAAATTTTAGTTTATCGGGCTTGTCATTTGTCAAGCACTATTATGTGCAAATGCAGAAATTAATTCCATTTTGATGAGTTTCAGTGTTGTAAAACTGCAATTATGTAACTAACATTGAGATATTTTTCCATAGGATGTTTATTTGCAATTTCCATTGCTAAGGAGGAAGCCACAAAATTGGGGACGGTCATCGGGATTGATCTTGGAACGACCTATTCATGTGTTGGTGTTTACAAGAACGGCCATGTTGAAATCATAGCCAATGATCAAGGTAACCGTATCACCCCATCGTGGGTTGCTTTCACCGACAGTGAGAGACTAATTGGAGAGGCTGCCAAGAATCTGGCAGCTGTCAACCCAGAAAGGACCATCTTTGATGTCAAGAGACTTATTGGAAGAAAGTAAGACTTACTCAAGTTGATTTTTGACAATTTGATTTGATGGGATCAATGGTGACTGACATTTATCTTTTTGCAGGTTCGAAGATAAGGAAGTTCAAAGAGATATGAAGCTTGTTCCTTATAAGATTGTCAACAAGGATGGAAAACCTTACATACAAGTGAAAATTAAGGATGGTGAGACCAAGGTGTTCAGCCCTGAGGAAATCAGTGCCATGATTCTGACTAAGATGAAGGAAACTGCGGAAGCATTCCTTGGAAAGAAAATTAATGATGCCGTGGTCACTGTCCCAGGTAACTGGAAGTCTGGAACAATTCACGACTTATTTCAACTGCAATTTGATTGTTTTCCTCCTGCTTTTGCCTTTATTTCAATATCTCACATTTTCCTTTAAAATGCACAGCTTACTTCAATGATGCTCAGAGGCAGGCCACCAAGGATGCTGGTGTCATTGCTGGTCTCAATGTTGCTAGAATTATCAATGAACCCACTGCTGCTGCCATTGCCTATGGATTGGACAAGAAAGGTGGCGAGAAGAACATTCTAGTCTTTGATCTTGGTGGTGGGACCTTTGATGTCAGTATCTTGACAATTGATAATGGTGTTTTTGAAGTTCTTGCCACAAATGGAGATACTCATCTTGGAGGTGAGTACTTTTATACACATACACACAAGCTAATGTCTGTTATTCTATGGAAATGAAATTATATAATCTTTCCTTAAATTTTTCCTTTTGTTGCTAATGTCTGCTATTCTGTTTCCCCTTTCCAGGTGAGGACTTTGATCAGAGAATAATGGAGTACTTCATTAAATTGATCAAGAAAAAGCATGGAAAGGATATTAGCAAGGACAACAGAGCACTTGGCAAGCTGAGGAGAGAGGCTGAGCGTGCAAAGAGGGCTCTCAGCAGCCAGCACCAGGTCCGCGTGGAAATTGAATCACTTTTTGATGGTGTTGATTTTTCTGAGCCACTCACCCGAGCTCGTTTTGAGGAGTTGAACAATGATTTGTTCCGGAAGACCATGGGGCCAGTGAAGAAGGCTATGGAAGATGCAGGATTACAGAAGAGTCAGATTGATGAGATTGTTCTTGTTGGTGGAAGCACAAGGATTCCAAAGGTACAACAGCTTTTGAAGGACTACTTTGATGGAAAGGAGCCAAACAAGGGTGTCAACCCTGATGAAGCAGTTGCCTATGGTGCTGCAGTGCAAGGAAGCATTTTGAGTGGAGAGGGTGGTGAAGAAACCAAAGGTACCGTAGTCTGTAATGTAGCTTTTAAATTACTTGTCCCGAAGTCATAAATATTATTTACGCAATTAGATTGCTAAGCATAACACTTTCTAATCGTTGTGTGGCAATATCTATCCAGACATCCTTCTCCTGGATGTGGCTCCCCTCACTCTCGGAATTGAAACTGTTGGTGGAGTCATGACAAAGTTGATTCCCAGAAACACTGTTATCCCAACCAAGAAATCTCAGGTGTTCACCACCTATCAGGACCAGCAGACTACAGTCTCCATTCAGGTATGGTGCCATTATTCAATTTGCATTAAAGGGCATGATGCGTGTTTTTCTTCTAAATCTAAAATGCATGACTGTAGTGAACTATGACGGGGTTGACTCGAATTCACTGCTTTTTCTTAATACAGGTTTTCGAAGGCGAGAGGAGTCTCACAAAGGATTGCCGCCTTCTTGGGAAATTTGATCTGTCTGGAATTCCTCCAGCCCCAAGGTTTGTTACCTGCTTTGATGATGCAGTGTCCTCTCTCAATATTGCTCAATTTAATGTTTGTAATTCATTGGTATTTTATTTGGTAATTGAACAGGGGTACGCCTCAAATTGAAGTGACCTTCGAAGTTGATGCCAACGGCATTCTAAATGTGAAGGCAGAAGACAAGGGCACTGGTAAATCAGAAAAGATCACCATTACAAACGAAAAGGGACGTCTTAGCCAGGAAGAAATTGAGAGAATGGTTCGCGAAGCAGAGGAGTTTGCAGAGGAAGACAAGAAGGTGAAGGAGAGGATCGATGCTCGCAACAGTCTTGAAACCTATGTATACAACATGAAGAACCAGATCAGTGACAAGGACAAGCTCGCTGACAAGTTGGAGTCTGATGAAAAGGAGAAAATTGAGACTGCTGTGAAAGAAGCACTGGAATGGCTGGATGACAACCAGAGTATGGAGAAAGAAGATTATGAAGAGAAGCTGAAGGAGGTTGAAGCCGTTTGCAACCCAATCATTAGTGCTGTGTATCAGAGATCTGGAGGAGCCCCAGGCGGTGGTGGTGCATCAGGCGAAGAAGACGAGGACGATTCTCACGATGAGCTCTAGATTAGTCGGAGTCTGTTAAATTTTTATGATAGCTGGGAATTAAGAAGTCCCCTGCGCTTGCTTTTTTGGTGGCGATGATAAGATACTTTGTGAAATTACTTGTACTTTACTTTTTTGCTTAGTTTTACGACGAAACTTCTTCAGCTGTATGAATGAGCCTTGTTTCACTTCTATTCTCTTACGCTCCTGTTTTTCGCGTGCTTCGTGCCTGTTTTGCCGACCATAAGTTTTTAAATCAAGTCTTTGTAATTTAAAACAGGAAACCTCCGTAAAATATTATTGAGCCATTTTATAAAAACTCACGTACGGATAT

>Glyma06g00310 GAAGATTCCAGAGGCGGCGTGAGTTTCGTCGCGGACGCCGTATACTCGCCCGAGGAACTGGTGGCCATGATGTTGGGTCACACGGCGAGTTTGGCGGAGTTTCACGCGAAGGTTCCTATAAAGGACGCAGTGATTGCGGTGCCACCGAATCTGGGGCAAGCCGAGCGGAGAGGATTGCTTGTGGCGGTGCAGTTTGCGGGGATTAACGTTCTGTCGTTGATAAACTAGCATTCTGGTGCGGCGCTGCAGTATGAGATCTGTCGAACGAGTCGAGGCACGTGATCTTCTATGACATGGGTTCCAGCAGTACCTATGCAGCGGTTGTGTATTTCTCGTCGTGTGGGAAGGTGAACCCGGAGCTTGGGGGTCAGCATATGGAATTGCGATTGGTGGAGTATTTTGCTGATGAGTTCAATGCACAAGTTGGTGGTGGAATAGATGTCAGGCATTTTCCCAAGGCCATGGCTACATTGAAGAAACAGGTTAAACGAAGAAAAGAAATGCTTAGTGCAAACACAGTTGCTCCTATTTCAGTTGAATCGCTTGATGATGGCGTCGACTTCGGGAGCACAATGAACCGTGAGAAATTTGAAGACCTCTGTCAAGACATTTGGGATAAATCTCTTTTGCCTGTGAAAGAGGTGCTTCAGCATTCTGGCCTGTCATTGGACCTAATATATGCACTGCAGTTGATTGGAGGTGCTACCAGAGTGCCAAAATTACAGGCTCAGCTTCAACAATTCCTTGGGAGAAAACAACTTGACAGGCATCTTGATGCTGATGAAGCAATAGTTCTTGGCTCAGCTCCGCACGCTGCAAATTTAAGTGACGGAATCAAATTGAAAAGCAAACTAGGAATACTTGATGCTTCCATGTATGGTTTTGTGGTTGAGTTGAGTGCTCCTGATCTTTCCAAAGATGAAAGCTCTAGGCAGTTACTTGTACCGCAAATGAAGAAAGTCCCCAGTAAAGATCCGTTAATCATAACAAAGATTTTGAAGTTTCATTAGCTTATGAAAGTGAGCATCATTTGCCTCCTGGTGTTACCTCTCCTGAAATTGCTCAATACCAGATATCTGGTTTGACAGATGCCAGTGAGAAATACTCATCTCGGAATCTGTCATCACTCATCAAAGCAAACATACATTTTTCTCTAGTAGGAGTGGGATTCTTTCTCTGGATCGGGCAGATGCCATTATTGAAATAACAGAGAGGGTGGAAGTTCCAAGGAAGAATATGACCATAGAGAATTCAACCATTTCATCAAATGTTTCGGCTGAATCTGCTGGTAGTAATAGTTCTGAGGAAAACATGCAAACTGATAGTGAGATTAGTAAGACATCCAACGGTAGTGCAGAGGAGCAAGCTACTGCTGCTGAGCCCGCTACAGAGGAAAAGCTGAAAAAGCGAACCTTTAGGGTACCATTAAATATTGTTGAGAAGATAACTGGACCTGGAATGCCTCTATCTCAAGATTTTCTTGCTGAAGCCAAAAGAAAATTACTAGCACTAGATGAAAAAGACGCAGACAGAAAAAGAACAACTGAGTTAAAAAATAATTTAGAAGGATAGGTGGAGATGTTTGATGCGGGAGGATGTATGATAAAGCTATGCACCTTGATGAATCCACTTGTAAAAGTGGTCCACCTTAGACTCCGTCATAAGGAAAAGATTGAAAACTTTCTACAAGTGAGGAACGCCAGTCCTTCATTGAGAAGCTTGATCAGGTTCAAGATTGGTTGTATAGAGATGGTGAAGATGCCAATGCCACAGAGTTTCAAGAGCTTCTAGATCAGTTAAAAACTGTTGGAAATCCGATTTTCTTCAGGTTGAAAGAGCTGACAGCTCGACCAGCAGCAGTTGAGCATGCTCATAGATACATTGATGAGTTGAAAGAGTGGAAAGCAAACAAGTCTTGGTTTCCACAAGAACAAGTATATGAGATTATAAAAAATTTAAGAATTGGCTGGATGACAAAGAAGCTGATCAAAAGAAGACTTCTGGATTCAGGAAGCCCGCATTTATCTCTGAAGCAGTATATTCAAAGGTGCTTGATCTGCAAAACAAGGTTTCCAGTATTAATAGAATTCCCAAGCAAAATACATGA

>Glyma07g00820 ATGAATGAAAATGTTGGATTGAATAATATAGAAGGGTAAAATTGCATTTAAGGGTTTCAACAGATGTTTCTAGAAACAGATGAGTGAGGTAGGAGCGGGACCCATCTATCTATCTATGTAGCAGTTAGCAGTGTTTCTTTGGAATCCAAAATTCCAGAGCTTTTCCGGGGATACGAGAATACAAACTACAATCTTCCCTTCACCCTCTCAGATCTAACTCATCCACCCATTCGGTTCGGTTTCCACTCCTCACCTGTTATTCAATCAAATTCATTTCTTCTTATTAGTTCATGTGCTAGCTGCTTTTCTTTTTAAATTTTCAGATTTGTTTTTTCCTGTTTTGTACGGAATTTCGATCGACCAAGATGAGCGTGGTTGGTTTCGATTTCGGTAACGAGAGTTGCGTTGTTGCGGTTGCGAGGCAGAGAGGGATTGACGTTGTGCTCAATGATGAGTCCAAGCGTGAAACGCCCGCCATTGTGTGCTTCGGTGACAAGCAACGCTTCATTGGCACTGCCGGTGCTGCCTCCACTATGATGAACCCTAAGAATTCAATCTCACAGATTAAGAGACTCATTGGTAGGAAATTCGCTGATCCCGAATTGCAGCGGGATCTTAAGTCATTGCCGTTTCTCGTCACTGAGGGGAGTGATGGGTACCCGTTGATTCATGCGCGATACATGGGTGAGGCCAAGACATTTACGCCTACCCAAGTGTTTGGAATGATGCTGTCGAATCTTAAGGAAATTGCGGAGAAGAATCTCACTACGGCAGTTGTTGATTGTTGCATTGGAATCCCGGTTTATTTCACTGATCTGCAGAGAAGGGCGGTGTTGGATGCGGCCACAATTGCTGGTCTGCACCCGCTTCGGTTGATTCACGAAATGACTGCCACTGCCTTGGCCTATGGGATTTATAAAACGGACCTTCCGGAAAATGATCAGCTGAATGTTGCGTTTGTTGATGTTGGACATGCTAGCTTGCAAGTATGCATTGCTGGATTCAAGAAGGGGCAGCTGAAAGTGTTGGCTCATTCGTATGATAGGTCTTTCGGCGGTAGGGATTTTGATGAGGTTTTGTTCCATCACTTTGCTGAGAAGTTTAAGGACGAGTACAAGATTGATGTTTTTCAAAATGCCAGGGCTTGCATAAGGCTCAGGGCTGCCTGTGAGAAGATCAAGAAGATGCTTAGTGCAAATCCTGAGGCACCTCTCAACATTGAGTGCTTGATGGATGAGAAGGATGTCCGGGGCTTCATCAAGCGAGATGAATTTGAGCAACTAAGTCTTCCAATTTTGGAACGTGTGAAGGGGCCTCTGGAGAAGGCACTTGCTGAAGCAGGTCTTACCGTCGAAAATGTGCACACGGTTGAGGTGGTTGGTTCAGGTTCTCGTGTGCCGGCCATTAACAAAATATTGACAGAGTTTTTCAAAAAGGAGCCTAGGCGGACAATGAATGCTAGTGAGTGTGTTGCTAGGGGATGTGCGTTGGAATGTGCAATTCTTAGTCCAACGTTCAAAGTACGAGAATTTCAGGTAAAGTTTTTTGCGTTGGATTGTTCTCTATTTCATTTCAATTCACAAGGACCCTCATATGCCACTGTTGGTTTTGTAGGCCTGTTTGCTTATTGTCTTTTGTACTTGTTGTAAACCGTACAGACCTTTAGGAATTACTGTATGATTTATGAATCTTAGCATGATTGGTGCTATTTTAATTACATATCTGTGGTATCTACTTGTGTTTAGGCCTTTAGGAGTTTCATGCATCAGTTTCTTCTGTGCTTTTTTACATATTTGTGAATTGTCCATATGCTAAGCTTTGCATGCGGTATGATGTTGCTATTTTTTTTCCCTTTTCATAATGTAAAGGTCAACGAAAGCCTTCCTTTCTCGATTTCTCTTTCATGGAAAAGTTCTGGTCCAGATGCACAGGACAATGGACCAGAAAATCAGCAGAGTTCCCTTGTTTTTCCCAAGGGTAATCCCATACCAAGTATCAAGGCACTGACATTCTACAGGTCAGGAACATTCTCTGTTGATGTACAATTTGGTGATGTGAGTGGGCTGCAAACACCTGCTAAGATCAGCACCTATACTGTAAGCCAGTTTCCAACTTGTTTCATTCTTTGTTACATTGTTTTTTTGGCAGTGCTGTGACTCTTTTGAGTGTAATCAGATTGGTCCTTTCCAAACTACAAATGGTGAAAAGGCAAAAGTTAAAGTGAAAGTTCGTCTGAATCTGCATGGAATTGTATCCCTTGAGTCTGCAACGGTAAGTTTTTTCTGTCTTTTTGGTACCCCTTCCCTCACCTACTATAACCAATTAACCACCCTTGTGAACTTATGATAGTATACAGAGCATATGTTCCTGCGATCTAACAACTTCTAGTTGTTTTCTTTAGCTCCTGGAAGAGGAAGAAGTTGATGTTCCAGTTAGCAAAGAAGCAGCAGGGGAAAATACTAAGATGGACATCGATGAAGTCCCAGCTGAGGCCGCTGCACCTCCTTCCTCCAATGACACTGGTGCTAATATGGAAAATGGAAAGGCTAGTATTGATGCCTCTGGGGTTGAAGATGGCATCCCTGAGAGTGGAGGTAAGCCTTTGCAAACAGATACTGATACCAAGGTAAGTACTGTTGAAAAAATATTCATGCATATGCTCGAAATGTCTTTTTCATTTTTTATGCCACTGTTGATTTTTTTAGCAATATCTTTGTTATTTATTTCAAACATGCTATTTTTTCTTCTTCTGATTTTATGGTGTTATGGTAAGTTAGATGATGGTACTATAGTTTAAGTTGTTTAAACAAATATTTTAGGCCACTGTTGATTTTTTTAGCAATATCTTTGTTATTTATTTCAAGCATGCTATTTTTTCTTCTGGTTTTATGTTGTTATGATAAGTTAGATGATGGTACTATAGTTTACGTTGTTTAAACAAATTGAAAACCAGTTTTCAGAAGAATTTCACAAATATTACAGTGTTGTTGAGTTTTAATGTGGCACTAATGTGTGTTTGCATCATCCTGTTGTTGATTTAATTTGCTAGATTTCCTGCTTCAGATTAAATGCAAGTTCTAGGGCTTGTTGACTTGAGAAAATGTTTTCTATTTTTGTTTCTTGTTTTTACTTTTAATTAAAAAATTATAACACATTGTTTTCATTTTGTTTTGTTTTTAAGGATTTGTATAAATAGTGAAAACATTTTTTTTAATTTACAAATCTTTGAAAACTGGAAACAAAGTGAAGACATGCCTTTTTTGTCATGATTTCTAAAAAACGAGAGATCAAAATATATATTTTTCTCTTACTAAAAAGACCTCATGTCTTCCATGAATGCACTGTCAGTCAGTACAATGATGTATACATATTATGTACTCTTCCTGTTGCTTTTTTCTGCTTCAGTTAGAGTTGTTCAACATGGTTAAGTGATATTTTGTAATTTGGTTGACTGGATTCTTAATGTTGTAGGTTCAGGCTCCAAAGAAAAAGGTTAAGAAAACAAACATTCCTGTAGTAGAGTTAATTTATGGAGCAATGGTGCCTGTGGATGTCCAGAAAGCACTAGAGAAGGAGTTTGAAATGGCTTTGCAAGATCGTGTGATGGAAGAAACAAAAGACAAGAAAAACGCAGTTGAGGCTTATGTTTATGACATGAGAAACAAGGTGGGCGCTATCTTTCTTGTTCTCTTACCCAGGGTCTAACATCACATACAAATGCCTGTCATATAATAATTGTTGATTTTTTTTCCTGTTATCATTTCAGCTTAATGACAAATACCAAGAGTTTGTCACTGCTTCAGAGAGAGATGATTTTACTGCTAAACTTCAGGAAGTGGAAGATTGGCTTTATGGTGAGGGTGAAGATGAAACTAAAGGTGTATATACTGCCAAGCTTGAGGAACTCAAAAAGGTATGAAGTTTTGTAGAATCCTAGCTCCCATTCCTCAGTCAAGCTGTAATATTGTACAAAGAAAAGGGATCAGAGTTCCTCTTGTTTTTTATGCACAAACATAAATGGTTTGAGGTATTTCCATGCTAGTCTTAACATAGGCATTCTTAATAGATATTTATTTTTGTGATTGCAGCATGGTGATCCAATTGATGAGCGTTACAAAGAATTCATGGAGAGGGGTACTATAATCGAACAGTTTGTCTATTGTATAAATAGTTACAGACAAGTTGCAATGTCGAATGATCCCAGATTTGAGCACATTGACATTAACGAGAAACAGAAGGTAATTGCATGTCAATTTTCTTTCCCTTGTTTCTGTGAGTGGTTGTGAATAAATCTGAAACCATTTATCTTTCCATATGTTTCTAGGTCATAAATGAATGTGTTGAAGCTGAAAAGTGGTTTAATGAGAAGCAGCAGCAGCAGAACTCACTTCCAAAATATGCCAACCCTGTACTCTTGTCAGCTGAAATAAGAAAGAAAGCTGAAGCTGTCGATAGGTATCTGTTTAAATTCTTGCTGTGGATAGGTAGTTTTTGGATTATTAGACATCATTTGGCTTAAGTATATGTTTAGTCTGTATGTATATATGTCTGATGCATTTGGTGTTAGTCATTTTTACATATTTTTTAATTGGTTTTGGTTCTCGTAATTTTTTTTAAAATAGTTCTTATTTATGATGTCACATCAGTAATTATTTATAGGTGACATAAGATGACATAGATAGCTAAGTAGTGAAGAACTATTTAAAAATTTATAAAATTTGTGAGGACCAAAATCAGTAATTTTTTTAAGGACTAAAATAAAGTTATATTTACAGAATTAAAAACACATTTTATCTTAGACTTTGTTGGTTGTCCTTTACTGTTGCATTGCTTAATGTATTCTTATAAACCAGTCAACTTTTTAAGAATATCTTTAATTCTCTTCATTATTTCTTTTTCCTGTTATTTTCATCTATGGTTTTCTGTTTAAAAAATACATATATTTTGAATAGGTTCTGCAAGCCGATTATGGCAACACCAAGGCCAACCAAGGCGACTACTCCACCAGGACCAGCAACACATCCATCTTCTCAGAGTGATGAACAGCAGCAGCAGCAGCAACCTCCTCAGGGGGATGCTGATGCCAACAGTAATGAGAATGGTGGGAATAGCAGTAGTCAGGCTGCACCAGCGTCTACTGAACCAATGGAAACTGATAAGTCTGAGAAAACAGCCTCTGCCTAATTTTTTGTTGTTGGTTTGCTCTCAAGTCTCTACTATCTGTATAAGCATTTGAGTATGAATTGGGCCCAGGAAAAGAGAATTTTGGTGGGTTGATAGTGAGTGTGGCTCCTTGGATGATTGGTGTAAGTAGAACCCTCTCGGTTGGTTGGTTGGTAACGTAAGTTTTAATTAATATTATGGTGGTTCTTTGGAATCCGGATTTTTTTCACAAAGAATTTTGAAGAAAAAACGACAATGTGGTTGGAATTGGCCAGAAGAGTTACTTTCTCTAGGTTTATATATAGATTAGTTATATAAGATTGTTAGACGATGGATGAATTGTAATGCTCGTTTCCCGTTTAATATTTGCGTTTATTTTACGTGGCATTATAACCCTTGCTATACCATTATTGGTTTCATGTTCGAGACAAGACAGTCCTGAAACGGTTCGACGATCAACTAAGTAATATAACCGGTTTGATCTGGTTGTTAGATTGGAAACGTTATAGAAGTGTGACTCGGTCAGTTTTTTTAGTGAACTGGTGGTCTAGTTGGCCAAATCAGTTTTAGTGAATCGTTGATCTGGAAGGTTTTCGAGTGAAGCAGACTTTGTGATTTTTAAAAGTATTTTTCATAAATTTTTATGAAATTTATAGATTATGATT

>Glyma07g02450 ATTCACACGTCGATGGAGAAAGTAGCGTTGCGTGTCCAAGGTGGGCAGATATAAAGAAGAATCTGGAACCAACCAGAAGCGGAGTAGACAAAAAGAACATTCTAGAATTCTGATTTTCAAAACAGAAAGTTATATAAACCCTCCCTTTCTCTCTACTCTAACACAATTCATTCGTAGCCAATTCTTATTCTTTTTCTCTCTTTCTCTCTGCTTGCGGTGCTTCACAAGCCAAATCTAACTTGTCTCTGTTTTCTCCGATCTAGTACTACTATCAATGGCCGGAAAAGGAGATGGTCCTGCTATCGGAATCGATCTCGGCACCACCTACTCCTGCGTCGGAGTGTGGCAGCACGACCGTGTCGAAATCATCGCCAATGACCAGGGTAACAGAACCACGCCGTCTTACGTCGCTTTCACTGATTCCGAGCGTTTGATCGGTGACGCCGCCAAGAACCAGGTCGCCATGAACCCCGTCAACACCGTCTTCGGTAAAATCCCTAACCGACTTTTTGCTTTATCGAGATCTGCATTGTTCCTAGATTTTTGAAACTCCACTTTTCTATTTTAGTAATTTGTAGTATTTTCCAATCCTAATGTATTATCTGGCCTATTATTTTTTTGATATAATAATAATAATAATAATAATCGTTGAATGGTTTATTATGTACACTCTTATTGTTCGTTGAAATCTGATGTTGAATTGAATAGCAATAGGCGTAGGCACTGTTAATACTTGTCGAAATGTGATTTTGAACTGAATAAATCATATTGTGTGCAATTTTATTATTTAGAGAGTTATTTGATCCTACAATTTTGAGGGAATATGGTATGCTAAAAGTTAAGATTTATCTTTTATTCTTTTTAATTTGGATTGTGCGGATTGTTTGGTGATTGGTTATTTGTTGCCTGATGTCAGATGCTAAGCGTTTGATTGGAAGGAGATTTTCTGATGCCTCCGTTCAGAGTGACATGAAGCTATGGCCATTTAAGGTCATCCCTGGTCCTGCTGACAAACCTATGATTGTGGTCAACTACAAGGGTGAGGACAAGCAGTTCTCCGCTGAGGAAATTTCTTCCATGGTTCTCATGAAGATGCGTGAGATTGCCGAGGCTTATCTCGGTTCCACAGTGAAGAATGCCGTGGTCACTGTTCCCGCTTACTTCAATGACTCCCAGCGTCAGGCCACGAAGGATGCTGGAGTCATTGCGGGTCTCAATGTCATGCGTATCATCAATGAACCCACCGCTGCTGCCATTGCTTACGGTCTTGACAAGAAGGCCACCAGTGTGGGTGAGAAGAACGTGTTGATTTTTGACTTGGGTGGCGGTACCTTTGATGTCTCTCTTCTCACCATTGAGGAGGGTATTTTTGAGGTCAAGGCCACTGCCGGAGATACTCATCTTGGAGGTGAAGATTTTGATAACAGGATGGTTAACCATTTTGTTCAGGAATTCAAGAGGAAGCACAAGAAGGACATCAATGGAAACCCTAGGGCTCTTAGGAGGTTGAGGACTGCCTGTGAAAGGGCGAAGAGGACCCTATCATCTACTGCACAAACCACAATTGAGATAGATTCTCTTTATGAGGGTGTTGACTTCTACACCACAATCACCCGTGCCAGGTTTGAGGAGCTCAACATGGATCTCTTCAGGAAGTGTATGGAGCCCGTTGAGAAGTGTTTGAGGGATGCTAAGATGGACAAGAGCACTGTCCATGACGTTGTCCTTGTTGGTGGATCCACTAGAATTCCTAAAGTGCAGCAATTGTTGCAGGATTTCTTCAATGGCAAGGAGCTTTGCAAGAGCATTAACCCCGATGAGGCCGTTGCTTATGGAGCAGCTGTACAGGCTGCTATTTTGAGTGGTGAGGGCAACGAGAAGGTGCAGGATTTGCTGTTGTTGGATGTTACACCCCTTTCTCTTGGTTTGGAAACTGCCGGCGGCGTCATGACTGTTCTCATTCCCAGGAACACGACTATTCCCACCAAGAAGGAGCAAGTGTTCTCTACTTACTCAGACAACCAACCTGGTGTCTTGATTCAGGTGTATGAGGGAGAGAGAGCTAGAACCAGGGACAACAACTTGTTGGGTAAATTTGAGCTTTCTGGCATTCCCCCAGCACCCAGGGGTGTTCCTCAGATTACTGTCTGCTTTGATATCGATGCCAATGGTATCTTGAATGTCTCTGCCGAGGACAAGACCACAGGGCAGAAGAACAAGATCACCATCACCAACGACAAGGGTAGACTATCGAAGGATGAGATTGAGAAGATGGTCCAAGAGGCGGAGAAGTACAAGGCCGAGGATGAGGAGCACAAGAAGAAGGTCGACGCAAAGAATGCTTTGGAGAACTATGCCTACAACATGAGGAACACCATTAAGGATGAGAAGATTGCATCAAAGCTTTCTGATGACGACAAGAAGAAAATCGAAGATGCCATCGAGAGTGCTATTCAGTGGTTGGATGGAAACCAGCTAGCGGAGGCTGACGAGTTCGAAGACAAGATGAAGGAGCTTGAGAGCATTTGCAACCCAATCATTGCCAAAATGTACCAGGGTGCAGGTGCTCCCGACATGGCTGGAGGCATGGATGAAGATGTTCCTCCATCTGGATCCGGTGGTGCTGGCCCCAAGATCGAGGAAGTTGATTAAATTGTTTCATTATTTAGATGGGATGGGCTTGTGTTGGATCTATTCCGTTTTTTTTTCCTTTATTTTATTTGGTCTAGAATATTGGCCCATTATTTTGGTTACTAACACCAGGTATGGTGATTGGTAATTCTTTTTTTTTGGTTTTGAATAGTTTATCCAGATTTCACCTCGATGGAGACTTGTTTAAAAATTTTTGCCAAAGAAAACAATTTTATGGGCCGTTCCTATTTAATGTTTTCTTAAATTTTGATGTTTGATATCTACGTTACTCGTGCAAATTGCGCTGTTCCTCTGGTATAGAAGCGAAAAATGTTTTTATAACAGTATTGTCTAGTTAAATGT

>Glyma07g26550 AAAGTGTTAATACATTAAGGTGTAGTTTTCTATATTTTCGTCGCACCGGGCACTGGGCACCCTCTCACGCTTGCGTATTTCTCGTTGCTTGTCTGGTTTACTTCTGTAGCACGATCAACAGCCATTGACACCGAAAGAGCGATCGAGAGTTGTTTCTGGGTTTTTATTTCCATTTCACCATTCCTTTCAAGGTAAAGAACTCTGCATTTTAAGTCTTGTTCTCTAACTGCAATTTTTTTCTTCCTATTTCTACTCTTATCACCCTGCTCACATTTTCTTCGCCACCTCTGGACCCTATTCAATTGTTTTTTTTTTTTTTTTTGTCTGTGCTTACGATCAATACTAGTGCCGAACCCTATTTATCATCCCTCCTTTGTACTATAGTCACCTTTTATTTTATTTTTGGGTAGCACTGCTTTTTGCTTGAAACACACCGTTTTGCTAACAAAACATTACTCTCAAGTCTAAGCCAACCATTTTCACTGCACGTCCTTGACCATTTCGTATAAGTCTCTTGTTCTAAATGTGAGAAATTGTGAATCCATATCAGCAATTATCACTTTTTATGTAACCCGTTACAACTTGCTATTGCCGGTTACAGTTGCTCAGTCACTTACATATTGCCCAATCCTTTTTTTTTTTTGCTTTAATATGTTTTTGTTAATATAAGTTAGTAATTTTTATTTTTGATTACTGTAAGTTCATATTTATTTTAAAATTATAGTTCTTATAAATTTATATTTTTTAATTTTAATTTTTTATTTATAATTCTCATGGATTCGCATAAAATTAAAAACATAAATTTATATGAAATAAAAACTGAGCAAAATATCTGAAAATAATTAAAATAATGTAAATTTACATGGAATAGAAATGATTTTTTTTACAAAAGACTTAGATAGAAAAAAAAGAACTTAGATAGATAAAAAAAACATATTTAACTTTTTTTTTCAACTTATGTCAGAACTTACATAAACAACTTTTTCATCTCTATTCCTCTAATTTTATTCTATTTTTTTCCCCAACTTTAAGACAACCCCCTTTTTTTTTTTAAAAGCCAAAGACCTCGAACACTAGTAGCTTCGTTGCGAAGTATTATTAGACTCTGAAAAGAACGACATTACTGAATTTCAGTTGATTGTATACTCGTGCCTAACAAACCTTTGTATTAGATAAAACACAGCTTTCATTTTGATCCTCCAAAGAGTGCGGGATTGAGAAGGAAAAAGGACCCATAGATAGATTCGGTTATTCCATTAATTTAAGGTAAATGTCCTGAATCAACACCCTTGTGTATTTTTTTTTATCTAAAACTTGCATCACTTAGGGTATGTTTGATAACCATTTTTAAAAACTATGTTTAGTTTTCAAAAGCTAAAAAGTAGAAAAAAAAACTTGTTTGATTGGTGCAATCAGTTTTTTAGTTAAGTTAAAATGGAAATAGCTACTGGTTTTCAGTTGCAGCCTCCAGCTTACCTGTGTCACTCCACCAGCTACAGAGTGTGTTTGCTATTTTAACCTATCTGCTGCCATGTAGAACTGGAAAGGAAAACTTCTTTAAGCTGTTTCTGTTTTCTAGTTTTTAAAACACTTTTTTTCTTCTGGAAACAGTTTCCAAAATTCAATAGATTTTAGATAATAAACTGATTGATAAATAAAGGAAAATTCTTTTGAAAACAGTTTTTAGAAAAGCTACTTAACACTTCTTCACTGTCATACTGAAATAATATTAGCCAAAGATAGCCAGAAAAGATTTGGAGTTCGACATACCAACGATGAGTGTAGTCCCTGAAATTAGTACCTGAAATTGACATTGGTCCAATATTTAATCTTGACTTTAGTGCCTGTCAATTTGATCCTATGTTTTCCAATTATGAATCCATTTAGTCCTTGAGTTTGATGCCTATCAATTAATTTGGTTCCTAACGAACTAAATTGATTGATTGTTGTCAAATTCAGGAATTGGTTTTCTTTTTTCATAATTTTAGGGGCTACATTAATTGGTGAATGAAATTTTAGCAACTAATTTGTTGTATTACTCATCTTTTAGCATACTTATAATTTTAACATACTTATAAATATGATTAAACCAAAGTGTGCTTGAATTAGTGGGACTTTATTCAAGGGAGGTGATTTTTTTTATTGGCTAATGTTAGTTGTTAGTTTGTTATATTTTGTTAGTGGGGGGATTTGAATCCACGACCTCTCCCTTTCTTCTTCTCCCTTCAATTACCAAGCCAACCTTATAATTTCATGGGAGATGATTTGTGACTTCTGTTTTCTGAAGGAAAATCAGGTAGTTCATTTTATAGAGTAGATGTAAACTACTTTAACTGTTAGCCTCATCTAATTTTAATATAGTTACTATGCTATTTGTAATGAAAGCATTTTTAATATGAATTGTTCTGCAAAGATGCCATCCACAATTTAAAAGCTTACATGTACATCATAATAAATCTATTTGGCTGAAACCCAACTCTAATACTTGGGAATTACTTGTAACAACCAGCATGATTTCTTTGACTGTGCTATATTTCTTCCCGGTCCTGACTTGAACCATTTTGATCTTATTTCATTTGCTTGAAAGATCATGGCCAGAGAATACGAGGGATGTGCAGTGGGAATTGACCTTGGCACAACTTACTCGTGTGTTGCAGTGTGGCTGGAGCAGCACTGTCGAGTGGAGATCATCCACAATGACCAAGGCAACAATACCACCCCTTCTTGTGTTGCTTTCACAGACCATCAAAGGTTGATTGGTGAAGCTGCTAAAAACCAGGCTGCTACCAACCCAGAGAACACTGTGTTTGGTAAGTTTTCTATTTTTATCCGTGCTTTCTTTTAAAAATTTGAAGTTTAAGATTTCTCCTAGACTTTTAGTTGGACAAATGAATGATCAGAATTTTTTAAACTTTTGTAGATGCTAAGAGGTTGATTGGTAGGAAATTTAGTGACCCTGTTATTCAAAAAGATAAAATGTTGTGGCCGTTCAAGATTGTTGCTGGTATTAATGACAAACCCATGATTTCCCTTAATTACAAGGGCCAGGAGAAACACCTTTTAGCCGAGGAAGTATCATCTATGGTCCTCACAAAGATGCGGGAGATTGCAGAGGCATATTTGGAAACACCTGTAAAAAATGCAGTGGTTACTGTGCCTGCTTATTTCAATGACTCACAACGTAAAGCCACCATAGATGCTGGTTCTATTGCAGGCCTGAATGTTATGCGGATAATCAATGAACCTACTGCTGCAGCTATTGCATATGGGCTTGACAAGAGAACTAATTGTGTTGGAGAGCGAAGCATCTTCATCTTTGACCTTGGTGGTGGTACTTTTGACGTGTCTCTCCTCATAATTAAGGATAAGGTCTTCCGAGTTAAGGCCACTGCAGGAAACACTCACCTTGGAGGGGAGGACTTTGACAACAGAATGGTGAACTACTTTGTACAAGAGTTCAAGAGGAAGAACAAAGTTGACATTAGTGGGAATGCAAGAGCCCTAAGGAGGTTGAGAAGTGCATGCGAGAGGGCAAAAAGGATACTCTCGTATGCAGTGACTACCAACATTGAGGTAGATGCTTTATTCCAGGGCATTGACTTTTGCTCCTCAATCACCCGTGCAAAATTTGAGGAAATCAATATGGAGCTCTTTGAAGAGTGTATGGAAACAGTAGATAGGTGTCTTTCTGATGCCAACATGGACAAGAGCAGTGTACATGATGTTGTCCTTGTTGGTGGTTCTTCTAGGATTCCAAAAGTGCAGGAGCTATTGCAGGACTTCTTCAATGGGAAGATTCTGTGCAAGAGCATCAACCCTGACGAGGCTGTTGCTTATGGTGCAGCTGTGCAGGCTGCTTTGTTGAGTAAAGGCATTGTGAATGTTCCAGACTTGGTCCTGTTGGATATTACACCTCTGTCTCTTGGTATATCGCTAAAAGGAGATCTCATGAGTGTGGTGATTCCTAGAAATACTACCATTCCTGTAAAGACGACAGAAACATACTCTACAGCTGTAGATAACCAATCTGCTGTCCTGATTGAGGTTTATGAGGGTGAGAGAACAAGAGCCAGTGATAACAATTTGCTGGGTTTTTTTAGGCTTTCTGGCATTCCTCCTGTTCCTCGTAACCATCTTGTGTATATTTGCTTTGCCATAGATGAAAATGGTATTCTATCTGTTTCTGCCGAGGAAAAAAGCACTGGCAATAAGAATGAGATTACCATAACCAATGACAAAGAAAGGTTATCAACCAAAGAAATTAAAAGAATGATTCAAGAAGCTGAGTATTACCAGGCTGAAGATAAGAAATTCCTTAGGAAGGCCAAAGCAATGAATGATTTGGATTGTTACGTTTACAAGATCAAGAATGCTTTAAAGCAAAAGGATATCAGCTCAAAGCTTTGCTCAAAAGAAAAGGAGGATGTTAGTTCTGCAATTACAAGAGCTACAGATTTGCTTGAAGGTAATAACCAGCAGGATGACATAGCTGTGTTTGAGGATAATCTGAAAGAGCTTGAGAGCATCATTGAACGCATGAAGGCCATGGGCAAAATTGTTTAGTTGTTGTTCAGTTGTAATTTCCTGTGGTGTTAACTTAGAATTTTGTGTGTGATAAATGCAAACTTTCATGATTGATGTTGAGCTAAAATAGCTGAAGTTTGATAACTTTGCAGAGGATGTGTTCTTAAAATTATTGCTATTATTGACTTGAGCACTTAACATTTCTGATTGATCCTTT

>Glyma07g30290 AAAAACGAAAGTATACGATACGAATACGATACCCTCAATTCCACCTCTCACGACCATCATTTCCTTCGTCAGCACAAAACCCTAAACCCTATTTTAAAGCCAAACCCCTTTCTTTCTCCAAAGCCCAATTCCAAAGCCCTCGTTTCACTTTTCCCAATCCGCCATGGCCGCCGCCACTGCATTGCTCCGCTCTCTCCGCCGCCGCGACCTTCCCTCTTCTTCTCTCTCCGCCTTCCGATCGGTACCATTCCGCCCTCTCTTTCGCATCTCCATTTTCACTCTTTCTACACGATTAAACCTCACTTCTAATTTTTCTGAGATGTTTGTTGAGTTATGTTACGAATACATCGAATCACTTTGTTTTTAGAAACTATAACTTATTGATTTTTAAGATCTATGAGACGAGGCTCTGTGTCCCGTGATTGTGGGACTTTGTTGTGAAGGTGAAATCTTCTTATGTGAAATAAGAAATAAAATTTTAACTCGTCAGTAATACTAACAGATCTTGGAATTTACCGTTTTTAGAAATGTTTTGAGAACCACACTTTTATATAAGATAGAGACACTTGTTAGTTTGAATGTCACTGATTATTAGGTTATATATTGGCTTGAAGTTTTGCTAGATAATCTGCGTTTTTTCTGTGCTGCTGACTTCATTGTAACTTTGTATTGTATACAAGTAAGAATGCTGTTTTGGATGTATGTAATCTTCTTTTTCCTCCATAATTGACATTTTTAGAACTGCCTATCCATTCGGGACCTAGTAAGTATATGAAGAATTGTTGATATCATGGCATCGTTTGTGCAATAGTGATTGTTGAACTTGTGTTGATCATATGTTAGCACTGTTCTGAAGGTAATTTTAATTTTCATTCACATGGTTGTGCAGTTGACGAGTGGCACAAAGACATCATATGTAGGTAACAAGTGGGCAAGTTTGTCACGACCGTTCAGGTGCCATTTGTTGTTCTGTTTTCATAATTGTTACACTTTATGTATAGAGAAAGATATGGGTACTTCCGATCTCAGATTTATTCATTGTGAAATTCGATCCTCGCTGCAGTTCAAAGCCTGCTGGTAATGATGTCATTGGAATTGATCTGGGTACTACCAATTCGTGTGTCTCTGTTATGGAGGGAAAGGTAAAGATTTCCTCTCTCTTAATTTGTTAGGTATGTTTTCCTTGATGGTGTCTGTACTGACTAATAGTTATTAAATTTTCAGAATCCTAAAGTTATTGAGAATTCTGAAGGAGCTCGTACAACACCATCAGTGGTTGCCTTCAACCAGAAAGCGGAGCTTCTTGTTGGTACACCAGCCAAGCGTCAGGCTGTGACTAACCCAACAAACACTCTTTTCGGAACCAAGCGTTTGATCGGTAGGCGTTTTGATGATTCTCAAACTCAGAAGGAGATGAAGATGGTTCCATACAAGATTGTTAAGGCGTCCAATGGAGATGCTTGGGTTGAAGCCAATGGGCAGCAGTATTCTCCCAGCCAAGTTGGTGCTTTTGTTCTCACCAAGATGAAGGAAACTGCTGAATCATATCTTGGAAAGTCAGTTTCAAAAGCTGTAATTACTGTACCAGCTTACTTCAATGATGCTCAGAGGCAGGCAACAAAAGATGCCGGTAGAATTGCTGGTCTTGATGTTCAGAGAATCATCAATGAGCCCACTGCCGCTGCACTTTCCTATGGGATGAACAACAAGGAGGGTCTCATTGCAGTTTTTGATCTTGGAGGTGGAACATTTGATGTGTCCATCTTAGAGATTTCTAATGGTGTTTTTGAGGTATTAACACTTGTTTATTCTATGCCTATATTTCATAGCCAGTAATTGGAAATAATGTTGGAAATTGTCACTAATAACTTTTTTGTGTCCAGGTGAAAGCAACAAATGGTGACACTTTCTTGGGAGGAGAGGATTTTGATAATGCTTTATTGGATTTTCTAGTGAACGAATTCAAAAGAACTGAGAGTATTGATCTTTCAAAGGATAAGCTTGCATTGCAAAGGCTTCGGGAAGCTGCTGAGAAAGCCAAAATAGAACTGTCTTCAACATCTCAAACAGAAATTAATCTTCCTTTCATCACTGCTGATGCATCTGGTGCAAAGCATCTGAACATCACATTGACTAGATCTAAGTTTGAGGCTTTGGTAAACCACTTGATTGAAAGGACAAAGGCACCGTGTAAGAGCTGTTTGAAGGATGCTAACATATCTATCAAGGAAGTTGATGAGGTTCTTCTTGTTGGAGGAATGACTCGTGTGCCTAAAGTCCAGGAGGTGGTTTCAGCGATCTTTGGAAAGAGTCCTAGCAAAGGAGTAAATCCTGATGAGGCAGTTGCCATGGGAGCAGCTATTCAGGGTGGTATCCTACGTGGAGATGTTAAAGAGCTACTACTCCTAGATGTCACTCCACTTTCTCTGGGTATTGAGACTTTGGGTGGTATCTTTACCAGGTTGATTAACCGCAACACCACAATTCCTACAAAAAAGAGTCAGGTACTCTTTAAATATTGACTGATCATTGTTTTGTAATTTCCAGGTTATGGGGCCAGTCTTCGTAAGTAATGTAACATTCAAATCATAAGATTATTTGCTTAAATCTTAATTGAAATCTCCCCCTCCCCCAAGGTTTGCAAATGTTCAAGGGGTAAAAAAAAATGTGCCTAAATCCCCCCCCTCCCCCCTAATTAGATTAGTAATTTTAGATGTTTAATCTTATATAATTTTGGTGAATATTCTCTATTTGGTAGAGTGTAATTCTAACTTGTGATATCCTTCAGGTGTTTTCAACAGCAGCTGACAATCAGACTCAGGTTGGTATCAAGGTGCTACAAGGTGAGAGGGAAATGGCTGTAGACAACAAATCGCTTGGAGAATTTGAGCTTGTTGGCATTCCTCCTGCCCCAAGAGGCATGCCTCAGATTGAAGTCACATTTGACATAGATGCCAATGGGATTGTTACTGTCTCTGCCAAAGACAAGTCTACTGGGAAAGAACAACAAATCACCATCCGTTCATCTGGAGGACTCTCAGAAGATGAGATTGATAAGATGGTCAAAGAAGCAGAGTTGCACGCTCAGAAAGACCAGGAAAGAAAGGCTCTCATTGACATCAGAAACAGTGCAGATACAAGCATCTACAGCATTGAGAAGAGTTTAGGTGAGTACAGAGATAAGATCCCCAGTGAAGTGGCCAAAGAGATTGAAGATGCAGTATCGGATTTGAGAACAGCAATGGCAGGAGACAATGCTGACGAAATTAAGGCAAAGCTTGATGCTGCAAACAAAGCTGTCTCCAAGATTGGAGAGCACATTTCAGGTGGTTCTAGTGGCGGTTCCTCAGCCGGAGGTTCTCAGGGTGGTGAACAGGCTCCCGAGGCCGAGTATGAGGAAGTCAAGAAATGAGACATTTAGGCTTGGTTTTGTAGCATTTGTTTCTTATTAATCATCACATAATTGGAAGAATACAAGGTTACTTATTTTGTATCTAATGTTGAAAGCCATTTTTTCTGGAAGGCTCTTTAATTTGTAGCACTTTTGGAGTCTGAAGTCCGAACACGGCTGTTTGAATAACTTCGAATTTTTTGCATAAAATTTGTTCACTACGTTTGGCAGTTGCTAGTATT

>Glyma07g32921 ATGGCTAAAGAAGGTCATAGAATTGCCATAGGAATCAATCTGGGAACAACTTACTCATGTGTTGCAGTATGGCGGGAGCATCACCGTCGAGTGGAGATAATCCACAATGATCAGGGCAACACTAGGAGTGAAGCAACTGTTGGTTGCTTCAGCTTTTTCACGGTGTCACCATGAATTTATGGACTAGAATCAATTCGTACTTGTTATCCGAACACATTATTAATCAGAATGATCAGAATTCTTTTAAGTTTGCAGATTCTAAGAGATTGATTGGTAGGAAATATAGTGATCCTGTGGTTCAAAAGGACAAGCTGTTATGGCATTCAAGGTTGTTGCCGGTACTAATGACAAACCTATGATCATCCTTAACTACAAGGGTGAGGAGAAGCACTTTTGTGCTGAGGAAAAAAATGTCTATCATCAATGGTTCTTGCGAAGATGTGGCAGATTGCAGAAGCGTTTTTGGAGAAACATGCAAAGAATGAAGTGGTTACTGTGCCTGCCTATTTCAATGATTCTCAGTATAAAGCTACCATAGATGCTGGAAAAATTGCAGGCCTAAATATTCTGCGGATAATCAATGAACCCGTTGCTGCGGCAATCATGCATGGTCTTGACATGAGGACTAATAATTGTGTTGGAGAGCGAAACATTTTCATCTTTGACCTTGGTGGTGGTACTTTTGATGCGTCTCTCCTTACTCTTAAGGGTAAGATCTTTAAAGTTAAGGCCACAGCTGGAAATGGTCACCTTGGGGGAGAGGACATTGATAATAGAATGCTGGACCATTTTGTAAAGGAGATCAAAAGAAAAAAAAAAGTGGACATTAGTGGGAACCTGAAGGTGCTGAGGAGGTTAAAAACCACGTGCGAGAGGGCAAAAAGAACACTCTCACATGCTGTTACTACCAACATTGAAGTAGATGCTTTATCTGATGCCATTGACTTCTGTTCTTCGATCACTCGTGCAAAGTTTGAGGAAATCAATATGGAGCTCTTTAAGGAGTGTATGGAGACAGTGGATAAGTGTCTTACTGATTCTAAGATGAACAAGAGCAGTGTACATGATGTTATCCTTGTTGTGGTTCTTCAAGGATTCCCAAAGTGCAAGAGCTATTGCAGGACTTTTCCAACGGAAAGGATCTGTGAAAGAGCATCAACCCTGATGAAGCTGTTGCTTATGGTGCAGCTGTGCATGCTGCTTTGCTGAGTGAAGACTGTCAAGGGAAGAAATTAATAGAATGA

>Glyma08g02940 CTCTCGCTCCCTCGTGCAATTTCAAAATTACATTACACTCTCTTCGACCTCGCATCGCACCTTCAACTTAACCTATTCCACTTGTAATCGTAAACGCTTTGCTAGAAGAAGAGACCAAGTGTGTCTGTGTGTGTGAGAGAGAGAGCAATGGCTGGCTCGTGGGCACGCCGTTCTCTGATTGTTCTAGCCATCATTTCCTTCGGTAATTTCACCTCCTAATAGATCTGTTCATGCTTTTCTTTTAGCTTTTGCTTAAATGCGCAAGTGGAAAAATGTAACCAGATCTCGCTTTGTTTGAACCTTTTTGGATCTGTTTTGTTTGATTCGTTTCGTGTTACGTAGCGGTAGATCAGTGACTGAGATCTTTAGAAGTAATTGTTTAGGATTTCGTAAATCGTGCTGTGTTGTGTTCTTCATTTCAGTGAAGTTCTTTGCGAACTCGTTAGTTCTAGGATCTTACATGTTTGTGTAATACGCGTTTCTCTGCATCTGACAATTTCGTCAACAGTGATCAGTTCTAGTTGTTATTCCGATTTCCGAGGGAGCGTTTTGTTGGTTTTGTTTTTGAAAATGCTAATTGTTGCAAATTACTTTTCGTAACCAACACACGTGCCTCTGATTTCTGTTAAATCAGTTTTTATAGTATTTCGTAACCAACACACGTAGTACCATTGATTTCTGTTAAATCAGGTTTTTTTTAATATATTTTAAATTTTTTGAATCAATGTTTCGTGCAAATATGTTTCTAACTAACCAGCTGCATTCTCGTTTGTTTTTTGTTTTTTAAGTTGTTGATAAAGATTTTCACTGTTAAGCCGAGTGATTAGTATTAGCCATGGAAATAAGTTTTGTTCCATTGTGCTGCTTAAGATTTTTGCTTTCATTGCATGCTTTTTTCAAAGGATTGGCGTTTTCGTATTTGCGATTTGAATATGACATCGAGAAGTTTTCCTCCGGCTGATAATTGAATACATATAGAAAAAAGTATAAAGTAAAGTGTCTTCATGTTAATTTTGTTAACGTATTCATATCATTATTATCATTTGTCAAGCACAATTATGTGCAAATGCAGAAATTAACTGCGTTTCGATGAGTTTAAGTGTTGTAAAAATTTAACTATGTAACTAACATTGAGAAATTTCTCCATAGGATGTCTATTTGCGATTTCCATTGCTAAGGAGGAAGCCACCAAGTTAGGGACGGTCATCGGCATTGATCTTGGAACAACCTATTCATGTGTCGGTGTTTACAAGAATGGCCATGTTGAAATCATAGCCAACGACCAAGGTAACCGTATCACCCCATCGTGGGTTGCTTTCACCGACAGTGAGAGACTCATTGGGGAGGCTGCCAAGAATCAGGCAGCTGTCAACCCAGAAAGGACCATCTTTGATGTCAAGAGACTTATCGGAAGAAAGTAAGACTTGCTCAAGTTGTTGATTTTTGACAATTTGATTTGGTGGGATCAATTGTTACTGACTTGCATCTTTTTGCAGGTTCGAAGATAAGGAAGTTCAAAAAGACATGAAGCTTGTTCCTTATAAGATTGTCAACAAGGATGGAAAACCTTACATTCAGGTGAAAATTAAGGATGGTGAGACCAAGGTGTTCAGCCCTGAGGAAATCAGTGCCATGGTTCTGATCAAGATGAAGGAAACTGCGGAAGCATTCCTCGGGAAGAAAATTAATGATGCTGTGGTCACTGTCCCAGGTAACTGGAAGTCTGAAACAATTTGCGCCTTATTTCTCCCCTTCAATTTGATTGTTTTCCTCCTGCTTTTGCTTTTATTCCAATTTCTCACATTTTTGTTTCAAATGCACAGCTTACTTCAATGATGCTCAGAGGCAGGCCACCAAGGATGCTGGTGTCATTGCTGGTCTCAATGTTGCTAGAATTATCAACGAACCCACTGCCGCTGCCATTGCCTATGGATTGGACAAGAAAGGTGGCGAGAAGAACATTCTAGTCTTTGATCTTGGTGGTGGGACCTTTGATGTCAGTATCTTGACAATTGATAACGGTGTTTTTGAAGTTCTTGCTACAAATGGAGATACTCATCTTGGAGGTGAGTACTTTTATACACATACACACAAGAGAATGAAATGAAATTATATATTCTTTCCATAAAATTTTCTTTCTGATGCTAATGTCTGCTATTTCTGTTTCCCCTTACTAGGTGAGGATTTTGATCAAAGAATAATGGAGTACTTCATTAAATTGATCAAGAAAAAGCATGGAAAGGATATTAGCAAGGACAACAGAGCACTTGGAAAGCTGAGGAGAGAGGCTGAGCGTGCAAAGAGGGCACTCAGCAGCCAGCACCAGGTCCGCGTGGAAATTGAATCACTTTTTGATGGTGTTGATTTTTCTGAGCCACTCACCCGAGCTAGGTTTGAGGAGTTGAACAATGACTTGTTCCGGAAGACCATGGGTCCAGTGAAGAAGGCTATGGAAGATGCTGGATTACAGAAGAGTCAGATTGATGAGATTGTTCTTGTTGGTGGAAGCACAAGGATTCCAAAGGTACAACAGCTTTTGAAGGACTACTTTGATGGAAAGGAGCCAAACAAGGGGGTCAACCCTGATGAAGCAGTTGCCTATGGTGCTGCAGTGCAAGGAAGCATTTTGAGTGGAGAGGGTGGTGAAGAAACCAAAGGTACCATAGTCTGTAAATATTATTTATGGAAGTAGATTGCTAAGCATTACACTTTCTAATTGTTGTGTGGCAATCTCTATCCAGATATCCTTCTCCTGGATGTGGCTCCCCTCACTCTCGGAATTGAAACTGTTGGTGGAGTCATGACAAAGTTGATTCCCAGAAACACTGTTATCCCGACCAAGAAATCTCAAGTGTTCACCACCTACCAGGACCAGCAGACTACCGTCTCCATTCAGGTACGGTGCCATTAAAGGGCATGATGCGTGTTTTTCTTCTAAATCCAAAATGCACCACTATCTGGTGAACTATGATGGGATTGACTTGAAACGATTCACTGCTTTTTTCTTAATACAGGTTTTCGAAGGTGAGAGGAGTCTCACAAAGGATTGTCGCCTTCTTGGGAAATTTGATCTGTCTGGAATTCCACCAGCTCCAAGGTTTGTTACCTGCGTTGATGATGCAGTGTCCTCTCTCATTATTGCTCCATATATTAATGTTAGTAAATTCATTGGTGGTATTATATTTGGGTAATTGAACAGGGGCACCCCTCAAATTGAAGTGACCTTCGAAGTTGATGCCAACGGCATTCTAAATGTGAAGGCAGAAGACAAGGGAACTGGTAAATCAGAAAAGATCACCATTACAAACGAAAAGGGACGTCTTAGCCAGGAAGAAATAGATAGAATGGTTCGTGAAGCAGAAGAGTTTGCCGAGGAAGACAAGAAGGTGAAGGAGAGGATCGATGCTCGCAACAGTCTTGAAACCTACGTATACAACATGAAGAATCAGGTCAGTGACAAGGACAAGCTCGCGGACAAGTTGGAGTCAGATGAAAAGGAGAAAATTGAGACTGCAGTGAAAGAAGCATTGGAATGGCTGGATGACAACCAGAGTGTGGAGAAAGAAGATTATGAAGAGAAGCTGAAGGAGGTTGAAGCCGTTTGCAACCCAATCATCAGTGCTGTGTATCAGAGATCAGGAGGAGCCCCAGGTGGTGCAGGTGGCGAAGGCGAAGACGAGGATGATTCTCACGATGAGCTCTAGATTAGTCTAAATTTTTATCATATATGGGAAATTAAGAATTCCCCTGCGCTCTGCGCTTGCTTTTTTGGTGCCGATGATAAGATACTTGTGAAATTACTTTTACTTTGTTTTTTTGCTTAGTTTTACGACCAAACTTCTTCATGAGTCTGGTTTCACTTCTATTCTCTTACGCTCTTCTTTTCGCGTGCTTCTCCGTTAAAATGTGCTCTGATGAAATTAATCCGTCTCTGCCCATGCCGACCATAAGTTTTT

>Glyma08g02960 GTTTTATTGGCCAAACAATGCTTTCTCAACCTTAGATTGGTCCACATCAGCACATATGACATGCTCGTACTTGCGAGTGAGCACTATAAATGGCGAAGTCCCCGCCGTGAACTCAATAGAACCTACAATTCATCGTCCATCGAGAAAGGAAGAAAAGAAACAAGGTGACATCATCATGGCTTGCTCGTTTTCTCGCGGGTCTCTGCTTCCTCTCGCCATCATCGTTTCCTTAGGTAATAATCAGATCTCATCACTTTTTCTCTTTCAATTTCAAATGTAGTAGCTACATCGATCTCACTACGATTCGTTTGAACCTTTTTCGTGCTTTGTTTCGATCTCTATTTCCATGGATCTGATAAGAACCTAACCCTATTTGCTTAACATTCTCATCACCGCTCTCGATTTAGTGCCACTGAGTATTATCATCATGCATTTCTCGCTCTCACTCTACACCTTTGAAATGGCTCAACAATGATTAGTCGCTGTTTTTCATCTGCGTCATTATTGTATCCGTTTTTCTCTAATTTCGCGAAAACAACGCATGCTGCATAAACCTTTTTTTCTTCGTTAGAGTACTCGTGTTGTTTGTCTTGTGCACCAGTAAATTAATTACATGCTTATTAATTTAGTTTCTCGAATTATAATATTCCTCATCTTTGGTGAGATTAGATTACATTATGCATGATTTGATACCTAAAACATATTTTTTTTCATTCGTTTATGTCAATCTTTGTCACGCCTCCAAATAATTGATTTCTTCCTTTTAAACTAAGATTTGCCTCCTCTACTAAAGCGAGCAAGAGTAAATGTAATCTAGTTGATTAGAAAATCAATTATTGATATTTCATCACATTCATAATTCGATGTTGTGCATGTATACTTGATGTCAACTATTGTCATGAATAAATGAGATTACTTACTTTACGCGAGTCAGTTTGTCATGAATTTAACTAGTTTGAGTTTACATTGTAATTAACCTTGAGAGAGTTCATCGCAGGATGTCTATTTGCGATTTCCATTGCAAAGGAGGAAGCCACCAAGTTGGGGACGGTCATTGGGATTGATCTTGGAACGACCTATTCATGTGTTGGTGTTTACAAGAACGGCCATGTTGAAATCATAGCCAATGACCAAGGTAACCGTATCACCCCTTCTTGGGTTGCTTTCACCGACAGTGAGAGACTCATTGGGGAGGCTGCCAAGAATCTGGCAGCTGTCAACCCAGAAAGGGTCATCTTTGATGTCAAGAGACTTATTGGAAGAAAGTAAGACATCCTCAAGTTTTTGATTTAGGACAATTTGATTTGATGTGAATAAAGGTGTCTGACATGTATCCTTTTGCAGGTTTGAAGATAAGGAAGTTCAACGAGACATGAAGCTTGTTCCTTATAAGATTGTCAACAAGGATGGAAAACCTTACATACAGGTGAAAATTAAGGATGGTGAGACCAAGGTGTTCAGCCCTGAGGAAATCAGTGCCATGATTCTGACCAAGATGAAGGAAACTGCGGAAGCATTCCTTGGGAAGAAAATTAATGATGCTGTGGTCACTGTCCCAGGTAACTGGAAGTCTGGAACAATTCATGACTTTCTGATTTTGCTTTTATGCCAATATCTCACATTTTCCTTTCAACTGTACAGCTTACTTCAATGATGCTCAGAGGCAGGCCACCAAAGATGCTGGTGTCATTGCTGGTCTCAATGTTGCTAGAATTATTAATGAACCTACTGCTGCTGCCATTGCGTATGGATTGGACAAGAAAGGTGGCGAGAAGAACATTCTTGTTTTTGACCTTGGGGGTGGAACATTTGATGTCAGTATCTTGACAATTGATAATGGTGTTTTTGAGGTTCTTGCTACAAATGGAGATACTCATCTTGGAGGTGAGAATTTTTTTTAATGAGTCTGAATAAGAAAAACTTATTATGTAGAAAAAATATTATTCTTTCCACAAAATTTTCTTCTGATGTTAATGCTTGCTATTTCTCCTTCACCTTACCAGGTGAGGACTTTGATCAGAGAATAATGGAGTACTTCATTAAATTGATCAATAAAAAGCATAAAAAGGATATTAGCAAGGACAGCCGAGCACTTGGCAAGCTGAGGAGAGAGGCTGAACGTGCAAAGAGAGCTCTCAGCAGCCAGCACCAGGTCCGCGTGGAAATTGAATCACTTTTTGATGGTGTTGATTTTTCTGAGCCACTCACCCGAGCTCGGTTTGAGGAGTTGAACAATGACTTGTTCCGGAAGACCATGGGACCAGTGAAGAAGGCTATGGAAGATGCTGGATTACAGAAGAATCAAATTGACGAGATTGTTCTTGTCGGTGGAAGCACAAGGATTCCAAAGGTACAACAGCTTTTGAAGGACTACTTTGATGGAAAGGAGCCAAACAAGGGTGTCAACCCTGATGAAGCAGTTGCCTATGGTGCTGCAGTGCAAGGAAGCATTTTGAGTGGAGAGGGTGGTGAGGAAACCAAAGGTACTGTAGTCTGTAATCTAACTTCTCAATTACTTGTCCAGAAATCATAAATATTATTTATGCAAGTAGATTGTGACACATTACGCTGTCTCATTGTTGTGTGGCTCTCTGGATCCAGATATCCTTCTCTTGGATGTGGCTCCCCTCACCCTTGGAATTGAAACTGTTGGTGGGGTGATGACGAAGTTGATTCCCAGAAACACTGTTATCCCTACCAAGAAATCCCAGGTGTTTACCACCTACCAGGATCAGCAGAGTACCGTCTCCATTCAGGTATGGCTCATTATTCAACTGCATTAAAGGGCACGATGCTCAATTGTCTTCTAAATTCAAAATGCATGACTGTGTAGTGTAGTGGACTTTAATAATTGATGCCTTTTCTCAATACATAGGTTTTTGAAGGGGAGAGGAGTCTCACAAAGGATTGCCGTCTGCTTGGGAAATTTGAACTGTCTGGAATTCCTCCAGCTCCAAGGTTTGTTTGCTGCTTTCATGATACTAGTGTCCTCTACAGATGTTGTTCAATTGATGTTTGTAACTCATTGGTATGTTATGTTGTAATTGAACAGGGGTACCCCTCAAATTGAAGTGACCTTCGAAGTTGATGCAAATGGCATTCTAAATGTGAAGGCAGAAGACAAGGGCACTGGTAAATCAGAAAAGATAACAATTACAAATGAAAAGGGACGTCTTAGCCAGGAGGAAATTGAGCGGATGGTTCGCGAAGCAGAAGAGTTTGCTGAGGAAGACAAAAAAGTGAAGGAGAGGATTGATGCTCGTAACAGTCTTGAAACCTATGTCTACAACATGAAAAACCAGGTCAGTGACAAAGACAAGCTCGCTGACAAGTTGGAGTCTGATGAAAAAGAGAAAATTGAGACTGCAGTGAAAGAAGCATTGGAATGGCTGGATGACAACCAGAGTGTGGAGAAAGAAGAATATGAGGAGAAGCTCAAAGAGGTGGAAGCTGTTTGCAACCCAATCATCAGTGCTGTGTATCAGAGATCAGGTGGAGCCCCAGGTGGTGGTGCATCAGGCGAGGACGACGATGAAGATTCTCATGACGAGCTCTAGAGATGTTGTTGCTTAAGCAGTAGCATTAAATGATGAGCAGTAGTGTAGGTTAAACGGAGTCTTCAAAATTTTGTAACAGGTGGAAATTGACAATAGTTTCCCCAGCTCTTGTATCTTTGGTGCCGAAAATACTTTAAAGATGAATGCTTGAATTTGTTTTAGGATGGCTAGTTTAACCACCATACTTCTTCGGCTTTCCGATATTCGTTTTTATCTTCATGCTAGGGAGGACGGTTGGACTCGGAACTACAGCCGTCTACGTATGTTGGAATTAGTGTCTGTTACGTAGATTGAGCTTAAAATTCAGTCTATCAAACCAAAGTAACTCTTTTAAAGATCTTTTGTCTGCTACTCTTTTAAAATTTATTTTAATTCATT

>Glyma08g06950 AGATACTTTCTTTAGAGAAAAGTATACTATACGAATACGAATACGATACCTTCATTTCCACCTCTCACGACCATCACTTCTTTCGTCAGCACAAAACCCTAAACCCTAATTTAAAGCCAAACCCTCCCATTCAGGCATTTCTCCAACGCCCAATTCCAAAGCCCTTGTTTCACTTTTTCCAATCCGATATGGCCGCCGCGACCGCCTTGCTCCGCTCTCTCCGCCGCCGCGACCTTCCCTCGTCTTCTCTCTCCGCCTTTCGTTCGGTACCGTCATCGCCCTCTCTTTCGCATCTCCATTTTCACTCGATTAACCTCACTTCTTATTTTCTGAGATGTTTGCTGAGTTATGTGACGAATATAGCGAATCGCTTTGTGTCTTGTGTTTAGAAACTGTAACTTTTTATTTAAATGATTGATTTTTAAGATCTGAGACGAGGCTCTATGTCCCGTGATTGTGCGACTTTGCTGCGAAGGTGAAATCTTATGTGAAATAAGAAATAAAATTTTAACCCGTCAATAATATTACTAGATCTTAGAATTTAACAGTTTTAGAAAGGTTCTGAGAACCGCACTTTATATAAGATTGAGAGATACTTGTTAACTTTGAATGTTACTGATTATTGGGTCATGTATTGGCTTGAAGTTTTGCTAGATTATCTGCGTTTTCTGTGCTTCTGACTTCATTGTAACTTGTATACAAGTACGAATGCTGTTTTTGATGTACGTAATCTATTTTTTTTTAGAACTGCTCATCTATTCGGGACCTAGTAAGTATATGAACAATTGTTGATATCATGGCATCATTTGTGGAATAATGATTGTTGAACTTGGTTGATCATGTCAGCATTGTTCTGAAGGTAATCTTAATTTTCTTTCACATTGTTGTGCAGTTGACGAGTGGCACAAAGACATCATATGTAGGGAACAAGTGGGCAAGTTTGTCTCGGCCGTTCAGGTGCCATTTGTTGTTCTGTTTTGATAATTGTTACACTTTATATAGAGAGAGATATGGGTAGTGGGTACTTCCAATCTCAGATTTATTCATTGTGAAATTCCATCCTCACTGCAGTTCAAAGCCTGCTGGTAATGATGTCATTGGAATTGATCTGGGTACTACCAATTCATGTGTTTCTGTTATGGAGGGAAAGGTAAGATTTCCTCTCTCTTAATTTGTTACGAATTTTTCAGTGGTGTCTGTGCTGACTAATATTTATTACATTTTCAGAATCCCAAAGTTATTGAGAATTCTGAAGGAGCTCGAACAACACCATCAGTGGTTGCCTTCAACCAGAAAGCGGAGCTTCTTGTTGGTACACCAGCCAAGCGTCAGGCTGTGACAAACCCAACAAACACTCTTTTTGGAACCAAGCGTTTGATAGGTAGGCGTTTTGATGATTCTCAAACTCAGAAGGAGATGAAAATGGTTCCATACAAGATTGTTAAGGCTCCCAATGGAGATGCTTGGGTTGAAGCCAATGGGCAGCAGTATTCTCCCAGCCAAGTTGGTGCTTTTGTTCTCACCAAGATGAAGGAAACTGCTGAGTCATATCTTGGAAAGTCAGTTTCAAAAGCTGTAATTACTGTACCAGCTTACTTCAATGATGCTCAGAGACAGGCAACAAAAGATGCCGGTAGAATTGCTGGTCTTGATGTTCAAAGAATCATCAATGAGCCCACTGCTGCTGCACTTTCATATGGGATGAACAACAAGGAGGGTCTCATTGCAGTTTTTGATCTTGGAGGTGGAACATTTGATGTGTCCATCTTAGAGATTTCTAATGGTGTTTTTGAGGTATTAACACTTGGTTATTCTATGCCTATATTTCACAACCTGTCATTAGAAATAACGTTGGAAATTGTTACTGATAACTATTTTGTGTCCAGGTGAAAGCCACAAATGGTGACACGTTCTTGGGAGGAGAGGATTTTGATAATGCTTTATTGGATTTTCTAGTGAATGAATTCAAAAGAACTGAGAATATTGATCTTTCAAAGGATAAACTTGCATTGCAGAGGCTTCGAGAAGCTGCTGAGAAAGCAAAAATAGAACTGTCTTCAACATCTCAAACAGAAATCAATCTTCCTTTCATCACTGCTGATGCATCTGGTGCAAAGCATCTGAATATCACATTGACTAGATCTAAGTTTGAGGCTTTGGTAAACCACTTGATTGAAAGGACAAAGGCACCGTGTAAGAGCTGTTTGAAGGATGCTAATGTATCCATCAAGGAAGTTGATGAGGTTCTTCTTGTTGGAGGGATGACCCGGGTGCCTAAAGTCCAGGAGGTGGTTTCAGCGATCTTTGGAAAGAGTCCTAGCAAAGGAGTAAATCCTGATGAGGCAGTTGCCATGGGAGCAGCTATTCAGGGTGGTATCCTACGTGGAGATGTTAAAGAGCTACTACTCCTAGATGTCACTCCACTTTCTCTGGGTATTGAGACTTTGGGTGGTATCTTTACCAGGTTGATTAACCGCAACACCACAATTCCTACTAAAAAGAGTCAGGTAATCTTTAAATATTGACTGATCATCGTTTTGCACTTTCCAGGTTATGGGGTCAGTCTTTCATAAGTGATGTAACATTCAAATCATAAAATTATATGCTTAAATCTTAATTGAAAATGGGTTCAAGGGATAAAAAAAATGTGCATAAATCCCCCCCTCCCCCCTAAGTAGATTAGTAATTTTAGATGTTTAATATATATAATTTTGGTAAATCTATTCTTCTCGATTTGGTAGAGTCTAATTCTAACTTGTGATATCATTCAGGTGTTTTCAACAGCAGCTGACAATCAGACTCAGGTTGGTATCAAGGTGCTACAAGGTGAGAGGGAGATGGCTGTAGACAACAAATCGCTTGGAGAATTTGAGCTTGTTGGCATTCCTCCTGCCCCAAGAGGCATGCCTCAGATTGAAGTCACATTTGATATAGATGCCAATGGGATTGTTACTGTCTCTGCCAAAGACAAGTCCACTGGGAAAGAACAACAAATCACCATCCGTTCATCTGGAGGACTCTCAGAAGATGAGATTGATAAGATGGTCAAAGAAGCAGAGTTGCATGCTCAGAAAGACCAAGAAAGAAAGGCTCTCATTGACATCAGAAACAGTGCAGATACAACCATCTACAGCATCGAGAAGAGTTTAGGTGAGTACAGAGATAAGATCCCCAGTGAAGTGGCCAAAGAGATTGAAGATGCAGTATCAGATTTGAGAACAGCAATGGCAGGAGACAATGCTGATGAAATTAAGGCAAAGCTTGACGCTGCAAACAAAGCTGTCTCCAAGATTGGAGAGCACATGTCAGGTGGTTCTAGTGGCAGTTCCTCAGCTGGAGGTTCTCAGGGTGGTGAACAGGCTCCCGAGGCAGAGTATGAGGAAGTCAAGAAATGACATTTAGGCGTGGTTTTGTAGCCATTTGTTTCTTATTAATCATCACATAATTGCAAGAATACAAGGTTACTTCTTTTGTATCTAATGTTGAAAACCATTTTTTGCTGGAAGGCTCTTTAATTTGTAGCACTTTTGGAGTCCAAAGTTCCGAACACACGGCTGTCTGAATAACGATTTTGAATTTTTTGCATAAAGTTTGTTCACTACGTTTGGCTCTGACTAGTATTTTG

>Glyma08g22100 GTGAGGTAGGAAAACTGCAAAAGCATCCGTGCGGGTGGGAGCGGGACCCACATCCACCAGTGTTTGTTTCTCTCGTATTTTTAAGACCTAAAGACCAAAATTCCACAGCAGCCCCCACCCAATATCTTTTCCGGGGGTACAATACAAATTCCGAACCCCCCATATTCCTCCCTCGCCGATCGCTCACGTCAGGTAAAACCCTATTCCTCCACCCTCTCAGATCTCTCCCTCCCTACGCTTGTTACATTCATCGGAATATCCCAATTCTGATCCAACCGATTCCGTCCTATACTCTTCATTTTCTTCACGTCTTAAATTACTGTTATATTTCTAATTCATTTATTTACTTTATTATTTTTGCGCGCCGTTTTTTGGACGATTTTGTGGTAATTGCGGGTGTTTTCGATTTTTCAGATTTGTTTTTTTTTTTCTTTCTGTTTTGGACGGAATTTTGATCCAGCAAGATGAGCGTGGTGGGATTCGATTTCGGTAACGAGAGTTGCATTGTTGCGGTTGCGAGGCAGAGAGGGATTGACGTTGTGCTCAATGATGAGTCCAAGCGTGAAACGCCCGCCATTGTGTGCTTCGGTGACAAGCAACGCTTCATTGGCACTGCTGGTGCTGCCTCCACTATGATGAACCCTAAGAATTCAATCTCCCAGTTTAAGAGACTCATTGGTAGGAAATTCTCTGATCCCGAATTGCAGCGGGATCTTAAGTCGTTGCCGTTTCTTGTCACCGAGGGGAGTGATGGGTACCCGTTGATTCATGCGCGGTACATGGGTGAGTCCAAGACGTTTACGCCTACCCAAGTGTTTGGAATGATGCTGTCGAATCTTAAGGAAATTGCGGAGAAGAATCTTACTACAGCGGTTGTGGATTGTTGCATTGGAATCCCGGTTTATTTTACTGATCTGCAGAGAAGGGCGGTGCTGGATGCGGCCACGATTGCTGGTCTGCACCCACTTCGGTTGATTCAGGAAATGACTGCCACTGCCTTGGCCTATGGGATTTATAAAACGGACCTTCCAGAAAACGATCAGCTGAATGTTGCGTTTGTTGATGTTGGGCATGCTAGCATGCAAGTGTGCATTGCTGGATTCAAGAAGGGGCAGCTGAAAGTGTTGGCTCATTCATATGATAGGTCTCTCGGCGGTAGGGATTTTGACGAGGTTTTGTTCCATCACTTTGCTGGGAAGTTTAAGGAGGAGTACAAGATTGATGTTTTCCAGAATGCCAGGGCTTGCATCAGGCTCAGGACTGCCTGTGAGAAGATCAAGAAGATGCTTAGTGCGAATCCTGTGGCGCCTCTCAACATTGAGTGCTTGATGGATGAGAAGGATGTCCGGGGCTTCATCAAGCGAGATGAGTTTGAGCAACTAAGTCTTCCAATTTTGGAACGTGTGAAGGGACCTCTGGAGAAGGCACTTGCTGAAGCAGGTCTTACCGTTGAAAATGTACACACAGTTGAGGTGGTTGGTTCAGGTTCTCGGGTACCGGCCATTAACAAAATATTGACAGAGTTTTTCAAAAAGGAGCCTAGGCGGACAATGAATGCTAGTGAGTGTGTTGCTAGGGGCTGTGCGTTGGAATGCGCAATTCTTAGTCCAACGTTCAAAGTACGAGAATTTCAGGTAAAGTTTCATACGTTGGATTGTTCTCTATTTCATTTCAATTCAGAAGGACCCTCATACGCCACTGTTGGTTTTGTAAGCTTGCTTGCTTATTGTCTTCTGTACTTGTTGTAAACTGTACTGACCTTTAGGAATTACTGTATGATTTATGAATCTTAGCATGATTGATGCTTATTTTAATTACATATCTGTGGTATCTACCTGTGTTTAGGCCTTTAGGAAATTCATGCATCAGTTTCTTCTGTGCTTTTTACATATTTGTGAATTGTCATTGCTGTGAAATTATTCATATACTAAGCTTTGCATGCGATATGATGTTGCTATTTTTGTAAAGGTCAACGAAAGCCTTCCTTTCTCGATTTCTCTTTCATGGAAAGGTTCTGGTCCAGATGCACAGGACAATGGATCAGAAAATCAGCAGAGTTCCCTTGTTTTTCCTAAGGGTAATCCCATACCAAGTATCAAGGCACTGACATTCTGCAGGGCAGGAACATTCTCTGTTGATGTATTATATGATGATGCAAGTGGGCTGCAAACACCTGCTAAGATCAGCACATATACTGTGAGCCAGTTTCCAACTTTCTTCTTTTTTTGGTTATATTATTTTTTTGGCTGTGCTGTGACTCATTTGAGTGTAAGCAGATTGGTCCTTTCCAAACTACAAATGGTGAAAGGGCAAAAGTTAAAGTGAAAGTTCGTCTGAATCTGCACGGAATTGTATCCCTTGAGTCTGCAACAGTAAGTTTTTTCTGTCTTTAGGGATCCCCCTTCCCTCACCTACTATAACCACCCTTGTGAACTTATGATAGTATACAGAGCATCTGTTCCTGTGATCTAACAACTTCTATTTGTTTTGTTTAGCTCCTGGAAGAGGAAAAAGTTGGGGTTCCAGTTACCAAAGAGGCAGCAGGGGAAAATACTAAGATGGACATTGATGAAGTCCCTGCTGAGGCTGCTGCACCTCCCGCCTCCAATGACACTGGTGCTAATATGGAAGGTGCAAAGGCTAGTACTGATGCCTCTGGGGTTGAAAATGGCATCCCTGAGGGTGGAGATAAGCCTTTGCAAAAGGATACTGATACCAAGGTAAGTACTGTAGAAAAAATATTCAAGCATATGCTCGAAATGTCTTTTCATTTTTTATGCCATTGTTGATTATTTTAGCAATATTTTTGTTGTTTTATTTTGAACATGTTTTGTTGAGTTCTTATGTGGTGCTTCTGTGTGTTTGCATCATCCTGTTGTTGATTTAATTTGCTTGATTTCCTGCTTCAGATTAAATGTAAGTCTTAGTGCCTGTTGACTTGAGAAAATGTTTTCTCCTTTTGTTTCTTGTTTTTACTTTTGATTAAAAAAATAACACATTGTTTTCATTTTGTTTTGTTTTTAATGATTTGTGTAAATAGTGAAAACATTTTTTTTACCATGCAAATCTTTGAAAACTGGAAACAAAGTAAAGAGGTATGCCTTTTTTGTCGCGATTTGTAAAAAATGAGAGATCAAAATAGATATTTTTCTCTTTATAAAAAGACCTCATGTTTTCCATGAGTGCACTGCCAGTCATGTACAATGATATATAATACATATTATATACTCTTCCTGTTGCTTTTTTTGTGCTTCAGTTAGAGTTGTTCAACATGGTTAAGTGATATTTTGTAATTTGGTTGACTGGACTCTTAATGTTGTAGGTTCAGGCTCCAAAGAAAAAGGTTAAGAAAACAAACATTCCTGTAGCAGAGTTAGTTTATGGAGCAATGGTGCCTGTGGATGTCCAGAAAGCACTAGAAAAGGAGTTTGAAATGGCTTTGCAAGATCGTGTGATGGAAGAAACAAAAGACAAGAAAAATGCAGTTGAGGCTTATGTTTATGACATGAGAAACAAGGTGGGTGTTATCTTTTTCTTCTTCTCTTACTCAGGGTCTAACATCAACTACAAATGCCTGTCATATAATTGTTGATTTTTTTTTTTCCGTTATCATTTCAGCTTAATGACGAATACCAAGAGTTTGTCACTGCTTCAGAGAGAGATGATTTTACTGCTAAACTTCAGGAAGTGGAAGATTGGCTTTATGATGAGGGTGAAGATGAAACTAAAGGTGTATATATTGCCAAGCTTGAAGAACTCAAAAAGGTTAGGAAGTTTTGTTGATAAGGGAATCCTAGCTCCCATTCCTCAGTCAAACTGTAATATTGTGCAAGGAAAAGGGACCAGAGTACCTCTTGCTGTTATCCTTTGTGTTTTTTTGCACAAACATAAATGGTGTGAGGTATTTCCATGCTAGTTTTAACATAGGCAATCTCAATGTGATTGCAGCAAGGTGATCCAATTGATGGGCGATACGAAGAATTCACGGAGAGGGGTACTATAATTGAACAGTTCGTGTATTGTATAAATAGTTACAGACAAGTTGCAATGTCAAATGATCCTAGATTTGAGCACATTGACATTAACGAGAAACAGAAGGTAATTGCATGTCAATTTTCTTTCCCTTGTTTCTTGGAATGGTTGTGAATAAATCTGAAACCATTTTTCTTTCTATGTGTTTGTAGGTCATAAATAAATGTGTTGAAGCTGAGAAGTGGTTTAATGAGAAGCAGCAGCAGCAGAGCTCACTTCCAAAATATGCCAACCCTGTACTCTTGTCAGCTGAAATGAGAAAGAAAGCTGAAGATGTCGATAGGTATTTGTTTAAATTCTTGCTTTGGATATGTAGTTTTTGGGTTATTTAGACATCATTTGGCTTAAGTATATATTTAGTGTCTATATGTCTGATGCATTTGGTTTTAGTCATTTTTACATATTTTTCAATTGGTTTTGATTCTCATAAATTTTAATTTTTTTAAAAATAGTTCTTATTTATGATGTCACATCAGTAATTATTTATAGGTGACATGATGCAGATCGCCAAGTAGTGAAGAACTGTTTTAAAAATTTATAAAATTTGTGAGGACCAAAATCAGTAATTTTTTTAAGGAGTAAAATGAAGTTATATTTACAGAATTAAAAACACATTTATCTTAGACTTTGTTGGTTATCCCTTTACTGTGGCATTGCTTAATGTATTCTTATAAATCAGTCTAGTTGGGGAAGGATTAGCTGTCACTTGTGAACCTTTTAAGAACATCTTGAGTTCTCTTCATTATTCCTTTTTCCTGTTTAAAAAAATACATATTTTTTGAATAGGTTCTGCAAGCCGATTATGACAACACAGAAGCCAACAAAGGCAGTTACTCCAGCAGGACCAGCAACCCCATCTTCTCAGAGTGATGAGCAACAGCAACCCCAGGGGGATTCTGATGTCAACAGTAATGAGAATGCTGGGAATAGCAGTAGTCAGGCTGCACCAGCATCTACCGAACCAATGGAGACTGAAAAGTCTGAGAACACAGGCTCTGCCTAATTTTTTTGTTGTTGGTTTGCTCTCAAGTCTCTACTATTTGTATAAGCAAAAGAGAATTTTGCTGGGTTGACAGTGAGTGTGGCTCCTTGGATATTTGGTGTAAGTAGAACCCTCTCGGTTGGTTGGTAACCTAAGTTTTAATTAATATTATGGTGGTTCTTTGGAATCCTGATTTTTTTCACGAAAGAGTTTAGAAGAAAAAACGACAATGTGGTTGGAATTGGCCAGAGTTACTTTCTCTAGGTTTATATATAGATTATTTGTATAAGATTTTTAGACGATGAATGAATTATAATGTTCATTTCCCGTTTAATACTTGCGTTTATTTTACGTGGCATTATCAACCCTTGC

>Glyma08g42720 GGGTAAGCCACCACTAGGACCAAATTGTACTATTTATATTTCCATCCCACCCTTGTTTCTTCACTCTTGAATTAGAGGGTATAATTACAAAACCACTTTTTACAAATATGACCCCAAATAAACTCACTTTTCTCATATCTGGCGTCGTTGCTCACTTCTGTAAAAACCCCTGCTTCGTGCCATTGACATAAAGCGAAGATTTTTCTCTCTCTCACTCTCTTTGTCTTCCATCTTCGTCACCACCCGCACTATGCAATCCCAAATGCTTCTTGATTCCACCATCCGTAAGGTATATTCCTTTCTTCGTTGCTGTTCAATTCAGATTGGCTCAACCCACTTTTTGTTAGCTTATGTGGCTTTTTTGTTACATTGAAAACATGCCCCTCTTTTCCCCCCTCAATTTTTCTGGGAAATGAAGGAAAATTCAACGGAAAAATATGATTTTAGTAATGGGTTTGGCGTAAATTGTTGGTTCCCCCTCCCCCATTTCACCATTTCCTTTTTGCCCCATTTTTTACTTTTGTTCCTGCTTGCACCTGATGAGCAATCTAAGGAATTTTCAGCAATAGTAGATGGAGTTTAGTTTGTTAGTACTTTCATTTTGATGATAAAGGTTTATCATTTTGATGTGTCTTTTTAAATCCCTTTTGTTTTGTTTTGTAGTGATTTGTGGTGAAGCCTAGGTTTCTGGGCTGGTTTTGTGGAATCTGGTGAAGTTTTGGATTTTGGGGGTGTTGGGTGAGTAGAGTGGGTGAATTGGATTGGACTAGTTGGTTGAAGTTTGGTGCAAGTGAGTTGAGATATGAGTGTGGTGGGGTTTGACATTGGTAATGAGAACTGTGTCATTGCTGTAGTTAGGCAACGTGGCATTGATGTTTTGTTGAATTATGAATCCAAACGTGAAACCCCGGCTGTGGTCTGCTTCAGCGAGAAGCAGCGGATTTTGGGGTCTGCTGGTGCTGCTTCTGCTATGATGCACATCAAGTCCACAATATCTCAAATAAAGAGACTAATTGGAAGGAAGTTTGCAGACCCTGATGTGAAAAAAGAGCTGAAAATGCTCCCTGGTAAGACTTCTGAGGGTCAAGATGGAGGCATTTTGATTCACTTGAAGTACTCGGGGGAGATTCATGTATTTACACCTGTTCAATTTCTGTCCATGCTCTTTGCTCACTTAAAGACTATGACCGAAAACGATTTGGAGATGCCCATTTCAGATTGTGTTATTGGGATCCCATCTTACTTTACCGACTTGCAGAGACGGGCGTATCTTGATGCAGCGAAAATTGCCGGGTTGCAGCCTTTGAGATTGATCCATGATTGTACTGCAACTGCCCTTAGTTATGGAATGTATAAAACAGATTTTGGCAGTGCAGGTCCAGCTTATGTTGCATTTATTGACATTGGTCACTGTGATACTCAGGTCTGTATTGCATCATTTGAGTTTGGGAAAATGGAGATACTTTCACATGCATTTGATAGGAGCTTAGGAGGGAGGGACTTTGATGAGGTTATATTTAGTCATTTTGCAGCAAAATTCAAGGAAGAGTACCACATTGACGTGTATTCTAAAACAAAGGCGTGCTTTAGGCTACGTGCAGCATGTGAGAAATTGAAGAAAGTTTTGAGTGCAAATCTAGAGGCACCTCTAAATATCGAGTGCTTGATGGACGGGAAAGATGTTAAGGGATTTATCACAAGGGAAGAATTTGAGAAGCTGGCATCAGGATTACTGGAGAGAGTTTCTATTCCTTGCCGCAGAGCATTAACTGATGCAAACTTGACAGCAGAGAAGATTTCTTCTGTAGAGCTAGTTGGTTCAGGTTCTAGGATTCCAGCTATAAGTACATCACTAACTTCTCTGTTCAAGAGAGAACCCAGCCGACAGCTGAATGCAAGTGAGTGTGTAGCTCGTGGTTGTGCTCTACAGTGTGCAATGCTCAGTCCTGTTTACCGCGTGAGAGAATACGAGGTTTGCGTATTTTTTTTCTCCTAATATATTCTCATCTTATCTTATGTTATAAATATATATTGCTGATTAGAATTGAGTTTGTAGATCCTTAGAAATGTAGTAAATTAGTAATTCTGAAAGGATCAAGTTGTTACCTAGGTTTATTCTAACAAGTTTTGTTTTCAACCTTTTGATTGTCTTCCATAGTTCCATTTTTTCTCTCTCTTTTTTTTTTTATTTGAAATGTAATTTCCATTACTCATTTGTATAGACTCACGTGCCTCTCTTTGTCATATTGTTCTCTATATAATTAATGTGATTTCTCATGTCTGTTCAGGTCAAGGATGTTATTCCCTTTTCAATTGGACTTTCATCAGATGAAGGTCCAGTTGCTGTGAGATCAAATGGTGTACTTTTCCCAAGAGGCCAACCCTTTCCAAGTGTTAAAGTCATAACCTTTCAGCGAAGTAATTTGTTTCATTTGGAAGCTTTCTATGCTAACCCAGATGAACTACCACCTAGGACATCTCCTAAAATTAGCTGTGTCACGGTATGCCCTCTTTAGAACATTCAGCTAATGTGAAGCAGTTAAAATTATTTTGTGCACATTTTTATCTGTTTTAAATTTTCTTGTCAACCACCCCACCCCCCTTTCTTAGTAGTAGTTATCATCCTCACTCTCCTGGTTTTCCTGTGTGTTTAATGCAAATCATGCACTATAGCTTTTGTGTGAGCACTTTTTTTAGGGGGTAAATCTATGTGGACTTTTTAACATTCTTTATTTTACTTTTTGGGTACTTAATTATTCTTATATATGTTTAAAATTTAAATCCCACAGAGAAGTTTGTTTTTGGTAGAATATCATCATTCCCACAATCTACACCTTTTCTTTTTGAGCTGTAATATTCCAATATTTAGAATCTGTTGATGTAATATCTCCAAACATTTTGTTGACTCTTCTCAGTTATTGGCATCAAGTGAAAAAAATCACTTCAATGGATCCAGAGCTGTTGCAATAGACCCCTCACGCTATTTCCCAACAAGAGATAAACTTCGTTACAAATGGCCTGTGACTGACTATTACTTGTTTTGACATAAAAGGAAACCATGTTGTAAAACTAATTAGGCATAACAATATGAAAGTCAGAATCAGTTTTCTGTGGATATGCACACACTTGACTAAATCATTATGTTAGGAGAAAGAAGTGTATGTGGTAGATCTAACGTCATTCTTTGTGATGGCAATTCAGCATGCATTATCTGCATGCCCACACCTTGAAATATGCTTATGCATTTGATCACTACTTCATTTCAGATTGGTCCTTTCCATGGATCCCATGGTAGTAAGATCAGAGTTAAAGTTAGAGTTCCACTTGATCTGCATGGCATTGTCAGTATTGAATCAGCTACAGTAAGTAAGAAGCCTCATTTTATCTTTCCATACTGCATTATTAGTTTTTCTAAGAGAGATCTTTGCACATAAACCTTTCTATGGTTTCAGTTGATCAAGGATGACATGGATGATTTGGTTATGGCTGGTGATTATCATTCAAATTCTGATGCAATGGACATTGATCCCATTTCTGAGACAGTTACCAATGGGTTTGAAGATGATACCAATAAGAAGTTGGAATTTCCATGTAGTTCTGTAAGTACTCATGTTTTCCCCTTCTCTTTATCTCACTTTCAGTTTTCCTTTCTGGGAAATCTCAGAGGTTGGAGATTTCTGGCAATTTTATCCTTTGAGCATGGAAAATCGTATAGATAAAATGTAAGAAGCAATTGCCATAATCTCTGATGAATTCTGTGACATTTGTCATGATTGATTTTCATAAAGTCCATTTGTTGTTAGGAGTAAATTTATTAGTTTTAGTTACCCTTTTGGTTCCTATAGTTGCAACCACTTTCCATTTTAGTCCCTATAGTTTAAAATATCTCATTTTGTTCCCTATAGTTGCAATTTTTTTATCCCTTTGGTTCTTGCCATCAAAACTGCTACAAAACAGAAACTTTCACTACCAAAAAATTTAGACAGTTTCAGTTTTGTGGTAATTTTAAAGGATCTGGTGTAAAGGGTTAAAAATTGCAACTATAGGGACCAAAAGGGCATTTAAACCATTTTATTATAATTATCATTCCTTTTCAAAGGCTGATTGCGATTCTGGTGATATAATATTTTTGCGGTGATAATTATGATTGCAAATATGATTCATTTTGGTATTGGGAGTTATTAAAATATTTCTATCATACCTTTTTACAGGCTGATGGTACAAGAAAAGATAATAGAAGGCTTAATGTGCCAGTGAATGAGAATGTCTATGGTGGAATGACAAAGGCAGAGATCTCAGAAGCTCTTGAAAAAGAACTCCAGTTAGCCCAACAGGACAGAATTGTAGAGCAAACCAAAGAAAAGAAGAATAGCTTGGAGTCTTTTGTTTATGATATGAGGAGTAAGGTATAGATCCAATGTCTCAAAAATTGTAGACACGACTGGGTATTTTGATGAAGGGTTATTTGGATTTTATGCGGTCACACACTAAGCATAAGCTATAAATAGTTTTGTCCATTGGCTGATCCTTTTTTTCAAAAAAGAAAACAGACATGTTCCTTTTTAAAGAATTGGATTTAACTTTTGTTAAGTTAATTAAAAGTCTTGAGTGTGTCACATGAGAAATTTTATGGTGTAGTTATTGAAATTTGAAAATTGATTGTCATGACTTTCTGTGGTGATTCATTTGTAAATGCAAGGCCGAGATTGGTTTCTTTGTAATCTTATCTGTGAAGGGTTGGAAATTTTAAATCTTTAAATTTGTATCTCTTTCAATGCTACTGACTCTATACTGGCATTTGAAGATTATGTTTTTGGTTCTATTTTGCTTTGTGAGCTGAAATTTAACTTCATTTATTTGAATAGCTCTTCCACACATATCGGAGCTTTGCAAGTGAACAAGAGAAGGATGGCATATCTAGAAGCCTTCAAGAGACTGAGGAATGGCTTTATGAGGATGGTGTTGATGAAACTGAACATGCTTATTCTTCAAAACTGGAAGATCTGAAAAAGGTTTCCCTCCCCCCCTTTTATTACTGAATTCCATGCTGTACTTCTTGTTTATAATCTATGTACTCTTGTAGCTGGTAGATCCAATTGAGAATCGGTACAAAGATGATAAAGAAAGAGTGCATGCTACACGAGATTTATCAAAGTGCATTTTAAAGCATCGTGCTTCTGCAGATTCCCTTCCACCCCAGGATAAAGAACTGGTACAAAAGATTTATGCTAGTATATGCTTTTGTGATTATGATGCTGCCCTTAAGATCAGCTTTCATGTGATGAGTTTATTGTTTCAGATCATCAATGAGTGCAATAAAGTGGAGCAGTGGTTGAAAGAGAAGATCCAGCAACAAGAATCATTTCCAAAGAATACTGACCCAATATTATGGTCAAGTGATATCAAGAGCAAGACAGAGGAGTTAAACTTGTATGATTTCTGTTTTCTTTTTCAGAAGTGTATATTTATTGTTTTCTATGAATCCATGCGACACAAGTCTAGAGAACAACTATTCATAAAATCTTAGTTAGGCAATCAAATCTATTGATTCATGTGTAATAAAGTTCCACATATGATATCAACAGATTTCATAGGGAATAATATTTCTCGTGATCATTTTGAATTAGGACAATACTTAGATACAATTTTTTAGGTACTTTTGATGTACTTCAATCAGAATTAGAGTTTCATCTTGTTAACCTAAGTTAACATTTAATGCAAACATTTATTACACAGATTACCGAGGTGAAACTCTAATTTTGATTGGAAAATAGCACCAAAAACTCCTAAGGAACCACATCTATGTTTTGTCCTTTGAATTATACACTTGCTTGTTCAAAACATTGTTCATATGGTCTTGTAAACATGCTTGTCTTTTCCCTTTGATTTTATCTCAACATTAACAAATGTTCTATTTTGGTGGTTTTCAGAAAATGCCAACAGATATTGGGATCTAACGCTTCTCCATCTCCAGAAGACAAAGACAAGCCGGATACTTTCAATGATCCATGAATACATTGATGTGATTATACCACATGGAACTGAAGACCCAAGTTTTCATCACTGATACCATCTGACAAATATAGAAAAAAAAAGTTTCCTTTCATTTTTGCTTTGCAGCGATTTGTATTTTCATAATCTATCTAATATTTTCTGATATGATATAGTTTCTTCAAATGAATCGAATAGATTCTGCTTTAGGGAAGAAAAAAAATGTGTGAAGTATTAGTGGCACTTGTTTCTGCTTTGTGCTCAAATAAATTCTTACAAAATTGATTGGTCAATCTCGAATAATGTCGGAGTCTGTTGTGAAGCTTTTAGCATATAAGAACGTGTTACGCGGCTTTATGATAATGAATGAAATTAAATGACTGTTATTATTATTGTTAGGCTGGCCACAATTTACTTTGCATAACTCTACTTTTTTTTTAGTCTATCAATTTTATATGTTGTTCTTCTATTCAAAT

>Glyma11g14950 ATTCAAACACGTTCTTTAAAGCACACCCCTTCCCACACATTTCTCCTCAATTCAGTTGAAGGAATTGTTGTAACTTTTTGATTCCTAAATTCTCTAAAATTCGAAATTCTAATTTCGTTAGGGGAAAGAGATTGTTTGTGTGTGAGTGAGTATGGCTGGAAAAGGCGAGGGTCCTGCTATCGGAATCGATTTGGGAACGACGTACTCTTGCGTCGGCGTGTGGCAACACGATCGTGTTGAAATCATAGCCAACGATCAGGGTAACAGAACTACCCCATCCTACGTGGCTTTCACCGACACAGAACGGTTGATCGGCGATGCGGCGAAGAACCAGGTCGCTATGAACCCAACCAACACCGTTTTTGGTAAGTACTACTTTCAACTCATACTGTAAATCCTTGATTCTCTGCAATTTTTCATTGTTCCAGTGAATTTATAACTCTACACGACGTGATTTATATCAGCACTTGTAGAAAATTCCAATTATCTGTAATCTAACCCTAGCTAGTAATATTCTGTAACGATTCTTTCGTGTTTATTTACAGGAAAAAAAAAATCAAAACAGAGTCGTGAATGCTTTTGTGTGGATTGTAGACGTTTGTTAATTAGGCTGTGATTTTCTTTTCAATTCTTTGTAACTCGAGTTCTATCATTAGTTCAATTGTCTATGGATTTTATTAAATGGCTACTTAATTGAATTTTCTGTGCAAAAGAATCCTTGGTTATGCGGTTAGATTGCGAATCCTTTTTGGTGCATGCAGTTGCTAAACATTTGATTGGTAGGCTGTGTATACTTGATTGAATTGTTTTATGTTTTGGATGATAAATTTATTGAATGGATACTTGCTCAAATAATCTGTGCGAAAGCTTCCTTGATTATGCGATTATATAGTGAATCGTTTGGTGATTGTATTTGTTTGATTTGGCGCATGCAGATGCTAAGCGTTTGATTGGAAGGCGTTTTTCCGATGCATCAGTACAAGGTGACATGAAATTGTGGCCGTTCAAGGTGATTCCTGGCCCTGCTGAGAAACCTATGATTGTGGTGAACTACAAGGGGGAGGAGAAACAGTTTTCCGCGGAAGAGATATCCTCCATGGTTCTTATGAAGATGAAGGAGATTGCGGAGGCGTATCTAGGTTCCACCATAAAGAATGCGGTTGTCACTGTGCCTGCTTACTTCAACGACTCACAACGTCAAGCCACCAAGGACGCTGGTGTCATTTCTGGGCTCAACGTGATGCGAATTATCAACGAGCCTACCGCGGCTGCCATTGCTTATGGGCTCGACAAAAAGGCCACTAGCTCTGGGGAGAAGAATGTTCTCATTTTTGATCTCGGTGGTGGGACTTTTGATGTCTCTCTTCTCACCATCGAGGAGGGTATTTTCGAGGTGAAGGCCACTGCTGGTGATACTCACTTGGGAGGTGAAGATTTTGATAACAGAATGGTGAACCATTTTGTTCAGGAATTCAAGAGGAAGAACAAGAAGGATATTAGTGGAAATGCCAGAGCTCTGAGGAGGTTGAGAACAGCATGTGAGCGGGCAAAGAGGACTCTCTCTTCCACTGCTCAAACCACCATAGAGATTGATTCCTTGTACGAGGGTATTGACTTCTACACAACCATTACCCGTGCCCGTTTTGAAGAGCTAAACATGGATTTATTCAGGAAGTGCATGGAGCCCGTGGAGAAGTGTTTGCGGGATGCCAAGATGGATAAGAGTACCGTCCATGATGTTGTTCTTGTTGGTGGTTCTACTAGGATTCCCAAGGTTCAACAGTTGTTGCAGGACTTCTTCAACGGAAAGGAACTTTGCAAGAGTATTAACCCAGATGAAGCTGTTGCTTATGGTGCTGCAGTGCAGGCTGCGATTCTCAGTGGCGAGGGTAATGAGAAAGTGCAGGATCTTCTTTTGTTGGATGTTACTCCTCTATCCACTGGTTTGGAGACTGCAGGAGGAGTCATGACTGTGTTGATTCCCAGAAACACAACCATTCCCACCAAGAAGGAGCAGGTGTTCTCAACCTACTCTGACAACCAGCCCGGTGTGTTGATTCAGGTCTATGAAGGTGAACGAACGAGGACTCGTGACAACAATTTGCTTGGCAAATTTGAGTTATCTGGAATTCCTCCTGCTCCCAGAGGTGTTCCTCAGATCACTGTTTGCTTCGACATTGATGCCAACGGTATATTGAACGTGTCTGCGGAGGACAAAACCACTGGACAGAAGAACAAGATTACAATTACCAACGACAAGGGCAGGCTTTCTAAGGAGGAGATTGAGAAGATGGTGCAGGAAGCTGAGAAATACAAGTCTGAGGACGAGGAGCATAAGAAGAAAGTGGAGGCCAAAAATGCATTGGAAAATTATGCCTATAACATGAGGAACACAATCAAGGATGACAAGATTGCTTCCAAACTGTCTTCTGATGATAAGAAGAAAATTGAAGATGCGATTGAGCAGGCTATCCAATGGCTAGATGGAAACCAACTTGCTGAGGCTGACGAATTTGAGGATAAGATGAAGGAGTTGGAGAGCATTTGTAATCCCATCATAGCAAAGATGTACCAGGGTGCTGGTGGTGATGCGGGTGGAGCCATGGATGAGGATGGTCCTGCAGCTGGCAGTGGAAGCGGTGCTGGACCCAAAATTGAGGAAGTCGATTAAGCAGAAGATTCTCATGGGTTGTTCTTGATTTTGTCAAAGACCCTTTTGAAGTTTAGATAACAGCGTGTCATTATGTTTTTATTGCGATTTTATTTTTCTCTTTTTTTTTTAATGTACTTTTCCCCGTTTCTACTTGAACAATGATATAAGTTTTTGCTCATATGGTCTATTGATTGTTTCGTTTCGGAGACTAAGGTGGTAAAAGTAAAACATCTTTTTCGCAAGTGATTGGAAAATAAATAGAGTGTAGGATATGTATCCTTTGAAATTAAGGGATAATTGGAGATTATTGAAAATTAGCGTGGCCAACAGAAATAAATTAATT

>Glyma11g31670 GAATCAATCTGGGAACAACTTACTCATGTGTTGCAGTATGGCGGGAGCATCACCGTCGAGTGGAGATAATCCACAATGATCAGGGCAACACTAGGAGTGAAGCAACTAATGATCAGAATTCTTTTAAGTTTGCAGATTCTAAGAGATTGATTGGTAGGAAATATAGTTGTTGCCGGGTGAGGAGAAGCACTTTTGTGCTGAGGAAAAAAATGTCTATCATCAATGGTTCTTGCGAAGATAATGAAGTGGTTACTGTGCCTGCCTATTTCAATGATTCTCAGTATAAAGCTACCATAGATGCTGGAAAAATTGCAGGCCTAAATATTCTGCGGATAATCAATGAACCCGTTGCTGCGGCAATCATGCATGGTCTTGACATGAGGACTAATAATTGTGTTGGAGAGCGAAACATTTTCATCTTTGACCTTGGTGGTGGTACTTTTGATGCGTCTCTCCTTACTCTTAAGGGTAAGATCTTTAAAGTTAAGGCCACAGCTGGAAATGGTCACCTTGGGGGAGAGGACATTGATAATAGAATGCTGGACCATTTTGTAAAGGAGATCAAAAGAAAAAAAAAAGTGGACATTAGTGGGAACCTGAAGGTGCTGAGGAGGTTAAAAACCACGTGCGAGAGGGCAAAAAGAACACTCTCACATGCTGTTACTACCAACATTGAAGTAGATGCTTTATCTGATGCCATTGACTTCTGTTCTTCGATCACTCGTGCAAAGTTTGAGGAAATCAATATGGAGCTCTTTAAGGAGTGTATGGAGACAGTGGATAAGTGTCTTACTGATTCTAAGATGAACAAGAGCAGTGTACATGATGTTATCCTTGTTGTGGTTCTTCAAGGATTCCCAAAGTGCAAGAGCTATTGCAGGACTTTTCCAACGGAAAGGATCTGTGAAAGAGCATCAACCCTGATGAAGCTGTTGCTTATGGTGCAGCTAATGATTCAAGAAGCAGAGGAATACCAAGCTGAAGATAAGAAATTCCTTAGGAAGGCCACTGCAATGAATAAATTGAATGATTATGTTAACAAGATGAACAATGGGTTGGAAAATGAAAATTTAAGTTCAAAACTCTGCTCAGAAGACAAGGAGAAGATAAGTTCTGCAATTACAAAGGCCACAAAGTTGATTGATGGTGATAATAAAAAATGA

>Glyma11g31673 ATGGCTAAAGAAGGTCATAGAATTGCCATAGGAATCAATCTGGGAACAACTTACTCATGTGTTGCAGTATGGCGGGAGCATCACCGTCGAGTGGAGATAATCCACAATGATCAGGGCAACACTAGGAGTGAAGCAACTGTTGGTTGCTTCAGCTTTTTCACGGTGTCACCATGAATTTATGGACTAGAATCAATTCGTACTTGTTATCCGAACACATTATTAATCAGAATGATCAGAATTCTTTTAAGTTTGCAGATTCTAAGAGATTGATTGGTAGGAAATATAGTGATCCTGTGGTTCAAAAGGACAAGCTGTTATGGCATTCAAGGTTGTTGCCGGTACTAATGACAAACCTATGATCATCCTTAACTACAAGGGTGAGGAGAAGCACTTTTGTGCTGAGGAAAAAAATGTCTATCATCAATGGTTCTTGCGAAGATGTGGCAGATTGCAGAAGCGTTTTTGGAGAAACATGCAAAGAATGAAGTGGTTACTGTGCCTGCCTATTTCAATGATTCTCAGTATAAAGCTACCATAGATGCTGGAAAAATTGCAGGCCTAAATATTCTGCGGATAATCAATGAACCCGTTGCTGCGGCAATCATGCATGGTCTTGACATGAGGACTAATAATTGTGTTGGAGAGCGAAACATTTTCATCTTTGACCTTGGTGGTGGTACTTTTGATGCGTCTCTCCTTACTCTTAAGGGTAAGATCTTTAAAGTTAAGGCCACAGCTGGAAATGGTCACCTTGGGGGAGAGGACATTGATAATAGAATGCTGGACCATTTTGTAAAGGAGATCAAAAGAAAAAAAAAAGTGGACATTAGTGGGAACCTGAAGGTGCTGAGGAGGTTAAAAACCACGTGCGAGAGGGCAAAAAGAACACTCTCACATGCTGTTACTACCAACATTGAAGTAGATGCTTTATCTGATGCCATTGACTTCTGTTCTTCGATCACTCGTGCAAAGTTTGAGGAAATCAATATGGAGCTCTTTAAGGAGTGTATGGAGACAGTGGATAAGTGTCTTACTGATTCTAAGATGAACAAGAGCAGTGTACATGATGTTATCCTTGTTGTGGTTCTTCAAGGATTCCCAAAGTGCAAGAGCTATTGCAGGACTTTTCCAACGGAAAGGATCTGTGAAAGAGCATCAACCCTGATGAAGCTGTTGCTTATGGTGCAGCTGTGCATGCTGCTTTGCTGAGTGAAGACTGTCAAGGGAAGAAATTAATAGAATGA

>Glyma11g31810 ATCCCTACCACAACACAAGCCAAAACCCAAACCTTCACCTTCGCTCGCCACTCCACACGCCATTGCAAGCCCCGTGCTTTCTCGCTCCTTCACGTTCCACACTCAAAACTCGAAACCCCACTTACCCAACTCTCTTAAAGTTCACAAATTTCCCAAAATTTTCGACCCCCTTTTGCTTCCAAAATCCACTACCAGTCCTCCTATCACCACTGATTTCAACCGTTGTTCCATAGCTTGGAAGGCAGAAGAAGATGGAGAAGCTTAACTTGGCACTCGTTTCTTCCCCAAAGCCTTTGATGTTGGGACATGTTCCTGCAAGAGACGTTTTCAGAAGAAAACACTTCTCTTTTGGGAGGGTCTTAATTGCTCCTCACCGCTGCAGATTCCGTGTTTCTGCACTCTCTTCCTCCCATCATAACCCCAAATCTGGTATTCAGTTTTTGAACTTCTTTTTATTGTTCTGTTTCTATGTATGTGTGTTTTTTGGTGGGGGGATTAGAGTTTTTTGTTATAATTTATGCTTGGTAGGAAAAAAGATTCATTGAATCACTGGATTCTGATGGAGTCAATCAAATGAAATGTGTGTTTTTGAAGTTGAAGGTAAATTGATAGGTCAAATGATGCTGTTGCTGTTTTTGTTTTGTTTTTATTTTTATTTTTTTGTTCCTTCTTCAGTGCTCCAAAACAAACAAACAAAAAAAGAAACTGAGTTGAACATGTTGGTTCTTTAATTCTTTTGTTATTGGACTAGAGCCATGGATACACTTGGTTGACAATTTCCAAGGGTTTAAGGGAGTGTTTGGATAAGCGGTTGGTGAAAAAACGCACTTTTTATTACATAACCCTTTTTGTAAGCTACTTTTTCTGACTTTTTTTAGAAAAGTTTTAAACTTTTTTTTTTATCCTTTGTGATTTTGGCTTCTAAACGTGTTAAGAAGTGTTTCTATGGTGAAACACTTTTTTCTTTAGAAAGTACTAACAAACGGGCCCTAAGTTTTGATAAATCACCAAAGCATTTTTGCAATTAGTGTATAGTGTTTGTGTAAAAGGAGTGGATTGTGAATTTTGGAGCAAGGTTATGCTCAAGGTCGATTGTGGTTGATAGCATTTTGAATCCATGGTTACAAATTTATTCCATATCTGTGTATTTGAAACCATTTCGGCAATGCATATTTATCATGTGTATTGACTTCTCTTCTTTTGTGGTTAATGAGTGCTTGTTGTACAAGTTGAATATCTGTCAATATTTTTCACTCATCTCTTAAGACGCACAAGTAATGCATGCTGGGAATACAGTGCAGGAGAAGCTGATAGTAAAGCATTTTGCTAGTATTTCTTCTTCAAATACTCAAGAAACAACATCAATTGGAGTTAAGCCACAATTATCACCATCTCCATCTTCAACTATGTAATAATTTACTCTTTTTTATTTAAATTTGTTGGTATCCTTTGCTCATTAGTTCACATTATTATTGGTCTATTTACTATATTTCCAGAGGGTCACCTCTCTTCTGGATTGGTGTTGGTGTTGGGCTTTCTGCACTGTTTTCAGTGGTGAGAACTGAGAAACATATCACAGCTCTGCTTTTCATACACTGATAATGAGTTTGTGTTTGATGTATGTTTAATAAATTGTGTTTTTTTGCAGGTAGCTTCAAGACTGAAGGTGAGTGCCACTTTGTCGCAAGGCGTTGATATAAGCTGTTTCATTCTTTGAGTTCCACTGTAATTATGGGTGTAAGAAATTTGCTAGTCATGGGTGTTAGGCAATAAGCTGGTGCATTCTTTGAGTTCTACTGTAAATATGGGGTAATAAGATGTAGGAAATTTTGCAAGTCATGGATATAAGCTTGTGCATTCTGAGTATCTTAAGTACCAAACATCACTCCAGCAATTGTTTTATAAGAGTCTGTGCATTCTTTTCTAGAATAGTTTTTAATCTTTATGATGGATTTCTTATGAAATTATTTGGAAGTTGAAGGCATGTATCCACAAACATATTGTTGTCCACGCTTCTACAAATTGAAATACACAAATTATGCAGATTGGCTGTATCTATTTTTAGAAACTTAAGTAACAGCCCAACTCACACCTCTTTTATCTGTTAATTTTTGTTTGGATAGTATGTTGTTTGAATGAGACTACTCGAATCATATAGCATTCTGAGAGTTTTATTGAATTTGAGATTTTGATTTATCTTTGAGAATTTGGGTATTGTTTATGATGGCATCTTCTATTGTTTGTGAAAGGACTGGGAGCATGGATCAAGAGGCTGAGATTAAGCCTACAAAGAAAGTCTTACAAATTTATTCATGTATGCCTAGTAACAGCCTGAACTCGTAAAACTTTACTTTTGGGCTTAATCTTAGCTTCTTGATCCATGCTTCCAGTGCTATCACTTTGTCAGTTGTATTAGATGCTCTGTCTAACCTTATTTCTCATGGATTTTGACTGGGTTTGCTGTAGAATTACAAGAACTAACCCTGAACATTTACATATTTTAAACATTTGTCTTTTGATTTCTTTTAGAAATATGCAATGCAACAAGCTTTCAAGACCATGATGGGTCAGATGAATTCTCAAAATAACCAATTTGGCAATGCTGCCTTTTCTCCGGGATCACCTTTTCCCTTTCCAATGCCTACAGCAGCAGGGCCCACTGCACCTGCTAGTTCTGCAACTACTCAATCTCGAGCACCTTCAGCATCTAGTGCATCTCAATCCACTATCACAGTAGATTTACCTGCAGCAAAAGTAGAAGCTGCTCCAACTACTAATGTTAAAGATGAAGTCGAACTAAAGAATGAACCCAAAAAAATTGGTAACTTAAACTTAGAATCTCTGCTTTGCAATCCTATTTCTTTCTTGTTAGATGAGACTTACTACTTGTGAAATTTATTAATGATGACGTAATGTGTTCTGTTCGTTAGGAGATGTATGTGGTTTCAGGTTAATTAAATCAATATTATTAATTAAATAATTAATAACATTTATTAATTCATTTCCTTTATTAATTAATTTTTAAAAAATTTCAATCAAGGTAATTTTTTTTAAAAAATTGCAACAAAATTGAAGATGGAAAACTTTCAGGGAGTTATATTTATAAGTAGCAGAGATGATATCTGATGTCAGCTTTTATATCAGTAAAAATTGAGTCTTTTCTTGTTAAAATTCTTAAGATTTTCTATTACAGCTTTTGTAGATGTTTCTCCAGAAGAAACTGTGCGGGAGAGTCCTTTTGAAAGTTTTAAAGATGATGAATCAAGTTCCGTCAAGGAAGCTTGGGTTCCAGATGAAGTGAGATTTTAAGATGTTTTTTGATAAAGAAGTTCAGTGACTTGTGTTGTTCTGAATATGTAATATGCATTTATTGAAGGTTTCTCAAAATGGAGCTCCCTCTAACCTAGGTTTTGGTGATTTTCCTGGTTCTCAATCTACAAGTAAGTACTAAGTAGAGTCTGTTGAAATTGAAAATATTTACAATTCATCTTTATTGGTGGAAAACTCCATGCAATGTGATACATCTATTCAAGTCTCAACTTCTAGGAAATTATAACAGTGTTTTACTGTGTAATGTTGTGTTTCTTGGATGACCATACTAAGCCTTTATTTATAATGAGCAAATAGGATCAGTATTGATTACATAGTATCTATCTCATAATGAGTAATAACTAATAATGACTAAATCAATAATTGCTATCTAATCATAAATAAGAGAATAATGTATAAATCTAAAAGAAATGCATTGACATGACATGGTTTTAGATCAGTCCTGCCAAATAAATGAGTTGTACTTGTTGTCATTATGCTAGTAAACATTGATTTAATCTTGTTATCATTGCTGAGTTTATCATGGACCTTTTGCACTTCTCATGTCATTGTAATTTTACTTTTTAACTATGAAGTTTTTCTTGCCAACTATTGTTTACTTTTTTTACTGATTAAATACTTCAGTACTTGGATTGAAATGATGTTTTAAATCAAATGACTAGTTCCATACAGCATTGTTTTCTAATCGTATTCATACTACTGGCTTAAATCAATTTAAATGTTGTTTATTGTCATGAAATATATATAGGTTTTACAATTATTGTTCTTTCAATGTTTCCTTCAATGAGCTGCATTATGGGTTAATCAAATATCCTTAACATGCCATTATTTATTTTTGGATAGAAAAATCAGCCTTGTCAGTGGATGCTTTGGAGAAAATGATGGAGGACCCAACAGTGCAGAAGATGGTTTATCCGTAATATTAGTCCCCTTCATCTTAATAATGATTATGATTTTGCATTTCTATTTTCTAGTAAATATTCTATTGTGGCTTTGTCAGTACTCAGTATGAATATGGATATGATTAAACTTTAAGTAGATCCTAGTTTTAAATATATTGAAGAAGGTTCCTTAAGTGGTAGAAGTCTAATCTTAGAGCTGCTGAGGACAGTAGTACTAGTACCTGTGATACTCAGAATTGGGGTTTAAAGTAGTTCCATTCTTTTGTATATTTCTATTGAAATATTTAGTTTGAAATTTGGAATTTGGATGTGTCTAAAATTCTTCTGAAGCTCATAATTATTTTGTCCCTGTGTCCTATTGGCACTTTTCTTCAGATGGTTTTAGGGGAAACCTCTACTTCTGTATGCTTTCATTTCTAATAAAATTTCTATATCCCCAAAAGAAAGTTTAATTGGTTGGCTGGTACCCTTTTAATTGTTGATCGTTTAGTGGAGCTAACTTATGTTCTATACAATAAACTGATTCCCAATGTGTATTTGTTTATGATTACCTGATGAAAATTTACTAATAGGTTTTACTTGCTTGTTTAGATTATTTAAGAACTTTTCCACCTTGTTATCCAAAAGAAGTTAATTTTTGGATTTTATTTGCAGCTATTTACCTGAGGAGATGAGGAACCCTACTACCTTCAAATGTAATTCTCTACTTTTAACTGTTTTCTGCTAATCATAGTTTTAAGATTCATTATTTGTGCAGTGTGTAATTTTCATTGCTAACACCTTCCTGGAAATAAATCATGGGTAACCACAATGCTGAATTTGTTTGTTATCACTTTTGTTTACATGTTCGATATAGTCTATATACAACACTTCTATATAAATATCCTTTTTCATTTGTTACCAGCATCATATAATGGAGGCACTTCTTTATGTAGTTTAAAAATATTTTGTAGTTCAAAAGTCATGTTTTCTTTGAATTGGTAGATATTAATTTCTACATGACATCTTAATATAATTGGTCAATGGGTAACAGTTGCTTAACTGTTGCTGCTTTTAATAGGGATGCTGCAGAATCCACAGTACCGTCAACAACTTGAAGAAATGCTGTAAGTTCTTCATACTGTTTCTTCAGCGGCTCAGAGCCTTTTTGTTTCACCAATATTCTACATTGAGCATTCATTTGGACAGATAAGCGTCTGGGAAAATGCTTACCTGCATTAAGCTTGTTTTACATATTACAACAAAAACTAGTTTCCGGGTGAATGAAAAAGTTCTCCATAATATACTATTCAGTTATTAGCCCTCTCATTTTGTGAATTTGATGAAGTGACAATTAAATCTGATCCTCTTTCTTTATCTTTTATATGTTAGACCTCCAATTTGGGATTAGGAAAAAATGGTGCGGTGGATGAAATGTCTGTTTTACTTCTACTTTTGTATTCCAGGCTCCCAGCCCTCCCTCTAACCTATGAGGGCGGAAATGCCTGTTCTTACCTCCTGACTTTGTTGTTTTAAATTTCTTCTCTTTGTCCTTCTCCCCTTGTTCTTATTTAGATTGATTTTTTTTTTGTTGTTGTTGTTGTTGCTAGGATTTATATGACTTCCTCACCTAGTGTGATAAGGCTTTGTTGTTGTAGGATTTATGTGACTTATAACAACCACATTTCATATAATTGCATTGACTTGAGATCTTAAGTAGTTGAAAAATGTATTTCTTATTTGTTATTTCCCTCTCAACCTTCATCCTATGTACTACAGTTAAATAAATATATGCATATTTTGTTAAGATATAAAATCTTCTCTGGTGTGTAAAGATTCATATATGCTGAATCCTTCCTCTTTTTTCAACATGTTTGATTTCAGAAACAACATGGGTGGAAGCACTGAATGGGACAATCGAATGATGGATACCTTGAAGAATTTTGACCTTAATAGTCCTGAAGTGAAGCAGCAATTTGGTGAGTTATTTTAATATTATTGTTATGCCTCAAGAAGTGCCATGTAGTTGATCAATTTAGCATAGTTCAATTTTTGCTAGCTACTCTTATTACTTGTTGAGTTGAAAATATTCTTAAATACAATTAACTCTTTTCTCGCTTCCATCCTATGGAAATTATATGTTCTACTTAGTTGATCATCTTGATTCTTGAAGATCAATACTAATTGATCAGTGATTTTTTTTTTCTCCTGAAGTTTCATTACATATGGGCAATAACTGTTTCTTTATTCATAGCTTGTTTTCCTAAATCAATTCCATCTGTCTCTTTACTGCTTAATATTCTAATGTTTTATTAATCAAATCATTGCTGTTAATTCTTCTTTATGGGTGATATACTGCTATTTGTTAAAAAATCAAAATAAGAATTTTCAGTGAACTAATAAGTGCCTTGGTAAGTCCCATAGAGAGAAAGCTTAAAGGTTCTCATTCAGAAAGACATCACATTTCATGCCAACTTATTAAAGGATAGTATTTGGTGAGATTCAGCATTCTTTTTGACTCATTGACATGGTTTGCTAGACTTTGCTGACTAGTTTATCTTTTTTATGCTAGTAATACACTTCATCAGTTTCTTTATGTCTCTAGATTTAGTTTATTGAATTTTATCTAATGATAATATCATGTATATTTTTAAGAGCCTAAGTATGAATCTTTCTTTCCACTTTGGGTCTTTTCTGAATATATTTTTACTTTCGCCAGATCAAATTGGGCTTTCTCCCGAAGAAGTCATTTCAAAGATTATGGCCAATCCTGAAGTTGCAATGGCATTTCAAAATCCTAGAGTTCAAGCAGCTATCATGGATGTATGTTCCAATTGAAATACTTCATTGGTCTTTCATATGAAAACTCTGGTATGATTTCATCTCTTGTGGTTGCAGTGTTCGCAGAATCCAATGAATATTACTAAATACCAAAACGATAAGGAGGTATGCTGATACTTCTGACTTTAATTTTGTCCTTCCGGGAGGAGTGTACTTTACCGAGTTTGGTTTTCTCTCCCTTTGTTTCCTGTTTGCTCTTTGGCATGCATAGGTTCTCTAGTTGTAATTAGTTTCAGGTTTATGTTAGGTGAAAATCTTTTGATGATTTTTGACGAGATGACAGGTGGTGGCAGTTTGAATTTGCATATTTCGCTACGTCTCTAACGAACAAAGCTATAATAACTACTCTCTTTTTCCTCTTAAACATTTCAGGTAATGGATGTCTTCAACAAAATATCAGAACTCTTCCCTGGAGTAGGTTCACCTTGATGCATTTGTGTGTTGTTGCTGTTTTCTGGGTCAAGTTATGCATCCTTTTCCTGATGACCTAAGACATTATTAGCTCCGGCCTAACCACACGGAACAATGCAAAACTCTTGTGTGCTCATATACTGCAGGCTCGCAGTTACAGCTCCGGATAGTAACTGTCTTCCCTGGGCTAAAATAATGCCAAAGTTGGATTTTTTGTTTCACTAAAAATAGTTGAGATTGTGTATATTGATGTTTTATGTTTTCATTAAATATTTTGTTTGAGGCATTGTATTTGCAGTTTGGTTTTTGAGTATTCAGGTGCCATAAAAATGTGAATGCTTCACATAATTGTAATACTGCATGAGATTAAACTAAACATGTAAGCGACCAAATAGGTGGCAACGGTCAAATAGTCATAGTGAACCGTGTAATCAAAGGGATATTCGTTGTAGTAGTTAATGTCAGTGTTTTTGATGATTTTAGTAATTTGAAGTTTGACTGTACTTTCAAGCGTTCTTGTACCTGTCGCAGTTGACTGGTTGGTAGATGCA

>Glyma12g06910 CACGTTCTTTAAAACACACCGCTTCCCTTTCCTCTCTTCAATTCAGTTCAAGAAATTGCTTTGCTGTAAGTTTCTGATTCCTTTCTTCAGAATTCCAAGTTTTCATTTCGTTAGGGACAAAAGATTGTGAGATAGAGTATGGCTGGAAAAGGCGAGGGTCCTGCTATCGGAATCGATTTGGGAACGACGTACTCTTGCGTCGGCGTGTGGCAACACGATCGTGTTGAAATCATAGCCAACGATCAGGGTAATAGAACTACCCCATCCTACGTGGCTTTCACCGATACAGAACGGTTGATCGGCGATGCGGCGAAGAACCAGGTCGCTATGAACCCAATCAACACTGTTTTTGGTAAGTACTCTCAAATCGTCCACTATTACCTTGATTCTCTGCTTTTTTTCTCTGTTTCACTGATTTTGGATGTCTACACATCGTGATTTATAATCATAAATTTCAATTATCTGTAATCTAACCCTAGTCAGTATTAATCTTTCACGATTCTTTCTTTTTAATTAAAAAATAGTGTAGCGAACTTAAACAGTTGTTTCATGTGTTAGTAAGCGTTTGTGAGACGTTTATTAACTTCTTACTTTGTGATTTTTTTTTTCAATTGTTCTTGATTTGAGTTTATTAATAAACTAGTATTTATTTGAATTAGTTGAGTCGTTTTTGGCGTGAGTCGTTTTTGGCGTGGTTTATTAAATAAGTACTTGCTTAATTGAATTGTTTTATATGTTTTGCATGATAAATTTATTAAATGGACACTTGCTTAAATAATCTGCGCGAAAGCTTTCCTGATTATGAGATTATATTGTGAATCGATTGGTGATTGTGTTGGTTTGGTTTGGCGCGTGCAGATGCTAAGCGTTTGATTGGAAGACGTTTTTCTGATGCATCGGTACAAAGTGACATGAAACTGTGGCCGTTCAAGGTTATTCCTGGCCCTGCTGACAAGCCTATGATTGTGGTGAATTACAAGGGGGATGAGAAGCAGTTTTCGGCGGAAGAGATATCTTCCATGGTTCTTATAAAGATGAAGGAGATCGCGGAGGCGTATCTAGGATCGACGATAAAGAATGCAGTTGTCACCGTCCCTGCTTACTTCAACGACTCACAGCGTCAAGCTACAAAGGACGCTGGTGTCATTTCCGGGCTCAACGTGATGCGAATTATCAACGAGCCTACAGCGGCCGCCATTGCTTATGGACTCGACAAGAAGGCCACTAGCTCTGGGGAGAAGAATGTTCTCATCTTCGACCTCGGTGGTGGGACTTTTGATGTCTCCCTTCTCACCATTGAGGAGGGTATTTTCGAAGTGAAAGCCACTGCTGGTGATACTCACTTGGGAGGTGAAGATTTTGATAACAGAATGGTGAACCATTTTGTTCAGGAATTCAAGAGGAAGAACAAGAAGGATATTAGTGGAAATGCCAGAGCATTGAGGAGGTTGAGAACAGCATGTGAGAGGGCCAAGAGGACTCTCTCTTCCACTGCTCAAACCACCATTGAGATTGATTCCTTGTATGAGGGTATTGACTTCTACACAACCATCACCCGTGCCCGTTTCGAAGAGCTTAACATGGACCTATTCAGGAAGTGCATGGAGCCCGTGGAGAAGTGTTTGCGGGATGCTAAGATGGACAAGAGCACCGTCCATGATGTTGTTCTTGTCGGTGGTTCTACTAGGATTCCCAAGGTTCAACAGTTGTTGCAGGACTTCTTCAACGGAAAGGAGCTTTGCAAGAGCATTAACCCAGATGAAGCTGTTGCCTATGGTGCAGCAGTGCAGGCTGCCATTCTCAGTGGCGAGGGTAATGAGAAAGTGCAGGATCTTCTGTTGTTGGATGTTACTCCTCTCTCCCTTGGGTTGGAGACCGCGGGAGGAGTCATGACTGTGTTGATTCCCAGAAACACAACCATTCCCACCAAGAAGGAGCAGGTATTTTCGACCTATTCCGACAACCAGCCTGGTGTGTTGATTCAGGTCTATGAAGGTGAACGTACGAGGACTCGTGATAACAATTTGCTTGGCAAATTTGAGTTATCTGGAATTCCTCCTGCTCCGAGAGGTGTTCCTCAGATCACCGTTTGCTTTGACATTGACGCCAACGGTATATTGAACGTGTCTGCGGAGGACAAAACCACTGGACAGAAGAACAAGATTACAATTACCAACGACAAAGGAAGGCTTTCTAAGGAGGAGATTGAGAAGATGGTGCAGGAAGCTGAGAAGTACAAGGCTGAGGATGAGGAGCATAAGAAGAAAGTGGAGGCCAAAAATACATTGGAGAATTACGCCTATAACATGAGGAACACAATTAAAGATGATAAGATTGCTTCCAAGCTGTCTGCTGATGATAAGAAGAAAATTGAGGATGCAATTGAGCAGGCTATCCAATGGCTCGATGGAAACCAACTTGCTGAAGCTGACGAATTCGAGGACAAGATGAAGGAGTTGGAGAGCATTTGTAACCCCATCATAGCAAAGATGTACCAAGGAGCTGGTGGTGATGCGGGTGGAGCCATGGATGAGGATGGTCCTGCTGCTGGCAGTGGAAGCGGTGCTGGACCCAAAATTGAGGAAGTCGATTAAGCAGACCAATTGTCATGGGTTGTTCTTGATTTTGCGAAGACCCTTTTGAAGTTTAGATAATAACCTGTCTTGTCATTATGTTTTGGATTTGGATTTTACTTTTCTCGTTTTTTTAATGTACTTTTCCCCGTTTCAACTTAACAATGATATAAGTTGCATATGGTCTATTGATTGTTTCGTCTCCATATTTGCTTAATTGCTTATGAAGTGGGTTGCTCTCCCATTTTTGAGAGTTG

>Glyma12g28750 ATGGCTTGCTCAAGCGCCCAAATACACGGTCTCGGAACCCCTTCCTCCCGAACCCTATTTTTAGGTCAGAGGCTAAATACCAAGGCCGCCTTTATCAAGCTCAAGTCCACACCTAGAAGACTCCGCCCTCTCAGAGTGGTTAATGAGAAAGTCGTCGGTATCGATTTGGGAACCACCAACTCCGCCGTGGCTGCCATGGAAGGCGGCAAGCCCACTATCATCACCAACGCCGAGGGCCAGAGAACCACGCCCTCCGTCGTGGCCTACACCAAGAATGGCGACAGGCTCGTTGGCCAAATCGCCAAGCGTCAGGCCGTCGTCAACCCCGAGAACACTTTCTTCTCCGTCAAGAGGTTCATCGGCCGCAAGATGTCTGAGGTCGACGAAGAGTCCAAGCAGGTCTCTTACAGAGTCATCAGAGACGACAACGGCAACGTCAAACTCGACTGCCCCGCCATTGGCAAACAGTTCGCTGCCGAGGAAATTTCTGCCCAGGTCTCTCTGTCGTCTCCGTCAATACTTGTTATTATTGTTGATTATTGATAATACCTTTACAACTGGTACTGAATTGTATGTTATATAGGTTCTGAGGAAGCTTGATGATGATGCTTCCAAGNNNNNNNNNNNNNNNNNNNNNNNNNNNNNNNNNNNNNNNNNNNNNNNNNNNNNNNNNNNNNNNNNNNNNNNNNNNNNNNNNNNNNNNNNNNNNNNNNNNNATCATTGATGAATTGGTTATTTTAAAGGGGTCGAATATCTTTTTCTTCTCTGGATAGTATATTATTTTTGTTGATTAATGCTTCTTATTGTAATTTATGTTTTTAGGCTGGTGTCTTGGCTGGAGATGTCAGTGATATTGTGCTGTTGGATGTCACTCCATTATCTTTGGGTCTGGAAACTCTAGGTGGTGTGATGACAAAAATTATTCCAAGAAACACTACTCTTCCCACCTCAAAGTCCGAGGTTTTCTCAACCGCTGCTGATGGACAGACCAGTGTAGAGATCAATGTCCTTCAGGGGGAGAGAGAATTTGTTAGGGACAACAAATCACTTGGTAGCTTCCGCTTGGATGGTATCCCTCCTGCGCCTCGTGGTGTTCCCCAGATTGAGGTGAAATTTGACATTGATGCCAATGGCATTCTATCCGTCACTGCTATCGACAAAGGCACAGGGAAGAAACAAGATATTACCATCACTGGTGCTAGCACCTTGCCTTCAGATGAGGTATCCACTATCGCTGACTATATTACTTGTATAATAGCCTGTGTATGTTGTGTTTCTTTTTATTATTATTATTATTGTTATTATTATTATTATTGTCTGTTGCTTGCTTCTTTTATGATGAAAGAAATAAATGGCAATGCGGATGGGACATTTGATGTAGGAAATTTTTTATGATGCTTATAACTATCTCTGGAATGAAATACAGGTGGAGAGAATGGTAAACGAAGCTGAGAAATTTTCGAAGGAAGACAAAGAGAAGAGGGATGCTATTGACACAAAGAACCAGGCAGACTCTGTGGTATACCAGACAGAAAAGCAATTGAAAGAGCTTGGAGACAAGGTTCCTGGCCCTGTGAAAGAGAAGGTTGAAGCAAAACTAGGAGAGCTTAAAGACGCCATTTCTGGGGGTTCAACCCAAGCTATCAAGGATGCCATGGCTGCACTAAATCAGGAAGTCATGCAGCTTGGTCAGTCCCTTTACAACCAACCAGGAGCTGCAGGCGCAGGAGGGCCAACACCACCACCCGGTGCCGACTCTGGCCCCTCAGAATCCTCAGGCAAGGGACCCGACGGTGATGTCATTGATGCAGATTTCACTGACTCTAAATGAGCAGGCAACTAGTTATTACCAATAGAGAGTATATGTTAGTATTTTTTCCCCCATTATATGTTTTCTTCTGTTCCAAATTCTTAATAATTTTTGTCAATGGAGAGTGGGTGCGTGTTTCCTTCAAATATTAGACACGGATTTCATAGGTTCGAATTGATGCTCGCTTTGTACCCTGGAATTTTGTCTCAGTAACAACACTGGTATTATTATGCGTGGCAAATTATTATAGGCTAAAAACGTTTCAGTAAGCTTATGAAACGTGATGACAGATTCTCAGGGAAAACGATCGATTCTTCCTACCTTTATAATACATTGCAAAAACACAAGCATAACAGAAATTGTTAATCGTTAACCAATGGTTTTTTTT

>Glyma13g10700 AAAAGCAAAACGACACTCGTTTGGTTTCAGTCATTATTATTTAAAATATTTTTATTGTTGATTTCATTTGCTCTTATTAAACTCACCACCACCACCACCACCACCACGCTCTCTTCCATTTTCCCGCAAAACAAACACATTCACATACTTTTCCCTCACTTTCTCGGCGGCCAAACGCCATAAAAATGGCGTCGAAGGTGGCGCTGATGGCGCTGTTCTCCGTCGCGCTCCTGTTCTCTCCGTCGCAATCCGCGGTCTTCAGCGTAGATCTAGGCTCCGAATCGGTGAAGGTGGCGGTGGTGAACCTCAAGCCCGGCCAATCCCCGATCTCCGTCGCGATCAACGAGATGTCCAAACGCAAATCGCCGGCGCTGGTCTCCTTCCATGACGGCGACCGCCTCCTCGGAGAGGAGGCCGCCGGCCTCGCCGCGCGCTACCCGCAGAAGGTCTATTCCCAAATGCGCGACCTCATCGCCAAACCCTACGCCTCCGCGCAGAGGATTCTCGACTCAATGTACTTGCCATTCGATGCAAAGGAAGATTCACGAGGCGGCGTGAGTTTCCAAAGCGAAAACGACGACGCTGTTTATTCCCCCGAGGAGCTGGTTGCCATGGTGTTAGGTTACACGGTGAATTTGGCAGAGTTTCACGCAAAGATTCAGATAAAGGACGCGGTGATCGCGGTGCCGCCGTACATGGGGCAGGCGGAGCGGAGAGGGCTGCTTGCCGCGGCGCAGTTAGCGGGGATTAACGTTTTGTCTCTGATAAACGAGCATTCCGGCGCGGCGCTGCAGTACGGGATCGACAAGGACTTCTCCAACGAGTCTCGGCACGTGATCTTCTACGACATGGGCGCGAGCAGCACCCACGCGGCGCTCGTTTACTTCTCGGCGTACAAGGGGAAGGAGTACGGGAAGAGCGTGTCGGTGAATCAGTTTCAGGTGAAGGACGTGCGCTGGGATCCGGAGCTCGGTGGCCAGCACATGGAGCTGCGGTTGGTGGAGTATTTTGCGGATCAGTTCAATGCGCAGGTTGGAGGTGGAATCGATGTCAGGAAGTTCCCCAAGGCTATGGCTAAGTTGAAGAAACAGGTTAAAAGGACTAAAGAGATACTTAGTGCTAACACAGCAGCTCCTATTTCAGTTGAATCGCTTCATGATGACGTCGACTTCAGGTACGTGGATTTTAAAATTGTATTTCTCATCAGCTTCTTCAGCATTCCTGATTTTTATGAATTTGTATGTTATTTCTCTATTGGACTGAACAAAAGTTGGTAGTTTGTAATGTGTAGATCATGGTGCAACCTTATTGTACAGTGTGTACTTTACCATCCTCTTTAGCTTATTAATACTTTATTAATATTATGAACAAGAACGGCAAGGAAAACAACAACCAATTTTTATAGCATATTATCATGGCAGACTAATATGGGAAATATTTAATCTAAAAGAATTTTGCACTTTTTCTTTTTTTGAAAAAGCCAATTCAACTGATATTTTTTCTAGGAGCACATTTTTTGTATTGGGGGGAGGGATGTTTTGTCTTTTCAAAATGATTTTTGATGGGAGTGAATTAGCATTTAGTCTCATTTAGTAGCATACTAAACCACTCAGTAATAAAACTTACTGTTTTTGTATGGGCTAAGTTTTATGTCAGTGATAAGATTATTGGGTATGTATCTAATAAGTACGGGTGTTGGAAACATATCCAGTATGGGTATTGGAATTGTATAGGAATACCCATGCATCATAGTTTCCCTGTAAAATAATTACAGCAGCAAAGGAAGGTAGCAAGAAGACTGTTGCCTTTGCTTGAACCGACTGCCTGAGAGTTGGTTAAGTGGCATCAGTCTTGAGAAATTGTGATATGACCTGCTCCAACCCTTTAGTAAAAACATGTTCTAGAATTTTCATTGGGTAAAAAATAGTTGGGAAAATTATAGAAAAAGGAATTGTTGATAACTGTATTTTCCATTAAAAAAAAATAGTTTAGCTTTCATATGTCAAACTAGTAGTTTCTTAAGTGGTTAGTTGTTTGCAATTAATTAACAAGAAGGAGCCTTAAGTTTTACTGGCTGGCTGAGAGTTGAGAAAGGGGCATCAGTCTTAGGAGTTGAGTTACATATAACCTTAAAGACAGCGCTGCATATTGGTTAATAGATAGGATTGGCTAGCATCTTAAATCTTATGGTAGAGATCAATCTGAAGGAAGTTGCTGTTTTCAAATGATGAAGAAATCTGAACAGGGGAAGTCAAGAAATACATCTAAAGAATGAAATTCCCTTATATGCTCAATCTTGTCAAGTGAAATGCATTGACTAATCTAAATAAAAATGACAAGGAAAATAAAATTTTACTTGATTATCAAGTTATGATTACTGCATATAATGCTTAAGATTCCTTCACACAAGGCTCCTTTTCTTACAGGAGCACAATAACCCGTGAGAAATTTGAAGAGCTTTGTGAAGACATTTGGGAAAAATCGCTCTTACCTGTGAAAGAGGTGCTTGAGAATTCTGGCCTGTCATTGGAACAAATATATGCAGTGGAGTTGATTGGAGGTGCCACCAGAGTGCCAAAATTACAGGTTTGAATAATCTATTTTCCTTCTTTGACAATGTGCACTTTGGGAAGTGTGGGATTTGATCTGCAGATATTTGTAAAATATTGAACTCCATGCTTGAGAGTCAGCAACATATGTTTACTGTATATATCATCTGTTATGTACATGATAAGCCTAGTTTCGAAAAATTAATGTTTCTTATTTCAGTATACAACTGTATCTGTATCTGGTAGTTGTATGCTGTCTGTATACTGCAGTTGCTCTGCTAGTATATTGTTAGCTGTTAAATGCAGCTTGTGCACATTCATCTGTTATTTAACAGAAGTATCTAACTGATCAATTCCACTGTTCAGTTAGATATTGGTCAAAATGTTTTGTTACAACTCTGTTCAACTGAACTGATTCAGTTGCAACTGTTTCCTGTACATTTAGAGTTGCACTCAGCATACAAGACTTTGTTTTTTTGCACCAGAAATACAGCTATACTGAGAGCATTTCAGACTGTCTCAGTTTTCTGTCCTTTTTCTCTCATTTTCCGTTCACTTGAACTTCAACAATTCTATAATGTATACTGTCAATCTAAGTTATTCATGCTGTCCAAGCTTGACTTTATTTTATATGAAGTTATGCACATATGTTTGCTACCTTTTTTTATAATATTGATTTGAATAATGAAGAAAAAGGAAGAGCTCAACTGATTGCTCTAATGTTAGCGTAAAGATTTCAACGAAGAGATTGGCAGCATGAATATAATTAAGTTTCATAACCTCTTTATTTCAAATCTGAATATAACTAAGTTTGAATATCAAGAATCCATGACATAGTTATCAACTTATATTTGGTATCTATTTATTGTTAAATGAAATTGCAGCACTATATTTTGTCTAAGAATTGTTTGATATAGTGTTTGAGCTTTACTTTCACAGATGTATTTTACTCAGCATTAATGTATAGAGGTCTGTTTATTGATACTCTGTTCATATTATTACAGGCTAAGCTTCAAGAATTCCTCAGGAGAAAAGAACTTGATAGGCATCTTGATGCTGATGAAGCAATAGTTCTTGGCGCAGCTCTGCATGCTGCAAATTTAAGTGATGGAATCAAATTGAACCGCAAACTAGGAATGATTGATGGTTCCTTATATGGATTTGTGGTTGAGTTGAATGGTCCTGATCTTTTAAAAGATGAAAGCTCTAGGCAGCTACTTGTGCCACGAATGAAGAAGGTCCCGAGTAAGGTAAATCAATTGATGATTCAATGCTGAATGTGACTACATATTATATGAAGAATGAATTTATCAATTCTATTATGAATTTATCAATTTTATTCTCCAATTTTATTAATCTGTTGTAGCATTTTTTACTCACCAGATGTTTAGATCCATTAATCATAACAAGGATTTTGAAGTTTCACTTGCTTATGAAAGTGAGAATCATTTGCCTCCTGGTGTTACCTCTCCTGAAATTGCTCGATACCAGATATCTGGTTTGACAGATGCAAGTGAGAAGTAAGGATAAAAAACTTGCTGAAGTTTTGTGGGCATATTGCTTCCTTAAGCCCCTTGAAGAATTGCTTTTCATGTAAAATTTAGAAACTCTAATGCCCTCCTATTTATGTTTCTCCTCTGTTATTCTAGATACTCATCTCGGAATCTGTCATCCCCCATCAAGACAAACATACATTTTTCTCTTAGTAGAAGTGGAATTCTTTCACTGGATCGGGCTGATGCTGTTATTGAAATAACAGAGTGGGTGGAAGTTCCTAGAAAGAATTTGACCATAGAGAATTCAACCGTTTCATCAAATGTTTCAGCTGAATCTGCTGCGGGTAATAGTTCTGAAGAAAACAACGAAAGCGTGCAAACTGATAGTGGGATTAATAAGACATCCAACATTAGTTCAGAGGAGCAAGCTGCTGCTGAGCCTGCTACAGAGAAAAAGCTGAAAAAGCGGACCTTTAGGGTACCATTAAAGGTGAAAGCTAACTGGCCACCTTTATTTTGTGACTATTTAAACTGGCTGGTGATTTTTGGGAATGAAACAAGTTCTTCTAATATAATTTTCTTGCTTCTTTGCTGTTTTTGCCTTTTGCTGGTGACAGATTGTAGAGAAGATAACTGGATTTGGAATGTCTCTATCACAAGATTTTCTTGCTGAAGCCAAAAGAAAACTACAAGTACTAGATAAAAAAGATGCAGACAGAAAAAGAACAGCTGAGTTAAAAAATAATTTAGAAGGATATATATATACAACTAAGGAAAAGGTTGGTAGAAAAACTATATGTTTTATGATTTTTGTTATGTTAACAGCCTTAATCTATTATGTGATGGGTAAATATTTATCCTTTAAATATTTCCAATTTTCTTTCAGAGATTCATTTAAAAGATTGACCTTTAATTAGTTGACTATGTCTAACTTTATACTGAGGGATTTTAACTCTTGGTCTTGATTATTGTTTTTGAGAGAAAAATCTATCAGAAGAGAATTAAGAAATGAAAGAATGGGGATGAGCATTTTTTTAAACCCAAAGTTTCCTAGCCTCCTGCCAAATTAACTTGAGATAATAATGCCCCATCCATTGATCTTCCTTTTTATTCTTGTCCTTGCATATAGCTTCATCTGAATTTGGTTGGAGTGTTTTTCTGATTGCCATGTATTTGTATTGGGATTTGGGAGAGTGGGATTTAGGAATGATGCTTCTAAAACTATTTTATAGGTTAACTGGCTGTATAAACATTATGCCTGTTGTTTTTCATTTTGGAATGTTTAAAATGGACTAATTTAACTATTTGTTTTTCTAATTGTTAATATGGCTATTTTATTCAATTTGTTATTTATGCTTCTCATCTTTTTATCAGATTGAAACTCTTGAGGAGTTTGAAAAAGTTTCTACAAGTGAGGAACGCCAGTCCTTCATTGAGAAGCTTGATCAGGTTAACCCTTTTGTTTGGGCAACTGAAAAGAAATGTTATTGATAAGAATATTGACTTAAGCTTTTTGCATTCTGTCAGGTGCAAGATTGGTTGTATACAGATGGTGAAGATGCCAATGCCACAGAGTTTCAAGAGCGTCTAGATCAGTTAAAAGCTGTTGGAGATCCAATTTTCTTCAGGTATGTTATATTCAGTATAACTATTAATTTCTTTCTGCATGCATTTTTGTGGTAAAGTTTGTTCTGAATTGGTTAACATTTGTTTCAGGTTAAAAGAGCTTACAGCTCGGCCAGCAGCAGTTGAGCATGCTAATAAATACATTGATGAGTTGAAACAGGTATCATCAACAATCATTACATGTAAAGTGGTGCTTTAGTGTTTTTTATTTTCTTAATGTTCTTTTTGAAGCCGAAAATTGGTTTTTGGTAGGGTTGAATTATGTCGATTGATCCTTGTAGCTAATCACTCCTAGGTTGTGTTTGGATGAAGACTTTTAAGATTTCAAGGAATTTGAGAAATGCCTTAAATTTGAATTGCTTTTATTAAAATTTCCTTGTTTTTTAAATGCTTTGCTTGGATAAAGCAATACATATTTTTTAAATTTCTAATTCTTAGTTTAGATAGGGTAACTCAATTTCCATCATATGCAAAATTTCATTTCAACAAGCAAGGAGGGGGGGGGGGGGGAGATAGATAGAGGAGATAGAGATGGGAAACGGGGAGGAGGGGAGAAGAGATAGATAGAAAGAGAAAGTGAGAATGTATTCAAATTCTTACTTTTTTGGTTGAATTTGAAATTCTATAATTTTATATAAAGAAAATATTTTCACGTTACTAAAATTTTAAATACACTATGCAAGGAATATATTTTAATGCAAAAAAATTTAAATTCTCACTTAAATTGGATTACTAACTTAAATTTCTCTATCTAAACACATTACTAGTGAAATTAGGTTTTGGTGGACATAGTTGTTTTTGCTAATCCCTTTGATAGATGGGTAGTTTATTGTTGTAAACATAAGTTGGATCAATAGGAACTGTAATATGATAACAATACTGTCTCTTTATTATTTCTCGTACATTAATTCAACTTCTTTCATTTGAAAGAATGTAGAGATTGGTCATCTGTTGCCCTTGTTGAGAACTAGAGATATTAGGTATCGACTAGGACAAGGTTCAAATCTGATACAACAGGCAACTTTCATTCAAGTGGTTATCTGTTGTTTATCCAGTTTTCATCATGTCAGCACTCATGTGCCCATTCATAGAAAAACATATGGACACATTCACACCACTTCATGAAATCATATAATCAAAAAACAATGAAAAGGCATTAGTTAGGTGAACCAAACATGTACCACGAGCAATTGATTTGCTGAAAGATGTCTGAAGCAGTATATTGTATATTGTGATTTATAAAATTATCTATTCATCTTCAGCCAGTTATTTTCTAATGTGAAACATTTATGACATTTGCAGATTGTTGAGGAGTGGAAAGCAAAGAAGTCTTGGCTTCCACAAGAACGAGTAGACGAGGTACTTGGTTTAACTTGATTTCAATAGCTTTGAATCTATTAAAGCTGATGTGTTACCTTCAGAAAATTCATTGTCTTTTGGTACCTAACTTGCAAGGTCATAAAAAGTTCTGAAAAATTGAAGAATTGGTTGGATGAGAAAGAAGCTGAGCAAACAAAGTAAGTTCTTATCATAGTAATTTAGTTGTTATTGAAGTTTTTTTTCACCTTGTCTTTTCAATATTGTGTGGACTTTGCTAGAGTCAAAAAAAGTATCTTCATGAGCGGCTGCCTGTATAAACATTTACCTCTATTTTTCTTTAATTGGGTATGATATTCCTTGAAAAGTGCAATCTTAATGGTTATGAATCTAAGTTATCTTTGAAAATGGTTTCCTTTCATCTGCTACTGTTAGCTTGTATAGCACAAAATATGATCAATGACAATGAACTACATAATGGAATATTAGTCTGCATATGAACGTCACTTCTGCCAACAGGACGTGTATGTGATGCACAGCGGATTGTGCTACCCAAGAATCTGTGGAATTCTTGAATAATAAAACCAGTCTATTAAAATATTAAGATAGCCTAGATTCCACTTGTTTTGAGCTTTAATGATTGTTCCCTGCAAGGTTTCATTGTCTAATATATTCATTTTCTAGATAAAAAATTCCTTAAGATGTTTTTTTGAGGGTGAGCCTCCCTCCCTCCCTTTGCTCTCTGTAAGGACTGTGTAAATGATTTCAGGATGACCAATGGATCTGAAGCATAACGTTGATGGCTTCAAAGTTTTAGATTATTTCCCTTTTTTGAGATCATATTTCATTTATGCACTTGTGAATATTTAGAAAATGGACCGTCTAGCTTTGTAAAGTATCACTTTTCCACTCTGCTGACTGCCAACTTGAGTTTGATCACTGAGGAAATTGCTATGTGCATACAACAGTTTTAATGTAACTGCCTAACTGGTTATTAATTTCTTATTCTGTACTTGTTTTGAAGGACTTCTGGATTCAGTAAGCCAGCATTTACATCTGAAGAAGTATATCTGAAGGTGCTTGATCTGCAAACCAAGGTACCCGTCTCTTCTACTTGTCTAATTCAAAAGACTCTGTAATCGCATGTGATTTTACAGGTCTAAGAAAACAGTGCCATTTCTTGTTGACTGTACATTTACAGGTTGCCAGTATTAATAGAATTCCCAAGCCCAAACCTAAGGTTCAGAAGCCCGTAAAAAATGAAACCGAAAGCAGTGAGCAGAATACAGAGAATTCTGATTCTAACTCAGCTGATAGTTCCTCTTCAAGTGATTCATCCGTTAACAGTTCAGAAGGCACAAGCGAAGAGACGGTAACTGAGCAAACTGAAGGTCATGATGAGCTATGATTAACCTGGCATACATGTTAGGCCGGTTTGAATCATCTACCTGCAACGTTTTTATAGTCAGAGATGTAGACTGGTAATGGTAGAATTAATAGGTGAAAAGGAGGCAGTGTCGGTTCTCGGCGAGCAGGAGAATAGAGTTGAGGCAACATTTGCTGGTTGAGTTCTAAAATTTTGAGGTTAGATGGCTACTACTGTAGTAGATGATTTATTACCCTTTTATATTTAAGGGGGCAACTTGTAATTTGCAGTCATATGAAAGCTTGTATTTGCCGAAACACAATGTGATAGGGGAAGAAAAAAAAAGAAAAACTCACGAGATTTGATTTGCTCAGAACATATAGGAATTTTGCTTGAGTGACTCGTAGTTCTAAACGATTTTTGTTTTTAATTAATTTAGCAGATACATCTTATCTTATTTTTAATTTTCAAGTTTTCTCATTCTGTCAAAAAAAAAAAAATTAAGTTTTCTCATAGTGTTAAGCAGTGCATGATTTTATTTATAACTAGATTAGCACCGAGTATAGTC

>Glyma13g19330 CACTCGCTGACACAACAAACGAAAGCATTATGGCTGGCAAAGGAGAGGGTCCAGCCATCGGAATCGATCTCGGAACCACGTACTCGTGCGTCGGTGTCTGGCAACACGATCGCGTCGAGATCATCGCCAACGACCAAGGTAACAGAACGACGCCGTCTTACGTCGGGTTCACCGACACCGAGCGTCTCATTGGTGATGCCGCAAAAAATCAGGTCGCCATGAACCCCATCAACACCGTCTTTGATGCCAAGAGATTGATTGGTCGTAGATTCAGTGATGCCTCTGTTCAGAGTGATATTAAACTGTGGCCTTTTAAGGTTTTGTCTGGACCTGCTGAAAAGCCAATGATTCAGGTCAGTTACAAGGGTGAGGATAAGCAGTTTGCTGCAGAGGAAATCTCTTCTATGGTGCTAATGAAAATGCGTGAGATTGCTGAAGCTTATCTTGGCTCTTCAATCAAGAATGCCGTTGTTACTGTCCCTGCTTACTTTAACGATTCTCAGCGTCAAGCTACCAAGGATGCTGGTGTTATTGCTGGTCTCAATGTGATGCGTATTATCAATGAGCCTACTGCTGCAGCCATTGCTTATGGTCTTGACAAGAAGGCTACCAGTGTCGGCGAGAAGAATGTCTTGATTTTTGATCTCGGGGGTGGTACTTTTGATGTCTCTTTACTTACCATTGAGGAGGGTATCTTTGAGGTGAAAGCCACAGCTGGGGACACCCATCTTGGAGGTGAGGATTTTGATAACAGGATGGTCAACCACTTTGTTCAGGAATTTAAGAGAAAGAACAAGAAAGATATCAGTGGCAACCCCAGAGCACTTAGGAGGTTGAGGACTGCCTGTGAGAGGGCAAAGAGAACTCTATCTTCAACTGCCCAAACCACCATTGAAATTGATTCTCTCTATGAGGGAATTGATTTCTACTCCACCATTACTCGTGCTAGATTTGAGGAGCTCAATATGGATCTCTTCCGCAAGTGCATGGAACCAGTTGAGAAGTGTTTAAGAGATGCTAAGATGGACAAGAGGACTGTCCATGATGTTGTACTTGTTGGTGGTTCCACCAGAATTCCCAAGGTGCAACAACTCCTGCAAGATTTCTTTAATGGGAAGGAGCTCTGCAGAGCATCAATCCTGATGAGGCTGTGGCATATGGTGCTGCAGTTCAAGCTGCTATCTTGAGTGGTGAGGGTAATGAGAAGGTTCAGGATCTTCTCCTTCTTGATGTCACCCCTCTCTCTCTTGGGTTGGAAACTGCTGGTGGGGTGATGACAGTCTTGATCCCCAGGAACACTACTATCCCAACAAAGAAAGAACAGGTTTTCTCAACCTATTCTGACAACCAGCCTGGTGTGTTGATCCAGGTTTATGAAGGTGAGAGAACAAGGACTAGAGATAACAACTTGTTGGGCAAATTTGAACTTTCTGGCATTCCTCCAGCTCCCAGGGGTGTTCCTCAAATCACAGTGTGCTTTGACATTGATGCCAATGGTATCTTGAATGTCTCTGCCGAAGACAAAACTACCGGCCAAAAGAACAAGATCACCATTACCAATGACAAGGGTAGGTTGTCAAAGGAAGAGATTGAGAAGATGGTTCAAGAGGCTGAGAAGTACAAATCAGAAGATGAAGAGCACAAGAAGAAGGTAGAGGCCAAGAATGCATTGGAGAACTACTCATACAACATGAGGAATACCATTAAAGACGAGAAGATTGGAGGAAAACTTGACCCAGCTGACAAGAAAAAGATTGAGGATGCCATTGAGCAGGCCATCCAGTGGCTAGACAGCAACCAGCTTGGAGAGGCAGATGAATTTGAGGACAAAATGAAGGAGTTGGAAAGCATCTGCAACCCTATCATTGCCAAGATGTACCAGGGTGGTGCTGGTCCAGACGTGGGTGGTGCAATGGACGATGATGTTCCTGCTGCTGGAAGCGGTGCGGGGCCAAAGATTGAGGAAGTAGACTAAATTTAGTTTATTTCTGCTTTTGATTTACTATTTATTTTTAAATGTCTGATTTCATGTCCTTTGGTTGGATTTAGTTTCTCAAGAAAGACAATATTTGGTAGTATGTCATGTCGATTTATGTTTTCAGTATCATGTTACCCTTTTCAGTTTTCACTGTGTTTTAGTGGACACATACAGGCTTTAAAGCTTATGTTATAAGCTGGTTTTTTAGTATGTTTATGTTTCTATAGCTTGTTCGTTACGGATAACTTAAAGTTCTAAATTCGTATCTGATGAGTCATTCTACATTCCTACGAATGACCTCTTGAAAAATTACACGGTAAATGGATAACGGAGGATAGCGTTCTGAAGCT

>Glyma13g19331 ATGGCTGGCAAAGGAGAGGGTCCAGCCATCGGAATCGATCTCGGAACCACGTACTCGTGCGTCGGTGTCTGGCAACACGATCGCGTCGAGATCATCGCCAACGACCAAGGTAACAGAACGACGCCGTCTTACGTCGGGTTCACCGACACCGAGCGTCTCATTGGTGATGCCGCAAAAAATCAGGTCGCCATGAACCCCATCAACACCGTCTTTGGTAAGATCCCCCTGCTTCAAATTTAGTATTGTACGGACACGGTCTTTTTTATGACATGTTTTTTTTTCACGTTGATGTTGCTGTTGTTGGTGTTTTTTTTTCGTGTTTGACTTGGAATAGATCGGTGTTTTTTTAGTTGTTCTTTTTTTTTTGGTAATTTTTTGTCCTGTTTGGTTTTAGTTTGCTGATGTAAGTGCCTTTTGCATTTCTTCTCCGGGGTGTTTGATATGGTTTTGATGTATACAAATAAGGAAAGCTGAATACCTTTTTTAACCCTGATATCATCCTCTTTAAGAAATTTTTATGCATTAAACTAGTTGTTAAGTGAAAGCATGATGATATTCTTATTAAATAAATTAATAAATAAACTAGTTGTTAAGTTCGTTTCCCTAAAATACTGCAATTGAATTGACATTGACTTGCAGATCAGGTTGTTTTAGTATTTGTTCAGTGCCTTTTTCTCTCAGAAGATGTTGGTTTGTATTTGTTAAGTGAAAGCATACTGTTGTCATTATTAAGTAAAACAGTTAGGGTTTTGACTATATATTGACTGTTTTGACTAAGTAGATTGAATTGTCATTGAGGTTGTTTTAGCATTTGTTCAGTGCCTTTTCTCTCAGGATAGATGTTGGTTTGTATTTGTTAATGTGAATTATTATAGGATTGCATATTTATATTTTTCTTTGTGCCTCTTGCAGATGCCAAGAGATTGATTGGTCGTAGATTCAGTGATGCCTCTGTTCAGAGTGATATTAAACTGTGGCCTTTTAAGGTTTTGTCTGGACCTGCTGAAAAGCCAATGATTCAGGTCAGTTACAAGGGTGAGGATAAGCAGTTTGCTGCAGAGGAAATCTCTTCTATGGTGCTAATGAAAATGCGTGAGATTGCTGAAGCTTATCTTGGCTCTTCAATCAAGAATGCCGTTGTTACTGTCCCTGCTTACTTTAACGATTCTCAGCGTCAAGCTACCAAGGATGCTGGTGTTATTGCTGGTCTCAATGTGATGCGTATTATCAATGAGCCTACTGCTGCAGCCATTGCTTATGGTCTTGACAAGAAGGCTACCAGTGTCGGCGAGAAGAATGTCTTGATTTTTGATCTCGGGGGTGGTACTTTTGATGTCTCTTTACTTACCATTGAGGAGGGTATCTTTGAGGTGAAAGCCACAGCTGGGGACACCCATCTTGGAGGTGAGGATTTTGATAACAGGATGGTCAACCACTTTGTTCAGGAATTTAAGAGAAAGAACAAGAAAGATATCAGTGGCAACCCCAGAGCACTTAGGAGGTTGAGGACTGCCTGTGAGAGGGCAAAGAGAACTCTATCTTCAACTGCCCAAACCACCATTGAAATTGATTCTCTCTATGAGGGAATTGATTTCTACTCCACCATTACTCGTGCTAGATTTGAGGAGCTCAATATGGATCTCTTCCGCAAGTGCATGGAACCAGTTGAGAAGTGTTTAAGAGATGCTAAGATGGACAAGAGGACTGTCCATGATGTTGTACTTGTTGGTGGTTCCACCAGAATTCCCAAGGTGCAACAACTCCTGCAAGATTTCTTTAATGGGAAGGAGCTCTGCAGAGCATCAATCCTGATGAGGCTGTGGCATATGGTGCTGCAGTTCAAGCTGCTATCTTGAGTGGTGAGGGTAATGAGAAGGTTCAGGATCTTCTCCTTCTTGATGTCACCCCTCTCTCTCTTGGGTTGGAAACTGCTGGTGGGGTGATGACAGTCTTGATCCCCAGGAACACTACTATCCCAACAAAGAAAGAACAGGTTTTCTCAACCTATTCTGACAACCAGCCTGGTGTGTTGATCCAGGTTTATGAAGGTGAGAGAACAAGGACTAGAGATAACAACTTGTTGGGCAAATTTGAACTTTCTGGCATTCCTCCAGCTCCCAGGGGTGTTCCTCAAATCACAGTGTGCTTTGACATTGATGCCAATGGTATCTTGAATGTCTCTGCCGAAGACAAAACTACCGGCCAAAAGAACAAGATCACCATTACCAATGACAAGGGTAGGTTGTCAAAGGAAGAGATTGAGAAGATGGTTCAAGAGGCTGAGAAGTACAAATCAGAAGATGAAGAGCACAAGAAGAAGGTAGAGGCCAAGAATGCATTGGAGAACTACTCATACAACATGAGGAATACCATTAAAGACGAGAAGATTGGAGGAAAACTTGACCCAGCTGACAAGAAAAAGATTGAGGATGCCATTGAGCAGGCCATCCAGTGGCTAGACAGCAACCAGCTTGGAGAGGCAGATGAATTTGAGGACAAAATGAAGGAGTTGGAAAGCATCTGCAACCCTATCATTGCCAAGATGTACCAGGGTGGTGCTGGTCCAGACGTGGGTGGTGCAATGGACGATGATGTTCCTGCTGCTGGAAGCGGTGCGGGGCCAAAGATTGAGGAAGTAGACTAA

>Glyma13g29580 ATGGCACCCGGAAATGTCAAAGCTATAGGCATTGACTTGGGAACAACCTATAGCTGCGTGGCAGTGTGGCAACACAACCACGTCGAGGTCATTCCCAATGACCAAGGCAACCGCACAACCCCTTCCTACGTTGCCTTCACCGACACCCAAAGGTTGTTGGGTGACGCTGCCATCAACCAGCGATCCATGAACCCACAAAACACCGTCTTTGACGCCAAACGTTTGATAGGTCGCAGATTCTCCGACCAATCCGTCCAGCAAGACATGAAGCTCTGGCCTTTTAAGGTTGTCCCTGGAAACAGAGACAAGCCCATGATTTCTGTCACATACAAAGACGAAGAGAAACTCCTTGCAGCCGAAGAGAANNNNNNNNNNNNNNNNNNNNNNNNNNNNNNNNNNNNNNNNNNNNNNNNNNNNNNNNNNNNNNNNNNNNNNNNNNNNNNNNNNNNNNNNNNNNNNNNNNNNAGAGACAAGCCACCAAGGATGCCGGGAAAATCGCGGGTTTGAACGTGTTGAGGATCATCAACGAGCCAACCGCAGCTGCTATTGCTTATGGTTTGAACAAGAAAGGGTGGAGAGAAGGTGAGCAGAACGTGCTTGTGTTTTGACCTCGGTGGTGGTACTTTTGATGTTTCATTGGTTACAATTGATGAAGGAATGTTTAAGGTTAAGGCCACGGTGGGAGATACCCATTTGGGAGGTGTTGACTTTGATAACAAAATGGTTGACTATCTTGTGAGTATTTTCAAGAGGAGGTACAAGAAGGACATTGGTGAAAATCCCAAAGCTCTTGGAAGGTTGAGGTCAGCGTGTGAGAAAGCTAAGAGGATACTCTCTTCAAGTTCTCAAACCACTATTGAGCTTGATTCTTTATGTGGGGGGGTTGATTTACATGCAAATTTTTCAAGGGCCTTGTTCGAGGAATTGAACAAGGACTTGTTCATGAAGTGTATGGAGACGGTGGAGAAGTGCCTCAAGGAGGCAAGGATTGCTAAAAGTCAAGTTCATGAGTTTGTTCTTGTAGGAGGGTCTACTAGAATTCCAAAGGTGCAGCAACTTTTGAAGGACATGTTCAGTGTTAATGGTAACAAGGAGCTTTGCAAAAGCATCAACCCTGATGAGGCCGTGGCGTATGGTGCGGCGGTTCAGGCGGCGATTTTGAGTGGCGAAGGAGACAAGAAGGTGGAGGATTTGTTGCTGCTGGATGTGATGCCGCTTAGTCTTGGAATTGAGACTGATGGTGGTGAGATGTCAGTGTTGATTCCCAAGAACACCATGATCCCCACCAAGAGGGAGAGTGTTTTCTCCACGTTTTCTGATAATCAAACAAGCGTTTTGATCAAAGTTTTCGAAGGGGAGCGAGCGAAGACAGAGGATAACTTCCTTCTTGGGAAGTTTGAGCTTTCTGGTTTCACTCCATCGCCAAGGGGAGTTCCACAGATCAATGTTGGGTTTGATGTTGATGTTGATGGCATTGTGGAAGTCACTGCTAGAGATAGGAGCACGGGGCTGAAGAAGAAGATCACGATCAGCAACAAGCATGGGAGGTTGAGTCCTGAAGAGATGAGGAGAATGGTGAGAGATGCAGTGAGGTATAAGGCAGAGGATGAGGAGGTGAGGAACAAGGTGAGGATAAAAAACTTGCTTGAGAATTATGCTTTTGAAATGAGGGACAGAGTGAAGAACCTTGAGAAGGTTGTGGAGGAGACCATAGAATGGCTTGACAGAAACCAATTGGCTGAAACTGATGAGTTTGAGTACAAGAGGCAGGAGTTGGAAGAAAAGGTTTTGAAGTTTATGTAA

>Glyma13g29590 TTCCCCTGTGACTCACTCTCTTCCTTCACTGCTAAACTCTTCCAATGGCACAAAAAAGTCAAAGCCATAGGCATTGACTTGGGCACGACCTACAGCTTCTTTGCAGTGTGGCAACACAACCGCGTCGAGGTCATTTCCAACGACCAAGGCAATCGCACCACCCCTTCCTACGTTGCCTTCTCCGACACCCAAAGGTTGTTGGGTGACTCCGCCATGAACCAGCGATCCATGAATCCGAAAAACACCGTCTTTGACGCCAAACGTTTGATTGGTCGCCGATTCTCCGACCAAACCGTTCAGCAAGACATGAAGATGTGGCCTTTTAAGGTTGTCCCTGGAAACAAAGACAAGCCCATGATCGCGGTCACTTACAAAGGCGAAGAGAAACTCCTTGCCCCAGAAGAGATATCTTCCATGGTTTTGTATAAGATGAAGGAAGTTGCCGAAGGGTACTTGGGTCATTTCATAAAAGATGCGGTTATCACTGTCCCCGCTTACTTCAGCAACGCGCAGAGACAGGCCACGAAGGATGCGGGGAAAATCGCGGGTATGAACGTGTTGAGGATCATCAACGAGCCAACCGCTGCTGCTATTGCTTATGGGTTGGACAAGAAAGGATTGAGAGTTGGTGAGCAGAACGTGCTTGTGTTTGATCTCGGTGGGGGTACTTTTGATGTTTCCTTGGTTACTATTTATGAAGGCATGTTTAAGGTTAAGGCCAGTGTTGGGAGACACTCATTTGGGTGGTGTGGATTTTGATAACAGATTGGTGAACCATCTTGTGAATGTGTTTAGAGAGAAGCACAAGAAGGATATTAGTGGGAATGCGAAAGCTTTGGCGAGGTTAAGGTCGGAGTGCGAGAAAGCAAAGAGGATTCTGTCTTCGACTTCTCAGACAACGATTGAGCTTGATTGTTTATACGAAGGGCTTGATCTGTATGCCCCTGTTACAAGGGCCTTGTTCAACGAACTGAACAAGGACTTGTTCATGAAGTGTATGGATACAGTGGAAAAGTGTCTCCTTGAGGCAAGGATTGATAAGATCCAAGTTCATGAGATTATTCTTGTTGGTGGGTCCACTAGAATTCCAAAGGTACAACAACTTCTGAAGGACATGTTCAGTGTCAATGGTAACACCAAAGAGCTTTGCAAAGGCATCAACCCTGATGAAGCTGTGGCGTACGGTGCTGCAGTTCAAGCAGCGATTTTGAGCGGTGAAGGAGATAAAAAAGTGGAGGAATTGTTGTTGCTGGATGTGATGCCACTTAGTCTTGGATTTGAGGGTGCTGGTGGTGTTATGTCAGTGTTGATCCCCAAGAACACCATGATCCCCACCAAGAAGGAGAGGATTTGTTCTACCTTCTATGACAATCAGAAATCTTTTAATGTCAAAGTGTTCGAGGGAGAACGAGTTAAGACAAAGGATAACTTCTTTCTCGGCAAGTTTGTCCTCAAAGGGTTCGATCCATTACCAAAGGGAGTGCCACAAATCAACGTTATCTTTGATGTGGATGCTGATGGCATTGTGGAGGTCACCGCAGAAGATAAAGCCACAGGAATAGAAAAGAAGATCACAATCAACAACAAGCACGGAAGGCTGAACCCGGAAGAGATAAGAAGAATGGTGAGAGACTCAAAGAAGTACAAGGCAGAGGATGAGTTGGCAAAGAAGAAGGTGAAGGCGAAGAACGCACTAGAGAATTACGCTTATGAGATGAGGGAGAGAGCAAAGAAGATTGAAGAGGCAGTGGAGGAAACCATAGAGTGGCTAGAGTGTAACCAATTGGCAGAGATAGGGGAGTTTGATTACAAGAAGCAGGAGCTAGGAAGGTACCTAAGAGATGATCCAAATTATAGTAACACCTCAAATTCTGGTGTTAAGGTTCCTATATGGTGTACATGGAGGTGTCCACCTAATGGGTGGGTTTGTCTCAACACAGATGGTTCGGTGTTTGAGAACCATAGAAACGGGTGTAGTGGGAGTGCTTGTGGGGGTCTAGTTCGTGATTCCTCGGGATGTTACCTAGGTGGTTTCACAGTTAACTTAGGCAACACATCGGTTACTTTAGCGGAGCTATGGGGTGTTGTTCACGGTCTGAAGTTGGCGTGGGATCTTGGTTGCAAGAAGGTGAAGGTGGACATTGATTCTGGTAATGCTCTTGGTCTTGTTAGGCATGGCCCTGTGGCTAACGACCCTGCTTTCGCGTTGGTTTCGGAGATCAACGAGCTTGTTCGGAAAGAGTGGTTGGTAGAGTTCTCGCACGTGTTTAGAGAATCTAACCGTGCGGCTGATAAGTTGGCTCACTTGGGACACTCGCACTCTCTGGAATCGGGTGCGAAGCGATTCTCGGACCCACCCTCTGCCCTTGTTGCTATTCTTCAAGATGACTTGGCAGGGCTTGCCAAGCAACGTGGTGTTAATTAAGTGTAAGAGACTGTTGACCAAGCAATGTAAATTAGTTAGAGCTTGCATTCTCGATCATCAAAGTATTTTGACTTTGACTGAGTGTGTACATAAAGTTTATCTGA

>Glyma13g29591 TTCCCCTGTGACTCACTCTCTTCCTTCACTGCTAAACTCTTCCAATGGCACAAAAAAGTCAAAGCCATAGGCATTGACTTGGGCACGACCTACAGCTTCTTTGCAGTGTGGCAACACAACCGCGTCGAGGTCATTTCCAACGACCAAGGCAATCGCACCACCCCTTCCTACGTTGCCTTCTCCGACACCCAAAGGTTGTTGGGTGACTCCGCCATGAACCAGCGATCCATGAATCCGAAAAACACCGTCTTTGACGCCAAACGTTTGATTGGTCGCCGATTCTCCGACCAAACCGTTCAGCAAGACATGAAGATGTGGCCTTTTAAGGTTGTCCCTGGAAACAAAGACAAGCCCATGATCGCGGTCACTTACAAAGGCGAAGAGAAACTCCTTGCCCCAGAAGAGATATCTTCCATGGTTTTGTATAAGATGAAGGAAGTTGCCGAAGGGTACTTGGGTCATTTCATAAAAGATGCGGTTATCACTGTCCCCGCTTACTTCAGCAACGCGCAGAGACAGGCCACGAAGGATGCGGGGAAAATCGCGGGTATGAACGTGTTGAGGATCATCAACGAGCCAACCGCTGCTGCTATTGCTTATGGGTTGGACAAGAAAGGATTGAGAGTTGGTGAGCAGAACGTGCTTGTGTTTGATCTCGGTGGGGGTACTTTTGATGTTTCCTTGGTTACTATTTATGAAGGCATGTTTAAGGTTAAGGCCAGTGTTGGGAGACACTCATTTGGGTGGTGTGGATTTTGATAACAGATTGGTGAACCATCTTGTGAATGTGTTTAGAGAGAAGCACAAGAAGGATATTAGTGGGAATGCGAAAGCTTTGGCGAGGTTAAGGTCGGAGTGCGAGAAAGCAAAGAGGATTCTGTCTTCGACTTCTCAGACAACGATTGAGCTTGATTGTTTATACGAAGGGCTTGATCTGTATGCCCCTGTTACAAGGGCCTTGTTCAACGAACTGAACAAGGACTTGTTCATGAAGTGTATGGATACAGTGGAAAAGTGTCTCCTTGAGGCAAGGATTGATAAGATCCAAGTTCATGAGATTATTCTTGTTGGTGGGTCCACTAGAATTCCAAAGGTACAACAACTTCTGAAGGACATGTTCAGTGTCAATGGTAACACCAAAGAGCTTTGCAAAGGCATCAACCCTGATGAAGCTGTGGCGTACGGTGCTGCAGTTCAAGCAGCGATTTTGAGCGGTGAAGGAGATAAAAAAGTGGAGGAATTGTTGTTGCTGGATGTGATGCCACTTAGTCTTGGATTTGAGGGTGCTGGTGGTGTTATGTCAGTGTTGATCCCCAAGAACACCATGATCCCCACCAAGAAGGAGAGGATTTGTTCTACCTTCTATGACAATCAGAAATCTTTTAATGTCAAAGTGTTCGAGGGAGAACGAGTTAAGACAAAGGATAACTTCTTTCTCGGCAAGTTTGTCCTCAAAGGGTTCGATCCATTACCAAAGGGAGTGCCACAAATCAACGTTATCTTTGATGTGGATGCTGATGGCATTGTGGAGGTCACCGCAGAAGATAAAGCCACAGGAATAGAAAAGAAGATCACAATCAACAACAAGCACGGAAGGCTGAACCCGGAAGAGATAAGAAGAATGGTGAGAGACTCAAAGAAGTACAAGGCAGAGGATGAGTTGGCAAAGAAGAAGGTGAAGGCGAAGAACGCACTAGAGAATTACGCTTATGAGATGAGGGAGAGAGCAAAGAAGATTGAAGAGGCAGTGGAGGAAACCATAGAGTGGCTAGAGTGTAACCAATTGGCAGAGATAGGGGAGTTTGATTACAAGAAGCAGGAGCTAGGAAGGTACCTAAGAGATGATCCAAATTATAGTAACACCTCAAATTCTGGTGTTAAGGTTCCTATATGGTGTACATGGAGGTGTCCACCTAATGGGTGGGTTTGTCTCAACACAGATGGTTCGGTGTTTGAGAACCATAGAAACGGGTGTAGTGGGAGTGCTTGTGGGGGTCTAGTTCGTGATTCCTCGGGATGTTACCTAGGTGGTTTCACAGTTAACTTAGGCAACACATCGGTTACTTTAGCGGAGCTATGGGGTGTTGTTCACGGTCTGAAGTTGGCGTGGGATCTTGGTTGCAAGAAGGTGAAGGTGGACATTGATTCTGGTAATGCTCTTGGTCTTGTTAGGCATGGCCCTGTGGCTAACGACCCTGCTTTCGCGTTGGTTTCGGAGATCAACGAGCTTGTTCGGAAAGAGTGGTTGGTAGAGTTCTCGCACGTGTTTAGAGAATCTAACCGTGCGGCTGATAAGTTGGCTCACTTGGGACACTCGCACTCTCTGGAATCGGGTGCGAAGCGATTCTCGGACCCACCCTCTGCCCTTGTTGCTATTCTTCAAGATGACTTGGCAGGGCTTGCCAAGCAACGTGGTGTTAATTAAGTGTAAGAGACTGTTGACCAAGCAATGTAAATTAGTTAGAGCTTGCATTCTCGATCATCAAAGTATTTTGACTTTGACTGAGTGTGTACATAAAGTTTATCTGA

>Glyma13g32790 CTGAAATACAATTATTATGACAATTCTCAAAAAGTACCAACCAACCAAGGAGAGGTCGTGGAATTCCTGCGTGCCACGTAGCCAAGGATGTCCGACGACACCGTTTGGGTTATCGATCTAGAACCTTCTTTCTCTGACTTTCTCTCTCTGAAGAACTAGAATAATCTGCCACTATCCTTAAAAACCAACACGGTTTTCTTTTTGGTTTGGCAACATAGCACCAAAACCCTAAACCCTTCTTCTCTGATATTTAAAAACCTATCTTGTCACCACATTCACCTTTCTCCATTTCCTTTCTGATCAATCAATCTCCTCTTCTCTTCCGGCCATGGCCTCCTTGCTCCGCTCTCTCCGCCGCCGTGATGTCGCTTCCGCCTCCCTCTCCGCCTATCGCTCGGTACCATGTCCACCTCTTCTATGCTTCCTTCTTTTTCTTTATTTTGAATATACTTGTGCTGGTTACTCCATGTTACCTAGGTTTTCTCGATTTGATCCTATTATGCCTTAATCCAGTTATGTCTTGCTTTTGTTCGGATTTTGGCTCGAGTTGTTTAGAATTTTAGTCTTCTAATTTTCGTAATTGATTTTCTATTTCTAGTGTTTTAGAGTGGGGTTTTAATACCAAAAGCGTTAAGCCGTTGCTGTAGTAGCTCAAGATTTTGCTAGATCATTGTGAAGTCACTTAGTAGCTACTCTACGATATTGCTTTTATTACTTCTAATATAAGTTTTTCATGGGTCACTTTTCTAGCTTCTATAATTGCTTTATCGTGCATCTAACCTTCCTATGCTCTTTAATCCTGGGGACTTAACGAAGTACGTGAATAATTGTTGATATTATGCCGTTGTCCATAGAATTTATATAATTAATTGATTTGTGTTGTCTATGATGTGTGGTAACTTTAATTTTGGAGGACTTTTTTTTTTTTTTTACTTTTGCATTGTTGTGCAGTTAACGGGCAGCACCAAGCCAGCATATGTAGCTCACAACTGGTCTAGTTTGTCTCGACCATTCAGGTGCCATTTGTTTGTTATGTGCTTTTGTAATAATCCTCATAAGGTGTTGATCAAAATTACTAACTATGACTTTTCATCTTCACTGCAGTTCAAGGCCTGCTGGAAACGATGTCATTGGTATTGATTTGGGTACTACCAATTCATGTGTTTCCGTTATGGAAGGAAAGGTAACAGTAACACATTAACTCATTAATGCTTTTGGTTTGTTTGTATGTGTTTCCATTGTGCAATTTTTGTGCTGACTCAAAATATGTTTCTCAGAACCCCAAAGTCATTGAGAATTCTGAAGGTGCACGAACAACACCATCTGTGGTTGCTTTCAACCAGAAAGGGGAGCTGCTTGTAGGTACCCCAGCTAAGCGTCAAGCTGTAACTAACCCAACAAACACTCTCTTTGGTACCAAGCGGTTGATTGGTAGGCGCTTTGATGACGCTCAAACACAAAAGGAGATGAAAATGGTTCCATTCAAGATTGTTAAGGCTCCAAATGGAGATGCGTGGGTGGAAGCCAATGGGCAGCAGTATTCCCCTAGTCAAATTGGTGCCTTTGTTCTCACCAAGATGAAGGAAACTGCAGAAGCTTATCTAGGAAAGTCAATTTCTAAGGCTGTAATTACTGTACCAGCTTACTTCAATGATGCTCAGAGGCAGGCAACAAAAGATGCTGGTAGAATTGCAGGTCTTGACGTGCAGAGAATTATCAATGAGCCCACTGCTGCTGCACTTTCATATGGGATGAACAACAAGGAGGGTCTCATTGCCGTTTTTGACCTTGGAGGTGGAACATTTGATGTGTCCATCTTAGAAATTTCTAATGGTGTTTTTGAGGTATGCTGATTTGTTTCTTCTGTCTTCCCTTATTAACCTGTCAGATGGAATAATATGATAAAAATTGTACTGACAACATCTTCATATCAAGGTGAAAGCAACAAATGGTGACACTTTCTTGGGAGGAGAGGATTTTGACAATGCCTTATTGGATTTTCTGGTGAATGAATTCAAAAGAACCGAGAGTATCGACCTTTCAAAGGACAGGCTTGCACTGCAGAGGCTTCGTGAAGCTGCTGAGAAAGCTAAGATCGAGCTGTCTTCAACATCTCAAACTGAGATCAACCTGCCTTTCATCACTGCTGATGCATCTGGTGCTAAGCATCTGAACATAACATTGACTAGATCGAAGTTTGAGGCTTTGGTGAATCACTTGATTGAAAGGACCAAGGTACCATGTAAAAGCTGCTTGAAGGATGCTAACATCTCTATCAAGGATGTTGATGAGGTTCTTCTAGTTGGAGGGATGACTCGTGTTCCTAAAGTCCAAGAGGTGGTTTCAGAGATCTTTGGAAAGTCTCCTAGTAAAGGAGTAAACCCTGATGAGGCAGTTGCCATGGGGGCGGCAATCCAAGGTGGTATTCTACGGGGAGATGTTAAAGAGCTACTACTCCTAGATGTAACACCACTCTCTCTGGGTATTGAGACTTTGGGTGGTATCTTTACAAGATTGATCAACCGCAACACTACTATTCCTACAAAGAAGAGTCAGGTAAGCCTTAACTCTACAGACTTTGCTTCAGACATTTGTTTCTAATTTAACATTGAAATCATGCATTTTATATTTAATTGAAATTTCCCTTGAAAGTATTGTATCTGCTGCAATACATAGATCCTATTTGATTTAGTGGAATGTATTATTGCTATGTGGTTGAATCTTTTTCCTAACTTGTATCATTATTTCAGGTCTTTTCAACAGCAGCTGACAATCAAACTCAGGTAGGTATCAAGGTGCTACAAGGTGAGCGGGAAATGGCTGCAGACAACAAAATGCTTGGAGAGTTTGACCTTGTTGGTATTCCTCCTGCTCCCAGAGGTCTGCCTCAGATTGAGGTCACATTTGACATTGATGCTAATGGGATTGTTACTGTCTCTGCCAAAGACAAGTCCACTGGTAAAGAACAACAAATAACTATTCGGTCATCCGGTGGACTCTCGGATGATGAGATTGAAAAGATGGTCAAAGAAGCAGAATTGCATGCTCAGAAAGACCAAGAGAGAAAGGCTCTCATTGACATTAGAAACAGTGCTGACACTACCATCTATAGCATTGAGAAGAGTTTAGGTGAATACAGAGAGAAGATTCCCAGTGAAGTGGCCAAAGAAATTGAGGATGCAGTTTCGGATTTGAGACAGGCGATGTCAGGGGATAATGTTGATGAAATCAAGTCAAAGCTTGATGCTGCAAACAAAGCTGTGTCCAAGATTGGAGAGCACATGTCAGGTGGTTCTAGTGGCGGTTCCTCAGCTGGTGGTTCTCAGGGTGGGGACCAGGCTCCTGAGGCAGAATATGAGGAGGTGAAGAAGTAAGTCGGGGAGGAGTTTGTATCCACTTTTTACTGATTTATTTGTCTAGATCACAAGTTAGAGATGAATCATCTTTTTTCATTTAGCATATAATATAAGGTTTTTTGTACTAAAAGATGGAGTTCATTTTTTACCTTAAGGCCTTTTAAACTTGTAGTACTTCTGGAGAACCAAATTATGCCTGTTTGAATAACGATATTCAAAATTTTGCCTAGTGACTAGTGACACATTTTTTACTCTTATTCGTTATCATGATTGGCATTTACTCGTATTTTACACATCAATTGAATTTATTTATTGCGTTAGT

>Glyma13g43630 GCGGCTCGTAGCCAAAGCAAACTCAAGACTCTAGCCCCTTTATTAAGGACCGTCTCGTCTTCTCGATAGTCCGTTAATATTCCGCTGCGGTTTTTGAATCTTTCAAGATTGTTCCAGAACCCTTCAAAACCCTCTCTTCTCTCTTTCTTCGATCGCTTCGCCTGGTAAACCCTATTTCTTCTTCGCCTTAGATTTATGTTGCTTTTGCTTCCTCTCATCATAGATCTGCCTGTTCTCGGTGCTATATTCATGCATTTTGCTCCACAATTAAGAAATTATTTTACCTGCTTGTCATTATTAGGAAGCCTTTTCTTTTATATTCAGATTTTTAGTTTTATTTTGTACTTTACCATACATGTTCTTGTATTTAGCCATGTTAAAATACAATTTCGATGAAAAAGGATTGGATGATTTGATCTGATGGACTTTAACTATATAGTGCTTCTTTTTAGATCGAAAGAAAAATATTTTGATTTAGTTTTGAAGGGCTGCTTGTGCTTTTAGTGCTGAATGTTATGCCATTACAGTTTAATTTATTTGAATAACTGTTTTATTTGATTGCTGCATGCTTTTATAGCTCAATAATACATGAGCTGGTACCCTAGGTATTGCCTAGATGTCATTTCTACTTGTCTTCGTTGTTGTTCTTTCTACTGAAGTCATTTTCTATTCAACTGCTAGTAAATTAGAATCGATGCATCAGATGTAAACATGACATGATTTTTGTACTTTCGCTTGTTAGATATTCTTTTTGGCTGAGATCAAGCAAAAATGAGCGTGGTTGGATTTGATTTTGGTAATGAGAGCTGCATTGTTGCTGTCGCAAGACAAAGGGGGATTGACGTTGTGCTCAACGATGAGTCAAAGCGTGAAACACCTGCAATTGTATGCTTTGGTGACAAACAGCGGTTCCTTGGGACAGCCGGGGCTGCTTCTACTATGATGAACCCAAAGAATTCCATCTCACAGATAAAGAGGTTAATTGGCAGACAATTTGCGGATCCGGAATTGCAGCAAGATATCAAGACGTTCCCCTTTGTGGTCACTGAAGGACCTGATGGATATCCTTTAATTCATGCACGGTACTTGGGTGAATCTAGAACATTTACACCTACCCAAGTATTTGGAATGATGTTATCAAACCTTAAAGAAATAGCTGAGAAAAATTTGAATGCTGCTGTTGTTGATTGCTGCATTGGAATTCCACTTTATTTTACCGATCTTCAAAGGAGGGCAGTCTTGGATGCTGCCACTATTGCAGGTTTGCACCCACTTCGTCTGTTTCATGAAACAACAGCAACTGCATTGGCTTATGGAATTTATAAGACTGATCTTCCTGAAAATGATCAGCTGAATGTTGCATTTGTTGATGTTGGACATGCTAGCATGCAAGTATGCATTGCTGGATTTAAAAAGGGGCAGCTGAAAGTGTTGTCCCAATCATATGATAGGTCCCTAGGTGGTAGGGACTTTGACGAGGTTCTGTTCAACCATTTTGCTGCAAAGTTTAAGGAGGAGTACAAGATTGATGTATTCCAAAATGCCAGGGCTTGTCTGAGGTTGAGGGCTGCCTGCGAGAAGCTGAAGAAGGTGCTTAGTGCTAATCCGGAGGCCCCTTTGAACATTGAGTGCTTAATGGATGAGAAGGATGTCAGAGGCTTTATCAAGCGTGATGAGTTTGAGCAACTAAGTCTTCCAATTTTGGAACGGGTGAAGGGACCTTTGGAGAAGGCACTTGCAGAGGCAGGTCTTACAGTCGAAAATGTGCACATGGTTGAGGTGGTTGGTTCTGGATCTCGTGTACCAGCTATAAACAAAATATTGACAGAGTTCTTCAAAAAGGAGCCTAGGCGGACAATGAATGCTAGTGAGTGTGTTGCCAGGGGTTGTGCATTGCAGTGTGCAATTCTCAGTCCAACTTTTAAAGTACGGGAGTTTCAGGTAAATTTTCCCCCGGCTGTATTGCTCTCTAGTTTGAAATGCCTTCTGCAAAGTTTTATAATTAAAGGGATCATGGACTTCAGCAGTTGTTTTGTTATTTTTTGGGTATATGTTAATTTACCTTGAAGGTTGAGTTACTTGTCTCTGGTATCTCCTTTTGCTTTAGATTATTTAGGGATTTTGAATTTCAATGTAAACTATGCTTGTTAATTACTATTTTGTTGTGGTGAGTTGTTTGTACGTGTGTTAGTATTATATTTTATATAATGTTGCTCTTTTGTTTCATGATATGAAGGTCAACGAAAGCTTTCCTTTCTCAATTTCTCTCTCGTGGAAAGCTCCAAGTTCTGATGCACAGGAAAGTGGACCAGATAATAAACAAAGTACCCTTGTTTTCCCCAAGGGAAATCCCATACCAAGTGTCAAGGCACTGACCATCTACAGGTCAGGAACCTTTTCTATTGATGTTCAGTATGATGACGTGAGTGGTTTGCAAACACCTGCAAAGATCAGCACATATACTGTAAGGCAGTTTTTAAGTTTTTATTTACATACACAACACATTCTTTTAGCAGTCATTTGACTCTCAAGTTTACTTAAATGTATGCAGATTGGCCCTTTCCAATCTACAAAAAATGAAAAAGCAAAAGTTAAAGTGAAAGTTCGGTTGAATGTGCATGGAATTATATCTGTCGAATCTGCAACTGTAAGTCTCTCTAAGCTTGTTGATTGCTTTTATTATTTCTCTGCTATACTTTTGAGGTCATTATGGAAGTAAACCATAGAGCCTCTGTTTCTAATTTCTTATTTTTTCCTCCTTAAGCTACTGGAAGAGGAAGAAGAAATTGAGGTTCCAGTTTACAAAGAACCTGCAGGAGAAAATTCCAAGATGGAAACTGATGAAGCTCCTGCTGATGCTGCTGCTGCTGCTGCAACTCCTAGCACCAATGACAATGATGTTAGCATGCAAGATGCTAATACCAAGGCAACTGCTAATGCCCCTGGAGCCGAAAATGGTACCCCTGAGGCAGGAGATAAGCCTGTGCAGATGGATACTGATACCAAGGTAAATAGTGAAAGGAAAATTTAAGCATATGATTGGAATTTTTTTTCATGGGTCAATTTTTAGATGTGATTGTTCTATTTCTGTTGATAGTTTGTGATTTGACTGGCTTTACTTATATAACAGGTTGAGGCTCCAAAGAAGAAAGTTAAGAAAATAAACATTCCTGTGGTAGAGTTAGTTTATGGAGCAATGGCAGCCACAGATGTCCAGAAAGCTGTAGAGAAGGAGTTTGAAATGGCTTTGCAAGATAGAGTCATGGAGGAAACGAAAGATAAGAAAAATGCAGTTGAGGCTTATGTTTATGACATGAGAAACAAGGTGAGGATTGCCATGGTTTTCTTGTTATTTGAGGTCAATTTTTTTATTTTTAATTATTACTAAAACTAATTTCTTGATATCATTTTAGCTTAATGACAAATACCAAGAGTTTGTCATTGATTCAGAGAGGGAAGCATTTACTGCTAAACTTCAGGAGGTGGAAGACTGGCTATATGAGGATGGTGAAGATGAAACTAAAGGCGTATACATTGCCAAGCTAGAAGAGCTCAAGAAGGTAAGAAATTTTAGGGATTAGAAAATCTCTGACCCGAGCTGATCTGGTATGCTAAGGCCTGGTTGTTTTAGAGAATACTTTTATCCCCACCCGTGTAAAAATAGAGACTAGAGAGACGCCTTCAGTTATTTTTTGTAGCATTTTGCATAAACGCCTGCGATAAGGATATATTTTTGTTTTAGTTTTTGCCATCTTAATGGATATTTACATGATTGCAGCAAGGTGATCCAATTGAAGAGCGTTACAAGGAATACATGGAGAGGGGTACAGTAATTGATCAACTTGCCTATTGTATAAATAGTTATAGAGAAGCTGCAATGTCAAATGATCCCAAGTTTGATCACATTGACATCAATGAGAAACAAAAGGTAATTGGTTACTGCTCTGATTATGTATCACTTTTTTTTCCTGGTTGCCATTTAGCTGAGATTATTTTCCGTTTTATTTGTTATCAGGTCTTAAATGAATGTGTGGAAGCTGAGAACTGGCTCAGGGAGAAAAAACAGCATCAGGACTCGCTTCCAAAATATGCCACCCCTGTACTTTTGTCAGCTGACGTAAGAAAGAAAGCTGAAGCTGTGGACAGGTATTGCTTTTGTTCTTTGGATTTTAGGTCGCACTTTCTGTAGCTCATGTTTCTTAACCTTGCTTGCTCATTGTTCCATGATAGGTTTTGTAAGCCAATTATGACGAAACCAAAGCCACTGCCACCCAAGCCAGCTACACCAGAAGCACCAGCAACCCCACCTCCTCAGGGTGGTGAGCAGCAGCAACAACCACCTCAGGAGAATCCTAATGCCAGTACTAATGAAAATGCTGGGGACAATGCTAATCCAGCCCCGCCACCAGCATCTGCTGAACCAATGGAGACTGACAAACCAGAGAACACAGGCTCTGCTTAATTTTTGTTAATTTTGTCTGGTCTCTACATTTTGCATAACTATTTGAATTGTGCTTGGAAAAATAGTTGTGGGTGAGAGTTGATGCTGAAATTCTGCTGGAAAAATTGGTAATGGGTGAATTATGGTCCCTGAGATTCGGATTATTTTTAGTCAAATCTACGTTCTCTAGGTTTTTAGATTATTATATGTGTACCATTTTCTATAAGTGGAATTACGATGCTTGTTTTCATTTGAGTTAAATGACCTGATTTTCCTTTTGCGTGGCATTATAACTTTCCATTTTCTTCAATCTCATGGTGTTGTTGTTTTGTGAATTGTCTTCCCTGATGGGGTTTGGAGTTAAGCCACTATGCCCTCAGACTTGGACGGCAGATTCAAAAAGCA

>Glyma14g02740 ACTCGTGTAAACTGATTATTGTCAGATCTATTGGTTGATGATAGCTCAAGTTTTCCATTACAAATGTTTTGTACGAGGGATAGAGATGGTATTATGGCTTGAGCGAGTGCGGTGGCGATTTTGTCTCTCTGCCATTACAACTGAATTATGTAGTCACCAACTGCTTCTCCATTTCACCATTCATCTTTCTCTCCAAACCCGCTTCACCAAAATCAGGTTTCTTCCATTTTCTCTCACTATTCCTTTTGGCTTATCTCATTTTTTTCTTCTCTCTCGAAGTTTCTTTTTACATTCAAAGCATTTCTTCAGCTTCATAACTTTCTTGCTTTATTCAAAGCATTTATTGTCACTATATCATTGTTTTCTATACCTTTCCATGATGCAGAAATAAAGTTTTCATTTTGATGTGTATGTTTCCAACCTTTTCTTTTTGTAGTAATAGCGCCGCGTGAAATGTGAAGCTTATGTTTGTGGCCCGGCTTTGTGTGAAGTTTTTGACTTTTGGGTTGTGTTGGTGAAGTGGAAAGGGTGAATTGGATTGAATTAGTTGTTTGTAGTTTGAGGGAAGGACGCAGTAAATTAAAATGAGTGGAGTGGGGATTGATATTGGAAATGAGAACTGTGTGATTGCTGCAGTGAAGCAACGTGTGATTGATGTTTTGTTGAATGATGAATCCAAACGTGAAACCCCTGGTGTGGTCTGCTTTGGAGAGAAGCAACGGTTTATAGGGTCAGCTGGTGCTGTTTCTGCTATGATGCACCCCAAGTCCACAATATCTCAAGTGAAGAGACTAATAGGCAGGAGATTTACGGACCCTGATGTTCAGAATGATTTAAAACTGCTCCCAGTTGAAACTTCGGAAGGCCCGGATGGTGGCATTCTGATTCGCTTGAAATACTTGAAGGAGATTCATGCATTTACCCCGGTTCAAATAGTAGCAATGCTCTTTGCTCACTTGAAGACTATAGCTGAAAAGGATTTTGGGACCGCAGTTTCTGACTGCGTTATTGGGGTTCCGTCATACTTTACCAACTTGCAGAGACAAGCATATCTCGATGCAGCAGCAATTGTTGGGTTGAAGCCTTTGCGGTTGATCCATGATTGCACTGCAACTGGTCTTAGTTATGGAGTTTACAAAACAGATATTCCCAATGCAGCTCATATTTATGTTGCATTTGTTGACATAGGTCATTGTGATACTCAGGTCTCTATTGCAGCATTTCAGGCTGGTCAAATGAAGATACTTTCACATGCTTTTGACAGTAGCTTAGGGGGGAGAGACTTTGATGAGGTTCTGTTTAGTCATTTTGCAGCAAGATTTAAGGAACAGTATAGCATTGACGTGTATTCTAATGGCAGGGCATGTAGGAGGCTGCGTGTAGCATGTGAGAAGTTGAAGAAGGTTTTGAGTGCAAATGCAGTGGCTGATCTGAGCATTGAGTGTTTGATGGATGAAAAAGATGTTAAGGGCTTTATCAAGAGGGAAGAATTTGAGAATTTGGCATCAGGATTACTGGAGAAATTTAATATTCCCTGCAACAAAGCATTGGCTGATGCCGGCATGACTGTAGAAAAGATTAATTCTGTTGAGTTAGTTGGTTCAGGTTCACGGATTCCAGCTATAACTAATTTATTAACTTCTCTATTTAAGAGAGAACTCAGCCGCACGCTGAATGCAAGTGAGTGTGTAGCACGTGGTTGTGCTCTCCAGTGTGCAATGCTCAGTCCTATTTTCCGTGTCAAAGAATATGAGGTTTGAATATTGAAACAAAACATTGATTCTTATCCTATCTATATGATATGTATATGTAGGAGAGTAGATTTTTGTGTTTGTAGATTCTTAGGTTAGAAATGTAATAATTACTAAATGAACCAAGTTGTTACCTAGGTGCTAGGTTTATTTTCTGCATTTAGTTTGTCTATCTTAATTGAAATATATCTAACTTCATAGATATCATTGTTTTTTTTTTTAGAATTTTGCATTAGCATGTATTGCTCTCTATAAAATTAATGTAATTTCTTGTGTTTGTTCAGGTCCAGGATTCTATTCCTTTTTCCATTGGACTTTCATGTGATGGAAGTCCAATTTGTGAAGGATCGGATGGTGTACTCTTCCCAAAAGGCCAACCCATTCCAAGTGTTAAAATTCTAACATTTCAGTGCAGTAATTTGCTCCATCTGGAAGCATTCTATGCTAATCCAGATGAATTACCACCTGGGACATCTCCTAAAATAAGTTGCTTCACAGTATGCCTGCTTTAGAACTTTACTATGATGTGAAAGAGTTTAGATCATCTTGTCCGTGATTCTTTGTTATTAATTTTTTTAATTAATTATTACCCTCTCTGTTCTTGCTTCTTTTGGGTGCTTCATGAAAGGCATGTGGTATAGTCTCTATGAGCTTATTCATCTTCTTCATTTTACTGTTTTGGTATTTTTAATTCTACAGTAAAAAAATAGTATATACTATAGGGTAATCGTCATCATAATTGCAGCTTTTACTTTTGAATTGCAAGTCTAGGACTCAGTTATGTATAAAGCTACTACTTGTTATACATGTGCTTGTATTTTATCATAGTTTCAACCGTTGATTTTTTATTCAATGGTTATAAATGCACTTTATTCTTTAGAATGTATTCCCTCACTTTAGAGGAGTCAAATCCAAGACCTGCAATGTTGTTTGAAGTTCACTGTTGAGTGGTAAAACCTCAACAAGGAATAAAGCTCAAAATTGCCAACTCAAGAATGTTATGATGGATAAGTAGACACACAAGGTAGAATTAGGAATGATTGCGTTAGAAAGTTAGGCACCATCTGTTGTAGAAAATATGGTAGAATCATGCCTTAAGTGGTTTAGACACGTGTGAAGACTTTGTAGAATCTCTAAAGAAAGAGAGTGAATCAGATGGAAGATATAATTAGAGGTAGTAAGAGATGAAGAAAAACTATAAGTTAAATCATTAAGAAAGATTAAGATTTAAATGGTTTGTTTATAGAGATTATTTATGATAAGACATTATGGTGGTGTTTGATCCATGTAGGGCTACTGTTGTTGAATCTTGGGACAATGCAATAGACACCTCCCCCTTTCTTGAAGGAGAGAAAGACTTCATTATTAAATGGCCTTGCTGACCATCATTTATTCTGATCTCAAAGGAAGTCATTGTTGTAAAAGTATTAGGTATAGCAATATGAGAATCTGTAGGTGTGCACATGCTTGAACAAATCATAATGTTCTTAGGAGAAGGGGAAGCAGATATGGTGGATCTAACACCATTCATAAATCTACTGTCTAAGTTTCTCAAATGGAGAGAAATGACTATATTTAATCTTTAGTATCTTCAATGACAATTGGCTAACATTTGCTAATGTCTATTATATAATATAAGACATCTCAATATTGAGATACTGCCATTCCTTGTGCCAGTGATTAAAAAGTGTTATGTATGCTTATGCCTACACCTTGTTTTTGGATGCTTATGCATTTGTTTGTCATTTCTATTTAGATTGATCCTTTCCATGGATCCCATGGAAGTAAGGCAAGAATTAAAGTTCGAGTTCAACTAAATTTGCATGGCATTATCAGTATTGAATCAGCTACAGTAAGAAACCTCAATTTTATCTCTTCATATTGCATTATTAGTTTTCTGAGAGATCTATCTATGCACATGGTCATTTTTGTCTTTCAGTTGATGGAGGATCATGTGGATGATTCAGTTACAACAGGTGATTATCATTCAAATTCTGAAGCAATGAATGTTGAACCTGTTTCTGAGACAGTTGAAAATGTCACAGAAGATAGTATCAACAAAAAGTGTGAAGCTCCACGCCATTTGGTAAGTACTCCCATGTTGTTCTGTCTCTCCCCCCTGCTCACCTTTTTTTACTGGAAGATATCTCAGATATTAGAGATTTCTAGCAAATTTTCCTTTTAACACGAACAATTTTATGGCTAAATTTTATTATCTGATTATTTCTGATACTGAATCTGTACCATGTCTTTGACTGTCAGTCTGTTGCAACTGTTTTTTATAATGTCTGTGATGTTGTATATGTACATTTTTATCTTGTGGAGATTGTTTTGATTGCAGATATGATTCATGTTGGTGTTTTGAGAAAATAAAAGTGTTACTATCATGCCTTGTTACAGGCTGATGGTACAAAAAAAGATAAAGCTAACAGAAGGCTTCATGTACCTGTGAGTGAAAACATTTATGGTGGAATGACCAAGGCTGAGATATTGGAAGCTCAAGAAAAAGAACTCCAGTTAGCTGATCAGGACAGAACAATTGAGCTGACCAAAGACAGGAAGAATTCGTTGGAGTCTTATATTTACGAAACGAGGAGTAAGGTACATGTTCAATATCTTAATGAGATGTAGACATCACTGGCTTCTCTGATTCAATTAAATTGTATAAGCGGTCACACATCTCATCTGCTAAAATAGATAATTGTTCATTGCGTGATCCTTTTTTTCTTCTTTTTCATATCTTTACTGTGAGGTGTTCCTTCTTCAAAAACTAGAGCCTTAAATTTTGCCATGTAAATATGAAAGTATTGAATATATCAAAATGAGAAAAAGCTGTTTTATAGTCATTGAAGAATTGATTTTTAATTTTAATGACTCCTGTCATGACTCAAGTAAATGTAAGGGTAAAACTTTGTGAAGCTGAGGATGGTCCGCTGTTATACTTTGTAATAAAATCTGCATAGGATATAAAATTAAAAGCTGAATCCTTATGCCTTTTCTTACCATTGATTTTATTAAAGCTGTCTTCATGTCTATGCTCTACTTTTCATTCTGTACTGGTATTTAAATTTATTTGCTTGTATGAATAGCTATTCAGCACATATCTAAGCTTTTCAAGCGAACATGAGAGGAAGGACATATCTAGGAGCCTGAAAGCGACTGAGGATTGGCTTTATGATGATGGTGATGACGAAACTGTAGATGCTTATTCTGCAAAACTAGAAGATCTAAAACAGGTTACTTTTGTTTCTCCAATCTCCATGTTTCATACGCCATACTTCTTGCTTACAATCTCCATCTTCTACAGCTGGTGGATCCAATTGAGTTTCGGTATAAAGACACAGAAGCAAGACCACAAGCTACAAGAGATTTGTTAAGTTGCATTGTAGAGTATCGAATGTCTGCAGATTCTCTTCCACCCCAAGATAAAGAACAGGTACAACAATAAACAAGTGAATAAATATAATTGTGGGATGTTTCCCTTAGTATCAGTTTTTAAGTGACAACTTTCTTTTTCAGATCATCAATGAGTGCAATAAAGCAGAGCAGTGGCTAAGAGAGATGAGGCAACAGCAAGATCTTTACCCTAAGAACTTTGATCCAGTATTATTGTCAAGTGATATCAAGAGCAAGACAGAAGATTTAAACTCGTATGATTTTAACTTTCTGTTTTCCTTTTCAGAGATGCATAACTGTAGGGTACTATGATCATTTATTTGGGTCAGAATTATACCCCTTTCTGAAGCAAAGCATTGTTCATTTGCTCCAATTTAACTTGATTGTCATTATATACATTGATTTTATCTCAAAACTAATGAATGTTCTATTTTGATGGGTTTTCAGAGTATGCCAACAGATATTGAAATCCAAGGGTTCTCCATTTCCAAAAGACAAGGGCGAAGACAAGCAGAATACTTCTAATCATCAATGAAGACGATGAATGAACTGAAGGCCTAAATTTTTAATAGCTTCTGACAAATGTGAACTTTTTTTTTAACACTTTTCCCCCTTCCGGGGAGTTGAAAGAGATAAATTCAAATAAGGGAAATCATTGGTAATTACCGGTTCAATCTGCTTTTTTTATTTTATTTTTGGCCCCACGTTCTACTAAAGCCATCCAATTAGTTAGTACGTAGACAAGCCAATCAAGTAGTTTATTGTTTGTCAAGGGTTTCTAGATTTTATGCTCGTCAACAAGTTTTCAATTTAATTCCAAATTGTATTCAATCTATCATTTTGGATGGAATAAAATCGTGAAGATTGGACGCTTCTGCAAAACTGACCACCTTTATGGCTTTATCTTTATTCGTT

>Glyma15g01750 CGCAAACAAGCCCAACTTTCCAGTTTGAGAAACCTAAAAAAAAAAAAAAAACAGAGAGCAAGGGTTCCGAGTATAAGCGGTAGATGTTTCTAGAAGTTGGGAGTATCTTCCGGACATCTCTGGAGCAATAGAGCACGTCTTTTGTTTTCCGTTCAAACTGGTTAATTTAACAATTGAAAAGGTCACAAACACAAATAGAAAAAATAAAGTAAACACAAACCCAAAATTCCACAGCGGCCCCCAGCCAAAGCAAACTCAAGACTCTAGCCTCTTTATTAAGGACCGTCTCGTCTTCTCGATAGTCCGTTAGTATGCTCTGCGGTTGTTGAATCTTTTTTAGATTATTCCTGAACCCTTCAAAACCCTCTTATCTCTCTTCTATCGCTTGGCCTGGTAAACCCTATTTCTTCTTCCCTTAGATTTTTGTCCCTTTTGCTTCCTCTCATCATAGATCTGCGTGTTCTCCGTGCATTTTGCTCTACAATTAAGAGTTTTTTTTACTTTCTTGTCATTATTGGAAAGCCTTTTGTTTTGTATTCAGATTTTTTAGCTTTATTTTGTGCCTACCATTTATGTTGTATTTAGCCTAGTTAAAATACAATTTCGATGAAAAGGATTGGATGATTTGATCCGATGGACTATAACTTTATAGTGCTTCTTTTTAGATCGAGAGAAAATATTCAGATTTAGTTTTCAAGGCTGCTTGTGCTTTTAATGCTGAGTGTTATGCCATTACGTTTTAATTTATTTGAATAACTGTTTTATTTGATTGCTGCATGCTTTTATAGCTCAATTATAATGCATGAGCTGGTACCCTAGGCATTGCCTAGATGTCGTTTCTACAGTCATTTTCTATTCCACTGCTACCAAATTAGAATCGATGCATCATATGTAAATGTGACATGTGATTTTTTTTATTTTCATTTTAGATATTCTTTTTGGCTGAGAGATCAAGCAAAAATGAGCGTGGTTGGATTTGATTTTGGTAATGAGAGCTGCATTGTTGCTGTCGCAAGACAAAGGGGGATCGACGTTGTGCTCAATGATGAGTCAAAGCGTGAAACACCTGCAATTGTATGCTTTGGTGACAAACAGCGGTTCCTTGGGACAGCTGGGGCTGCTTCTACAATGATGAACCCTAAGAATTCCATCTCTCAGATAAAGAGGTTAATTGGTAGACAGTTTTCTGATCCAGAATTGCAGCGAGACCTGAAGACATTCCCTTTTGTTGTCACTGAAGGACCTGATGGATATCCATTAATTCATGCACGGTACTTGGGTGAAGCTAGAACATTTACACCCACCCAAGTATTTGGAATGATGTTATCAAACCTTAAAGAAATAGCCGAGAAAAATCTGAATGCTGCCGTTGTTGATTGCTGCATTGGAATTCCACTTTATTTTACTGATCTTCAGAGGAGGGCGGTCTTGGATGCTGCTACTATTGCTGGTTTGCATCCACTTCGTCTGTTTCATGAAACAACTGCAACTGCATTGGCTTATGGAATTTATAAGACCGATCTTCCTGAAAATGATCAACTGAATGTTGCCTTTGTTGATGTTGGACATGCTAGCATGCAAGTATGCATTGCTGGATTTAAAAAGGGGCAGCTGAAAGTGTTGTCCCAATCATATGATAGGTCCCTAGGTGGGAGGGACTTTGACGAGGTTCTGTTCAATCATTTTGCTGCAAAGTTTAAGGAGGAGTACAAGATTGATGTATTCCAAAATGCCAGGGCTTGTCTGAGGTTGAGGGCTGCTTGCGAGAAGCTGAAGAAGGTGCTTAGTGCTAATCCCGAGGCACCTTTGAACATTGAGTGCTTAATGGATGAGAAGGATGTCAGAGGCTTTATCAAGCGCGATGAGTTTGAGCAACTGAGTCTTCCAATTTTGGAACGGGTGAAGGGACCTTTGGAGAAGGCACTTGCAGAGGCAGGTCTTACAGTTGAGAATGTACACATGGTTGAGGTGGTTGGTTCTGGATCTCGTGTGCCAGCTATAAACAAAATTTTGACAGAGTTCTTTAAAAAGGAGCCTAGGCGGACAATGAATGCTAGTGAGTGTGTTGCCAGGGGCTGTGCATTGCAGTGTGCAATTCTCAGCCCAACTTTTAAAGTACGGGAGTTTCAGGTAAAAATTTTCCCTGCCATTATAATGTTTTAAAATTATTCAATATGGAACTGTTCAGATCTTTTTACGAATCATGGACTTCAGCATTTTTTATTTTTTTTTGGTATATGTTATGTACCTTGAAGTTTGAATTACTTGTCTCTGGTATCTCCCTTTGCTTTAGAAATTGTGCTCGTTAACTACTATTTTGTTGTGGTGAATTGTTTGTATATGTGTTAGTATTATAATTTTATATAATCTTGCTCTTTCTGTTTCATGATGTGAAGGTCAATGAAAGCTTTCCTTTCTCAATTTCTCTCTCGTGGAAAGGTCCAAGTTCTGATGCACAGGAAAGTGGACCAAATAATACGCAGAGAACCCTTGTTTTCCCCAAGGGAAATCCCATACCAAGTGTCAAGGCACTAACCATCTACAGGTCAGGAACCTTTTCTATTGATGTTCAATATGATGATGTGAGTGAATTGCAAACACCTGCAAAGATCAGCACATATACTGTAAGTCAGTTTTTAAGTTTTTATTTACGCACACAGACAACACATTCTTTTGTCAGTCATTTGACTCCCAACTTTACTTAAATGTATGCAGATTGGACCTTTCCAATCTACAATAACTGAAAAAGCAAAAGTTAAAGTGAAAGTTCGGTTGAATCTTCATGGAATTGTATCTGTCGAATCTGCAACTGTAAGTCTTTAGTCTTGTCGATTGCTTTATATTTATCTGCTATACTTATCTTGTGAGCATCTGTTTCTAATTTCTTATCTCCCCCCCCCCCCCCCCAAAAAAAAAAAAAAAAAAGCTGCTGGAAGAGGAAGAAATTGAGGTTCCAGTTTCCAAAGAACCAGCAGGAGAAAATACCAAGATGGAAACTGATGAAGCTCCTGCCAATGTTGCTGCACCTCCTAGCACCAATGACAATGATGTTAACATGCAAGATGCTAATTCCAAGGCAACTGCTGATGCCCCTGGGTCCGAAAATGGGACCCCTGAGGCAGGAGATAAGCCTGTGCAAATGGATACTGATACCAAGGTAAATGGTGATAGGAAAATTTAAGCATATGAATGGAATGTTTGTGTGTCATTGGTCAACTTTTAGATGTGATTTTTCTATTTTTGGTATTTAGAGCTTGTTATGATATTGTGTGGTTTTCTTTTTATATAGTATGAGTAGTATAGACATCAATAAGATGCTATTGCTTGGCAATTGTTGATTGTGATTAGAGTTGGGGGGTTATACGATCTTGAATGAATTAAAACTAGTAGATTTTGATGACTAAGATGACAGTGTGACAACTTAAATTGTTTAAAACATTAGTCTGAATCTGAATACTTGTAAAGTAGGCTTGTTGTTTAGATTTATTGTGGTATTTCATTGTATTTGAGTTTTACAAATATGGATTTAATTGATTTTTTGCCCTTATTTGGAGGTATATGAGCTTGTTTGGTTTGTTTTCTGCTTTTCATTTACAATTGATGTTTTTTGCAAAAATACCGTCTTGTTTTCATGTTGTTCCTTTTCTCCAATTTTTCTCAAGGAAAATGGTAAAAAAAATAGTTTTTAAAACAAGAAAATAAACCAAGGGTGATCCAAGTCCTCCCCTTCCTATAAATATATTGTGAATATGATTTTGTGTGTAATATTATATGCTTGGTTGGATTTTGTGCATCAGTTAAATTGTCAAACATGGTCTGTTGATAGTTCGTAATTTGACTGGCTTTACTCATAACAGGTTGAGGCTCCAAAGAAAAAAGTTAAGAAAATAAACATTCCTGTGGTGGAGTTAGTTTATGGGGCAATGGCAGCTGCAGATGTCCAGAAAGCTGTAGAGAAGGAGTTTGAAATGGCTTTGCAAGATAGAGTGATGGAGGAAACAAAGGATAAGAAAAATGCAGTAGAGGCTTATGTTTATGACACGAGAAACAAGGTGAGGATTGCCATGGTTTTCTTGTTATTTAAGGATGAATTTTGTATTATTACTAAAACTAATTTCTCATTATCATTTCAGCTTAATGACAAATACCAAGAGTTTGTCGTTGATTCAGAGAGGGAATCATTTACTGCTAAACTTCAGGAGGTAGAAGACTGGCTATATGAGGATGGTGAAGATGAAACTAAAGGTGTATACATTGCCAAGCTAGAAGAACTCAAGAAGGTAAGAACTTTTAGGGGTTAAAAAAATCTCTGACTCTAGCTGATCTGGTATGGCCTTGTTTTAGAAAATACTTTTATCCCCATCATAGCAAAAATCTTTTCAAAGATGGAATGATTAAGGAGGCACCTTGTATTATTTTTGGTAGTATCATGCATAAATGCCAGCTAAAAGGAAATATTTTTGTTTAAGTTTTTGCCATCTTAATGGATATTTACATGATTGCAGCAAGGTGATCCAATTGAAGAGCGTTACAAAGAATACATGGAGAGGGGTACAGTAATAGATCAACTTGTCTATTGTATAAATAGTTATAGAGAAGCTGCAATGTCAAATGATCCCAAATTCGATCACATTGACATCAATGAGAAACAAAAGGTAATTGGTTGCTGCCGTTTCTGTAAGTCTTTTAATTCATGGTTGCCATTTAGCTGAAATTGTTTTCCATTTTGTTTGCAGGTCTTAAATGAATGTGTGGAAGCTGAGAACTGGCTCAGGGAGAAAAAACAGCAACAGGACTCTCTTCCGAAATATGTCACCCCGGTACTTTTGTCAGCTGACATAAGAAAGAAAGCTGAAGCTGTTGACAGGTATTTGCTTTTGTTCTTTAGATTTAGATCGCACTTTCTGTAGGTTGTGTTTTTTAACCTTGCTCATTGTTCCATCTATACAATGATAGGTTCTGTAAGCCAATTATGATGAAACCAAAGCCACCACCACCCAAGCCAGCTACACCAGAAGCACCAGCAACCCCACCTCCTCAGGGTGGTGAGCAGCCACAGCAGCAGCAACAACAACCACCTGAGGAGAATCCTAATGCCAGTACTAATGAAAAGGCAGGGGACAATGCTAATCCAGCCCCACCACCAGCATCTGCTGAACCAATGGAGACTGACAAACCAGAGAACACAGGCTCTGCTTAATTTTTGTTGATTTTGTCTGGTCTCTACGTTTTGTATAACAATTTGAATTGTACCTGGAAAAATCGTCGTGGGTGAGAGTTGATGCTGGGATTCTTTGCTGGAAAAATTGGTAATGGGTGAATTATCTGAGATTCGGATTATTTTTGGTCAAATCTACGTTCTATAAGTGGAATTACAATGCTTGTTTACATTTGAGTTAAATGACCCTATTTTCCTTTTGCGTGGCATTATAACTTTTTCATTTTGTTCAATCTCGTATTGTTGTTTTGTGAATTGTCTTCCATTTCATTCTGCGATTGGGTTTAGATAT

>Glyma15g06530 CATTACATTTTGCCTGAAATAGAATTATGACAATGACATTATCAAAAAGTAGCAACCAAGAAAGGAGAGGTCGTGGAACTCTCATGGACCACGAAGCCAAGGATGTCCGACGACACCGTTTGGGTTATCGATCTAGAACCTTCCTTCTCTGACTTCCTCTCTCTGAATAACTAGAATAATCTGCCACTATCCTTATAAACCGACACCGTTTTCTTTTGTTTTGGCAACACGGTACCAAAACCCTAAACCCTTCTTCTCTGATATTTAAAACCTCTCTCAACACCTTCACCTATCTCTATTTCCTTTCTGATCAATCAATCTCCTCTTCTCTTCCAGCCATGGCCTCCTTGCTCCGCTCTCTCCGCCGCCGCGATGTCGCCTCCGCCTCCCTCTCTGCTTATCGCTCGGTACCATCTCCACCTCTTCTATGCTTCCTTCATTTCTTTATTTTTTTTTATGGTTGCTCCATGTTACCTAGGTTTTCCCGATTTGACTCAATTATGGCTTAAATCCATTTATGTCTTGCTTTTGCCCGGATTTGGCTCCAGTTGTTTAGAATTTCAGTCATCTAATTTTCGTAATTGATTTTCCATTTCTAGTGTTTTTGTTGTTTAATTATACCAAAGGTGTTAAGCAGTTGATGTACTGGCTCAAGATTTTGCTAGATCATCCGTGTTTTTGTGGACTCACTTAGTAGCTACTCTACGATATTGCTTTTATTACTTCTAATGAAAGTTTCTCCTGGCTCACTTTTCTAGCTTACAATTCTATAATTGAATTATCGTGCACCTAACCTTCCTATGCTCTAGAATCCTGGGGACTTAACGAAGTACGTGAATAATTGTTGATTATGCCGTTGTCCTTAGAATTATATAATTAGTTGATTTGTGCTGTGAATGATGTGTGGCAACAATGATTTTTGGAGGAAGTTTTTTCTTACTTTTGCATTGCTGTGCAGTTAACGGGCAGCACCAAGCCAGCATATGTAGCTCACAACTGGTCTAGTTTGTCTCGACCATTCAGGTGCCATTTGTTTGTTATGATTTTGTAATAATCCTCAAAAGGCGCTGATCAAAATTATTCATGATGACTTTTCATCTTCATTGCAGTTCAAGGCCTGCTGGAAACGATGTCATTGGTATTGATTTGGGTACAACCAATTCATGTGTTTCCGTTATGGAAGGAAAGGTAACAGTAACACATTAAATCATTAATGCTTTTGGTTTGTTTGTATGTGTTTCCGTTGTGCTGACTCAAAATATATGTTTCTCAGAACCCCAAAGTTATTGAGAATTCTGAAGGTGCACGAACAACACCATCTGTGGTTGCTTTCAACCAGAAAGGGGAGCTGCTTGTAGGTACCCCAGCTAAGCGTCAAGCTGTAACTAACCCAACAAACACTCTCTTTGGTACCAAGCGGTTGATTGGTAGGCGCTTTGATGATGCTCAAACACAAAAGGAGATGAAAATGGTTCCATTCAAGATTGTTAAGGCTCCAAATGGAGATGCTTGGGTGGAAGCTAATGGGCAGCAGTATTCCCCTAGCCAAATTGGTGCCTTTGTTCTCACCAAGATGAAGGAAACTGCAGAAGCTTATCTAGGGAAGTCAATTTCTAAGGCTGTAATTACTGTACCAGCTTACTTCAACGATGCTCAGAGGCAGGCAACAAAAGATGCTGGTAGAATTGCAGGTCTTGACGTGCAGAGAATTATCAATGAGCCCACTGCTGCTGCACTTTCATATGGGATGAACAAGAAGGAGGGTCTCATTGCCGTTTTTGACCTTGGTGGTGGAACATTTGATGTGTCCATCTTAGAAATTTCTAATGGTGTTTTTGAGGTATGTTGATTTGTTTCTTCTTCTGTCTTCCCTTATTAACCTGTCAGATGGAATAGTATGATAAAAATTGTACTGACAAATGACAACATCTTTCATATCAAGGTGAAAGCAACAAATGGTGACACTTTCTTGGGAGGAGAGGATTTTGACAATGCCTTGTTGGATTTTCTGGTGAATGAATTCAAAAGAACTGAGAGTATTGACCTTGCAAAGGACAGGCTTGCACTGCAGAGGCTTCGTGAAGCTGCTGAGAAAGCTAAGATCGAGCTGTCTTCAACATCTCAAACTGAGATCAACCTGCCTTTCATCACTGCTGATGCATCTGGTGCAAAGCATCTGAACATAACATTGACAAGATCGAAGTTTGAGGCTTTGGTGAATCACTTGATTGAAAGGACCAAGGCACCATGTAAAAGCTGCTTGAAGGATGCTAACATCTCTATCAAGGATGTTGATGAGGTTCTTCTTGTTGGAGGGATGACTCGAGTTCCTAAAGTCCAAGAGGTGGTTTCAGAGATCTTTGGAAAGTCTCCTAGCAAAGGAGTAAACCCTGATGAGGCAGTTGCCATGGGGGCAGCAATCCAAGGTGGTATTCTACGGGGGGATGTTAAAGAGCTACTACTCCTAGATGTAACACCACTCTCTCTCGGTATTGAGACTTTGGGTGGTATCTTTACAAGATTGATCAACCGCAACACTACTATTCCTACTAAGAAGAGTCAGGTAAGCCTTAATTCTACAGAGTACAGATGATTGCTTGAGACATTTGTTTCTAATTTAATATTGAAATCATGCATTCTATATTTAATTGAAATTCCCCTTGAAAGTATTGTATCTGCTGCAATACATAGATCCTATTTGATTTAGTGGCATGTATTATTGCTATGTGGTTGAATCTTTTTCCTGACTTATATCATTATTTCAGGTCTTTTCAACGGCAGCTGACAATCAAACTCAGGTAGGTATCAAGGTGCTACAAGGCGAGCGGGAAATGGCTGCAGACAACAAAATGCTTGGAGAATTTGACCTTGTTGGTATTCCTCCTGCTCCCAGAGGTCTGCCTCAGATTGAGGTTACATTTGACATTGATGCCAATGGGATTGTTACTGTCTCTGCAAAAGACAAGTCCACTGGTAAAGAACAACAAATCACTATTCGGTCTTCCGGTGGACTCTCGGAAGATGAGATTGAAAAGATGGTCAAAGAAGCAGAGTTGCATGCTCAGAAAGACCAAGAGAGAAAGGCTCTCATTGACATTAGAAACAGTGCTGACACCACCATATATAGCATTGAGAAGAGTTTAGGTGAATACAGAGACAAGATTCCCAGTGAAGTGGCCAAAGAAATTGAGGATGCAGTTTCGGATCTGAGGAAGGCGATGTCAGAGGATAATGTTGATGAAATCAAGTCAAAGCTTGATGCTGCAAACAAAGCTGTATCCAAGATTGGAGAGCACATGTCAGGAGGTTCTAGTGGCGGTTCCTCAGCTGGTGGTTCTCAGGGTGGGGACCAGGCTCCCGAGGCAGAATACGAGGAGGTGAAGAAGTAAGGCGAGGAGGGGTTTGTATCCACTTTTACTGATTTATTTTGTCTAGAGTTAGAGATGAATCATCTTTTTTCATTTAGCATATGATATAAGGTTTTTTGTACTAAAAGGTTGGAGTTCATTTTTTACTTCATGTACTTCTGGAGAACCAAATTATGCTGTTTGAATAACGATATTCAAATTTTTGCCTAGTGACCGTTGACGCATTTTTTACTTATTATCATTGGCTTTTACTAGTATTTTGCACATCAATTGAATTTATT

>Glyma15g09420 ATGGCAACAAAAAAAGTCAAAGCTATAGGCATTGACTTGGGCACCAGCTACAGCTGTGTGGCTGTGTGGCAACACAACCGCATCGAGGTCATTTCCAACGACCAAGGCAACTGCACCACCCCTTCCTACGTTGCCTTCAACGACAACCAAAGGTTGTTGGGGGACTCCTCCATGAGCCAAAGATCCATGAATCCGCAAAATACCGTCTTTGACGACAAACAAACGTTTGATTGGTCAACGATTCTCCGACCAAACCGTACAGCAAGACATGAGCGAACATGAGAGTATTGATCACCAAGACCTTTCAAGAACTTGATTACCTTATCTTGCTCCTTGAATTTGCGTAAATCTGTAGCAGCACCACAAGAACACGGCATAGCGCAAGTACAATCACGAATTGGACGAAAATTCTCAATTTCTTCCCAAAGCGTCATCAATTTGGTGAAATACGAAGAGATGTCAAGAGTTCCTTGTTGAAGACGAGCTACTTCCTCTTGAATATCAGCAACGCGAAAAATATCACCCTCTTGAATATCAACAACATGAAAAATATCACCCTGAGAGAACCGATTCTCCAAACTTTTCCATACAAGAGAAGCGCGATCACACCAGAGGAGAGATTTTGCGATTTCTTCAGAGATTGAACGTTGAAGCCATGAGAGAACGAGGTTATTACAACGAAGCCAGGGTTCATAGAGAGGATCAGAGATAAGGGGAGGGGAAAGAGTGCCATCAACAAATTTGACCTTGTTCTTGGAGATAAGAGCCACCTTCATCGGTCTGCACCAGATCTGATAGTTTTTGTTATCAAGCACAGGTTGAACGAGAACAAGAGAAGGATTCTCATTCGGATGCATGTAGTATGGATTAGAGGGATTGGTAGCAAAATCAGAGTAAGAAATCACCATTGTTGAGGTGATTCAGCAAGAAAGAATTGTAGAACGCGAAGAAGATGAAGGTGCAAAGAAAAAAATTGCGGAAACTGTTTCAAATTGATACCATGTTAACTTTGGAATACCAAAGGAGAGAAATTATGAAATGGAATATATTCATTGTAAAAAAACATAAAACAATGCATAAAGTGTAATTAATATATATAGTAAACTAGCTAACTAACAAACTCTTAACTTGGCTAAATTAATTCAGTTACAACTATGATTAACTAAGATTAACTAGAGTTACTATTTACATCGGCCTTTTAAGGTTGTCCCTGACAATAGAGACAAGCCCATGGTCACGGTCACTTACAAAGGTGAGGAGAAACTCCTTGCCCCCGAAGAGATATCTTCCATGGTGCTGTTTAAGATGAAGGAAGTTGTCGAAGCCCATTTGGGTCATTTCGTAAAGGATGCAGTGATCACTGTCCCTGCTTACTTCAGCAACGCGCAGAGACAGGCCACTAAGGATGTCGGGAAAATCGCGGGTTTGAACGTGTTGAGGATCATCAGCGAACCAACTGCGGCTGCTATTGCTTACGGGTTGGACAGAAAAGGATTGAGAGTGGGTGAGCAGAACGTGCTTGTGTTTGATCTCGGTGGTGGTACTTTTGATGTTTCCTTGGTGACTATTTATGAAGGGATGTTTAAGGTTAAGGCTAGTGTGGGAGATACTCATTTGGGTGGTGTGGATTTTGATAACAAATTGGTGAACCATCTGGTGAATGTGTTTAGAGAGAAGCACAAGAAGGATATTAGCGGGAATGCGGAAGCTTTGGTGAGGTTGAGGTCAGCGTGCGAGAAAGCAAAGAGGATTCTGTCTTCGACTGCTCAGACAACGATTGAGCTTGATTGTTTATATGAAGGGGTTGATCTGTATGCCACTGTGACAAGGGCCTTGTTCGAGGAACTGAACAAGGACTTGTTTATGAAGTGTATGGAGACGGTGGAGAAGTGTCTCCTTGAGGCAAGGAGTGATAAGATTCAAGTCCATGAGATCGTTCTTGTTGGTGGGTCTACTAGAATTCCAAAGGTACAACAACTTCTGAAGGACATGTTCAGTCTCAATGGTACCACCAAAGAGCTTTGCAAAGGCATCAACCCTGATGAAGCTGTGGCGTATGGTGCAGCAGTTCAAGCAGCGATTTTGAGCGGTGAAGGAGATAAAAAAGTGGAGGAATTGTTGTTGCTGGATGTGATGCCAATTAGTATTGGATTCGAGGGTGCTGGTGGTGTGATGTCAGTGTTGATCCCCAAGAACACCGCGATCCCCACCAAGAAGGAGAGGGTTTGTTCCATCTTCTACGACAATCAGAAATCTCTTACAGTCAAAGTGTTCGAGGGAGAACAAGTTAAGACAAAGGATAACTTCTTTCTCGGCAAGTTTATCCTCTACAGGTTCGATCCATTACCAAAGGGAGTGTCACAAATCAGCGTTATCTTTGATGTGGATGCTGATGGCATTGTGGAGGTAACCGCGGAAGATCAAGCCAAAGGGTTAAAAAAGAAGATCACAATCAATAGCAAGCATGGAAGGCTAAGCCCGGAAGAGATAAGAAGAATGGTGAGAGATTCAAAGAGGTACAAGGCAGAGGATGAGGTGGCAAAGAAGAAGGTGAAGGCGAAGAACACACTCGAGAATTACGCTTATGAAATGAGGGAGAGAGCAAAGAAGATTGAAGAGGCAGTTGAGGAAACCATAGAGTGGCTAGAGTGTAACCAATTGGCAGAGATAGAGGAGTTTGATTGCAAGAAGCAAGAGCTAGGA

>Glyma15g09430 ATGGCACCCAGAAAAGTCAAAGCCATGGGCATTGACTTGGGCACCACCTACAGCTGCGTGGCTGTGTGGAACCATAACCGCGTCGAGGTCATTCCCAACGACCAAGGCAACCGCACCACCCCTTCCTACGTTGCCTTCACCGACACTCAAAGGTTGTTGGGCGACGCTGCCATCAACCAGCGATCCATGAATCCTCAAAACACCGTCTTCGACGCCAAACGTTTGGTCGGTCGCAGATTCTCCGACCAGTCCGTACAGCAAGACATAAAGTTGTGGCCTTTTAAGGTTGTCCCTGGAGCCAGAGACAAGCCCATGATTGCTGTAACATACAAAGACGAAGAGAAACTCCTTGCAGCCGAAGAGATATCTTCCATGGTGCTGTTTAAGATGAAGGAGGTTGCCGAAGCCCATTTGGGTCATTTCGTAAAGGATGCAGTGATCACTGTCCCTGCTTACTTCAGCAACGCGCAGAGACAGGCCACTAAGGATGCCGGGAAAATCGCGGGTTTGAACGTGTTGAGGATCATCAACGAGCCAACCGCGGCTGCTATTGCTTACGGGTTGGACAAGAAAGGGTGGAGAGAAGGTGAGCAGAACGTGCTTGTGTTTGACCTCGGTGGTGGTACTTTTGATGTTTCCCTGGTTACAATTGATGAAGGGATGTTTAAGGTTAAAGCCACGGTGGGAGATACCCATTTGGGAGGTGTTGACTTTGACAACAAATTGGTCAACTATCTCGTGGGTATTTTCAAGAGGAGGTACAAGAAGGACATTGGTGAAAACCCCAAAGCTCTTGGAAGGTTGAGGTCAGCGTGTGAGAAAGCTAAGAGGATTCTCTCTTCAAGTTCCCAAACCACTATTGAGCTTGATTCTTTATGTGGAGGGGCTGATCTACATGCAATTGTTACAAGGGCCTTGTTCGAGGAACTGAACAAGGACTTGTTTATGAAGTGTATGGAGACGGTGGAGAAGTGCCTCAAGGAGGCAAGGATTGCTAAAAGCCCAAGTTCATGAGCTTGTTCTGGTAGGAGGGTCTACTAGAATTCCAAAGGTGCAGCAACTTTTGAAGGACATGTTCAGTGTTAATGGTAACAAGGAACTTTGTAAAAGCATAAACCCTGATGAGGCCGTGGCGTATGGTGCGGCGGTTCAGGCCGCCATTTTGAGTGGCGAAGGAGACAAGAAGGTGGAGGAGTTGTTGCTGCTGGATGTGATGCCGCTTAGTCTTGGGATTGAGACTGATGCTGGTGAAATGTCAGTGTTGATTCCCAAGAACACCATGATCCCCACCAAGAGGGAGAGCGTCTTCTCCACTTTTTCTGATAATCAAACAAGTGTTTTGATCAAAGTGTTCGAGGGGGAGCATGCAAAGACAGAGGATAACTTCCTTCTTGGGAAGTTTGAGCTTTCTGGTTTCACTCCATCGCCAAGGGGAGTTCCACAAATCAATGTGGGGTTTGATGTTGGTGTTGATGGCATTGTGGAAGTCACTGCTAGAGATAGGAGCACGGGGCTGAAGAAGAAGATCACGATCAGCAACAAGCATGGGAGGTTGAGTCCTGAAGAGATGAGGAGGATGGTGAGAGATGCAGAGAAGTATAAGGCAGAGGATGAGGAGGTGAGTAACAAGGTGAGGGCCAAGAACTTGCTTGAGAATTATGCCTTTGAAATGAGGGACAGAGTGAAGAACCTTGAGAAGGTTGTGGAGGAGACCATAGAGTGGCTTGACAGAAACCAATTGGCTGAAACTGATGAGTTTGAGTACAAGAAGCAGGAGTTGGAAGAAAAGTTTCGGAAGTTTAGGTAA>GLYMAGTCTGTACAAAAAAATTAAAATAAATGTGATATAGATATAAAAAAAAATGCAGAGAAAAGTATTAGACATTAGACGGTTACTTTCTGGGAGGGTCTAGAACATTGAAGAGTGGTGTATTAGTATTAGTTATATGAAGCTGTCGGTTCTGCGTCTCATTCTCACATTGTGCTGCTGCGCCACTCAGGTTACTTTCACCTCTTCCACTACTCTTTTCTTATGCTAATTATTATTTTTATTTATGCTCACATTCTCACTACAAACGCAGTTTCACTTTGATGTCGAAATGTATCAAAAGATAATAAGAATTTGAACTGCTTTTATCTATCTTTTGTTTTAACCGTGTGCTTGATGAGGATTCGTTCCCTGGATGCTACTTAACTACCTTAATTAGAGAAAGAGATTCGTTGTTAGGCCCTTTGTGTTATTCAAATTCGTCGAAAGAATAATGCATTGTAAGTGCGAGTTTTTTTGCTCTAGATTGAGTGGAATTGAAATTACTCTTTATAGTAGCTTGTAGTGTACATGTTTGTAAGAAGACCCGCGAGGAATTTGTTTTGTAGTTTTTCACACCAGTACTTTTGGTGATATTCTTGGTATGTTGGATATCCCTCCTTCTCATCAACGAAGGAACTGAAATACGAACGACATCAGAATGTTTCCTATTTTACTTCGTGTGGTTGTTTGGGAAGATTATGTAACTATGTATGATTTTTTTTGTGATAAACTTACATATGAATTTACTTGTATGATGCAAATTTACTTCTAGAAACGTTATCTACACAAATTTCAAGAAAAGTCTAGTTTAACAGCTACAGAATGGATGACTCGTGCTCAATCATGAATCATCGCATGCTGATATGTTTCTTGTGTTTTTTCTTTTATATATGCTCTGTTCCTCACTTCTACGATGGTCATGTGGTTTTTGGACTAGAGTGCTCAATGTTTCCCATAATGAAAATTTCATAAAAAATAATGATAACTGAATAGACTGTTACTTTTCTAAGCTATGAACTCAAACAGTTTATAATGTTTGGGAGAAAACTCACAACTTGCTTCTCTCTGTTAAACGCAGGATGGTGGAACCTGCATATACTGTGACATCTGACAGTGAAACCACTGGTGAAGAAAAATCGTCTACTTTTCCTGAAATAGCAATTGGCATTGATATTGGCACATCACAATGTAGTGTTGCTGTGTGGAATGGCTCCCAAGTGGAGCTTTTGAAGAACACAAGGAATCAAAAGATTATGAAATCATATGTAACCTTCAAAGATAACATCCCTTCTGGTGGAGTTAGCAGTCAACTCTCCCATGAGGACGAGATGTTGTCTGGAGCCACGATTTTCAACATGAAACGCTTGATTGGAAGAGTTGATACTGACCCTGTTGTCCATGCATGTAAGAATCTCCCATTTCTAGTGCAGACTTTGGACATTGGCGTTCGGCCATTTATTGCCGCATTAGTGAACAATATGTGGAGATCCACGACTCCAGAAGAAGTCCTGGCAATATTTCTGGTGGAATTAAGAGCAATGGCTGAAGCTCAGCTGAAACGAAGAATAAGAAATGTGGTTCTTACCGTCCCAGTTTCATTCAGTCGATTTCAGCTAACCCGGATAGAACGTGCTTGTGCCATGGCTGGCCTTCATGTTCTCAGGTTGATGCCTGAACCAACAGCTGTGGCTTTGTTATATGGACAGCAACAACAGCAGACTTCTCATGAGAATATGGGCAGTGGAACTGAGAAAATTGCTCTCATTTTCAGTATGGGTGCTGGTTATTGTGATGTTGCTGTCACTGCTACAGCGGGTGGAGTATCACAGATTAAAGCCTTGGCAGGAAGTACCATTGGTGGTGAAGACTTGCTTCAGAATATGATGCATCATCTGCTACCAAATTCTGAAAATCTATTTAAGAACCATGGGGTCAAAGAAATTAAACAGATGGGCCTGCTTCGAGTTGCAACCCAGGATGCAATTCGCCAGCTTTCCTCTCAGACCATCGTTCAGGTTGATGTAGACCTGGGAGATGGTTTGAAGATATGCAAGGCTGTTAACCGGGAGGAGTTTGAGGAGGTAAACAGAAAGGTGTTTGAGAAATGTGAAAGCCTTATCATACAGTGTTTGCAAGATGCCAAGGTAGAAGTTGAAGAAGTAAATGATGTGATAATTGTAGGCGGATGTTCTTACATCCCGAGGGTGAAAAATCTTGTTACTAACGTATGTAAAGGCAAGGAACTTTATAAAGGCATGAATCCTTTAGAAGCTGCTGTTTGCGGTGCAGCAGTGGAAGGAGCTATTGCTTCAGGCGTCAATGATCCCTTTGGGAACTTGGACTTGTTAACTATCCAAGCTACACCTCTTGCCATTGGGATTCGAGCTGATGGGAACAAGTTTGTCCCTGTAATTCCGAGGGATACTACAATGCCAGCACGGAAGGAGCTAGTTTTCACAACTACTCATGACAATCAAACTGAGGCGTTGATCCTTGTCTATGAAGGAGAGGGTGAAAAGGCAGAAGAAAACCACCTATTGGGATATTTCAAGATAATGGGAATACCTGCTGCTCCTAAAGGAGTTCCAGAAATCAATGTGTGCATGGACATAGACGCTGCAAACGTGCTAAGAGTTTTAGCTGGTGTTGTGATGCCTGGTTCTCGCCAACCTGCGATTCCTGTTATGGAGGTAAGGATGCCAACGGTGGATGATGGGCATGGTTGGTGCGCCGAGGCTCTAAATAGAACCTATGGTGCCACACTGGATTTAGTTACTCTCCAGAAGAAGGCATGAAGTGATACACTACTACACAACATCATTGTATTTGTACCTTTTTTTTTAGTTGTTTTCTTGTGTTTGTTTCGGCAAATAGAAGTTGATGTACATGGCATGTCTGATGTTTGTATTTTGTACCTAGAATAATGGAAACAATGCGTTCCAAAAGCCTTAAGTTCAAAATGATTTCAATGGAAATTTTAAAACATGCTGACGGTAGTTGGGAAACCTTCAAC

>Glyma15g10280 TCATGTGTTGGAGTGTGGCTGGAGCAACACAATAGAGTAGAAATAATTCACAACCAACAAGGCCACAAAACCACACCTTCTTTTGTTGCTTTCACAGACAATCAGAGATTGATTGGTGATGCTGCTAAGAATCAAGCTGTCACCAACCCAGAAAACACTGTTTTTGGTATTTTTTCTATTTAGTTAGTACTGCCTTCCTTTTTATGATTTTTCTATACTTTTATGTCCAGATTTCTTATAACTTTTGCAGATGCAAAGAGATTAATTGGCAGGAAATACAGTGATCCCATTATTCAAAAAGAGAAAACGTTGTGGTCATTCAAGGTTGTTGCCGGTATCAATGACAAACCCATGATTGTTGTTAAGTACAAGGGTCAGGAGAAGCAAATTTGTGCTGAGGAAATATCATCTATGGCCTCATAAAGATGCGGGAGATTTCAGAGGTTTATTTGGAAACACCTCTGAAGAATGTTGTTGTTACCGTACAAAAGATGCTGGTGCTATTGCTGGCCTAAATGTTATGAGTATAATTAATGAACCTACTGCAACAGATATAGCATATGGTCTTAACAAGAGAACTAATTGTGTTGGAGAGCGAAACATTTTCATTTTTGACCTTGGTGGTGGTACTTTAGACGCTGCTCTCCTCACGATTAAGGACGTCTACGAAGTTAAGGCTACTGCCGGAAAAAGTCGGACTTTGACAACAGAATGGTGAATTACTTTGTAGACGACTTCAAAAAGAAGAACAAAGTGGACATTAGTGGGAACCCAAGAGCACTAAGGAGGTTGAGGACTTCATGTGAGAGGGCCAAAAGAATACTACCAACATTGAGTTAGATGCTTTATTTTAAGGGCATTGACTTTTGCTCTTCAATAACTCGAGGAAAGTTTGAGGAAATCGATATGGAGCTGTTTGAAGAATGCATGGAGACAGTAGATAAGTGTCTTACTGATTCTAAGATGGGCAAGGGCAGTGTCCGTGATGTTGTCCTTGTTGGTGGTTCTTCTAGGATTTCCAAAGTGCAAGAGCTATTGCAGGACCTCTTCGATGGAAAGGATTTGTGCAAGAGCATCAACCCTGACGAGGCTGTTCCTTATGGCGCATCTGTGCAGGCTGCTATGTTGAGTGAAGGCATTAAGAATGTTCCAGACTTGGTTCTATTGGGTGTTACACCACTGTCACTTGGTATATTGACCAAGGGAGATGTCATGAGTGTGGTGATTCCAAGGAATACTAGGATTCCTGTAAGGAAGACGCAAGTATGCTGTAATTTAGATAACCAAAAACGTGTTCCTTTTAGTGTTTACGAGGGTGAAAGGGCGAGAGCCAATGATAATAATTTGTTGGGTTCTTTTGTTCTTTCTGGTTTGCCCCCTTCTCCTCGTGGTCATCCTTTAGATGTAAGTTTTGCTATAGATGTAAATGGTATCCTATCTGTTTCCACCGAGGAGAAAACTAGTGGCAATAAGAATGAGATTACCATAATCAATGACAAAGATAGACTGTCAACTGAAGAAATTGGAAGATTGATTCAAGAAGCTGAGAAGTACAGGGCTGAAGATAAGAAGTTTCTTAGGAAGGCCAATGCTATGAATTCTTTGGGTTATTATGTTTACAAGATGAGGAATGTTTTAAAGAAGGATATTAGTTCGCTTTGCTCAAAAGAAAGGGAGAAGATCGATTATGCCATTACTAAGGCCACAAATTTGCTAGATGATAGTAAATACCAGTATGAAGTGGAGGTGTTTGAGGATCATCACAAGGAGCTTGCCAGCTTCTTTGAATCCATCGCGAGCAAGATTGGTTAG

>Glyma16g00410 TTGAGAGATGTGAGAAAAGAAGATCCTAAGAATTACACAGTAAAATAAATATAAAAAGAAAAGAGTTAAAAATGGAAAGGAGGAGATGGTTCTAGAATATAAACAGGTGGCAGCACTGAGAACATAACATAAGCCTCATCCTCTTGAAAAACCCTTGTTCTTCTCTCGAGGATTAGGATTAGGGCTTCCATTACCAACACCAACACCATCACCATCACCATCTATGGCTTGCTCAAGCGCCCAAATACACGGCCTCGGAACCCCTTCCTTTTCCCGAACCCTATTTTTAGGTCAGAGGCTAAATACCAAGGCCGCCTTTATCAAGGTCAAGTCCGCACCCACTCCCAGGAGGCTCCGCCCTCTCAGAGTCGTTAATGAGAAAGTCGTCGGTATCGATTTGGGAACCACCAACTCCGCCGTGGCCGCCATGGAAGGCGGTAAGCCCACCATCATCACCAACGCCGAGGGCCAGAGAACCACTCCCTCCGTCGTGGCCTACACCAAGAACGGCGACAGGCTCGTGGGCCAAATCGCCAAGCGTCAGGCCGTCGTCAACCCCGAGAACACTTTCTTCTCCGTCAAGAGGTTCATCGGCCGCAAGATGTCTGAGGTCGACGAAGAGTCCAAGCAGGTCTCTTACAGAGTCATCCGAGACGACAACGGCAACGTCAAACTCGACTGCCCCGCCATTGGCAAACAGTTCGCTGCTGAGGAAATTTCTGCCCAGGTCTGTCTATTTTTGCTTTACTTTACTCGTTCTTATGTTGCTATCTAATGTTATTATTATTGATACCAAGACTTTTGTACTGTTTTATTATATATTATAGGTTCTTAGGAAGCTTGTGGATGATGCTTCCAAGTTTTTGAACGATAAGGTTACCAAGGCTGTTGTTACTGTGCCTGCTTACTTCAATGACTCCCAAAGGACTGCCACCAAGGATGCCGGTCGGATTGCTGGTCTTGAGGTTCTTCGTATTATCAATGAACCAACCGCTGCATCCTTGGCCTATGGCTTTGAAAAGAAAAACAATGAAACAATCCTTGTTTTTGACCTTGGAGGCGGCACCTTTGATGTCTCTGTGCTCGAGGTTGGTGATGGAGTGTTTGAGGTCCTCTCTACTTCTGGTGACACCCACTTGGGTGGTGATGACTTTGATAAGGTACCTTTCCCCTTCTTTCTTCTCTTGTCTTGTCTTGTCTTTAGTAGTGTGAGTAATGAATGATTTTCTTTTTTACTTGCTAACTGCTTGTTTCCTATTATAGAGAATTGTTGATTGGCTGGCTTCCAACTTCAAGAGAGATGAAGGCATAGACCTTTTGAAAGACAAACAAGCTCTTCAGCGTCTCACTGAGACAGCCGAGAAAGCAAAGATGGAGCTCTCAACATTGACTCAAACTAACATCAGGTATTATTTACTTTTCTCCTCAATCCTATCCTGTTGCTTTTTCATTGTGCTCTTGTTACCAAACCACTGTTGACATTAATGCCATATGTTTTCAGTTTGCCATTCATAACTGCCACGGCTGATGGACCCAAACATATTGAGACCACCATCACAAGGGCTAAATTTGAGGAATTGTGTTCAGATCTTCTTGACAGGTACTGAATTTGATGAATGGATGGCAAACATGATTTCACTAAAGCTGAACAAACTACTCAGCTACAGTAATACTTGTGTTTCAGGCTCAGGACACCCGTTGAAAACTCATTGAGGGATGCAAAACTCTCGTTTAAGGATCTTGACGAAGTCATCCTTGTTGGTGGATCAACACGTATCCCAGCTGTTCAGGAGCTTGTAAAGAAGTTGACTGGCAAGGACCCAAATGTCACTGTCAATCCAGATGAAGTGGTTGCCCTTGGAGCTGCTGTTCAGGTGGGATTTGGCTTTTATCATTAATTGGAAGGGGACAGAGAATGTTTGTTTAAGCTTTTCAGTGTTTGCTCAAATGTTTTGTTTTGTGGATAGCATATTATTTTTTTGTTAATTAATGCTTCTTATTGTAATTTATGTTTTTAGGCTGGTGTCTTGGCTGGAGATGTCAGCGACATTGTGCTGTTGGATGTCACTCCATTATCTTTGGGTCTGGAAACTCTAGGTGGTGTGATGACAAAAATTATCCCCAGAAACACTACCCTTCCCACCTCAAAGTCTGAGGTTTTCTCAACTGCTGCTGATGGACAGACCAGTGTAGAGATCAACGTCCTTCAGGGTGAGAGAGAATTTGTTAGGGACAATAAATCACTTGGTAGCTTCCGCCTGGACGGTATCCCTCCTGCACCTCGTGGTGTTCCCCAGATTGAGGTGAAATTTGACATTGATGCCAATGGCATTCTCTCCGTCGCTGCTATTGACAAAGGCACAGGGAAGAAGCAAGATATTACCATTACTGGTGCTAGCACCTTGCCTTCAGATGAGGTATCCACTATCTCTGACAATCAATTACTTTAATAGCCTTTTTATGTTGTTTGTGTGTGTTTTTTTTTTTAATTGCCAGTTGCTTGCTTCTTTTATGAAGAAAAAAAAAAAGTGCCAATGTGAAGGGTCATTTGAGTTAGGAGTCTTTTTATGATGCTTAGGACTAACTCTGGAATGACATACAGGTGGAGAGAATGGTAAACGAAGCTGAGAAATTTTCAAAGGAAGACAAAGAAAAGAGGGATGCCATTGACACAAAGAACCAGGCAGATTCTGTGGTGTACCAGACAGAAAAGCAATTGAAAGAGCTTGGAGATAAGGTTCCTGGCCCTGTAAAAGAGAAGGTTGAAGCAAAACTAGGGGAGCTTAAAGATGCAATTTCTGGGGGTTCAACCCAAGCTATTAAGGATGCCATGGCTGCACTGAACCAGGAAGTCATGCAGCTTGGTCAGTCCCTTTACAACCAGCCGGGAGCTGCAGGTGCAGGAGGGCCAACACCACCTGGTGCCGACTCTGGCCCCTCAGAATCCTCAGGTAAGGGACCCGACGGAGATGTCATCGATGCAGATTTCACCGACTCTAAATGAGCAGCAGCCAGCTAATTACACCCATAGAGAGTATATGTTAGTATTTTTTTCCCCGTTATATGTTTTCTTCTGTTCCAAATTCTTAATGATTTTTGTCAATGGAGAGTAGGTGTGTGTTTCCTTCAAAAATTAGACACGGATTTCATAGGTGCTCATTGATGCTCGCTTTGTACCCTGGAATTTTGTCTCAGTAACACTGGTATTATTATGGGTGCAATTCATTTTATATAGGCTGAAAGCGTTCCACTAAGCTTATGAAACGTGATGACAGATTATCAGGGAAAACGATTCTTCCAACCTTTACTTTAACGTTGCAAACCACTCTTCGGAGGTGATTGCTTAAGGAATTAACAAGTAAAAAAATCATTTAAAATTATGGTGGGATATTTTACTTTTGTTAAA

>Glyma17g08020 CTTCAACTAAACTGCTAAAGTGAATATTTCTCGATTTCTCTCTTACTCTGTTTTCTTTCTGGAACATGATCAAACACTCGAGAACTCTCTAGCCAGCTCTGCTACTTTCTTCCTAATCTCGTATATTCTCGAACTCACTCTAATTTCTAACGCGGACTATAAATTGCGGTTTGGGTTCTCTCTCTCTCCTCACTCACAACAATATCTGCACATAAAAAACCACTCTACAAAACTCTCCAGTAATTCTGAAAATTTTATTTTCTTTTGGTGAAATTAACAGCTCTGTGATTGATCAATGGCGACAAAGGAAGGCAAAGCCATAGGCATCGATCTCGGCACGACCTACAGCTGCGTGGGCGTGTGGCAAAACGACCGCGTCGAGATCATCCCCAACGACCAAGGCAACCGAACCACTCCCTCTTATGTAGCCTTCACCGACACCGAGAGGCTCATCGGAGACGCGGCGAAGAACCAAGTCGCCATGAACCCGCAGAACACCGTCTTCGACGCCAAGCGTTTAATCGGTCGCAGATTCTCAGACTCTTCAGTTCAAAACGACATGAAGCTGTGGCCGTTTAAGGTCGTGGCTGGCCCTGGCGACAAGCCCATGATCGTGGTCAATTACAAAGGCGAGGAGAAGAAATTCTCCGCCGAAGAGATATCTTCCATGGTGTTGGTCAAGATGAGGGAAGTGGCAGAGGCGTTTCTCGGACACGCCGTGAAGAACGCTGTTGTCACTGTCCCTGCGTACTTCAACGACTCGCAGAGGCAGGCTACGAAGGACGCAGGGGCAATTTCGGGTTTGAATGTGTTGAGGATTATCAATGAACCCACCGCTGCTGCCATTGCGTATGGGTTGGATAAAAAAGCTTCGAGAAAAGGTGAACAGAACGTGCTTATCTTTGACCTTGGTGGTGGTACTTTTGATGTTTCGATATTGACCATCGAGGAAGGGATTTTCGAAGTGAAGGCCACTGCTGGTGATACTCATCTCGGAGGTGAAGATTTTGATAACAGAATGGTGAATCACTTTGTTTCTGAATTCAAAAGGAAGAACAAGAAGGATATTAGTGGGAATGCCAGAGCGTTGAGGAGGTTGAGGACAGCGTGTGAGAGAGCCAAGAGAACGCTCTCTTCCACAGCGCAGACAACTATTGAAATCGATTCACTATACGAAGGGATTGATTTCTATGCTACAATTACGAGAGCTAGGTTTGAGGAGATGAACATGGATTTGTTCAGGAAGTGCATGGAGCCGGTGGAGAAGTGTTTGCGTGACGCCAAGATAGACAAGAGTCAGGTTCATGAGGTTGTGCTTGTTGGAGGTTCCACTAGGATCCCCAAGGTTCAGCAACTCTTGCAGGATTTCTTCAACGGGAAAGAGCTTTGCAAGAGTATTAACCCCGATGAAGCTGTGGCGTACGGTGCTGCTGTTCAGGCCGCGATCTTGAGCGGCGAAGGAGACGAGAAGGTTCAGGATTTATTGCTGCTGGATGTTACACCACTCAGTCTCGGTCTTGAAACTGCTGGTGGTGTCATGACTGTGCTGATTCCGCGGAACACAACTATTCCCACGAAGAAGGAGCAGATTTTCTCAACCTATTCTGATAACCAGCCCGGGGTGTTGATCCAAGTGTTTGAAGGAGAACGGGCTAGAACAAAGGACAACAATCTTCTCGGGAAGTTCGAGCTTACAGGGATCCCTCCAGCACCAAGAGGAGTGCCTCAGATCAATGTCTGCTTCGACATCGACGCTAACGGGATTCTGAATGTCTCTGCAGAGGATAAGACTGCTGGTGTGAAGAACAAGATCACGATCACAAACGACAAGGGTAGGTTGAGCAAGGAGGAGATTGAGAAGATGGTGAAGGATGCAGAGAGGTACAAGGCAGAGGATGAAGAGGTGAAGAAAAAAGTGGAGGCTAAAAATTCGCTTGAGAATTACGCGTATAACATGAGGAACACGATAAAGGATGAGAAGATAGGAGGGAAGCTGAGCCCGGATGAGAAGCAGAAGATTGAGAAGGCTGTGGAGGATGCGATACAGTGGTTGGAGGGAAACCAGATGGCGGAAGTGGACGAGTTTGAGGACAAGCAGAAGGAGTTGGAAGGGATCTGCAACCCCATCATTGCTAAGATGTACCAGGGTGCTGCTGGACCTGGTGGAGATGTTCCTATGGGTGCTGACATGCCTGCTGCTGGTGCTGGACCTAAAATTGAAGAAGTTGACTAAGCTTGTGGAATATCACTAACTTCAACTTTGGGTTGCTTCTTTCTTATTAGGTGCTCTGCCAGAGATGTAAGCTTTAATAATGTAAGTTTTAGTGATGTAAGGTTTCATGAATCGTGAATAAGGTCTTTTTAGTTTCATGTGAATTTCAGAGATGATGGAGCTTGAGATTAATGTAATTTTTATACTGTTTGGCTTTTTAATGTAATGTGATTTAAAAAAAAACGTTTGTTCCTGTCTGCTTTATCAATTCCCCGGAGAAAATTATGACGCTTTCTTTTCATGCATCCAACACCTAACACCTGTGCTTATG

>Glyma17g11650 TCAAAAGAATTGAAAATAAAATATTTTTTCTGAAAATAAAAGTAAAATATACTCCATAATTTTCTTAAAACCCCCTTTGAACCCTAACGCCCAGCAGTACAGCATCGTCGCCGTCAACACACCCTCCGTCTGCCGGCGTCGCACCTCGCACGAAGCCACAGGTTTTCCCAATTGACTCATGGATATATTCAAGCCTTTTTTAAAATAAATAAAAATTGATTTTATCATTATGTATCATTGATTTATTCTCCCCGTGAATTGGCTACACTGCTATATGTTTTCACTACCCTGTGAATGATGCGGAACAATTCTTTGTTTGTAACACTCTAGGCTCAAAAAAATATTTTCTTTTCCCTCTTTTTTATTCTGGGTTTTCTGATTATACACTCTAGCCTAATGAATAACCAGTAATCAGCTTTTTTGTTTATTTTTTCCCCGAAAGTGAAAATGAAGAGAATGATAGCGTTGGGATTTGAGGGTTCAGCAAACAAGATTGGTGTTGGGGTAGTGACCTTAGATGGCACAATTCTGTCAAACCCACGCCACACATACATCACCCCTCCTGGTCAAGGCTTTCTTCCCAGAGAGACAGCACAGCACCACCTACAACACGTTCTTCCCCTCGTCAAATCCGCTTTGGAAGTCGCACAAATCGCTCCGCAGGACATTGACTGCCTCTGCTACACCAAGGGTCCCGGCATGGGAGCTCCTTTGCAAGTCTCCGCCATTGTTGTCCGTGTTCTCTCACAGCTTTGGAAGAAGCCGATTGTTGCTGTCAATCACTGCGTGGCACACATCGAGATGGGAAGGATTGTAACCGGTGCTGATGACCCTGTTGTCTTGTATGTTAGTGGTGGCAACACTCAAGTCATTGCTTACAGCGAGGGGCGTTATAGAATCTTTGGAGAAACTATTGACATTGCTGTGGGGAATTGCTTGGATCGCTTTGCAAGGGTCTTGACGCTTTCCAATGATCCAAGCCCCGGATATAACATTGAGCAGGTGGTGCTCTACTAAAACCCTATTTGAATAGACTTTTTCACAAGTACTTGTAAGAGAAGAAAATGAATAAACTTCTCTGTAAGTACTTGTAGGAGAAAAAGATAAGAAGGCAAAATGAATTGATGTTTTCCATTAAGTTAAAATCAACTTATGTAGCTCGGCTTTTGAAGAAGTTAAGTGAGAGAATTTTTACAAAAGTTAAGTGCATAAGTTGATTTTAGCTCATCAGAGAGCTTTATTCATTTTATTTTATTATTCTACTAGTGTTTATCGAGAAGTTTATTCAAACAAAGACTGACTTGTTTTACATAGTTTAGGCCATGTGCATGATATGATATATGGGCTATTTTTGTTATCCTTTTTAATGGAATTGTCTTACCTTAAATATGAATTTTTTTTCTCAACTTGGCTTATGTAGTTTTATTATCCTTCAGCTTGCAAAAAAAGGAGAGAAGTTTATAGACCTGCCTTATACTGTTAAAGGGATGGATGTATCTTTTAGTGGAATATTGAGCTATATTGAAGCAACCGCTGCTGAAAAGCTAAAGAATAATGAGTGCACTCCTGCGGACTTGTGCTACTCTCTGCAGGTGAATTCAATTTGTGCTGATCCCTTTTTCTCCTGTTGCATTAAATTAATGAATAAATGAATTTGCAAGTTGATGTATTTTAGTATGTTGCAGGAGACACTGTTTGCCATGCTTGTGGAGATAACGGAGCGGGCTATGGCTCATTGTGACACGAAAGATGTGCTTATAGTTGGTGGTGTAGGTTGCAATGAGCGGTTGCAAGAGATGATGAGAATCATGTGCTCTGAACGCGGCGGAAGATTGTTTGCCACCGATGATAGATATTGCATTGATAATGGAGCAATGATAGCTTATACTGGCCTCCTTGAATTTGCTCATGGTGCATCAACTCCACTAGAGGATTCTACGTTCACCCAGCGGTTCCGGACAGATGAAGTGAAAGCAATATGGAGAGAAGCAAATTTGGAAAATTTGAATGGGCTTGCAGAGAAGAGTATTTGATTTGTCAATCTTGAGGTGCGATCCAATAGATTAGTGTAACATTGATTTTAAAGTGAAGATTTATGTTTGGGTATTTTTATTCTAAAAGAAAGTTAATTGTAAAATTCAGTATGAAATTTTCGATCCAACACAAAAGTTACTTAAATTTACTTCAGTTCAGAGTCAATTCTTGAACATGAGACTAGACATCTGAACATTTACCTAAAATTATATCAGCTCATATTTTAACATGATTTTGAATGTCTTAACGTAAATCAAACACGCACTAAATGTATGCTTAGTTTTGCCAAAAAAATATTTTCCATAGTCAAACTTAGGTGGGAAATTTGAATAAATTGTTGTGTTAAATTCATTAATTAAGATCCACGACCCTGCTTTCTTTTCTCCAGTTTTAGCATAATATGTCAAAACTCAAAAGGACTTGACTGCATCATTTGTTACTATTTGGTTTCTACTAAATCTGTGATAAAACAACAATATAAATTGAATATTTCGTGTTTCATAGTCTAAGGAATTTATTTTTCCTCTGACCTCACAGGTTTGAGCCTGAGTGCTCATCAGTTTGTCTAAACCAATGTCTATGGAAAGCCTTTGCAGTTTGTCCTGGTATTGGTGACTTTGCAGTAACTCTTTATCTCTCTTAGGACATATACTGGTCTAAAACTTATCCATGTAATATCTATGTGTTCACTAAGGCAGTCAATCTTTAGGCTTTGTTTATGTATTGGCCTGCAGATCAAGTTTGCCTCTGCATTTCACTATTGGTGACAAGCACTGAAAAATTATGAAACATAACATTTTCAGCATATCAAGTAATTAGGTTGGTCTTTGGAGAATTTCATCTATTACCTTAATAACCCCCTCC

>Glyma17g14280 CGAAAAGGAAAGTTGAATTGAGTTGAGTGGATTACGGTGGGCGTTAATAACTAAAGTAATATTTCCTAACACATATCCAAATAGAAGCTACTAGAAGCCCAATCCGTCAAGAAAAGTCTAGAGACCCTGTCGTGTAACATAAAAACCACCGAAGAAAACATCACTGCGCCGCGCTGCCACACCAAATAAACACAAACATGGACGCATCAAAACTCAATCAATTGAAGCATTTCATCGAACAGTGCAAGTCCAACCCTTCCCTCCTCGCTGATCCTTCACTCTCCTTCTTCCGCGACTATCTTCAAAGGTAAAATAAATAAAATTCAATTCAATTTAATCAGCACTCGTATTTGCGTTCCAAGACCACACTTTTATTATTATTAACTTACTGTCTTTGATTCTTCTTTTCTTCAGTCTCGGCGCGAAACTTCCTGAGTCTGCTTATTCCGAATCGGTACCCTAATGTTCTATAAATACTTGCCACTACTTTTTTAAAAAAAAAAAAAAATCGCGCGTTGTGTGTGAATCCTTATATACGTTGCTTTGTGTTGTGTACGGAAGACGGGCGTGGAGAGGGATGAGGACATAGAGGATCTTACGGAGGAGCACGAGAAGGTAGAAGAAGAAGAAGAAGAAGAGGACGATGTAATTATTGAATCCGATGTTGAGCTTGAGGGTGAAACCTGTGAGCCTGATGATGATCCTCCACAGAAGGTTTTTTTATTTTAGTTTTTGTAAATAAAAAAATAAAAATTTTAGTTGGTTCATGTTGCATTTGGGTTTTTAATTAAGGCAAATGAGTGTCACGTTTCAGATGGGAGACCCCTCTGTCGAGGTTACTGAAGAGAATCGTGATGCATCGCAGATGGCCAAAATTAAAGCCATGGATGCTATTTCTGAAGGTAACATTCATTGTAAAGCGGATAATGCTGATGTATGTGATTTATTTATTTATTTATTTTTTACATGGCAATCGTTTGGGATGTGGATGGATTCACAACCATTGTGCAAGTAAAAAGGAAATACGAAAACATTAAGAGGAGTGGCCTTATTTATCTGTGTCAATGATACTTAGAATGCACAGAAAAAAATGCAGTAAGATATACTACAGTTTTTGGATTGGCACAACGATAAAGTTGGCCTTGGTAATTGGTTGGTCATGGGTTCAAATCTAGAAATAGCCTCTTTGCATATGCAAGGATAAGGCTGTGTACAATGACCCCCCATACCTTCGCATAGCGAGGAGCCACCGGGTACTGGGGTATGTTAGTTTCTTTTTTTTTTAGGATGGCTCGGGTTGTGAACTCTTGTAGATGGTAAGGTAGGCAGGAAGGAACCCTGTAGGATTCAAGGATTTTTTTTTTTAAAGTTTTTACTGTATAAGGAAGCTTGTATATCTGTCTAGTTCATAGGATGTAACTGACATTATTTGTGTTGTCTGGAGCCATCCCTAATGCTAGATGCATTCTTTCTTTTGTTATTCATCATTTGGAAATCAATGCTAAACATATGGAGTTTGATGCTTTACATCCTCAGTTTAAGTATGTGACCTTTTACCAGCTAATAAATGCTACCTCTTCTCAAAGTGTTTAGGTAATTCTTTAACAGGTTGTATACACAAATACTCAAAGAACATGTATTGTGCATTGTCGCATTGTGCAATTATTCTTCCAATCAAAGAGCAGCATTTCAAATTTCATAGTATGATCTGTAGTGTGGGAAACACTCTCCCTAGCTTAGAATGTGGCACCCTAGATAACAAGCAGGTGTTGATTGTGTGGTTGGATGATATTACCCTCTGTTCCGTCATGCTCATATGACTATCTTCCCTCAATCAACCACAGGCTCTGATAGCACTTGTGGATGAGGATCCATAATAAACATACAAGGATGAGGAAATGAAAACACAACGACAATAATAATAAGTTGCAAACTCACCTCTGTCCATGATCTTTAGAATGGATAAATTTTTTTTTCCATTAACAAAATATGCTACATCTTTTGAACAACCCAAGTTGCAAACTCTCCCACTAAACTCACCCCAAAATACTCCAATTTCAATCATCTATCAAGTGTTTATGCTATTCTTTCTGGCAATATTATTTAAAACTGCAACAGTAATTGGAGATCATTTGACTATGTAATGCAGGTAAGTTGGAGGAGGCGATTGAGAACTTAACAGAAGCTATTTCACTCAATCCTACCTCTGCCATAATGTATGGAACTAGAGGTATGACCTGATTTAGTTGTTCTCTTTCTACATTGTTTTTATCACCAAGATAAGGATTAATTCTTAATATTTTATAAATCATTTACAGCCAGTGTTTACATCAAAATGAAGAAACCCAATGCTGCGATCCGTGATGCAAATGCTGCTTTGGAGGTTATATTCTCTCCCCTATGTTGCTTAGGCTGACAAACTGAATTACATCCTTGCTTGTCTTAAAATAGATCTGTGAATAAAGTGTACGTGTTTAATGCTTTTGTTGCATTATTCTCTGAAAGAACAAGGCACTAGAGTAAACTTGTCTCAAATGTGAGCAACTAAGGCAATATCCATTCAAAAATACTTAATGGGTTAAGATCCTGATAAACTTCAATCAAATTATTGGTAAATACACACTTTCTCACTTGACTAAACTCATTTTGGGGGAGCTTTTTCCCCTATGAAGTGCATGATTTTGGAAATATATAGGATCTAAGCATTTTTAAAATTTGGGCTTTAGTTTTTTCTTTTTTTGGGGGGTGGGGGGGAATCTGTAAACCAAATATTTCCTGTGTTACATAACTCATTAGGCAGTCACTCCTATAAATGTCAAATCTGCCCCTTCTAAAATTGTATTGTATAGCACTCCTGCTTTTTTTTGGTTCTGCCCCTGCTTGTGCATATGACAACTGGATTGAGGGATTATGGGTTTCAATCAGTGTTATCAATGGCGGAAGACCAAAATTCTGCCATATAAACATGCCATTGCAGCCTATGCCACTGCCATAGCAGGCTTCCCTTCACAAATTGCTTATGGCGGCCATAGCGCTGTCATTTAACAACCCTGGTTTCAATAACATTTCTTTGTTCCTGTATACAAGGATTTTTATTTTATATTGCTTTTTCCAGATTAATCCTGATTCTGCTAAAGGATACAAATCACGTGGCATAGCACGAGCAATGCTTGGTCAATGGGAAGAAGCTGCAAAGGATCTTCATGTGGCTTCAAAGTTAGACTATGACGAGGAAATAAATGCTGTACTAAAAAAGGTTTTCTTTCTTGTCCCTTCATGTCATTTATCTTCCACTGATAACAGCCTCTAGTAGTGGGTCATACAACCAGTGCAATCTGTTGTTTTTTTACAGTATGTGATGTTATCCTCATAAGTGATGGATTCCTTGAATTGATTTACCTGTAAACATAAAAGGAAATATACCTTGAAATATTATTTGGTGTCTCATTGTAAATATTGGATTGCCTAGGTGGAACCAAATGCTCACAAGATAGAGGAACACCGTCGGAAATATGAAAGGCTGCACAAAGAAAGAGAGGATAAAAAAAAGGAGCGTGAGAGGCAGCGGCGCCGTGCTGAAGCTCAGGTTCACAATTATGATATAGATATGCTAAAATAGAGCATGCCATAAAATGTCATACCCATGTTATTTAGTTGCTAATTTGCTGTGTATTTTTCCAGGCTGCCTATGAGAAGGCTAAGAAGCAAGAGCAATCATCTTCCAGTAGAAATCCTGGAGGCATGCCTGGTGGGTTTCCTGGGGGCTTCCCAGGGGCCGGGGGCATGCCAGGGGGCTTCCCAGGGGCCGGGGGCATGCCTGGGGGCTTCCCAGGGGCTGGGGGCATGCCTGGGGGATTCCCAGGGGCTGGTGGCATGCCTGGGGGATTCCCAGGGGCTGGTGGCATGCCTGGAAACATTGATTTTAGCAAAATCTTGAGTGTAAGTAAATCTGGCCTTTGGATGTTTTTGTGTGCTTTTGAGTTGCTCTAAAAAAAATCTCTCTCCCTAATGAACATGCGCACAAAGCTTATTTTCGTTTTGATATCAAATGTTGCCTGATAACATTTCTCAGGACCCTGAACTGATGGCGTCATTTGGTGATCCAGAGATTATGGCTGCTCTTCAAGATGGTAATTATCAGTTCTAATGTTTGGTTCCATTCTTTTTCTGTTCTACTTAATTTGGACTTCTCTCTATTTGAGAACCTATTTGACGTTGGCAAAGCTTGAATTTTTATAAACTTCTGACTTAGTTGAACATGCAATGTCATGTGCTCGTCTTTGCATGGTTATTTTTAGATTTAAATACTCAGTAGTTGACAAGACTGAGAAATATATAAATCCCCTGCTCTCCGCTCTCTCTCTGTCTGTCTCACACACATTTAGTAGTTCCTATCCTGTTTGGGCATAAAGACCAAATGAAATTGATTGACCTGGCTACTGGTTGTGAGGAATTGAATGGGCAGTGGGACAGTTTTGAAGTACAGCTTATAAAATGTGAAAGAATGAAACTTTTTATCGTTTACAACTCTACATAACAATTCATAAGAAACTGCTCTCTCTCTCTCTCACACACACTCACACAGACACACAGTATTTTCATATCCATACTAGCTTAACCTGCTCAGGAATAAATGACCATTTGAAATGATCTATTAGGAAACGTCAAGTTCGATAGGAAGTTGATGATGACCCAGTTACACTAAATATGTAGAAAATAGTTGATTTGCTGGTAATTAATTCCAATTTCTCCTTTTTTATTTTTTTTTGTGTGCAGTTATGAAGAACCCTGCTAATTTTGCCAAGCACCAATCAAATCCAAAGGTAGCTCCTGTAATTGCGAAAATGATGACCAAACTTGGAGGTGGTCCCAAGTGAATACTTTTAGATGCTTGTAAGTTATTTGAGACTTCAACTTTCCAGCCCTCTGGCATCGGGTATTTAATGTTTATTGCATTTTTAATCTTACCGAAATTATTAAACTTTTTACCGTTTGTTAGTCCTGTTTCTTAGGTGCCCCTATATAGCTCGTGTTGGTAGTATGTTTATTTGTCAACAATGCAATGTTATTTTCCCGAGTGAATTTGAACGTTTGAAGTTTATATTTTTGCGTTTCAGGAAAAATGAAATAATAGTTTCTTAGCTTTGTCCAGTGATTCCTGTAGAGGTCTGG

>Glyma18g05480 CCACAACACAGGCCAAAACCCAAACCTTGACTTGGCTCGCCACTCCACACGCCATTGCAAGTCCCGTGCTCTCTCGCTCCTTCACCTTCTACTCCCCAAACTCGAAACCCCACTTACTGAACTTTGTTAAAGTTCATAAATTTCACAAAATTTTTCGACCTTCTTTGCTTCAAAATCTACTTCCTTCAAAATCTACTTCCTCTCCTATCACTAACCGTTTCAATTTTGATTTTTGGGCTCCATAGCTTGGAAGGCAGAAGAAGATGGAGAAGCTTAACTTGGCCCTTGTGTCTTCCCCAAAGCCTTTGATGTTGGGACATGTTCCTGCAATAGACGCCACATCCAGAGACGTTTTCAGAAGAAAGCACTTCTCTTTTGGGAGGGTCTTAATTGCTCCTCATCGTTGCAGATTCCGTGTTTCTGCACTCTCTTCCTCCCATCGTAATCCGAAATCTGGTATTCAGTTTGTAACTTCTTTTTTTTTTTTTTTGTTGATTATTCTCTTTCTATGTGTGTGGGGCATTATAATTTTGTTAGAATTTATGCCTGGTAGGAAAAATATTCACTGAATCACTGGATTCTTATGGATTCAATTACATGAAATGTGTGTTTTGAAGTTGAAGGTGAATTGATAGGTCTAATGATGCTCATGCTGTGTTTTTTTTTTTTCTTCATCATTGTTCTAAAACTTAAAGAAGAATCTGAATTGAACATGTTGGTTCTTTTGTTATTGGACTAGAGCCATGAATACCCTATGTTGACATTTTTAAGGGTTTATAGGAGTGTTTGGATAAGCAGTTAATGAAAAAACGTACTTTTTATCACGTGACCTTTGTTTTGCAAGCCACTCTTTCTGAATTTCTAGAAAAGTTTAAAACTTTTTTGTTTCCTTTGTGATTTTGGCTTCTAAACGTGTTAAGATAAGTGTTTCTATGGTGAAGCACTTTTTTCTTAAGAAAGTACTAACAAACAGGCCCTAACTTTTGATAAATCACCAAAGCGTTTTTGCAATTAGTGTATAGTGTTTGTGGAAGGAGCGGATTGTGAATTTTGGAGCAAGGTTATGCTCAAGGTCGATTGTGGTTGATAGCATTTTGAATCCATGGTTAAAAATCTATTGCATATATGTGTGTATTTTGAATCCATTTCGGCAATGCATATTTATCGTGTGTAGTTACTTCTCTTCTTTTGTGGTTAATGAGTGCTTACTTGTTGTACAAGTTGAATATCTGTCAATATTTTTCACTCATCTCTTAAGATGCCCAAGTAATGCATGCTGGGAATACAGTGCAGGAGAAGCTGATAGTAAAGCATTTTGCTAGTATTTCTTCTTCGAATACTCAAGAAGCGACGTCAACTGGAGTTAACCCACAATTATCACCATCTTCAACTATGTAATCATTTACTCTTTTTTTTCTTTGGCATATTAATTTTCTTAAAAAAAAATTGTCATATTTTTATAATGGGTAACTACTAACTACTGGGGGTGGGGGTGGTTGATCGATACTTTATTGATTTAAATTTGTTGATATCTATTGCTTATTAGTTCACATTCTTATTGGTCCTATTTACTATATTCTAGAGGGTCACCTCTCTTCTGGATTGGTGTTGGTGTTGGGCTTTCTGCACTGTTTTCAGTGGTGAGAACTGAGAACCATAATCACAGATCTACCTTACATACACTGATAATGAGTTTATCTTTGATGTATGTTTAATAAATTGTGTTTTTTTGCAGGTAGCTTCAAGATTAAAGGTGAATGCCACTTTATTGCAAGGCATTGATATAAGCTGGTTCATTCTTTGAGTTCCACTGTAATTATGGGTGTAAGAAATTTTGCTAGTCATGGGTGTTAGGCAATAAGCTGGTGTATTCTTTGAGCTCTACTGTAAATATGGGGTATAAGATGTAGGAAATTTTGCAAGTCATGGTTATAAGCTTGTGCACTCTGAGTATCTGAAGTACCAAACACCACTCCAACAATTGTTTTATAAGTGTCTGTGCATTCTTTTCTAGTATAGTTTTTAATTTTTACGATGGGATTTCTTATGAAATTACTTGGAAGTTGAAGGCACGTATCCACCGGCATATTGCTGTCGACACTCTAGAAATAGAAATACACAAATCATGCAGATTGGCTGTATCTATTTTTAGGAACATAAGTAAACCGCCCAACTCACCTCTTTTATCTGTTAATTTTGTTTGGATAGTATGTTGTTTGAATGAGACTACTCAAATCATATAGCATTCTGAGAGTTTTATTGAATTTGAGATTTTGATTTATCTTTATGAGAATTTGGGTATTGTTTGTGATGGCATTTTCTATTGTTTGTGAAAGGACTGGGAGCATGCATCAAGAGGCTGAGATTAAGCCTACAAAGAAAGTCTTACAAGTTAATTCATGTATGCCTAGTAATAGCCTAAACTTGCAAAATTTTATTTTTGGGCTTAATCTTAGCTTCTTGATCCATGCTTCCAGAGTCCCAGTGCTATCACTTTGTCAGTTGTAGTAGATGCTCTGTCTAACCTTATTTTTCATGGATTTTGACTAGTTTCATTGTAGAATTACAAGAGCTAACCCTGAACATTTTACATATTTTAAACATTTGTCTTTTGATTTTCTTTTAGAAATATGCAATGCAACAAGCTTTCAAGACCATGATGGGCCAGATGAATTCACAAAATAACCAATTTGGCAATGCTGCCTTTTCTCCGGGATCTCCTTTTCCCTTTCCAATGCCTACAGCAGCAGGGCCCACTGCACCTGCTAGTTCTGCAACTACTCAATCTCGAGCACCTTCAGCATCTAGTGCATCTCAATCCACTATCACAGTAGATATACCTGCAGCAAAAGTAGAAGTTGCTCCAACTACTAATGTTAAAGATGAAGTCGAAGTAAAGAATGAACCCAAAAAAATTGGTAACTTAAACGTAGAATCTCTTCTTAGCAATCCTATTTCTTTCTTGTTAGATGGGACTTACTACTTGTAAAATTTATAAATGGTGAGGTAATGTGTCCGCTAGGAGATCTATGTGGTTTCAGGTTGATTAAATAAATGTTATTAATTAAATAATTAATAATATTGATTACTTGATTTCCTTTATTAATTAATTTTAAAAAAATCAATCAAGGTAAAAAAAAATTAAAATTTTTTGCTACAAAGTTGAAGATGGAAAACATTCAGGGAATTATCTTTATAAGTAGCAGAGATTCTATCTGATGTCAGCTTTATATTTGTGTATAAGTTGAGTCTTTTCTTGTTAAAATTCTTAAAATTTTCCATTACAGCTTTTGTAGATGTTTCTCCAGAAGAAACTGTGCAGGAGAGTCCTTTTGAAAGTTTTAAAGATGATGAATCAAGTTCCGTCAAGGAAGCTCGGGTTCCAGATGAAGTGAGTTTTTAAGATACATGTTTTTAGGTAAAGAAACTCAGTGACTTGTGTTGTTCTTAATATGTATGCATTTATTGAAGGTTTCTCAAAATGGAGCCCCCTCTAACCAAGGTTTTGGTGATTTTCCTGGTTCTCAATCTACAAGTAAGTACTAAGTAGAGTCTGTTGAAATTGAAAATATTTACAATTCGGCTTTATAGGTGGAAAACCCCATGCAATGTGATGCATCTACTCAAGTCTCAATTTCTAGGAAATGCATTGACATGACATGGTTTTAGTCTTTTAGATCAGTCCTGCCAAATAGATGAGTTGTACTTGTTGTCATTATGATATTAAATTTAATCTTGTTTTCATTGCTGAGTTTATCATGGATCATGTGCACTTCATATGTCTTTGTAATTTTACTTTTTAACTTTGAAGTTTTTCTTGTCAACTATCAATTACTTTTTGACTCTGATTAAATACTTCAGTACTTGGATTGAAATGATGTCATAAATCAAATATGACTAGTTTCATACATCATTGTTTTCTAATCACATTCATACTACTGGTTTAAATCAATTTAAACGTTGTTTGTTGTCATGAAATATATATAGGTTTTACAATTATTGTTCTTTCAATGTTTCCTTCAATGAGCTGCATTATGGGTTAATCAAATGTCGTAAACATGCCATTATTTATTTTTGGATAGAAAAATCAGTCTTGTCAGTGGATGCTTTGGAGAAAATGATGGAGGACCCAACAGTGCAGAAGATGGTTTATCCGTAATATCATCTCCCTTCATCTTAATAATGATTATGATTTTGCATTTCTATTTTCTAGTAAATATTCTATTGTGGCTTTGTCAGTACTCAGTATGAATATGGATATGCTTAAACTTCAAGTAGATCCTAGTTTTAAATATATCAAATAAGGTTCCTTAAATGGTAGAAGTCTAATCTTAGAACTGCTGATGACAGTAGTACTGGTATCTGTGATGTTCAGAATTGGGGTTTAAAGTAGTTCCATTGTTTTGAAATATTTAGTTTGGAAGTTTGAAATTTGTATGTGTCTAAAATTCTTCTGGAGCCTGTAATTATTTTTTCCCCATGTCCTCTTGGCACTTTGCTTCTGATGGTTTTAGGGGAAACCTCTACTTCTGTATGCTTTCTTTTCTAACAAATTTTCTTTATCCCCAAAAGTAAGTTTAATTGGTTGGCTGGTACCCTTTTAATTGTTGATAATTTAGTGGAGCTAACTAATGTTCTATACAATAAATTGATTCCCAATGTGTATTTGTTTACGATTACTTGATGAAAATTTACTGATAGGTTTTACTTGATTGTTTAGATTATATAAGAACTTTTCCTCCTTGTTATCCAAAAGAAGTTAAATTTTGGGTTTTATTGCAGCTATTTACCCGAGGAGATGAGGAACCCTACTACCTTCAAATGTAATTCTCTACTTTTAACAGTTTTCTTCTAATCATATTTTTAAGGTTCATTATTTGTGCAGTGTGTAATTTTCATTGTTTAATACCTTCCTGGAAATAAATCATGGGTAACCACAATGCTGAATTTGCTCGTTTTCACGTTTACATGTTCAATATAGTCTATATACAACACTTCTATACAAATATCTTTTTTCATTTGTGAGCAACATCATATAATGGAAGCACTTCTTTACGTAGTTTAAAAATATTTTGTAGGTCAAAAGTCATGTTTTCTTTGAATTGGTAATATTAATTTCTACATGACATCTTTAATATAATTGGCCAATGGGTAATAGTTTCTGTACTGTTGCTGCTTTTAATAGGGATGCTGCAGAATCCACAGTACCGTCAACAACTTGAAGAAATGCTGTAAGTTCTTCATACTGTTTCTTCAGAGGCTCATAGCCTTTTTTGTTTCAGCAATATTATTTCACTGATCATTCATTTGGACAGATAAGCTTCTGGGAAAATGCTTACCTGCATTAAGCTTGCTTGACATATTTCAACAAAAACTAGTTTTGGAGTGAATGAAAAAGTCCTCCACAATTTACTATCCAGTTATTAGTCTCTCATTTCATGAATTTGATGAACTGACAATTTAACCTGATCCTCTTTCTTTATCCTTTATACATTAGACCTCCAATTTGGTATTAGAAAAAAACGATAGCTGTGGATGAAATGTCTGTTTTACTTCTACTTTTGTATTACAGGCTCCCAGCCCTCCCTCTAACCTATGAGGGTGGAAATGCCTGTTCTTACCTCCAGACTTCGTTGTTTTAAGTTTCTTCTCTTTGTCCTTCTCCCCTTGTTCTTATTTAGATTGATTTTTGTTGTTGTTGTTGCTAGGATTTATGTGACTTATAACAACCACATTCCATGTCATTGCATTGACTTGAGATCTTAAGTAGCTGAAAAATGTATTTCTTATTTGTTGTTTCCCTCTCAACCTTCATCCTTTGTTCTGCAGTTAAATAAATATATGCATATTTTGTTAAGATATAAATCTTCTCTGGTGATTCATATATCCTGAATTCTTTCTCTTTTTTCTACATGTTTTATTTCAGAAACAACATGGGTGGAAGCACAGAATGGGACAGCCGAATGATGGATACCTTAAAGAATTTTGACCTTAATAGTCCTGAAGTTAAGCAGCAATTTGGTAAGTTATTATAATATTATTCTTATTCGTCAAGAAGTGCCATGTCTTCTCTGAGAGCAGGATAAACTAAGCAATAGGTTAATTTAACGTAGTTCAATTTTTGCTAGCTACTCTTATTACTTGTTGACTTGAAAATATTCTTAAATACAATGAACTCTTTTCTCGCTTCCATCCTATGGGAGTTATATGTTCTACTTAGTTGATCATCTTGATTCTTGAAGATCAACAGTAATTGATCAGTGACTTTTTTTTCTTCCTGAAGTTGCATTACATATGGGCAATAACTGTATCTTTATTAATAGCTTGTTTTCTTAAATTAATTCCATTTGTCTGGTTACCACTCAATATTCTAATATGGGCAATAACTGTATCTTTATTAATAGCTTGTTTTCTTAAATTAATTCCATTTGTCTTGTTACCACTCAATATTCTAATATTTTCTTAATCAAATCATTGCTGTTAATTCTTCTTTGCGGGTGTGATATACTGTTATTTGTTAAAAAAAATAAAAATAAGAATTTTCAGTGAACTGATAAGTGCCTTGGTAAGTCCCATAGAGAGAAAGCTGTGAGGTTCTCATTCAGAAACACATCACATTTCATGCTGACTTATTAAAGGATACTATTTGGTGTGATTCAGCATTGTTTTTGACTCATTTACATGGTTTGCTATACTTTGCTGACTAATTTATCTTTTTATGCTAGTAGTACATTTCATCAGTTTCTTCTTGTCTCTAGGGTTTAGTTTATTGAATTTTATCTTTGATAATATCATGTATATTTTAAAGAGCCTAAGTATGAATCTTTCTTTCCACTTCTGGTCTTTTCTGAATATATTTTTACTTTCACCAGATCAAATTGGGCTTTCTCCAGAAGAAGTCATTTCAAAGATTATGGCCAATCCTGAGGTTGCAATGGCATTTCAAAATCCTAGAGTTCAAGCAGCTATCATGGATGTATGTTCTAATTTAACTACTTTATTGGTCTTACATATCAAAACTCTGCTCTGATTTCGTCTCTTGTGGCTGCAGTGTTCGCAGAATCCAATGAATATTACTAAATACCAAAATGATAAGGAGGTATGTTGATACTTCTCTGACTTCAATTTTGTCCTACCTGGAGGAGTGTACTTTACCGAGTTTGGTTTTCTCTCCCTTTGTTTCCTGTTTGCTCTTCGGCATGCATAGGTTCTCTAGTTGTAATTAGTTTCAGGTTTGTGTTAGGTGATTTTTTTTTTTTTTTAATGGTTATCAATGAGACTACAGGTGGTGCTGGTGGTTTGAATTTGCATGTTTTACTACGTCTCTAAAGAATAAAGCTATGATATAATTACTCTCTATCAATAATTCCTTATGAATATAAATATTGTCAATTTTTAAAGATTTGGCTGTTGAATGTTTTATCTGATGATTGCTTTTTCCTCTTTAAAACATTGCAGGTTATGGATGTCTTCAATAAAATATCAGAACTCTTCCCTGGAGTAGGTTCACCTTGATGCATTTGTGTGTTGTTGCTGTTTGCTGGGTCAAGTTATGCATCCCTTTCCTGATGACCAATGAAGATTAGCTCGGCCTAACCACACGGAACCATGCAAAACTCTTATGCTCATGGCTGTATACTGCAGGCTCGCGGTTACAGCTCCATTGTGACATGCGGATAGTAACTGTGTCTTCTTCGCTGGGCTAAAATAATGCCACAGTTGGATTTTTTTGTTTCACTAAAATAGTTGAGATTGTGTAGATTGATGTTTTATGTTTTCATTAAATATTTTGTTTGAGGCATTGTATTTGCAGTGTGGTTTTTGGTTGAGTATTCAGGTGCAGTAAAAATGTGAATGCTTCGTATAATAATTTCATAAAGTATAATCTGAGATTAGGGGCCAGTTTGTTTTTTTATTTGTTAAAATAAGCGTTTATTTTAATGAAATAAATAGTTTTCTATTTTTTTA

>Glyma18g05610 ATGACAAAATGTTACGGCTTAGTTTCTTGTGTTAAAAATAGGAAATAATATGTTCTAGAATGGGCTTCTTAGGAATTGTTAGGAATCTGTTTTGGGCTTTTGGCTTGCTGTTAGGTGCTTATATAACAGCTAGGGTGGGTAATATATGACCATATAAACCTATTGTATTGCTCTGAGTAGGAGAGAAGAATGAATTTAGAATTTAATTTTTATTTCCCTTTTTCCTTCTAATTTCTACGGAGTTCCCTTGAAATGAAAAGTACCATGTATCACAAATGTCATCCTTGCTAAATTAATCCACTTAAATATAGTAATAAACAATGTTAAGCATTTTACGTAAGTCTCCCTTCTATTTCTTAAATTATAAACAACAAAGTTGCAGTACAAATCACGATTCAAGTCTACACTCAATCCATTACCTCATTGTACTGGGTCATCATTTCCCAAGTTGATCTAATAAACGATTACTTATTACTATCCTTAAAGAACGGAAATTAATGAATGAAGGCTTTCAGTCTTTTAGTTTTCCAATGCGCATACTTCACTGTACTACTGTGCACGAGGAGACGCTGTAAATGGGAATAATTAAGTCTTGATTTCCGATGACTAAGCATGTGTAATTTCATTTTTTGGAATCAACATGCACACATGTATATAAGTTTGGTTTTAAGTGTTTCAGGTAATTTCATAGTATTTCACAAATACTTCTGTTCGGTGTATCTTAGTCTTCACTGGCTTGAAACCCAATAGCTAAAGAAGATCATGGAATTGCCATAGGAATCGACCTTGGCACGACTTACTCATGTGTTGCAGTATGGCAGGAGCATCACTGTCGAGTGGAGATCATCCACAATGATCAGGGTAACAATACAACTTCTTTTGTTGCTTTCACGGACGACGAAAGGTTGCTAAAAATCAGGCTGCTACCAATCCAGAGAACAATGTCTTTGGTGCATTTTCTAGTCTTAACTACGAGTGTTTTTTTATATTTTTTTCTTATGAATTGGTGTTAACTTTTATTTAAGCCTAAACTTTTATCAGTGCAATGAGTTGACCGCAATTCTTTTAACTTATGCAGATGCTAGGAGATTGATTGGTAGGAAATATAGTGATCCTATATTGTTCAAAAGGACAAGATGTTATGGCCATTTAAGGTTGTTGCTGGTACTCATGACAAACCTATGATTATCCTTAACTACAAGGATGAGGAGAAGCACTTTTGTGCCGAGGAAATATCGTCCATAGTTCTTGCAAAGATGTGGGAGATTGCAGAAGCGTTTTTGGAGAAACGTGTAAAGAATGCAGTGGTCACTGTGCCTGCTTATTTCAATGATTCTCAGCGTAAAGCTACCATAGATTGCTGGTCTCAATGTTATGCGGATAATCAATGAACCCACTCCTGCAGCCATTGCATATGGCCTCAACAGGAGGACTAATAATTGTGTTGGAGAGCGAAAAATTTTCATCTTTGACCTTGGTGGTGGTACTTTTGATGTGTCTCTCCTTACTCATAAGGGTAAGATCTTCCAAGTTAAGGTAACAACTGGAAATGGTCACCTTGGGGGAGAGGAAATCGATAACAGAATGGTGGACTACTTTGTAAAGGAGATCAAAAGGAAAAAAAAAGTAGACATTAGTGGGAACCCAAAAGCGCTAAGGAGGTTAAAAACTGCGTGTGAGAGGTCAAAAAGAATACTCTCATGTGCTGTAGCTACTCACATTGAGACATATGCTTTATCTGATGGCATTGACTTCTGTTCTTCAACCACTCGTGCAAGGTTTGAGGAAATCAATATGGATCTCTTTAAGGAGTGTATGGAGACAGTAGATAAGTGTCTTACTGATGCTGAAATGGACAAGAGCAGTGTACATGATGTTATCCTTGTTGGTGGTTCTTCTAGGATTCCCAAAGTGCAAGAGCTATTGCCAGGCTTTTTCAATGGAAAGGATCTGTGCAGGGAGCATCAACACTGATGAAGCTGTTGCTTATGGTGAAGTTGTGCAGGCTGCTTTGTTGAGCGAAGGCATTAAGAATGTTCTAGACTTGTGCTGATGGATGTTACACCACTGTCACTTGGTATGCTTAAAAAATGAGATATCATGAGGGTAATTATTCCAAGGAATACTACTATTCCTGAAAAGTGATGGAAAACATATCAACAGAATAACAAATTCAATAACAGAGCAATAGCTATCGAAAATAAATTTTCTCAAAATTGTAAGAATTTGTGAAAAATACTAATAAAACATAGAACGCACAATATAAATTAAGAGCACACAAGAGATACAATTTGTTTAATGTGAAAAATCCTTCAATGGAAGATTTAAATCACGGATCGTCCAGAATAAAAAAATAATTTAACTATAATCAATAAAGGTATCAAAAAGTCTCCAACAAGTGTACTAAAATATGCTATAAATAGGCAGATCAAAACAAAACATCAATGCTTACAATAAAAAAATAAGAAGATGAAATTTCAAAATATAGAACAAAGAATGCGAAATACTTATCTCCCTCTCCAGATATCATTTTGACAATTCAATCGGACAATTTTAAGTATTATGCGTGTAGAACCTATTGTCCAAAAATCCGTCCAATCTAATGGTGGACGAGTTGCAATCTTAAAAATGCTCTCGGTGAGTGTGTAAAAAATTACAATGATTTTCGCTCTCCTTTTTCTTTCAATGTTTTGTAGTTGTTTTTCCTCACAAATGAATGTTCACAAGAATGCTTTACAGAGTTTATGATAATCAATCCTCTGTTGGAATTAAGGTTTATGAGGATGAGAGAACAAGAGCCAGTGATAACAATTTGCTGGGTTCTTTTAGTCTTTCTGGCCTTCCTCCAGCTCCTCATGGCCATCCTTTTGATGTGTGTTTTGCCATTGATGAAAATGGTATCCTATCTGTTTCTGCTAAGGAAAAAACCACTGGCAATAGCAATAAAATTGTCATAACCAATGAAAGAGAAAGATTGTCAACGGAAGAAATTAATAGAATGATTTAACAAGCAGGGGAATGCCAAGCTGCATATAAGAAATTCCGTAGGAAGGCCACTGCAATGAATAAATTGGATTATAAAGCATACAAATGGAGAATGCGTTGGAAAATGGGAATTTAAGTTCAAAACTTTGCTCAGAAGACAAGGAGAAGATCAGTTCTGCAATTACAAAGGCCACAAAGTTGCTTGAAGGTGAAAATCAAAATGGTGAAATAGATGTGTTTGAGAATCTCTTTGAACGTGTCATTGGAAAGTTTGATTTTTAA

>Glyma18g11520 AATTGCAAAACCACTTTTTACAAATATGACCCGAAATAAACTCATTATTCTCATATTTGGCGTCGTTGCTCACTTCTGTAAAACCCCCTGCTTCGTGCCATTGGCATAAAGCGAAGATTTCTCTCTCTCTCTCTCTCTCTCACACACACACACACACTCACACAGACTCCCTCTTTGTACTCCATCTTCGTCACCATCCGCACTATGCAATCCCAAATGCTTCTCGATTCCACCATCCGCAAGGTATATTCTTTTCTTCGTTGCTGTTCAATTCAGATTGGCTGAATCCACTTTTTGTTAGCTTATGTGACTTTTTTGTTACATTGAAAACATGCCCCCCTTTTTCCCCCTCAAATTTTCTGGGAAATGGAGGGAAATTCAACGGAAAAATATGATTTTTAGTAATGGGTTTGGGTTAAATTGTTGGTTCTCCCTCCCCCATTTCTCAATTTCCTTTTGCCCCCATTTTTGACTTTTGTTCCTGCTTGCACCTGATGAGCAATCTAAGGAATTTTCAGCAATAGGAGATGGAATTTAGTTTGTTAGTACTTTCATTTTGATGATAAAGGTTTCTCATTTCCATGTGTCTTTTTCAATCCCTTTTGTTTTGTAGTGATTTGCGGTCAAGCCTAGGTTTCTGGGCCGGTTTTGTGGAATCTGTTGAAGTTTTGGATTTTGGGGATGTTGGTTGAGTAGGGTGGGTGAATTGGATTGGACTAGTTGGTTGAAGTTTGGTGCAAGTGAGTTGAGATATGAGTGTGGTGGGGTTTGACATTGGTAATGAGAACTGTGTCATTGCCGTAGTCAGGCAACGAGGCATTGATGTTTTGTTGAATTATGAATCTAAACGCGAAACCCCGGCTGTGGTCTGCTTTGGCGAGAAGCAGCGGATTTTGGGGTCTGCTGGTGCTGCTTCTGCTATGATGCACATCAAGTCCACAATATCTCAAATAAAGAGACTGATAGGAAGGAAGTTTGCGGATCCTGATGTGGAAAAAGAGCTGAAAATGCTCCCTGTTGAAACTTCTGAGGGTCAAGATGGAGGCATTTTGATTCATTTGAAGTACATGGGGGAGATTCATGTATTTACACCTGTTCAATTATTGTCCATGCTCTTTGCTCACTTGAAGACCATGACCGAGAAAGATTTGGAGATGCTCATTTCGGATTGTGTTATCGGGATCCCATCATACTTTACCGACTTGCAGAGACGGGCGTATCTTGATGCAGCGAAAATTGCCGGGTTGAAGCCTTTGAGATTGATCCATGATTGTACTGCAACTGCCCTTAGTTATGGAATGTATAAAAAAGATTTTGGAAGTGCAGGTCCAGTAAATGTTGCATTTATTGATATTGGTCACTGTGATACTCAGGTCTCAATTGCGTCATTTGAGTTTGGGAAAATGAAGATACTTTCACATGCGTTTGACAGGAGCTTAGGGGGGAGGGACTTTGATGAGGTTATATTTAGTCATTTTGCAGCAAAATTCAAGGAAGAGTACCACATTGACGTGTATTCTAATACCAAGGCATGCTTTAGGCTACGTGCAGCATGTGAGAAATTGAAGAAAGTTTTGAGTGCAAATCTTGAGGCGCCTCTAAATATCGAGTGTTTGATGGATGAGAAAGATGTCAAGGGATTTATCACAAGGGAAGAATTTGAGAAGCTGGCATCAGGATTACTGGAGAGAGTTTCTATTCCTTGCCGCAGAGCATTAATTGATGCAAACTTGACAGAAGAGAAGATTTCTTCTGTAGAGCTAGTTGGTTCGGGTTCTAGGATTCCAGCAATAAGTACATTACTAACTTCTCTGTTCAAGAGAGAACCCAGCCGACAGCTGAATGCAAGTGAGTGTGTAGCCCGTGGTTGTGCTCTACAGTGTGCAATGCTCAGTCCTATTTACCGTGTGAGAGAATACGAGGTTTGTGTATTTATTTTTCCAAATCTATTCTCATCTTATCTTTATGCTATAAATATATATTGCTGATTAGAATTGAGTTTGTAGATCCTTAGAAATGTAGTAAATTAGTAATTGTGAAAGGATCAAATTGTTACCTAGGTTTATTCTAACAAGTTTTGTTTTCAACCTTTGATTGTCTTTGATAGTTCCATTTTTTTTTCTCTTTTTCTGATTTAAAATGAAATTTCCATAACTCATTGGTATAGACTCATGTGCCTCTCTTTGTCATATTGTTTTCCATATAATTAATGTGATTTCTCATGTCTGTTCAGGTCAAGGATGTTATTCCCTTTTCAATTGGACTTTCATCAGATGAAGGTCCAGTTGCTGTGAGATCAAATGGTGTACTTTTCCCAAGAGGCCAACCCTTTCCAAGTGTTAAAGTCATAACCTTTCGGCGAAGTGATTTGTTTCATTTGGAAGCTTTCTATGCTAATCCAGATGAACTACCACCTGGGACATCTCCTATAATTAGTTGTGTCACGGTATGCCCACTTTAGAATATTCAGAAAACGTGAAGCAGTTAAAATTATCTTGTGCATTTTTATCTGTTATATATATTTTCTCGTCAACCACGCTACCCCATTTCTTAGTAATAGTTACATCCTCACTCTCCTGGTTTTCCTGTGTGTTTAATGCAAATCATGCACTATAGCTTTAGTGTGAGCACTCTTTTTTGGGGGGTAAATCTATGTGAACTTTTTAACATTCTTTATTTTACTTTTTGGTACTCAAATATTCATATATATATGTGCTAAAATTTAAATTCCACAGAAAATTTGTTTTTGGTAGATATTCATCATTACCACAATCAACACCTTTTCTTTTTGAGTTGTAATATTCTGATATTTAGAAGCTGTTAATGGAATATCTCCAAACGTTTTGTCTGACTCTTGTCAGTTATTGGCATCAAGTGAGAAGAATTACTTCAATGGATCCGGAGCTGTTGCAATAGACACCTCACACTATTTCTCAATGAGAGATAAACTTCATTACCAAATGGCCTGTGACTGTGACTATTACTTGTTTTGACATAAAAGGAAACCATTGTTGTAAAACTAAGTAGGCATAACAATATGAAAGTCAGAATCTGTTTTCTGTGGATATGCACACACTTGTCTAAATCATTATGTTAGGAGAAAGAAGAAGAGAATGTGGTAGATCTAACATCATTCTTTGTGCTGGTAATTCAGCAGGCATTATCTGCATGCCCTCACCTTGAAATTTGCTTATGCATTTGATCACTACTTCATTTCAGATTGGTCCTTTCCATGGATCCCACGGTAGTAAGATCAGAGTTAAAGTTAGAGTTCCACTTGATCTGCATGGCATTGTCAGTATTGAATCAGCTACAGTAAGTAAGAAGCCTCATTTTATCTTTCCATACTGTGCTATTAGTTTTTCTAAGAGAGGTCTTTGGACATAAGCCTTTCTATGGTTTCAGTTGATCAAGGATGATTCGGTTATGGCTGGTGATTATCATTCAAATTCTGATGCAATGGATATTGATCCTATTTCTGAGACAGTTACCAATGGGTTTGAAGATAATACCAATAAGAACTTGGAATCTCCATGTAGTTCTGTAATTTCCCTTTCTCTTTATCTCGCTTTCATTTTTCCTTTCTGGGAAATTTTCAGATTTCAGGCAATTTTATCCTTTGAGCATGGAAAATCATATAGCTAAAATGATAATAGCAATTGCCATAATTTCTGATGAATTCTGTGACGTTTGTCATGGTTGATTTTCATAAAGTCCATTTGTTGTTTGGAGTAAATTTATTAGGTTTAGTTACCCTTTTGGTCCCTATAGTTGCAATTTTTTTAACCCTTTGGTCCTTGCTGTCTATAATTGCCTAAAGTCGTTACATCTAAATCTTTTAAAACTGCTACAAAACTAAAACTTTCACTACCAAAAAATTTAGACAGTTCCAGTTTTGTAGTAGTTTTCAAGGATCTGGTGTAAAGGGTTACAAAATTGCAACTATGACCAAAAGGGTAATTAAACCATTTTATTATTACTATCATCCCTTTTTTTCAAAGGCTGACTGATTCTGGTGATATAATATTATTGTGGTGATAATTATGATTGCAAATATGATTCATTTTGGTATTTGGAGTTATTAAAATATTTCTATCATACCTTTTTACAGGCTGATGGTACAAGAAAAGATAACAGAAGGCTTAATGTGCCAGTGAATGAGAATGTCTATGGTGGAATGACAAAGGCAGAGATCTCAGAAGCTCGTGAAAAAGAACTCCAGTTGGCCCATCAGGACAGAATTGTAGAGCAAACCAAAGAAAAGAAGAACAGCTTGGAGTCTTATGTCTATGATATGAGGAGTAAGGTACAGATTCAATGTCTCAAAAATTTGTAGACATGACTGGCTTCTTTGATGAAGGGATGTTTGGTTTTTATGTGGTCACACAGTAAGCATAAGCTATAAATTGTTTTGTCCATTGGCTGATCCTTTTTTTTTTCAAAATGGAAAACAGACATGTTCCTTTTTAAAGGATCAGATTTACCTTTGTTAAGTTAATTAAAAGTCTTGAGTGGGTCATATGAGAAACTTTATAGTGTAGTTATTGAAAATGGATTATGGCTTTCTGTGGTGATTCATTTGTAAATGTAAGTTCGCAATTGGTTTTTTTGTAAACTTATCTGTGCAGGGTTGGAAATTTGAAATCTTTAAATTTGTATCCCTTTCAATGCTACTGACTTTATACTGACATTGGAAGATTATGTTTTTGGTTCTATTTTGCTTTGTGAGCTGAAATTTAACTTATTTGCATATTTGAATAGCTCTTCCACACATACCGAAGCTTTGCAAGTGAACAAGAGAAGGATGACATATCTAGGACCCTTCAAGAGACTGAGGAATGGCTTTATGAGGATGGTGTTGATGAAACTGAACATGCTTATTCTTCAAAACTGGAAGATCTGAAAAAGGTTATTTTTTCTATTCTTTTCCTTTTATTACTGAATCCCTTGCCGTACTTCTTGTTTACAATCTATGTATTCTTGTAGCTGGTAGATCCAATTGAGAATCGGTACAAAGATGATAAAGAAAGAGTGCAAGCTACAAGAGATTTATCGAAGTGCATTTTAAAGCATCGTGCTTCTGCAGATTCCCTTCCAACCCAGGATAAAGAACTGGTACAATAAGATTTAGGCTAGTATATGCTTTTGTGATTATGATGCTGCCCTTAAGATCAGCTTTCATGTGATGATTTTATTGTTTAGATCATCAATGAGTGCAATAAAGTGGAGCAGTGGTTGGAAGAGAAGATCCAGCAACAAGAATCATTTCCTAGAAATACTGATCCAATATTATGGTCAAGTGATATCAAGAGCAAGACAGAGGAGTTAAACTTGTATGACTTTCTGTTTTCCTTTTCAGAAGTGTAAATTTATTGTTTTTATTGAATCCATGGGACACAAATAAAACTTGAGTCTAGAGAACAACTATTCATAAAATCATAGTGTTAGGCAATCAAATCTATTGATTCATGTGTAATAAAGTTTCACATATGATATCAATAGATTCATAGGGAATAATATTTCTTATGATAATAATTTTGAATTATGACAAAACTCAGATACAATTTTTTAGGTACTCTTGATGTACTTCAATCAGAATTAGTTTCATGTTAGCATTTAATGCAAATATTTATTACGCAGATTACCAAGGTGAAACTCTAATTTTGATTGGAAAATAGCACCAAAAACACCAAAGGACTGCATCTATGTTTTGTCCATTGAATTATGCACTTGCTTGTGCAAAACATTGTTCATACGGTCTTGTAAACATGTTTGTCTTTTTCCCTTTGATTTTATCTCAACATTAACAAATGTCCTATTTTGATGGTTTTCAGAAAATGCCAACAGATATTGGGATCTAAGGCTTCTCCATCTCCAGAAGACAAAGACAAGCCGGATACATTCAATGATCCCTGAATACATTGATGTGATTATGCCACATGGAACTGAAGACCCAAGTTTTCATCACTGATTCCATCTGACAAATATAGAAAAAAGTTTCCTTGCATTTTTGCTTTGCAGCGTTTTGTATTTTAATAATCTATCTAATCTTTTCTGGTATGATATAGTTTCTTCAAATGAATTGAATAGATTCTGCTTTAGGGAAGAAAAAAATGTGTGAAGTATTAGTGGCACTTGTTTCTGCTTTGTGTTCAAAGAAATTCTTACAAAATTGATTGGTCAATCTCGAATAATGTTGGAATCTGTTGTGAAGCTTTTAGCATATAAGAACGTGTTACGCGGCTTTATGATAATGGATGAATTAAATTATTATTATTGTTAGGCTGGCCACAATTT

>Glyma18g13077 CTCTCTTATCATACAGCTGTTACTCTTTTTCCTTGATATTCTCGCTTTCTCTCTCTCGGGTAGCGCACTGGTTGCGCTTCTTCATTGCGGCACTGAGTGAGGCGGTTCACTCTTGCTGCCTCTTTCTCTTCTCTGTCCCTAGAAACAAACCATCATCTTCTTAGATCTATACAAAAAGCAAAACCCTAAACACTTCCTTGCCTTGTTTCGGTTCCTCTTCCAATTTGATCTCATCATCAATGGCTTTCGCATGCAGCAGAATCGCGCAACGAACATCAATTTCATCTATAAAATCAGCCATCAAATCCAACATTCGCGCTTCTTCATTCTCCAAACCAGCTTCCTCCTTTTCTCCTATTTGCCAATCCTTATTGGCCAGGTTTCCCTTCGTCCTACGATGTTCTTTTTGCTCCTAATCACAAACAAGCCTTAGAATTATCGATTTGTGGTTGTTGTCGTTGGTCTGTTGCAGGATTTTGCAGGAGCTGAGATGCGTGCAGTCGATGTTGCCGCTGCACAGCACGGTGGCGGCGGCGAGAATGATGTCGTGTCTGACTTTCAAAAGTTGTCGAGCGCTTTCACCGGGTACTCTCTGCTGCACCTCTCCTAGCCTCTAATACTCTCCTTTTCAAAAGTATTCGCAAACCATGAGTTGCTTCTCGGCTTGGGTTTAGTTTTTCTATGGATTTTCTTTTTTCTCTTCCCGCTTTCGAATAAGCTTTGTGAATTGTGAGTGCACTGCTAAAATTAAGAAGAGAAAACTAATACACTTAGTCTATCTATTTATTTAATTAGGACCCATGCTTTGGTATTTTTCTTAGGCAACTAGCAGTTTTTCATTTGAGTTTAATTGGCGAATTTGGGATTTTCAAAATCATTTAAAAAGCTTCACTCTATTTGATTTTGCTTTCTAAATTTGTGCACCATTTAGCCTCTTGATAATACTGTTGTGTTAATTGTCGATACAAGTAGAAGATAATTTCCCAATCTATATCAGAGGTGTTCTGCTGTTTCTCCAACCTCTTGGCTATCTCAGACTGGTAGAAGCTTTCCTTTAGGTCTTCCTCATAGTATGTATTGATTAACTGCTTCACCTTCCTCATAGTATGTATTAAAAAGTCTTTTTAACAAATAAATTGGTGATGCACCCTCAGGTCTTGAAGTGAGAGACAATGCAATATCCATGGATGCAATTTCTGCTACTATCATATCTTATAATTTTTGGCCTCCAATATAGGTTTGCAGACAAGATTCGTGTGAAAATAAACTTAGGACTGCTTGTGTAATTGTTGTTGCAAATGCTTGTGTTTTACCTACTACTACTACTACTACTATTATTATTATTAACAAAATTTTGACAGAGTTCTTTAAAAAGGAGCCTAGGTGGACTATGTGTGCAATTCTCAGCCCAACTTTTAAAGTACGGGAGTTTCAGGTAAAATTTTTCTGCCATTGTAATGTTTTAAAATTACTCAATATAAAACTATTCAGATCTTTTTAGGAATCCCGGACTTCAGCATTTGTTTTTTTATTTTATTTTTTTGGTATATGTTATGTACCTTGAAGTTTGAATTACTTGTCTCTGGTATCTCCCTTTGCTTTAGAAATTGTGCTCGTTAACTACTATTTTGTTGTGGTGAATTGTTTGTACGTGTTAGTATTATATTTGGCATGCATGTTTCAAGACCACTAGGATTTTCCACCTCACCAATTGAGATTGATCCATAATCAGGAAGTTTGTGACAACCAGGCCAAGTTTCCTCATCAATGTTCCCCAAAACACTTAAAATTCTGCTGACTCTGAAATATTCTTCATGACTCAAGCTTACCTGATTTGGATTTTCATATGCAGTTTGGTTTAAGTTAACTAATTGAGAAATAGATTCAGGATGTTGGGTTGAGCTTTCATCATATAATAAACTAGGAATAGTTTTGAAGCATAACTTTTCCTTTTCCGCTGTTTGGGTAAAATGACCATGATTGTTAGTTATTAGTGACGAATGTAATTCCTGTTACATGGTTTGTGACAATCATTGTGTTCCTCTCTTGAGCATCTTCCAGTCCAATTATTTGATGTGTAGTCATGGTATCAAAATCTATTAACTAATAGACCATTCTCTGTAAGGCTTCTTTTTTACCTTGGACCTTGGTGTAACTGGTTTTTGAAAAATACTTGTTTAGGATAATCTTGTGTCACATAGCTTGGACTAATCCATTTCATTATTAGTAGAGCTCCTTTGACATGATCATATCCTATACTTCATGTTAATTACTTTCTATTTCTACGTAATTTATCGCATTATAAGTGTATGCACCTCTTCCTTTAATTACACTCTATAATCAGAATATGAGATTGATAACTGATGTATTCTTTGAAGGAAGTGAGCTTTGTAAATTTATGCTTGAAATAGTGGTCATCACACCAGTTTGGTTGGTGTCGGTACTTTAGACATAGCTAGAGTGGGCCAAAAATGCAAGACTTACTTTTCTTAACTTTTTTTAGTGTATGAGAATGATGGGTCTTGAGTGGAAGGATATTGCTTTTGTTCAGAAGTCAAATTTGGAGAGTTATATACAATGAATTACAGATTTCCTTGGTCCGCATGAAAGGTACGTTTCATTCCTGCTAATATTTTTTCTTGTCAGCTTGTTTTCTTTACCTTCATTTGAATTTTTTGTGGCTTTATTTTACTTTTTGTGGTTAGATCTTTTTCTTAAAACTGAAATCATGGGATGAGTTCTAAAAAACACGAATTATCTAAGGTTATTTTATATTAAAGAAAGTTATGAAAAAGATTAAAAAAATATCGTTGGGGTGAAATGTGAGAGCCCAAACACAATTCGCGTTGAGCCTGAAGGGCCCACGAAGAGCTGTGTGTGGCCTTTGGTAGATACTAATGGACTGTTTTGAACTATATCCAAGCTTAATATATCTAAATATTTATAACACAATGTTTTGTAACTTTTGTAGAATTGATTAGCTTAAATGGCTGGTCCTAGGATTGTCCATGCTATCTTGAAAGGTCCGAGTGTGGTTAAGGACATCATAATTGGAATAACACTTGGCTTAGCTGCTGGTGGTGTGTGGAAGATGCACCACTGGAATAATATATATATATATATATATATATATATATATATATATATATATAAAAGTCATAATAACAATGTATTAAATTGAAATTTTATTAAAATATTTATTTTAAAAATAAGTAAAAAAAATGTGTATGTGACTGGGGGCAGGGTGTTTATAAAAATGGTCCTATTTTTGTTTAAGAAAACTATTATTTAAAAATTATTGTTATTATTTAAAGGAAAAATGGTCCTAAATCACATTTACCCTGCTAGTTTCTTTACACTATTTCATCCAATTTCACTCTTTTTTCGTTAAAAATGTTATTACTTTTCAAATATCCTAAATCATCTCCTTCATGATTAGAACAAATTTAATTTCCAAGTTAATAATAATAATAATAATAATAATTATAAAGAGAAACCTACTAAGATCAGTTTAGCGACGGAGGTTTGAATAATTCCTATGATGTATCATGTTCTAACTTCATCACTGCCTCTGTACAACAATAAAAATTAGAACAATGTCAAAGAGTAAAAATATTTCATTTACACTGATATCCAATAATTGAGTCTTATAATGCAAATGTTTTGTTACAATGGTCATAATCACATTATTGTAGTCAATAGTACTAATAGCCTAGTTTATTTATTTGCTTTCACACCTCTGTTATGTTGATGCTCAACCCTTAAAACAGCAGTAATAGGCTTTTTCTATCTTTAGAAAAAGCAACAAAGTTTAAAATTTTTCTTATGAGCACTTGTTTTGAAAGAAAAGAAAATTAAAGAAAATAGATTGAAAATATTTTCTTAATAATTAATTGCTAACAATATATAATTTTAAAAAATGTTATATGGAAGGACTACATGTGCTTAAAAGTTAATAGATTATTTACTTAGTTAACTTTTGTTTTCCTATTTTATCTCCAATAATTAATTGATTAATATAAAAAAACACTACGTACAAATAAGTCTATGAATTTAATTTAAATACTATTAAAGGTTCTATAGTGGACATTAAGAATGGAAATAAGTTGGCTATTAGGAGATTATAGCACGGCTTATATATAATGAGGTCTAACGTACATGACTTATTTATTAAATAGCTAGATGAAACTTAGCTCTTGAAAAAAGTCTATAAAGTATGATAGTCCATTCGAGTTGTTTATACAATATTTATAAATATAATTATTAATAAACTTACTTAAGTCAATAAACATATCAACTTATTTATCGGTCTTATCATAAATTAGATTTTTAAATTGACTTACAAGTTAAGTAAATTTTATACATGTGAGGCCTTTAAAAAAGACTTACAACATTGTAGCCTTGGTGCATTTCATTTTTCGTAAGCTAATTTAAAAAAGAATTGTTATTTATAAATAAATAGAGTTTTAGAAAAATGATAAGATTTTATAAATAAATAAATAAGCAGATATAATTATTAATTAAAATAATAATTTGAGAGAAAATAAAAAGGGTATTTATTTATTTGTTTGATAGAGAATAAAATAGAGTTTATTTTTATAAAATAATAAATAAATAAATAGACTAAACCTAGCTATAAATAGCGTTAGGTCAGTTTTCATACTGACGGTATCTCTTCTTCCCCTCATTTTCATTTTTCCCCTTCTCCTCTCAAAACTCTTTCTTTTTCTCGCAGCCCACCAAACCTATCTCATAAAAATGACGATCTCGAATTCATTCACCGTTGGATCGTCGTGAAATTTGAGCACCCTGTTCCCAACCCAATTCCGAGGATTCTCATCGTTGGGAATTTCAAAATCATGTCTGAGCTTAGAGTAAAACCCTTTGCACCGTAGCTCTCTCTTTTCCCGTAGAAACCCAAAACGGTCTCAGTAAAACTACGATCCCGGTTTCGTTAACCATTGGATTTTCATGAAATTTTGATATGTGGTTTGCGATTCAATTCCACACACCTTCCCTATTGGGATTTATGAGATAATATTCATGGAGAGAGAAAAAGGAATCACACGAAGACAATACATATGGAGGCTTCAATCCCTTCTCCGTCTCGCTAACGTTTGGGAACTCTATCGGAGCAGTCGGAGGAAAAACTGGAGGAATCTCAGGAAACCGCTAGAGATGTTGTTATCGCTGTCGGAAGACACGTGAGTCCGCTTAGAGGTAAGGGATGAGTTATTCACAATTGGGGGTTAGTGAGAACATGTGTAGGGATCCTTAGAGGATTAAATTGGGGTTTTATTTTGGGATGTTTATTAAATTGCAATTTTTCCTTTATGATTACATATAAAATATTGATGTTCTGATGAGAGTTGCTTGATAAATTGTGCTCTTGATATTTGTATATTTTGACCTATGATTTTGATATAATTGTGTAATATTATTTGGGGGGTTTTAGTCCCCAGGTTTTGATAGTCTTTTGTATTAATTGTTATATTGAGGATATGAAATGATAATTCAAATTGTGAGTATGTGGTGAATTGAACATGTGATTAATGGTGGAATACATGTATATTGAGATGTGTATTGTGTTGTGAGCTATAAATTGTGCAATCACATAACCGTAACACCCTTTAAGGGCGACGAGTTTTGCACGACGAGTATTGTGATGGGATCCATTGTGGGAACCCGACGAGTTTAATCACAAGCACGATGAGTTAAAATGATTTTGAAAATAATTTAGTAGTGTCTGTATTGTATAGTTCATATGTAAAGTGAATATGATTCATGAGGTGTGATAACATGCTATTTTGATATTATACCATTGTAATTGAGATCGAGTGTATGTGATAAACTGAGTATGCACGTGATTGAGATGTTGTGTGCATTGAGTTATAAACTGTTGATTGTACAATCACACGACTATAAGACCCTTTAAGGGCGACGAGTTAATGCTAAGACCCTTTAAGGGTGACGAGTTTATGCTAAGTCCTTTTAAGGGCGATGAGTTAATGCTAAGACCTTTTAAGGGCGACGAGTTAATGCTAAGACCCTTAAAGGGCGGCGAGTTAAAAATATTTGAGAACAATTGAGGAGTCGTGTGTTTTGTACAGTTCATAGATAGAGTCTGTGTGCTAAAATGTTTTCTGGGTTGGACCTGAATCAGGAGGGAAAGGCCTTGACGGACTCTTCGGAGTGTAGGCCTTGGGGGTCATCCGGTTTGAGTGCTCCTTTAAGCCTATATTGATCCCATATGGTTGGAGCATTCTCGCAAAACACTGTGACCCTGACTGGTCTCCCTATGATATTACCTAGTGAGAGTGACTTGACTTGCTAATGTGTGGCTTGTCTTGTCACGTACTCCTAGGCACCTGACGAGGTTTTTCACTGATATGGTACCACATTACATATAGGATTGAGTCTTAGTGTATTTGTTGCATAACGCTTGTGTATTGTTCTTTATTGATTGATTTGATGATATTGTGTTTTGACTATTGAGTATGCGAATGTTGTGAAAACGAATGAGACGTGTGGTAAAATAACGTGAGTTATGCTCAAGTGAATTGTATTTTGTTATATAATATTTATACCTATACGTTGTCTTATTTTTCTCTATTAGTTAGGAATGTGATAATTCACTCCCTCGGTTATTTTTGTTTGGATCCTGTGATGATCTTGAACTTTGTGTTCGGGGGAGCAGATGACTAGGTGAGTTGCTTTAAGGAACGTTGTGCTAAAGGACGTCGAGACACAATGCTCTGATAGGATGTGACATTAGGGTATAAGTTGTTATATTAATTGTATGAGGTCTTAGACAACCTTGTTTTGAGCCGAAATAATTTATTAATTATTTGAACAAGTTTTGTTATGATGTTAGAAAGGTGAATGTGAGCCTTCTACCCTCTTGAAAAGCTTGTATTTAAATGTGTTTTAAAAACTTTTGATTAAGTTTGATTTCTTATTTCTTTTATTATTAGTACAAATATGTATGGGGTAGAGGGTGTCACAAGTGCTGCTCATTTGCTATGATATATCTCTTCTAGTAAATATTTAATAGCTACCTCATTGTTATTGTTAAGTATATTAGGACTAAGCGGTGAAGGCAGCATTTGAGAGTTTTTGAGCAACACGTTGAAAGGGGTTTGGATGAACACGTTTGTGACATGTGTCATTCAGCATTTGCTGAGTAAGAACTCAAAGAATACATGTTTATTCTGTATTAGTATTTGACTAAAAAAAGTATTAAATTAAGAAATTAACTAAAAAGAGTTAGTTCTACACAAGTATCAATAGTTTACTCTCAATTTTATAAACCTAAAACTCCATGCAAATTCTACCCAAACCTCCTATGTGATATATGTTTTAAGGGTATTGTTAGGTACACCCAGCATTATTGTTGGTGCACCCAGCAAATTTTCTAAATGCCCAAAGTACCCCTTCCTCTGCTGCTCCTGCATTCCGTCTTGTCGATGAATAGTGCCGCCATGAACAGTGCTTTGCACCAGGCACCAAGCTTTTTAATTTAAACGGCACAATGCTTCATTTGTGCATTCTTTCGCTGTTTTCTCTCCTCCGTGCACAGTGCTTCTTCCCCGTAATTCTTTTGTTTCGTTGCGATCGTTTTGGTTCGGTGAACGGTTAGGTTCCGTTAAGGTGAAGGTGAAGGCGTTGCGGTGGTCGGTGGCGACAGACGAGGTACGCAGTGTTGGGTGCGTTCTGTTCTGGTGTATGGCAAATCGTGCGGATCAAGTTGATCCGTAAGAAGTTTACGGATCAACTTGATCCGCAGTTATATTTCATTGTTGGGGATCAAGTTGATAGTTGCGGATCAAGTGGATCCGTGAGTCTTTTGCAGATCAAGTTAATTCGCAAGTTGCTCGCGGATCAAGTTGATCCGCAAGTTTTTAGCAGATCAAGTTGATCCACCAGTTGCTCGCGAATCAACTTGATCCGCTAGAAACTTGCGGATCAACTTGATCCACGAGAAACTTGCGGATCAATATCCTCCATAACTAATTTGCGGATCAACTATAACATATGCGGATCACATTACAACACACCCGGATCAAACAACGTCAATTGGGTACATAGTAATCACTTTAAATGCTCATGGGGTATCTTTGTTTTTTCGCTATGGGTGCTAGGTGCCCCAACAATAATGCTGGGTGCACCTAGCAACACCCATGTTTTAAACATGAAAAAGGGAACGAGAGACCCAATTTTCTATACCCGTGCTAATATCAGGTATGACTAACATTGATAATCTCTATCATTGTCTGCAGGTTCAAGGAATGGGGATTTCATAAAGGTTTGGGCAACACTACTCTGGTTTTGGAAAAAGTATGACATCTTTTTTTTTTCTGGTTTTGGAACACTACTCTTTATTGAATAACTTCAATGAGGCTGAGGCTTTTTTTTTTAAACAGGTGGTATATATTCTTGATCAAGTAAGGGCCTTAGAGGAAGAGATACTCCATAAGATTGAGCTGCAAGGCTTGGTGTTATTTTGTCTTTTAAGGTTTGTGTCCTTGTACTATCTATACCATGCTAATAGCCATCATATCTTCCTTCTTGTTTCTCATTTGTAAATATTCAAATTAATAAATGTATTTAAATATATAAATTATATGTTGGATTTGTCATGAATAGTTTAAATTGTGATGCAAAGTTATTTTTAACTATAGGCAATTTCTTTTAAAAATGCCTATTGAAATTCTATTTTGATATTGTGATAAGAGTAAATTTGCAATTTTTTTTTGCTTATGTTGTATATATATGAATCTAATGATTTACTTTCATTTTCTTGACTGAGTTAGTGTATCTAATAAGTGTTTGAAATTCACTTTGCTTCTTGTCTATTTAATTATTTCAATTAATCATTGTGAGATTGAAATATAGAGAATAAAAACTAACTTCCATAAACCAGCAGCCACTCGAGAAAAGATAAATGCTAAAGTTGTTGGTGTTCTTTGGATCGAACCTCAAGGAAGAGCAAGAAGAAGGAAAAGGAACCTATGGAAAAGCCTCCGAGCAAGTCACAAATTAAGAAAAGGCCTTCTATGTGGTAATTTCCGAACCACAATTTGGGTTAAGATTATTTATTTGATACAAGTTGTTTGGTTTTTTTTCCACTAGGAGTTTACAATTTTATTTCTCCCATTGTATTTAAATAAGATATATAATGCCCTTTCATGCTACTGTGCATGTTATAAATAAGTTCCTCACTATCTTGATCTCTCATGTAGGTTCATAGTGTATGGGCAGCAGTAGAAGCTTATCCAGCCCCTTCTATTGTTCTACATCACATTCACATGATGTACTTCATGTTTTTGACGATTTTAGGGAGGCTTATGCTTGGCTGATTCATAATATAGAAAAAAAATGATAAAGTAAGTTCTATAGGATTTCATTATTTCTTTGTTGAAATGGATTTGTGGTGTCTTGTGTACAACTTTTTTATTGATGTTGCCAACTTTGATTATCTTTGCCTTTTTCTCTCACCAAGTGCAGAATTTAGGCTAATTACTGATATTCTTTCTGAAAAATATTGTTGGAACTTGGAATTAATGGTGATAGTTATCTTGCATTATTTCTTGTGTTTATACTAATTCCTGAAATTTACATCATGTTTCGAAATT

>Glyma18g52470 ATGGCAACAAATGGCAAGACACCTGCGATAGGAATCGATTTGGGCACGACATACTCATGCGTTGCAGTGTGGCGGCATGATCGAGTGGAGATCATCGTGAACGACCAAGGAAACAGAACAACACCCTCTTATGTTGCTTTCAATAACACCCAAAGGATGATTGGTGATGCTGCCAAGAACCAGGCTGCTACCAATCCAACCAACACTAGCACACCGGTGATAGGGATCGATCTAGGCACGACATACTCGTGCGTTGCAGTGTGGCAACACGACCGTGTGGTGATCATCACGAACGACCAAGGGAACAGAACAACACCCTCTTGTGTTGCCTTCAAAAACACCCAAAGGATGATCGGTGATGCTGCTATAAATCAAGCTGCTGCCAATCCAACCAATACTGTCTTTGGTGCTAAGCGGCTAATTGGTAGGAGATTTAGTAATCCAGAGGTTCAAAGTGATATGAAGCAATGGCCATTCAAAGTCATTGCTGATGTTAATGACAAACCAATGATCGCTGTTAATTACAATTGTGAGGAAAGGCACTTTTCTGCAGAAGAAATTTCGTCCATGGTTTTGGAAAAAATGCGAGCGATTGCAGAGTCATTCCTTGGATCAACAGTGAAGAATGCTGTTATCACTGTGCCAGCTTACTTCAATGACTCTCAGCGACAAGCTACCAAAGATGCTGGTGCCATTGCTGGCCTCAATGTTTTGAGAATCATCAATGAGCCAACTGCTGCGGCAATTGCATATCGGCTTGAAAGGAAAAATTGTAATAATGAAAGAAGGAATGTTTTTGTGTTTGATCTTGGTGGTGGTACTTTGGATGTGTCTCTTCTTGTTTTTGAGAAGGATTATATCCGAGTTAAGGCAACATCTGGAGACACTCACCTCGGAGGAGAGGACTTCGATAACAATATGGTGACTTACTGTGTGAAAGAGTTTCAGAGAAAGAATAAAAAGGACATTAGTGGAAACGAAAGAGCCCTTAGGAGGTTGAGGACTGCTTGTGAGAAAGCAAAGAGAATACTTTCATCCACTGTAATGACCACCATTGAGGTAGACTCTTTGTATGATGGTATTGATTTCCACTCATCAATAAGTCGCGCAAAGTTTGAGGAACTCAACATGGACTACCTTAACAAGTGTATGGAGTTTGTAGAGAAGTGTCTGATAGATGCTAAGATGGACAAGAGTAGTGTTCATGATGTTGTCCTCGCAGGTGGATCTACTAGGATTCCCAAATTGCAGCAACTATTAAGTGACTTCTTTGATGGGAAGGATCTCTGCAAATGCATCAATGCTGATGAGGCCGTTGCATATGGTGCTGCTGTCCATGCTTCTATGCTTAATGGTGAGTCCAGTGAGAAGGTTCAAAACACTTTACCGAGGGAAGTCACTCCTCTTTCCCTTGGGTTGGAAAAAGAAGGAGGTATCATGAAAGTAATCATTCCTAGGAATACTAGCATTCCTACAAAGATGGAAGATGTATTCACAACACATTTGGATAACCAAATCAATATCTTGATTCATGTTTATGAGGGTGAGAGGCAAAGAACTAGAGACAACAACTTGTTGGGCAAGTTTGTGCTAGAAATTCCTCCAGTTCCGCGTGGTGTTCCTCAAATAATCGTTTGCTTTGAAGTTGATGATGAGGGTATCTTGCACGTCTCTGCCAAGGAGAATTCCTTGGGAATAACCAAGAAGGTGACCATAATAAATGACAAAGGAAGGCTTTCTGAGGAAGAAATTAAGAGGATGATATCAGAAGCAGAGAGGTACAAAGCTGAAGATGAGATGTATAGGAAGAAGGTAGAAGCAAGGTATGCATTGGAGAAGTACGCATACAACATAAGGAATGCTATAAAGCATAAGGGGATTAGTTTGAAGCTTTCTCCGGAAGACAAGGAAAAGATCAATGATGCAGTTGATCGTGCCTTAGAATGGCTTGAGGTCAGTGTGGATGCTGAAAAAGAAGATGTTGACAATTTCCGGGGCAATCTTTCTAGCGTTTTTGATACAATCATGGTTAAAATGATAAAGGGTGAGGATAATGGTGCGCCTCCAGAATCGCTGGTTATCAATATTGGCAAAATTTGGTCTCCAGGCAGTGTATTCAGCTGTTACAGGTGATATCATTGGATTTGTTTCCGTGATTGTTGACTGTTTGGCAAATTAGCTATTATTTATCATTTTGAAGATAGAAATTAAATCAATAATAAGAAAACATGAAAGTTCATTTGTAATGCAGAGAATTGAGTTGAGAATCTTTTACTAGCATTTGTAGAAATCCAATTGTCATCTATTCTAG

>Glyma18g52471 ATTGAATGACATCATATTGTGAAGTCATGCGTTGCAGTGATTCATCATATTGAAAGTGATTCAATAAAAATTGAACTTGTAAAGTACTTTGTTCTCATATTGATTATTGCAACTTCTTGTCCAAGCTCCATTGTCTTGCTAGGAAGAGAAAAATGGCAACAAATGGCAAGACACCTGCGATAGGAATCGATTTGGGCACGACATACTCATGCGTTGCAGTGTGGCGGCATGATCGAGTGGAGATCATCGTGAACGACCAAGGAAACAGAACAACACCCTCTTATGTTGCTTTCAATAACACCCAAAGGATGATTGGTGATGCTGCCAAGAACCAGGCTGCTACCAATCCAACCAACACTGTCTTTGGAAAAATACTAAACCCTTTAGCTTAA

>Glyma18g52480 GGATTGTTTAATATATTATCTTGTGAAAGTGATGTACCACGCGAAGATTTGATTCCAAGTACAGTATATCTTTTTACATAGGTATCTTGTAAAGTACTTTGTTCTCATATTGATTATTGCAACTTCTTGTCCAAACTCCATTGCCTTGCTTGGAAGAGAAAAATGGCAACAAATGGCAAGACACCTGCGATAGGAATCGATTTGGGCACGACATACTCATGCGTTGCAGTGTGGCAGCGTGATCGAGTGGAGATCATCGCGAACGACCAAGGAAACAGAACAACACCCTCTTATGTTGCTTTCAATAACACCCAAAGGATGATTGGTGATGCTGCCAAGAACCAGGCTGCTACCAATCCAACCAACACTGTCTTTGGTAAAATACTAAACCCTTTAGCTTAATTATGCTTGCTTTAAATGGGAACTAAAATATATAAAGGTCATTTGCAATTTTTTCATTCATGAATATACTCACACGTGCATCCACACACACATACTAGTATTGGTTTAACTTCTGAAATGATCAAAATCCTAAAGGGAAGCAAGATATAAGGTTTCACATTGTTTGATAGGTTACTTGTTCGAACTTTTACTTGTCTCTTTAGATCGAGTGAGATAAATGTAAGCTTTTTTTAGCATAAATTCCCACTAAAAAACATTTTCCTTTGAATATACACTGCTTATATTAAGAATGGCTTGGAGAGTATATATCTTGGAATATATCTTCTAAAAAAATTTAATATATCTTGGAAGGAGAAAGCTTCAATAAGACTAAGAATAAGTTTTTGAGAATGTGGAAGTTTTAAAGTGAAAAAGTGTTTTCTACTTCTCCTTAATATAAAAAGCATTTAAGAGGGTTGAAAATACAATATAGCACAGTTAGACATCACCAAAAAAATTTGACCAATTAAATACAGCATATAATTGCTTTGTAAGTACGTACTAGTAGCAATGTAGCATCAAATTGATCAATATATATATGTGTGGATGGTGAGGTTGTCTGACATCATATTTTTGGGAAAGACAACTCAGTATACCTTGCTACCGTTACATATGGTCATACTCTGGATTGGGTCTGAAGATCTTTGAATTTGTTCCCTCTTGTGCTTCATTAGCCTCCTTTCTTGTTGATCTTTTCCACCACTCTTTATATATGATATTGTTGATTTTGTGGAGGAAATTATAAAAAATAGAGGAGAGAAGAGAGACAATACGTATGCAGAGGAAATAGAATTATTCTATTCTAATTCAAATTGTTCTCATCAGCGATACAATAAATAGCAAAATATAAACTAATTAGATAACAAGATATTAGCAAAACAGAATATTAAAACTAACTTGCTCCTTAAAGATAAGAATCACTTATTTGACTAGGCTAATCCATTAAAACTGACTTACCCCTAAAATATAGGAAATCAACTTATTTACTAAATCCTATTTTAAAAATTGAAAAATAAAATATTATGCAGTTACAAAAGTATATCTTCAACACTCCTCCTTGCTTTTGGAACTGCAAACTCCAATTCTTTGAGTTTAAGATTGTTGATAGGTAGTGACTTTGTAAACATGTCAGCCAATTGATCTTTAGACTTGCAGTAAATTAAGTTCACTTCTCCACTTTGTTGCATCTTTATCAAATAATAAACTTGGATGTTGAAATGTTTAATCTTCCCATGACACACGGGATTGTTTGCAATAGCAATGACTACTTGATTGTCAACAAAAATTCCAGTTTTGTTCTTTTTAGAAAATAGAATGTTTGACCTTGTTGAAGTGAGATACATTAGACATCCAATCAAGCTCCCACAATATCCTTCATCAATGTTATCAACACCTTCTTCCTTGCTAAACTTCTCCTTTTGATTCATTGGTGTGCTAACAGACTTGCATTCTTCCATTTGAAACTTTTTCAAATTTTCTTTTGCATATTTCCTTTTTTGCATGTTTAGCATTCTTTCAAGATGGCAATGATAAAGTCTCTTGTGCCAGAGTTCCGTGGGTGTGACTTGAGTGAAATAGGCTGTATGCTCCTCCTCTGCTGGATCAAATGAGAAGCTTTTACCTTTCATTTTAACCCTTAGAACTTCCCGGCCAAAATTGTCATAGATAAAGCAATGTTGATGTTCAAAGGACACTTTAAATCCCTTTTTAATCAACTGACCTACACTTAGCAAGTTTTGGTCAATGTTAGGTACATAAAGAACATCTGATATTAATTTGATACCTGAACACGTTGAAATTGCAACAGTTCCTTTTCCTTTTACAGGAATATAGCCACCATTCCCAATTCTGACCTTTGAGACATTAGTTGGCTTCAAATCTTTGAATAAGGTCTTATCATATGTCATGTGGTTCGTACAACCACTATCAATCAACCAACTTTTCCTTGATTCACTACTCAAGAAGCATGTGGCCACAAACAGTTGGTCATCTTCTTCTTGATTAGCAATCTGAGCTCCCTCTTCATGGTGATTTTTGTTGGGGCAGATCACCGCTTCATGTCCTATCTGGTTGCACTTGTTACATTTTGCATCTGGTCTCCTCCAACATCTGAAAGGTGCATGACCTTTTTTGTCACAATGCTGACAAGGTGGATAATTTTTCTTTTTATCCTTACCTTGGTTGTTTGCACTGTTTTCGCTGCTTGCTGGTTGATTCTTCTTGAAAAAATCATTTTTGCTTTCATCAACTTCATGATGTTTGGCGGGCAAAGCACCTTCGACAACACGATCTTGCCTCATCAACCTTCGCTGCTCTTGAGCTTGCAGGGCATGTAGCACTTCTGTCAATGTGATTTTCGACAGATCCTTTGTGTTCTCCAATGAAGCTATAGATGCTTCATACCTCTCCGGCACCGTTACCAAAATTTTCTCTACAATTCTCGAATCAGCAAAATCACTTCCCAACAACTTTATCTTGTTGGCAATACCCAACAATTTGTTTGAGTATTCTTTGATTGTCTCTGACTCTTGCATCCTTTGAAGCTCAAATTCTCTACTTAAATTCAGCACTTGCATGCTTCGTATTCTATCATCTCCAGCGTATTCCTCTTTCAGATAATCCCAAATTGCTTTGGGTGATTTAAGAGTCATGATTCTGATGAATATCATTTGTGAAACACCAGTGAACAAACATGATCTCGCATTTGCCTTCTTCATCTTTCTTTCCTTGTGATTTTTAATTTGGGCCATGGTGGGATTTTCAGGCAGCGGAAATATTTCATAATCCTCTTCCACAGCATCCCATAAATCCAAAGACTCCATGTAGGATTCCATTTTCACTTCCCAAAGATCATAATTCTCTCCATCAAAGATTGGAAGAGTTATGTGGGAAAAACTTGCTTCACCTTCCATGGTAGAAGTCCCGTAAGAATAAGGCTCTCGATACCAATTGTTGGTTTTGTGGAGAAAATTATAAAAAACAGAGGAGAGAAGAGAGACAATACGTATGCAGAGGAAATAGAATTATTCTATTCTAATTCAAATTCTTCTCATCAACGATACAATAAATAGCAAAAGATAAACTAATTAGATAACAAGATATTAGCAAAACAGAATATTAAAACTAACTTGCTCCTTAAAGATAAGAATCACTTATTTGACTAGGCTAATCCATTAAAACTGACTTACCCCTAAAATATAGGAAATCAACTTATTTATTAAATCCTATTTTAAAAATTGAAAAATAAAATATTATGCAGTTACAAAAGTATATCTTCAACATGATATAATGGCTATAATGTCGGTTTTTTGGAGCTTGTCTTCCCCCAAATATTGGGGAAAACTGATCCTGGGTAATGTAATAAAATCATTTTTTTACAAATTGTAATAAAATCTTATTACATAACTAACATACTGTATTAGTTTTTATTTAATTAAAAAGTTTGAATGTGTTTTATTGAATACGCACCTAAACTTCTTTTTTTCTACAAGGTCAACACATTTTTTTTAATAAAAATGCAACTATAAAAGCTTCGCTCTTTTGCTATGACTTTTCTATAACGGTTCTTCTCCTGTTGCAATTATTATATATAGGAATAATATGGATGACGATGCCAGCTTTTCAAGTTTTGAAGTCAAATCTCCTTACTATAAGCAAACCGGCCGTATGAAAGCTTTCTCCTTCCTCTTGTAAAGGATAAAGCATATATAGTATAAAGAATAAAATACATAATTATTCTTAATTAAAAATTAATGATTAAATTTTGATAATTTTATAATTTATTTATATATTTTATTATAATTTTAATCATTAATAAAAAACAACTATTATTAATATATTTTTATCTTCTAAAATATACTCTAGTACACCCTTTAAAAACGACAAATCTATAAATCTTGTGATATTATATTAACTGTCAATATTCTATATATATATATGATTAATTATATCATATATCCTAACTGTTAATTATTATATGAGAAATTGAGAATGGATCTTCTAGTGAAAATAAATATTTTTGGAAATGAATATCATATCACTTTTTCTTTATAAAATATTACTATTTTAGATTACAACATGTTTAGATATAAGTTGGGCTCAGTAAGTTCACATTCTAGTGCTCTTTTTATTAAGAAGCTAAAAATCAAATCATTGCTGAGCTTGCATATGAATGTGGGCTTGCAACCTCTTAAAAAAACATCATGTTAGTATTTCATCAAGCTCACCCTAAAACATAAGAGTTAAAATGCTATATATGTTCAGTGTAAAAAAATTTATTTTGTGAGTCCATTCAAAATTATATTATCATTATTATAAAAAAAATTAATTATATAATAATTTGTGATGGGAGCAGAATGCAAAAATTCTTTCTTTATTTATTCCTACTATTGTTATTATTATTTGCATGCTACATAATGCTATCATCATTATATCTAAGGATTTATTAGAGTGTCTCCGATGAGGTCCCAACATTTTGATTGTTGATGACATGATGGCTACGTATAGCAATTTAAAATACTATATATATCACTGAATATAATGAGATTTGTTTTATTTTGTAGACGCAAAGCGCCTAATTGGTAGGAGATTTAGTGATCAAGAGGTTCAAAGTGATATGGAGCTATGGCCATTCAAAGTCATTGCTGATGTTAATGGCAAACCAATGATTGCTGTTGATTACAATTGTGAGAAAAAGCAATTTTCTGCAGAAGAAATTTCATCCATGGTTTTGGCAAAGATGCTTGACATTGCAGAGTCTTTCCTTGGATCAACAGTGAAGAATGCTGTTATCACTGTGCCTGCTTACTTCAATGATTCTCAGCGACAAGCTACTAAAGATGCTGGTAAAATTGCTGGCCTCAATGTTTTGAGAATCCTCCATGAGCCAACTGCTGCTGCAATTGCATATCGGCTTGAAATGAAAAATTGTAATAATGATAGAAGGAACGTTTTTGTGTTTGATCTTGGTGGTGGTACTTTGGATGTGTCTCTTCTTGTTTTTGAGAAGGATCATATCCGAGTTAAGGCAACTACTGGAGACACTCACCTCGGAGGAGAGGACTTCGATAACAATATGGTGACTTACTGTGTGAAAGAGTTTAAGAGAAAGAATAAAATGGACATTAGTGGAAACAAAAGAGCCCTTAGGAGGTTGAGGACTGCTTGTGAGAAAGCAAAGAGGATACTCTCATGCTCTACAATGACCACCATTGAGGTAGACTCTTTGTATGATGGTATTGATTTCCACTCATCAATAAGTCGCGCAAAGTTTGAGGAACTCAACAAGGACTACCTTAACAAGTGTATTGAGTTTGTAGGGAAGTGTCTGATAGATGCTAAGATGGACAAGAGTAGTGTTCATGATGTTGTCCTCGCAGGTGGATCTACTAGGATTCCCAAATTGCAGCAACTATTAAGTGACTTCTTTGATGGGAAGGATCTCTGCAAATGCATCAATGCTGATGAGGCCGTTGCATATGGTGCTGCTGTCCATGCTTATATGCTTAATGGTGAGTCCAGTGAGAAGGTTCAAAACGCTTCACTTTGGGAAGTCACTCCTCTTTCCCTTGGGTTGCAAGAAGATGGAGGTATCATGAAAGTAATCATTCCTAGGAATACTAGCATTCCTACAAAGATGGAAGATGTACTCACAACACATTTTGATAACCAAACCAATATCTTGATTCATGTATACGAGGGTGAGAGGAAAAGAACTAGAGACAACAACTTGTTGGGTAAGTTTGTGCTAGAAATTCCTCCAGTTCCACGTGGTGTTCCTCAAATTAGCGTTTGCTTTGAACTTGATTATGATGGCATCCTACATGTTTCTGCCGAGGAAAAATCTAGGGGAATATCCAAGAAGCTGGCCATAACAAATGACAAAGGAAGGCTTTCGAAGAAAGAAATTGAAAGGATGATATCAGAAGCAGAGAAGTACAAAGCTGAAGATGAGATGTATAGGAACAAGGTACAATCAAGGCATGCATTGGAGAAGTACGCTTACAACATGAGGGATGCTATAAACATTAAGGAGATTAGCTTGAAGCTTTCTCCAGAAGACAAGAAAAATATCAATGATGCAATTGATTCTGCCTTAGAGTGGCTCGAGGTCAGCATGGATGCAAACCCAAATGATTTTGACAACATGCGGAGCACTCTTTCTAGCGTTTTTAATCCAGTTATTGTGAAGATGATAAAGGATGAGGATAATGTTGCGCCTCCAGATACTGTTGCTAGCTCTGGTAGCAATAGTGTGAAGAATGGCTTGTTATCAATATTGGCAAATTTTGCTCTCGATGCAGTGTATTCAGCTGCTACAGGTGATATCATTGGATTTGCTTCCGTGATTGTTGACTGTTTGTCAATTTAGTTAGTTTATTAAAACTTGGTAAAATGTCAATAGAGCAAGCGGTAAATAAACAGCTTCTTATTCAAAGTGTGAGTTTCTGACTAGGAACTATTATGAGATTAATTAATGTGTAAGACTTGTTTTCTGGTGTATCTGATCCCATTTTATCAGATTGATTTCTTTTTCCTGCATCTTGTCATGCATTGCTGGTAGTAATAACAAAAAGACGTTCTGGTTAAAAGAATTGGGGAAATTCGCTCTCAAAGCAGTATTTTTAGCTGAGGAGAAGCAAAATGAATAGCATGCTATCAACCTTAAAGGTGGCAGTTTCTGTTGTTAATGTTGTAATACATGATTTCTGGTGTATGTGATCCCTTTTGTCAGAATGGTTTATTTTTCTC

>Glyma18g52610 ATTCACACGTCGATGGAGAAAGTAGCGTTGCGTGTCCAAGGTGGGCAGATATAAAGAAGAATCTGGAACCAACCAGAAGCGGAGTAGACAAAAAGAACATTCTAGAATTCTGATTTTCAAAACAGAAAGTTATATAAACCCTCCCTTTCTCTCTACTCTAACACAATTCATTCGTAGCCAATTCTTATTCTTTTTCTCTCTTTCTCTCTGCTTGCGGTGCTTCACAAGCCAAATCTAACTTGTCTCTGTTTTCTCCGATCTAGTACTACTATCAATGGCCGGAAAAGGAGATGGTCCTGCTATCGGAATCGATCTCGGCACCACCTACTCCTGCGTCGGAGTGTGGCAGCACGACCGTGTCGAAATCATCGCCAATGACCAGGGTAACAGAACCACGCCGTCTTACGTCGCTTTCACTGATTCCGAGCGTTTGATCGGTGACGCCGCCAAGAACCAGGTCGCCATGAACCCCGTCAACACCGTCTTCGGTAAAATCCCTAACCGACTTTTTGCTTTATCGAGATCTGCATTGTTCCTAGATTTTTGAAACTCCACTTTTCTATTTTAGTAATTTGTAGTATTTTCCAATCCTAATGTATTATCTGGCCTATTATTTTTTTGATATAATAATAATAATAATAATAATCGTTGAATGGTTTATTATGTACACTCTTATTGTTCGTTGAAATCTGATGTTGAATTGAATAGCAATAGGCGTAGGCACTGTTAATACTTGTCGAAATGTGATTTTGAACTGAATAAATCATATTGTGTGCAATTTTATTATTTAGAGAGTTATTTGATCCTACAATTTTGAGGGAATATGGTATGCTAAAAGTTAAGATTTATCTTTTATTCTTTTTAATTTGGATTGTGCGGATTGTTTGGTGATTGGTTATTTGTTGCCTGATGTCAGATGCTAAGCGTTTGATTGGAAGGAGATTTTCTGATGCCTCCGTTCAGAGTGACATGAAGCTATGGCCATTTAAGGTCATCCCTGGTCCTGCTGACAAACCTATGATTGTGGTCAACTACAAGGGTGAGGACAAGCAGTTCTCCGCTGAGGAAATTTCTTCCATGGTTCTCATGAAGATGCGTGAGATTGCCGAGGCTTATCTCGGTTCCACAGTGAAGAATGCCGTGGTCACTGTTCCCGCTTACTTCAATGACTCCCAGCGTCAGGCCACGAAGGATGCTGGAGTCATTGCGGGTCTCAATGTCATGCGTATCATCAATGAACCCACCGCTGCTGCCATTGCTTACGGTCTTGACAAGAAGGCCACCAGTGTGGGTGAGAAGAACGTGTTGATTTTTGACTTGGGTGGCGGTACCTTTGATGTCTCTCTTCTCACCATTGAGGAGGGTATTTTTGAGGTCAAGGCCACTGCCGGAGATACTCATCTTGGAGGTGAAGATTTTGATAACAGGATGGTTAACCATTTTGTTCAGGAATTCAAGAGGAAGCACAAGAAGGACATCAATGGAAACCCTAGGGCTCTTAGGAGGTTGAGGACTGCCTGTGAAAGGGCGAAGAGGACCCTATCATCTACTGCACAAACCACAATTGAGATAGATTCTCTTTATGAGGGTGTTGACTTCTACACCACAATCACCCGTGCCAGGTTTGAGGAGCTCAACATGGATCTCTTCAGGAAGTGTATGGAGCCCGTTGAGAAGTGTTTGAGGGATGCTAAGATGGACAAGAGCACTGTCCATGACGTTGTCCTTGTTGGTGGATCCACTAGAATTCCTAAAGTGCAGCAATTGTTGCAGGATTTCTTCAATGGCAAGGAGCTTTGCAAGAGCATTAACCCCGATGAGGCCGTTGCTTATGGAGCAGCTGTACAGGCTGCTATTTTGAGTGGTGAGGGCAACGAGAAGGTGCAGGATTTGCTGTTGTTGGATGTTACACCCCTTTCTCTTGGTTTGGAAACTGCCGGCGGCGTCATGACTGTTCTCATTCCCAGGAACACGACTATTCCCACCAAGAAGGAGCAAGTGTTCTCTACTTACTCAGACAACCAACCTGGTGTCTTGATTCAGGTGTATGAGGGAGAGAGAGCTAGAACCAGGGACAACAACTTGTTGGGTAAATTTGAGCTTTCTGGCATTCCCCCAGCACCCAGGGGTGTTCCTCAGATTACTGTCTGCTTTGATATCGATGCCAATGGTATCTTGAATGTCTCTGCCGAGGACAAGACCACAGGGCAGAAGAACAAGATCACCATCACCAACGACAAGGGTAGACTATCGAAGGATGAGATTGAGAAGATGGTCCAAGAGGCGGAGAAGTACAAGGCCGAGGATGAGGAGCACAAGAAGAAGGTCGACGCAAAGAATGCTTTGGAGAACTATGCCTACAACATGAGGAACACCATTAAGGATGAGAAGATTGCATCAAAGCTTTCTGATGACGACAAGAAGAAAATCGAAGATGCCATCGAGAGTGCTATTCAGTGGTTGGATGGAAACCAGCTAGCGGAGGCTGACGAGTTCGAAGACAAGATGAAGGAGCTTGAGAGCATTTGCAACCCAATCATTGCCAAAATGTACCAGGGTGCAGGTGCTCCCGACATGGCTGGAGGCATGGATGAAGATGTTCCTCCATCTGGATCCGGTGGTGCTGGCCCCAAGATCGAGGAAGTTGATTAAATTGTTTCATTATTTAGATGGGATGGGCTTGTGTTGGATCTATTCCGTTTTTTTTTCCTTTATTTTATTTGGTCTAGAATATTGGCCCATTATTTTGGTTACTAACACCAGGTATGGTGATTGGTAATTCTTTTTTTTTGGTTTTGAATAGTTTATCCAGATTTCACCTCGATGGAGACTTGTTTAAAAATTTTTGCCAAAGAAAACAATTTTATGGGCCGTTCCTATTTAATGTTTTCTTAAATTTTGATGTTTGATATCTACGTTACTCGTGCAAATTGCGCTGTTCCTCTGGTATAGAAGCGAAAAATGTTTTTATAACAGTATTGTCTAGTTAAATGT

>Glyma18g52650 CAACCTCTGAAGAGAACTACTGCGGGTAAATAAATGGGCTGGATTAGAAAAAGGGCTTGCCACTTGACAAAAGCGGCTATTGCTTCCTGCACCTCCCTTAAAAAAATCAATTTTGAGTGAGTTGGGCCTATTAAACCCGGAACCAGCGTAGCAGTGGGTTTGGTTTGGTCCAATGAACTGAACTGATTCAAAGAGAACGAGCAACGAGGCAAATGAAAAGCGGCTTCAGTTAAGGTGAGGGCCCCACGTAAGAGTAGAAGATTCTAGAGTTAGACACAGTGTCTCTCTGAAACCTATATATACCTCACTTCTCCTCCTTCCATTCATTCACAGAAAACTGAACCAAGAAACCCAAGTTAAGGTTTTTTGCAATCTTCCCTTCTAAAAAGAAATGGCCGGAAAAGGAGAGGGACTCGCCATCGGAATCGACCTGGGCACCACCTACTCCTGCGTCGGAGTCTGGCAGCACGACCGCGTCGAAATCATCGCCAACGATCAGGGCAACCGTACCACACCCTCTTATGTCGCCTTCACCGATACCGAAAGACTCATCGGAGATGCCGCTAAGAACCAGGTCGCCATGAACCCCATCAACACCGTCTTCGGTAATTCTTCTCAGTCCTCATTATCTGCTCAATAATATTTTTACCTACATTTTTCTAACAAGCTTTTTACATTTTATCGTAATGTTTTATGTTTTATTGTTCTCATTATTTTTTTTATCCGCAAATATTATTTTGTTAGTTTTGTTAGAAATGGACTCGAACACGCGACTTCTCCCCGTACCTTTCGTCAAACACCGGGTCAACCTATATCTCCTTTTAACGAGTATTAGTGTGGAGTTTTGTTGTAAAATCTTAATTACGCGTTTAAGCTGAGTTCATGTTTGGTTCATTTCAATTACGATTATTCAAGCCTTCTTCTTTTTAAGAAATAGGATGACAATTTTCAAGTTTTCAAGGTTCTTGATATTGCGGGTTACAATTGTGGAAGCCTCTGAAAACTTGTGGACCGTTTCGTTATTATTTTATTAAAATGAGCTCAATCCAAAATTACATGTTTGGAAGCAGATGTGGTCCTCTTTGGTTTGTTTATAGGGTTTGAATTGAGATCAATATATATATATATATATATACATATATATATATATATATATATATATATATATATATATATATAAAATATTATATAAAATAGGAATTCTCTTAATCTACCCTAATTTAAGAATTGTTTTTTATTCCCGCGATATACTTGTTTCAATATTCTATATATGTACAGTGTGGAAGGTTTTCATTCTATTGTGGTGTAATTTGTGAGTTGATGATGCTTGGTGTGATTGTTGATAACTGGGGGTTGTCACTTTGCAGATGCGAAGAGGTTGATTGGTAGGAGAGTTAGCGACCCTTCTGTTCAGAGTGATATGAAGTTGTGGCCATTCAAGGTTACTGCTGGTGCTGGTGAAAAACCCATGATTGGTGTCAATTACAAGGGTGAGGAAAAGCAATTCGCTGCTGAGGAAATCTCCTCTATGGTCCTAACAAAGATGCGGGAGATTGCAGAGGCTTACCTTGGGTCGACTGTGAAGAATGCCGTTGTTACTGTGCCTGCTTACTTCAATGATTCTCAGCGTCAAGCCACCAAAGACGCCGGTGTCATTGCTGGCCTCAATGTTATGAGAATAATAAATGAGCCAACTGCTGCTGCAATTGCATACGGGCTTGACAAGAAAGCCACTAGTGTTGGTGAGAAGAATGTCTTGATCTTTGATCTTGGAGGTGGCACCTTTGATGTTTCTCTCCTCACCATTGAGGAGGGTATCTTTGAAGTTAAGGCCACAGCTGGAGACACTCACCTTGGAGGGGAGGATTTTGATAATAGAATGGTGAACCACTTTGTACAAGAGTTCAAGAGGAAGAACAAGAAGGATATTACTGGAAACCCAAGAGCCTTGAGAAGGTTAAGAACCTCTTGTGAGAGAGCAAAGAGGACACTCTCATCCACTGCTCAGACCACCATTGAGATTGATTCTCTGTTTGAGGGCATTGACTTCTATTCAACCATCACTCGTGCCAGGTTTGAGGAGCTCAACATGGACCTCTTCAGGAAATGTATGGAGCCTGTGGAAAAGTGTCTTAGGGATGCAAAGATGGACAAGAGCTCTGTTCATGATGTTGTCCTTGTTGGTGGCTCTACAAGGATTCCTAAAGTTCAGCAGCTGTTGCAGGACTTCTTCAATGGGAAAGATCTTTGCAAGAGCATCAATCCCGACGAAGCAGTTGCTTATGGAGCTGCTGTCCAGGCAGCTATATTGAGCGGTGAAGGCAATGAGAAGGTTCAAGATTTGTTGCTTTTGGATGTCACTCCATTGTCATTGGGTTTGGAGACTGCTGGAGGTGTCATGACTGTATTGATTCCAAGGAATACTACCATTCCTACTAAGAAGGAACAAGTGTTCTCTACATATTCAGATAACCAACCTGGTGTCTTGATTCAAGTTTATGAGGGTGAGAGAACAAGAACCAGGGATAACAACTTGTTGGGTAAGTTTGAGCTGTCAGGCATTCCTCCAGCTCCCCGTGGTGTCCCTCAAATCACTGTTTGCTTCGATATTGATGCTAATGGTATCTTGAATGTCTCTGCCGAGGACAAAACAACTGGCCAGAAGAACAAGATTACCATTACCAATGACAAAGGAAGGTTGTCAAAGGAAGAAATCGAGAAGATGGTTCAAGAAGCTGAGAAATACAAGTCTGAAGATGAGGAGCACAAGAAGAAGGTTGAGGGAAAGAATGCCTTGGAGAACTATGCCTACAACATGAGAAACACAATAAAGGATGAGAAGATCAGTTCAAAGCTTTCCTCTGAAGACAAGACAAAGATTGATAATGCAATTGAGCAGGCCATTCAATGGCTTGATACCAATCAGCTTGCAGAAGCTGATGAATTTGAAGACAAGATGAAGGAGCTTGAGGGCATATGCAATCCCATTATAGCGAAGATGTACCAAGGCGGAGCTGGTACTGGTGGTGACGTCGATGATGATGCTCCACCTGCTGGTGGTAGTGGTGCTGGCCCCAAGATTGAGGAGGTTGATTAAGTGGACTAGTTAGTATTTGGAAGGAATTAGGAAAATAATGTTTGTTAAGGTTTTTGGGTTTAGGCTATATAAATTTTTTGCTTTCATGAATTGTACTGCTGTTTTCAATTTGTCATTTGTTTGATAATTGAAAACTAGCCCCGCTTTGCAAATTGTTACTCTGGATAATATAATTTTAGCGTC

>Glyma18g52760 AGGCAGATATTAATTCCTTTTATGCAATCAATATATATACGTATGTTTAATTATAAATATTTTAGCTGATTTTCATATTTCACGCGTATAAAAATCTAACTTGAAATATTCAACCATACCAGAAGTCTTTCAAAGCCTGCATTCAATTACTACTGTTCATCTGGGTCTTCATTCACGTGAAATAGAATGGCCAAAAACCAGGGATTTGCCGTAGGAATCGACCTTGGTACAACCTACTCGTGTGTTGCAGTGTGGCAGGGGCAACAAAATAGAGTAGAAATAATTCACAACGACCAAGGGAACAGAACTACACCTTCTTTTGTCGCTTTCACTGACGATCAAAGGTTGATTGGTGATGCTGCTAAGAATCAGGCTGCAGCCAACCCAGAAAACACTGTCTTTGGTACGTTTTTTTTCCTTCTTGATTATTCTCTGGACTATTAAAGGCAAAATGAACAGCAACATATTATAAATTTTGCAGATGCCAAGAGGTTAATTGGTAGGAAATACAGCGATCCCACTATTCAAAATGATAAAATGTTATGGCCATTCAAGGTCATAGCTGATAATAATGACAAACCCATGATCACCGTTAAATACAAGGGCCACGAGAAGCTCCTTTCAGCAGAGGAAGTGTCATCTATGATCCTCATGAAAATGCGGGAGATTGCTGAGGCATATTTGGAAACACCGGTAAAGAGTGCTGTTGTTACAGTGCCTGCTTATTTCAATGATTCTCAGCGTAAAGCCACCATAGATGCTGGAACCATAGCTGGCCTTAATGTTATGCGGATAATCAATGAACCCACTGCAGCAGCTATTGCATATGGCCTTGACAAGAGAATTAACTGTGTTGGAGAGCGTAACATTTTTATCTTTGACCTTGGTGGTGGTACTTTTGATGTTTCTCTCCTTACAATTAAGGACAAGGTCTTCCAAGTCAAGGCTACTGCAGGGAACACCCACCTTGGGGGAGAGGACTTCGACAACAGAATGGTGAATTACTTGGTGCAGGAGTTCAAAAGAATGAACAAGGTCGACATTAGTGGTAACCCCAGAGCTTTACGAAGGTTGAGAACTGCGTGCGAAAAGGTGAAAAGGACACTCTCGTTTGCTGTTACAACCACAATTGAGGTTGATTCATTATCTAAAGGCATTGATTTCTGCATTTCAATCACTCGTGCAAAGTTTCAGGAACTTAATATGGATCTCTTTGAGGAGTGTCTGAAGACTGTTAATAAGTGTCTTACAGATGCAAAGACGGACAAGAGCAGTGTACATGATGTTGTCCTTGTTGGTGGTTCTTCGAGGATTCCCAAAGTGCAGGAACTATTGCAGGAATTCTTTGAGGGAAAGGATTTTTGCAAGAGTATCAACCCTGACGAGGCTGTCGCTTATGGAGCTGCCGTGCAGGCTGCTTTGTTGAGTGACGACATTCAGAATGTTCCAAATTTGGTTTTGTTGGATGTTGCACCGCTGTCACTTGGTATATCCACAAAAGGAGATCTCATGAGTGTTGTGATTCCTAGGAATACTACTATTCCAGTTAAGAGGACTCAAGGAAGATAACCAAACCTCTGCGCGTATTGAGGTTTATGAAGGCGAGAGAACCAGAGCAAATGATAACAATTTGCTTGGTTTCTTTAGTCTTTTGGGTTTGGTTCCTGCTCCTCGTGGCCATCCTGTGGATGTATGCTTTACAATAGATGTAAATGGCATTCTATCTGTTTCTGCGGAGGAAACAACCACTGGTTATAGGAATGAGATTACCATAACCAATGACCAAAAAAGGCTTTCAGCTGAGCAGATTAAAAGAATGATTCATGAAGCTGAGAAATATCAGGTTAATGATATGAAGTTCATGAAGAAGGCTAATACAATGAATGCTTTGGATCACTATGTTTACAAGATGAGGAATGCATTGAATAATAAGAATATCAGTTCAAAGCTTTGTTTACAAGAAAGGAAGAAAATCAAATCTGTAATTACAAAGGTGACTGATTTGCTTGAGGGTGATAATCAGCGGGATAAAATAGAGGTGTTTGAGGATCATCTAAATGAGCTTGTGAACCTCTTTGATCGTGTCATTGGCAAGTTTGCTTAGATGTTTTTTAAGTTTCTATTTTTTGCAATGCTGAGATCTGTCATTTTGTTTGTATTGAATGCAAACTTTTCTTAT

>Glyma19g35560 GCCAACGACTCTAAAGAACATTCTGGAAGGACTCCAAACCCTAACTATTCTACTATATAAGCTAACTATTGCCCCTCAGATCTCTCACTTGAATCACAGTGGTGCCTTATCGCTGTGGTTTTCATCGTCTTCCTTCTCTTATATTTCTTTTTTTTTTTTGTCTTCGCCGCACGCCGAAACAATGGCCGGAAAAGGAGAGGGTCCTGCTATCGGAATCGATCTCGGAACCACCTACTCTTGTGTCGGTGTGTGGCAACATGACCGAGTTGAAATCATCGCCAACGACCAAGGGAACAGAACGACGCCGTCTTACGTCGGCTTCACTGATACCGAGCGTCTCATCGGTGATGCGGCTAAGAACCAAGTCGCCATGAACCCCATCAACACCGTCTTCGGTAACCTCTCTTAACCCCAATTTTTTTTATCTTCTGTTTTAGTTTTTCTGTTTATGCATTTTTATTTTAGTTATCAGTTATAGGCTAAACTATTCACTTTCTCCTTATAGTTATTCCATTTTTTTGCTTAATCTGTTTTTTTAGTCCTCTAATTAGAATTTGCATTTTATTCTCTCAGTTTTGGGAAATTCTTTTTTAGTCCCTCTTGTGGTCAATAATCGTTCTAGATTGTACATCCAAACCGTTAAAAATAATTCATGAAAGACAAAATCAAGTATTTTACGAAAGCAATGTAAATTATAATATTTATAACTAAAAAGGACAAACCCTAATTTAAGACCCTAAAAACATTTTTTTAGGGTTAGTATACATGCTCGGAAGGGAACTAAAACCTAAAATTAGATAAAACTGTAAGTATCAAATGAATAGTTTAACCCTAGCAATATGTATATAATACCTCAAGTTTTCAGATCTACTGTGGTATAGAAATTTGATCAATTATAAGATTCATGGTATTGAGTTAATTTAAATATTTTTAAGAATGTTTGATTTATGTTTCAAATTGTAAGTGTTATTGTGAGTTGCAAAATGGCTTTTTCGTAAGATGCTTTGTTTTTGTTGCAGATGCCAAGAGGTTGATTGGTCGTAGATTCAGTGACTCCTCTGTTCAGAGTGATATCAAATTGTGGCCTTTTAAGGTCATTGCTGGTGCTGCTGACAAGCCAATGATCGTGGTTAACTACAAGGGTGAAGAGAAGCAATTTGCTGCAGAAGAAATCTCTTCTATGGTGCTCATCAAGATGCGTGAGATTGCTGAGGCTTACCTTGGCTCCACGGTGAAGAATGCTGTTGTCACTGTCCCTGCTTACTTCAATGATTCTCAGCGTCAAGCTACCAAGGATGCTGGAGTCATTGCTGGTCTTAATGTGATGCGAATTATCAATGAGCCTACTGCAGCTGCCATTGCTTATGGTCTTGATAAGAAGGCCACAAGTGTTGGTGAGAAGAATGTGTTGATTTTTGACCTTGGTGGTGGGACATTTGATGTTTCTTTACTAACCATTGAGGAGGGTATCTTTGAGGTGAAAGCCACAGCTGGTGACACCCATCTTGGAGGTGAGGATTTTGATAACAGAATGGTGAACCACTTTGTTCAAGAGTTTAAGAGAAAGAACAAGAAGGACATAAGTGGGAACCCCAGAGCACTTAGAAGGTTGAGGACTGCTTGTGAGAGGGCCAAGAGAACATTGTCATCCACTGCCCAGACCACCATTGAAATTGATTCTCTCTATGAGGGAATTGATTTCTACTCCACTGTTACTCGTGCCAGATTCGAGGAACTGAACATGGATCTCTTTAGGAAGTGTATGGAGCCGGTGGAGAAATGTTTGAGGGATGCTAAAATGGACAAAAGAAGTGTTGATGATGTTGTCCTTGTTGGTGGTTCTACCAGAATTCCCAAGGTTCAACAACTGCTGCAGGACTTCTTTAATGGAAAGGAGCTGTGCAAGAGCATCAATCCTGATGAAGCTGTTGCATATGGTGCTGCTGTTCAGGCTGCAATTTTAAGTGGTGAGGGCAATGAGAAGGTTCAGGATCTTCTCCTCCTTGATGTCACCCCTCTATCTCTTGGTTTGGAGACTGCTGGCGGTGTGATGACTGTCTTGATCCCTAGGAACACTACAATTCCAACCAAGAAGGAACAGGTTTTCTCAACATACTCTGACAACCAGCCTGGTGTGTTGATCCAGGTCTTTGAAGGTGAAAGAGCAAGGACTAAAGATAACAATTTGTTGGGCAAATTTGAACTTTCTGGCATTCCTCCTGCCCCCAGGGGTGTTCCTCAGATTACTGTGTGCTTTGACATTGATGCCAATGGTATTTTGAACGTCTCTGCCGAAGATAAGACCACTGGCCAGAAAAATAAGATCACTATCACCAATGACAAGGGTAGACTGTCAAAGGAAGATATCGAGAAGATGGTTCAAGAGGCTGAGAAGTACAAATCTGAGGATGAAGAGCACAAAAAGAAGGTTGAGGCCAAGAATGCTTTGGAAAACTATGCATACAACATGAGGAACACTGTGAAGGATGACAAAATTGGTGAGAAACTTGATCCTACTGACAAGAAGAAGATTGAGGACGCAATTGAGCAAGCTATCCAGTGGCTAGACAGCAACCAGCTCGCAGAAGCAGATGAATTTGAGGACAAAATGAAGGAATTGGAAAGCATCTGCAATCCTATCATTGCCAAGATGTACCAAGGTGGTGCTGGTCCTGACATGGGTGGTGCTGGTGCTGGGGCAGCCGAGGATGATTATGCTGCTCCTTCTGGTGGAAGTGGTGCTGGCCCCAAGATTGAGGAAGTGGACTAAATGTGTAGTTGCTTTCATCCATTTATCTGTTTAAAACTTATTTGATGCTTTTTTATTGAATTTTAACTTTTATGATTTTTGTTGGATGGGATACTAATTGTTTTGCCCGTGTGATATGAACGTTGCTTTACGCATTATGTCATTTGCTTTATTTCATTAGATGAGAATACTCATTGTTTTACACTTTGTTCGTAGGAATTATTCTGTCTTGATATTTTAAATGGCATGATCATCTATTTTGCCA

>Glyma19g44140 CACAAAAACAAAAACATAACATAAACCCTCCCAATCCCATTCCAAAGTCTAAGGAACGGTTATCCCTCACAAACCGCCGGCTGTCTCAGGTAAACAGGTGTTTCTTCGCCTCCCATCTCAAATTTTCAGCTTTTCCTACACTCCACGCTCAAAAGTCAAAAAATATTTTTCTTTTTCTTCTGGGTTTTGTGATTGTACACTCTAACCAGTAATCAATCATTTTTGTTTTTCCCTAGAAAGTGAAAATGAAGAGAATGATAGCCTTGGGGTTTGAGGGTTCAGCGAACAAGATTGGTGTTGGGGTAGTGACCTTAGATGGCACAATTCTTTCAAACCCACGGCACACATACATCACTCCTCCTGGCCAAGGCTTTCTTCCCAGAGAGACAGCACAGCACCACCTCCAACACGTTCTTCCCCTTATCAAATCCGCTTTGGAAACTGCACAAATCACTCCACATGACATTGACTGCCTCTGCTACACCAAGGGTCCTGGCATGGGAGCTCCTCTGCAAGTCTCTGCTATTGTTGTTCGTGTGCTTTCGCTTCTTTGGAAGAAGCCAATTGTTGCTGTCAATCACTGTGTTGCACACATTGAGATGGGAAGGATTGTTACCGGCGCTGATGACCCCGTTGTCTTGTATGTCAGTGGTGGCAACACTCAAGTCATTGCTTACAGCGAGGGGCGTTATCGAATCTTTGGAGAGACCATCGACATTGCTGTGGGGAATTGCTTGGATCGCTTTGCAAGGGTCTTGACACTTTCCAATGATCCTAGCCCCGGATATAACATTGAGCAGGTCCTGCTTTACTACGTTTGAATGAACTTCTTCCTAAGTTTTTATAGGAGTGGAAACTAAGAAGACGGTATGAATTGAGTTTCTCCCAGAAGTTAAAATCAACTCATGCACCTCAATTTTTGGAGAAATTAAACAAGAGAGCCTCTACAAAAGTTAAGTGTATAAGTTTATATTAGTTAATAGGAGAAACTCAATTCATTTTATTTTCTTATTTTCTTCTTCTACAAGTAATTGTGGAGAAATTTTCCCAAACATGGTCAAACTGTGTTTTACATAGTTTATTTTATGCCTTGTGCATCATATGATATATGGGATATTTTTGTTATACCTTTAATGTGAAAATCTTTCTCAACTAATTATCTTATCCTTCAGCTTGCGAAAAAGGGAGAGAAGTTTATCGACCTGCCTTATGTTGTCAAAGGGATGGATGTATCTTTTAGTGGGATATTGAGCTATATTGAAGCAACTGCTGCTGAAAAGCTAAAGAATAATGAGTGCACGCCTGCAGACTTGTGCTACTCTCTGCAGGTGAATTTAATCTGTGGCGATCCCTTGCCTTCCCATTATATTATTAATTAATGAATGATGAATTTGCAAGTTGATGTGTTTTAGTATGCTGCAGGAGACATTGTTTGCCATGCTTGTGGAGATAACGGAGCGGGCTATGGCTCATTGCGACACAAAAGATGTTCTTATAGTTGGTGGTGTAGGTTGCAATGAGCGGTTGCAAGAGATGATGAGGACCATGTGCTCTGAACGTGGTGGAAGATTGTTTGCTACTGATGATAGATATTGCATTGACAATGGAGCAATGATAGCTTATACTGGTCTCCTTGAATTTGCTCATGGCGCATCAACTCCACTAGAGGATTCTACATTCACCCAGCGGTTCCGGACAGATGAAGTGAAAGCAATATGGAGAGAAGCAAATCTGGCAAAATTGAATGGGCTTGCAGAGAAGAGCACTTGATTTGTCATTCTTGAGGTGCAGTCCTATTCTTGGGCTGTTGGGCACTCAATTAAGCCTTTAGTGTAGAATATTTAGCGTGTTTTTGGGTGAGTTCCTGCAAAATTATTTTTTTTGTTAGAATTGTTTTTGATGTGAAGAATTTATGTTTGGAAGTTTGTGTTTTATAAGTTGGTAGTAAAAATTCAGTATGAAATTTTTGATCCAACACAAAAGCTGCCTAAAGTTGTTTCAATTTATAATCAATTCTTC

>Glyma20g16070 ATGATTGATTGGAATTATGGACGGATGATGACTGCTTGGTGAGTGTATGTTGGACTAATGGATGTTTGTGTATGATTATGGTATTTGTTAATGTTTTCTTACAAATCATGGTTATTTTGATTTTTGTGTTAATTTCTTTTATAATAAATTCACCCTTGCAATTTTTGTATTGTGTGGTGGTACCTGTGATGATCAGGAACCTTTGTCCGTGGGAGTAGGCTAACAACAATAGAGTACGTGAAATTAGATTCTTTTGTGGAGCCGCCAAACGGACGTGATGACATTGAGATTATTTTGGGATGGAGTTGTGTTTTATTAATCAACTTCTCTGTAGCTAGTTCTATAATTCCTTTTTTGAATTGAGGATGTAAATCATATTTAATTATACATATAAACTAATTTACTTTCCGTTATGTGAATGATGAGTACTGAGTTTATATATATATATATATATATATATATATATATATATATATATATATATATATAAGTAATTGTGTATTGTTTGGTGAATGTACATCGCAAAAAAAATTTACTCTCATTTTTCATAAGTAAATTAACAAAATTTTCCTTTAAAAATTTAAATTTAAATGGTTTAGAGTGATGATATCGTAACGGCGAGACGAGTCATTACATTGGTTGTGAGGAAACAATCCCTAAATAAAGTGGTCCAATTATAGTTCTTGATGAAACAAAATTATGTGTCAGAGGTCTACCAAAGGCTATGCGCATAAGGGACGAGATTGACTCAATATAATCTCAATGTACTCAAACATGTAGTCAATGCATCCATTAAGGGCACAACAAGACTAATTATCCACAAAATTGATCTCACGTAAATCATAATAATTTGTCATGCATTTAAATTTATCATATTTTAAGAACCTATAATATCTTCACTTGTTTTCACAAATACATATCAACTATTAAATATTAATATTATATTTTGTAACAGAGTAACACTGCTTAAATATTACAAATAAAGACAACTAAATTATAAAAAAATAGCAAAATATAACAAATTAACGAAAAACAAAACAGACAACCCATAAAGTAGTGGAAACCAATTATCTAACCTAAAAATTAATTAAAAAAAGGCTAAAATTGTTTCCATCCAAAAGAAAAAAAAAGATAAACCTAATTGAAAAAAATACTTAAAATAATTTTATTTTCTACAACTAAATTCTCAAAAAACTTAGAGAATTATATCTAATTTTAAATACATTAAAAAAATAAACTTTTAATAAAAAATTCACTTCTATTTATGTCAAAGAACAAGATGACGCCTCTTGTTGACAATGAATAGGATGACACCTCATATTCAACTACAAAAAAATATAAAGGCAAACCAGATGATACCTCTAGACAAACAAAGTGACATCCCCTATTGAGACTATAAAAAACGAAAAACAAGAAAGACAAATCATAGGATAACGCCTCCTTAAAAACAGGATGATGCCACTTTATTTGACACACTCACATGAAAAAGAACTTTAATTGGCATAGTCAAATTTTACATGGTTAGAATTACCCATTTATGTATATTGTCTTGAGACCCTCCCATAGATGTTTTGTTTAAAAAAGTACTCTATTTTGGTGAAAAAGCTTATAAACGGAGTGGTGAGTAAGGTTTTTGCAATCACGCAATGTAAGCACATAGTCTATAGTCTATAAGGTCAGAAGCGTCATTTACTGCATCATTTGCTAGACTCTTGACTCAGCGAGAACCTTCAGCAAAAGGGTGAAGGACGACTCAAGAACAACACCACACACCATCTCAATTCTCGCCACGTGTACATTCTTCCACGTGTCCTTTAATCACGGGCTAAATCCTTCTTCACTCTGTCACACTTCCTCCTCCGTATAATACACTCCAAAGTCTCCAAACAAACTCGTTTTCTGTTTCACTGTCATTATTATTTAAAATTATTATTGTTCATTTTATTAAACTCACCACCGCGCCACCACACTCTCTCGCATTTTCCCGCAAAACATATTCACTTAATTTTCCCTCACTTTCTCAGCGGCCAAACGCCGCAAAAAATGGCGTCGTTGAAGGTGGCGCTACTGGCGCTGTTCTCCGTCGCGCTCATGTTCTCTCCGTCGCAGTCCGCGGTCTTCAGCGTCGATCTAGGTTCCGAATCGGTGAAAGTGGCGGTGGTGAACCTGAAGCCCGGCCAATCCCCGATCTGCATTGCGATCAACGAGATGTCCAAGCGCAAATCCCCGGCGCTGGTCTCCTTCCACGACGGCGACCGCCTCCTCGGTGAGGAGGCCGCCGGCCTCGCCGCGCGCTACCCGCAGAAGGTCTATTCCCAAATGCGCGACCTCATCGCCAAACCCTACGCCTCCGGGCAGAGGATTCTCAACTCAATGTACCTTCCCTTCCAGACCAAGGAAGATTCACGAGGTGGCGTGAGTTTTCAAAGCGAAAACGACGACGCCGTTTACTCCCCCGAGGAGCTGGTGGCCATGGTGTTAGGTTATGCGGCTAATTTGGCAGAGTTTCACGCGAAGATTCCGATAAAGGACGCGGTGATCGCGGTGCCGCCGCACATGGGACAGGCGGAGCGGAGAGGGTTGCTTGCGGCGGCGCAGTTAGCGGGGATTAACGTTTTGTCTCTGATAAACGAGCATTCCGGCGCGGCGCTGCAGTACGGGATCGACAAGGACTTCTCGAACGAGTCTCGGCACGTGATCTTCTACGACATGGGCGCGAGCAGCAGCTACGCGGCGCTCGTGTACTTCTCAGCGTACAAGGGGAAGGAGTACGGGAAGAGCGTATCGGTGAATCAGTTTCAGGTGAAGGACGTGCGCTGGAACCCGGAGCTCGGTGGCCAGCATATGGAGCTTCGGTTGGTCGAGTATTTTGCGGATCAGTTCAATGCACATGTTGGAGGTGGAATCGATGTCCGGAAGTTCCCCAAGGCTATGGCTAAGTTGAAGAAACAGGTTAAAAGGACTAAAGAGATTCTTAGTGCTAACACAGCAGCTCCTATTTCAGTTGAATCGCTTCTTGATGACGTCGATTTCAGGTACGTGGATTTTAAAATTGTATTCCTGATTTTTATGAGTTTGTATGTTATACTCTATGGGACTGAACAAAAGTTGGCAGTTTGTGATAAGAATCATGGTGCAACCTTATTGTACACTGTACTTTAACATCCTCTTTAGCTTATGAATACTTTATTAATTTATTAATATTATGAACGAGAACAGCAAGGAAAATAACAACCAATTTTTATCCTATATATTATCATGGCAGACTAATATGGGAAATATTTAGTCTAAAAGAATTTTGTACTTTTTCTTTTTTTGAAAAAACCAATTCAACTGATAATATTTCTAGGAACTAGGAACACATTTTTTTGAATTGGAAGGAGGGATGTTTTGTCTTTTCAAAATGATTTTTTGAAGGGAGTGAATTAGCATTTTGTCTCATTTAGTAGGCATACTAAACCCCTCAGTAACTAAACTTACTATTTTTTATGGGCCAAGTTTTATGTCAGTGATATGATTATTGGGTATGCATCCATATGTATCCAATAAGTACTTGCGTTGGAAACATATCCACTATGGGTATTGGAATTGGATAAGAATACCCATGCATCATAGCTTCCCTGTAAAATAATTACAGCAGCGAAGGCAGGTACCAAGAAGACAGTTTGCTTTTGCTTCAACTGACTGCCTGAGTTGGTAAAGTGGCACCAGTCTTGGGAAATTGTGATCTGATCTGCTCCAACCCTTCAGTAAAAGTATGTCCTAGAATTTTCATTGGGTAAAAAGTAGTTGGGAAAATCAGAAAAAAAGGAATTGTTGATAATAGCATTTCCCATTAAAAAAATAGTTTAGCTTACATATGACTGACTAGTAGTTGTTTGCAATTAATTAGCAAGAAGGAGCCTTAGGTTTTACTGGCTGGCTGAGAGTTGAGAAAGGGGCATCAGTCTTAGGAGTTGAGTTACATATCCATACCAATATATAAAGGTGATACAACTATGTATAACTGTTTTTTTGGTCTTTACATTTTTAGACCATTTTATCCTTAATAATATTAAAGTAGTAACAGTAAAGTAATATAATAACCCCCACTGCGTACTGCCCCCACCTTATCACAAAAACTGTCCACTAAACCTTTTCTCCCTCAAATATTCATGTGCCTATATATACTCCTCTATACCGTATTCTTCTAACTTTTTCCGCAAGGAATGTTACTAAGCAGTTTGCAAAATAGACGTAAAACTGAGGTGGAGTAGTGGAACAGTTATTTTCCAAAGAGGCCACAACTGTCTCCTTCTTCCTTCTTTCAAATTAAGTCCCACTAATTTGTCACCGTCTGCAAATGTTTAGATAATCTTTACTTTCTACACATATTTTTTTATATTTACTTTATATATTTATCCATTTCTCTTATTTTTATTTTCGGCACATATCTTACTTTTAAATATATAAAAAAATCCTTAGAATTGAAGCATATACACTCTCAGAGCAAAGATAATTAAAGATCAAAATAAGAAGAAAAACATTCAACACGTGACATAATAATGTTACCATTTCAATATTTCACAAATGCATTTATCAAATATAATGATATTCAAGTATGTACCACAAAGGATTTGATCATCTGTGTATAGATAGGCATGTATTTACAAACATAATATATTAATTTATCATGTTATCATAACAAAGAGACATAAGAAGTTAAATTATTTATACATTAGAAAACATATTAAACTAAAATTAGTATGCCCATCAATTTACATATAATAAGAAATATTATTTCAATATATAAATTTTAGCACAATAAAATATGATAGCGCAGTAGAAATGTGGACACACAAGCATGACAGCGCCTGTGTTTCCACTAGTACCCTTAAGGACAGTGCTGCATATTGGTTAATAGATAGGATTGGCTGGTATCTTAAATCTTATGGTATCAATCTGAAGGAAGTTTGATGTTTTCAAATGATTAGGAAATCTGAAAAGGGGAAGTCAAGAAATACATCTGTAGAATGAAATTCCCTTACATGCCCAAGCTTTTTAAGTGAAATACTTAGACTAATCTCAATAAAATGACAAGGAAAATAAAATTTTGCTTGATTATCAAGTTATGATATATTTATACAATTGTACTGCATATAATGCTTAAGACTCCTTCACACAAGGCTTCCTTTCTTACAGGAGCACAATAACCCGTGAGAAATTTGAAGAGCTCTGTGAAGACATTTGGGAAAAATCACTCTTACCTGTGAAAGAGGTGCTTGAGCATTCTGGCCTGTCATTGGAACAAATATATGCAGTGGAGTTGATTGGAGGTGCCACCAGAGTGCCAAAATTACAGGTTTGATTAATATTCTTTTTCCTTCGTCAACAGTGGTATTTGATCTGCAGATAGCTGCATGATATTGAACTCCATGCTTGATAGTCAACAACATTTGTTTACCATACATATACCATCTGTTATGTACATGATAAGCCTGATTTTGTCAAATTTTTTATTCATATTAAACTATACAACTGTATCTGTATCCTATAGTTGTATGCTGTCTGTATACTGCAGTTGCTCTGCTAGTTGTTAGCTGTTATACGCAGCAAGTGCACACTCATCTGTTATATAACAGAAGTAACTAACGAATCAGTTTAACTGTTCAGTTAAATAGTGATCAAAACTCAAACTGTTTTGTTACAACTCTGTTCAATTGAACTGATTCAGTTGCAACAGTTTAGGTTGCACAGTTTCCTGTACATTTAGATTTGCACTCAGCATACAAATCTAATTCTTTTCTTGTACCAGAAACACAGTTATACCGAAAGAATTTCAGGCTCTCACTCAATTTTCTGTCCTTTTCTCTCATTCTCTGTTCACTTGAACTTCAACAATTCTATAATCTATATGTCAATCTTAGTTATTCATGCTGTCGAAGCTTGACCCTATTCTATGTGAAGTTATGCTTGCTACTTTTTTTTTTATTGATTTGAATAATGAAGAAAAAGGAAGAGCTCTACTGATGACTCAAATGTGAGCACAAAGATTTCGATGGAGAGATTGACAACATGAATATATTAAATTGCATAACCTCTTTTATTTCTAATATGAATATTACGAAAAAGTTTGAATGTATGGAATTCATGACGTATTTATCAACTTATATTTGGTATCTATTTATTGTCAAATGGAATTGCAGCACTTTATTTTGTCTATGTATTATATGATATAGTGTTTGAGCTTTAGTCTCACAGATGAAGTATTTTACTCAGCTGTCCATCATTAATGTATAGAGGTTGGTTTATTGTTTTTCTGTTCATATTATTACAGGCTAAGCTTCAAGAATTCCTTGGGAGAAAAGAACTTGATAGGCATCTTGATGCTGATGAAGCAATAGTTCTTGGTGCAGCTCTGCATGCTGCAAATTTAAGTGATGGAATCAAATTGAACCGCAAACTAGGAATGGTTGATGGCTCCTTATATGGATTTGTGGTTGAGTTGAATGGCCCTGATCTTTTAAAAGACGAAAGCTCTAGGCAGATACTTGTACCACGAATGAAGAAAGTCCCGAGTAAGGTAAATCTATTGATGATTCAATGCTCAATGTGACTACATATTATATGAAGAATGAATTTATCAGTTATATTCTCCAATTTTATTAATCCATTTTACCATTGTTTACATACCAGATGTTTAGATCCGTTAATCATAACAAAGATTTTGAAGTTTCACTAGCTTATGAAAGTGATAATTATTTGCCTCCTGGTGTTACCTCTCCTGAAATTGCTCAATACCAGATATCTGGTTTGACAGATGCAAGTCAGAAGTAAGCATAAAAAAATTCCTGAAGTTTTGTTGGCATATTGCATATTTAAGCCCCTTGAGGAATTGCTTTTCATGTAAAATTTAGAATCTCTAGTGCCCTTCTATTTATGTTTCTCCTCTGTACTTCTAGATACTCATCTCGGAATCTGTCATCCCCCATCAAGGCAAACATTCATTTTTCTCTTAGTAGAAGTGGAATTCTTTCTCTGGATCGGGCAGATGCTGTTATTGAAATAACAGAGTGGGTGGAAGTTCCTAGGAAGAATTTGACCATAGAGAATTCAACCATTTCATCAAATGTTTCGGCTGAATCTGCTGCTGGTAATAGTACTGAGGAAAACAACGAAAGCGTGCAAACTGATAGTGGGGTTAATAAGGCATCCAACATTAGTGCAGAGGAGCAAGCTGCCACTGAGCCTGCTACAGAGAAAAAGCTGAAAAGGCAGACTTTTAGGGTACCATTAAAGGTGAAAGCTAACTGACACCCTTATTTTTGTGACTATTTAAACTGACTGATGATTTTTGGGGGGGAAATTGAAATAAGTTCTAATATAACATGCTTCTTTGTTTTTGCTTTTTGCTGGTGGCAGATAGTTGAGAAGATAACTGGATTTGGAATGTCTCTATCACAAGATTTTCTTGCTGAAGCCAAAAGAAAATTACAAGTACTAGATCAAAAAGATGCAGACAGAAAAAGAACAGCTGAGTTAAAGAATAATTTAGAAGGATATATATATACTACCAAGGAAAAGGTTGGTAGAAAACAATACGTTTGTTATGTTAACAGCCTTCATCTATTATGTGATGGGTAAATATTTATCCTTTAAAGACTTTCAATTTTCTTTCAGAGATTCATTTAAAAGATTGACCTTTAATTAGTTGACTTTGTCTAAGTTTATACTGAGGGAGTTTAACACTTGGTCTTGATTATGGTTTTTGAGAAAAAAACCATTAGAAGAGAATTAAGAAATGAAAGAATGAGAATGAGCATTTTTCTAAACCCAAAGTTCCCTAGCCTGCAGCCAAATTAACTTAAGATAATAATGCCCCATCCATTGATCTTCCTTTTTATTCTTGTCCTTGCATTTAGCATCATCTGCATTTGTTTGGAGTATTTTTCTCATTGCCATGTATTTTCATTGGGATTTAGGAATAATGCTTCTTAAATTATTTTATAGGTTAACTGACTGTATAAAACATTATGCCTGTTGTTTTTCATTTTAGAATGTTTAAAATGTACTAATTTAACTAATTGTTTTTTCTAATTGTTAATATGGCTATTTTATTCAATTTGTTCTTAATGCTTCTCATGTTTTTATCAGATTGAAACGCTTGAGGAGTTTGAAAAAGTTTCTACAAGTGAGGAACGCCAGTCCTTCATTGAGAAGCTTGATCAGGTTAACCCTTTTCCTTGGGTAACTGAAAAGAAATGTTATTTACAAGAATATTGGCTTAAGCTTTTTGCATTGTCAGGTGCAAGATTGGTTGTATACAGATGGTGAAGATGCCAATGCAACAGAGTTTCAAGAGCATCTAGATCAGTTAAAAGCTGTTGGAGATCCAATTTTCTTCAGGTCTGTTATATTCAGTATAATTATCAATTTTACTCTGCATGCCTTTTTAGTGGTAAAGTTTTTCCTGAATTGGTTAATATCTGTTTCAGGTTAAAAGAGCTTACAACTCGGCCAGCAGCAGTTGAGCATGCTCATAAGTACATTGATGAGTTGAAACAGGTATCATCAACAAACTTTTCATGTAAAGTGCTTCAGTGCTTTTTATTTTCTTCATGTTCTTTTTGAAGCCGAACGTTATTTTTGGTAGGGTTGAATTACGTCAATTGATCCTTGTTGCTAAGCAAGCACTCCTAGGTTGTGTTTGGATGAAGACCTTTAAGATTTCAAGGAATTTGAAATGCCTGGAATTTGAATTGCTTCGATTTAAATTTCCTTATTTTTTAAATGTTATGTTTGGATAAAGTAATTCATATTTTTTAAGTTCCAAATTTTCACCATATGCAAAATTTCATTTTTAACAAGCAAGAGAGAGGGGAGAGAAGAGCAAGGGAGGCAACAGAGATGGGAAAAGGGAGAGAAGGGGATGAAAGGGGAGAAAAAGAGAAGAGTGAGAGAGGAAATAGAGATGGGAAAGGTAGAGAAGAGAAGAGAGATATGAGAGAGGGGGAGAGAAGAAAAGGAAAAGAGGGAAGAGAGAGAGATGAATAGATAGAGAGAAAGAGAAAATGAAAATGAATTCAAATTCTTACTTTTATGGTTGAATTTGAAATACTATAATTTTATATAATGATAATATTTTCACAAATTACTAAAAATTCAAATACTTTTTAAATACACTATCCAAAAAATATATATTATAATGCAAAAAAAAAATTAATTCTCACTTAAATTGGATTAATAACTTAAATTTCTCTATCTAAACACATTACTAGTGAAATAAGGCTTGGTGAACATTGTTGTTTTTGTTAATCCCTTTGATACATGGGTAGTTTATTGTTGTAAGCTAAAGTTGGATCAGTAGGCGCTGAAATATGATAACAAGATTGTCTTTATCATTTCTATTACATTTTAGCATCTTTCATTTGAAAGAATGTAGAGATTGGTCATCTGTTGCCTTTGTTGAGAACTCGAGATATTGGATATCGAAAGGGACAAGGTTCAAATTGATCTGATACAACAGGCAACTTTCATTGAAGTGGTTATCTGTTGTTTATCCAGTTTTCATCATGTCAGCACTCATGTGCCCATTCCTAGAAAATCATATGGACACATTCACATCACTTCAAGAAATCATATAATCAAAATACAATGCATTAAAAAGGTGTTAGTTAGGTGAACCAAACATTTACCATGAGCAATTGATTGGCTGATAGATATCTGAAGCAGTTTATTGTATATTGTGATTTATAAAATTATCTATTCATCGTCAGCCAGCTATTTCTAATGTAACTTGAAACATTTATGACATCTACAGATTGTTCAAGAGTGGAAAGCAAAGAAGCCTTGGCTTCCACAAGAAAGAGTAGACGAGGTACGTGGTTTAACTTGATTTCAATAACTTTGAATCTATTAAAGCTGGTGTTACCTTCAGAAAAATCATTGTTTTTTGGTACCTAACTTGCAAGGTCATAAAAAGTTCTGAAAAATTGAAGAATTGGTTGGATGAGAAAGAAGCTGAGCAAAAGAAGTAAGTTCTTGTTGTTATTGAAGTTTGTTTTTTACCTTGTCATTTCAATATTATGTGTGAACTTTGCAAGAGTCGAAAGAAGTATCTTGATGAGCAGCTGCCTGTATAAACATGTACCTCTATTTTTCTTTAACTAGATATAATATTCTATGAAAAAGTGCAACCTTAATGTTATGAATCTAAGTTATCCCTTGAAATGGCTTTAAAATTTTAGATTATTTCCCTTTTTGAGATCATATTGTACTTTTTCATTTATGCACTTCTGAATATTTACGAAAATGTACCAGTCTAGCTTTGTAAAGTATTATTCACTTTTTCACTTTGCTCACTACCTACTTGAGTTTGATCATTGAGGAAATTGCAATGTGCACGCAATAGTTGTAATGTAACTGCCTAACTGGTTATTAATTTCTTATTCTGTACTTGTTTTGAAGGACTTCTGGATTCAGTAAGCCAGCATTTACATCTGAAGAAGTATATCTGAAGGTGCTTGATCTGCAAACCAAGGTACTCGTCTCTTCTACTTGTCTAAATCACAAGACAGTGTAATTGCATGTGATTTTACAGGTCTAAGAAAGCAGTGTCATTTTTTGTTGATGTACATTTACAGGTTGCCAGTATTAATAGAATTCCCAAGCCCAAACCTAAGGTTCAGAAGCCTGTAAAGAACGAAACTGAGAGCAGCAGTGCGCAGAATACAGAGACTTCTGATTCTAACTCAGCTGATAGTTCCTCTTCAAGTGATTCATCTGCCAACAGTTCAGAAGGCACAAGCAAAGAGACGGTTACTGAGCAATCTGAAGGTCACGATGAGCTATGATTAACTGGGCATGCATGTAAGGCCGGTTTTAATCATCTACCTGCAACGTTTTTATAGTCAGAGATGTAGACTGGTAATGGTAGAATTAATAGGTGAAAAGGAGGCAGTATTAGTTCTCAGCGAGCAGGAGAATAGAGTTGAGGCAACATTTGCTGCTTGAGTTCTAAAATTTTGAGGTTAGATGGCTACTACTGTAGTATAGATTATTTATTTTCCTTTTATATTTAAGGGGGAAACTTGTAATTTGCAGTCATATGAGAACTTGTATTTGCCGAAACGCAATGTGATAGGGGAAGGAAAAAAAAAACAAAAACTCTCGAGATTTGATTTGCTCAGAACATGATACGGGAATTTTTGCATGAGTCACTCCTGATTCTAAACGATTTTTGTTTTTAATCAAATTAGCAGATACATTTTATCTTATTTTTAAT
