# Supplementary material for: Genome-wide analysis and expression profiling under heat and drought treatments of HSP70 gene family in soybean (Glycine max L.)
Source: Front Plant Sci. 2015 Sep 25;6:773. doi: 10.3389/fpls.2015.00773 (PMC4585176; doi:10.3389/fpls.2015.00773)
Supplement: Supplementary file 4 [file DataSheet4.DOCX]

>>Glyma01g44910 chromosome:V1.0:1:55381508:55383508:1

AAATTATATTACATTTATTTAAATTAATTAAATAAAATAATTACCAGGAAAATATCCAAA

GCCAGTTTTTTTTTTCCCTTTTTAGCTGAAAACTGAAAATAGTTTTTAAAAAACAAATAA

CACATTATTTAAAATTTAACAATTTGTTGGATATGTATATCTTTTACTACATGCTAGAAG

TGTCCAGACAGAGTCCTGTGGGTAACGTGGCTTAGTCCAAGGAACGCATGGTATGGTATC

TCTAATTGTTCTTTTCTAGAGTATTCTTTTTCTGATTTTGATCTTGGAAGCAAGGTAACA

ATAGCAAAACAGTTTATTCACAAAATTATCTACTTCAAAAAATGTAACCAGAAGTCCAAA

TTGGTATTGTTGACTATATTTGATAGCCGTGAATTATATAAAGATGCATCATTGTTTAAG

AGTGTTTAGTTTTTTTTATATTGAGAATGAGACTTGAAACAGACTGATGGAGTAAAATAT

TCCATTAGTTAGTGTACATCTTCAAGTTTTTTTATTTTTTGCCTGATTTATTGTTATTGG

CTGATTCTGCTGGAGTGAACTTGTATAGAGGTTTTTAGATTTGAAGGACGGTCTCTAATA

TAAAAAAATTGGTATTTCATTGATTGAGTATTTAGTTATCATATTATTAATTAGAAATAT

TTATTTATGGTAGTACTTTACAAAATAAATTAGTTCAATATAAAGACATAATTAGATCGT

ATAAAAAATTCATTAAAATGCATCCAAACTAAACTAAAAATTAAAATTGAGTAAAAATTA

AACTAAACTAAAAAAATTGAATAAAATTTAAAATTTGATATAGTACAGTAAATATAAAAA

TAGAAGAGGTTAACAGTTATCTGTTGTTCAAATTCAATGTAGACAACATGTTCAAATCTA

ACAGTAATCCACAATCCATCTGTTTAACCTAAGCATTGGGCTTTCTCTCAGTAGTGTACT

TCCATTTCATTCTGAAGTTTGGATCAGTGGACCAAAAAGACTATAGCTGAAAAACGAGCT

TATTAACGAAGGTAAACTCCACATTCATGGAAAGGACACGGCTGAAAACATGACTGCAGC

GAGCGAAACTGGAAATGTTCCTGAACAAGGATGTTACTGTAAATTGACTCCTGACTGAAT

TCAGCAAATTAATAACAGTTAGAGAATGAATCGCTCTCTATATGCTCCTGAACTATGGAG

CTTTTGCACATTTTGGCATGGCAGCATAAGCAAACAAGGAAATGACCATTGTGATTTTGC

TCCTCACTTGCACAGGATATATAGGCCAGTACCATAGGAATAGCAATTTTGCTCCTCCCT

CAATCAATTAAGCATGTCTTTAGCCAAGTAATTTATTTGCCTTGATGTAAATACTTAACT

TTCTTTTAGTTTTCAGAAAATGTATAGTTTATTTTTTTTAAGTTATTTCTATGTATCAAT

AATGTAAAATAATTTAATATTATTATCCAATCATAAATTACCGTTTAAATAAATTATTTT

ATAAATTAATAATGAAAATTCATGCTTATTTTATGTTAAACAACTAATTAATGATTCCTA

TTTTTCCACATCTTCTCCCAAAAAGAATTATTTAATTTGAAACCAAAAACATCTCTATAT

ATAATAAAAAAGAGTTGATATTGTTCTTTGGTTGTTTAAGACTAAAACATGAATGTTTCA

AATATAATTAGTCATGCCTTAGCATGCATGCGTAACCGGAAAAAAAGAAAAGCATGCCGT

AGCGGTTAGATTAACTGAAAAAGGGTATATCGTGGTGTGAGGAAAAAAAAGGAGGGAGTG

AAAGAGAATAGATGAAAAATTAGATTAAAATATTTAAATTACAGCTAAGTAAAAGAAAAA

TTATTATTTTTTAATTATTTTCATGGATGCGTTATTATTTGAAGAAAGAGAATGTACTGC

TTCCATAAAATACATAACCTCTAAATCTATGATTTTTTAAAAATATATCTATTTCCAACC

AACTTACCACTTCATGCACAT

>Glyma02g09400 chromosome:V1.0:2:7363311:7365311:1

TTGCACAGAAACGTGACACGGGGTGGATTAACACGCAGCACCCAATGACAGTTAGTCTAT

GACAGTGGCGCTTGGGAGTAGTAATCTTTCATAGGCATCATTCTTTTGCCCTTCTCTGTT

TTTTCCCTTGTGTTTTTGTCCAATTGAGTTACTGCTGATATTAGCCATGGATTGGGGATA

AGAAAACAATTGCAGGATTCGGTATGCATCTTCTGACCTAGTGTTACGTTCTCTGCTTAG

AGAGAGAGAGAGAGAGAGATCGGCTATGAAGGCAGCCAATGGAAGACAATTTAACTTGGA

CTAACCTTTGACTCTTTCAGCATTTCTGAGTGGTGTGGTTGTATAGGTAGCTTTATCCCC

TCCTTTACTCCTTATAATTTGTAATACGCAAATAGTGTGATTGTGATGGTTCAGGCTTGT

TCATATGTACTAGCGGTTGATCCGTGACTGTCATGCACGGAGTATATGTTTGGTTGTGAA

ATGATATCAATTCACAATGTTTCTAATTGTTAGAGAAAACACCTTGAAAATAATTCTTTT

AAATAGTTATTGATTATAATTTGTGGGTTGATCTGTCCAAATACAATTTTTCATAGAAAA

TAAATGCAAAAATTTTAGACAAATTATTGCTGATAAATTTGAGTGTGATAATTAATAAAA

GATTATCAAAATTTAATGAGTATTTAGGTTAATTATTGTCTAAGTTAAATAGAGTCTTCC

GGTAAAAAGAAAAAGGAAGTATAGCACTCTAGCTCCATTAATTGAGTAACACGTAAATTT

TTTTATTTGTCTTTTATTTTTGTGATAAAAAAAAGATTCTCAGAGTAAAATTTAAGTTCT

TTATTTATTTATCTTATAATTAAAACTTCAAGTGAATTTTTAATTACTAATATATTAAAA

ATATTAGGGTGTTAATTTTTTTTGGTTGAAATAAGGATGTTAATTTTAAACTGACAGATT

AAAAATATAAAGAATAAAATTAAACTTTTTATAGTATAAAAAATTAAATTAAATCTTTTT

TATTCTACAATCAAACTAATTCTAATTGAAAAGAAAGAATTAAAATATAATTTAACCTTT

TACGAATTAAAATAATATTTTATAATATTTTTTATGAAAATATTTTATAATATATATATA

TATAATGCTAAAAAAGTGTAATTTTCTATATTTTTGTGATTTTTTCTCACTCGATGCTTT

TTTTTATTTTCATTTAACCATTCCTTTCAACGTAAACAACTCCTCAAATATACATTTTTT

TTCCCCTTCCTATTTCTACTCTTTGCACACTACACACGTTTTCTTCGCCAACCTTTGGAC

TCTAGTCACTTTTTTTTTTTTTTTTTTGTGTGTGTGTGCTTACGATCAACACCAGTGCCG

AACCCTACTTACTTATCATCCCTCCTTCGCAGCAGACACGGGTAACACTGCTTTTTGTTT

TAAACACACCGTTTTGCTAACAAAACAATACTCTAAACCATTTTAATACAAGTCTTTTCT

TTTAAATCTGAGAAATTGTGAATCCATTTAAGCTTTTGGTCAATGGTCAATGTAAGTTTA

TATTTTTTTGACTTTAGATTCCTGTAAAATCTTTTTTCATTTTTTTTAAAGTTTATGTTT

TTTATTTTTGATTCTTAAAAGATACTTTTGTTCGTTTTTAGTTCTGGTAAGTTTAAATTT

TTAAAATTTTGATTTCTGTAACACAAACTTAATGAGACTATAAATAAAAAAGTAAAATTT

TAAAAGAACTATAATTAAAAAAACATAAATTTACAAAATTAAAAATTAAAAAAATATCAA

CTTACAATAATTAAAATTATAAAAATAAATTTACATGAATAAAAAATGAAAAAGTATTTA

CCTGGAACAAATCATATTTAATTTAAGCCTATTAACAATTATCACTTTTTATGTAACCTT

TCACAACTTGCTATTGCCGGTTACATTTACTCAGTCCTTTTGTTTTTCCAACTTTTGGAC

ATCTCTCCAATCAGAGTGTGC

>Glyma02g10195 chromosome:V1.0:2:8077000:8079000:1

CCCTCAACCTCATAGCAACCCACTTATACATCTAGAAGATCCATGAGACAAATACATAGA

CCAAGTAAGTATAAAGACTTCCATGTCACATACTCACCATTAACTGCTGCCAGTCACTCT

ACAGGTACTAGCCTTTATCCTCATAGCTCTGTTCTTTCCTATAACATATTATCTCCCTCT

TATCACAATGTTATTTCATCTATCACACAAGATGTTGAACCTCAGTCATATAGTGAAGCA

TCTAAGGATCCTAATTGGATTCAGGCCATGCAGAGATCAAAGCATTAGAGCTTAATGATA

CCTGGATTCTTACTAACCTTCTTAAGCACAAAAATGTAATTGGTTGTAAATGGGTGTACA

AGATCAAGCACAGATCTGATGGTTCAATAGAGAGGTATATAGCCAGGCTAGTGGCTAAGG

GCTACACTCAAGTGAAAGGACAGGACTATCTAGATACTTTCTCTCCACTAGCTAAGTTAA

CCACAGTGAGACTTCTATTGGCTTTAGCTGCTATTAATCAGTGGCATCTCAAGCAACAAG

ATGTGAACAATGCTTTCTTACATGGTGATTTAAATGGGGAAGTGTACATGACTATCCCTT

AGGGTATGCAAGTGGCTAGACCAGGACAAGTTTGCTAACTTCAAAGGTCTTTATATGGCC

TAAAACAACCTAACAGGCAATGGTATGCCAGATTATCATCTTTTCTGATTTCCCATGGAT

ATAAACAATGTGCTTCTGACCATTCCTTGTTTATTAAGCATGGTTTTAACACAATTTCTA

TTTTACAAGTTTATGTTGATGACATTGTCCTATCAGGCAATGATTTGTCTAAAATTCAAA

GGATTACACATCTTCTAGTTAGTGCTTTCAAAATAAAGGATTTAGGAGATTTAAGGTACT

TTCTGGGTTTCGAGGTAGCCAAAAGTTCTATTGGTATAAACCTCTGTCAAAAGAAGTATG

CACTGGATATTCTCAGTGATGTTGGCATGCTTGGTTCTAAGCCAGTGTCCACCCTTTGTG

ATTATATTACCAAGTTGTATCAACACCTAGGGTCTCCTCTTTTAGTAGAGGATGTTTCTT

CTTATAGGAGATTAATTGGGAGATTGATCTATTTGACTAACACAAGGTCAGATATTACAG

GTGTTGTGCAACATTTGGGCGAATTTTTTGCTAATCCCACTTCAAATAAAAAGCAAGCTA

CTTTTCGAATCCTTAGATACCTCAAAGGGACTTCCAGGTGTAGGGATTTTTCTGTCTGCT

GCTAGTAATACTCATTTAAAAGGTTTTAGTGATTCAGATTGGGCTGGTTGTATTGATACT

AAAAGATCTATCATTGGTTATACTGTGTACATCGGTGATTCATTAATCTCGTGGAAGTCC

AACAAGCAAGGTACTGTATCCAGAAGTTCCTCTGAAGCTGAATATCGGGGTTTAGCAAGT

GCCACTTGTGAGCTACAATGGCTTACTTATTTGATGGAAGATTTTAAAATTGATTTCCAG

CATCTTGTTGTGTTGTACTATGATAACAAATCTACTCTTCATATAGCAGTCAATCCTATT

TTTCATGAGAGGAAAAAACACATTGAAATTGATTGCCACATAGTTTGAGAAAATATGCTT

ATTGGTCTTGTGAAATTACTTCCTGTTTCTTCTGCAAATCAGTTAGCTGACATCTACACT

AAGGCTCTCTGCCTTGTGCGTTCCAGTTTCTGTATTCCAAGATAGGAATGTCTGATATCC

ATTCCTAGCTTGAGGGGGGCTCTTAGCATAGATTACTATGTAACTAGAGTTAGATTAGTT

GGTTAATCTCTATGTAACCAGAGTTAATGAGTTCGTTATGAGTTAGTTACTCTTGTAACT

AACTATATAACCAACTGATATAACTAACTTTGTAATCTGATTCATTCTATGCAATTCATT

GTTTCCTTCATCTCTTTTTCTCTCTAACTTCCTCTGCAAGCTTCTTCTGCAAGCTTTGCA

TTCATTCATTCCTGGCAAACA

>Glyma02g10261 chromosome:V1.0:2:8153483:8155483:1

GTCCAGGACATCCTGCCCGAAAATACTGGAGTTGCTGCACAATGCACAAGGCAAGATAAA

AGAAGTGAAGCTGCAGGATCCACGATGTCGGATACGATGTCCAGGACATCTGGCCCGAAA

ATACTGGACACATAAATCTGTTATATCTTTAACAGATTATTGTGCAGTTAGCAACAGATT

AGACGATCTATCTCTAGGAACGAATTAAAAGATAATTAAAGTTCGAATTACAAACTTGAA

TAGTTCGTTCAGGGATTAAAGATTAAAGATAAAAACTAAAAGATCAAACTTTATCTTTTA

GATCTTTAAGTGCAGATTTTCAGGAGAATGATAGATCTCATCCAGCGCAAGCTGTTGCAG

CCCAGATACGCACACTGCTATATAAACATGAAGGCTGCACGAGTTCTGTACCAAGTCCGG

GATTGAAGAGTTATTTTGTGAGTTTTGGGACTTGAGTGTTTTGTGAGCCACCTTGATGTT

ACTCTAACATCAAGTGTTGGACCTGAGTGTGTAGAGTTGATCTCTATAGTGTGTGGAGTT

GATCTCTATTGTTCAGAGAGCAATCTCTGGTGTGTATTTGATTTAATTGTAAACACGGGA

GAGTGTTTGAGAGGGAGTGAGAGGGGTTCTCATATCTAAGAGTGGCTCTTAGGTAGAGAT

TGCACGGGTAGTGGTTAGGTGAGAAGGTTGTAAACAGTGGCTGTTAGATCTTCGAACTAA

CACTATTTTAGTGGATTTCCTCCCTGGCTTGGTAGCCCCCAGATGTAGGTGAGGTTGCAC

CGAACTGGGTTAACAATTCTCTTGTGTTATTTACTTGTTTAATCTGTTCATACAGTCAAA

TACAATCTGCATGTTCTGAAGCGTGATGTCGTGACATCCGGTACGACATCTGTCCCCAGT

ATCAGAATTTCAGGAAGAACACGTCTGGATTTGTGTTTTCATTAGGAGCCGGAGCAGTTT

CATGGTCTTCCAAGAAGCATCCAATGGTAACATTGTCTACAACAGAAGCAGAGTACATAG

CCGTTGCATCCTGTGCATGTCAATGAATCTGGATTAAAAGAATATTGGAGACCTTTGGTT

TCAAGGAACAAAAAAATATTTTAGTTTTATGTGATATCAATTCAGCTATTTAGTTGTATG

AAAATCCAGTATTTCATGGAAGGAAGCATCAAGATGAGTTACTGCAGCACTCAGAATCAA

TTGGCTAACATAATGACTAAGCCACTCAAATGGGAACAGTTCTTAAACCTTAGAAGCATG

TTGGGCATGATTGAAGCATCAGAAGTAAACTAAATGTTTTTTTTGGCATCTAGTTTAAGG

GAGGGAATGTTAGCAATTAAATAACTTTGTTAGTTTCTATTTTTTGTTTTCAAGTATTAA

GTTGACCTGTTCTTTAGGGAGTGAGAGTTAAAATTTAGGAAACCACCCACGATTTGCGTG

GAGGTGGGACATTTTTTTATTCTCGTATTTCCTATATAGATGTGATTCATCTTCCAGTTT

GAATAATACAAACGGGTCCAGAAATTTAAAAATATCTTACCTAATTGTGTTCACATTTTT

TACCAGTAGTGTGTAGTTTGGTTATACAGCATTATTCATCCAAATTGAACTCAACCAAAC

ATGAGTTAGGGTTAGTCTTTCTTTTTCATATTAGCTAAAAGAAATGTAAAATGATATAAA

GGTGTTTAAATTAGTATAATTTTGGTGCGTTCTTTGATTTTGTTAAAAATTACGGACCAC

AATCTCGGAAGAACCTTGATGACATTGTGGTCAGCGTGGACCGTTTTTTTAAAATCTTAG

ATTATTTTATTAAAATGAGCTAATGCATATGTTGGGGAAATTATTTATAAAATATCAATT

CTTTTAATATACATCAATTAAGAATAGTTTTTTTTAATCATATGAAATAGTTGTTTCACT

GTTTTGTATGTTTATAGTGTAGAAGGTTTTTATTTTAATGTGGTGTGTGATTTGTAAGTT

GATGTTTGGTATGATTGTTGA

>Glyma02g10320 chromosome:V1.0:2:8184068:8186068:1

CAATTAAATAAGATGTTTGGATAAAAATTTGAAAAGAAATTGAAATTTAAGTATTTTAAT

AAGGGATTTTCATTAACTTAAATATTAGCATTTGAAATTCTTTCATTTTATGAAAGAATT

TGAAATTCTTTTGTACTTTAGGATATCTCCATGACACCTTCTCCCGAAAATAGGAGATCT

CCATGTAGAAATCACGCATGCGACGGAGAGCCACCACTAGGCGCGGCAGGTGGCGGCGCC

ACTTGGCCTAGGCGGCACGGTGGTGGAGCCGGATGAGGACCTGCACGATGCAGAGGCGCG

CAGGCAGAGCGCCTCCTGGAGCGGGTTCCAGCCAGCGGCATTGTGGAGGGAGATATCGAT

AGGGTTTTCTCTCTTCATCGTTCTCTGTGTCGCTCCGTTTCAATGGCTCAGCAAGACGCT

TAAAACGGCGGGTCGAAGAATACCGCAGCTGCTGCTACGGTGTCGTTTGTCGCTATGAAG

CCATAACTCCTCGTCGAAGCTCCTAAGGCCAACGTCGCCATTCTGTTCTTCAAGGTCGTG

TTTGGCGCTGAGGAAGTTGGTCGTACGCTCAACCCTAAGCACAAAGCCAAGCACGAGCTC

CCTCTCATACTCTCCGCAGAACTCAAAATCGCTGGCTCCACCATTCTCGTCACTGACCTC

GTTGATGACACTTCTTCGCCGTTAGTTTTCTCTCATATTCACGTTCATATTCTCTAGATC

TTGAATATTAACATTGCTCCTATTTTGTTTCTACTATTTTTTAAGATTTTACTGATTTTC

TCTGCTATTCGGTTGTTTAGGTATTTTGTTTTGTTTATTTTTTATATTAATTAGTTTATG

CTATTTCATGTAATCTTTTCTGTATATTTTGTTTTATATGCACCCGATTTGTGATTAATA

GAAAATACAAATGATTTTTCTTCTGAGAATCGTGATTTCCTTTTTGTCTTTATTACTCTT

CCATAGTTCTTGCTCTTACTTTTCTTGAATTTAATAAGGAGAAACTCTTTTTAACCTTTT

TGGATGTAGTACTTATTGTTCTTACGGGAAGAAATGAATGAAGAAGGAAAGGATCTAACT

CACGTTCTCTTTTTGTTATTTAAGAACAGTTCACCTAAATAAATATAAAATTTTAAAAGA

GAATACATTTGATTATTTTATCAAAATAACAAATTTTAAAAATGAAGAAATTCAATTTCT

TTATTCAAACACAAAATTTTGAAAATGAAGAAATTTAATTTCTTTATCCAAATACAAAAT

TTTAAAAATGAAGAAATCTAATTTTTTTATCCAAACACAAAATTTTGAAAATGAACGAAT

TTAAATTGAAACATTTGAAATTCTCAAAATTTAAAATTCTTTAAAATTTTAAATTTCCTC

GTCCAAACATACTCTAGAGAGAATGCATCACAATCGGGGACAACAAAGAAAGTATTTGCA

GCAAATTTTGCATTTTGTTGTTCCAGTAACTGCTGTGTCTCTATAGTCTATACAGCACGT

AATTTGTGGATTCAGTGGAGCAACAAATACCGAATACGACAACCGTGCGTACTTCAGTTT

TTTAATTAGCATTTGAAAGATTTACAGAGTGTTGAAAACAAGCTCGACCGTTTAGATATT

AGTAAGATAGGACTACTCTCATGGTTTAAATCTCAATATCAACTTGAAAATCTGGTTTAT

CAAATTAGATAATCTTAAGCTATAACTCAATTCACCTAAACTTTGATTAAATTTAAAAGC

CAATGTCTATAAGAAACTCAATTAACCAAAGATTAGGAGAGGTACTTCTTAAGAAAGGAA

TTATTTTCTTTAACCTTCATGATTCGAATTAATTAAAAGTTGAACAAATAATAATCCTCT

TGTAACATTTCTTTTTTTAACAAATTTCTAGCTTCATCATTCTACTAAAAGTCAATTACT

CGCGTGATAAGAAGAACCTTTTATGCTTCTAAACCAGAGGAACAGCGCAATTTGCGCTAG

TAACGTTATCTATCAAAGACC

>Glyma02g36700 chromosome:V1.0:2:42133636:42135636:1

GTCTCTTGGCAATCAAAGAAGCAATCAACCGTATCACGGAGCTCCTCCGAAGCTGAATAT

CGCGCTCTCGCTTCCACTACTTGCAAGCTACAATGGTTGACTTTTCTGCTTCAAGACTTT

CGCGCCACATTCATTCAACCAGCAACCTTATACTGCGACAATCAGTCAGCAATCCAAATA

GCCACTAATCCTGTTTTCCATGAACGAACCAAACACATTGAGATAGATTGCCATATTGTT

CGACAGAAGCTTAACTCGGGTCTCATAAAACTCCTTCCGGTTTCATCATCCCTGCAACTC

GCAGATATATTCACTAAGGCCCTTTCACCCACCATTTTTCAGCACCTTTGTAACAAGCTG

GGAATGATGAATATCCATTCCCAGCTTGAGGGGAGATCTTAAAAGTATAACAATATCAGT

TAGATTAGTTAGATTGGTTAGGAAAGTTAGTTTTCGGTTATAACAACCATGCATCACATG

TATATAAGTTTCCACCATTGATAAATAACACAAGAGCTGAAGCTGATCATTTTCTCTAAG

TTGTTCAGAACCCTCTTCTTCCCTTATGATTTCATGTATCATTAATAGGTATAAACCTTC

TAATCTGAAATGCCAAATTTATTATAATTGAACTTTGTCCAATACTTTATAAATTACATA

ATATCCAACGCGCTAGCTAAAAGAATTGTGGCCAAAGCTTCATATTTTCCAATTTAGTAC

CTATAATTTAAAACATGCTAATTTGGTCCTGATAGTTGTCTTTTTTTTTTTTAATCTAAC

CTGGTCCTTGCCATGAAATCATTTTAATGTTGTTTGTTTATTTAACCATGACATATTATT

GTTCTTTATATTGGAGTTCTCTCTTCTGCCCCCGGCGACATATTATTATTCACTCTATTG

TTTGTTGACCACAACCTTCTGCCACTGCAATCCACTACCACCCAAAATAGATGTCGTCAC

AAACTAAATAGAAGAACCACAACTCCACCAATATCATTCATGGTTTGCAGATGAAGATTC

ACACTTCTCTAAATAACTCCTTTCTCAATGTCATTGACACAAAACTCGTGCTATAAAAAG

TCACGTTTACAAAAATCCATAGTTAAAAGTCACGTTCGAACGAACGATATTAATTGTAAT

TCTACAACAAAAATGAATTTAAAAAAAAAATGTAATTATAAGAACTAAATTGACATATTT

TAAACTATAAATACCAATTCCTCTCAAAAGAAAAATTACAAGTACCAAATAAAAAATAAG

TGTAACTGTATGGTTAAATGCATTTAAACCAGCATAATTTTTTTGATGTAAGAGGATCAA

TTTCACATATAGCTCATAAGTAAATCCTTGTTCTACTCTTGCTATTGTCCTTCATTTTGC

AATCCTCATCCTTTCAACAAGTGTTTTAAAACCGTCATCGACAGCCCACGCACCGCCCCT

CTTCACTCTCTATTCTCTCCACGTGGACTGCTCAATCCAGCTTCCCTCCAAAACTCTTCA

ATTCAAGTTCTAGACGCTGTAGGAACACAGATGCTTACATCAGCTCCAGTATCATTTTTT

TACCCCAAACATCAAACAAGTTAAAAATTGAAAAACATACCATATAATGGGAAAGGATCT

ATGCCTAATCTTGATATGCATCTTTTATTTATTTATAGAAATCTACTTAGTTAATGTCTA

CCAAACATATAGATTCATGATTTGTATAAAGAAAAATTATTTATTATCTTTTATCTGATA

AATTTCACCAGAATTGTTAATTAAAAGTTTTAGCGCGCACTTTTACACTATAAAATTCAT

TCAAACTTTACTAAAATTATATATTAAGAATGAGTTTTCTTAATATACACGTCTTTGTTT

TTTATTTTATTTTTAGTGTATTAATAAATTATAACAACAAAATATCTTAAAATATATAAA

TATAACTTGTTAACATACTCATTAAATAAAATAAAGATATATATGTTAATAAATATTGTG

AAATTTGCTGCACACTCTTTC

>Glyma03g03250 chromosome:V1.0:3:3034627:3036627:1

TTTTGGAATTCAAATTGATCAAATCTGATTCAAAGAGTTTTGATTGAATCATTTATGTAT

TTGGTTTGATTTTTTATTGGGTCATTTAGGTATTTTGATCAAAATCGAACCAAATTGATC

AAAAAAATTTTTATAATTGTTTGATTTGACTTTAAATATTATTAATATTAAAAGTCCAAA

AATCACTAGTATTTGACCTTATTAAAATATTACTTTTAGAGAACATTATTATTTGTTTTA

TCTTTTTTCATATATATTTTTTCTGTCATATTTATAATATTAATTATCATATTGTAAGAA

ATAATGTGATTCAAGTTATTACAGTAAATTTTGTATTTTTTAAATTTATATTAGTCTATT

TTAAAATGTTATGTAAATGATATTAAATGAATCAATTTTATTATTTTTAGACATTTGAGG

ACTATTTTAACTGTATTAAATATTTGGATGAATTATGATATAAAATAATAATTAAAAATC

AAATTTAAATAAGTATGACATCATAAAAAATTTCATCATAACACCACCCAGAATCAAACC

CATTGTCAGTTGTTATAATGAAAGAAAAAAAACACACACTACACATAGATATTCTGTTGT

ATAAGCCCATGTGGGTCTCAATTTTCATACATAATAATATAATATTCTTTTTTTTGAAGG

AAATACATAATATAATATATAATACCCATAGTTTCTAACTTCAAATTAGATAAGAGGCTA

ATGTTGTTAAATATATACAAAAAATAACAAAAAAATGTTTCATGTTCTTAAATTCATTTT

ATTTTTTTATCTAAAAAAATAAAAAAATTAATAATTTTTGTTGTCTTTATTAATTCTTTT

TTTTAAGCTCCTGTAAAATAAATTATTTTTGCTCCCTCTATCTTGAAAACATTAGGCCGT

GTGTTTAGTGTCTCTTCTTTTTTCTATTTTAGTTGATTTTTTAATAACTATAAAAAATTC

TCATTATTATAACTTTATTTCATATTTTAAAGAAAATAACAAAATATATTATCACTGTTT

CTTAAAATTCTTAATTAAAAGCATGGAATATGTCAAAAAAATATTTTCTAATTATTAATA

AATATATTAATCAATTGAAAATAGAATCAGATAAGAAATTTGTAAACCTCAAGCTATCTT

TGTATCTTTTATATATTCAAGACTATGAGGAGATGTACTTGCATTTTTTTAGAATTGGAA

GAATCATAATGAGTAGGACGGCAATAGAAGCTTAGAGAAGGAGAATGGAGAACACCTAAA

ATTTTCTTAGATATACACACCTTCTAATTTTTTTTCGTTCTTTTTTCTGTAAGAATATAA

TTTTGACGACTATTAATTGCAAGCATAATTTAATATTTTTAATTTCTAGGGTTAGAAAAA

GAACGTGGTGGACATGGATTCAAGCATCTCTCCAGCAAGTAAGCATATGATAAAACATTT

TTAATATTAAATTGTGTGCAGGTAAGCATGTGATAAAACATTTTTAATATTAAATTGTGT

TAAAAAAATATGTTATATAAACTAATTGTGCGTAATTGTTGTATTCTAAAATCAAGAATT

GACCTTTTTTTTCATAGATCAAGAATCTTCTTTGTAATTAAAACACGTTGTTCAATTTCA

TGATTAACTTGTTTTATCAGAATTCTAAGATAAACTATTAATCCTTCTTTCAAAATCCTT

CTATTAATTAATTGGTAAATTCTTAGAAAAAGATGTAAAAAGAAATTCTTAGAAAAAGAT

TCGCTCTAGCTAATAGCAATAGCAAATCATTTGGGTTTGTTTGTGTTATGCATGCAGTGT

GAGAAATTTCTTAAATCTGGGGTACCTGGGTTTCATCATGGTTGCACGAAGGAACACAAA

GTTCATAATGAATGTACAGTTTGTTTGGTGACCCTACTCACAACACGTGTGAAAGGTACG

TGTGCATAGTCTCCCATGAGGGTATAAGTCGTTGGACAAGCACATAGATCAACAATAAGG

AAAAGAGTACCTTTCCCCATA

>Glyma03g17870 chromosome:V1.0:3:22493770:22495770:1

CATAGAGTAACAAGCCACTGGTAATCGATTACCAGTTATGTGTAATCGATTACACAGTGC

ATTTTACAGGTTTTCATGTTCTGAAGCTGTGTAATTCGTGTTTGGCCTCTGGTAATCGAT

TACCAAGGCTGTGTAATCGATTACCAGAGATGAAAAGCCTTGAGGTACACCTTTTAATTG

TGTGTAGTGGTTATGGGACACTTTGTGTTGCTATTGTAGCTAGAGCTCTCGTGAAAGAGT

CTATCCCTTTCCTTTTTATTTCTTGTAGATCGTGATGGCGGCGCAAGTAATCCATGATCG

AGTGGAGATGGAGTGCCTAGAGGGAGCTTGGGAGACCCTCGAAGGCAATGCGAGGTGCCG

ATTTCGGGGCACGATTCGATTCATGGCTACTTCATTGGTGCATCCAGAGGAACTCGCGCG

CACGCTTCAGCGCACTGTGGAGTGGATACTACCCACGCCCACACCATATCGTCTAGTGGA

GCCAGTCCAAGTGATCGAGGTGATGTCATCTGAGGAAGACCCTGAGGAAGACCAAGAGGA

GCTACCTCCTGAGCCTGCTGTGGATGCTCTTGACTTCCTAGAGGGTGATGAGGACCCACT

CCTTGAGGTGGATTCTCCCGAGGTCGTCATGTCGGTATCTGAGGCAGACTCTACGGAGGA

GAGCGGCCCTGGAGAGATGGCGACTAGTGGAGGCTATTCATCATAGTAGACAGCTCATTA

GACTAGGTTCATATACTTTTTGAGGGTGGGTGTATCTAGTGCTGACTGTTAGGTTTACTC

TTTTGTTTTTGTATGGGTAGACCTATTGTATAGGAATTTGATGATTGTATACATGTGGCT

GAAGCCACTACAGTGGATACTTTTGCCCTGGATGACACTATGTATTTTGCAAAACTCCCA

TATTTTGGACAGCTTTAAATGATGAATGTACTTATGTTTACTCTTGTTATTTGAAAAGAA

AGCATTAGCAAACTTTATTTGCAAAAGAGTTTATCGACCGTATTTTATTTTATTATTTTC

ACGTGACGACCTAAAGTAATGGCTGACATACCTTTCCTTCTGAAAAAGCAAAATATTTAG

AGGTTATGAGTGATCAGAAAGAGATGACTCCGAATAGAGTTGTGAACTGGCCATTCAGGA

CTCTATAGTGATGATTTTCCTTCTGAACTTAATTATTGTGAAAAGAGAAAGAAATGAAAA

AAAAAATAAAAATTTCCATGGTCTTTGTTGTTTAAAATTATTACTGTTAAACCAGTCATT

ATTTTGGGGATGCCACAGCCTGCCGCGGCTTGCCTACTCATTTTTTTCCTCCTCCATTTT

CACTTCCGCCCTTCAATCTTTCTCTTCTTCTTTCTTTCTTCTTTTACAATTTTTTCTCCT

CCCTTTCTCATTTTCTAGGCATGCCTCTTCTTCTTCCTTCTTCCTTCCTCTATCACGGCC

TCATCCTATGCCTCTCCTTCCTCAGCTGTGACCTCCTTCTTCCTTCCCCTCCACTATGAT

CACGACCTCCTTCTTCCTTCCTCTTCGTCGTGGACACGACCTCCCTCCCCCTTCCCCTTC

GCCGCAATCCCCTTCCCCCCTTCTGAAACTCCGTCACAGCCTCCTTCCCCCTACGCCGAT

ATGTGTATTTTTTTTTTTTTTTGTTTTTATGGTTTGTTGGTCTCTCTCCTGGCTGGTTTT

TCTTCTTCTGTTGGTCTCTCCTGCATTTTGATGTTAGGGACAGAGAGTAAAACAATTTTT

TTTTAATAATGTGTAGATCTTAAGGGTTCTTCTACAAGAACTCATTCTTCATGTTGGAGA

TCTGTCCAACTACTTTATTGGGAGATCTTATGAAAACCAAATTTTGAGCTTCCTTATTCA

AGATCTGACAATAAAATTAAGCGATAAAAGATAAAGTGTAAGATATATCTAATCCATATC

CCATTGATACTAAAAAATGTCTGTTTTTCAAAATATAAACACTAAAAAATATATTAAAAT

TTTTGAAGACTATAATATAAT

>Glyma03g32850 chromosome:V1.0:3:40588047:40590047:1

TTTAAAGTCGTTGCCGACTTAGCTTTGCCAGTTGGGTACGCATAATTTCCACGTTCGCGG

ATCTGAATTTTCTAAATCGTGGGCCTGACTCTTTCTTGATCCAGCCCAATAAGTCACCTA

ACTTTTTTTCAAATTTCTTTTTTTATGCAATTATTTTGACAAAAATATGTATCTAAATAA

CATAAGATAAGATTTATTGAGACGTAAAAAAAAAAAGATAATATTTATTGAAACATATAT

TAATACTCATAACAAAAAAAAAGAAACATATGTTGATAAATAAGATAAGATTTACTAAAA

TATATGTTAAAAAATATTTTTTACCCGGGATAAGATTTTAAAATATATTTCTGTTTGTAT

CTTAAGAAAATATTCATATACGCACTAAAAATATAAAAGCGTGTATTTTTCTACAGTGTT

TATATATATATATATATATATATATATATATATATATATATATATATATATATATATATA

TATATATATATATATATATATATATATATAAAAGATATGAGAACGACAAAGCGGTATTCA

TTATTGTCTATTTGATGTTAAAAAATGCATGTGAATCAATCTTCTATTATTATTTAAGGT

GAAGAAGTTTTGTACATAAATTTTTGTGCTTTGATATTATTATTAAAGCAATTTGTATAG

ATTATTGTTTAATTGATACTACTATTAAAAACGCATGTCAACAACGAATATATTATCTGT

AATCTATGTAGTAATCAAGCATTAATTAATTGCATCATTAATGTCATAATTAAGTTTTGT

TTAAAAACTTTATTAATGTCATAATTAAGAATTATTTTAAGTTAAACCATTTTATAATTG

TCCCAATAGTAAATACGCAAATGTATAAAGTTCATAACTTGATCATTCTTAATATATTTT

TAACACAAATTAGTAACCATTAATCACTTATATATTCAAATGTTTATTTAAAGTTATATT

ACACTTTTAATTCTTAATATTTTATGATTTGTATAAGAGTAAAAGAAATTTATACAAATA

TAATTGGGGAACAAAAAATGTACACATCCATAAACTTCAATTCTTAGACTTTGATTTGTA

GATCCTAGGTTACTAAATTTTCAGTCTTGCTGCAAAATTTTGACAGCAATGAAAATTCTT

TCGAGAATTACAAATATCATCAAACATCATAATTTAAAACAAAGAGCTTGACTTTTTTTA

TGTCTTACAGAGGAGAAGAAATTTTCAAGATGAACATTGTAGATTTGAATTTTGAGAAGA

GTGTTTAACCACGATATACTATAAAACATAATTCAACTTCTATGTATAAAATCTAAGAAT

AATCAAGAAAGTACATATTTTTCATACCTAACTACTATTATATGATCAATCCACCTATTT

TTTATTGGTCAAAATAATTATTGATAAATTGAAGTTTTAATTATCATTTCGATCTTCAAA

TTATTTTAAATGATTCGATTTGAAATCTTCAAAATTTTAAAAATATCAATTTGATTATCA

ATTTTTTAAAACGTTTAATTTGATTCGTAAGTTTTTAAAAATTAATCAAGTTAGTTTTGA

AATGACTTTAATAAATTGTTCACCTTGATTCTCAAGTTTTTAAAATTTTGAAAACTAAAT

TAATTCATTTTTTAAAATCTTGAAAACTAAATTAAACGTTTTAAAAATTTTAGAATCAAA

TTGATTTATTTTTTAAAATTATTTTTAATTTATCATTTTTATCTTTATTTCTAAATTGAA

TTTAAATGTTTTTAAAAAAAATTATCAATAAAATATCTTACAATATAAGATCGTGTTTGC

ATTGTCATTTGTACTGCAAAAACACGAATCTCAATACACCTCCACTATAAAACATAATGA

AGGGTTTGATTTTGCAAATGAAGGTTAGAATTGTCAAAATGACAATCCAAGCATGCACTA

AATTATATCATAACTATAAATTATAATTTACAATGTTTAAAAGATTTCTTCATTATAAAT

TTTAGTTATATAAAACAAATA

>Glyma05g03770 chromosome:V1.0:5:2916810:2918810:1

AAATCTTTCGAAGAAAACCTTATTAAATGCACAACATTGATTCATGGGATCAAAACAAAT

ATATCATCATTGACTACATAATTCTATTTCCTATTTTATGTTTTTTATGTCTACACATAT

ATATAGGTTATATTTGGCAAAATGAGCCAAAAATTGAAAAGTTAACTGCACTTAATAATT

AAAAAACTTATTAAATTATAAGTATCGGATAAAATTAAATATTTAACTAATTAAAAAATA

TAAAATAATAGAAAAATGAACAATATTAAAATAATGATTTATTTAAAAAAGATAATTAAA

AAATTCGATAAATATATTAAAGATAAAATGAAAGAAAATTTAAATAACTAAAAATTAGTA

TTTTAAAAGACGTACTTCAATTAATATTTTAAAAAATTATTAGAAACAACTAAAAAAATT

ATCTATTGAACAATAAAAAACTTTTTAACTAATATAAAAAATTAAAAGTTAATTTAAATG

TCTTGCAAATATAATCATATTTTATATAACATTTGTAACAAATGAGTGACTTATTGAATA

ATATTATTATTGTTTTCTTTCATTTTCAATATTAAAATTGAGGTTACATCATGTAGTTTT

TGAAACAGACCGCACGCAAGTGATGATGAATTGCATGGCATCAAGACGTTTTTCATTGAC

CATTTTGGTGCAATTCCAAAAAACATGTGATGATTTCCTTTAGAGTATGTTTGGATGGAG

AAATTTAATAGGGTAATTCAATTTTTAAAGAATTTTAATTACTTTTCAATTAAATAAGAT

GTTTGGATAAAAATTTGAAAAGAAATTGAAATTTAAGTATTTTGATGAGAAATTTTAATT

AACTTAAACATTAGCATTTCAAATTCTTTCATTTTAGGAAAGAATTTGAAATTCTTTTGT

GAAACAGCTTCTCTCACCATTTTGCTTTTGATCTTCTTCCTCTACTTCCGCAAGAAGCGG

AACCTGGCAGCGAAACATAGCTCAGGGAGCATCCCTCTGGTGTCCAAGGAGATCACCATG

GTCAAGGCCTCGGATCCGAAGAAGATGGAGGAGGCGGAGGTCAAGGTTGAAATTGGTGGG

GTCCAGCACCATCGAAACAGCGAACTAGTGTCGGTGGAGGATCTCGATATAGGGTGGGGC

CGCCTCTGGTACACCATTTGGGAAGTGGAACTCGCCACGCGCGGGTTTGCGGAAGGGAAT

GTTATTAGGGAAAGAGGCTACACCGTTGTGTACAGAGGAGTTCTGCACGATGCTTCCGTC

GTGGCTGTCAAGAATCTTCTCAACAACAAGTATGTCACTTTCTCTAATTCTCCAACATTT

TTTTTTCTTCAATTTTTTACTTTGCTAGGATTCCTCCAACAATTTTCGACCACCTGCAAA

TATATTCATTTAGCATAAACATCTAATTTCATCCCTCAAAAGAAAATAACCTTTTATTTT

TTATTTTCAATTATTAGAAATCAATAATCAATACACCATTTTATTTTAACTTCTAACTGT

TGTTACAGTTTTTTTTTTTAACACAAAAATGAAGAAATTCAATTTTTTTATCCAAACACA

AAATTTTAAAAATGAAGAAATTTAATTTTTTATCTAAACACAAAATTTTAAAAATGAAGA

AATTTAAATTAAAACATTTAAAATTTTCAGAATTTAAAATTCTTAAAATTTAAAATTTTT

TAAAATTTTAAATTTTTTTATCCAAACACAGATCTTTCATAATTGTTTCCGTTTGTACGT

TACAACCAAATCAGTTGTCCATTCTTTAGCAAGGATATCTAGATTTTAAGCTAGTGCTTA

TGATTTTGATTGCATTCCTAGTTGTATCCACTCCGTTATGATGGTTAAAACGAGATGAAC

ATATTTTTTGTTAAAAAAAATAAATAATTTTAAAATCAAATAAGAAAAATAGTGTAATTT

GCTGAGATATTATTCACTTTTTTTTTTAAAGAAAATCTATCCAGTTGAATCTTGCCAAAT

CCAATACAATCCTAGGATACG

>Glyma05g15130 chromosome:V1.0:5:16400621:16402621:1

AGATGACATAGTCCTCCTTGGAGAGTCGAGGGAGGAGTTGAATGAGAGGTTGGAAACTTG

GAGACGAGCTCTAGAAACACATGGCTTTCGCCTAAGCAGAAGCAAATCGGAGTATATGGA

ATGTAAGTTCAACAAAAGAAGGAGGGCTTCTAACTCAGAGGTGAAAATAGGAGACCATAT

TATCCCTCAAGTCACACGGTTTAAATATCTTGGGTTTGTAATACAGGATGATGGGGAAAT

TGAAGGGGATGTGAATCATCGCATTCAAGCAGGATGGATGAAATGGAGAAAAGCATCGGG

GGTGTTATGTGATGCAAAGGTACCGATCAAGCTAAAGGGAAAGTTTTATCGGACTGCGGT

AAGACCGGCGATTTTGTACGGAACAGAATGTTGGGCGGTCAAGAGCCAACATGAGAATAA

AGTAGGTGTAGCGGAGATGAGGATGTTGCGGTGGATGTGTGGTAAGACTCGACAGGATAA

AATTAGAAACGAAGCTATTAGAGAGAGGGTTGGAGTAGCGCCTATTGTAGAGAAGATGGT

GGAAAATAGACTTAGGTGGTTTGGGCATGTAGAGAGAAGACCGGTAGACTCTGTAGTGAG

GAGAGTAGACCAGATGGAGAGAAGACAAACAATTCGAGGCAGAGGAAGACCCAAAAAGAC

TATAAAAAAGGATCTCGAAATTAATGGTTTGGATAGAAGTATGGTACTTGATAGAACATT

ATGGCGGAAGTTGATCCATGTAGCCGACCCCACCTAGTGGGATAAGGCGTTGTTGTTGTT

GTTGTTGTATCATATTTTCTATTCATTTATTATTTTTTTAGTCTATATAAAATAACATAT

GATATGACTATAATTATAACCCAAATGGGACGAACAGAGTATTTTTTTTTTAATAGTATT

TCTTAAAATCTTGTTATAGTTAGACAATGAGCATCTAAATTCTCTGTTCGTCCCATACAC

TCTTTTTTCTCAGACTCATCGAAGGAGTGTGTTTCAAGTAGGACAAGGTGACCCCAAACG

GATCCACAGATTGTCAGTGTCGTGGATGCAGAAGAGATGTTACAGAACATAAATGGTGTT

GGTTACTCTACCAAAAAATGCATATATGAAGGCGCGTGAAGAATACGCCACGTGATCACG

CTAAGGAAATAATGAGGCTGCTATATAACAGTGTTACTGTTACCATTTATTTTTTTGTTC

CATGACAGGAGTATGCCTCCAATTAGACCAGTTTATGTAAGAACTAAGCCAAGAAAGATT

TAGGAAAGGGGAGATTACTGAAGTGCAAGGGTCACTGTACCAAATCAAACACACCATCTC

TCTTTAGAATAACACAAATTGTTGTGTTCCTTTTCTTCTATTTAGTTTCTCTCTTTCTTT

TTCCTTAATCCTCTGGTCCAGTTCTATCATTCTATTAATCTGATTTTCTGATAGTGTTCC

TTCTCTTTGCAAATGTTTTCTCGTCTCTTTCTTCTCTTTAAATATCTTAGATAGATTTTA

ACATATATTTTCAATTTTTTCAGACTCAATTCATGCTTGACTAGCTTGCTGATCCACATT

TTTTCTATAAAATTCATAGTTGCATTGACCATTTAAGCAAATGCTAATGGTAGCTGGTTT

TGAGAGATATTGTTGTATTAAGTGATAAGTGTGGATAGAATAGATGATTGTTTTAACTGT

TTGTTTTGTTTTGGTTGATCAAAGCAGCAGAGGTCTTGAAAGCTCAAAGGGACCTCCATA

TTTCTCGTTGGTAAGTATACCAACTAGCTTATAAATTAAAGGACTCGGATGTTAAGGTTC

AGTTATTTTTTATATCATTCTTTCCTCTTGAGGAAATCTTTCCAAGAGTGTCAGTGCAAT

TACGCACATTGCAATTGTACACACTTTCTTGATTTTCTTGTTTTTTTCTTCTTCCATAAC

TAGCTGTATTTGTACATTACAGCATGTCAATATTAGTTTCCTCTTATTTCATACATAAAA

AGAATTATGGTAAAATAGTAT

>Glyma05g36600 chromosome:V1.0:5:40431696:40433696:1

TACAAAGATGTTGAATAATGTGAAAGTCGTCGAGAAGTTGACAAATTAATGAAAGACATC

GTAAGGGTGATGGACGATCCAGTAACCTTTAATTATTCTAGATATACCATTAACTGTAAT

TATGAAATTATGAGTCTTTAATTACTCTAAGTACATTAACTGAAGTTATAAAGACAAGAC

TTTAATTTCACTAGTGCACACATAAAGACACTAAGGCTATCAACTACTAAAACCAATAAA

AGAAAGACAAAAAAAATATGACTTCAATTAATTCTCTGAAAAAAAGAAGAGGAATGATTA

GTTTGAAAAGATAAAGGACCATAGTGAAAAAAAAAGACTAAATAGGTCACTTGACCATTA

AAAAAATAAAAAATGGAAAGAAATAGGTGATTAAGAAAATTAAATATTTAAATATTTCTA

CCTTCGTTCACTTCTTTACCTTATGCTATCTTTTTTTTTTGAAAAAAAATTAAGTTAACA

AACTCTGACCGAATATCAAAGATTATATATATATATATATATACATTCAAAATTAGAACA

TGCTCCTAACATACATTTCAGATGTGATCTTGTGTTTTTAACGCCAACTGATGGATTTAA

CTTAATTTATTGTCTCTAATTAATAATAACAATAAATCACAATATAAATTATTTATGATA

AATCTAAAATAGTAGTAATGTTCAAAATATTTATTTATTACAAGTTTTAATCATAATATA

ATTTATATATTTAGAAGCATTTATTTATTACCAATTATAAATTAGATATTGATTGTGTGC

AGTGCACAAGTTAAAATACATAGAATGAAAAAGTCTTTACGTGCTCTCATTGTTCCACTC

TGCCTCTCACATCCACTTTCATTTGAATAATACTCTTCTCATGAAGTGGTGACTTCACTC

TTGTAACATGCATATCACATATATTCTTATACTTTTAATAATAACGGTTTAACTGTAATT

AAATACTGAAAACCAATTTCAAGTATATATATTTTGTCTTTAAAATTCAAATATGCATTT

AATGTAATGCAGTAAAAAAATATTAATATGATATTTAATATTTTTCTTAGAATTAGCATT

TAATGTTATGCAGTAAAAAAACATATTAATATAGCATTTAATGTTTTTCTTAGAATAAGC

ATTTAATGTTATTAATTAATGTAATATTTGAACAAATGTAAATAAATATACTTTTTTTTT

CCTTAAATAGCGTGACAATATTAAAGGAGAATTTGATTCTATTTGAACTCTCTAGAAAGT

AATTCCACTTAAGTTGTTCTGTCTTTCTCCAAGCCATCCCGAATTCCTTGGAGGCTGAAA

AATCCTTGGCTCAATTGTATTGATCTTGTTTCTAAAATGCATTTTCATGTTTCCCACATG

TAGAGAAGAAAATTCTTGGACGAACAAGTTAAACTAACCATTTTTAAGGGTGTATTAGGT

TTAGAAAATGGAAATATATATAAGAATTTTTTTTGAATTAAACTAGGACGTAAAAAGTGT

GGGTCTAAAAAAAATATAAAATTTATCTCTAATATTATTTTTCTCCTCTCTACCAAACAC

ACCATAAATATTAATACTCTTACATGGTGAAATTTTGTTCCTAATTTTTATATAATTGAT

TATATGCACAATAGGTCAGGTCAACCTAACTACAGGTATTGTTGATTCTTCCTTCCATGG

ATTTTGGTATAGTCCCCCATGCTTTTTTGTTATCTTTTTTCTTATGTATAATACGTTGGC

TGGTTGAGGGTGTTAGGGATAGGTGTCAACATAGCCCAGATTTACCTCATTTTTTTCACG

ATAGCTTCTAGCCTTCTCTAATCTTTAAAATATTTTAATAAATATAAAAAGCTATTAATA

TTAACTTACTTATTTTAATTATTGTTCTTTTCATATTATTTTATTATTCTTTTTAATATT

ATATTTTAAGATATTCTAACCATTACGGTAACTATTTGCATCTTTCGGTTTTCATTAAAT

CGAGCACAATGGTTAACAACT

>Glyma05g36620 chromosome:V1.0:5:40448142:40450142:1

AAGCAATATTTACATAACTAAAAATACTAAAGCAACTAATAAAACACAAAGGAAATGAAA

AGGCTTACTTTCTCTACGTTGACTGTGTCTGAGGAGCAACCTTAGAAGCCAATTAAGTGG

AAAGAATAACATGCCTTACTCTTCTTCATCAGTGTTTTTTTTTTTTTTTTGGAGACAAGG

TCTTTGATAATAAGCAACAATTACGAGATAGAGAAAATTTATATCTTTTGCTTAAAATTT

ATCTTTAGATGAGAATCTTACTTCTTTTTGTCTTAATCCTTCCATTTGCCTTAAAGATAC

TGCCACCCTCTCTTAAGAGTTATTTGGTTGAAAATAAAAAAAAAAATGAAAGAAAATAAA

TATTTGAATTAAAGAAAAAAATAAAGGTGTAGGATTCACATCAAAGTTAATTTTTTTTTC

TGTTCTTTCTATTTAGGGTGAGTTTATTTAAACTTATTTATTGAAATAAATATTTATTTT

AATCAAATAAGTAACTTTTTATATTTTTAGTATGTTTATCTAAACTATTTTCACTTAAAA

AAATATAATTTTTTTTACTTATTTTAAGAAGTAAATTCTTTCTATTTCTTAAAAAAATAC

TTATTTAAAAGAAATTAAAAATTTAAACAACTCTTAATATCATTGCATAAAATATAATAT

AAACTCATAGATCCTTTGAAGAATTTGAGATATACTTGAGAACCAAACAATTTAGTCTTG

TGATTCTATGAATTACTCAGTTGTGTTTGTTATATTTATTTAGATGCATTGAAGTATTGT

CGTTATTTAAATATTTTGTATTCAAAATTTTCATGAATTGTGTTTGTTTAATATGATTTT

TATTGATGTGTAAATATATACATATGTAATCTTATTTTAAATTCATTTTAGATACCATGG

TTTTTCATATTAGCACAATGGTTGAGAACAACATTGTTTGCGAACTAACTTTTGTTAGTT

GTTTTATTCACTAAAATTATCATCTCAGTGTAAGTGTTGTTTTCAAAGCATAAATTTGTC

GAGTATGAACATACTTAAAGATATAATTTACTTAGTTACATTTATTTTAGTTTGGTTACT

ACTTCAGAATTAAACATAAAATGCATCACTTTCGTTATATTGTTAATATTCATTTAATTT

AGATGTTCATATTTTGAGGATAATATTTTTACTTTTATAAAAAATTATTTATTTGACTTT

ATTTATTAAGTAATTTAAGACTATTATAATTTTGAGATTTTATATTTTTTTAGTACGAAT

TGTTATTTTAGTGAAAGGCATTACAAGAAATATGTATACCAAACCATGTTTAAGGTAAGT

TAGTTTAAACTAAAAAAATATATTTTTTATATAGTTTTTTAATAAATAAATAGAATTTGC

TTATTAAAATAAGTGAAAAAATTGTTCTTTCAAGCAAAATAATTTAGACAAACACACTAA

AAAAATATAGAACTGTTCATTCTAACAAATAAATTTAAACAAACTAATGTTTAATGATGA

CAATATAAGAGGGTGTGTAATTGAGTTTTAATCTTGTTGGTCTTTTATTTTTAATATTGA

TCAAAGAGTATTAGTATAAGAGTGAATACGATGACCATCGTTAAAAAAAATATAGGATTG

TTTAAAATTTAATTTCAAGCTAGAATAATTAAATGCAATTTTATAACTTTTTTTAGAAAT

TACTTAAAAATATTTCTGTAAAATAATCAAAATACTCTCGGTTGACGGCATAAATCTATC

CAGGACTTTTTTTTTCCCAAAAAAAAAATATTTTTAATATGTCTATAATTTGAAAATTTT

CATTTAAAGGGAAAAAAAATCAAGTATTTTTTAAATTGGAAAAAGTTTGGAACAAAACTG

ATTTATTGGGTAATTATTTATCATTTTTACATGAAAATAGTTATATAAAATTATTTAATA

AATGAGAAATTTCAAGGAGAACAAATTTATTTTGGGCTTGATTTAGGCTAAAGATAAGTA

GAGTGGTGTATCAACAAGGAG

>Glyma06g00310 chromosome:V1.0:6:111629:113629:1

CCTGATCAGAAGTAGGAAGAGAGAAAGAGCAATGAATATGATGAAGGTGTGGGATGCTGC

TCAAGGTACCCACAACCACAACGACATCTGGCACTGCCCAACTGCAATGGCGGTGCAGTG

TAAGTGTAGCAACCACCACACTGGCGTTTCCGGCGGAAAGAGGAGAATGGAATTGGAGGG

TGAAATGCAGCAGGGGCGGCGAGTTTTGGGCCTGGGAAGGCAGATTGTGCGACGGGCACT

TGACGAAGGCTACGACGTTAGGTGTCTGGTCAGGCCCAGGCCCGCTCCTGCCGACTTCCT

TCGCGATTGGGGAGCAACAGTTGTGAATTTTCTATTCCACTCATAGTCATAAGGTTATTG

TTTTCTCTTTCTCTTTCACAGTAAACCAGAAACCATTCCTGCTACCTTGGTCGGCATACA

CACTGTCATTGACTGCGCCACTGGCCGTCCCGAGGAGCCCATCAAAACCGTAACTAACAA

TCACACGCACTTGATTACTTGTTTCTTTTCTACATTATCATTATCACTTATCACATATCA

CATACATTATAGGTTGACTAGGAAGGTAAAGTGGCTCTTATACAATGTGCCAAGGCAATG

GGAATTCAGAAATATGTCTTCTACTCCATCCATAACTGCGACAAACATCCCGAGGTTCCC

CTCATGGAGATCAAGTTTTGCATTGAGAAGTTCCTCCGAGACTCTGGCCTCAATCACGTC

ATTATCCGCTTATGCGGTTTCATGCAGGGCCTTATCGGTCAGTATGCAGTTCCTATCCTA

GAAGAAAAATCTGTTTGGGGTACTGATGCCCCCACCAGAATTGCTTACATGGATACTCAG

GTAATACTATTACTCTTCTGTCATCTCCCTTTCTTCATTTTTCTTTCTAATCAGTCTCTC

TTTCTCCACCTTTAGGATATAGCTCGCTTGACATTTATAGCCATACGAAATGATAAATTA

AATGGTAAACTTCTTACATTTGCTGGTCCTCGTGCCTGGACAACCCAAGAGGTAACTTTA

CTGCTTCTTCTCTGCTTGGTCTTTCTCTTGCTTAGATCCTCAATTGTCACTTTTTTTTCT

TCTTCTTTTAGGTGATAACCTTGTGCGAGAGGCTAGCAGGCCAAGATGCTAATGTCACTA

CTGTTCCTGTCTCCATTTTAAGACTCACTTTTTTGAGTGGACAAATGATGTTGCTGACAG

ACTCGCATTTTCAGAGGTACTATCCATACCTTCCTTCATCTTTGCTAAACTGACTCACTA

TTAGTTGCAGGGCATTGCTCCACTACCCATCTTTTCCTTTCTAAATACTCATTATGAAAT

CAACTTTTAATACCCTTTAGTTTCTAGTTGCCTTATTACTGCCATCTCATAACTAGAATC

TTTCATAAATTTTAATATATAAACACTGTCTGCCTCTATGATGATTCACCGAAACAAATA

AACGTGGGGTCGCAGGTTCGGTGCACTCACCAACTTAATTTAGATCTGTTTGGATAAACT

TCTTCATAAACACTTTAATTTTCTCCCTTAAGTTTAAATCAACTTGTGTACTTAACTTTT

ATAGAAGCTCTCATTCTTAATTTTTCCAAAAGCTGAAATATCTGATTTGATTCGTGTACT

TGGATTAATTCGGGTGGAAACTAAATCCAATCCGACCAATCTTGGTTTGTGCTTCCAGTC

AAATTAAAATGAGGGCTGTATTTTAGAAATAAAATATGAAATTATAACGATTGTTTCAAG

AAATAATGGTGTACATTCTTCCGGTTTTATAGTTTGCTAAGTTACAATTTAAGTTGTTGA

TTTTTTAATCTTCTGTTATAACTTTATTCCAGTGGGATAATGCTGTGATGCTGTTGTTGT

TGACTTATATAGATAAAACATCATTTATTTAATTTTACTGGACAATCACGATTCCACCAG

CTAACTAACTATGAACTAAACGTTCACTAACTCAACCGTCAAAGAAAAAGAAGGTTATGG

AGGATTCTTCCTCATTTGAGT

>Glyma07g00820 chromosome:V1.0:7:443423:445423:1

ATGTTAGCGGACATCGACCGTTTCCTCTTTGAAAATTTCAAGTCCCTATTCCTCGATGAC

CGTGAAGAACCCGACAACAACATTAACCGCAGAGCAGAAATGTCTCCCAAACTGGACCCC

ATCCGCTTTGACTCTTCTAGAAGGTTCTTCACGGACGCAACCACCGAAGAGGGGTCCAGC

TCCGCCATGTCGGAAAGCGAGACCGCCGAGGAGTCGACGGTTGTTCCGGGAAATTGTGTG

GTGGTGCTGGCCAACTCAGGGAACCCCAGCGAGGATTTCCAGCGATCCATGGAGGGCGTG

GTGGAAGCGAGGTACGCAAAAAATTAATTAATATTTTTAGTGAGGCACATAGTGACGGAC

TTTTAGTGACCAACTGTAGCGACGAAAAATTGTTTTTCCTTGTTAGTTCTGATATCGCAA

AATTTTGGTGATGAATTTTTTCAACATGCAATTTATTTTGTGACTAATTTTAATTTTTTC

TGGTAGATTAAGGAATTGTGAAAAGGTGGATTGGGACTTCATGCAAGAGCTTTTGTTTTG

TCACATGAACCTCAACCAGAAGAAATCGCACAAGTTCATTCTTAGCGCCTTTGTTAATGT

CGTCACTGCCATGCGCAGCCCGCCGGAGATTGCTCCGCCGAAGCCTACGCCGCCGCGAAG

CGTTCGGACAGTTAGGATCGGAAGGGAGGTGCGGAAGAAGACGAAGGAAGCAGTTACCTT

GGAATTTAGATCGCAGTAAATTACTTGTGGATGGAGGAGGTTTAATTAGTCGTAATAAAA

GCATGGAAACAACTTGTATGAAACTTGGATTTAATTGAAATTTATTTTGTGGAGATAGGA

TAAATTTCTATTAAATATGTGTTTAACAAAGTTTTATACCGAAACTAAAAAAGAACATAT

GTTTTTCTTTTTCTTTTGTTTTTGTTTGGTTAAGAACTGCAAGCAAATAAGGTGAATGAA

AATTTGTACTTCTGTCGCATTTTCTCGTTTCTTGGCACTCATCCTTTTTACGGTTAAAGA

AGAGGAAAATATCTCTAATTTTTACTTATAACTTAAATTAAATATAATACAAATTCGGTC

GACCAGTTTATTTAACTATCAAAATATTTTAATATTTATTTATTAATTTATAATTTATCT

ATTAATAATTCATTGTATGTAACATAATTAGGATAATGTTTATTTTTTAGATTAAGTAAT

AATATATATTTTTTGGTAACCATCATAAGTTTGAGACCTCGAGATATTCCCCCACAACAA

TTAATTATGAATCTTAATAATATTTTTATTTATAGAAGGGCAAATATTACCTTTTTTCTC

TCATATTTTTTATCTTGAATGTGTTGTTCAAATTTAAATATACAATTTAAGTAAATCATT

GAAATATTTGGTATTTTTGTCTACCTCTAAATGTTTGTTATCTATATTTTTCTATCATTA

TTTTGTTAAAATTGTTAGACTGATTTGCATGCCAAATAATTTATTAAACTGGGAAAAGGT

TTTCATTTAAAAAAACTAATACACATATTTATTTAAGCACGTATATTTTTTAAAAAAATA

CTTAGTAATATTTGGAATCAGTTTCTTCATGTCTTCATATATAAAAAGTAGTATTTTAAT

TAGGTTTAAATATAATTTTAATTCTTTTATTTTGTTTAATCTGCAATCTTGGTTCTTACA

TTTTGTTTTAAAAAAAATCATAATTTTAATTCCTATATTTCAAAATAAAAATATTTAATT

ATTTTATTTTAAAATTTATAATTTTGATTCTATTATTTTAAAAAAATATGTAATTTTATT

TCAATTTTCAATTTTATCTATTTTTTATTTCTTATATTATAATTAATTAAATAATTTTTA

ATGATATCTTAAAAAATATATAAGATTTATGATTTAATTAGATAAAAATAAAAGAAATAA

AATATAGGTAAAATTAAAAATTGAATTAAAATTTTAAATTTTATAAAATAAAATAATTAA

ATATGTATATTTTAAAATTTA

>Glyma07g02450 chromosome:V1.0:7:1668640:1670640:1

GTGGTGCCGAGGTCAATGCCAATGCCCTTACCTTCTGTCTTGGAAGCCATTGATATTTAG

CCACGTTTTAGCATTGCCAAATCATGCATATACATGGTTATGCCGATTTGCACTGTTACA

GGGATTGTTTTTTACTTAATGACATGTTATTTATTATACTTGTTCCGATCGAGTTGTCTG

GTGGAATCTCTGATTTCCGATATCGAAGTAAGGAGAAATACCTGCAAAAAGAACTCCGAC

GAGTAAGTCAATGGTGTTCCGAGGTGTTTATGTGTGGGATGATGTTCGGAGATAGTTTCA

TGATATTCCGAGCCGCATCAGTGAAATGCTCGTCTCGTGGCCGAGTTCACGGATGACAGT

TGAAACGGTCATGTACTGTGCACGTGGGGACTCCCCTGCGGAGTTTTTGGGACTGTAGAG

TACACTTGTTCTCTTTTCAAATAGTTGTTTCGACTTTATTGTTAAGCATTTTTCCTAGAA

CAAGAACTACCCTTCACGAGACAGAATTACGGCTTTAACATCACCCTTAAAATAAAAGGA

ACATACTCGTACCAAAGGTATGCTAAATGTGTGTAAGGGCCTGCTCCATATTACACACTT

TACCCATGCTTGAGCTTGAGACCTTTTCACTGTCTCTGAAGCAGCATGGACAGAACAGGT

CACTTATTCTTATTCTGTCTCCAGAAAATAGTTATCCCCATGCTTCTCAGTTCTCAATTC

CATTTACAAGTATTTTCTTTCTCAAGAATCTTATTCGAAACATGATTTGACACTAAATAT

GAAGACTTCTCTTAACTGATATTTAAACCTTGAGTCTATCTTGTCTCAGTTCTCAATTCT

TACCTTCGCTTAATGAAATAAATTAATAAAATCCATAAATACCTTAACCTTACCCTAGAC

CAGATAACTCAAAACCTGATACCCCTACTTGTGCTAGGCTTTGGACATTGTTCAACGAAA

ATCCAATATACCCAATAATTTACAAAGAATCAGCCCTTGCTTTCCCTATTCCCAACCCCT

AAGATGAAGGTAAGATTCCTTGCTTTGTTCTTACTCATTTCAGGTATTCAAGCCATATCA

ACCTCAGAGTTCAATATGAATTTCACTTCAAAAGGTAAAGGCCTAATCCAGAGCAATGGA

CCAGTACAAGAACTTCTTGGTACTCGACTTCTTCAGCCAAATATTTCCCATATTTGAAGG

CACCCTGGTTTTTTTCAGTGTCACTATTATATTAATGTTTTTCAGTGATGTATAGGGAAA

TTATATGGAAGCATAGAAGACTCAATAAGGTTGAACGACAAAGTTGAGGAAACTAAAGAC

GGGAACAAAGAAGAACGACTTAATACAACAAGAAAAGCACAAGGTGGAGGAAGTTCAAGA

GGCACAGGAAGTAGAGGAAAGACAGGAAGCGGTGGCACTGCTGATGTTAATCGCCGGCCA

CGTCAAAGTTCTGCACCATCAGAGCCTCTTTTTTGGGTCTCAATATTTAATCCATGCGTA

AGCTTGGCTTTAGTCATATTGTTTTCCCTTCCACTTGGTTTGATGTAATGCATGCAGAAA

AAAGTGGTTACATATGATATATGTTGTTCTTGTTTAATGCCCCTAAATAGTGCCGTCTTC

TTTTTTCAATATATATTTTTAGTGCCGCCGTTACTAAGAGCGTCTTTGCTTGTACATCAG

TTTGTGTCCTCCTTCTCAATGCCAACATGTTGTCATCCATCATATCAATGAATGGAAGTT

TGAATCTTTGCCTGCTCAAGGGTAAGTTTTGATGCAAATATGAAAAGGTTGAACTTGTGG

TGTTGAAATTCTTGTGATATCCAAGAGAAGCAGAAATGGAACTAATTTTACTTTTTTGTG

CAATTACAAATTATTTACTCAATTCATCAATGTTAATTGTTAAATCATTCTCTTATGAAC

TCCAGAAGCTTATATGCCAAAAATAAAAACTGCTCTGATTATCATATTTTTTTAATTAAA

TTGAAAATGGCGGCCAGATGA

>Glyma07g26550 chromosome:V1.0:7:29386810:29388810:1

TAACACGCAGCACCCAATGACAGTTAGTCTATTACGGTGGTGCTTGGGAGTAATCTTTCA

TAGGCATCATTCTTCTACCCTCTGTTTTTTCCCTTGTGTTTTTGTCCAATTGATTGTAAG

GTTATGTAACTGGCATGCCGAGGACCAGTGTTACTACTGATATTAGCCATGGATTGGGAT

AAGAAAAACAATTGCAGGATTCGGTATGCATATCTTCTGACCTAGTGTTATGTTCTCTGG

TTAGAGAGAGAGAGAGAGAGAGACCGGCTAAGAAGGCAGGCAATGGAAGACAATTAACTT

GGACTAACCTTTCACTCTTTCGGCATTTCTGAGTGGTGTGGTTGTATAGGTAGCTTTATT

CCCCCCTTTACTCCTTTTTTTTGCAGTTTATATTTGTAATATGCAGATAGTGTGATTGTG

ATGGTTCAGGGTTTGTCAATATAATACTAGCGGTTGGTCCGTGTCATGCACGGAGTATTT

GCTTGGTTGTGAAATGATATCGACTCACAATTATTCCTAGTTTTGTTAGAAAACACCTTG

AAAATAATTCTTATAATTAGATATTGATTATAATTATAATTTGTAGGTTGATCTGTCAAA

ACACAATATTTTTATAGAAAATAAATGCAATTTTTTTGACATATTATTGCTAATAAAATT

GAGTGCGATAATGATTAATAAAAGATTGTCCAGATTTAATGAGTAGTGAATAGGAAAATA

ACTAATTGAGTGCGATAATGATTAATAAATTGAGTGCAAGTTTACTATATCAAGATATTA

ACGAGGCTGGTATAGCGAATAGGAAAATAACTAATTAGTAAAAAGCCACTAATTATTTTT

AATGAATTACCTCTAAGTCTTTGCAAGTTAGTCGCACACTATCGCTAGCTCTGGATAAGC

AATATAAATGCACTTTTTTATTATTATTTATTCATGAGAATCAAAGACACGTTTATAATA

ACTGATGCATCCTGGTCATTGAGTCAATAAATTGAGTTAGAATGCCTTGACTAATATAAA

TGCATATTAAACAATAAAATTATAAAATGACATGAACAAAACTACCTAAAAAGGCATACA

CTATAAGCTTAATCATAAGAGAAGCTAATGTAGTGCATAACCTTTCAAGATAAAATCTAA

GGAGAGTATTGGATTAAGATTAGGCATCATTCTGATGACCCCTCCACTCTCGTCCCCTTC

CACAGTTCAAGCCTTACTCCAAGCTGTAATTCCTTTCTTTGAGCCTCCCCTCTCCCTAAA

GGTCACATCTTCACCTTCAGCGCCTTCAACTAAGATGTTGAGCTCATTAACAATTATTTG

GTCCTTAGAATTTGCAGCAACTCCAATTGAGCCATGGTCTTGGTGCCGCCCACCGCGACC

TTGATTGCCCAACTACAGTGCTTGGTGATGACAACGATCGAAGGGTGTTGTCTTTGGTCT

TGAATGTCCTCTCTTTCTCGATTTATGATCCTTTATGTTCTCTTGTTTTTTTTTTTTTTC

TATTTTCGTTCTTATGTTTTAAAAATTCTATTTTAGTCCCGTATGTTTTATGAAAATTTC

ATTTTCATCTTTTTGTCAAATTTTTGCTAACAGCGTCAATGTTTTAAATTTTAAATTAAT

TTCACTGTATGTAAATTCCAAATGCATAAGACCTGATAATCTTTTGGTTTATTGTTCTCA

TGCAAGAACACACTGAAGAATGCATAAACACGTTGAGAAGTTGAAGTCACAGAAGTATAC

TTGTTTGTCATCATTAGAAATTTACACATTGGCAATTTTTAGTGATTATTATATTAAATT

GATTATTTTAAAATTTAAATTATTAATATTGTTAAAGAAAAATTCATAAAATGATCAAAA

TTAAACCTTTTGAAAATACAAAATCACTAGAGTAAGAAATTTGGATTATATAGGACCGAA

ATAGTAAAATAAAAAAACAAAAATAATAATTTAGCAAAAAAAAAGTACAAAGTTAATATA

TAATTTTATTAAAATAGTCAA

>Glyma07g30290 chromosome:V1.0:7:35321611:35323611:1

CTGAATCACCTCATTTTAGAGTATTAATCTGGCAGCACATCAACCAATTTATGTGTCATG

TTTTTCAACTATTGTAATAATATTATTACCCAGTAAAAATTCTTTTTGCCTACTTCATTA

AGCAAAAAAATCCTTTTAAATATAGCTAGAGTCCGTTTGATTAGCTAAAACATATGGTAC

TAAATATTTTTTTTCGATAACTATACTGAACATTCTTGTTTCAGGCTTGATTTAAAAAAA

ATGTGACAACACAAAAATTCAGCTTTTTGTTACTCATAAAATTTTGGAGACAAAATTATA

TTTCAAGTATATTCAAAAAATAATACATCTCCCTTTTCTCTTATTTCTTACCTATGTTAT

GTCTGTAGACACAACTTGAATAATTGCATTTGAATTTTACTATTAACTCGTTACAACAAT

CTTATCTTGCAAAGTCGTGTACATAATTATTCAAGTTAGACAATTATTTTAAAAGTTCAC

AACCTCTATAGAAAAAAAAAAAAAAAAAAACAAAAGTTGTAGTAGGATTTATGACTTTTG

CATACAAAGTCATAGTTTCATTTATGACTTACCCCTAAATGGTTACTTTTTCAAAATCTA

CCCCTAATTGTTAATTTTGAAAAAAAAAATTATCCTCTTTTAATATTAAAAAAAAACATT

TTGTGTTGATCACCTAGCCAACGTAGGCCATACACTGGTCCCTTCCAATGTACCATGCTT

GTCTCTCCTGCAACTATTTTTTTATATGTTTTATAATGCTTTGGATAGTAGTTTACCATA

TCTAATTAATTAGTTAAATTTTTTTGTTTTTTTCCCCATATATATCCCTAGAAAAGAACA

ATTGTCATCAAGTTTTCTAAAAGTATCTTTTTTTCCCCTTAACTCTAATTATTACACCAC

TCAATAATTTTAAATTAAATTTTTTTATCTCAAAATCTATTGTTGAATTGAAAGTTTTTA

TATACATTTTATATTTTTAAAATTATTTCATACTTTATTCATATACATCAAAATAGATTT

AATACATATTTTTTAAAATATATTAAAATGTAAATCATTTGATTAATTATCACATTATTA

TTCATCTTTTTACACAAACAATCAAATTAATACATATTTATTGAAGATGTGTTAGTGAGA

CCTTTTAAACATTATTTGATCTTGTGTTGAGATATGAAAGTTTAAAATGAAGACAAAGTC

ATAGATATAAAAAAAATTGATGAAGACAAAGTTAGATACTTATAAATTTATGCAAGACTT

ATCATCCTAACCAATTTTGGCTCAAGAGATTCACTCTACGAGTTTTTCTTTTTTTTAAAA

AAAAAATCATTTTGGGAGTTTGAAATAAAAAATAATATTTTCCGAATCATGAAACTAAAC

AATTTTTTTTTTTTGCAGAAAAAAACGAAAGTATTTTGGGATAATTTGCAAATTAAGAAG

TAAAATATTTTACAAATACTTCAATAAAGTCTAAAATTGAATCTAAAGACATCTTGACAA

AGGTGCAATGTTAAACAGGCATTACACCTATTGATGATTAGCTGGAGAGGGTACGTTGAA

TATTTTTTTTATAGTTAGATGACTTATTTTAAAAATAGAGAATTTAAATTGAATAATTTA

CTTTGAATATTTTAAAAAGATACTTTTCAAAGTGTATTATAGAGTACGAGTGGAAGAAAA

GTTGATAGCGGGAGAGAATCCAAATTCTCATTTTTAGAGTGTAGAGTGAAAGGAAAGAAG

ATATATAAAAAAAAAATACAGGAAGAGAAAAGAAAAATCATAAATATGCAAATAATACGG

TGGACAATGAAAAAAGAAGTAGAAAAAATAAAAGAAAAATAAGACAAAAATGTAAGATAA

TAATATAATAATTTATTTTTATTCACAATAATAATAATAATAATTCATTTATATATCAGG

CGTCCATTTTGTTGGGTTGTTGGAAGAAAAAAAAAAATTGTATGTCTTGAAGAACACGAT

ACATTCTTAAAAAAAAAAACA

>Glyma07g32921 chromosome:V1.0:7:37778575:37780575:1

AGTTTAATATGAAGTTTTCTTGGGGTTGATATCAAGTTTCTGGAAATATCGGAGTTAAAT

AATAATAGAAAACCATTGTCACGTAAGTCATTAAATGTGATTCTTTTTCGGTTTGTTCCC

TACATTTGTCAGTGCTATAATTTAGCTGACATTATTATAACAGCAGCAAATAGTGTGAAT

CAAATTATATACAAAGAAAAGAAAAAAGGACAACTTGGTGCCATAAAGCTCCCCCATGAG

AGTCTAGGAAAGGATAGATTATTACATGGTCTTAATAATGTCATATTCCTTACTAATTTT

AATATGATATATAACTAAAATTTTCAGTTTACGAAAAAATTAATAAAATTTGATTATGTG

TCATAAAAATTAATCAATTTTCATATGACGACTGAATTTTTCAGTTTACAAGAAATAATC

AATAAAACTTGGTGATGAGTTACGTAAAACATGGTGGTATAAGATTATATAATAATTTCT

AAAAAAAAAAAAGGGTAAATCTCGCTCATATTATGAGTTTATTGCAGGTAGCCTTAAACC

TTGCATTTGTAAGATGCTGATTCCACAACTAAAATGTGACCTAATTTTTCAATTTGCACT

AAAGAATTAAAAAAGAATCAACTTGTGTTACATAAATACTGACCAAGATCATGGTGGTAC

GAAACCATGTAATAATTTCTTAAAAGAAAAAGGCCAGTCTCACTCATTATGAGTTTATTG

CAGGTAGCCTTACACCTTGCATTTGCAGGAAGCTGATTCCACCACAACTCAAATGTGACC

TAGTTACACAACAACTCTGATCGTTACACCAAGGCTACCCTTCAATCAAATAAAAAACAA

ATGTACATGTAAAAATTGAATAGAATTTTCTTTTCATATACTGATCAAATTGGAAAAGAG

TGTTCTCACATCCAAACCCAAATTGAAAATGGGTGTGAAGTATAAAATCTGAAAATCAGA

ATGAAACTAGTTCCTCATCAGAAACGATTCTATAATAGTTATTCACTATAACCTTTAAAG

TATAACAACGTCTGTCACAAGTTATGAGGGAGAAAAAACAAGCTATAAATTATGTAGTCA

CTTCTGTTGAGAAGTCTAACTCACTGGTTCTCAGATTCCCCCGAGCATTTCTTGTTCCAG

CTGCCTGAACACGCTTTGCGCTCCCCGCGTTTTCTTTTGTTCTTGAGAATGTTCCCATCT

CTATACATACAGGACTTCAGAGTTGATTGCTCTTGAGACACTGCAGGGTTTGTTGTTGCC

ATATGAATATCTGACATGGGGATAGGAACAGGATTCAGACTTTCTTCTTCCCCTTCACCT

CTCTCCACATCCTGAAGCCTCTCCACAGGATACTGACTTGCACTTTTAAAGGTGTGCTCA

TCTGTTTCAGCTACCTCATGTATCAAGACATTGTCCACCATCTGCAACCATAAATGTCAA

ATGTGAATATTTTCTGATATGTACAAACCACCATCATAGATAACAAACTTTGATAGCAAT

AGCAGACCCAAGAGGTGCTTCAAGAGACCAGCCCACCTCATTCAGTCACGTGGGTAAAAC

TAGCAGCAGCATATCCCTATTGCCTAATCCACTATCACTACCCATAACATAATCTGTTAT

AACAGAATTCTGTCCCACTAATTTTGTTATTCTGTTTCGTTATCGTTTAGCAGCACTAGG

TGCTTAGACTCCATTATAAATAATGTAAAGCAATAGGAATGAGGGATATCAGAAAAGCTT

CTTACTATTTCCCTTTTGTTCTTTACTATTCCTGTTATTTTTCTAAGTTTCTGCAGCTCC

ATTTCTGTGTCCTATCAATTGGTCCAACCTGCCGGTAACCTGCTGGAAGCATGACTGATC

CCCACCTCTGTCGGTGGCAGCCTATCCGCCCAGCCATCCACCACCACCCACATCTGCACT

TATTCTACCCCCCACAGGTCCCATCCCTTCCATTTCACCACCCTTCGCCGTTTTCCCTGT

TGGAACCCCTGCCTTCGACCA

>Glyma08g02940 chromosome:V1.0:8:2027878:2029878:1

GAAGTTAAAGCTTAATTACGTTTTTAATTCTTTAAATTTAGTTAATGATTTTTTTGTCCC

TAAAAAAATTATGTTTTTATTAGTCTCTCAAATTTTTGAAAAATTAATTTTAATATTTCT

TATTTTTTGTTAATAATGTCACAATTTAAGGTAATTGTACATTACAAATTTTTTTGATTC

AATTTTTTTTAAAATAATTTATGAAAGTTTAAAATCACAAAAATATGTAAAAAAAAAAAT

CACAAAATTTTCACATTTTTATTTTTCTTTGAAATCGTTGACTGTTAGAATATGTAAAAG

ACGATCGTCAAACCATACATGAAAAATATGTTTAAGGTTTAAAATTATCATAAATTATTT

TTAAATAATTTTTTTAAAAAATGGTGATTGAAAACCACCAGTAAGTGTAAAACTATTAAT

AACAACGAACTAAATTAAAAGCAGTTTTTTAAAATTCGAGAGATTAATAAAAATATATTT

TAATATTAATATATGATATAATATAAATATGGATAGACACTGGTAAAGATATTAATATGA

AATTTAAATATACAATACAATAATTTTCTATTATTGATATTTAATAAATTATTTATTACT

TTTTGAATCATTTAGTACTTAAGATAATATAAATTAAAAACATTTTTTTATTTTTTATCA

TAATTTTGAATTAAAAAAAACGCGGACATCCTTTTACTAGATTGCAATCCTACTTTCAAA

TTGATAAATTATAGAGAATAGAGAAATAGTAAAAAAAATGATTTCTTTTTAAAAAAAAAA

ATCAAAATCATGATAAAGGAACAACCAAATTATTTATGTAATTCAATTACATTTGAACAA

ATTTTATTTATGAAAATAACTTGTTATTGCATTTAGTTTGGTTTATAAATTTTAAATTAA

AATAAAATCCATTTTAAAGTCACTTATTGTCTTAAATAAATTATTTACGAGAATTAAATA

AAATAAAGTGTTTTTTAAAAAAATATAAATAGTCATAATGAGAATTTGGTAAGAAAAATA

CATAATGGGAATAAATATGATTTTTTTTTATTAATATAAATTAATAGCATTTTGATAGAA

AAAATATTTTTTTAAAATTAATTTATATATAAATAATAAAAAAATTAATTATCAGTTCAA

ATTAAAATAATGATAAAAAATAAAATAGATTATTAATCAATGTTGATATGAAATTTTAAT

AAATAATATAATTATTTTTTATTTGTCAAATGTAATCAATTAAATTTTACTTTTTGAATA

ATTAGTACTTAAAGATAATATTAATTAAAAAACATTGTCTTTTTTAATGCAATATATGAT

ATATAACATTATAAAGTTTAAATTGTCATTTTGGTTTTCCATAATTTTTTAAATTTATGA

TTTTAATTTTCTTGTATTTTAATTATAACATTTGATTTTTCTAATTTTATAAATGAATAA

TATTGGTCCTTCTTATTAAGTTAATTATAAATTAACAAAAATTATTAATTATTAAAAAAT

TAATAATAATCACACTTCTCCATAATATTGTATTTTGTTTCACCTCCACTGCCACATAGG

AGTCCACCAATAGTAACAATATATTAGGATTAATAATTTTTTATTAATATTTTAATTATT

AATAATTTTTATTTAATTTATAATTAATTAAATATCTTTAATAATCATAATTAATAATTA

ATAATTTAATGAGGACCAAAAATCTCAATTTATAAAACTAAAAATAAAAAAAAATAAAAA

ATTTAGAAGCAACAAGATTATAATTTAGCATATGATAAAATATAATATATGATGATAAGA

AGAGGATCCTAGATGATTGCCCTTGATAAGTTGAGGTCGTAAAGATGGACGGCACAGGTG

TTCCCAACACTAGAACGAATGCCATTGGTTGGAACAAGCAAAAGGCGCATTTCTATTGGT

CCACGTCATCATTAGTGACATGTTCGTGCGTCAAAGCTTCAAGTTCAGGAATTGACCAGG

TTTGGGCACTACATAAAAGCC

>Glyma08g02960 chromosome:V1.0:8:2041506:2043506:1

ATTTTGATAGTGAATCATAAAGAATATGTTTCCAAATACTAATCTTATTGATATAAATTT

TACACATAAAACAGATCACGAATTCTCTTATTAATAAAATTTTATACCACTAATGTAAGT

CAATCCTAAAAGTTAAAATAATTTCAATTAAATTGATTTTTAGTTTTAATCTTACTTTTT

TAAAAAAAAATATTAAAAAATTATAAGATCCTGATTATATCTTAATTATATGTGATCGCA

TCTTACCTAATCATATTTTATCCCCAAATGTTATGGAAATAAGATATTGTCTTACCATAA

GTAGCAATTATATAATTGATACTCAACCTATGAACAAGAGTAATGGTTGAATGTTTGATG

GTTGATATATAAAAATGGGAAATGCTAAATATTGAGAAAGCTGTGCACATGCTCAAGGAT

TGGAAGCTTGCTAGAGTGGTGTCAGATATTGATCCAAGACGCAATCAGCTCTACATAGGA

TGGGGGATGTGCATCAGAGACTGAGTTTGTGGCTGCTAAGACCATGTTTTCTGAGTTTGG

TTTTGATGTTATACAAGGTGAAGCTTTGGGCCTCTTCCATGCTTTATCATGGGTAGTTCA

GCTGGCCTCCCATATGTCATTTAAGAATATGGGTTTTATCCTGTCAGAGTGCAAGACTTT

GCTTTTCTTTCATTGCAACTTTGTTAGGCCACAAGCAAATATGGTGGCTCAAGTGTTAGA

AGGGCATCCATTGGCTATGCTAGTCCCAATTTGTTTGATTCTATGCCCACTTGTATTTCT

CAGTTTGTTATTATGATGAGTTAAAGTTCGTCTTCTTAAAAAAAAATCATCTTTAAAAAA

ATGTTATTTAAAGTAATATTTTAACAAAATAATATTTTCATAAGTTTGTTTGTCAAATTA

TACTCTTAAATTTGTTTGGTAGACAAATTTATAGCTTAATTTATTATTATATTATTTATA

TATATAACTAAAAAAATAATTTATCAAAATACCTGAGTTGTAATAAACAGTGATATTTGT

CTTTGATATTTACTAATGAAAAAATAATAATTCATTTTTAAAAAAAATTATAACTTATTT

ATTTAAAAATCAATAAATTAACTTAATTATGTTGTTTATATAAAGAAAAAATATTTCATT

TATATATTTTTAAAAAATACTGAAATTCAAGTTAAAACTAAAGATATTAAAAATAATAGA

CTAAAAAAAAGATAAAATGTTAATTTAAGATTTATTTATTAATTTTGTTTCTATAGTTTA

TAAACTGATCTCTTTTAATCTCTATAGTCAATAAGAATATATTTTAAATTCCCAAAAGAT

CTCTTTGTTAATATTTTTTAAACTTTGTTAACTAAAAAATTTTAACGGCAGAGATTTTTT

ATGAATTAAAATATAAACGGTAAGGACTAAGAAAATATATTTATTAAGTATAATGATTAA

AAAAATATATTTGAAAATATAAAGATGAAATGAATAATTAAACCTAAAAATAATTCAGAC

GATACAAACATTTTACAAGAATATATCATATGCATGGGTGTTTGAAATTTGAAGATAAAA

TATAAATTTAAAATTAGTTTTAACTTTTGATATTTAGTTAAGTTAGCAAGTGTAAAATTT

GAAAGAAAAAAACAACAAATGATTATATAACACATCAAACAGAAATTTGAAAAAAAAGGA

CAATCTATATCCAAGAAATTAGACACTATAATTAAAAAAAAAGGAAAATGAATAAGCTTA

ATAAAATATAAAAAAAGGAGAGAGCTCTCAAAATAGGTGGATTAGAGTACAATTTAAAAG

ATTATCAGTGTTTAAAAATAAATTTATATTATAATTTAAATTTTTTATAATAAAATTTAA

TTGTAATTTGATTTATATATTTATTATTATATTATAAATTTCATTTGTATTAAATTAAAC

ACATATGTTTGACTAAAATCCTAGTTTTGAATAAATGAAGCAAAGAATGAGACGTGAGGT

GTCCCCAAGCAAAAGGACCAG

>Glyma08g06950

chromosome:V1.0:8:4984577:4986577:1

TGTTCTTCAAGACGTACAAAAGTTTTTCTTTTACAACAACCCAAAAAAATGTATACCCAA

TATATAAGTGAATTATTATTTATTATTCTGAATAAAAAAAATACTCTGGATAAAAGTATA

TTATTATTATTATTATCTTACCATTTTTATCTTTTTTTTTTCTATTTATTTTTTCATTCT

CTTTCTTTATTTTTTTTATATCTTCTTTCCTTCCATTCCACTTATCTTTATCTTTATCAA

AACTTTTATATCAATGACTTTGTCTTAATCAAAACTTTCATATCTCAACAAGATCAAATC

ATATTGAAAAGGTCTCACTAACACATCGTCAATAACTTTATATATAACGTGTTTTTTATC

CATCATCTAATTATTGAATTATAATTATATATTAATTTAATTGTTTATGTAAAAAAATGA

ATAATAATATAAAAATTAATATAATGATTTATATTTTAGTATATTTATAAAAAAATTGAT

TTAATGAAATAAGAAAAAGAATGTTTCCTTGGTAATAATATTGTTACAGTAAGTGGAAAA

CATGACACAAAAATTTGTTGATGGTAGAGTAGTCTGCTATCAAGGGCTGCCAAATAACAT

GTAGTTGTTCAGACGTGAGAGATATTACTTTATTACGAGGCCGCATTTAATAAGGTAAAA

GTTTTTTATTTTTTGAGAATTTTAATTTAGTTTCTCCTTGTTTGATATTAATTTTTAAAA

ATTCAAAATTTTGGAAAATATTATCTTTTAGAAATGTTTTTAGTTAGTTTTTTTTTAAAA

TCCACAGGGGAGGTCGGAATCATTTCTAACTTTCCTGGTTAGGAAAAAATTATTTATATG

GCAAGACTACATTACCCTTTTTTAAGTATAAAATATCCTCACATAGATTTCCTCTTTATC

CAAAAGCTTATTTCCATGGTTAAACATCTTTACTTGACCTATGACATTTAGTAATATTTT

TTTTATCTAATAGTAAAAATATTATTAAAAAGTTTTAATAGCATTTAATAAACATCATAA

CATGAGATATATACAATATTTAATAAAATATCATGAAAATATAATAGTAATATTTATTCA

TATTTAATAATATTTTTAAAATATTTTTTAAAAAAATTTAACAATATTTTTACTAAGAGT

TAGAAAAAAATATTATTAAAGGTTATATGTATAACAGTGATTCAACTTATCCACATGATT

ATATCTCCTTTTTTTTAATTTTGAAATTTGCAAAAAATCTCTATTTTTCTTTTTTTATAA

ATAATTTTATATTTTTTTAAACGTAAATCTCCAACTACTTTATATTTAAGTTTATTTAAT

TATTTATTATTTCATCTTGTACAATATTTTGTAAATATTAAATGATTTCTATATTAACCA

CCTAATATTTCTTCTATGAATTCAAAAAATCTCTTTAACAATTATATTTATTTTGTTGCA

GGACCTCCCTTACAGTAGAGTTTAATAAAATTATTTTAACAAAATGGTATATTTGTAAAA

AAAAAAAAATCGTATACATCCATGCAAGTTCTACTATAATCAAATATGAAAAACTTTAAT

TCCAAGAAACATATATAATCAACCAAACGCATATTAATGATTCTCAAGAATCATAATGTC

TTATTTTCATATTGATTATGATATTTTCAGGTATAAAAAACCTTTTTCTCTACTAGATGC

TTGTGATCTCTTGACATAAAAAAAATTTCTCCCATACTAAACTCTCCCTAAAGTAAGGTG

ACATGGTAAAAAAATTAATCAAGTATGAAATAGAAATTTCATATACATATAATTTGCCTA

ATTCTTATTCTTATATTTAAATATGTATGGATTAACTTCATGCTAAAGAATAATCAATTG

AAAAATCTTTTTTTTTTTACTTTTTCTTAGTATTCCCATATTTCGTGAGATAAAAATAAT

TTTTGTATGTTATTCAAACACACTAATCAACATCAGTTCAGCTCGCCTGTTCCGATGGTT

CTGCTGCCAAATTTTGGCAAA

>Glyma08g22100 chromosome:V1.0:8:16777202:16779202:1

TTTTAATATAAAACATCTATCAAATAACTCAAATCCTTAAGATTTTATTAGACTTTTTTC

TATTGGTTCTCTCATCACACATATCTTTTTCTCTGGTCTTCAAGATGTGTGTGTCATATA

TTTTTTTCCTATTCTTTTGGAATAAAAAAATATCAACTATATTCCTTCCCTTTGTATTTT

TCTCTTTATTTTCTAAATTAAATTAATATTTGTCTTATTTGTTAAGATTAATTATGCTTT

CCTTATATTGACTATGTTCATTACTTAGTCAAATATTTTCATAATTGTCATCCTTAATTT

TGTCATCCACACAACTCAATAACTTTCTTACTCATTTTCCCATCCTTAATTTCATGTTAA

GACTAATCATGCTTTGCTTATTATTGCATCCCATCTAAACATGTCATTAAATTTATCATG

AAATAATTATAGTAATTAAAATAAAATAAGAAAATCAACATATAATTTTAAATCTATTGT

TCAAACCCAAAAGTGAGAATGAGAATATGCTATTGTAAGAAATGTTACTATAGGAGAACA

TAAAGGTATAATCAATATAGCTATTAAAAGATTTAAAAAACTATATTGGTTTTACTTTAT

TTTTTTTATCTAAATCCCATCATTTTTAATTATTTTTTTCATTAACTCTTGTAATTTTGA

TTGTGTTTTTAAAAATTCATTGAATCCTTTCAAATCTTAAAAATTTATAAAATCATTTTA

AATCCTTTAAATTCGTACGATAAATCTATTAAAATTCAAACTACTATAGAAGTCTTCTAA

AGAAATTTGATTAGTCATAATATTCATACATAATATTCAAAATCAATAAGACTTTTTCAT

GCTAAAATAATCTTTTAAATTTTTAATCCAATATACTTTCTAAATCAAGAGGAGTACTAT

CTTTGTTCTTATTTGTAAGACTCAATTATCTAATTCATGATTAAAAATTTATATATTTAT

CTCTTTTAGATGCATGTGAATACAAATCTCTATCTTATTTAATTATGAGTGTTTTATTAT

AAAAATAATTAATGCACGAGATAATATGGATTTAATCTTATATAAATCAACAGACACGGT

TAAAAAGCTTGTTCTTATAAATTGAAACAAAAATAGATCTTTTATCAAAACTAGCCTTGT

GTTGTCATTAAATTTGTTATTTTGTATCTTCCACTACAAAATAGAAAAAATTATTAATTG

TTTTCTATTAAAATTAATTAAGGATATTTTAATAAAAAACAATACAAAATATTATAAATT

AAGTCTTATAAAAAAATAAACATAATTTTAATTTAAGTTTTATAAATAGAAATTGAAGAT

GTAATTTATTTAAAATATACACATAAAAACTCATATTTTCACACAAAAAAGCTCATATAA

TACAATTTTTAAATTTAACAGTAATATAGCTTAGTAATATTATTTAAATTATTTACTACA

TTTGTTTTTAAATATAGGATTTTTTAAAAAACAGTTATCTCTTTTTACAAGACTAAGGGA

GAGATATTCAATTACGATTTCAAAAGATTTTAAAAATTATTTTTTTATAATTTTTTTAAA

AATATTTAATCAAAATTTTTAAATAATAGAAATACTTCTTATAGTATTCAATCAGGATTA

TTAAAATATTTTAAAAAATTCAATAAAGTAAGCTGATATTCAATTAAATTTTTTTATAAT

TTTAAAAAAATCTTATAATATTTAAAAGTATATAAAATTTGATAAATTCTTTCTTAACAT

GAATTTTAATAAACTTCTAATATCAATATCAGTTAGTTACTTGACAACAAGAATTAAAAC

TAAATTTTTTAAAGAAACTAAAACCAAAATAAATCCTTAAAAGACTAAAGTAAAAAAAAT

ATTATTTTATAGGGAGTAAGTTATTATTTAAACCAATAATTTGATTATGATATAATGATT

TTAATTATTTGATTATATAAAATAAAAATTTATTTAATGGTATACAGTATGAGGGAGAAG

ATGCTTCTAGAAACAGTTGGG

>Glyma08g42720 chromosome:V1.0:8:42701873:42703873:1

TAAATAAAGCCTCTAACTACATAAAAAGACGCACCACATTAGACAATGATGCACTTCACT

AAATACAAGGTGGTTAGTTTTCTAAAGTTAGATTGGATTGCTACCCAAAGAAGATAATCA

TAATGCTTTATCAATTGTGGGTGATTGAAATATGACAAAAGTAGGCTATTGTGGGAGAGT

CGATTAAGTCTCCTACAGTGGAAAAGTCATTTTGACCATAGGATGTGTGAGACTACCGTA

AGATTGCATACATCGGATTATCATTTTTTTTTAAATGCTCTACTTTCTTGTCATGCAAAT

TCGATTCATTGCCCCTCCACTTCTCTCTCCCTTCCATCTATTTCTTCCTCCTCAAATAGT

CAAGCTTCTCCAGCCCATCTTCCTCTACCAACAACTTCACCAAAACCCACTCGAACAAAC

TAGATTCGAAGTCAACCCATTCCCACCCGTGGCGCCTCCCTCTCCCATTCACACCTTTCC

ATCAGTGTCCTTGTTGGTGTCAATAAGCTATCCATGGATGCCTCCCACGCCTTCTGCAAG

TTCACGACAAAGATAAAAATATCCTAAGTAAGGTCGACCACATTAAGATCATAGAAGTGT

GTTTATTTTTTTTTCTCAATTTATGTTTTTTTTATATATTTCAATTTATTTTATGGTGAT

GTTTGTAATTTAATTTGGGATCTAGTGAATATATGATGATGTTTGCAATTAAATTTGGAA

GCTGGTCAATAAATTGTGGCTTATTCAAAATTGATGACATGAACAAGGAGATAAATACAC

TAATAAAGAAATTGGAAAAGAGGGCCAAAGCTCCAAATCAAAGAAATGGAGAAGAAGAGA

GGTCATCTAAAAAAATTAAGTGTAGCCATTTTTACTATACATCAACACTTGTCTCTAGGA

AAAACACATTTTACAGCCAAAATGACTTTTCCCACATGTGAGGGACTTAATCTACTCTCC

CATAATAGCCTACTTCCAAACATTAAATGCTTCAATACTAACCCAACTTCAAACAATAGA

TAAAACACAAAAAAGAATATCGTTTGGAGACAACGATTACTTTATCAAGCAAACCTATAA

ATATCGCTTATGGAACATTATTGTTTTAATGTGTTTAAAATTATCAAAATAACCTCAAAA

CTTTTGGAGGAAAATTTTGAACTTGTGTATATTGCTTTATATAATAAAGATATATTTTAA

ATTATTTTTTTTTGTAATTAAATAGTGCATGAAACACAATTAAAAAATATTTTAATTACA

TCTTTTATTTGTGAACATTATTATTTTAATATGTTTAAAATTATCAAAATAACCTCAAAC

TTTTGGGAAAATTTAGGCTTGTGTATATTACTTTATATAACAAAAATAAAAATAAAGATA

AAGATAAAAAATAATTATTTCATCAGAAATTTGTAATGATACATCTAATTGCATCATTTT

CATAATATATTTTTAATTATTTTATTTGTAACTAAGTAGTGGATGAAACATAATTAAATA

TTTTAATTGCATATTTTGTTAGTCAACATTGTTATTTTAGTGTAATTAAAATTACAAAAT

AATCTTATTGGGGAGAAATTTGGACCTCTGTATATTACTTTATACAATAAAAATTTAATT

ATTTAAAAATAAATTTATAATTAATATGATATGTCTAATTGCATCATTATCGTAATATAT

TTTAAATTATTTTATCTATAATAAAATAGTGCATGAAACCTAATTAAATGACATTTTAAT

TGAACATTATTGTTTTAATGCATTTAAAGTTATCAAAATAACCTCAAACTTTTGCGAGAA

AATTTGGACTTGTGTATATTAAAAATAAAGATACATTAAAACAATGGGAAATAATAAAGA

TTATATTAAGATATATTAAGCATTAATATTTTTATTAAAACAAATTATTTATAAATATGC

AAACCTACTTTTTTTAAGTAAACATATCACCCCACTTTTATATATTTAAAAAAAATTCTG

AATTAGAAAAAGAGGCCAAAG

>Glyma11g14950 chromosome:V1.0:11:10699039:10701039:1

TTCTGGAAGGTTCGGTTCATGCGCTGTGAACCGTTGGATCAGTAATTTTGGACGGTAGAT

ATTTTTGTTCCTAATGGAATTGGACTTGGGGAAAAAGCGAAGTGTTGTGTGGGGTTTCTC

CAACGTTCTCTAGGTGATTCGAGAATTTTCCGATTACGAAACGGTGCTGGCGTGGCACGC

GCTTAAACTAAAGATTTTTAACCGTTGACGTGAACTGGTCGCTTTTTTTTTTTTTGGTAT

TGGATAGATTGTATCTGTGATTCTCCCGCGCCATTGGTTGAATTAATGACGTCGTCCGTG

CGCTTTAGATAACTGGCCAATTGGATTTCACTAACTCCGTGTAAAGCGCGTTAGTTAAGT

TTTATTTTTGGTGTGTTTCTTGCCCGTTCAAAAAGCATGATTTTATTATCTTTTCAAAAT

TCACTCATTGCAGTAAATCAGTAATCACCCTGGCTTTTACTTTTACGATTTTCTTTTCAA

TTCAATCGTTCTCTAAATTATATCTTGAGTATATAAGATAGTAGCTTTAAATTTAACCTT

AAAAAATAATAATAATAACAATATTTTAATTTAATATTTTAAAAGTGTAAAAACTACTAA

ATAAAAAATAATCTACTCTTATCTTATCAGATATATGTTCAAGATATTTTAAAAATCAAT

GATAAATTAATATTTTTATTTAATCTTTTAAAAGATAAAAAAATACTAAATAAAAAATAA

TATAGTCTTATCTTATCTTATAAAATACGCAACTAAAAAAATCATCAGTTCATATTTTCA

AAATTTACTCGTTACTAATCAAATTCTTTTTTTACAGTTTTTTATCCTCCTCCGATATGT

TTTTTTTCTTTAAAATATATTTAACATAAAAAAATAGTAATTAAATATTTTAAAAGAAAA

TACTGAATAAATATCACTTGTATAAATTATAAAACTTTTTATGAAGGACTATAAATTTAA

ATACTTATTTACCTTATTCTCTTCATTTTCTCACATTTGATTTGCTTAAATTTTTTCTCA

CAAATAAAGATAAAATTCTTATTTTAATTATTTTTTCTAATTCTTTCATGTTTTTCTTGA

TTTAAACTAATGTGTTTTTCTCATCTCCAATTTCACAAAGATTTTCTTGAATTTTTACAT

GTTTAAATGAAATTACAAATCTGATGGAGTTTTTGACATTTTTACAGTATGTTTAGATAG

AGAATTTTAACTGAGAAAAATACTTTATCAAAGAATTTGAATTTCTGTAATCTATAATTC

ATTGTTTGAATGTTTTTTTTATGAAGAATTTAAAATTTTGGAATTTTAAAACAGAATTTT

AAACAATTAAAAAGGTGAGAAATTGAAATTCTCTTCTTATCAAGAAACACCGTCTAAGAT

TCTGGAACATTGATTGTTGGGTCTCGGCATAGAGGAACTAGCACACCAATCATATCTTTT

CTTTTTTTCATTCATTTTTTTTCACCCTCATAATTTTAACTTTTTTTATCCAAACACAAA

ATTTTGAAAATAAAAGAATTTCAATTGAAGTATTTAAAATTTTTAGAATTAAAAATTTCT

CAGAATTTTAAATTCCTCCATCCAAACACACTCTTAAAATATATGGTAATATCTCTTTTT

GCAACTTGTTTAAATGGAATTACACATATAAGCAAAGTGCATTGCAAAACATATCTAGTG

TGTTATGTGGTTTAAAGAAACGAGAGGGCGGGGGGAGAGAGAGAGCCCCACAAAGGTGTT

GAGAGAATAACAAATAACACATAGAAGGAATAACAACTTGGCTCTTTGATGTCCCATGAA

AGGGCACTTGGGTCTCTCATAAATGCAAGCTTCCTATACGCCCTCTTTATTTGCTCATTT

GATGCACTCTTCAACACTTTAAGTATACTTTAATAGCTCTTTTTGCAACAGTTTTAAAGT

TATCTTCAAAATTAGTTAAAAAATTAATTAAAAGTTAAAAATGAACTAATAACCAATGAT

AACTAATAATTAAAAGTTAAG

>Glyma11g31670 chromosome:V1.0:11:33021169:33023169:1

CCTATGGCAATTCTATGACCTTCTTTAGCCATTGAGTTTGAAGTGAATGAATACTGAGAT

ACACAGAACAGAATGACAGAAGTATTTCTGAAATACTAGTACTATGAAAATTACCTGAAA

CGCTTAAAACTATACTTATATACATGTGTGCGTGTTGATTCCACAAAGTGAAGTTGCACA

TGCTTAGTCATCGGAAATCAAGACTTATAATCTCATTTACAGCGTCTCCTCGTGCACAGT

ACACTGCAGTATGAACATTGGAAAACTAAAAGACTGAGGCTGAAAGCCTTCATTCATTAA

TTTTCGTTCTTTAAGGATAGTATAAGTAATCTTTTATTAGATCGACTTGGGAAATGATGA

CCCAGTAAAATGATAACTTAATTATATCCTTTAATGGACCAATTATGCAAGTTTTGCCCT

CAAATTAAATTATAACTTAAAGAATCCAATTAATCATACTTTAATAAATTATTTTCCTAA

AACTATATAAAACTTCGTACCCCATTGCCCAGAGGCTCTTCGCTATGCGAAGGTATGGGG

GAAGGATATTGTACGCAGCCTTGCCCTTGCATATGCAAAGAGGCTGTTTCCGGATTCGAA

CTCATGACCAACAAGTCACCAAGGCACAACTTTACCGCTGCACCACGGCTCGCCCTCATT

TTCCTAAAACTATATACCAAACAAAATCTTTTACAAAAACATACTATTAAAACAAATTGT

CGTAGCCTTAAACCATATTTAAATTAGTGATCATATTGTTAGGAAGTCTCACATTTGTGA

GGGGTTAGAAAGTCCTACATCATCTGTCTCAATTCTAGAGATGCAGCTTATATATTTGTT

GGACAATTTTACTTAGTGACTAAAATCTGACACATGACCCACTAATTTTTAATTTTGATC

ATCCTTGTATAATTACATAACCTAGATTTATAGTTATCATTTCTCACACTCCTCCACACT

AAGAAGAGACTCAAAATTTCTATGCTCAGTTGCAGAATTCAAATATTAATTTATATGCTC

GGGTCTTTTCCGAAATCATTAAACAAATATCTAGCCTTTGAAATCAATTCTCCAGAGAAA

ATGATATTGATTTATATTGAATGATGTTTTTGTTACTGCAAATTGCTAGTGTGTTTTTTC

CTAAAAAATGTAGCAGAATCAGAATAAGAATATGCCTTTGTGCAGAGGTTACTAATCACT

TGGAAGCAAAGATTAGTTTGTCCATTAAGTATAGAAAGAGGATGCAAACTAAGAACTGTA

AAAAAAACACTTAAACATTTGTACTCTTAAAAATAAGTGAATGTGTGACCTGCAGATGAT

CACTTTAGATCAAAGATGATTGCTTGTCAATAATTATTACAGCAAGAGCCGCAAACAGAG

TAGTCAAAGAGGCATATTACAAATACACACTTAAAACATCTTAATCTTAAAATATAAGTG

ATTCTATGAACTTCTGCAGATGATCATTTAAGATCAAAGATGATTGCTTTTCCATAGGTA

CAAAAAGAGATGAAAACTGAAAACTAGTCAAAAAGGCATAATACAAATACAACTAAATAT

AGGAAAAACTGTAGAATCATTGTTACCACTGGATACATAGCCAAGCTCAACATTGCAGTG

AGACTACTAATAAATGCACCCAAATAAAGGCGATGGCTACACAAGTTCAATGTACGCCAT

AGGTGCATTATCACCCCGCCTTGGTAGGGTTCTTATAATTCTTGTGTAACCCCCATTCCT

TTCGCCATAGCGTTCTGGAACCTCCGCAAACAAAGCATGGACAATCTGCTTCTCATAGAT

GAACCCAAGGGCTTGTCTTCTTTTATGAAGAGAACCATCCTTTGCCAAAGTGATCATTTT

ATCAACAAACTTCCTAATTGCACTGGCCCTTGCTCTGGTGGTCTTTATGCGACCATATTT

AAGGAGCTGAGTTGTAAGGCCTCGGATGAGTGCGCGGCGCTGATCAGGGGGTCTGTTGAG

TTTGGGCACTTTCCTCCCATG

>Glyma11g31673 chromosome:V1.0:11:33021200:33023200:1

TTGAGTTTGAAGTGAATGAATACTGAGATACACAGAACAGAATGACAGAAGTATTTCTGA

AATACTAGTACTATGAAAATTACCTGAAACGCTTAAAACTATACTTATATACATGTGTGC

GTGTTGATTCCACAAAGTGAAGTTGCACATGCTTAGTCATCGGAAATCAAGACTTATAAT

CTCATTTACAGCGTCTCCTCGTGCACAGTACACTGCAGTATGAACATTGGAAAACTAAAA

GACTGAGGCTGAAAGCCTTCATTCATTAATTTTCGTTCTTTAAGGATAGTATAAGTAATC

TTTTATTAGATCGACTTGGGAAATGATGACCCAGTAAAATGATAACTTAATTATATCCTT

TAATGGACCAATTATGCAAGTTTTGCCCTCAAATTAAATTATAACTTAAAGAATCCAATT

AATCATACTTTAATAAATTATTTTCCTAAAACTATATAAAACTTCGTACCCCATTGCCCA

GAGGCTCTTCGCTATGCGAAGGTATGGGGGAAGGATATTGTACGCAGCCTTGCCCTTGCA

TATGCAAAGAGGCTGTTTCCGGATTCGAACTCATGACCAACAAGTCACCAAGGCACAACT

TTACCGCTGCACCACGGCTCGCCCTCATTTTCCTAAAACTATATACCAAACAAAATCTTT

TACAAAAACATACTATTAAAACAAATTGTCGTAGCCTTAAACCATATTTAAATTAGTGAT

CATATTGTTAGGAAGTCTCACATTTGTGAGGGGTTAGAAAGTCCTACATCATCTGTCTCA

ATTCTAGAGATGCAGCTTATATATTTGTTGGACAATTTTACTTAGTGACTAAAATCTGAC

ACATGACCCACTAATTTTTAATTTTGATCATCCTTGTATAATTACATAACCTAGATTTAT

AGTTATCATTTCTCACACTCCTCCACACTAAGAAGAGACTCAAAATTTCTATGCTCAGTT

GCAGAATTCAAATATTAATTTATATGCTCGGGTCTTTTCCGAAATCATTAAACAAATATC

TAGCCTTTGAAATCAATTCTCCAGAGAAAATGATATTGATTTATATTGAATGATGTTTTT

GTTACTGCAAATTGCTAGTGTGTTTTTTCCTAAAAAATGTAGCAGAATCAGAATAAGAAT

ATGCCTTTGTGCAGAGGTTACTAATCACTTGGAAGCAAAGATTAGTTTGTCCATTAAGTA

TAGAAAGAGGATGCAAACTAAGAACTGTAAAAAAAACACTTAAACATTTGTACTCTTAAA

AATAAGTGAATGTGTGACCTGCAGATGATCACTTTAGATCAAAGATGATTGCTTGTCAAT

AATTATTACAGCAAGAGCCGCAAACAGAGTAGTCAAAGAGGCATATTACAAATACACACT

TAAAACATCTTAATCTTAAAATATAAGTGATTCTATGAACTTCTGCAGATGATCATTTAA

GATCAAAGATGATTGCTTTTCCATAGGTACAAAAAGAGATGAAAACTGAAAACTAGTCAA

AAAGGCATAATACAAATACAACTAAATATAGGAAAAACTGTAGAATCATTGTTACCACTG

GATACATAGCCAAGCTCAACATTGCAGTGAGACTACTAATAAATGCACCCAAATAAAGGC

GATGGCTACACAAGTTCAATGTACGCCATAGGTGCATTATCACCCCGCCTTGGTAGGGTT

CTTATAATTCTTGTGTAACCCCCATTCCTTTCGCCATAGCGTTCTGGAACCTCCGCAAAC

AAAGCATGGACAATCTGCTTCTCATAGATGAACCCAAGGGCTTGTCTTCTTTTATGAAGA

GAACCATCCTTTGCCAAAGTGATCATTTTATCAACAAACTTCCTAATTGCACTGGCCCTT

GCTCTGGTGGTCTTTATGCGACCATATTTAAGGAGCTGAGTTGTAAGGCCTCGGATGAGT

GCGCGGCGCTGATCAGGGGGTCTGTTGAGTTTGGGCACTTTCCTCCCATGTCTCATGGCA

GAGACTCGACCACCATTGTCA

>Glyma11g31810 chromosome:V1.0:11:33163094:33165094:1

AGGCAAAGTCCGTTATGGAGATGAAGTTTTGATGATAACAAACTAGAAGGAAGTAAAGTC

CTAAGTATGGATGTTCATATTTGATCAGACTAAAAACATCGCAAATCGATAAAAAAATAA

ATGAAAATTGATTTTCGATTAGTCTTTGCGAAATCAAACCAATCAAAACCAAATAATATT

TTTTTAATCAAATCAACTAAAAAGATGAAAGGACTATTGCATAAGCATTGAAGTAACCAA

TAAGACAATAACATCTACTAGTAGAACTTTATAGTAATCAATCATCAAAAGCAACATTCT

TGACAAATTTCGTAAGTTGTCGATGAATGACTCACTGGAAATCCACTCACATCAGTTGTT

ACATTTTCAAATTTAAACTGTGTTTGCTTATTTCAAAATAAGGAAGCAAAAATCACAATG

AAAAGATTTAAGAAGCAACAAAAAAGGCAAAATAGACATTCAAACTAAAATTGTGTGAGA

GGGATAACTCACCACTACTTGGTTGAGTTTAAATGAGTTATAGATGGGTGGGTTGGTGAT

GGTAAAAATATTAAATTTTGGGAGAATAAATGGGTGGGTGATATGCCTCTTCAAAATTTG

TTCGCAAGAATTTTCACAAATTTGGAACAAAAAGAAGAGGTGATTGGGAATATGGGAGAG

TGAAGGAATGGTAAGTAGGAATGTGGTTTTGTTTGGAGAAGTGAGTGGTTTGAATGTGAA

AAGAGGGTAGTGAAGGATTTTTTTAGGGTGTTGGAGGGTGCAAATGTTGGCAACGGAGGG

GAAGATCATTGGATTTGGTTAGTAGATACTTCAAATTCATACACGGTGAATTCGACATAC

AAAGCTTTGTATGGTTCAAGGTTTGACTCATCCGAGGTGGTTTTTTTAAGGCTATGTGGA

AAATTAAAATTCCTCCAAAGGTGTCATTTTTGTTGTGGAGAATATTCTTGAATAGGATCC

CAACAAAAGTGAATCTACAACATAGACAAATCCAAATTCAAGATAAAAAATTATTGTGCA

TGTTTTGTAAAGAGGAGGTAGAGGATTTGCCCCATCTATTATTTAAATGTCCTATTTCAC

ACAATATTTAGAAATGTTGATACAACATTATGGGTTTAAATTCTGCACTGCCTCAGTTAG

TATTGGATCGTGTTTTGCAACATGCACATGGTTTGGTTGGGATAAAAGAAAATGATTGGT

GGGTTGTGGAATGGTGTGCTATCCTCTAGATTCTATGGAGTCAACGAAATGAAATGGTGT

TTGAAGGAAAAGAATTTGACTGGGAGAAAGTTTGACACAAAATCACGTTCTTTGTATGGT

CATGGTTAAAGGCTTTTTAAGGAGATTTTTCTATCACCTATCAACTTTGGCAAGTTGATC

CAAGTGCTTAGCAAATTTTAAGTTTGTAGGGTGATTGAGTATGAATTTTGGTTTGATTTT

AGAGTTGAGGTGTTGTATCTTTATTTACATAACTTTTAGTGAGTATATAGTAAGATTTAG

TTTCTCTTTGATGTATTTGTCTTTGTTGCTTGATTAGAGTGAAAGTATTAATATGAAGAG

TTTTTTGTTTTTATCCTCCTTGAACATGATTAATATGGTGTTTGGTAAAGAGAAATAAAA

TGGAATGAAAAATGAATTGAAATGAAAACTTTAAATCAAAGTGGAATATAAAAATGTAAA

TTCTATTTTATTTTACGGTTTATTTCATCCATTACGAATTATTTTTCACTCCTATCAAAC

AAGAGATAAAAAAAATAAACATTGAAATATGTTTCAATTAATTTATTTTATATTAAAATT

ATATCAGGATCTTTTAAAAAATAATACAAGACTATTTTTTTTTATTTTATAAATCAATCA

AAATCAAAGGTCCCTTTCCTAAGCCTAATTGGACTGAAAACAATAGGCAAAAAGAAAAAA

AAAAAAGTCAACAAATACTCTATGTACGACATCGTTCTAATACATGTAAAGTTCAAGAGC

TTTTTTTTTTTTTTTTTCTTA

>Glyma12g06910 chromosome:V1.0:12:4709528:4711528:1

GTTTGAATTCTGGAAGGTTCGGGGCATGGGCTGTGAACCGTTGGATCAGTAAATTTGGAC

GGTAGATATATTTGTTTCTTAATGGAAAAAGCGAAGTGTTGCGTGGGGGGTGTGGGGTTT

CTCCAACGTTCTCTAGGTGATTCGAGAATTTTCCGATTACGAATCCGCGGTGGCGTGGCA

CGCGTTGGAGCTCCACGCGCGTAGACTAGGATTTTTAACCGCTGACGAGGGGAAGTTGCT

TTTTTTAGTTTATCTTTTTGGTATTGGATAGATTGTAATAGTGATTCTCCCGCGCTATTG

GCTGGATTAATGACGTCGTCCGTGCGCTTTAGATAACTGACCAATTGGATTTCACTAACT

CCGTGTAAAGCGCGTTAGTGGTTTTATATTTGGGTTTGTTTCTTGCCCGCTCAAAAAGCC

TGTTTTATCTTTTTCAAAATTCACTCATTACTAATCAGTAATCACCCTGGTTTTTACGAT

TTTTTTTTCTTTTCAATCCTTCTCTAATTATGTTTTTCCTTTAAATTATATCTTGAACAT

ATAAGATATCTCTTTTAATTTAATATTTTATAAATAAAAACAATTTAACCTTTTAAAAGT

GAAAAAAGAAAATACTAAATAAAAATATATCTACTCTTATCTTTTCATATAAAATATGAA

ATTAAAAGATATCTTATGTTCAAGATATTTTTTATAAAATATTTTAATTTAATCTTTTAA

AAGAGAAAGAAAAATACTATAGAATACACAACTAAAAAAATCAACGATTTATCTTTTCAA

AATTCACTCGTTACAAATCAAATTGTCTTTTTTACAATTTTTTATCCTCCTCCGATATGA

TCTTTTTCTTTAAAATATATTTAACACACGGAATAGTCTAATTAAATATTTAGAAATAAA

TTACTAAATAAAAAACACTTATATAAATTAGGATATTTTTTTGAATGACTATAAACTAAG

ATACTTATTTACCTTATTCTCTTCAATTTTGTTTTAAAAAAATTCTCACAAATAAATGTA

AACTACGGTCCCTTATTCTCACAAATATATATATATATATATATATATATATATAATATT

CCTCATTCGTTCTAAAAAAAAACTTACCTCCCAATATTTCTCTAATTTAATTCTTATTTT

AAATATTTTCCTTATTCTTTCATATTTTTTTTTATTTAAACTAATGTGTTTCTCTCATCT

CCAATTTCACAAAGATTTTCTTTAATTTCTACACGATTTAACGAAATTATAAATCTGATG

CACCTTTCAATACTTGAAGAATATCATAATACCTCTTTTTCTAACACGAGATTAAACGGA

ATTACAAAGTGCATTGCATAACATATCTGGTGTGCTGTGTGGTTTAAAGAAATGAGAGAG

CGAGATAGTACCCAACAATGGTGTTAAGAGAATGACAGAGAACAGATAGAAGGAACAACA

ACTTGGTTCTTTGATGTGCCATGAAAAAACTTGGATCTCTTAGAAACAAAACTTTAATTT

TAGGGCTCTTCTATGCTCCTTAGAATCTCATTGGAATTGGAATAAAATATGAAGATTATG

GTTTTGGATGAGAAATTAAATGTGTGTAGTGTAGTGTGTTTAAGGATTATTCAATTGTTT

CTTATATTATGTGTGGTGCTTATAGAGTGGCTGATCTAAAAGTGATTTCACCAAGACTTT

ATCATAGAGTCAACAATACGTTTTGGCTTTACAATGTGTTTGCTTCGAAACTGGTCAATC

ATAGTGTGAGTGTGGGTGTGGGGGAGGGGGGTCAAGAGTGAGGAAGTCGTAGGTGGGGGA

GGTGAGGGACACAATGTGATGGCCCAGTGCGAAGGAACGACCATATAGGTGAAGAAAAAA

AAACATATTTTGAAAATTAGACATTTAGTTAATGTCATCTTGACTAAGGGACTAAAAAAA

TATTTTTTGAATTTAATGAACTAAAAAAATATTTTTTGAATTTAATGAACTAAAAAAATA

TTTCAATTTGAAAGATTAAAA

>Glyma12g28750 chromosome:V1.0:12:32103319:32105319:1

AAGTATGAATTGAATATTCAACAGGGTGGGGAGAAGATGAAATGAATAGGTACTACTACC

ACCTTCCTCAACCTGCCACACTTCCCACTACAACTACTACTAGGTTCTATTAGTTTTTGT

GATTTTCTTTATTCATTTCACATTTTAGTTAGACATTTTCACTTTGTTCAAATATTTTGT

TGTTGTTATAATCGTGACCAAAGGGGTATCATTTATGCCACGGTCATATCATTTTAATTA

CAAATTTACAACAAAATTATTTGCAAAAATGTAAAGTTTTAGAGATGTGGTTAACTTCCA

CTTCAATTTTGATTTTTTTTCTTTCAATGCATTGGTTTTTCTTTTTCTTATTTTAAACAA

ATTTCAATGCCTTCTTTATTGGTTTTCACTTTTTATTACTCTCTTTTACCAAGGCCACAA

GCTGTTATATATTGTTTGTTCTATGTCATATTTTGTTCTGTTATGAAACTATTCTATGTT

TTGTGAATTTAAGTAGTGCTTCAATTCCTTGACTAATTAAAGTCATTGGAATAATGTCTC

AATGTCTTATAAGCGGTGCTGAGAATCTTGAGACTAAGTGTTTTTAAATGATTTTTAGTT

TTTTATTTTATTTTTAAGATACTTATTTAATAATTTCAGGAAGGAAACTACAATGGTATA

ATTCTTAAATTATTCTCGATGAAGGGAAATGGTTTTCTTTTCAACCTTTCATGTGTTGTT

GTTAATTGAAATTAAACTTTATATCAGTTGAATAATATTTACTTGTGTCATCATTGATGC

AATTGAGTGCCCATCTAACTGCATTAGCTAATTAGTCTTCAAATAGGTTTAATACTATTA

TGGTTTAATTAACAAATATATGTTGCCTACTATTGGATATTAGCATAGTGCACATAGACC

AAACTTTTATATTTCATAGTAACCAATTACTATATATTGGCTAGAATATATAGTGAGAAA

AGTCTTATTTCTTATCATCAAATCCTTCTAGCATCGATCCTGGATGAAAATATTGTACTT

GATGACAAGGAAATAGTTGTGTAAGCAATTTCTGTGGCAAGGTTACAAAAGGTGGAATTA

CTAGAGCAAAAGAACAATTGATAGCAAACTCGGGGAATGTTGCTCCCAAAGAAGTTAGAG

AAGAACTATGGAGATGTTTGAAGGATAAGAAGAAACAAGAAAGGAAAACATTTCATAAGA

TGCGTCAACATCTTCTTGAAGACTATGGTGATAGTGATGAAGAAAGAACTAAAAAGGTCA

TGTGTTCTCACTTTCTCTTCATGGATTCAATTTTTAATACTTTTATCATGAGACACCCCT

TTTTAGATTATCAAATTCATCAAATTGGGATAATTTTTTAAAAAAATTGTGCATTATTTT

TTAGTTTCTAAGTAGGTCATTAAATTTTTTAAAAAATTAATCATATAAATCTATATCTAT

CTATATAAATAGGCTGGTCAAAAACCCAAAATAAAAATCTTATATTGTTTACACGTCCCT

TAAAATAAAGATAAAATGAGAATTTTTAAAAAATAATACTATTAGGTGTAAAGACTGGAT

AGAAGAAAAGAGAACAACAAAAGAAATAGATAAGACGGAGAAAAAATCCAAGTCTTTTTA

TTAACAAAAAGTAGTTTTTTTAAAAAATTATTTTTTTATACTTTTTGGCAAATTTTTCTT

TTTAATTTAAGTATAATTTCAAAATTGAAAAGAAAAAAATATACAATGACCCGTGCTGTA

TGTACGGATAAAAGAAAATAAGAAGTTTGAAATGGAAATGTGTAGATAGGATCGAATGGC

TGGAAATGGGGGTCTCTGAATCGAACGGGAAAGGAGGAGATGGTTCTAGAATATATACAC

GTGGAAGAACTCGCAACATAACATAACCCTCATCCTCTTCAAAAACCCTTCTTCTCTTCT

CTTGGTTTCGTTCTGTGCATCTGCCAAATTAGGATTAGGATTAGGGCTACCAATACCAAT

ACCAATACCAATTCTATTCTA

>Glyma13g10700 chromosome:V1.0:13:12771269:12773269:1

CCAACTGTGATCACCATTGGGTCATCTTGCTTGGGATTGACTCCTACAAAGTCGGTGTCG

GTGAACGTTATCAGTTGGATTGATTTCCTTGAGCTCGCAATGGAAATATGGTTCATGCTT

TTGATGGGGCGCAAATGCCTCTTTTCTCTACTATGCCAAACGTACTTGGTTGCGGAAGGC

GAATAACTCCTCCATCTGGATGTATCCCGCTGCCTTTGCTCGAAGCTCATTTAGATATTT

GGGTTGCTTTCGACAAAGGCTATTAGTGAACGACCCTAGCTTAAGTGTCATGATCTTAGA

TGGTAGTGTGACACCAGGGTCGAGGTCTCTAATTCTAATGGAGATAGTGGCGTACCTCTC

CATAAAAGACTACAATGACTCATCATTAGCTTGGCAGAGGTTGACCAGAGTGGTGAATGT

GGTGTGGTGAGGGCGGCTAGTTGTATATTATGCGCCGAAGCATTAGGCTAAGGTGAAGAA

AGAGTCAATGGATTGTGGGGGTAGACGTTTGTACCATGTTAGTGCCTGCCCTTTGAGGGA

GGTAGGAAAGACCTTGCATATCACGGTGTCATCATTGAAATACAAGTTGACTTGCATGCT

GAATGCATCAAGTGTTCAACGGGATCTGAGGTACCGTCATATCATTCAATGTTCAGGGGT

TTCCAGGTGATGGGTAGAGGGGTATCCATGATGCCATCCATAAAAGGGTGCCACTGAACT

GCATCATTATGGATGTTGAGTGATCTAAGTCGGGAGTGGATTGAAGGTTGCTTGTGGTGA

TTGCATGAGTAAAGTTGAGTGTGACTTTCCTAGGTATGTGTCTCGGCATAGGTGGGTGGT

AGGGACAGGGACCGAGGTAGAGATAGTGGTGGTGGGGGTGGCGGAGGTGAGTGTTGGATT

GTAAGTTGAGCTATAAGGTTTGCGTTCTCCTCCCTTAATTGTCACATGTCCACCTCATGT

TGCCTTAAGGCCTCCAAATCGACCTTCGTTTTCTTCTATAGCTGTTCCATTTGTTCTCGA

ATTTCTCGTATCGTTGATGTCTTGGCTCGTGTGGAGTGCGCAAACGAATTGGGATCGCCT

CTCCTTGTTGCTATCATCTTGTGGCTGTAGGAAAAATTATCCCCGGTCCCAAGGCGGGCG

CCAAATGTTCCTACTGAGTTCCAACGAGGTCCGACCAATCTGCTTGGTCCTCTTCTTTTG

AGTGGAATCTTCGATCTCCGGCGATGAAATGGGGGTAGGTACTTACAAAAGGGACTCCGA

CAAGCAAGTCAGTGGTTCTTTGAGGTGTCTATATGTGGGATGAGTATAATGTATGGTTCT

ATAGATACACATTTGACGTTGAGGAGGTTGCATCATACTCAGAGTTTTGTTGTCCAACCG

CTCCTCTATATGTCATGCTGGGTTTGTAAGAGATCATTGGGGTCCATCATGTGTCAAATA

CGTGTTGAGTTCATGATTGCACGATACATTAACTTACAAGAAATGATTGAGACCTATCGC

GTGTCAAGTCTATGAGTGATACAATACAATATTTAATTTAATTTTAATTCAATTATGGTT

ATTGTCATTTTAGATTAGTGTAACTAAAGTAAATATATGAAACATCATATAATAACTGTT

CTCATAGATTGCATTTAATAAAAATATATATAATCATAATTTATTATAATAAATAATTAA

TATACACTAAATTATAAAAATAATATAATAATTTTGAAAATTATTCTAACATAATTTAAT

ATCTTTATATATATATATATAGATTGATGTGAGCACATACTCTCTAAGGTTAGAAGCGTC

ATTTACTGCATTTGCTAGACTCTTGACTCAGCGAGAACCTTCAGCACAAGGGTTGGAGGA

GGACTCAAGAACAACACCACACACCATCTCAATCTTTGCCACGTGTGCATTCTTCCACGT

GTCTTTGTTTCACTGGCTAAACCCTCCTTCACTCTGTCACACCTTCTTCTCCTCCGTAAA

ATACACTCGAAGGCTTCAAGA

>Glyma13g19330 chromosome:V1.0:13:12782190:12784190:1

CATGGCCACTTGCAGTACTTGCATACTCGTTTGAGTCGGAACCTGGTCGAACCATCGGCT

CTGATACCAGACCTGACATCACACTCTAACCCAAAACCTTAAGGTTCAAGTTTATGGGTT

TTCTTCTCACTTATATGGTGTTCAATCTTTCTACTTCTACCTAATGTCGGACTTCACCTC

ACATTTGTAACCTAACACAAGACAATGTAAAATATAATTAATATATGATATATTATATAT

AAAAAATTGATTTTCATATTTTTTTAAAGATCGTATGATTTGTTCTATGATTTACTACAC

TGATTTTATTTTATCATTTTTCTTTTTCACCTGATAAGTAAATACAATTTGGAATAATGT

GATGAAACTTGGGACTAACAAAGCCTAAATCCTATTTTAAGGTTTGGACATTGGAGAGTT

CTACACTTAATCATGTAGTTATTAAGTGATAAGTTATCAAAAAGTAAATATATATTGTTG

ATATAGGTAATGCTAATGAATTCTTTTAAATTATTTTCAACCGTACAATATTATATTTGC

ATTTTCTAGATCATTTTCATTCTGGTGTAATTCGATTGGGGTATTAAATGTGTAACATTT

TTCATTACAGTAACAATCCATAGTAAACTATACTAAAAAGAATATGAAAACCCAAAATTA

TAATAGTTTGAAAATATCTCATGTATAATCAAATAAATGAGTCTTTACATAAAAAAAATA

TTATCATCAAAATATAGGCATCTAAACTAAATGAATGCTAAAAATATAACGAAAGTAATC

ATATTTGGCATCAAGTACTAACCTAACTAAATAAATGTAACTATGGAAAACTATGCCTTC

AAGTCTGCTCGATTTCGATAGCTTCTTCCTCGTTCTCTGAAAACAACACTTATAATGAGA

TGATAATCTCAGTTAATAAACTAATTTCTAAAAGGAGTTCGTAAATAGTGTTGTTATCAG

TTGGCATGGTAATTCGAAAAACCAACTATGGTGGTATCCACAGTAAATTTAAAACAAGAT

AATATATATATATATATATATATATATATATATATATATATATATATATATATATATTAT

GAAGGTTCAATTAATCTAATTAGAGGTAATTCAATCGAATTCATCCTTAATTACATTGAG

ATCACAAATCAGATTGAAGTATCATCCAATTAATCTGTAATTAGGATTCAAATTAGAAAA

TAAATCAATTACCTAATTCATTTTCAATCCTAAATGTCTTTAGGATTGGAGAAGAAGATG

AACATTTACAACATAATTCGAAATACTTAACCAAAACTCAAATCCATCATTGCTTGAACT

GGATTCTTGGATTGATTAGTTGTTTAGTCTTCTATAGTCATCATCAATGGAAGATTGAAC

AAGAGAAGAAATGAACAGAGTTGAAGAGATGAAGAAATATGAAGAACAAGTGAGAGAAAA

AGAGAGAGAAGAGTTTGATCAAAACTTGCATTAGAAGTTTCTAAAAGTGTGTTTTGTGCC

AAATGATTCTAACTAACTCTGAAAATACACTAAGTAAACTAATATATACTCTAACTAGTC

AGAAGTAATGGATGGCCTCTGATTAGGTCCAACTAATCTCTCTAATAAGTTAATTACATA

ATGCAAAAGCCCAAAATTTGCAGCCCAAAATCCAAGTACAGAGGCTCTGATTTTCAAACC

CAAAATGACCCTCAAAACAGAAAAATTGGCCCTCTCTGACAAATTTGTAGCTTTTTCCCT

TGGCTTTCTAGGGACTACTCATATTCTCCATTTTGAGTTATGTAGCATCCTATAGGTTTT

GCATAAGATAGATAGGTAAATTAAGCACAAAGTCTGAAAATAAGCAACAATTACCAATTA

AGTCTAATTATTTTCCTAAGACCAAAACTGAGCTAATGCGAGAAAATAAGAGTCAAAGAA

AAGTCAAATAAGCTCAGAAGAATAGAAAAATAATAAACTAAAAATGCTTTATCAAATTCC

CTCACATTTATCTTTTGCACT

>Glyma13g19331 chromosome:V1.0:13:22887383:22889383:1

TAATGCTTTCGTTTGTTGTGTCAGCGAGTGAGAGAACGAAGAGAGAGATCTGAGAGAAAG

GTTCGAAACCGCGGCTGGGTTTCGAGATCGAGTGCAGAGAGGGCGAGGGAAGGAACGAGA

GTGGACCTTATATAGCAATTTCAGCAATGCTTTGCGCTTGTTAGGGTTTTTGTGGTTCTT

TTTTTCCATAATGTTCCAAGACCTTAGGGGCCGTTGGATGTTGAGGCTAGCATGTTTGGA

CAGTTAAGATTCGAATTTTTCCTGGCGGGCTATACTTGGTCGGGGGCAAAATTGTAAAAA

TCATTCTATTGGTCTTAGAATTCAATTTCAATCCCCGACCCCAAAATTGAACATTGATTG

TCATTAGTTGACAGTCAATTATCGTGCTTTTATTGGACGTTGATTTTCTACACCTAATTA

AGGTCAACATCTTATAAAAGTGCGACACATGACAATTAAGGTTCATTGAAAGTGCGACAT

GTGGAGGTAAACAATTTACCGTGAACATCCAATTTCGGGATTGAGGACTAAAATAACAAG

ATGACAACAAAATGAGAAACAAAATTCGTAAATTAAATTATAGGAAGACCAAAATTAAAT

TCTAAAATAAATAAAAAGACCAAATTTACAATTTTACCTTTTTTTTGTCCAACACTTCCT

TCCTCACTCTTTAATGCATCATACATTTAGAGTTGCCAAATTCTTAAATTGACAAAAAAA

CATGCGTTTGCAAACCAATCATGTTGCCCATACCAACAATGATGACCTCAAACAATTTTC

TGATTGGCTAGTAGATATAGGTGATGACAAACTTGGAGAACCTAATGATGGATGTGGCGA

AATCACCTTCCCAAATGAGTTTCTTATCAAGGACTTTAATGATCCTATCCAAGCAATTGT

TGAGGCAACATATCTAGACTTATTACAAAATTATAGCAACAGAGATTTCTTGCAAAAAAA

AGTTATTCTAGCCTCTACAAAAAATGTTGTTGACAATATAAATGACTATGTCCTATATTT

GATATCCAATGAGGAGAAAGAGTATTGTAGTGCCGATAGTGTTGATAAATCTGATGAACT

ACTCAATCCTACATTTGGAGTTCTAACACCTGAATTTCTGAACTCGTTGAAAACATCAGA

AATACCTAATCACAAGTTAAAAATCAAGGTTGACACTCCCATCATACTATTACGAAATTT

GGACAAGACAAATGGGTTATGCAATGAAACTAGGCTTATTGTCACAAGACTTGGTTCAAA

TGTGATTGAAGCAGAGATTATTACTAGACCCAATATAGGTCATAAGACATACATACCAAT

AATGAATATGTCTCATTCTGATTCTCCATGGCCATTCAAACTAATTAGAAGACAATTTTC

ATTCATGGTTTCATTTGCAATAACTATAAACAAGTCTCAGCGACTGTCCTTGGCACATGT

AGGATTGTATTTACCAAACCCAGTATTTTTCCATTGCCAACTATATGTTGTACTTTCACG

AGTGCAAAGTAAAAAAGGACTACATATTATTATTCATGACAAACAAGACACTCCAAAAAA

TACTACCATTAATGTAGTATACAAAGAAGTATTTGCAAACTTATAAAGAATGTATGCTTC

CCAAATTCTCATTGTTACATATGCATTATGGTTAATCAAATTCATTTTCTATCAAACTAT

ACAGTAACACATCCTACATTTCCTACCTACAACATTATTTATTGACTTGATTTTCAACAT

ATCATTTTCTAATTAGAGTATTACAATACACTACATATTTAGCTTTACCAGTACTTATAC

AGGTTTCAACATGCACATTCTTTTGCAATTTGATTATGATCAAATCTCAAATGCATTTTG

TATATTAGCTTTATTTCTTTATGTTGCAGGGGAAAGAAGGGAAATACTGCCAAATTTGTT

ATTAGGTCACAACCAATAAAGCATATTCACATCAATCTACCCCTTTTTAGGTTAGTATTG

TGTATAATAGCACTAACCTAC

>Glyma13g29580 chromosome:V1.0:13:32471031:32473031:1

GTAATTTCCCATAATTTGATGCTCAACATTTTACATACTTTGGACCCAAAAAGTAACCAC

AAGGACGTCCCTACCTACTAAGTAAAAAATCCAATTCAACCTGGCATTCAACATTTCGAA

AAACCATCAACCTAGGCACAAAGCGGGAAGGATGGACCATTCATAAAACGATGATATATA

TATATCATCCTTAAGCATGTAGATTATTCTATCCGTGAAATTTGAGTACATTTTGCTATT

CCTAAATATGTTGATTGACTTATTGATGAAAAATTATCTCTTAAATTACAATCAGTGTTA

TTAAATATAACAACTACTTAATTTGTTTGAGTAACAACTACTTTTTTTTTTAAGGAAACA

ACTACTTAAATAATCTAATTTTATGATTATTAAAATAAATTTAAATTTATGATATTATTA

ATTAACATTAGTTAATTTAATTATTTATATTGATCTTGATAAATTTGTGGCATTATACAG

TTGTAGTATATTTCAAATTAAATTAAATTATTTATTTAATTATTAACTTTAATATGATTT

TTTCCCCACTTATAACCTAATATGGCTAAAGTACCCAATCAAATGAACCCCTTGGCCAAG

AAGGAGTCGTATGGGCCAGGAGGGCAGAACTAATAATGGTGGTACCATGGTAGTGCGCAA

GGGAATATGCATTTCCCTTTCCAACATCTTATCTATTTACCAGTGAAGTGAATCTTTTTT

AGTGACGTATTACTATAAACTTAAGACTTTATTAATATTGCTCGTATAACTTTTTGTATT

TTCTTGTAATATTATATTATCTATTATATTTATGTTTTTGTCTTTTTCTTTTTGCAATCA

CTCTAATCAAGACCTTTACCTTTTCAGTGAAATTTTCTATTCTCTTGAAGAAGGATAGGC

TAGGTTAATTTGTTTTTAGGTTTCCATAGAATTTTTTTTATAAAAAAATCATGATTAGAA

TTGTCGATCTCTTTTCGCAAGGAAGTCTCTATTCACCCTTATAATTTATTAAACGAAATT

ACAAAAGATAAATTTATTATTTAATTAATTAAATCAAACGACTAGATTATTGTAATTCTT

TTCACAATTGAAAATGATCTTATTTTTGGTCAATAAGAAAAAGATGATCTTAATTTCGAA

ACGAAATAGGATTTCATGTAGTTTCTCTCTCATTCTAACACTTTATAAAAATTACAATAA

ATAATATTTTTATATTTTAACCTTATTTTAATTTATTTATTTATAAAAAATTATCACAAT

TTGGCCTTTTTTTTAACTTGATCCTTTTTACTAATTTATCAATTTTAACATTATTTAAAA

CTATCGTAATTAATATAAAAAACTCACAATTGACAAAAAATATTATAATATCTGATAATT

AAATATTTATACTATTTCATTTCATTTTATAATTAAATTTATTAACATTATACTTAGTTT

ATAAATAAATTAATTTTATAATTAATTATTCTGACGATAAAGAAAAAAAGGTACTCGTAA

GAAATAGTAATATGAAAAAAGTAAATCTATATTTATAGTAACCCTCTCTAATTTCTTTTT

TAAATTGAGAATGTAACAATTTTTTTTTTTTTTTGTGTGAAAGGAGGAAAAAGAATGTAA

CAATTCACTATGTATTGAAATCACAAATTCCAAAATTTAGAAATTTGGAAAAATATGTCT

CTTCAATGAGCCAGTAACAATTAACAAGAAGTCTAGATAACTTTCTTCCCACTTTACTAT

GGATTAATCAAGGACCTCCCTTCCTCGTAAATTACCAAATCAACGTCTCCAATTTTCTCC

ATGGTTCTTTCCACTCGCATTCCCCTCAAGTACGGGAAAAAGTGTTTCTCCTACGCCCTG

TTTCTTTTCCACGCCTTTAAATAGACACAGAGTGAGTGTCTCACTTCTCATTCATCAACC

ACATACACATTTGCACTACCCACTTTTCCTCTGTTTATTCTACTCCCCTTTTTTTCTCTC

AAGTTCTAAGTTGTTCTCCCA

>Glyma13g29590 chromosome:V1.0:12:32103319:32105319:1

AAGTATGAATTGAATATTCAACAGGGTGGGGAGAAGATGAAATGAATAGGTACTACTACC

ACCTTCCTCAACCTGCCACACTTCCCACTACAACTACTACTAGGTTCTATTAGTTTTTGT

GATTTTCTTTATTCATTTCACATTTTAGTTAGACATTTTCACTTTGTTCAAATATTTTGT

TGTTGTTATAATCGTGACCAAAGGGGTATCATTTATGCCACGGTCATATCATTTTAATTA

CAAATTTACAACAAAATTATTTGCAAAAATGTAAAGTTTTAGAGATGTGGTTAACTTCCA

CTTCAATTTTGATTTTTTTTCTTTCAATGCATTGGTTTTTCTTTTTCTTATTTTAAACAA

ATTTCAATGCCTTCTTTATTGGTTTTCACTTTTTATTACTCTCTTTTACCAAGGCCACAA

GCTGTTATATATTGTTTGTTCTATGTCATATTTTGTTCTGTTATGAAACTATTCTATGTT

TTGTGAATTTAAGTAGTGCTTCAATTCCTTGACTAATTAAAGTCATTGGAATAATGTCTC

AATGTCTTATAAGCGGTGCTGAGAATCTTGAGACTAAGTGTTTTTAAATGATTTTTAGTT

TTTTATTTTATTTTTAAGATACTTATTTAATAATTTCAGGAAGGAAACTACAATGGTATA

ATTCTTAAATTATTCTCGATGAAGGGAAATGGTTTTCTTTTCAACCTTTCATGTGTTGTT

GTTAATTGAAATTAAACTTTATATCAGTTGAATAATATTTACTTGTGTCATCATTGATGC

AATTGAGTGCCCATCTAACTGCATTAGCTAATTAGTCTTCAAATAGGTTTAATACTATTA

TGGTTTAATTAACAAATATATGTTGCCTACTATTGGATATTAGCATAGTGCACATAGACC

AAACTTTTATATTTCATAGTAACCAATTACTATATATTGGCTAGAATATATAGTGAGAAA

AGTCTTATTTCTTATCATCAAATCCTTCTAGCATCGATCCTGGATGAAAATATTGTACTT

GATGACAAGGAAATAGTTGTGTAAGCAATTTCTGTGGCAAGGTTACAAAAGGTGGAATTA

CTAGAGCAAAAGAACAATTGATAGCAAACTCGGGGAATGTTGCTCCCAAAGAAGTTAGAG

AAGAACTATGGAGATGTTTGAAGGATAAGAAGAAACAAGAAAGGAAAACATTTCATAAGA

TGCGTCAACATCTTCTTGAAGACTATGGTGATAGTGATGAAGAAAGAACTAAAAAGGTCA

TGTGTTCTCACTTTCTCTTCATGGATTCAATTTTTAATACTTTTATCATGAGACACCCCT

TTTTAGATTATCAAATTCATCAAATTGGGATAATTTTTTAAAAAAATTGTGCATTATTTT

TTAGTTTCTAAGTAGGTCATTAAATTTTTTAAAAAATTAATCATATAAATCTATATCTAT

CTATATAAATAGGCTGGTCAAAAACCCAAAATAAAAATCTTATATTGTTTACACGTCCCT

TAAAATAAAGATAAAATGAGAATTTTTAAAAAATAATACTATTAGGTGTAAAGACTGGAT

AGAAGAAAAGAGAACAACAAAAGAAATAGATAAGACGGAGAAAAAATCCAAGTCTTTTTA

TTAACAAAAAGTAGTTTTTTTAAAAAATTATTTTTTTATACTTTTTGGCAAATTTTTCTT

TTTAATTTAAGTATAATTTCAAAATTGAAAAGAAAAAAATATACAATGACCCGTGCTGTA

TGTACGGATAAAAGAAAATAAGAAGTTTGAAATGGAAATGTGTAGATAGGATCGAATGGC

TGGAAATGGGGGTCTCTGAATCGAACGGGAAAGGAGGAGATGGTTCTAGAATATATACAC

GTGGAAGAACTCGCAACATAACATAACCCTCATCCTCTTCAAAAACCCTTCTTCTCTTCT

CTTGGTTTCGTTCTGTGCATCTGCCAAATTAGGATTAGGATTAGGGCTACCAATACCAAT

ACCAATACCAATTCTATTCTA

>Glyma13g29591 chromosome:V1.0:13:32476808:32478808:1

ATAAATTTCCAGTAATTATTCTCTTTCTTTTCATTATTTTTTTATTAGCAAAAATCAACG

ATAATACCAAATAATTTATACATTATTTAACTAATTGAATTAGATTCTATTCATTTTCTC

TTTTCACTATATAATAATAACAAATCATTTTTGTTAGAAAAATAAAATATAAAGAGGTAT

TGACTTACTCTAAGAGTAACTTTAGAGTTATTTTCCTTCTTTATCATTCATTTTTCTTAT

ATCTAGTGATTGTAATAAATAATTAATGTTAATCCTTAGATTTTAACAAAATGAATGATG

GTAATAAAGAAAAGAATTAAAATAAGTGAATCTCTCTTTATAAAAGATAATATAAAGTGA

TCGAGAAATATTTATTCGATAACCTTATATTATATTATATTATGACAAATGTTTGGCAAT

TTCCTTAGGACATTAATTAAGAAAGTTTTTATTGAAAGACAGAAAATTGTGTTTTTCATA

TTTTTTTTTTTCACATTCTTCTATATTATATAAATATTTATCTTTAGTCTTTAACTAATA

TCTACTTTAAGAAGCATTTGATGGGAGATAAGTTTTTCATTAACAAAAATCATAATTGAT

GAGAATCATGAATCCTAAGAATGATATTTCTTGAGAATAAATAAAATATGTTTGATTGAT

TATTGTGAATATTTAGATAAATTTTATAGGAATAAAAGAGTTACGTTATATTTTTACTGG

TTATCTTGATTTAATACGATTAATATGATACTTTTTATTATATTAAAAGAAAAATTAAAT

TGAAAAGGTAAGATTTTCACCTTCCTTTATGGGAAAGTTTGTGTGCTAGTTAAAATATAT

TATAGGAAAATATCATATTTCCCAATAAATGTTATTATTTAAAAATAAATACCAAACATG

AAAAGTTGGGATTCTTAACCTATTTTTTTCCCCTATCAATGAAACACTTCCTTAGTACAC

CCGTTGGCAAAATCATTAGATTATTACTCATTTTTTGTTTAGGAGAGCAAAATAAATAAA

ATAAAATAAAATATTTAATTAATATAGTGTAATAAAGACATGAAATGTATTATTCATTCA

TGGTGTGAAGGATAATATAAAATAAAAAATATTGAACTAGACAAATATTCAATAACTTAT

AATTAACTAAATCTTGTATAACTTTTTATCTGTGTCTAGTAAAAATATAATATTATTTCT

ATTTCTGACTTTTCATTATTTTACACCTTATCCTACACCTTTTCTCTTTTTTTAATATGA

ACCTTATTCTTTAAACTTTATCTCCAAATGTCATACCTACCGCACCTACCACTGTTGCTG

ATGATGAGATTCAAGTAAGTGATACGTTTTTATTTTCCTTCCACAATGTGATTAATGTCC

CGTCACAATGTTACGGCTTTTTAACTTTTTATTTACTTCTTATACCTTTTTTATTTCTTG

TTAAAATAAAAGTATAAAATAATACTTTAAAATATATCACAATATTAATAAATTCTAAAT

ATTAGAAAAATATAATAATAAAATTTATGTTAAATAATAAAATAATTTCAAAATCTGATT

CTATATTTTTTTAATACAAATCAAACATTACACTATATAAAAAATATTATATTAAAATTT

ACATTATTTTATTCTTTAAATAACCACGTCAAATAAAATACAATATAAAATTTTTATACC

TTCTATTATCGAAACGTATAACACAAAAAGTTACTATTCTATCTGCTTTTTCTTTTTTAT

GTCTGTCTTTTTTTTTTTTAACACCCTAAATTCCATCTCTCTCCTTGGGAAACCTCCGAC

TCCCAAAACCAAGTGTTCTCTTTTCCAATATTTTGCAATGCCCCCAATTTTCTCCCCTTT

GCTTTCCAAGCAGTTTCCCCCAAATTCTGAAATCAAAGGGTTTCTGAATTTCTGATAATC

ACACTCTCATACATATATAATAGCAGACCCATCTTACTCATCTCTCACCAATCACACAAC

CAAAGACCCTTTTCTCTCAGT

>Glyma13g32790 chromosome:V1.0:13:34850495:34852495:1

AATGGAATTCTCATGAAGCACAGATTCTCTGGTACAGTAAAATGGAGTTTGTGATCAAGG

CAAGCTTTATTTCTTATTCTTTCATTGTTTTTCCCCCCCTTTAGGAGAGGATATATGGAT

CTATCACTTTCTTTTTGGCAACTCTTATTAGTACATTTTAAACTTGTACAGTATACTTTT

CTTTTTTGGTTAGCGGCAGATTTTTTTAATTTCTTATTTCTTATGTTATTAATTGTCTCT

GATTGATCATGGAGGACCAGATCAAGCTGCTTCGCTGGGACTGTAATTGCTATTCTCCTG

CCCTTTTCTAAGCTTATTAGTTCACTTTCTTGACAATAATTTTTTCTCTCTCTAGAGAAA

CAATCAAACAATTGTTCCTGTATCAATTTTCTTTTCTCATTGGGAAGGTTTTTATTATTT

TTTGTGTTTGTCAATTTTCATGAAATTGTTCAAGTTTACGAAAAATAGTGAAAATTATAT

AATTTAAAAAATGGTGTAACGACTACAGATTAAATTCCTAACACTAAAAAATGGTGCAGT

GAGATTAGAAAGTGTGTGTATCACATAAAAATGGTGATATGCAGTGCAATGGATAATTTT

TTTTTTAATCGATAAAATCTTACACCAGCTGAAACCACGGCAATCCGGTACTTATACAAG

CATATGATATGCTACTATGATGCTTCTTAAGAGCTCCCTCAACCGACCATGGATTCTTAA

GAAAATTTTGGTTTAGCATGATGCTCTTAATTAATTTTAACTTGCAAGGTCATGAGCCAG

ACCTTAGAAATGTTTCAATTTGTAAACTAATTCCTATTATTCACATTAGAGAATAAAAAA

TGCCCATAAACTGACTAAATAGTCAATTTAGTTCCTAAAATTATATTAAGCTATCACATT

AATTCATGAAACTAAAAAAATCTTAAAATAAGTCCTTTAAATTTTTATTTAAGTTCCTAA

ACCAAAATGAAAAAAAAATATGATTAATTTTCGGAACTTAAATGAAATCCTGAATTGTTT

GAGTTTTGTTTGATTTTTTTCTTGATTTTAAAAATTAATGTGACAACAAGATAGAATTTT

AATGATTAAATTGACCATTTACTCTTAATAGTAGTAACTAAATACAAAAAAAAAAAAATG

ATAGCCGCTAGCTGATGTTAGGTTAAAAAAGTATTTTTTAAGAAAAAAATTAAGAATTAG

AATGATAAAATACAAGAAATTCATTTATCGATAGGTATCTTTCTTATGTATCTTAAAGTT

CGCCGATGTATCTTAAAGTTCGCCCATATTTTAAATATGAAAAAAAAATCTCTCCTCTAA

ATTACCTACACGATTTCTTAAATTTTATTAAACCCTACACATTTTTTTATATATATACTG

GGACAAATGAGACACTTCCTATCTCACTGTTTACAAAGCAACTGAGGTTCATGTGATAAT

GACATAGTTAAGTATGATGTTCACATCTTGAGAATGATAACTAATAATATTTTTTTTTTG

TTTTTGATTTTTCTGAATAGGTAGTAATGAAAAAAACAATTGAGGATAGATTGGAAATAC

ATAATAAATGTGTATATTAAATGAGATAATTTTTATTATAATAATTATAGATAGATTATT

ACTAAACCCTACACATTAAGTCCGTACAAAAAGTTTTCTACACATGTATGTATCCATGAT

TTGGTATATTTCATATGCTCATTGTTGATTAAGGCAGTGTTTTATTTCCTACGATAGAAA

TAAAAGGCCTCCAACGGGAATGACAGGGACGGGTTTGATGCAGATGATTCCTGAAAGAAA

TAAGTAATTAACTAGTCCAATCATCTAATTTTTTTATTTTATTCCTACTAAACAACTATA

CAATACTATTTTATTTCTACTGGATTTCTTTTTTTTTTTTCAAATTATTCATTCTCCACT

AAATCAAACACAGCTCAATACCAAGTGTTAATCAACAGAAAAACTTCAAACTACCATTTG

ATTAGTACATTAGATTTTGCC

>Glyma13g43630 chromosome:V1.0:13:43247474:43249474:1

ATTACAATTAACTACATAAATATTAACAAATAATTTATAACTAGTTTATCATAAGATAAT

CTGTAATAAAAAATAATTAATAATTTATAACTAATTTATAGTTACTTATTTTTATATTTC

TCATAATAAAAATTAAAAGGTAATTAAAATATAAATTATAAGAACTAAAAATATTACTTT

CAAATTATATGGACTAAAAGATGATTAAAATATAAAATTTTAAAGACTAAAAATATCATT

TTTAAACTACAAGAACTAAAAGGAAATTAAAATATAAATTATAGAGACTGAAAAAATCAT

TTTAAAAGGAACTAAAAAGTGCAAATGATAAAACTATAGAAATCAAATGAGTAATTTAAC

CTTAAAGTATAAGAGAGTATATGAGATAGATGTTAGAAGTGTGTTTGAATTTTAAAAATA

AAATATTTGAAATTAATTTTTGCATTCAGAGATATAACTAGTTAAAGGGTTCAAATAACA

TTAAAAATATTATATATATATATATATATATATATATATATATATATATAATTTTTTATA

ATCAATTACATTTAAAATATTTGAATTAATATATTGTTATCCGCCGAATGCTAACCTTTA

ATTTGTTCGTTTTTTCATTTGATATTAAAATTATAAACAAAATATACAAATGTGAGAGTG

AAGTTCACTACTTTGTGAGATGAAAGTATTAAAGGAGAGTAAAAATAAAATAAAAAAGTG

GAGAAAATAAAATAATGAGTATCACAAAATTTATTTTTATCATTTAAATTTTTTGTTTAA

TAACTGGGGTTCGTAGTGGTGGAGTGGTGGAGGAACTGGATAGGAAAAAAAATAATTATA

GTTGTAAGATATCTAATAAAATTATTGTTGGAATTGAAATCTCATTTAAAAGAAAAACAT

CAAATATCTGGAGTTCCTTTTTGTATATTATATGGAATATGATCCGTAAGGATTAAGGAT

CATGATTGAAATTTTTTTTATCGTCTTTCTTCAGAAAGAGGCGAAACATAATCAACTTTA

ATTCGGGATAGCTATTCGAAATATTAGTGAAATACTAAATTTATTATTTTATGTTTACTT

TTCTTGAAAAAAAAATTACCAAAAGAAGTGGAGGGTTTTTTCAAATGATAAGGGACTGGA

TCAATTATTCAATCAAATGATATTGAGCATGTTTCTAATTTCTTCTTGAGAAACAAGCCG

ACTATTTCTTTGTAAAATTGTGCTCAATTTCATATGTGGCATAAAAAAGTATGTTAAAAT

CCATTGAATCCTAATCCTAATACGGTCGTAATTCAAAATTGAGAAATGCGTACAAATTAA

TTTTAATTTAATAATAATTATTGCTATAATTTATTTTATACTTGATTTAACTTATTTAAC

AGAGACTAACAAGAAATAAAAAGGGACTGATTTCCAATCTATAGTTAAAGTTTAAGTTAA

ATAAATCAGGGGTAAAATTGGTTTTTCACTGCAGGAAGAAAATCCAAAGATCCACAAGAG

CATGTTTTCCAGCGAAGGCCCGCAAACAAGCCCAACATTCCAGTTTCAGAAACCTAATAA

AAAGCATAAAGCAACGAGAGAGCAAGGGTTCCGAGTATAGATGTTTCTAGAAGCTGGGGG

ATCCTCCAGACATCTCTGGGACCAATCGAGCACCTCTTTTGTTTTCCGTTCAAACTGGTA

ATTTAACCAGAAAAAAATGTTACATAAACAATCATGTTATTTAACATCTAAATATGGAAA

TAAAAAATAAAGAAAATGATATATAAAATTTATGACATGAAAAGATAAACAAAAATAAAT

AAGAATTAGATGAATATTTAAAATAATTAGGTATTAAAGAGGTATTTAAAAATTACAAAG

CTCACAAACACGGTTAAATAAGAACTTTGTTGACTAAAATCGAAAATTTTATGGACAATA

ATTTTCTGCTTTCAATAAAAATGTTTATATTTTTAATTTTTAACTAGTATCATAAAATAA

AATTAAAAAAAAAAACCACAG

>Glyma14g02740 chromosome:V1.0:14:1732679:1734679:1

TAGTAAATATGCAACATTAATTATATTTATGGTTGTTATTTTTAAATAGAGTCTCAACAT

TAGTAATGATATTTCTTATCTTTTTTTCCCATGTTTTCATTACATTTTTTACACTTATTA

ATTATTATTTATGACATCTTTTATCTCGTTGCTATCACATTTTTCATTTTTTTTTTAATC

TTCAATGGTCAATTTTTTTTATACTAATTTTAAGTTTATTTATGTAATACGTGTTTATGT

AAAAAGTTAGTGATAAAGATAAAACACGGGAATCCAATTAAAAAAAATTCTCTAGACAAG

GATGTAAAATTATTTGCTTCTCTCTCATTTGTTATTTGGTGAAAATTATAAATGAAAGTA

ATTTAACACAATTAATGAAAAGAATTTACTTGGACATCTGGCTTCTCCAAACATTAATTT

CCTATCCCATAGTTATAATTTATCGTATCTTGATTTGTATGGGCATATCTTTGTATCAAT

ATTTTCATTTTTACTAATCCACCTTGCTCCTTGTTCTTTTAAATTTCAACATTTTACCAT

CACAAATAACTCTCAATTATTTTTTTTTGTCTATTTGATATATTTGATATTTTATCGTGT

GAGTGACATCATCTTGATTTTCATGGGATCATCCAACAAATCATCAAAGGGATTAAACTT

TTTTTTTATACTTTAATAATTTAAATATCTATCTTTTAAAAAATAAATATTTAATAAATA

ATTTTTATAAAAGTATTTATTAGTTTTACATGTTAAAGTAAAATTGATTTTTATTTCAAA

TGATACAAGAATTAAACATGTGTCATATACTCTCTATTATAAAAAAATGAAATATAAAGA

AAATAGTGTGATAATCATAATATTTGAATTTATTGGGGGTTGAAAAAACAAAGTGAAATA

GTCTTCATAACGATATCTGGAGTTTAGTTTAGTTATATTAGACAAGTAACTCTTTAAATA

TGGTGTAAAAAGATGATACAGAACCAAAGATCTTTTCATAAGTTTTTTTATTCTATTATA

CTGAATAGAATTACATAAATTATAAAAAAAAAATAGTTATTTATATGACTTTAATTTAAA

GTATTCTGCATGTTTAATCTCAACATCATGATTTATTAATCTTCCATCAATCTCTCCCCG

ATCACATCTACTACAAAAGATACTTAATCACTCTATGCTTGCAAAAATGATCTAACATTC

ACTTTATTTTTTAATCAAAATCAGAGATGATAACACACAATAAAGTTTACTTAAAAAAAA

AAAAACTCAATGGCTAGTTACTAGTGACAGTTGGATCCTTGGGCTGGTGTGACCAATAGT

TTTCATGGAATACAATGACTAATGAGTGTTCTCTTTACAAGTCTTCCATATTCAAAATCA

CTCCTAGGATACAAATTAATTAATAATTTCTTCTTCTTCAGAGTGTGGATCAATAATCTT

GTTGCCTTGATACATGGTTATTTGAGATAACCATTTTAAATAAAACTTTTTAATTGTCCA

TAGACTATTTTTTTAATATATTCTTTTAATACAATTATTTTTCATAAAAGATGTATATTT

ATAATAGGGTAAATTCATTGTTATCTATTTTAAATGTTTAGCATGTAATTTTTTGAATAA

ACATCTTATTTAGAATTTATCTATGCAGTCCTTTATTGTATTTTTGTGGCATAAAAAAAA

TAAAAGTTTAATTCTCACCTATGGTATCATGAACATAAGTTAGTTTTGATTAAACACATA

CAGAAAAATATAGATCTGACTTGTCTTGTTACTTAGTCATATAAATATGCAAGATTGATT

ATAGTATAGTAGTTATTACTTTGTTGTTAATATTAACAATGGTGTTCAATACACATTTTA

CTTATATTATAAATAATATATTAAAATATAGTTAATTGAGGAAGTATCTACCATATTTTA

AATTATATAAAATTCTATTTACTTAATTTTGTAATGACACGATTTTTTTTTAACCTTATT

TGACTAGTTGGACCTTGATTA

>Glyma15g01750 chromosome:V1.0:15:1159313:1161313:1

GGGCCTTCGTCGTAAAACAAGGTCTTGTCCGTTTTCATTTCTTTTGTAATATCTATTAGG

ATATTCTTCCTTCTCCACTCAATCATTAAAGTGAAATGATGAAATTATTCCTTACTTCAA

ATTCAAATACATTCATCTATTTCCTCCGCACACAACATTCACTTGATCCTTCCTACTATG

CTGCCACTAGTCTCTTCCCTTTGTTAATCTTTATTATTATATAGATCATCTTCTTGGTGG

TGTTGTCTCCAATTAGCATTTGTCGCTGTGAATATCCTATCTTCCCATTGAAAAAAATAA

GTTCTTTTAAGGAATGTAATAAAATTACATATATATATATATATAAGTGTATTTATGTTC

TCATTGATTTACTCTTTATTTTTTTTCTTCTTATTTTCACTCTCATTTAAATACTCTCGT

CTCATGAACTAATGTATTCCACTCTCTTAACACATATCGGATTTTTTTTTTATATATTTC

TAATATCAATCGAACAGTTGAAACATATTATCAAAAATTAATTTTAAATATATAGAAGAT

ATATAAATTTGATCTTCAAAAATCAAATACGTTCTTATAATGTATATATATGATATATTT

CTTTTATGCTTTTGATGTTAATTGAATCCTTTGCCAAGCTACAATTGGACATCATAATTA

ATTTTAAATATTTTATCTTCAAAATTTAAATACGCTTCAAACCTGTATCTCATATATTTT

CTCTTGTACTTTTTAAATATCAGCTGAATTATTTATGTTAGCATTTATGGTTTTCAATCT

ATCATTTTTAATATTTTTAGTTTTATCTTACAAAAAAATATCTTTAATTCTTACTTAAAT

TTCAATATTTTTTTTAAAATGATCAATAATTAAAAATAATAATATATCATAATATAAATT

ATTTATCATAAATTTAAAAGTACAAATATATTCATTAATTACATATTTTAATTAAATATA

ATTTATATATTTAAAATTTATTTTTATAAAATAAAAATTTAGCATGTGCAATGCATGGAC

TAAAATGATAGTTTCTATGCAATCTCTATTAAATTAAATCATAAAACAATTTGGACCAAT

AAATATTACTACATTAAATTAATTTGTAAGTATTTGCCCATTTTTAATTAGAGGTGTATT

CAATTTTTTTTCTGCACGGCGTATTCAATTAGGATTCTAAAAATTTTAATTTTTTTTTAA

ATATTCAATCAAGATTTTTAATAAAAAATAAATCTTATGATATTTAATAAGATAAATATT

TTTTAAGGAATATAATAAACTTCATCGGTATTTAATTGAGATTTTTTATAATATATAAAA

ACTATTATGATATTAAAAATTAAAAAATATATAAATTTGAATGAATTTTATTACTTCAGG

AGGTAACCATTTTTAATCTTTTTTCTTTGGTTTCCCGCAGGTATAATTTTTTTGGACTAA

ATAGTATCAGCGAGGAATCAAATGTCGTTGTGCGAGAGTACTAGTCCCTTCCGCTACCCC

CAACCGCTTTGGTAGCAATTTAGAATCTATTTCAGTTGTTGTATTTTCTTTTCAATTACC

GGAGTATGATTTGGAGTTGTTTCAAATTTCAATTGCCAATTAGTGAGAGTCGCGGAAGAG

ACAGCTCTTGAGTTTTTGACATTGCAATTATAAACTTGCCTTTGGATCGTTCAATTGGAA

GTGATACCGCAAGGTTAATGTTATTACTTGTTACTGGGTAATTTGATAATGATGTGGTTC

TGTCATAGATTTGTATCTCTTCATTAGATGCACGGTTTACCATCAAATCGAATGCTATAG

TAGATTATAGCTTCTTGTCGAGGAAGCACAGCATACGGGGTATGGATATGATACAACAAA

TTTAAAAATTTATAGGACACGAGAGAATTCATGTTTAAAAATGAGTATAAATCATAAATC

ATTATAAACATCAAGATTAAAAAAAATTATCTAATTATAAAAATGTTTCAAGTGTCTAGG

TAATATTTGACATGGATATGT

>Glyma15g06530 chromosome:V1.0:15:4618773:4620773:1

GTTGTACTAATCAAATGAACCCATCTAGTGGTACTTTTAAATTTTCTGTGCATTAACAGT

TTACACTTAAATTAATCAACAATGAACATCTGAATTTACCAAATCATGGATATATACATT

TGTAGAAAACTTTTCGTATGGAATTAATGTGTAGGGTTTAGTAATAATATATATAAAAAA

ATGCGTAGGGTTTAGTAAAATTTAAGAAATCGTGTAGGTAATTTAGAGGAGATATTTTTT

CATATTTAAAATATGATATAGGTGAACTTTAAGGTACATAAAGAAGGGTACATATAGATA

AATGAATTATCATTCTGATTCTAGTTTTCATATTTATTTTTTTCTTAAAAAATAATACTT

TTTCTAACCTAACATCAGCTGGCAGCTATCATTTTTTAATTGGTTTTTGCATTTAATTAC

TATTATTAAGAGTAAATGACCAATTTAGTCACTAAAATTGTATCTTGTCACATTAGTTTT

TAAAATCAAGGAAAATTCAAATAAATCTCTAAAAAAATTCAGGATTCATTTTAGTTCCGA

AAATTAATCTTATTTTTTTCATTTTGGTTTCTATACTAAGGACTTGAGTGATTATAACAA

CATGTATTTAGAAATTAAAAAAAAAAGTTTACTTTGGGGACTTGAATGAAACCTAAAAAT

TTGAAGGACTTATTTGAGTTTTTTTTAGTTTCGTTAATTAATGTGATAGCTTAATACAAT

TTTAGGGACTGACCATTTACTCAGTTGACTAATAACATTTTTCATTCTCTAATGTGAATA

ATAGGAATTAGTTTACAAATTGAAACATTTCTAGGTCCGACTCTTGACCTTACAAGTTAA

AATTCGTTAAGAGCATCATGCTAAACCAAAATTTTCATATTAAGCACCTTGTTTTATTAA

GAATCCATGGTCGGTTGAGGGAGCTCTTAAGAAGCGTCATAATATCATATCATATGCTTG

TATTTTTATTTTTTTGACAGATCATATGCTTGTATAAGTACCGGATTCTTATTCCTTACG

TATTGACCATGGTTTCAGCTGGTGAAAGATTTTATTAAGAAAAAAAAAACTATCCACTGC

ATTTCAGATCACCATGGTTTCCCTTTGTCAGTCTCACTTAAGATTAAGCTTTTATTTGGG

TTATTATTGATAAAATAAAATACGAGTTAGAAGACAAAATTGACATTTAATCTTTTTTTA

TTCAAATTTAAGTTATTCTATTTTCATTTTAAAAAATATTTATACAATACACAAAGTATT

TGTTAGTTAAATTCAACAGACAAATACAAAAAAAAGAATAAAAACCTTCCCAATGAGAAA

AGAAAATTGATACAGGAACAATTGTTTGATTGTTTATCAAGAGAAAAAAAAATTATTGTC

AAGAAAGTGAACTAATAAGTAATAAGCTTAGAAAAGGGCAGGAGAATAGTAATTACAGTC

CCAGTGAAGCAACTTGATTTGGTCTCCATGATCTATCAGTGACAATAAATAATATAAGAA

ATCAAAAAATCTGCCGCTAACAAAAAAAGACAAATACACTGTACAAGTTTGAAATGTACC

AATAGTTGCCAAAAAGAAAGTGATAGATCCATATATTCTCTCCTATAGGGGGGAAAAACA

ATGAAAGAATAAGCAATAAAGCTTGCCTTGATCACAAACTCCATTTTACTGTACCAGAGA

ATCTGTGCTTCATGAGAATTCCATTTAGGCTTCAGATGATTGTTTTACTGACCACTAATT

GCCAGTTTATTACGTTTCTTATGTGCGTGCGAGCCATTGAAAAGCAGCTGATAAAATAGC

TTGCAAGTGAGGCATTCTTCAAAACAGAAGGAAGTAATAATTGACTTAAATGTTCGTTGA

TAGAATGAAATAGGTAATGCCATGAACCATCAAATCCGAAGATAGCCCCCTCGCTCCTGC

TCTCCTATTCTTAATGATCTCCTGAGCAATGACTTCCTGTTCAGTGACCAGCTTCTTCCC

ATAGCTCTCTTATTTCGCCAG

>Glyma15g09420 chromosome:V1.0:15:6735395:6737395:1

TTGGAAGAGTTTAACAGAGAAGAAGAAAAGAGACGAGAGTTAGTTACCCACCGGAGAACA

GAGAGAAAAGGGTCTTTGATTGTGGAAGAATGGATGTGATTGATGAGTGACGGGTAAGAT

GGATCTATGTCTATCTATCTATAGGCATAAGAGTGTGATTATCAGAATTCAGAAACCCTT

GATTTCATAATTTGTTGGGAAACTGATTGGGAAGGGAAGCAACATGGAGAAAATTAGGGG

CAATGTTGAAAAAGATTGGAAAAGAAAAGAGCAAAGAGAGAGACGCGCTTTAGTCATAAC

GAAGAGAGGGGATGGCTAATACAGGGTTATCTAACAAATATGTAGTATTTCTTAATCAAT

TCTCTCATATATTAGTAGAATTCATATGATAATCCATCCATCCATATAAAATCTACTATA

AATAAGTACTTTTGTCCATGTAATTTGTACTTGATTACAAATTCATTCTTGAACTTTGTA

AATTATTAATTTAGTTTACAAAAGTTTAAATATAAAGATAATTTCATTAAGTTGTTAAAT

CATTTTCATCTTTGTTAGGTTTACTTGTCTTAGGTGATGTGGCATTGATGTGACACATTT

ATGTGTATGAAAGTGAGCAACATACATTAGATCAAATATTTGTGAATCAATTTGTCAATT

TTTTTTTTATTTTTACATCTAAAAATCACCACACAACCATAAAAAGCGATTATTCGGATT

TTTCTTCATTTTACCATTGTGAGAGAACTTGAACCCAATGTTCTTGATTGTTTTCCTTCT

CCGTCATTTGCATTATTGGTGGCTAACAATGTCGGTTGGTGACATTTTATTTGTGGTCGT

CGTTTGAATATGATATTTATCTTTGAGTTGAAGATGAGAGGTCATCTCAAAATATAAGGT

TGCATTTTGTTACTTCGTAGCACCACAAATGCATGAATGAGAATGCTTTGTTTTAACCTT

TATTTCCCAACCACAATTTGAAATTTATATTGTGATAGTGTTATGTTCCAAGTGCAAGGG

AAATCATGGATGTTAATCCATGATTTGGTATCTTTTTTTTTTTGTTTTGGGTATATTCTC

ATGTTACAAATTTTACTACTTTTTAGTTATGTTATGAACATAATTTTAAGCTTAATCATA

GTGTTATTTTCAAAATTTGGTGTGTATCCTGCTATGAATCCTTTTTTACAAGAATTCCTT

CTTATCTACTTTTAGATTTGGTCTGAAACATCTTCCTCATCACAATTTGGTTATGTTATG

AACAAAATTTTGAGTATAACGATGAATGCATGTTTTAAATTCATACGAGTGTTCTGGATC

ATTTTGTTATATCTACTAGGGTATATCTACTAAGGACCAAAATGTCATAGAGTAACAACA

TATTTCCACAAAATCAAAAAGAAAAAGTACATCCTCTCAAAATAACATTAGGTTTGTAAA

ATAAAAAATAACATTTTAAAAAAGTTTCATTAACAATTTAATCTAAAAATTGACATAAAA

AAATATCATTGAACATATGGACTTGTCATGTTACCCTTCACATACTACGAATATACTCAA

TGTTAATGAAATCAGAGATCACTTAACGATTGAACTAAATTGTTAGTATATTTATACTTT

TGTAGATTAAATTGAATATTTTCATAGTTTAAGGATGAAATTGTCACTGGATATAAATCT

TGGAGATAAAAATGACTATTTATTCTAAAATGTAATTTAGTTTATCTGGGAATTTTTTTT

TTAAAATCTCTAATTTTTATTTCTTCAATTAAAAAATTAATATTTCTTTCTTATTTTTCT

CACTTTTTATACTTAAAAAAATAATGTGTAATTACAAATTAAAATATTTCATCAGGAGAG

TCTGAGCTAGAGTCAACTACAAAACAAGTCTATCAATAATACTGAAAAAAGCGGGTTAGG

TAGCATAGGTTCCTTTTCCCTTTGGCTACTTCTTTGCCAAGGAGTTGATATATTCAGTGA

TTGGATGTATAACAACAAATA

>Glyma15g09430 chromosome:V1.0:15:6737540:6739540:1

GATTCAATTACCTTTTAATTATAAACCCAATCGGTTAAATTATTTGAACCATCGGTTATA

AATCTCACTGATTCAATTTCTAAAGATGACGGTAGACATTAAAAGTTGAATTTTTTAAAA

ATTTAGGCATTGATTTAGTTTCTTTACATTTTAAAACAACATCAATTTAATCATTAATTA

TCTTACCAATTTGTGAGTATTTTTGCTGGTATCCCCGGGTACAATTTGTCATTCCTAAAC

GCATTCACTCATTATTGATGAAAAATTATATCTTAAATTACTGTTAGAGTTTAATGTTTA

TAGATAAATAGTATAAAATAGATTTACAATGTACAACTTTTTACATTATTATTGTATAAC

ACTTTTTCTTTTAATTTATTATATTAAATGTCACTTTTGATCTTTAATATTTTAGTATTT

TTTGTTTTTATACATTAAATTTTAATTTTTTTTTATTTCAATCATTTCTTTAATTTTTCA

TTTAAAATAATAGAATTTTATTAACTTTATTTTTAGTTTTGAAATAATTAATTTAAAATA

ATAATGTCTAAAACCATCACTGTGTTTTCTAGAAACAAATTATGCGAGAAATATATTTGT

GTTTTTGGTTGACTTGCGTTTGTTTCTGTGATAAATTATAAAATTTGTTTTTTAAAGATA

CATAATTTCAATTTTTTAGTTATTATTATTTTAAATTAATTATTTTAAAATTTAAATTTA

AGTGAAACACTAAATTTAATGAAAAATAAAAGAAAAAACAAAAATAAAATTCTGAAAAAC

ATAAAAATGTAAAATAAATTTTTTAAAATTTAAGGTATCAAAATAAAAAAAATATTAAAA

TATAAAGGATCAAAATTAATATTTAGCTATATTATTAAATATAGCATATACTTAAATAAT

ATAATTTTATGGTTATTAAAATAAAATTTAAATTTATAATATTATTAATTAAATTAACTA

ATTTAATTATTTTTTATTAATTTTGATAAATTTGTCACATTGTACAGTTGTAGTATATTT

TAAATTAAATTAAATTATTATTTAATTTTTTAGGTAAGTTTATTTATTTAATTATTAACT

TTAGTATAATTCTCCCCCATGTATAATTTGGCTAAAGTACCCAATCAAATGAGCCCCTTG

GCGGAGAAGGAGTCAAATGGGCCATGGGGGCGGTACTAATAATGGTACTATGGTAGTGCG

CAAGGGAATATTCATTTCCTTTCCAACACCCAGTAAAGTGAATCTCTGTGAATTGTGAGT

CACCTATTACTTATTAATATTGCTCGTATAACTTTCTGTATTTCTCTTTTTTACAATATT

AGTATCATCTATTATATTTATATTTTTTTTGTTATTATCACTCTAATTAAAACTTTTATG

TACCACAAAAAAATTAAGATTTTTACCTTTTCAGTAAAATTTTCTATTCTCTTGAAGGAT

AGGCTAGGTTAATTTGTTTTTAGGTTTCCAAAGAATAAAAAAAATCATGATTAGATATTT

AGATTTGTCGATCTCTTTTTTGCTAGGAAGTCTCTGAATCTCACGCTTATAATTCCTTAA

TCTAAATTTCAAAAGATAAATTTATTATTTAATTAATTAAATCAAACGAGTATAAAAAAA

TAAATGACTAGATTAATGTAGTTCTTTTCACAATTGAAGATGATCTTAATTTTGAAACGA

AATAGGATTTCATGTAGTTCCTCTCTGTTTCTAACACTTTATAAAAATTAAAGTAAATAA

AATAAAGATTAATTAAAGACCTACATTCTTCGTAAATTACCAAATCAAATTAAAGTCCCC

AATTTTCTCCACGGTTCTTTCCACACACCTTCCCCTCAAGTACCAAAAAAAATGTTTCTC

GTACGCTCAGTTTCTTTTCTATGCCTTTAAATAGACATAGAGTGTCTCACTTCACTTCAC

TTCTCATTCATCAACCAAATCCACATTCTCTCTCTGTTTGTTCTACTCCCCTGTCTCTCT

TTCAAGTTCTTAGTTGTTCTA

>Glyma15g10280 chromosome:V1.0:15:7461615:7463615:1

ACTACGTTGTACCAAGATCGATTCCTATGACAAATTTCTGATCTTCTTTGGCCATTCTAT

TTTAAGTGAATGTATAGTTAAATACAGAAAAAGTGAAGCTTGTAAGTATGAAATATAAAA

AATACTAGCTGAAATATATACATGTATGCATATTTTATTTTTTGGTTTAACCAAATGTAT

ATGAGGTTTCTTATTTGGCACACAACTAATCTCCTACCGGAGGCTATTTTGAAGTAAAGT

AGTACCTTCAGCAGTAACTCCATACTATACATGTATGCATCTTGATTTCGTAAAATGAAT

GCACATATATCTGCCTAGCCATCGGTAATCATGACTTAATTATTCCCATTAAAAAGGTCT

CTTCGTGCACAGTGCATCTGTATCTGCAGGATATGGAAAGTGGAAAACTAGAAAACGGAA

AGCCTTTTAGTGATAGTGTGATATACTACTATACGTAAGTTACATTCATTCACCAACAAA

AAAAAACGTAAGTTACATGCATTAGTTTTCCTTCTTTAAGGGATAAAGTGATCTTTGAGG

TAACGATGGCCCAATCAAATGAGGTAACGATTGAGCTAAAATGCAAATGACACAAATGCA

AATAGTAGTAATTGCTTGAGATTTAGGGGATTAGTTTAGCAAGCATAGTGATCATCATTT

TATAAAATTAATAATGATATATTGAGGGACTTTTTAAAATCATAAAACGTTTTAAATTCC

AACAGAAATGGATAGCAAGTCAATTTCATGCCTTGTGATAGAAAAAGAAAAGTCGTAGTA

AATTATATTTTGCTTTGTATATGCTCAAATCACATTTTTTATTTTCTTTTCTTTTAAAAA

TTTAAAATTTAAAATATACTGTGTAATAGTATGCTACACATTCATCATTTTAGAAAAGTC

AAATGAATTATTTGATTTTTTATTATATTTTTTTATTCAATTTGATTATTTATCTTTTAA

AAAGTTTAATTTGATTTTTTATCTTATTTTTTGGTTTAATTTAATTCTTTATCTTTTAAA

AAAATTAGTTATTTATCTTTTTTTAAAGTTTTATCATTCGATATTTAAGATTGACGTCAT

TAATCATTTAAGAATGAACTTTTTCAGTTAAAAATGAAGTGGAAAGGAGAGAAAAAATGA

ACAAAAAAAATTTATTTGTTAACGATGTCAATTTTAAATAGATTAAAAGATAAAAAAAAT

CAAATAAATCATTTAGTCTTTTAGAAATGAATGAGTTATATTCAAATTTTTTAATATAGA

TGAACAAATGAATGAGTTATACTTAAATTTTTTAATATAAAAGAACATTTTTAAGGCAGA

TGAAACTTAGGCCCTTTTCTGAAAACAGTTTATTAACCCTTTTCTGAAAACATTCTTCAC

ATTCACTAAGTACATCTTCATGTCCTGCAGGGAGGATTGGAAATTTTGCAAGAACGCTAG

AGTTCCTTTGTACCGTGAAATCTGGTTAGTTATTTTTTATTCTTCAAACTGCATTTCCTG

ATCTTCCTTTGGTTTTTGAACCTGAATGTATTCATAAAGTCGAGCTCTTAGTCATCCAAA

CATAAACATGACTGACACATCAATTTAGTCATAAAGATGATAGACATATATCAACTTAAT

TATAAGTTGCAGCATATATGAGATCAGATTTGTTGACTCATATCAGCTTGCTACAGGCAG

AAGGGGAATTGATAAGTAAATTTACATACTGTTCCTTGAGGACTGAATCAAATTTGTATT

GTCATGTTCAACTTGTTCCTTAGTTTACATATACGGTGATTGTATATAATCCCCTTATAG

ATTACTTTTGCTGTCTTCGGAGCACTATACTTCCAGAGAATGTATGGTCGGTTAATCATA

TGGTCCATATTCCATACTCCATATGGTTAGATAGGAATCATGTTATGATTTATGGCAAGT

TCAACACACTTGATGAGGTGATGGACATGGTAAAATTAAGATTGTATCTGGTTGAGATCA

AAAGTACCATAATTTCAATTA

>Glyma16g00410 chromosome:V1.0:16:134433:136433:1

AATCATTTAATGAATAATTATATTGCTCACTTTAGTGCTATGAATTATATAATTTATAAT

TTATGCATAAATTTAAAATATAATTTAAGATCAGATTAAATCAAAATTTGACGTTAATGA

AGAGTTAAATTGCTAATGTTAATGTTATTTATTATACAATATTGATAATTATAATTAAGT

TAATACTATATTTTTAAAAAATAAAATTCATGCATAAATTTAAAGAAAAAAAATATTAAT

GTTAATATTATTAAATTTGAAACAAAAACATAAACGTACCCTTTCATTCTTGTTGTCTTG

GCTTACCTTATTGATCGGGCAGAGGCGGCTAAACACCTGCAACTCTGCAAGCTGGAGCCG

GATAACTCTTTCTCTGTCATTGTGAGGAAGCTCATCAATTTTCAATTATCATAATTAAGG

AAATGGAGGTTTGGATGGACAACCTTCATGCAGGACGAGGAAAGCCGTTTCTGATATGAA

GACCAATTGCTACCTGTCTTGTCGTTTTTATTGTGGCTTTCTAATTATATTAGTAGGGAG

ATTAATTAACCACTAGGATATTAAAGGTGTTTTTAGCATCATTTGAGCATTCCATAATAC

TACCAATCATTTTTTTTTAGAGGATCAGCGAGTCAATTATGTCTGCTAGGTTGGTCTTTG

ACAGCTTGTGGTAATTGGGTGGCTACTACTGGTAGATAATATATAACTATTGCTATAAGA

TTTGTGCCTTGCAGAACTACAGTTCAAATGGTTTTAGTTAATGTAAGGTGCAATGCTGGC

TGACATTTGTACGGGTGTAGAAGAAGTTTGAAAATAAAACTACTATTATATGTGATAATT

TAATTTTTTTTCTCTATAGCAGTGCTACATCAGTTAATTCATTATTACTTGGATTCGCGG

CATGTCAAGTAAGGTTTTTCGTTTTGCAAATTAAATCACATGCTCCACCACTTGTGCGAA

CTCCGTCAATTCCTTTGAGTTTCATTCTTGCGAACGTACTCTCCAGGGATACTTAACGCG

TTAGTTACAGCGTTGTATGGGTCGATACGCACAGCGCCCCCAGTATCCATCGTTTACGGC

TAGGCTAGGACTACTGGGGTATCTAATACCATCTCCCCTAACTTTGGTTTATCATAGTCA

GTGTCGGCCTTGGTCCTCCTAATAGGCTATAGAAGCTATTACAAAAATCTTATGAATAAA

GATTAATTATTGTCTTTGGGATTGCATATGTAGGCAACGAGAAATTAATCAAATGGGGTA

TTAAGCCATATATATGTTTGTCGAAAAAGTAAATGATAAAGGAACAAAACTTAGTCATGA

GCAAACAAGTAATTTTTTTTATATTTTTAAAAGTTATTTTTTTATTTTTTTAGTATTATC

ACTCAAAATTTATTTTGATAACGCCAAATGATAAGATCGCTGTCAAATTTTAAGGATAAG

AATAAAGATAAATATATCTAAAGCTATTTTGAATTATTTAAATTTATAATTATTAAAAGA

TTACCATTTGATAAAAATAGAGTCAAGCGAAGTCAATAAAAAATATTGGTTTGTTACGGG

TCCCAAAACTTGGAATGATCAGAGAGGTGATTTAAGAAAGATAAGTTTAGCAAGGTAGAC

CTTGAAGATAAAGAGCAAAAATCGGAAGTAAAAGAGCTCCATTGAAGATAAAATTAGTCG

AAACCTTTATCAACTAGCAAAATTGATAAAGTGGCCGTTAATTAATGGCCTAACTTGAAG

CACCATTTTGGTTAGTTATTTCTTATTATTTTGAATTCATTTATCTTTAGTCACATATTT

TTCAAAATTTTGTTACAATTCACTTTGAAATCAAATGGCTTATAGAGTAACTTGAAAATT

CTTTACCAGATTTTATTATCAATACACAGATTGTTTGATCATATATTTAAATTTTAAAAT

CATGTTGGAGAAAAATTAACTGAAACATATTTAATTAGTATCCAAAAGTGTTGGGATGCG

GCGATAAAGAACGTCTGTGCT

>Glyma17g08020 chromosome:V1.0:17:5930881:5932881:1

GTTGACTTTATTTTCTTAATATAATTTTACTATTATTTAGTAAAGTTTTAGTGCATTTTA

TCAGTGTAAAATTGTGCGCTAAAACTTTTAATTAAAATTTTCATAAAATTTATAATACTA

AAAAAATAAATATAATAATTTAAAAGTATACTAGTTTTGAGAATGGAATTTATAGTTAAA

TTTTACATGAAAAAAATTGTAAAATTTGTATACAGATCATATATTTTTCTTTTTAGTAGA

CATCAACTAAATAAACTTCTATTGATATAAAATTGATCTATGGAGTAAATGATATTTTAA

AAAAGGACAGGTCATACAAACTAACTACTGACATTTTTTTTTAAAAAATCTATAACTAAA

AAAAGATACATATTAAGATTAGGCATAAATCTTGTCCCATTATATGCTATGTTTTCAATT

TTCAATCACTTGTGTTGATGTTTGGGGTAAAAAAATTGATAGTGGAACTGATGTAAGCAT

CTATGTTCCTACAGCATCTAGAAACTTGAATTGAAGAGTTTTGGAAGGAAGCTGAATTGA

GCAGTCCACCTGGAGAGAATAAAGAGTGAAGAGGGGCGGTGAGTGAATGTCGACGACGGT

TTTAAAACACGCGTTGAAAGGAGGAGGATTGCAAAATGAAGGACAGTGACAAGAGTAGAA

CAAGGATTTACGTACGAACTATATGTGAAATTTATCCTCTTGACATCAACAAAATAATGT

TGGTTAAAATACATTTACCATATAGTTACACTTTTTTTTTATTTACCTGTAGTTTAAAAT

ATGTCAATTTAATTTTTATAATTATATTTTTTTAAAAAAATTGTTATAGAATTACCGTTA

GCATTGTTCGTTCGAACATGACTTTTCACGGTGGAATTTTGTAGACGTGACTTTCCATAG

CACATGGGTTATGGAACCGGGTGTGTCAATGACATTGAAAAAGGAATTATTTAGAGAGAA

AAACTTAGAAGATGTGAATTTTCAGGTGTAACCCATGAATGAGTGACATTGATGGAGTTG

TGGTTCTTGTATTTAGTTTGCGACGACAACTATTTTGGGTGGTGGTGGATGGGAATTTTT

GGAAGGTTGGGGTAGACAAAACAATAGGGTGAATAATATGTCGTCAAGGACAGGGGATGG

AAGTTCAATGAACAATAATATGTCATGGTTAAACAAACAACATTAAAGTGGTTTCATGAC

AATGATCATATTGTATATAAAAAAAAGGTAATTATAAGAATCAGATAAACATGATTCAAA

CCATAGGCACCAAATTGGAAATGTAAAGCTTTGGATACAATTCTTTTAGCTAGCGTGTTG

GACGTTATGTATTTTATAAAACGTTGGACGAAATCTACTTTTAAACGCTGTTGGACGCAA

GTTTAATTTAAACAAAATTTGTCATTTAAGTTTATAAGATTTAAGCCTAAAACCACATTG

GATACATGTATTCTCTGCATGTCGACTCATTCAAAAATCATTATTTCTTAAAATTTCAAT

TATCTTCAAAACCATGAGATTTTTTAGGAGCTACACATCTTTTATGTTTTTGTTCTAACA

ATCTTCACATTTGTTCTTTCAATTTTGTCATTGTCTTTAGCGGCCATCGTAACAAAGAAT

TTTCATCATAATGGTTATCCAAACCCATAGAATAATTTCAAATACACCTCACGATAAGCT

CAACTCTAAACAAAAAACAAGGACATTTGGAAGAGTTTTACTGAGCCTCGTGCTGGACAC

CATAATGTTATATTGATAGAGTGAAAATAAATGAAAAGAAAACGAATTGAAATAAAAGTT

TTGAATTAAATTAGAGTGAAAAAGTGTAAATTTTATCTTATTTTACTGTTTATTTTATTT

CTTTTTCATTCTTTTTCCACTCAAAGGAACAGACACTTAGAGATTGTCCAACGAAATTTT

ATGGCTAGTTCATTCAAGTGTGTTAAGACTTTCTTTTTAAATGCTAAAAGAATTTAGACT

TAAGCTATTTGGTGGTTATCC

>Glyma17g11650 chromosome:V1.0:17:8748031:8750031:1

AGAATTAGAAAAAAAAATGTTTGGTTTAGCTTATTTTCAATTTTCAGTAAATAAAAATAT

TAAAAATGTGTTTTTTAGGTAAGAAAATGTGTTTAGTTTGATTATTTTCAACAAAAGTTC

CTGAAATATTTTTTTATACAAAGTTTATAATAAAATTTTGAATAAAAATATATTTATATC

TTTATTAATGAAGCTTTTTTTACAGCATAGGCTGGGAATATATATTTATTCATTAATTAA

CCTATGAATAATTTTTTTTATTACCAGCAATATAAATCTTTATCAATGTCCAACGATAAA

CACTGAAATTAATTAACTTTTTCTTAATCCATAAATTGTTAAAATATTGTTTTTTAATAC

TCGTATAAGATTTTGTAGAAACTATTTTGGTAGTTACCTTGAAAATATTTAAATGATACC

TATAACTAATTTAATATTCTTACAATTGTTTAAGAAAGTAAAGAAAATATCAAAAGAAAT

TTGATATTTTATGAACTAATAAGACATTCACATTTCACAAATCAAATTTAATAAAATGTG

AAGATAGAACAATGTTTTGGTTGGGTGAGGCTATTGACATTTTAATTAATTCATCATAAC

AAAAAATGTGAATCATTTAATTTTTCTGTATTGGTAACAATTTTGTTTCTGCAGTTAAAG

CTTAAATTATGTTTCGTTTACTTTTTTCCGTTTAATTGATATAATCAGAAACATTACTTC

TACAAAATTTAATTTTCAATGATATTTAACAATACAAATAGTATTACCATATCAAATATT

GTGTAACAGAAAAAATAACAAACAAGTGGGCTCATAACTTTCTTTCTATTATGAAATTAC

ATACTTGCTGTCAGTAACGCTTGCAATTTCCAAACGAAGCTGTTAACATAAAACCAAAGG

CCAAAACTACTGCAGTTGCATAAATTGCCAACTCAACGTCAACGAGAAAGTTGGTAAAAT

TGATGTCTTTCCTCTAGAAGTGAACCTAGAGGGGCGTGACAGGCATTCTGACCCAAATAA

AAAATCCTTCCAAGTTCAGTTTTTTTTTTTTTTTATCTATTTGTAACAAAAATTCCAGCT

TTATATATTTATTGTAAACATCAAAATATTAGTAATATGTTTGATAAAAATAATATTAAT

ATGTTGGTTATTGGGCAGGTGCATATAAAAATCTAACGCGTGCTGGGTTCCCTCAAAGTG

TTCCCTTTTGGGTCAAAGGTTTGAACAGCTAATCCTAGAGAAAGGAAAATAATGGGTCTC

ATAAACGGGATGCATGCATAAATATACGTTTCAAATATTAAAGTATATGTTGTCGATATT

GTTAATTATGATTTATGAAGATTAATGTGGTGTAGTGGGTAACAGATGAGGTAGGAAAAA

ATGCATTTTTTTTTTCAGTTGTAACTTTTAAGGTCAGCTTGGACTGAACTAATACTTCAC

CAAACAATGATACCTTTTCCTTTAATCTAAGAAATAAAAGGAAAAAAAAAAACAAATAAC

ATTGCTGTAGTTGATTGCGTAGGCCCTGCTCCCATTCATCCTAATCCAAATTGTCTCAAG

TTGTGGATACGAAGGATATACTTTAAAATTCAAAACAATATGGCAACATCAAAGATGGAC

CCAATAAACCAATTATATCCATCAACCCTAGTTATGTAGATGCTTTTTCGGAGGTGCATA

TGAGAAAGTATAGTAGTAGTAGCTCAACAAATTAATGCTTTAGACCTCAAGCTAATGCTA

ATGGAGTAGATGAACTCATTTTCAATCTGGCATGTATAATTGAGTGAGACACTTGCTACT

TGATTAAATTGGAACACTTTTAGGCTTATAATTTCCACCCAAATTAGTTACATTTATGCA

CTAATCCTGCAAAACAACAAACGTTAGGTGGGCACGGCACCAATTTGAGGACTTCTTGTT

TTGTGGGGCGTTACCCTTTGTTGATATTTTGGGATGTTATCGAGTTTGATTCTTTTTACT

AATAACATTAACATTTTGTTT

>Glyma17g14280 chromosome:V1.0:17:11032223:11034223:1

TTATATTTTATTATACATCTTAATTTCAAGTTAATATGTTTAAATACCAATTATTTAATA

ATAAATAAATGTATAATACTTTTTAACTAAATAAAATTTATCTTTCAAAATTAAAATACA

TAAAATATAATACAAATTAAAATTAATAATAATAACTTTTAAAATACATAAAAAAAACAT

AAATAATCTATCATTAAAATAGAGTTCATTTTTTTATTATTTTTACATTCAAAATATTTT

CAGATATGCTTCATTAAATGTGCTTGAAGATGTTAATGTATTTACTTTTGATGACTCTCC

TTTTTAAATATCTTCTAAAATCGGGATGAGGTTCATTAAAAATTTTGGGTGTTGAGGCAT

CATTGATTGTTTGGAGTTAATTTATACTAAGGAAATTCACTATTCATTTTTTACCGTTAT

AAGGAAATGCAACGATTATGTGAAATGGTCAATTTTTTATTTTTTGTAATAACATGTAAC

GGTTAAGAAAATATAATCTGTCATTTAATTACTAAAATTGCATAATTTAATTTACTTTGT

CAAAGTAAGAGTACCTCCATTGGAAAATTCATCTCCATACACTTCCGTTAAATGTTTTGG

AATTGAGAAATATCTTGATATGTCTTCTATGATAGGTCTAAAGAAGAAAGTCATATTTAA

TTAATTGAGGGATAGAATTTGGAGGAAGATACAACACTGATCTAAAAGACATCTCCCAAA

ACCTAAAAGAAAAGATTTTATCAAGTCAGTTGTTCAGAGAAGTCCTTCCCGTTGCGTGAA

CACTTTTATGCTTCCATAAACTCTTCAGAAGAAAATTCAGAGAATGTTGAACTTTTTTTT

GTGAGAATTAAACAATAACTCAAACAAGGGGATAAACTAGCTCAATTGGAATTGATTGGC

TACGAGGAAAGAATATGAAGGTATGAGTTTCCAATATTTTTATGCTTTTATATTAGCCAT

ACCGTGGAAGCAAGGATGAAAATTTATTTCTGATCACGGTGCTATTGCTTCATTAGTTTT

AAAAACTAAATATTCATTAGTTTTAAAAACTAAATATTCATTAGTTTTAAAAACTAAATA

TTTTCTAAAGGATGACTTTTTGGATTCTCATTTGAAACATAACCCAAGTTACATTTGGCG

TAACATCTTCTCTTCACACGTGATGGTTGAATGATGGTAAATTGATCAATGTAAGGGCAA

ATCCATGACTTAGGGCAGGTGATTCCTCCTATATACAAACTCTTTTAATTTCGAGGTTGC

GAAATTTAAAGGCTAATGATTTGATTGATCCCTTGAATGGTAAATGGAATTCTGTTTTTA

TTCATGAGATTTTTCATGACACGGATGCACAAAGGCTAATGATATGCCACTATGTTTGCA

AAATGCTAAGGATAAGGTCATTCGGAAGCTTAGCAAGGATGACAACTTCATTATGAAATC

AACCTATTACCACATCATGGAAAATATGGTTGATAATGTTGATTTTCATGAGAATGGTGA

TTGGAACTCAATATGGCAGTTGGATGTTCCTCAAAAAATGAAGCTATTACTTTGGAGAGT

TGCAAGAGGTTGTCTCCCAACAAGAATGCGTCTGTCATCTAGGGGAGTTGTTTGTCCAAT

ACTTGTTCCTTTTGTGAAAACCCTGTTGAAATGGATTGACACACATCCATTGCATTTGTA

ATTATTTGCACTTCTGGTGCCTTAATGAAATTCATTGCTGATTAAATAGTGCCTTAATGA

TTTTGATTTCACTCCTAGTAGTATGCACTCCTTTATTACACATTGAAACGAGACGAGCAT

ATTTTTGTCAATAAATAAATAAATTTAAAACCAAATAAGAAAATAAGAAAAATAGTGTTT

TTGGTAAAGTAAAATCTTTTTCTTTTTTTTTTGGTAAAGAATAAGAAAACTAGTGTAATT

TGCTGAGATGTTCACGTTTTTTATTTTATATATAAATCTATTCACTTGAATCGTCCCAAA

TCCAATACAATCCTAGGATAC

>Glyma18g05480 chromosome:V1.0:18:4155147:4157147:1

GTAGGGATAAGTTAAAAAAAAAAAAAAAAAAGCCTTTGAACTTTACAAGTATTGAAACGA

TGTCGTCTATAGAATATTTGTTGACTTTTTTTCTTTTTTCCCTATTTTATTAGAAAAAAT

CTCTTTTATTATTATTATTATGATTATTTATGTACTTTATTAAAAAAAACTGTGTAATAC

TATATTCAAATTATCATATTCATATTTTTTTTCTGACAAGTAATTATCATATTCATATTT

AAAAGATAGTTTATATTACATTAAAAATACTATAAAGTCAACAAATATATAAAAAAAAAG

AAAAAATAGTTTTATCTATTATATTATATATTATTATACAAAATAAAAAATCTTTATCAT

TTTATTAGCCAAAAACATCTATTATTATTATTAAATATTTCTTTTTGTGCAATATTATAT

TTAAATTATTACGTTGATATATTTTTTTCTTAAGAGCAATTATCATGTTGATATTTAAAA

GATAATTTATATTGCATTAAGAATACTATAAAGTTAGCCAATAAAAAAATAATTTACATC

TATTGTATATTATTACTGCTCAATTTTCTTTTATTCCCTCTCTAACTTTTTTTCCATTTG

CTTTTATTTTTTTTAGATTTTTTATCTTCTATTTTCTAAAAACACACAACTCCTACCAAC

TCCTTTTCAATTTTTCCCTTCCTCTCATTATTATTACATATGAAAAAATATATTTATAAT

TTATCATATTATTATGATATTACATACTATTATCTATGTACTACGTACCGTATTAAATAA

TTTTTGCTTTAATATTATATTATAATTTTTTATTTTTTTAAATAATATGAATTAATATTT

TATTAATCTGTTAATACATATAAAATATATTTTATCCTTCTTAAGATACACATAATTCTC

TTCTAAATTTTTCGTTCCAAAACTTTTTTTCTCTAGCTTTTCTCTCTGTCTCTCTTTATT

TTAGAATATTTTTATCTTCTTTCCTCTTTCGTGTTTATTCTTATATATATTCTTTAAAAT

TAGATTACAAATATATTGTTTTAAAGTAATAGTGATTTTTAGCGGAAGGATGGGACGGAA

CGGATAGGATATATCTCACCCCAAATGATAATATTTTTATTTTAAAATATTATAAAACCA

TTTTTTAATTAAAATAATTTTACAAATATATATATTTTTATTAATATTTTTATAAGGTAA

ATATTTTATACTAAAAATTAATTTAAATTTAATTTAAAATATATACAAATATATTATTTT

AAAGTAGTAGATGTTTATAATGGTTCAGAATGGGACAAAATAAATATACCTAACATTGAA

CAAAGAGTTGGGGTGGGTACAACTTAATTATAAATTAATTAGCTAAATTTATTTTTATTT

TAAAATTTTATAAAACAACTTTTAAATAGAAATTGTATAAAATAAATTTAAATAATTTCA

TAAATAAATATTTCTTGATCAATATTTATATTATAAGGTTATTTTAAATGTAATTTAAAA

TATCTACAAATATATTATTGTAAAGTAGTAACAATTTTTTAGTGGACAAGAATGGAAAAG

GACAAATATATCTGATGATCCCGAATAGAGATGACCGTGAGTAAATTTTTTTATAAAATC

GTTAAAAAAAATATGTTTTTAACAGTAGTGAAAATCCTTGAGGGTTCTTAAACTCAAAAA

TCTTCATAAGATCAATTTTTGAGCTTAAGAATCCATTCCAAGATTTTCCATGAAATAAAT

ATGCTCTTAAAATTAGAATTATAAGTAATGTAAATAATAATAATATATTAAATTTTGAAT

TATAGTCTCTTGAGATTATGCTAAATAAATTAAGTATGCATTGCCTAGCCCAGTCATAGG

CTAATACGTAAATTTGACTCCAATCTGCAGCATTTTTTTAATACGTCCTCAAAAACAAAG

AAATATATTAATTAAATTCAATCATATACCAAATCCATAGACACTGCGCACAATACGTTT

CATTCCATCGATGGTTCGTTC

>Glyma18g05610 chromosome:V1.0:18:4216455:4218455:1

ATCAAAATTATAAGTTAGTGGTCATGTGATCACTTATTTAAATATGATTTAAGGTTAACC

TATTTTAATATGTTTTTGTAAAAGATTTATTTTTGGTATATAGTTTTTAGTAAAAGAATT

TATTAAAGTATGATTAATTCTATTCTTTAGTTGTTATATTTTAATTTGAGGGCAAAGCTT

GAAAAATTTAGTCCAAGTAAAGGATATAGTTGGAAAAACAAAAAGTAGTATATGGATAGT

GAAAAGGTTATTTTGAATCGTAGAAGTTACAACTAGATTTCCCCCTCAATATATGCTTAT

TATAAAAACGGATTGTTTTTGGCTCCAACTCCATTTGTTATTGGTCACTACCAAGGGGGG

TTCACACCTTTCTTTTTTTATTCGCAAATACCCTTTTTATGGAAATATAATTTTGTTGTA

TATTTATACAATGAAATTGTGTTTTCGTTATTTATTGAGAGAATGTGAATAAAAATAAAC

AACGAAAATAATATTTCGTTGTACAATATGCAATAGAATCATGTTTTCATTATTTTTTGA

AAGGGTGTGAAAAAAGAATAATGGAAGCATGATTGTGCTACACACTACACATAAAATTGT

GTTTCCTTTGTATACAGTATAACAGAATCATATTTCCATTATTTTTTGAAAGAGTGCAAA

AAAATAACAATGGAAATACAATTTCGTTATACACTATACAACATAATCGTATTTTTGTTA

TTTTTCGAAGGGGATGCAAAAAGAAAAAACAACAGGATTTTTTTTTGACTAAAAAATGGA

AGGTAAAATTGCATTTTTTGTCCTCCTAATTGTTTCCAATTTCGATTATAGATTCCCTTT

AAATTTATTCACGAATTTAGTTCTCCAATCATGTTCAATCCCGTAAATATGATCCTCAAG

TCCAAACTTAAACGTTGATTGTTACAAAGGAACATTGACTACCACGTATCACGTTCTGAT

TGGACACTGACAACAACAATATATTTTCGTTGTCCAATTAGAACGTGACATATGGCGGTC

AACGTTCCTTTGGAACAATCAATGTTTAAATATGGACTTGGGGACCACATTTACGAGATT

TGACATAATTAGAGGACTAAATTCGTGAATAAATTTAAAAGAAGACTAAAATTGAAATTG

GAGACAACTAGGGGGACCAAAAATATAATTTTGTCAAAACGAAACTACAATTTCATTACA

TGCTTCCCTCTTCTTCTTCCTTTACAAGAATAGATCTGCTGATGGTAGGGGCATGCGAAT

TAAGGCAAGGAGTTGCGATGGCGTGGTGGTGAGGATTCATGGTGTTGGCTGGGCTGGTGA

GGGGTCGCACACTCGCAAATCTGAAGTTGGGAATGGTGCACGATGCTGGTGGTGGTGTTG

CATTCAGATTTGATCGGTGAGGGGGTTGTGTAGTGAGATTGTGACGCAATGAGGGGGTCC

GTTTGAGGAGGTGGTGACGTGTTGAGGCCAATACCGCATGGTGCGATGCTGATGGGTTAG

GTGGGGTGAAAAGGTGAATGGAAAGAGGGAGGGATAAAAAGGTCTTTTCTTACGTTTGTA

GGTGCACTAACAAATGAGATTTGGGCCAATAGCAACTTCCATTATAAAGGATTTTTCTTT

GGGCACCATGCAGGTTGCTGGTACACCCAACATAACATGTGAAATTCCAGTTTTGCCTTT

TTATAAAACTGACTTTCACGTTATTAACACGACGCTCTAACCAACTATGCTAATAAACAA

ATTATGTTATAAAATAATTAATGTCGCTATATATAATACTAAAAAAATTTCTAATGTATA

TTTAATGCACATGTAAATTTACATAATAAAATTTGTGATAATTAATTTTTATCTAATAAT

TAATTTGTTTACATATATAAATATGAGCTTACGGATTATCAATATGTATAAGTTTTACAG

AAGGATAAAATTGGAAATCACATGTTCTGCTGGGTGTGTCAACAATTTGCATGGTGCCCA

AATAAAAACCCCATTACAAAA

>Glyma18g11520 chromosome:V1.0:18:10259654:10261654:1

TATACCCTCTAATTCAAGAGTGAAGAAACAAACAAGGGTGGGATGGAAATAGTACAATTG

GTCCGAGTGACTTACCCTCTGGCCTCTATTTCTAATTCAGAATTTTTTTTAAAAAATATT

TTTAATTAATAGAAATAGTACATTGACTTTTACTTCAGCTTTCTGCAAAAGCACACCGGA

GTTTTTTCCATTGAAGTAAAAAATAGACATACCAACAAAGCAACTACATTGATATATCCT

TGTTACCGAAGGAAAAAAAGTGTTGATATATTCTTGTTACCGAAAGGAAAAAAATTGTTT

AAAACAGAAAAAAAATCTATTTTATATAAAAGAAAATTTTAATAATGTGAAGATTTCAAG

TGTAAGAATAATTGGTGCTATGAGAGAAGACAAGTGATCACCCATCTCTTTAATGTGCAG

CCTATGGTTGTTGTATTTATACTGAAATTAATAGATTTGTGATAAAATCGAAACAAGTAT

CTCATCTTTAAGTCATTTTAAAATATTATTTTATGTTATTTTTTATTGCTAACCATTTAG

TAATTTGAAATTAAAAATAATAATTGACCAAGTTCAAGGTGTGTGTAATAGTTCTTCAAA

CCAAATTTCAAACATGAAATCCTCCCATTTGTTTCTTTTCTTTCACCAATTATTTATATT

GCTAGCAAAAAAATATAATAATATTTCACTTAGGAAATTAATTAATCATTTGATCATCTT

AAATTCTCTAATTTAATAGATCATCAAATATTAAAATAATTTTCTTAACATAAATTAGAA

TACTTGTTTATGTGTGACCCTATAGATTCAATACTAAGCCGGTAATATATTAATCAAATT

AATATATTAATTAAGATAGAAATCTAGCAACACTCATTAATGTTCAAATAGTATGAAGTA

GCATTTTACTTTCAAGAACCATTAGAAGAATAATATAATAATTTCTTTCATATTTATAGA

TCTGGGTTAACTCTAGAGTATAGTATTCTTTATAATGACTTTCTTCTTTCATTTAGTTTT

TCTGGCCTGGATACAATTTTATTTGTAGAGTTCGAACTCATTACCAAGATTTGATGGATT

CCTTCTTGATTAATCATTAATTCTACATGTATTTAATCATGTCCAATATCCATTCAACAA

GTGCGCTAAAGCATTAGGCGTCTAGAATCGAAACATAACAAATAACCTGTTAATTACTAT

GACAATCTCAAGTCAAAGAAAACTATTAAACTTATTCTTGAGAATTTTTTATTGACAATT

TATGGTAAATTTAACCATTAGAAAATCTCAATTGAGTTAGTTCAATGATGACATCTACAT

ATATATCATTTATATATGCAATTTAATAAATGAGATCTATTAATATTTATCCAATAAAGG

CCATTACATATATATTGATATATCCAGATTCACAATAATCTTACGATCAAGAACAATTTA

GATTAAAATTATAAATGACTTGTTTCTCATTATCATAATCTCTATCATGATAACACGCCC

CTAATTTTAATCAAGATAAAATTGACAACATGATGAATTGGATCTTGGACATACATATTT

ATCCCCAACAATCTCTCACTTGCACTAGAGCCCATCACTCATGTATTTCATTGCTAATTC

CAACTTATGTTCATCAAACTACTTTAGTTTTTAATTTTGAGTCTCCATAATGAGGTACAA

TCTTTAGTTCTTCTTAAGTACTTAAGAATGGTCTTTTCCACTTTCAATGCTCCCCACCAA

GACTTTCTTGATATCAACTTGTTACACCTAATGGATAAATGATATTAGAACATGTAAAAA

TCATGTCATATACTATAGCTCCCACTATGCTAGCATATGGTACTCTAGTTACGTATTCTC

TCACTTCATGAGTTTTAGGACAATCCTCCGTACTGAGAGTAACTCCAATTTCTATTAGCA

AATAGTCGCTTTTGGAGTTTTCATGTTATACCTCTTTAGGATGGTATTAATGTACCTAGA

TTGGGAGACACCGAACAACCT

>Glyma18g13077 chromosome:V1.0:18:12514456:12516456:1

ATCTCGTGACCTTGACAAAAACTGATGAATAAGCAATACAATTGTGTAAATAGCATGATG

AAAAATATGTTTTTCTAAACAATAACGCCCCATGTATCTTCTTTTTAAACAATAGCTCTA

CAGTTTGGAACATCTTTTATTTCTCCGTAGACCAAACTACCAAATTTGTTTTCCAAATTT

GGTGTGTCAAGAATCAAATGCTTGAAACCTAGCTAGGATCCCCATGTACTGGTAAACATA

TTTGAGAATATCCATACTTTCAAGTTTTCCTCCTATCGTAGATAGTGACATAAATTTTCT

GGATAGCTTACTATGAATATTCAACCATTCTGGAAAATTCTTTTCAGGAAAGCCTTCAAG

TTGCAACATTTTTAACTCTGAAGGCAAACTGATTGGAATATCACCGTACCTTGTGTCTGA

CACACCCCATGATATTTTGAGATGCTCATGTGATGACAGTTCTCTCAAGCTTTCAACCTC

TCCTTCTTAGATCACAGCCCCACTTCCTATATGTATGCTGAGTCGCTTCAGTCTTTCCGA

ATTTGCAGCAAGATCTGATATTCTGCAAGGAGTATTGCTCGAACTACCTAATGCAAATCC

CTTGAGTACTTCAAGCTTTGTCAACTTCTAAATCCCCTTGGGCATTCTGTCCAACAAGTA

GCATTGAGACAAATTCAAATGTCTGAGGTTTCTCAATGAAGCAATATTATCAGGTAATGT

TTCTAGATAATGACAAGCTTTGAGATCTAGAGTTTCTAGACTCTCAAGTTTAAGAATGGA

GCGTGGCAGCACAGATTTTGATGATATCCCACAAAAGCTAAGATGCCTCAAATGCTTTTG

ATTCCCTAACTGCTTCAGGAATTCTTCACTCTCCACTTCAACATGTTTTGGTGAACCATG

CAGCCAACGACCAAGTTTAAGCACCTCCAAATGTTCCATTTTGGCCATCCATTGGGGTCC

AAGATTAAAATAACTCGCACCAACAATATAAACAGCTTTACAATGGTTAGAAAACCCATC

ACTGAGTTTAACTTTATGTCTGTCAAGTGCTAAACAATCATACCTAGTATCATGGCCATA

AAATGAGGACCGATGTTATTTGTGAATAAGATCCAAAAAGCTGCACTTCATCATTTTGAT

ACAATGACCTCACCGACACTGGACGAACCCGAGGATGAATTTTACATTTACTAATCTCTG

GATAACTGTTAAAATTAACTCGAACAATTACTCCATAATCCACCAACACTTCAAACACAT

CCTCGCCATTTTTCTCTCCTGTTTGCTCTGTTGTATTTTCCACCAAACCCAGTCCATTCC

ACCAGAGAATTATATTCATTTTCTTTATAACACTTTCGGGCAAATGTAAGAGAGACAAAA

AGCAACGTTTTTGAACAGGATGAAGCTTGTTATATTCTACCTTAAACCTTTCCAAGGCCC

GACCTTTTGAAACTTGACTAAAGGCCCAAGTATATCGAAGTGGCTTCTCATCTGGAGCTT

CCATGCTTTTGATTGCACCTTCAGGCAGCAACTTTTGAGTTGAGATCCTTATTTTCTGGA

AAATGTGTCCCTCCTCTTCCGTCAACTTACGTATGTTTTGTTTGCGAATATAGCCATCAA

TAACCGCCAACGTGTCAAGGAGTTCTTCTTCATTCTTCTTCACCATCCAAAACAGGTCAT

TTATCTAGTCAACTCAGACTTCAAATCATTCAAATTCGCTTGCTTTTCTACTCTCACCAG

GCGCTTCAACAATGTAGGCACTGCTTTCATGGGATTTGTTCGAATTGACAGCTTTTTTTA

TCACTACTACAAAAAGTGTTTATTACGACAGCTCAACTATAACGGTTGGCCAAAGACCGT

TTTTGAAAAGTATTCAGTGGCAATATTGTAATAAATTATGAGTTTAAGGACGGTTATTTA

GAAAACCGCCGTAATTGAATGATGTACAAAGACGGTCTTCCAAACCCGTCTTTGTATTGT

TACACTTTTTTTCATTTTCTC

>Glyma18g52470 chromosome:V1.0:18:61063907:61065907:1

AAAAAAATAAAAACATGATGACACATTTAAATACTTTTTTTTTCTCGACACACATTTTCT

AAATCATTTTTAGAAAATGAAAAGAAAATAGATTCTCCTCTTCGTAAGTCAGATCATGCA

AGACAGATTCTGTATGTGGGACAAACCCAAGCTCCTTTATCCCTATACTTTCTAATATCT

GAAGCTCATTGAAAATTTCCTTAGCCATTGGGTGTGAACAAGAGAGCACAACTAAAAATC

TAAAAGCACTATTGAAATTAAGCTTTTCGTTACCATAAACTTGGGCCTTGAAATGTATAT

ATAAATATGAAGTAAGTTGAGCTAAAGACGTTTTCTTCATGTACCCGCATTTTTTCTTCT

ACACCCAGTATATTTTTTCTTCTACACCCAACATATTTTTGACCATTATAACATTGCCCT

TACGGATTGGTAATTCAATTCGAATTATTAAAAATATGTTTTACGAAATAATTTAAAATT

AATGATCAATACAGGTCTTAAACTTATGACTTTCATTCTAACCAATTGAACTAATATGTC

AATTATGCTAAAAAATAATTAATGTTGTTATATATAACACTAAATTTTCTAATTCATATT

TAATACACATAAATTTTACATAATAAATTTTGTAACAACTAATTTTGATCTAATAATGTA

TTTTTTTAATATATAAATTTTTATTAAACCTATAAATTTCATTTAAAATTTATATATGTA

AAAAAATATTATTAGATCAAAATTAATTGTTACAAAATTTATTATCTAAACTTATATGTA

TATTAAATATAAATTAGAAAATTTTGTGTTATATATAACAACGTTAATTATTTTTTAATA

TAATTGACTTATTAGTTCAATTGATTAGAATGTTGTGTTAATAATGTGAAAGTCATAAGT

TTGACACCTCTATGAGTCATTAATTTTAAATTATTTTGTAAATTAAAACGTATTTTTAAC

AATATTTATGGGTCATATTATATAGATTATCAATTCTTAAGAGTTCATACGCATCTGTAA

GGTCCATATGTATGATGCTTGTGGATTGTAAATGTTGAAATGGTAAAAAATATGATAGTA

GAAGAAAAAAGGATGACGCAGAAAGAAAACGCCAAGCCTAAGCAATACATGCATAGCTAC

AAAGTTGAATGAAGCCAAGCCCGTCAACTTTTGTACAGCTAAGCAAACACCCAAGAGACA

TGTGCTGGTTAGGCTGGTGCTTAATTTCCTTTGTCAATATCATGGAGGTTCGACAAATCT

TTTAGGCTGGTGCTTATCAAGGAATAGTTTATAAATAAATTTAGGTTAGAATGAGTCAAA

CTGATTTATTTCTATCTAAGAAACAATTGTTCAAATTTGTAAAATATTTCACACCCTTGA

TTAAACAACTGTTGATTTTAAAATTGTGTTTCAAGTTTTAAAAATCCAATATACAACTAG

CTAGTATTATAACATGAACTCAACAATCATGTGAAGAGTGAAGACATCTTCAAATAATTA

AGAAACGGTCAATAGTACAAGTGTACTGCAACTATATTCTTTTGGTTTCGTTGGGATATA

TTGTTATAAGTGGTTTGCTTTGGGTAGGTATAACCATACGTTTTCTATCTTTGATATATA

TATATATATATATATATATATAAAGCCATTATTTATGTTTTCTTTAATGATGACAGCTAG

CCATCCTTAAAGACTGAAAGGTTCCATAGAAAAAACAACAAGGAGTTTTGAAAAAAAGTG

CAATCTCGATGAACAACTTGCATTGCAACTTGTTTTCTCAAATGGTTGAGTCGTATAAGA

AAAAACAAAACTCAATTTACAGTCATTATTGAAAGTGATTCAATAAAAATTGAATGACAT

CATATTGTGAAGTCATGCGTTGCAGTGATTCATCATATTGAAAGTGATTCAATAAAAATT

GAACTTGTAAAGTACTTTGTTCTCATATTGATTATTGCAACTTCTTGTCCAAGCTCCATT

GTCTTGCTAGGAAGAGAAAAA

>Glyma18g52471 chromosome:V1.0:18:61065504:61067504:1

ACGTTTTCTATCTTTGATATATATATATATATATATATATATATAAAGCCATTATTTATG

TTTTCTTTAATGATGACAGCTAGCCATCCTTAAAGACTGAAAGGTTCCATAGAAAAAACA

ACAAGGAGTTTTGAAAAAAAGTGCAATCTCGATGAACAACTTGCATTGCAACTTGTTTTC

TCAAATGGTTGAGTCGTATAAGAAAAAACAAAACTCAATTTACAGTCATTATTGAAAGTG

ATTCAATAAAAATTGAATGACATCATATTGTGAAGTCATGCGTTGCAGTGATTCATCATA

TTGAAAGTGATTCAATAAAAATTGAACTTGTAAAGTACTTTGTTCTCATATTGATTATTG

CAACTTCTTGTCCAAGCTCCATTGTCTTGCTAGGAAGAGAAAAATGGCAACAAATGGCAA

GACACCTGCGATAGGAATCGATTTGGGCACGACATACTCATGCGTTGCAGTGTGGCGGCA

TGATCGAGTGGAGATCATCGTGAACGACCAAGGAAACAGAACAACACCCTCTTATGTTGC

TTTCAATAACACCCAAAGGATGATTGGTGATGCTGCCAAGAACCAGGCTGCTACCAATCC

AACCAACACTGTCTTTGGAAAAATACTAAACCCTTTAGCTTAATTATGCTTGCTTTAAAT

GGGAACTAAAATATATAAAGGTCATTTGCAATTTTTTCATTCATGAATATACTCACACGT

GCACACACATACTAGTATTGGTTTAACTTCTGAAATGATCAAAATCCTAAAGGGTTAATC

TAATGAAGCAAGATATAAGGTCTCACATTTGATAGGTTACTTGTTCGAACTTTTACTTGT

CTCTTTAGATCGAGTGAGATAAATGTAAGCTTTTTTTAGTATAACTTCCCACTAAAAAGC

ATTTTCCTTTGAATACACACTGCTTATGTTAAGAATGGCTTGGAGAGTATATATCTTGGA

ATATATCTTCTAAATTTTTAATATATCTTGGAAGGAGAAAGCTTCAATAAGACTAAGAAT

AAGTTTTTGAGAATGTGGAAGTTTTAAAGTGAAAAGTGTTTTCTACTTCTCCTTAATATA

AAAAGCATTTAAGAGGGTTGAAAATACAATATAGCACAGTTAGACATCACCAAAAAAATT

TGACCAATTAAATACAGCATATAATTGCTTTGTAAGTACGTACTAGTAGCAATGTAGCAT

CAATATATATATATATATATATATATATATATATATATATGTGGATGGTGAGGTTGTCTG

ACATCATATTTTTGGGAAAGACAACTCAGTATACCTTGCTACCGTTACATATGGTCATAC

TCTGGATTGGGTCTGAAGATCTTTGAATTTGTTCCCTCTTGTGCTTCATTAGCCTCCTTT

CTTGCTGATCTTTTCCACCACTCTTTATATATGATATAATGGCTATAATGTCGGTTTTTT

GGGCTTGTCTTCCCCCAAATATTGGGGAAAACTGATATATTCCTTTTTTCCCACGGATTA

AACGGGGATAGAAAAAATATATATATATATATATATATATATATATATATATATATATAT

ATATATATATATATATATATATATATATATATATATATATATATATATATATATATATAT

ATATATATATATATATATAAGATAATTTATAAGACATTTAATGTTATTTTAGGATTACTC

TATTAAAGAGAATACTACTGGTTATTCAAATTGTAACTATCATCTTGGTGGTTTGAGTAC

CGGTATTAGCTTATTAGGAATGAATTCTTAAGTAAATTATTGAATTTAAATTTTTTAGAT

AAAAAATGTGGTAAAAAATCCTACTAAAGATATAAATTTCTGACAAGCGTTAATTATCAA

CAAATTAGTAAATAATTTACATGAATATGGTGATGATAAAAAAAAGTTACTTAAATAGGA

AAGAAATAAAAGATGTAACGTATCGTGTTATGATTCATATGAGAAAAAGTTACTTATTAA

ATATATAAAGTGTTTTAATTG

>Glyma18g52480 chromosome:V1.0:18:61073242:61075242:1

TTAATTACAATTTTAAATTTACGTATTATCATTTAAAAATAAACAAATAAATTAACGTTA

ATTACTACTTTAAACTTGCTATACACGAATAATGACAAAGGTTGAAGACTTACTTCTCAA

GTCAATGAATACGGGACACGGGTTCAAAACACACTTCCCTTGTCTTTTCCCACGGATATC

AAATTCCTTTTTTCCCACGGACCACCGGCTCACGTAAATTCATCTCTTAATGTGGGTACA

TTAACTAGTCAAAGAATATTGTAAGAAAACAAGTTTTGTGTGACCCTCAATAAATTGATA

TGACGAGCACATAAATCAACCAAACACAAGTCTTTATGGGACCCTCAATAAACCGATACT

ACAAGTCTAATTTTATTAACTTATTTCATAGAAGATGATATGTGATTGTGAAATAGAGAG

ATGAAATTATTAGAGATTAAAGAATAATTTGTTAAAAAGGAAATATTATTGAAAATAAAA

TAACATAAAGTAACCATTCAAATGAATAAAGTAGAGATGAAATCACCCAAAGAAATCGCA

GTTATTGAAATGACTCAATTGAGTATATTTGAGATAAAATAAAAGTCATGTTTTTATTAC

TGCAAAAACTATTTCATTAAAGATAATTAAAATATATTATTTTTTATACTTTATATTTAT

TATTTCAAAATTAAAATAGAAAAAATAATAATATTCAATTGTATAAAAACAATTGTTTAT

GATATATATATATATATATATATATATATATAAGATAATTTATAAGACATTTAATGTTTT

TTTAGGATTACTCTATTAAAGAGAATACTACTAGTTATTCAAATTGTAACTATCATCTTC

GAGGTTTAAGTGGTATTAGGAATGAATTCTTAAGTAAATTATTGATTTTAAATTTTTTAG

ACAAAAAATGTAGTAAAAAATCCTACTAAAGATATAAATTTCTGACAAGGGTTAATTATC

AACAAATGAGTAAATAATTTACATGAATATGATGATGATAAAAATTTAAAAAGTTACTTA

AATAGAAAAGAAATAAAAGATGTAATGTATCATGTTATGATTCATATGAGAAAAAGTTAC

TTATTAAATATATAAAGTGTTTTAATCGTTCAAACTTAACAAGTTGGAGCGAAAAGTATA

GTCTTCATGAGCGAGAAGTATAGTCTTCGTGAGAAAAATTGTCAAAAATAAAATGGATGT

AGAAAAGAAAAAGATGATGCAAGAAGAAAACGCCAAGCCTAATCAATACATGGATAACTA

CAAAGTTGAATGAAGCCAAGCCCATCAACTTTTGTACAGCGAAGCAGATAACCACCCAAG

AGGCCTAATAAGAAAAGGTCTTAAACAATCACGGTAAAAACTATTTCCGTGGTCAATATT

ATGGAGGTTCGACAAATCTTTTAGCCTGGTGCTTAACAATTAAATTTGATGACAAGGAAT

AATTTATAAATAAATTTAGGTTAGAATGAGCCAAAATGATTTATTTCTAACTAAGAAACA

ATTGTTCAAATTTGTAAAATATTTCACACCCATGATTAAATAACTGTTGATTTTAAAATT

GTGTTTCAAGTTTTAAAAACCCAATATACAACTAGCTAGTATTTTAATATGAACTCAACA

ACCATGTGAAGAGTGGACATCTTCAAATAATTAAGAAACGGTCCATAGTACAAGTGTACA

GCAACTATATATATATATATAAAGCTACTATTAATGTTTTCTTTAATTACAGCTAGTCAT

TCTGAAAGGCTGAAAGGTTCCAAATAAAAAACAGCAAGGAGTTTTGAAAAAAAGTGCAAT

CTCGATGAACAACTTGCAACTTGTTTTCTCAAATGGTTGCGTGTTGTATATAAAATAAAA

TAAAAAAACTCAATTTACAGTCACTATTGAAAGTGATTCAATAAAAATTGAATGACATCA

TTATTGGGAAGTCATGTTGCTTAGTTATTATATGTATACTTTTATAGGAATATCTCAATC

ATTGTATTGTTTGAACAAGTG

>Glyma18g52610 chromosome:V1.0:18:61170753:61172753:1

TCAACGTCTCAACAACAAACGGAGTTGATACTATCTATGATGGAATTGTTTAAGTCAATT

CATATAGATTTTTAACCATAAAATTTGTGGGATAAATTAAAGGTAAGTTAAAAGTTGTAA

CATTCGACTCTTTTAAAATGAGTTATTTATTTTAGAGAGTAAAATATGCAATTTCAGTTA

TTTATAAAAAAAAATCAATAGTTAATATATTACTTGCAACATGCACAACAAATCATTGAG

TTTTCATCAAACATTGAATTTATTTATTTTACTCTTACTCGCTTTAAAAAATCTAAATTG

GTGTATATATAATAATTGCGGATATTTATTTTAATAATATATATATATATATATAATAAC

CGAAATGATTTTTTGCTTTTACCAAGCAAGTTTTATTTACTTTAAAAAACAAAACAATTT

TCTAAAATAAAAATAAAAATAAAAATAAAAATAAAAACACTTCCTTATTTTCCAACACAT

GCATGAAATGGACTATCATTTTCATTTGTTGGGTGAGACACCTTACCTAAAATGTTCCTT

CGATACCTAAAGAGTATATAACTATTAATATTTACTATATATATGTGTATTCTATCATTA

GCATAAATATTTCAAACTTTTTTTAAAAGGTTTTATTATGTTACTTTCTAAGTATAGACT

TCTTTACACATTTTTTTTTTTTTTATGTTTTGGATTGAAGAATTTGGAATAAGAAAAAAA

TATTCACTTGTGAACTGCCCTTTCTGGAACAATGCATACACAAATCTAGAAAAGTAATCC

AGAACATAAGTTTTTTGGATTACTTATATCAAAATTATAAATTTGGAATTACACAAGTTT

TTGTTTTGTAATCCAGATTTGATATTCCAAAAATGATTTTTACATTGTTCTTGAAATGAA

AAAATTATATTGGATCCAATATTTCAAAACCAACCTAATAACAATGCTAATTACTGCTTA

TATGCAGTGAGAATTCACGAAGTGAAGTTTTGAATATTATACCATTATGCAGTTCTGGCT

TTCAATTAAGCCCTTCCTCAGTAAAGAGGCCAAAGGATTTAAGCAAACAATGAGAATATT

TAACATAAAGCCCCAACTAAATATTCTTGTTGTATCCATGAGCTTTTAACAACAATAAGA

GTATTGTACTTGTCATGAACCAACCCTCCAAAAGATTTAACTGTTGGGCAAAAGCACATG

AACCAATTATCCAAAAAGTGCAATGATGGTGGTATATATTGTTGCATACAAGCACAAATG

GTTAAATCACATAAAGATGAAAGGAAGGAGGAAAAAAGGAAAGGAGGTGTGCTAAAGGGT

ATTTTTGTCTATTTTGGTTGTTTTAAGTTTTTACGGGGTGAAAGAAGCAGAAGCTGGGAT

GCAGGAAGCAATGGCCTAAGAAGTTTTAGGCCTGGGCTTATTTATACTCTTCAAAGCAGT

TTCTATTTATACTCTGTCGTTACTTGTACACTCCCCTTTTCCCTTTCATTTCCTAAATTA

CTCTCGTGAACCCTCTCAAAATTTCATAATATATATATATATATATATATATATATATAT

ATATATATATATATATATATATATATATATATATATATATATATAGATCATAAATGATGT

TTAGATTTAAATTAAACAATTTAAATTATGATGTATAGTTTTATTTTTTTTAATTACTGA

CAATAAATTTTTGAAAAATATTAAAAGATATAAAAAGGATAAACGACTAAGAAGATCAAA

TATCAAATATATAAGTAAAGATAAAAGAGAATACTTTAAAATGATATGATTGAATAAGAT

AATCAAAGATAATATGTATCAAATATTGAATTGATTTTAAAAGAATAAATAATTATTAAA

AATATAAAATCAAATATACATAGATAGAGATAAAAAAAAAGATTATAATTTTGGAATATG

ATTAAAAAAAAATCTTTTAACCAACTACACAGGTAGGTAATGTGTTTGTTGTAGAAATAT

ATTAAAAAACAAGATAATATA

>Glyma18g52650 chromosome:V1.0:18:61206577:61208577:1

GTGCATCTAGACCCGGCCAGAATAATAGAGCGTTAACAAAATGAACCGTTCATGTCTGTC

TTTGCTTAAATCAAAGATATTACTGCTGTCAAAAAAACAAAAAAATAATAATCAAAGATC

ATACTTTTATAACTAATTTAAATAAAAGCATAATTAATTGAAGTGACAGCACATGCGATT

GTGAAACGCACGATTGCAGTTATTAAAAAATTATATTAATTAATATTAATAGGACTTAGA

TATTAAGTTTATATTAAGATTGATTTTATCTGATATATGAGATGCACAATAAGAGTAACT

TTCATCTTCTCAACCTCCCCTTCTGATTTGATTTCCTCTTAGGGGCCGTTTTACAGGAAA

AAAAAAGATTTTTCATTTTCGGAAATTATATTTTTTTGGAATGAATAAACTTTTTTTTTA

CAATTGAAAATATTTAAATAAATTTCTCAAAAATCAAAGAATTTAGTATATTTTTATGAA

TTATTTAATTATTTTTCACATTATATAATTTAATAAAAAGTAATATTATATGTTTACGAT

AATAAAAGAAAAATTAAACTCAAGAAAATAAGATTTTCAGCTTACATCGTGAAAAATTAA

TTAAAAAAATATTTCCAAAAAACATCGTTTCTTATGAATATTATTTTTTTAAAAAAATGT

ATATCCAACATGAGAAACTAAGTTTCTCAACTTATAATTCTCAGGAAATAGAAAATTTCT

CTCCTATCAAACACCCCTATTAAAGATTAGGCTCAATTTTTATTCTTATTTCATTTTGGA

TTTCATTTTAAAATATTAATCAATATTTATTACATTGTATAAAATATTGTATATTAACCA

AAAAGAAGAAAGATATATTGTACTAAATTTTAATGACGTACTATTTATAATTCACATTAT

GTTTTATTTTTAATGATTCAAAATTTAAAATGATACTACCTAGTTCAAGATATAAAAATA

TCTCTAAATTCAAAGAATACATTGTTATGTTGTCTACTATAATTTTAATGCAAACCAGAT

CAAAATTCAATAGTCTCTTGATGTAACAAACTGTGTATTATACATGCGTGTTTATTATTT

GACGTACAATAATGAAATGAATCAAAACATGAAATTAATTCACAATGTGAGTTACTTCAA

ATGAAAACAAACACAAAGGATTGTTCATGTCTTTTAATTTTTTTTGTTTAAATGCTAGTT

AACCGTTATATATGATATAGTAAGTAAAATATTTAATTATAAGAGAATATATTCTTTATA

ATTTGAGAAAAGGAATTTATAATATTCTTTTTCCTTATTTCTAAATAAGTGATTCAACAC

AAATAATTAATATGTTGAAATTAATGTTAAATAATTTAAGTATTACTTAAATTTGATCTT

TAACGGAAATAATTTTTAATCATATTTTATTTACTTTTTAGTTGAATTTTGAATTAGTCA

AAAAAAATTTAGCGTGGTGAAACGAACAATTAAGATAAAAAAATTTAAATATCATAAGAG

ATATATACACTAAAAAAATATATACTAATGGTGAGAAAATATTTGTCAATAGTATTACCT

CCACTGATATCATTTTAATTAATTTAATAATATATATATATATATATATATATATATATA

TATATATATATATATATATATATATATATATATATTATACCAATAGACAATTTCATTTAT

TAACTCCCTTGTTAACAGATTAATGTTTTTTAACCACAACTTAAAGCTGGGTCGATCTTG

AATTCAAATATAAATATGGGTATTAATAAAAAGTAAACAACTTATGCACAAAAAGCAATT

TTCTTTGGCTTTTACTCTTAAAATGAAATAGTTTTATCTTTTTCGGTGGATCTTTTTCTT

TTTGTATCTTACAGTAAATGGAAACACACCGATTGTTCTTATATATAATTGCAAAATCAA

TATATTTAATGTATTACTTTTGAATACTAATTTGATTACTGAATAAAGTAAAGAAGAGAC

ATTTATTACACTCGGTTTCCT

>Glyma18g52760 chromosome:V1.0:18:61264485:61266485:1

TGGCCATTGGAAATCATTACTTAATTATTCGAAGTGATTTCCAATGGAATTTGGAAAACT

AAAAATCAGAGAGCATTCGTTAATGTTCTTTGTTTAAGTTGATAAGGTGTTAGAATAGAA

TCGTCATCCGTATTGAAGTTAAGTAGTGTATACATGTGCCCAGTCATAGGAAATGGCACA

AACTTAATTACTCCCATTAACTAGGGCAGGAAATTGGAAAACTAAAAGACTTTTAAGTTA

TCATTCAATGATTTTCCTTCTTTAAGTTGATAGGGCAATCTTTTATGTATGACAAGGAAA

TGATGATCCGATCAAGTTAGGTAGTTAGACTGAATCGTGGTTCACAGTTTCCTAACTTTT

TTTTGTTGGAATTTAAGAAATTGAATGGACGTAAATAACACATATTGTATTGTTTATAAG

ATTTAAGTAGACTAATTTAACTGCTCTTAAAAAAAAGTGGATTAATTTAACACGGATGAT

GATGGATGTCATTTTATAAGTAAAAAAATAATTTTTCATTTCTAAGAATTATGTTTCCTG

CAAATATTGTATAAGAATAGAAAACATAACTATAAAAAAAAAAATTACGTCATGAGAATC

TTAGATTCTAACCCTATCCTATGGAAAAGTTTTAGTGAAAAATACCTAATTTAAAAACAA

TTTCTAAAAATGTTAGATTCTTGTAAATGATTTTTAAAATACTCAACTTAAATTCTTGAA

AAACTAAAATTTTTCTACCAAACACCTTCTAAAGAAAAAATTAGTAGGATAACATGTACT

TGTACTAATATTAATTTTATAATTTATATATTTTTATATTATTATGATTTTGGCTGAAGT

TTTAGGTTGTGAGGAATTTGCTGCCCAAGCCCCAATTGCTCTTGGAATTACTCCAAATTT

GATAAGGTTCTTCCTTGCATCTTGTAAAGATTAAAGGCAATGATATCCTCTTTTCCTTTA

TGCGAAGAAAAAAGTGAAGGTTTACAGTGTAAACTCTTAAAAGTACATACTTTATTAAAA

AAAAAGTAATAAAATTGACTGTGTTTAACGTGGACTGATTTATTTTCTACCATTAAAAAT

ATCCCTTAGATTAATCAATAGTCTATTTTCTTATCATGGACTTTAAACACCAAATCCTTT

CTCCTTTCTTGCCGGCGATGCTTGTCCACCGTGCGCCTCCGCCGGAGATATATTCGGCAG

CGGACCCGCAACCGGAGACGATTTTCGATGGCGGCACCGCCACTTAAATTTTTCTCTCCG

TTTTTTTTTTTCCATTCCCTTCCTCTTCTCTGCTCCTCCCTCAAGCCTTTGTTCTGCAAC

ATTTTATTTCTCCTCCTTCGTTGACAACGGCTAGTTCTACGCTACCAGCGTCCCTCACCG

TCAGTCTAGATCGAAATCAAGTTCAAGGACGAACGTGTCGACACACCGCTTGCTCCTCCC

TCAACACTTCCGCACATTGTCAGCAAAGGCACCGCAGATTTGATCGTCGGCGGCGTGATT

AGCAGATCTGGATAGCCATGTAACGCGACGTTGTTCGCAGTTTTTGGTGTTTTTTTTTTT

TGTTTAACTGATTCTTAAGCTTTTAGTCCCCGAGACTCACTCTGTTTCACTATTTCCGGA

CTTTTTGGATTGTTTGTTTTTTCGTTACTTTGTCAATTTTTGCTTTGCTTGTCTCTGATT

TTTATCGTAGATCGCCATCCATTTTTTCTTCTTTATTTTTTGGCACGGAGATTGTTGGTT

TGGGTGGAGATAGTCAAAGAAAGAGAAGAAGGAAGAGGATAGGAGAAGAAAGGGTGGAGG

GGGGGGGGGGATACAACAAGAGAGTCACCGTCGTCGGAAGTCATGGTGGTTACCAGTCGA

AATTTGAGGAGCCGAGGAGCGAGAGAGAAAGAAGAAAAACATTTGGTACAACATGCCTAC

CAAATTACTGTGTACCTGAGATAAATCTAACCGTATAAAAAAGGCAAAATTACATTTTTT

CCCCCCTTCTCTACAATTTCG

>Glyma19g35560 chromosome:V1.0:19:43118308:43120308:1

CGTTGCTAGTTGGGTATGCATAATTTTCACGCTCGCAGATCTGAATTTTCCAAACCGTGG

GCCCGTGGCGGGCCTGACTCTCTTTCATCCAGCCCAATAAGCCACCTAACTTTTTTTCAA

ATTTATTTTTTATATAAATATTTTGACAAAAATATGTATCCAAATAAGATAATAAGATTT

ATTGAGACGTAAAAAAAAAAAGATAAGATTTACTGAAACATACATTAATAAATAAGACAT

TTTTTTACAGAATAAGATAAAATTTAATAAAATAAATGCTAAAAATGTATTTATTAAGAT

AATATTTTAAAAATATATTTCTGTTTGTATCTTAAAAAATAGTATTCCTATACACAAAGA

GTATTTTTATAAATAAAAAAGAAAGAAACAAAACAAAAAAGAGATATCAGAAGGAAGAAG

TGGTGACTGGCTGGCCCCTTTGCTGCCATCATATATTCATGTTATTATATGTTCATTTCA

AAAGTACTACTATTAACTTATTTAACAAATTACAACAACACCCCTTAAAACTTTGTGTAA

TACACACAACACTGTCTTTTGTATTTTCTTAAACTAATCTCCCTTTAAGTTTAAAAATAT

TAGCAAAAGCGCCAACCACTTGATTATAATAATATGAACAACCACTTTGGGTAATGTTGA

TAGCAAAGTTAGAGTGTGATTCATGGAGCATGGATGAAGCCTAGCCGAAGAGCCATCAAT

GACTAAACCGCAATGACCCCTAAGCACCCCACTGCAAGCTGCCTGACGAGACAATCTCAC

ATACGCATCACAATTCAATTTGAATAAGTCTGTGTATAAAACTAGCGCCACAACATGTGC

ATTGTACTATTTAGAATTTAGCATGAAATAATTGTTAAAGTAAAATAATGATAAGATAAA

ATTATCATGAAATGTTCAAATAGGAGAGATATTCAAAATTATAATATATGCGTGAAGCAA

TAATTTTAGAATAATGTAAATATTTAGTTATGAAATTTACATAGAAAATCAAAAGTCCCC

CTAATACAATAAGACCTATATCAAGATGCTAAAACAAATGCAAGTGCGCTTGTCTATACA

TAAGAAACTTAATCTTGCTAATAACCTCTATTACAGAAAATTGATAATCTTCAAAAATCA

GCTTGTTCCTAGATAGCCAGATGCAGTAGACTGTAATTGTTATAGTCAAACAACGTAGTA

TATTTTTTTTAACAGCTGATGTGGATCTACCCCTAATTAAAAAGTCAGTCATATACAGTA

AAGAAGTTAAACGTCGACAGAAAAGAGCCAAATCACGAATGTTAGCCCAAAGTTCATTTA

TAAGGAAGACAAATACACTCAGATGAAAAACACACTCATATTATTCATAGCCATTGAGAT

ACTTGGTCTGCCATTTATATTTACCATAAGTTGAAAATTAAAGCAAAGTTTTATTTAATA

TTATGTTGGATTGTTGCTGATCAATCGTTCACAGTAGTTTCTAGTTTCTGAATCAAAAAA

ACAAAATCACTGACACTAATTTGAACTAACGCTCTCTATTTATTTGAGAAGCATATCCAC

ATCAGTTGTAGAACCATATATAATTAACGATCCCTGGCATTTTTTTATTCGTTTGGCATA

AGTACCATTATTACTTCACTCTAACTCTATTCCACCGAAAGAACTAACAGTATAGCCGCG

TAAGTAAAATAACTTAGAATCGAATTATTCATATCAAATTTATAGAATAATATTCTTTTA

TTCCTCGATTATTTTATAATTTTTATTACATAGTAGTACTATTTCTTGTACCCAAAAAAA

AATTAGTTCTATTTCTTCGCTTTATAACTCTCGCAATCATATAAACTTCAGCCAAATTTT

GTCCCTATTAATGTTTGGTAAGGTAAAAGTAAAAGAAAACTTTGAATTTTAAAATTAAAA

CGCTAAAAGAAAAAATTCTGGAAATTATTTTTTCTCTCACTTCTTCTGATTCTTTTTTCA

ACTAAACATAAAATATTATTA

>Glyma19g44140 chromosome:V1.0:19:49646396:49648396:1

TGATTGAGTGTGTCGTCCGTACAATCACTAATTTTCATTTTTTGGCAAATTAAAAACTGC

TTATATCGCGTTGGCGGACGAGTGAGGTCCACGACACGAGTGCTTTGGTAAAGTTGGGTT

TTCGTTGGTGGAGATGTGAGTGTGACAAATAAAGGTTTTATTTTTTAAATAAACGAACGA

ACATCGTTTTAGTCAAGGCAAGAATATTCAAACGGCTCTTCTCAGTTGGGATTGGGATGG

AAGAAGAATTGCCGACTGAGTGTGAAAGTAACCTATAATTATAATGGGGTGTCATCACTC

ATCAATGCACAGTTTGTAGCAGAACAGAACAAAAACAAATGTGTCACATCTTATTTTAAC

AATTTCCTTTTATATTTTCGACGTTTTAAACTAGAGTTGAGCATGAATAGGATTCAACGT

TTGCACCCATTCCTGGCTCAGTTCGAATTTTAAGAATGTCACTGACCAATTATTTTTCTT

CATCACTTAATTTTATATTGTTTATATTATTCATTATTATTTTAGAATTACAGTTTGTGT

TCATAGCATTAATTTTATAATATGATATTATTTTAAGAATACAATTATGTAATATTTTAG

TTGCAAAATTCGAATAGTTACGAAAAATATCAATATCAACTTATTTTAAATGAATATGTA

AAAAATTATGTCTTTCACAACATCTACAACAAAATAAATTATATTCATTTGTTGTACACG

AGTTTATTTAATTTTTCTTCCTAACTAAAATGGCTACAGAAAAATAAATAATTTAACTTA

ATACATGAATGTAATTTATTGTTATCCATTAAATTGTGAAACATATTTTTAATATTTATA

AAACATTAGATATTCTTATCCATCTAGTAATCGGTGCAGCAAGTGAGGGTATCGCTGTAT

CAGGACTAAGGGTAAAACTTCCTGCACTCTCAGTGTTCTTATTATATTATGAAATGCAAA

ATGGATATTTTATAATACAATGGCAAATACTGAAGGTGAAGGCAATAGTGGTAGTACAGA

AGCAAAGTCTCATATGAATTATGCAATCTGTATCAGTTTTATGGGAAGACAAAATGAGAA

TTGCACAAGTTATGCTGGGTGCACCAGCAATTTCATGGTGTGTGTAGCAACACCTTTAAT

TTATCCTTAAAAAAACACTAGAGCCGAATTTGTCAAATTTCATTAGGGGAACTGCTATTT

ACACTAATCCACTCCCTTTTATGATTTGTTTTTCCAAAATACCCTTAATCTGCACTCTGC

TACCACCCCAACTTGGCAAATCCAATGCCCCTCTTCCTTAAAAACCCTAAGCTTCATTGA

CCTGCTGATACCTGCACCACGGGCTTGGATTCGAATTTTTGCATAAAGTCGCAATGGATG

GCAGCTAAATATCATGCACCAAGTAATTAATTAGTTAAGCAATTAATGTTGAGAGGAAAA

CTTGTAACTAGGTCACCAGATCACCTACGAGAGAAACTAAATAGCAAACAAAAAGAAATA

ATGATTATGGAAGATGTGAATAATTCCATACATTTAATTTCCAAATTCCAATATTATTGT

CATTTGCGGTGACAATTTCCTTCATTCTAACAAATCTATTTTTTGAACTTCTTCCACATT

TCTTTGATCTAGTTGGAGTAACTCTCACTGTCAGCTCCCATTTCACCTTTTCTAGAAGCA

ACAACAACGTTTTGCTTTACCAACCCAAAGCTGAATAACTATTTTTCATTTAGTATCTAT

GCATCAGGTCGGACTTGGCGGAGAACATCTACTATAAAACATTTGTGGAAAAAGGCAACA

AGAATTACAAAAAAGGGAGTGCAATTTTCAGGCTATTTTAAGAAAAATAATTATAAAGGG

TAGTGTATGAGTAACCAGAGAAAGTGAAAATAGCAATTTCCTTTCATCAGGGAGCCCAAA

AAACTGTCGGGCCGAATGGAGTCTATAGAGAATCTTATTATGTCCAAGTATCCAACGTTA

GCCAAATGAAAAAAAGGAAAC

>Glyma20g16070 chromosome:V1.0:20:22180179:22182179:1

TGTTCAGTATGATGCATGCACTTGATCTCAACTCTCAAAGGCAATATGGTACTATTCGTC

AGGAAATAGCCTAAACGTGTCCATGCAACACTCTCACTTAGGAAAATTAGGTAGCAAGTG

TCAAGGTCATCCTGTCGTGCATAGGCAACTCCCCCCATGGTGACCAGCCTGAGTCTCAAG

GGAGTTCCAAACTGAGTGACATGTCCCCAAGTACAAGTATTTCCCCTCATGAGAAACTAC

AAGTACTTACTGACAAAGTTTATATTATTTTCATGCAATATGAAGTATGAAACATAGGCA

CGATCAATGCACTGACCATGGATAATTAAAGATTCTAAGTCATCCCCCTCTAGAGATGCT

TAAAACTCTTTAACCACTATATTTTCCCTATCAGGGATATCCATCATGGTCACTACACCC

CCCATGTACATACACATCACACATCATCACAATGACATTTTCAACATCAACAATATCTCA

ATGTCATTATCAACATCAACATCATCTCATCTCAAAGTTATTCTCAACATCAATATCATC

TCATCTCAATGTCATTCTCAACATCAACATCATCTCATCTCAATGACATTATCAACAACA

ACAACATTATCTCATATCAACATAATCATTAATAACAACAACATCTCATATCAATATTAT

CATAAACATCAACATCGTCTCGTATCAATTATTATCAATATCATTTTCAATAACAACATC

ATTCTCTATCAACATTATCATAAACATCAACATCACCTTATATCAATTAATATCATCAGT

AAATCACATTCCGCATATACATACATATATAGTTTATGCCTGAGATTTACACTCCCCAGG

TCGTTAGACAACACAAGTCTAATAAAAACAATAATGTCATTTATCAATAACAAACGTATC

GCATCCTATTAGTAAAAAAAATTGTTTTTCTTGAAAACCAGCATGCAACAGGGACAGACA

TACATCCCCATAGTTAGGTTCTCTAACCCCAACTATGGTATCAAAAACCATAAATTACAA

TAAACTCCCCTCAACTATCGTGAGCTCTTTGTCAGTTCCTCTTTGTGTCGCTCAAAGACC

TCTCTCGTTCATGTTCACCAGTCCAAACACAACGTTCTATATACCAAATCAAAATAAATT

TAGTATAGATTTCAAAAATAAGGTTAATAACAACATTTGGAGCCAATTACCCCTATCAAA

ACATAAAAGGCTAAGGGGTGTTTCAGATTCTACTAAAAGGAGACGTCGTTTTGAAATTTT

GATCACGCCAATGTGACCAGAGTTTAGCGAAGGTCACAAAAATGACATCAATTTTATAAA

AATATAACATTTATACATCTCATTTTCAAGGGTTTTTCAAAGGAAGTGTAAAAACACCAT

ATTACAGTACCCAAAACACAAGAGACACTAAGAGAAGCTCAAACTGACTAGGAGAAAGAT

TTAGAAGTCAAGATTTCCTCAAAGAAACTACGAAAGAAAGGATTTGAGGAACTGTTCTCC

ACCGAATCCTTGAGGTGGATTCTGAGGATTCAGCTCCAATTAAAACATTCCTCTCAGAGT

GGTGGTTCGGTGGCAAGCAATGGCAGCTCGTGGTGGCCATCGATCGTCGTTGGTGGTGGA

GGAAGAGGTGTTAGGGCTTGGGTGGGCATTTGTTGAGAAAAGAGTGAAAAATCGTGTTTT

TCACGTTGAAAAACGTATTTATAATCAGTAAATCTTGCTTAGCGAGCTTGTTTGGCTAAG

TGGGAGTCCACTTTTGGCGCTAAGCGCGACTTTTCGTGCTTAGCGCAATTCCTCTCAAGT

TGAGATTTGTGTTGAGTGTGACAATTTGCGCTGAGTATAATTCCTTTTGTGCTAAGCACG

ACAATTCGCGCTAAGCACAAGGGAATTGCACTTAGCATGCTTCTCACGCTAAGCGAGAGA

TAAAAAATTGTTATTTTGAAAATCCCAACAGTAAAATCATGGGGACATGCATTGGGAACT

TGCATTCAAAAGTTAAAGATG
