# Supplementary material for: Genome-wide analysis and expression profiling under heat and drought treatments of HSP70 gene family in soybean (Glycine max L.)
Source: Front Plant Sci. 2015 Sep 25;6:773. doi: 10.3389/fpls.2015.00773 (PMC4585176; doi:10.3389/fpls.2015.00773)

The schematic diagram was derived from MEME. The ordering of the motifs of the HSP70 proteins in the diagram was automatically generated by MEME according to scores.


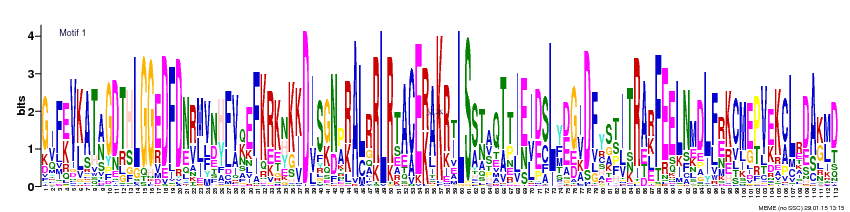

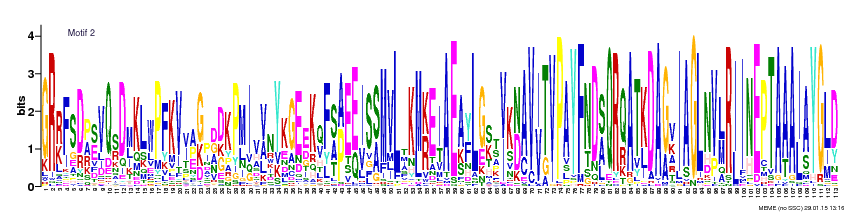

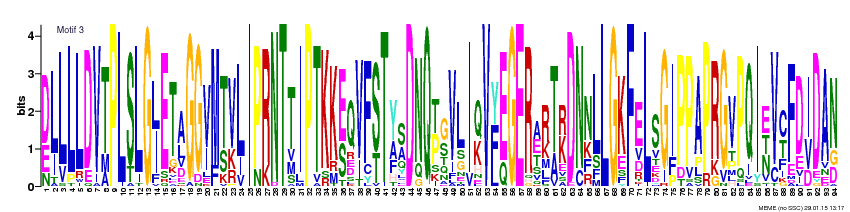


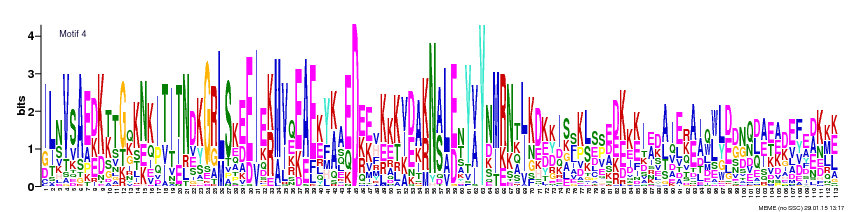


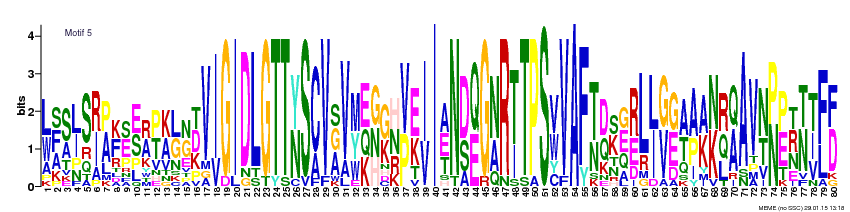


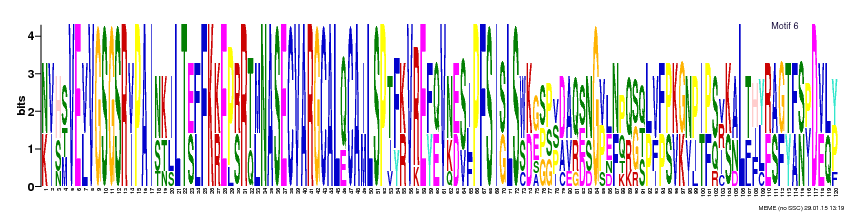


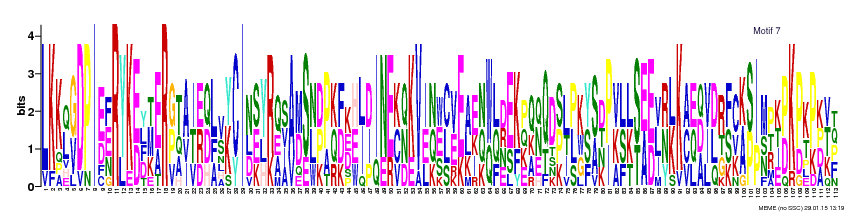


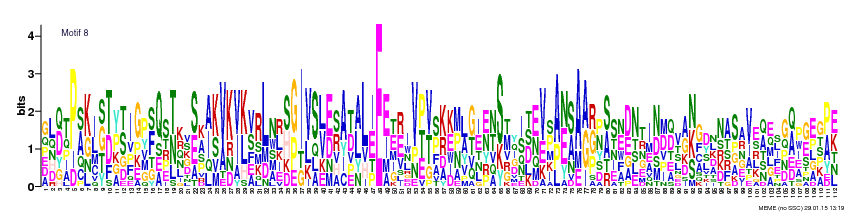


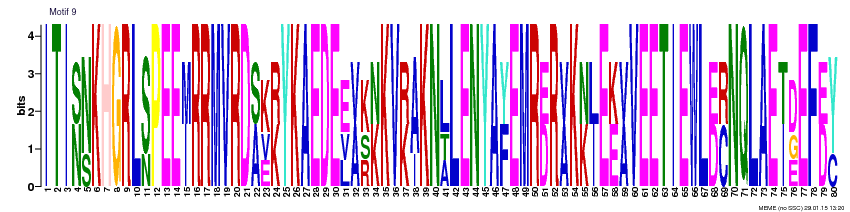


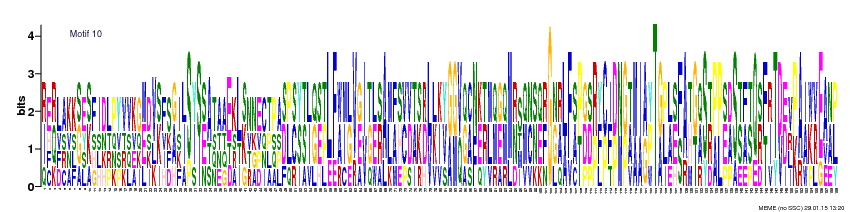


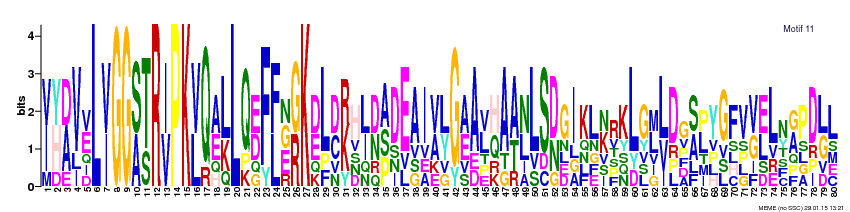


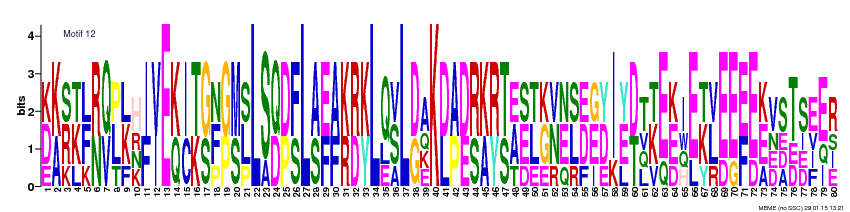


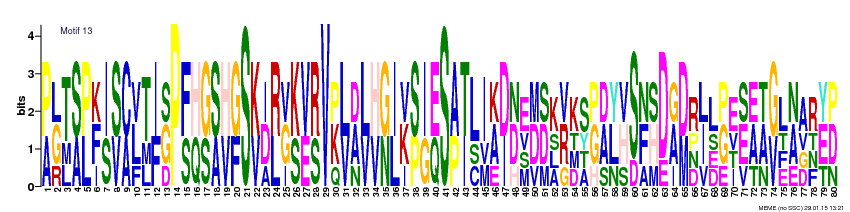


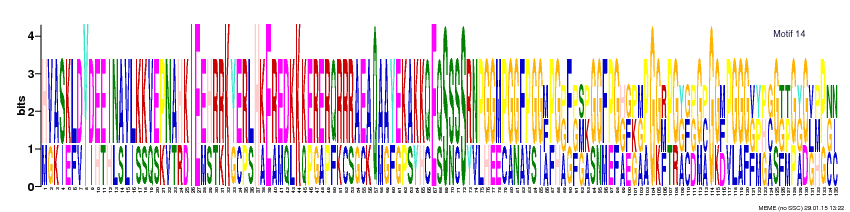


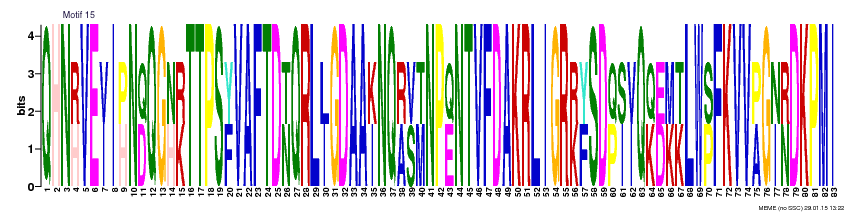

Supplement: Supplementary file 7 [file DataSheet7.DOCX]
